# Expression partitioning of homeologs and tandem duplications contribute to salt tolerance in wheat (*Triticum aestivum* L.)

**DOI:** 10.1038/srep21476

**Published:** 2016-02-19

**Authors:** Yumei Zhang, Zhenshan Liu, Abul Awlad Khan, Qi Lin, Yao Han, Ping Mu, Yiguo Liu, Hongsheng Zhang, Lingyan Li, Xianghao Meng, Zhongfu Ni, Mingming Xin

**Affiliations:** 1 Qingdao Agricultural University, Qingdao, 266109, China; 2 State Key Laboratory for Agrobiotechnology, Key Laboratory of Crop Heterosis Utilization (MOE), Beijing Key Laboratory of Crop Genetic Improvement, China Agricultural University, Beijing, 100193, China; 3 Northwest A&F University, Yangling, 712100, China

## Abstract

Salt stress dramatically reduces crop yield and quality, but the molecular mechanisms underlying salt tolerance remain largely unknown. To explore the wheat transcriptional response to salt stress, we performed high-throughput transcriptome sequencing of 10-day old wheat roots under normal condition and 6, 12, 24 and 48 h after salt stress (HASS) in both a salt-tolerant cultivar and salt-sensitive cultivar. The results demonstrated global gene expression reprogramming with 36,804 genes that were up- or down-regulated in wheat roots under at least one stress condition compared with the controls and revealed the specificity and complexity of the functional pathways between the two cultivars. Further analysis showed that substantial expression partitioning of homeologous wheat genes occurs when the plants are subjected to salt stress, accounting for approximately 63.9% (2,537) and 66.1% (2,624) of the homeologous genes in ‘Chinese Spring’ (CS) and ‘Qing Mai 6’ (QM). Interestingly, 143 salt-responsive genes have been duplicated and tandemly arrayed on chromosomes during wheat evolution and polyploidization events, and the expression patterns of 122 (122/143, 85.3%) tandem duplications diverged dynamically over the time-course of salinity exposure. In addition, constitutive expression or silencing of target genes in Arabidopsis and wheat further confirmed our high-confidence salt stress-responsive candidates.

Salt stress is becoming a particular constraint on global crop production, which is estimated to affect approximately 20% of irrigated land worldwide and will result in the loss of up to 50% of land by the middle of the twenty-first century^1^. High concentrations of salts result in a combination of ion imbalance and hyperosmotic effects and contribute to direct or indirect pleiotropic inhibition of crop productivity at the physiological, biochemical and molecular levels^2^,^3^. For example, salinity stress is expected to cause chloroplast damage, a decreased photosynthetic rate and increased photorespiration rate, accumulation of reactive oxygen species (ROS) and enzyme inefficiency^4^,^5^,^6^. Correspondingly, plants have evolved different mechanisms and have undergone a series of physiological and biological changes to counter these adverse effects, including salt exclusion and compartmentalization^7^,^8^. This phenotypic plasticity is driven by the activation or inactivation of various genes to alleviate or prevent ROS damage, re-establish ionic and osmotic homeostasis and resume growth under high-salinity conditions^8^.

To understand the molecular mechanisms underlying salt tolerance and improve the potential yield of crop plants, much effort has been devoted to genetic model systems. ROS scavenging was proved to be an effective way to alleviate oxidative damage, and the manipulation of related genes such as superoxide dismutase, ascorbate peroxidases and glutathione reductases, improves salt tolerance^9^. In addition, maintenance of Na^+^/ K^+^ homeostasis is another strategy for achieving enhanced salt tolerance, as excessive Na^+^ disrupts K^+^ uptake and cytosolic enzyme sensitivity^10^. It has been reported the regulation of low Na^+^ concentrations is primarily attributed to the activity of K^+^ and Na^+^ transporters and H^+^ pumps, and the SALT OVERLY SENSITIVE 2 (SOS2)-SOS3 protein kinase pathway, together with the Na^+^ transporter SOS1 drives the secretion and sequestration of toxic sodium ions in *Arabidopsis* cells^11^. The growth of salt-tolerant plants should resume when subjected to salt stress, although at a reduced rate. This process is potentially involved in the control of hormones and cell division related genes. For instance, a recent study supports the hypothesis that accumulation of abscisic acid is induced by salt stress, which then increases the expression level of *ICK1*, a cyclin dependent protein kinase inhibitor, ultimately hindering cell division^11^,^12^. Therefore, all of the three interconnected aspects mentioned above are necessary to achieve salt tolerance in plants, and these mechanisms are potentially helpful for breeders to improve crops with wide-ranging adaptability to salt stress.

However, salinity tolerance is a quantitative trait regulated by multiple genes^13^, and the salt responses of crop plants might be more complicated than those in model plants due to genetic variation because *Arabidopsis* is more sensitive to salt stress than most crop plants^14^. Thus, it is plausible to hypothesize that crop plants, particularly wheat, utilize a more complex system in response to salt stress than *Arabidopsis*. Several genes have been identified to play an important role in the response to salt stress in wheat; e.g., *SIMILAR TO RCD-ONE* (*SRO*) regulates ROS accumulation and scavenging to maintain their homeostasis by controlling the expression pattern of NADPH oxidase and NAD(P)H dehydrogenase, in conjunction with ascorbate-GSH and GSH peroxidase. Constitutive and attenuated expression of *SRO* in wheat demonstrated the necessity and sufficiency of this gene for enhancing salt tolerance^15^. In addition, Zhao *et al*., (2014) reported that ectopic expression of the wheat *allene oxide cyclase* gene, which is involved in the alpha-linolenic acid metabolism pathway, also resulted in improved salt tolerance *via* the accumulation of higher concentrations of JA^16^. However, the mechanisms by which genome-wide gene expression is regulated to control the response to salinity stress and ultimately adversely affect wheat production remain ambiguous, although Kawaura *et al*. (2006, 2008) showed that approximately 19% of the 32,000 wheat ESTs were up- or down-regulated by high salt concentrations in the roots or shoots at 1, 6 or 12 h using a microarray approach^17^,^18^.

Polyploidization has long been recognized as a driving force for the broad adaptability of plants to hostile environments^19^. For example, tetraploid *Arabidopsis* was reported to exhibit improved tolerance to salt stress compared with diploids through balancing K^+^ and Na^+^ concentrations^20^. However, the molecular mechanisms underlying the better accommodation to environmental constraints observed in polyploids remain a challenging, unanswered question. Recently, it has been proposed that partitioning expression of the homeologous genes potentially accounts for the enhanced tolerance to abiotic stresses observed in polyploid plants; e.g., one copy of the *alcohol dehydrogenase A* gene (*AdhA*) specifically responds to water submersion stress, whereas the other is up-regulated in cold conditions in allopolyploid cotton (*Gossypium hirsutum*)^21^. In addition, high-throughput transcriptome sequencing revealed that partitioned expression of homeologs occurs intensively in response to heat and drought stresses in allohexaploid wheat^22^. In this study, we focus on the extensive identification of salt-responsive genes and examine their distribution pattern on the wheat chromosomes. Moreover, given that allohexaploid wheat contains three subgenomes, we also attempt to determine the expression partitioning of homeologs in response to salinity exposure over time.

## Results

### Qing Mai 6 (QM) Exhibited Better Performance Compared with Chinese Spring (CS) under High-salinity Conditions

**Figure 1:**
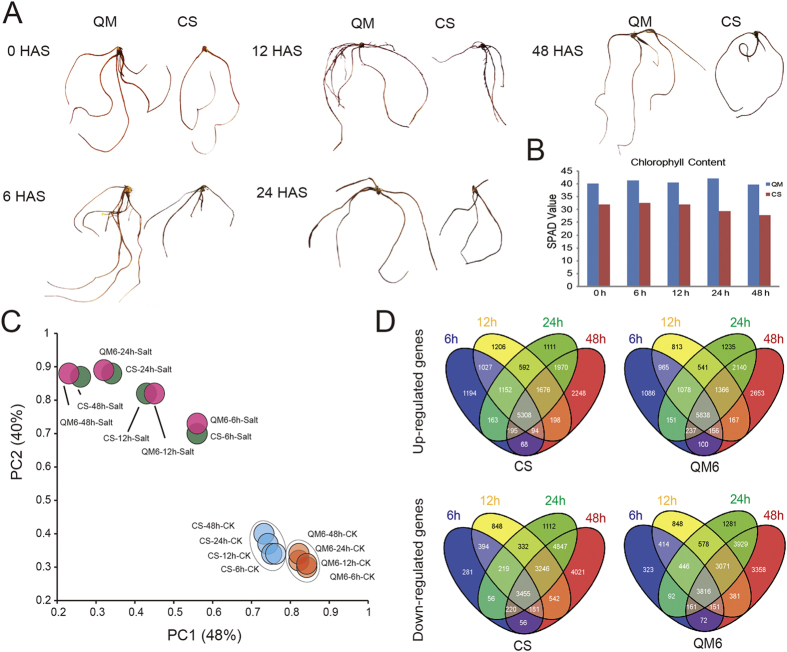
DAB staining, chlorophyll content measurement and comparative analysis of transcriptome profiles of wheat seedling root under salt stress. (**A**) DAB staining of root in QM and CS after salt stress at different stages. The staining color of root was darker in QM than in CS at 6, 12, 24, and 48HASS. (**B**) Chlorophyll content measurement of leaf in QM and CS before and after salt stress. The chlorophyll content is lower in CS than that in QM at 0, 6, 12, 24, and 48 HASS after salt stress, but no difference was examined before and after salt stress in neither CS nor QM. (**C**) Principal component analysis (PCA) of mRNA populations for QM and CS before and after salt stress. Principal components (PCs) 1 and 2 account for 48% and 40% of the variance, respectively. PCA plot shows two distinct groups of mRNA populations. Group I: CK for both QM (yellow) and CS (blue) at 6, 12, 24 and 48 HASS; Group II: Salt stressed QM (red) and CS (green) at 6, 12, 24 and 48 HASS. (D) Venn diagrams showing overlap of up- or down-regulated genes in response to salt stress at 6 (blue), 12 (yellow), 24 (green) and 48 HASS (red). QM: Qing Mai 6, CS: Chinese Spring.

QM is a common wheat cultivar in the Huang and Huai River Wheat Zone of China and has developed a widespread reputation for outstanding salt tolerance. Hydroponically grown QM exhibited a stronger root system and decreased H_2_O_2_ accumulation compared with the salt-sensitive cultivar CS at 6, 12, 24 and 48 hours after salt stress (HASS) with 150 mM NaCl, as shown by 3,3′-benzidine (DAB) staining (Fig. 1A). However, there was no difference in the leaf chlorophyll content after salt stress in either QM or CS, although a higher leaf chlorophyll content was found in QM compared with CS (Fig. 1B). Therefore, we next focused on the differences in the salt responses of the QM and CS roots by comparing their transcriptomes after a time-course of salt exposure.

### Transcriptome Sequencing, Read Mapping and RNA-seq Data Processing

To investigate the transcriptional reprogramming of wheat roots in response to salt stress, we performed deep sequencing of 10-day wheat roots subjected to salt stress at 6, 12, 24 and 48 h, together with the respective controls for both QM (salt-tolerant) and CS (salt-sensitive), using the Illumina sequencing platform. In total, 109 G high-quality adaptor-trimmed paired-end reads were produced from 16 libraries, with an average of 27 million reads per sample. Read quality was examined using FastQC software (www.bioinformatics.babraham.ac.uk/projects/fastqc/), after which the high-quality sequenced reads were mapped to the wheat reference genes released by IWGSC using the Bowtie2 pipeline^23^ allowing two mismatches. Approximately 66% (~43–73%) of the sequencing reads were mapped to the reference genes, and only the uniquely mapped reads (~37–60%) were retained for further expression analysis^24^ (Supplementary Fig. S1). Finally, we identified 36,804 genes that were up- or down-regulated in wheat roots under at least one stress condition compared with the controls (fold change ≥ 2 and false discovery rate (FDR) adjusted *p* < 0.01). Principal component analysis (PCA, Fig. 1C) showed the global mRNA populations of the salt-treated samples deviated greatly from those of both the CS and QM controls. A total of 5,308 and 5,838 up -regulated genes were identified in all four stages after salt stress in CS and QM, accounting for 29.16% and 31.51% of the genes of these cultivars. Correspondingly, the number of down-regulated genes in CS and QM is 3,455 (17.44%) and 3,816 (20.17%), respectively. Among which, 1,194, 1,206, 1,111, 2,248 genes in CS and 1,086, 813, 1,235, 2,653 genes in QM were specifically up-regulated at 6, 12, 24 and 48 HASS, respectively (Fig. 1D).

### The Salt-responsive Genes of CS and QM Were Enriched in Distinct Functional Categories

Based on the global comparison of transcriptomes, we identified thousands of stage-specific salt-responsive genes in CS and QM, respectively, which might suggest that distinct functional categories are enriched in the two cultivars. To confirm our hypothesis, we performed separate Gene Ontology (GO) enrichment analyses of stage-specific up-regulated genes in CS and QM that were responsive to salt stress at 6, 12, 24 and 48 HASS (Fisher’s exact test, *P* value ≤ 0.01). Consistent with our expectation, significant differences in the enriched GO terms were found between the two wheat cultivars in the time-course comparisons, although enrichments in the “post-embryonic development” (GO:0009791), “protein modification process” (GO:0006464), and “lipid metabolic process” (GO:0006629) categories were shared at all stressed stages in both cultivars. The CS genes that were specifically up-regulated at 6 and 12 HASS were enriched in the “cell death” (GO:0008219) and “jasmonic acid biosynthetic process” (GO:0009695) terms. In contrast, the corresponding QM genes were enriched in the “cell growth” (GO:0016049) and “response to abscisic acid stimulus” (GO:0009737) terms. More interestingly, QM genes were particularly enriched in “potassium ion transport” (GO:0006813), “establishment of localization” (GO:0051234) and “response to salt stress” (GO:0009651) terms (Supplementary Fig. S2). At 24 HASS, the enriched GO categories appeared to be similar to that of previous stages for CS; however, QM showed two significant alterations in the “ion homeostasis” and “cation homeostasis” categories. Furthermore, the functional category of “response to stress” was not enriched in the CS up-regulated genes at 48 HASS, and was replaced by the “RNA metabolic process” term (GO:0016070), whereas QM still exhibited higher enrichment in the “response to salt stress” , “ response to abscisic acid stimulus” and “response to osmotic stress” categories compared with the controls (Supplementary Fig. S2).

### Intensive Partitioned Expression of Wheat Homeologous Genes Occurs in Response to Salt Stress

Allohexaploid wheat harbors three subgenomes (A, B and D, respectively), and exhibits improved tolerance to abiotic stress compared with diploid and tetraploid wheat^19^. Although the underlying mechanisms remain largely unknown, the expression partitioning of homeologs is likely to play a crucial role in the response to different environmental stimuli^21^,^22^. To examine the partitioned and temporal expression patterns of wheat homeologs subjected to salt stress, we first identified 4,780 triplets (14,340 genes) to distinguish the origins of their homeologs, which included exactly one representative member from each subgenome, and then quantified their transcript abundance according to the A-, B- or D-specific reads^22^,^25^.

In total, 65.2% (3117/4780) and 69.5% (3322/4780) of the homeologous locus derived reads fundamentally deviated from the expected ratio of 1A:1B:1D (Chi-squared Goodness-of-fit test, *P* value < 0.01) under normal conditions in CS and QM, respectively (Supplementary data sets 1). Then, the candidate genes were further narrowed using more stringent criteria, where the maximum expression level was required to at least 1.5-fold of the minimum expression level (Expmax/Expmin ≥ 1.5) in terms of the SNP-associated reads that mapped to a homeologous locus. Finally, approximately 50.1% and 55.4% of the triplets exhibited biased expression patterns among the homeologs in the untreated CS and QM samples, whereas the proportions increased to 59.9% and 62.6%, respectively, after salt stress (Supplementary data sets 2).

**Figure 2:**
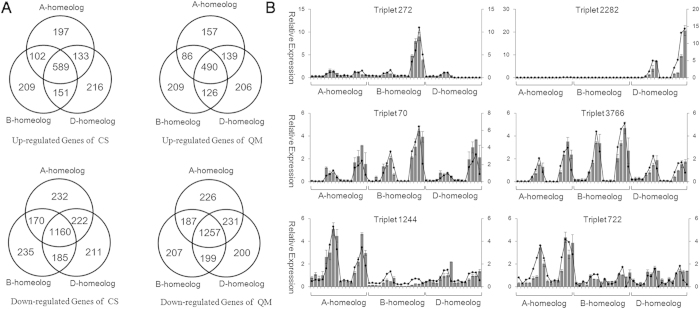
Comparison of salt stress responsive A-, B- and D-homeologous genes. (**A**) Venn diagrams showing salt responsive homeologous genes in A-, B- and D-subgenome, respectively. (**B**) Validation of expression partitioning of homeologous genes using qRT-PCR. The bar charts and curve charts represent homeologous genes expression profiles analyzed by qRT-PCR and RNA-Seq data, respectively. Gene expression values obtained from qRT-PCR and RNA-Seq were normalized and scaled to comparable level. The partitioned expression of homeologs in Triplet 272, 2282, 70, 3766, 1244 and 722 validated by qRT-PCR exhibited similar changing trends with RNA-Seq data.

Among 4,780 triplets, 3,615 and 3,604 showed differential expression of at least one homeolog in the response to salt stress in CS and QM, respectively, based on the criteria of a two-fold change (Fig. 2A). Specifically, 485 (260), 824 (400), 553 (1,184) and 449 (1,547) A-homeologs; 495 (252), 837 (385), 615 (1,142) and 471 (1,540) B-homeologs and 557 (237), 875 (360), 572 (1,143) and 487 (1,547) D-homeologs were up-regulated (down-regulated) at 6, 12, 24 and 48 HASS in CS, respectively, whereas the corresponding numbers for QM were 606 (196), 499 (925), 483 (1,404) and 447 (1,524) for A-homeologs; 636 (195), 507 (895), 489 (1,346) and 478 (1,456) for B-homeologs and 666 (189), 558 (894), 490 (1,400) and 490 (1,524) for D-homeologs, respectively (Supplementary Fig. S3). Among the salt-responsive genes, 2,537 (63.9%) and 2,624 (66.1%) triplets showed an unequal contribution to salt tolerance between the homeologs under at least one stressed condition in CS and QM, respectively, among which approximately 2,115 (53.3%) and 2,319 (58.4%) triplets showed significantly biased expression patterns before salt stress. Furthermore, the expression partitioning of homeologs in response to salt stress presented cultivar-specific and temporal patterns. Interestingly, more homeologs were up-regulated than down-regulated in the salt-sensitive CS cultivar (403 vs. 187), while the opposite situation was observed in the salt-tolerant QM cultivar (180 vs. 430) (Table S1). In addition, these differentially expressed homeologs exhibited dynamic patterns at 6, 12, 24 and 48 HASS, with more up- or down-regulated homeologs being observed at later stages than at earlier stages in both CS and QM.

To further confirm the expression partitioning of the wheat homeologs and their altered responses to salt stress at 6, 12, 24 and 48 HASS, the homeologs of six triplets (Triplets 70, 272, 722, 1244, 2282, and 3766) were examined through qRT-PCR analysis using homeolog-specific primers that we validated *via* nullisomic-tetrasomic line detection in a previous study^22^. The partitioned expression of homeologs resulting from the qRT-PCR analysis was consistent with the observation made based on RNA-seq data (Fig. 2B). Specifically, the A- and B-homeologs of Triplet_2282 (UDP-Glycosyl transferase superfamily protein) were completely silenced in all samples, while the D-homeolog was particularly induced by salinity in both CS and QM. However, the increase in the expression level in QM was significantly higher than in CS (Fig. 2B). The expression levels of the A- and D-homeologs of Triplet_272 (SNARE-associated Golgi protein family) were quite low at all stages in both cultivars, but the B-homeolog was specifically up-regulated at 6, 12, 24 and 48 HASS in QM, but not in CS. This is reminiscent of the pattern observed for its homolog in *Arabidopsis*, where constitutive expression of *AtSFT12*, a Golgi SNARE protein gene, leads to tolerance to high concentrations of NaCl, whereas loss-of-function of *AtSFT12* results in sensitivity to salinity^26^. In addition, the partitioned expression of Triplet_722 and Triplet_1244 shared similar patterns, as the transcript abundance of the B-homeolog was fundamentally up-regulated after salt stress, but the A- and D-homeologs exhibited no obvious responses to salt stress. The expression levels of the three homeologs of Triplet_3766, annotated as a sodium/calcium exchanger family protein, were all significantly up-regulated at 6, 12, 24 and 48 HASS compared with the control, but the expression level of the B-homeolog showed much stronger alterations in response to salinity compared with the other two homeologs.

### Characterization of Hotspot Regions on Wheat Chromosomes Encoding Similar Salt-responsive Genes

**Figure 3:**
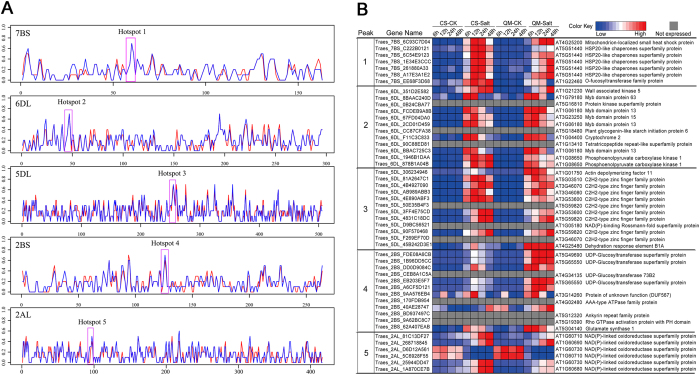
Chromosome distribution of salt stress responsive genes in CS and QM. (**A**) Distribution of salt responsive genes in CS and QM was analyzed along each chromosome by using a sliding window algorithm. Line charts show the percentage of salt responsive genes in each window, red and blue lines represent CS and QM, respectively. We observed salt responsive genes displayed a biased distribution and formed salt responsive hotspots. Pink boxes marked five representative hotspots. (**B**) Salt stress responsive genes contained in the five marked hotspots. Heatmap shows expression profiles of these genes in responsive to salt stress, and wheat genes were annotated by BLAST searching against Arabidopsis protein database.

To examine whether the salt responsive genes are randomly distributed on the wheat chromosomes, we mapped 69,658 genes expressed in the roots to the reference genome released by the IWGSC and compared their overall expression levels before and after salt exposure. Surprisingly, the arrangement of salt-responsive genes on wheat chromosomes was not random, and a number of the salt stress-related genes were closely clustered (Fig. 3A). To identify the high-confidence regions containing the tandemly arrayed salt-responsive genes, we examined the positions of all of the expressed genes along the wheat chromosomes, and characterized 143 hotspots in the wheat genome that contained at least three consecutive salt-responsive genes (Supplementary data sets 3,4 Supplementary Fig. S4). Further investigation demonstrated that these regions were unevenly distributed in the three subgenomes, with the D genome (57) exhibiting more salt-responsive hotspots than the A (36) and B genomes (49). Moreover, homeologous group 2 (2A, 2B and 2D) presented the most hotspot regions among all seven homeologous groups, occupying approximately 27.0% of the genome (Supplementary Fig. S4). More interestingly, functional annotation revealed that all hotspots, except one, contained at least two genes which encode similar proteins belonging to one family, indicating tandem duplication is one of causes resulting in salt responsive hotspot formation. For example, five HSP20-like genes (Hotspot1) and five Myb family genes (Hotspot2) were arrayed consecutively on chromosome 7BS and 6DL, respectively, and displayed up-regulation trends after salt stress in both CS and QM (Fig. 3B). In addition, nine C2H2-type zinc finger family proteins (Hotspot3) and four UDP-glucosyltransferase super family proteins (Hotspot4) were also tandemly distributed. However, only seven out of nine C2H2-type zinc finger family proteins were simultaneously up-regulated after salt stress in both CS and QM, while the other two exhibited no expression in all 16 transcriptomes, indicating that subfunctionalization, neofunctionalization or pseudogenization might have occurred during the gene duplication process. Similarly, among the four UDP-glucosyltransferase genes, Traes-2BS_CEB8A1C5A was not expressed in the examined samples. Nevertheless, it is worth noticing that, unlike the C2H2-type zinc finger family genes, the expression levels of the other three UDP-glucosyltransferase genes were higher in QM than that in CS at 6, 12, 24 and 48 HASS. Moreover, six genes encoding NAD(P)-linked oxidoreductase super-family proteins (Hotspot5) that are co-located on chromosome 2AL showed differential expression patterns before and after salt stress. Among these genes, four NAD(P)-linked oxidoreductase genes (Traes_2AL_81C13DF27, Traes_2AL_25944DD47, Traes_2AL_1A870CE7B, and Traes_2AL_268718845) exhibited a higher expression level after salt stress in both wheat cultivars, although there was some variation between CS and QM. Unexpectedly, the other two genes (Traes_2AL_D6D12A561, Traes_2AL_5C6928F55) showed contrasting expression patterns, where the expression levels were down-regulated compared with the controls when subjected to high salt concentrations. In addition, we identified another 41 hotspots with at least one member that showed no response to salt stress, likely due to pseudogenization during wheat evolution and polyploidization events.

### A Subset of Candidate Genes are Necessary and Sufficient for Salt Stress Tolerance

**Figure 4:**
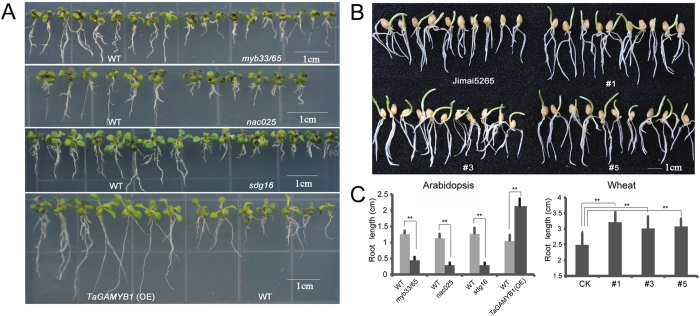
Analysis of salt sensitivity in Arabidopsis wild-type (Col-0), mutant of *mya33/65, nac025* and *sdg16*, overexpression line of *TaGAMBY1* as well as wheat *TaRSL4* overexpression lines and its wild-type Jimai5265. (**A**) Analysis of phenotypes of the wild-type, *mya33/65*, *nac025* and *sdg16* mutants, as well as *TaGAMBY1*overexpression line on salt mediums. The seeds were first planted on horizontal mediums for 5 days, and then their seedlings were transferred to vertical mediums with 100 mM NaCl. Photographs were taken at 10 days after the transfer. (**B**) Analysis of root length of wheat *TaRSL4* overexpression lines (#1, #3 and #5) and its wild-type Jimai5265 seedlings. (**C**) Relative root elongation of Arabidopsis wild-type (Col-0), mutant of *mya33/65*, *nac025* and *sdg16*, overexpression line of *TaGAMBY1* as well as wheat *TaRSL4* overexpression lines and its wild-type Jimai5265. Data represent mean ± Std (standard deviation) of the relative root length of seedlings.

The RNA-seq data provided us with a list of candidate genes that respond to salt stress, including a large proportion of TFs. To determine the biological relevance of these candidate genes, we first compared the performance of WT and mutants *Arabidopsis* plants in which the wheat homologs were silenced in the presence of high NaCl concentrations in MS medium. The RNA-seq data demonstrated that the expression levels of Traes_4DL_F0BE36C9D.1 and Traes_3B_BB32DAA31.2 (homologs to *Arabidopsis AtNAC025*) were dynamically up-regulated after salt stress in both CS and QM, but their read counts and fold changes were markedly higher in the salt-tolerant QM cultivar compared with salt-sensitive CS cultivar. Accordingly, the *Arabidopsis nac025* mutants grew more slowly and were weaker compared with the WT plants after exposure to salt stress, indicating that *nac025* was necessary for improving salt tolerance (Fig. 4). In addition, the read abundance of a wheat histone-lysine N-methyltransferase homologous to *Arabidopsis AtSDG16* was also increased in response to salt stress in CS and QM, and, consistent with our expectations, *AtSDG16* silencing resulted in shorter roots in the mutants compared with WT plants in MS medium containing 150 mM NaCl. Similarly, the transcriptional abundance of a wheat MYB gene (homologous to *AtMYB33*) was significantly increased under salt stress. Interestingly, *Arabidopsis myb33/myb65* double mutant seedlings (*AtMYB33* and *AtMYB65* were functionally redundant) exhibited higher salt sensitivity compared with WT plants, whereas overexpression of the wheat homolog (*TaGAMYB1*) resulted in enhanced salt tolerance in terms of root length compared with WT plants (Fig. 4). However, mutation of another *Arabidopsis* MYB transcription factor, AT1G08810 (homolog to Traes_4AL_7800B74E3.2), whose expression level was not altered in response to salt stress, did not lead to obvious phenotypic variance compared with WT plant under high-salinity condition.

Furthermore, we found that all three homeologs of *TaRSL4* (Traes_2AS_9A3F73BE3.1, Traes_2BS_659F9ABB9.1 and Traes_2DS_7C9C33E09.1) were responsive to salt stress and showed a higher expression level compared with the control at 6, 12 and 24 HASS. However, there were quantitative differences, with the A-homeolog of *TaRSL4* showing the highest expression level among three homeologs following salt stress (Supplementary Fig. S5). In a previous study, we demonstrated that *TaRSL4* was transgressively expressed in tetraploid and hexaploid wheat varieties compared with their dilpoid progenitor and positively associated with root hair development^27^. Accordingly, it has been reported that hexaploid and tetraploid wheat exhibit better fitness than diploid wheat under salt stress conditions^28^. Interestingly, T_4_ generation of wheat transgenic lines with constitutive expression of the *TaRSL4* A-homeolog (Traes_2AS_A204857F71) exhibited enhanced tolerance to salinity, showing approximately 23% longer in root length compared to the control (Fig. 4). Taken together, the results of high-throughput RNA sequencing of roots from salt-tolerant and salt-sensitive wheat cultivar provided us with a list of high-confidence salt stress related genes and shed light on the mechanisms underlying salt tolerance in wheat.

## Discussion

Being sessile, the ability of plants to sense and properly respond to various environmental stresses is central to their survival. Given that environmental conditions are highly variable, sensing and response mechanisms should change rapidly and require constant innovation to cope with stresses. Bread wheat is an agriculturally important allopolyploid species that shows broad adaptability to unpleasant environments compared with its progenitors, possibly due to an improvement of physiological processes, such as photosynthetic capacity, transpiration and metabolism^28^,^29^,^30^. Several genes have been reported to be responsible for salt tolerance in hexaploid wheat, e.g., *SRO* gene (Traes_5BL_79B792C51.2), encoding a poly (ADP ribose) polymerase (PARP) domain protein, improved the growth of wheat compared with the control when exposed to salinity stress by modulating redox homeostasis and maintaining genomic stability^15^; *TaAOC1* gene (Traes_6DL_BDDF27A65.2) encoding an allene oxide cyclase, enhanced the salinity tolerance of both wheat and *Arabidopsis via* regulating JA signaling pathway^16^, which was also significantly up-regulated after salt stress in both CS and QM. However, it has been challenging to identify the molecular mechanisms used by polyploids to accommodate themselves to the environmental constraints, although by comparing the salt tolerance of a synthetic allohexaploid wheat with its tetraploid and diploid parents, as well as a natural hexaploid bread wheat, Yang *et al*. (2014) found the expression of the *High-Affinity K *+* Transporter 1;5* (*HKT1;5*) homeolog on D-subgenome (Traes_4DL_3F8034BFD.1), a major salt tolerance gene, was immediately altered following allohexaploidization, suggesting that salt stress induces enhanced expressivity of the D-subgenome homeologs might contribute to the wide-ranging adaptability of natural hexaploid wheat^28^. In addition, it has been reported that functional partitioning of the homeologus genes of *alcohol dehydrogenase A* (*AdhA*) in allopolyploid cotton (*Gossypium hirsutum*) enables the plants to better cope with water submersion and cold conditions in the natural environment^21^, which sheds light on the possible underlying mechanisms.

As an allohexaploid, each gene present on the chromosomes of wheat should theoretically exhibit three homeologs (A, B and D), which are expected to share similar expression patterns. However, substantial, rapid, dynamic changes in the genome, chromatin structure and epigenetics occur due to genome shock during polyploidization events^31^,^32^,^33^,^34^, a phenomenon that polyploid plants showing genetic and epigenetic changes during the first few generations following wide hybridization, which unavoidably leads to gene expression partitioning among three homeologs, ranging from slight alteration to complete silence, and subsequently, neofunctionalization and subfunctionalization^34^,^35^,^36^. For example, approximately 55% of genes are reported to be expressed from only one or two homeologous loci in wheat roots and shoots due to genomic sequence loss or transcriptional silencing^37^. In addition, based on observations of the gene expression patterns from wheat chromosomes 7A, 7B and 7D, only 1,291 out of 2,386 (~54%) genes were expressed from all three homeologous loci, which further confirmed that substantial gene expression partitioning occurs among wheat homeologous genes^38^. Moreover, in a previous study on leaf transcriptomes, we found that ~68.4% of homeologs showed differential expression patterns under drought, heat and drought & heat conditions, which is a higher percentage than under normal conditions, indicating that abiotic stress might trigger gene sub- or neofunctionalization during wheat evolution and polyploidization events^22^. Accordingly, more than 60% of homeologous genes exhibited partitioned responses under high-salinity conditions, and the temporal or cultivar-specific expression features were confirmed through qRT-PCR. Interestingly, more triplets exhibited expression partitioning in the salt-tolerant cultivar (66.1%) than in salt-sensitive cultivar (63.9%) (Fig. 2), which is consistent with our hypothesis. Together, sub- and/or neofunctionalization of homeologous genes could partially contribute to the improved tolerance to salinity stress observed in polyploid plants, which is consistent with a previous report that allohexaploid wheat exhibits enhanced tolerance compared with allotetraploid and diploid wheat^28^. Furthermore, comparison of homeologous gene expression patterns between salt-sensitive and salt-tolerant cultivars suggests that expression partitioning of homeologs in polyploid wheat plays an important role not only in broadening adaptability to stressful environments *via* natural selection, but also in the improving performance of new cultivars *via* human selection.

Duplicate genes, which are derived from lineage-specific expansion during the course of evolution and polyploidization events, have been recognized as one potential sources of evolutionary novelty due to subfunctionalization and neofunctionalization^39^,^40^. For example, Leu-rich repeat genes have been shown to be important for disease resistance in plants, and these genes show a higher proportion of tandem duplications than other gene families^41^. Duplicate genes in *Arabidopsis*, poplar, rice and moss tend to be involved in the response to environmental stimuli based on analyses of Gene Ontology categories^42^. In addition, a meta-analysis of wheat leaf transcriptomes during the response to drought stress, heat stress and drought & heat stress revealed that a subset of stress responsive genes also exhibited a tandem distribution pattern on wheat chromosomes (Our unpublished data). Similarly, our results revealed that a number of salt-responsive genes were tandemly arrayed on hexaploid wheat chromosomes, forming hotspots containing consecutive salt-responsive genes. More interestingly, the expression patterns of duplicate gene members under salt stresses have fundamentally diverged for portions of tandemly arrayed families (Fig. 3), which likely provided a pool of highly dynamic targets for natural and human selection to meet the challenge of ever-changing environments. However, it is also worth noticing that some duplicate genes are likely to be pseudo-functionalized based on the expression analysis. Remarkably, the distribution of hotspots in the wheat sub-genomes was not even, as more salt-responsive clustering regions were located in the D genome. This finding is consistent with a previous report showing that diploid species with the D genome are relatively tolerant to high salinity, and a new synthesized hexaploid wheat variety (AABBDD) exhibited enhanced tolerance compared with its tetraploid parent (AABB)^28^,^43^,^44^,^45^. Therefore, plants have evolved complex interactions with environmental stress in terms of gene tandemness, because abiotic and biotic constraints impose intense selection pressure on plants and increased the probability of retaining tandem duplications, which, in turn, improve the tolerance of plants to unpleasant environments.

## Methods

### Plant Materials and Stress Treatments

Qing Mai 6 (QM) is a leading wheat variety in Shandong province, which is one of the most important wheat growing region in China. This elite wheat cultivar was released by Qi Lin (Qingdao Agricultural University) in 2007 and it developed a reputation for salt tolerant in Huang and Huai River Wheat Zone of China. ‘Chinese Spring’ (CS) is a well-known salt sensitive wheat variety which has been widely used in the study of salt response. Seeds of the wheat cultivar ‘QM’ and ‘CS’ were surface-sterilized in 1% sodium hypochlorite (NaClO) for 15 min, followed by washing in distilled water for several times, and then laid on moistened filter paper for 2 d at 20 °C. Seedlings of uniform size were grown hydroponically in half-strength Hoagland solution in a growth chamber with 22 °C/18 °C (day/night), 16 h/8 h (light/dark) and 50% humidity. Six independent biological replicates were employed, with three for sequencing and the other three for experimental verification. Salt stress was applied to QM and CS by the addition of 150 mM NaCl to the hydroponic solution after one week and seedlings in normal growth condition were taken as control. All experiments were performed in parallel. Roots were collected separately at 6 h, 12 h, 24 h and 48 h after stress treatment and frozen immediately in the liquid nitrogen, and stored at −80 °C for further use.

### RNA Isolation, Library Preparation and Transcriptome Sequencing

The total RNA from roots of QM and CS was extracted by using TRIzol reagent (Invitrogen, CA, USA) according to the manufacturer’s instructions and the concentration measurement was performed on NanoDrop 2000 spectrophotometer (ND-2000, Thermo Fisher Scientific, Inc., MA, USA). Next, RNA integrity was assessed using an Agilent 2100 Bioanalyzer (Agilent Technologies, Inc., CA, USA). Then, paired end (PE) sequencing library construction and sequencing were performed using TruSeq RNA Sample Preparation Kit v2 (Illumina, San Diego, USA) and HiSeq2500 platform respectively (Illumina, San Diego, USA). After that, Illumina pipeline (http://www.Illumina.com) was employed to process and filter raw data to generate FastQ files. Finally, approximately 109 G high quality 125-bp pair-end reads were generated from 16 libraries.

### RNA-Seq Reads Mapping and Expression Analysis

The high quality reads from each library were aligned to wheat reference genome released by IWGSC (http://plants.ensembl.org/Triticum_aestivum), by Bowtie2 with the parameters ‘−5 5 −3 5 –no-unal –phred33 –end-to-end -p 1 –reorder –score-min L,−0.6,−0.3 -L 15^23^’. Only uniquely mapped reads with no more than two mismatches were used for further study. Differential expression analysis was performed by using R software (ver. 3.0.1) and two-fold change and false discovery rate (FDR, Benjamini and Hochberg’s method) adjusted *P* value < 0.01 (fisher’s exact test) were taken as the criteria^24^.

### Homeologous Genes Expression Analysis

Homeologous genes expression analysis was performed using our previous methods^22^. Specifically, homeologous triplets were first identified by comparing genes of A-, B and D-subgenome from IWGSC using BLASTN (e-value cutoff 1e-10) with minimum 75% sequence coverage and 90% sequence similarity^25^. Secondly, all the aligned sequences were clustered and only clusters that had exactly one representative member from each subgenome and located on similar position of homeologous group were retained as triplets. Next, the high quality reads were mapped to identified triplets by Bowtie2 with the parameters ‘−5 5–3 5 –no-unal –no-hd -a –phred33 –end to- end –ignore-quals -L 15 –mp 6,6 –rfg 7,6 –rdg 7, 6 –score-min L,−0.6,−0.63 -reorder’ and only reads mapped to all three homeologs were then divided into 10 groups depending on the SNPs information between the triplets. Fourthly, gene expression level was calculated based on the 10 group of reads above, and if reads mapped to two or three homeologs, the reads abundance would be divided proportionally based on the counts of A, B and D specific reads. Finally, we conducted a Chi-square Goodness-of-fit test in R software to examine whether the expression level of triplet homeologs is biased or not, that is, if the transcript abundance of A-, B- and D homeologs showed significant difference (*P* value < 0.01) and the ratio of maximum expression value to the minimum was equal to or greater than 1.5 (Exp_max_/Exp_min_ ≥ 1.5), these three homeologs were defined as triplets with unequal contributions to their expression.

### Chromosomal Distribution Analysis of Salt Responsive Genes

Analysis of chromosome distribution of salt responsive genes was performed using a sliding window approach. We applied the algorithm of a window size of 10 genes per window and window shift of 5 genes per step to the linear genome zipper released by IWGSC^25^. The visualization of chromosomal distribution of salt responsive genes along wheat chromosomes was performed by R software.

### Heatmap and Principal Component Analysis (PCA)

Hierarchical clustering analysis of the expression data of genes was performed based on average linkage clustering with Cluster 3.0. Heatmaps demonstrating the gene expression data were created by the Java TreeView^46^. Principal component analysis was performed using ‘principal’ fuction in R software (ver. 3.0.1). And PCA plots among the biological replicates are generated by ‘scatterplot3d’ package in R software (ver. 3.0.1).

### Quantitative Real Time PCR (qRT-PCR) Validation

Two microgram total RNAs of each sample were used to synthesize the first-strand cDNA by using oligo (dT)_18_ primer with M-MLV reverse transcriptase (Promega, USA). QRT-PCR was performed in a 10 μL reaction volume containing SYBR Green master mix (TaKaRa, Japan) using CFX96 Real-Time PCR Detection System (Bio-Rad Laboratories, Inc., USA). The threshold cycles (Ct) were averaged for triplicate reactions and the values were normalized according to the Ct of internal reference gene (Ta-actin, 5′-GACCGTATGAGCAAGGAGAT-3′ and 5′-CAATCGCTGGACCTGACTC-3′). The primers used in qRT-PCR analysis were listed in Table S2.

### Plant transformation

*TaRSL4* gene cloning, vector construction and plant transformation have been performed in our previous study^27^. Briefly, pAHC25 plasmid with target gene were transformed into 2-week-old immature wheat embryos of Jimai5265 using PDS1000/He particle bombardment system (Bio-Rad) with a target distance of 90 mm the stopping plate and helium pressure of 1300 psi. The transgenic lines were screened according to the PCR analysis for the presence of the bar gene, followed by examination of *TaRSL4* expression level. At the T4 generation, three independent lines (#1, #3, and #5) with relative high expression level of *TaRSL4* were used for salt stress analysis.

### Salt Stress Treatments for Experimental Validation of Stress-related Genes

The Arabidopsis seeds of wild-type and mutants were first sterilized for 15 min in solution containing 12% NaClO and 0.1% Triton X-100, followed by washing with sterile water; Then, the sterilized seeds were incubated at 4 °C for 3 days in the dark; Subsequently, the seeds were sown on Murashige-Skoog (MS) medium (1% Sucrose and 0.6% agar, Sigma-Aldrich) for 5 days under 16-h-light/8-h-dark condition at 22 °C in a growth room, and seedlings with uniform size were chosen for salt stress treatment on MS medium with 100 mM NaCl. The wild-type and mutant seedlings were photographed and their root lengths were measured at 10 days after the transfer.

The sterilized wheat seeds were incubated at 4 °C for 3 days in the dark. Seeds with uniform germination were surrounded with filter paper soaked with 150 mM NaCl and grown in a greenhouse under 16 h /8 h and 24 °C/16 °C condition for light and dark circle, respectively. The measurement was performed at 3 days after the transfer.

## Additional Information

**Accession code**: Sequence data from this article can be found in the GenBank data libraries under accession number SRP062745.

**How to cite this article**: Zhang, Y. *et al*. Expression partitioning of homeologs and tandem duplications contribute to salt tolerance in wheat (*Triticum aestivum* L.). *Sci. Rep*. **6**, 21476; doi: 10.1038/srep21476 (2016).

## Supplementary Material

Supplementary Information

Supplementary Dataset 1

Supplementary Dataset 2

Supplementary Dataset 3

Supplementary Data
