# Supplementary material for: Expression partitioning of homeologs and tandem duplications contribute to salt tolerance in wheat (Triticum aestivum L.)
Source: Sci Rep. 2016 Feb 19;6:21476. doi: 10.1038/srep21476 (PMC4759826; doi:10.1038/srep21476)
Supplement: Supplementary Information [file srep21476-s1.doc]

**Expression partitioning of homeologs and tandem duplications contribute to salt tolerance in wheat (*Triticum aestivum* L.)**

Yumei Zhang^1,4^ , Zhenshan Liu^2,3,4^, Abul Awlad Khan^2,4^, Qi Lin^1, *^, Yao Han^2^, Ping Mu^1^, Yiguo Liu^1^, Hongsheng Zhang^1^, Lingyan Li^1^, Xianghao Meng^1^, Zhongfu Ni^2^, Mingming Xin^2, *^

^1^Qingdao Agricultural University, Qingdao, 266109

^2^ State Key Laboratory for Agrobiotechnology, Key Laboratory of Crop Heterosis Utilization (MOE), Beijing Key Laboratory of Crop Genetic Improvement, China Agricultural University, Beijing, 100193, China.

^3^Northwest A&F University, Yangling, 712100, China.

^4^These authors contributed equally to this work

^*^**Corresponding author**:

Qi Lin:

NO.700 Changcheng Road, Chengyang, Qingdao, Shandong, China, 266109. Email:

[nxxlinqi@163.com](mailto:nxxlinqi@163.com) Tel:0532-88030267, Fax: 053286080447

Mingming Xin

NO.2 Yuanmingyuan Xi Road, Haidian District, Beijing, China, 100193. Email: mingmingxin@cau.edu.cn, Tel: 010-62731452, Fax: 010-62731452.

**Supplementary Figure S1.** Statistics of RNA-Seq data and reads mapping.

**Supplementary Figure S2.** Heat map showing difference of enriched GO categories of salt responsive genes between QM and CS.

Functional enrichment analysis indicates that GO terms related to cell growth, potassium ion transport and response to abscisic acid stimulus were over-presented in QM up-regulated genes at 6 HASS and 12 HASS, while cell death and jasmonic acid biosynthetic process were specifically enriched in CS up-regulated genes at the corresponding stages. In addition, ion homeostasis and response to salt stress were differently enriched in QM and CS, respectively, at 24 HASS and 48 HASS. HASS: hours after salt stress. Purple: CS enriched GO terms; Blue: QM enriched GO terms; White: not enriched.

**Supplementary Figure S3.** Comparison of salt stress responsive homeologous genes in A-, B- and D-subgenome between CS and QM.

Venn diagrams show the number of up- or down-regulated homeologous genes in A-, B- and D-subgenome in CS and QM at 6, 12, 24 and 48 HAS.

**Supplementary Figure S4.** Distribution of tandem repeated salt stress responsive genes on 21 wheat chromosomes.

Red bar represents tandem repeat of salt stress responsive genes and the black triangles mark the hotspots shown in Figure 3.

**Supplementary Figure S5.** Partitioned expression patterns of *Ta-RSL4* homeologs based on the RNA-seq reads.

**Supplementary Table S1.** Number of up- and down-regulated homeologous genes specific in QM or CS at 6 HASS, 12 HASS, 24 HASS and 48 HASS.

**Supplementary Table S2**. Primers used for homeologous gene expression validation.

**Supplementary data sets 1**. Analysis of partitioned expression patterns of wheat homeologs based on Chi square test.

**Supplementary data sets 2.** Detailed expression partitioning of wheat triplets and their expression trends in response to salt stress.

**Supplementary data sets 3**. Details of salt responsive gene clusters on wheat chromosomes.

**Supplementary data sets 4.** Sequences of genes located in salt stress responsive hotspots.

Supplementary Fig. S1


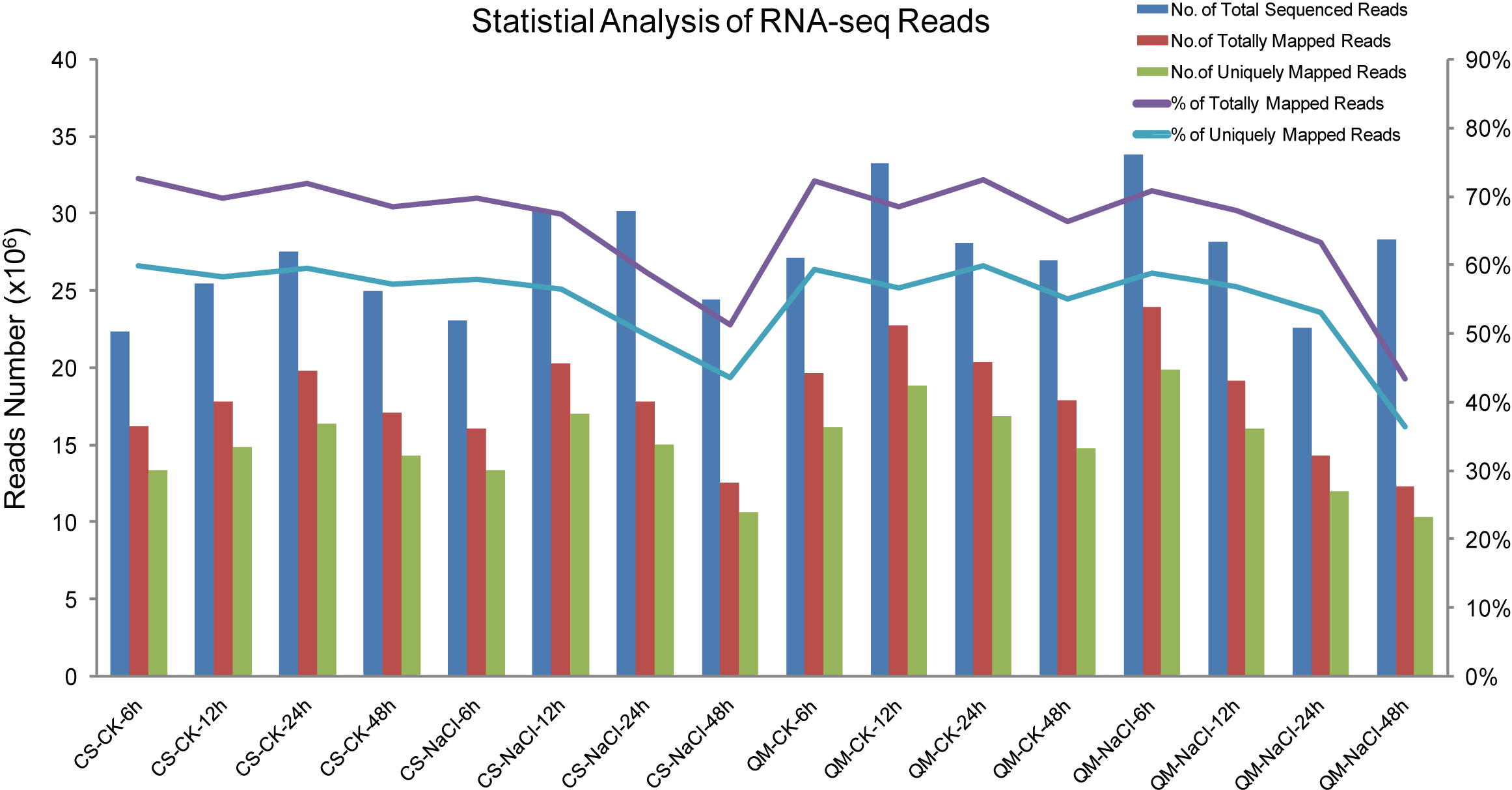


Supplementary Fig. S2


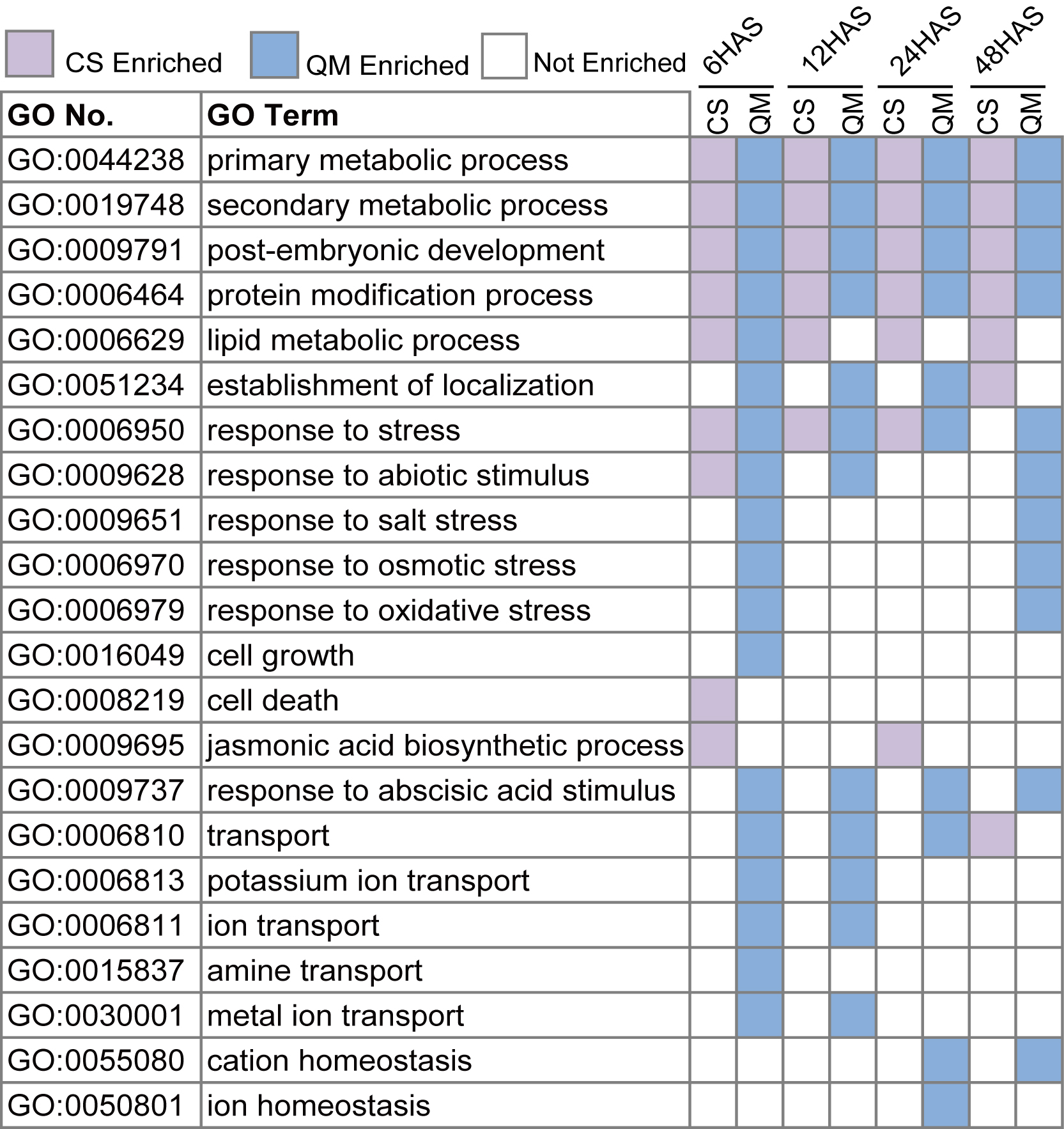


Supplementary Fig. S3


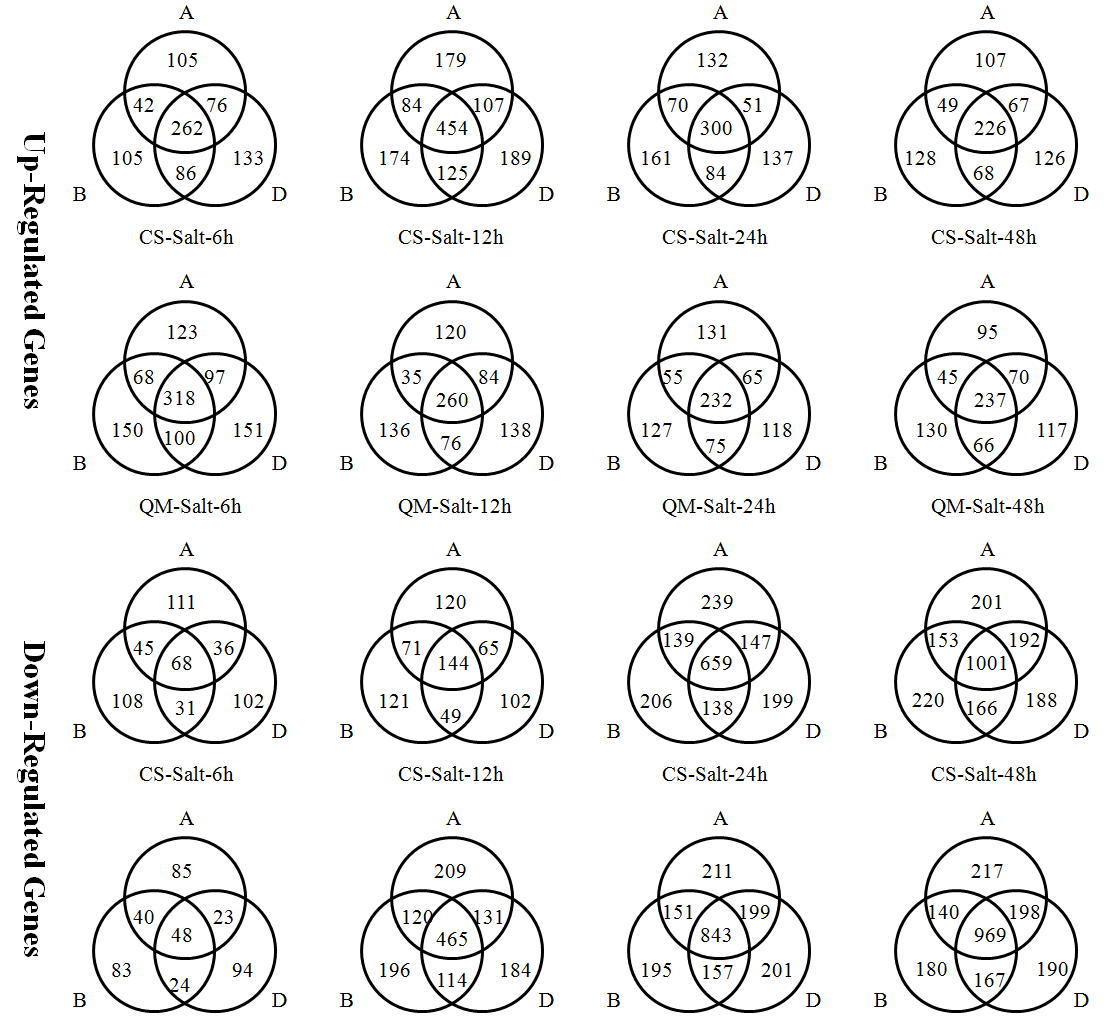


Supplementary Fig. S4


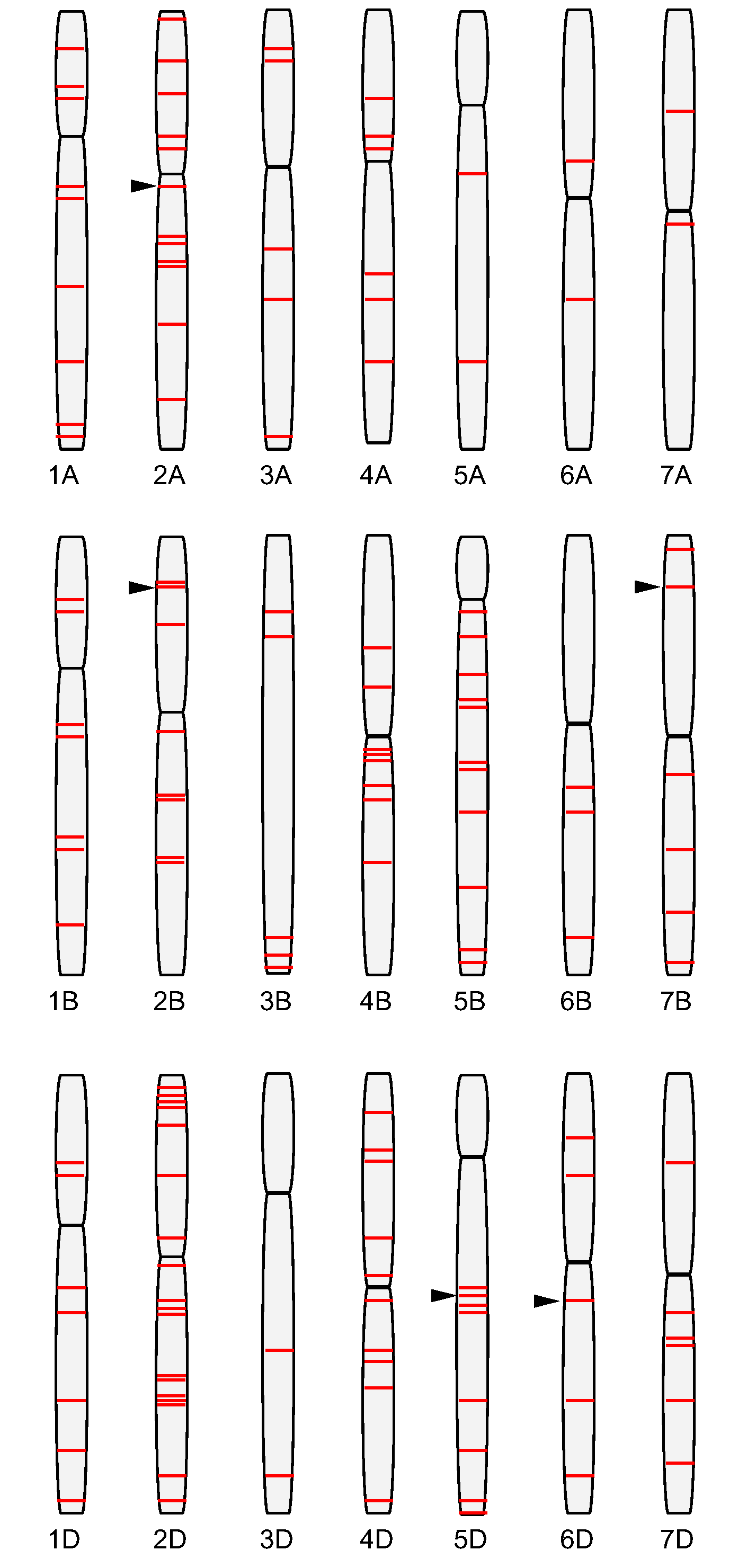


Supplementary Fig. S5


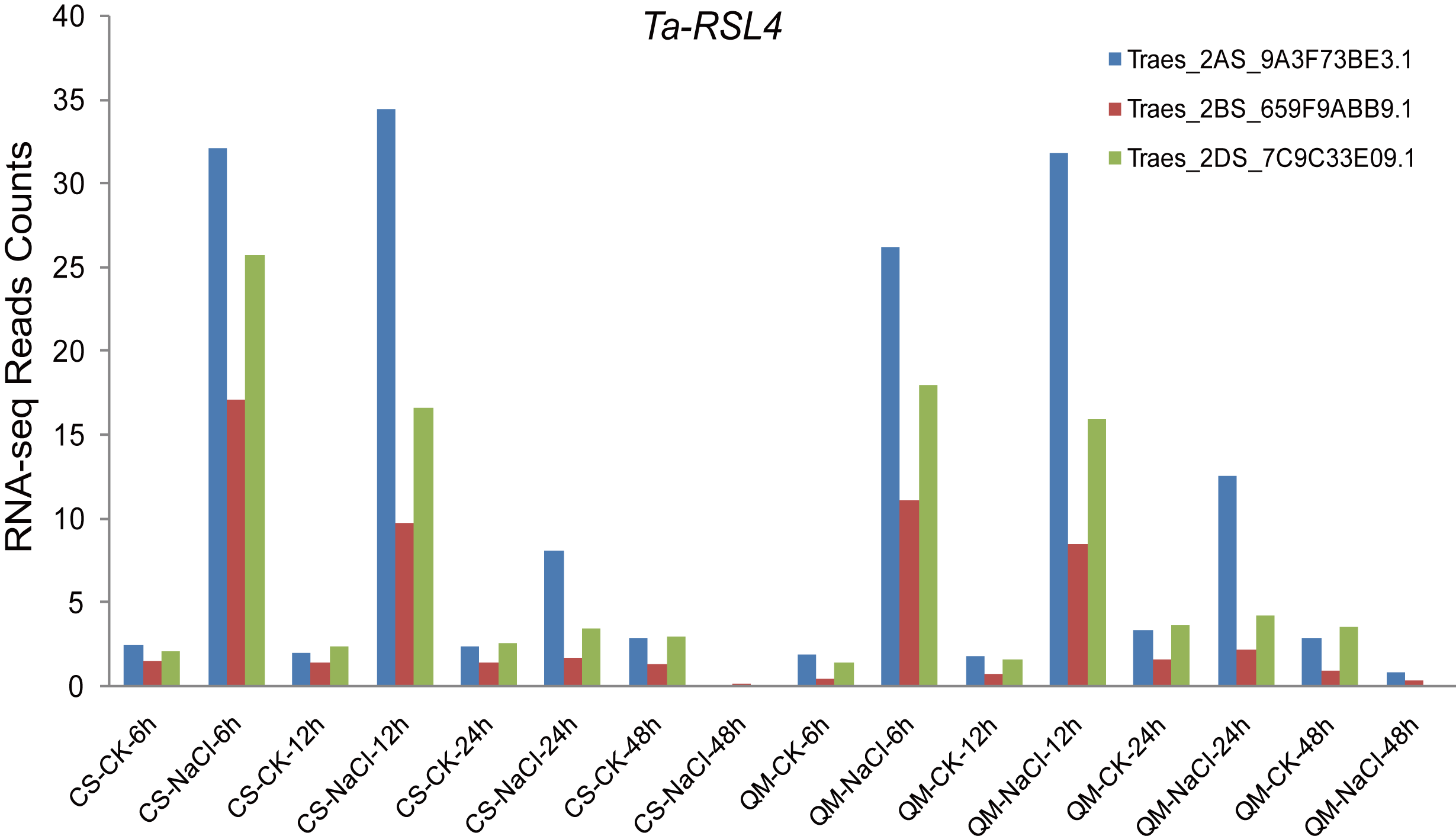


Supplementary Table S1

|  | **6HASS** | | |  | **12HASS** | | |  | **24HASS** | | |  | **48HASS** | | |  | **Total** |
| --- | --- | --- | --- | --- | --- | --- | --- | --- | --- | --- | --- | --- | --- | --- | --- | --- | --- |
|  | A | B | D |  | A | B | D |  | A | B | D |  | A | B | D |  |  |
| NDRH_CS_specific | 7 | 5 | 3 |  | 5 | 6 | 3 |  | 22 | 24 | 19 |  | 34 | 35 | 24 |  | 187 |
| NURH_CS_specific | 11 | 5 | 16 |  | 67 | 61 | 79 |  | 24 | 34 | 25 |  | 22 | 31 | 28 |  | 403 |
| NDRH_QM_specific | 7 | 12 | 8 |  | 45 | 52 | 40 |  | 43 | 44 | 56 |  | 41 | 44 | 47 |  | 439 |
| NURH_QM_specific | 15 | 19 | 24 |  | 14 | 17 | 21 |  | 10 | 11 | 7 |  | 9 | 15 | 18 |  | 180 |
|  |  |  |  |  |  |  |  |  |  |  |  |  |  |  |  |  |  |
| NDRH: NO.of_down_regulated_homeologs | | | | | |  |  |  |  |  |  |  |  |  |  |  |  |
| NURH: NO.of_up_regulated_homeologs | | | | |  |  |  |  |  |  |  |  |  |  |  |  |  |

| Supplementary Table S2 | |
| --- | --- |
| ***Primer name*** | ***Primer sequences (5'-3')*** |
| Triplet_70-A-L | GAAATTCAACATTCCAGAAGCTTTTTAG |
| Triplet_70-A-R | CTGCTAGCCTTGTTGAACGACC |
| Triplet_70-B-L | GAAATTCAACATTCCAGAAGCTTTTTAG |
| Triplet_70-B-R | TCCTACTAGCCTTGTTGAACGATG |
| Triplet_70-D-L | AAATTCAACATTTCAGAAGCTTTTTGC |
| Triplet_70-D-R | TCCTACTAGCCTTGTTGAACGATG |
| Triplet_1244-A-L | CCTTAAACCCCATGGTGAACAG |
| Triplet_1244-A-R | AGCCGTGTGATTTGCCTATATACTG |
| Triplet_1244-B-L | CCTTAAACCCCATGGTGAACAA |
| Triplet_1244-B-R | GGCTGTGTGATTTGCCTATATGCGT |
| Triplet_1244-D-L | TCTTAAACCCCATGGTGAGCAG |
| Triplet_1244-D-R | AGCTGTATGATTTGCCTATATGCGT |
| Triplet_722-A-L | GGCATGCCAATTTAGAGGCTAG |
| Triplet_722-A-R | CAAACAGGGAAAGCTGGTACTTGAGA |
| Triplet_722-B-L | GGCATGCCAATTTGGAGGCTGT |
| Triplet_722-B-R | CAAACAGAGAAAGCTGGTACTTAAGT |
| Triplet_722-D-L | GGCATGCCAATTTGGAGGCTGT |
| Triplet_722-D-R | CAAACAGGGAAAGCTGGTACTTGAGA |
| Triplet_272-A-L | GACATGGTTCACCCTGGAC |
| Triplet_272-A-R | CTTGAAGTGATTTGGTTGACTTGT |
| Triplet_272-B-L | GACATGGTTCACCCTGGGT |
| Triplet_272-B-R | CTTGAAGTGATTTGGATGACTTGT |
| Triplet_272-D-L | GACATGGTTCACCCTGGGT |
| Triplet_272-D-R | CTTGAAGTGATTTGGATGACTTAG |
| Triplet_2282-A-L | GACTTTCTCCAACTACGACGCAA |
| Triplet_2282-A-R | CAATCAATCCATTCTTCTCACATGA |
| Triplet_2282-B-L | GACTTTCTCCAACTACGACGCAA |
| Triplet_2282-B-R | CAATCAATCCATTCTTCTCACATTT |
| Triplet_2282-D-L | GACTTTCTCCAACTACGACGCGG |
| Triplet_2282-D-R | CAATCAATCCATTCTTCTCACATTT |
| Triplet_3766-A-L | CATTTCCTTCATTGCGATGCCCT |
| Triplet_3766-A-R | GGTTAAGCCCCTGACGTAGACC |
| Triplet_3766-B-L | CATTTCCTTCATTGCGATGCCCT |
| Triplet_3766-B-R | AGTTAAGCCCCTGATGTAGACG |
| Triplet_3766-D-L | CATTTCCTTCATTGCGATGCCAC |
| Triplet_3766-D-R | AGTTAAGCCCCTGATGTAAACG |
|  |  |
