# Supplementary material for: Expression partitioning of homeologs and tandem duplications contribute to salt tolerance in wheat (Triticum aestivum L.)
Source: Sci Rep. 2016 Feb 19;6:21476. doi: 10.1038/srep21476 (PMC4759826; doi:10.1038/srep21476)
Supplement: Supplementary Data [file srep21476-s5.doc]

>Traes_2DL_ECC8B1C6A

GCCGGGCACCGGCTCCTGCGCGACCACGGCATGGACTTCCACCCCGCCGACGCGTGCATG

TCCAAGGAGTTCATGGAGTACATCGCGAGGACGTGGCATGACGGGCAGGGAGCGGCGGTC

GGCGGCATGGTCATCAACACGTGTCGTGCGCTGGAGGGCGAGTTCATCGACGTGGAAGCC

CAGAGCCCGGAGTACAGCGGCCAGAAGATCTTCGCCATCGGGCCACTGAACCCACTCCTC

GACGCGAGCGCGAGGGCGCCGGGGGAGGCGCGGCACGGGTGCCTTGACTGGCTCGATAGA

CAACCGCCGG

>Traes_6DL_FC640A994

TCTTTCTGATCTATGCACAGCACATATATGACACTTTGCATAAACAATGTGATTTCTATG

ATCTACCTTGTTCACAGTATAATACCAAGTTACAGAATCTTGCTGCAATGCTAGATCGAT

TTCAGCAACTTATGCTGTTGATTGGTTTTTGCCTTCGTTATGGAACTTTGATGCAATGCT

AGGTTGTTTTCTGCAACTTACACTGTTGATTGCTTTTTGTCTTTGTTACAAATCGTGACC

CTATGTTTGCGACAGGTTCTCCGAAGGTGCCCAACTCATGCATGTTTGCTGGTTCGAGAT

CCGCGGGAAGATACATAGCAAGATGCTCTCCCAGGACACAACCTATGCCGCCTACA

>Traes_3B_24A29254B

ATGACCTTCGCCGGCAGCGGTGATGGCCAGAGCGGCTCTGCGAGGGCGCACTTCGTGCTG

GTACCCATGATGGCTCAAGGCCGTACCATCCCCATGACCGACATGGCATGCCTGCTGGCA

GAGCATGGCGCGCAGGTCAGCTTCATCACCACGCCGGTCAACGCCGCTAGGTTGGAAGGC

TTCGCCGCTAAGGTGGAGGCGGCGGGCCTGGTGGTTCAGCTCGTGGAGCTCCACTTCCCG

AGTGTAGAGTTCGGCCTACCAGATGGGTGCGAGAACCTCGACATGATCCAATCAAAGAAT

TTGTTCTTTAACTTCATGAAGGCATGTGCCGCGCTGCATGAGCCGCTCATGGCGTACCTC

CGTGAGCAGCAGCGCTCCCCTCCTAGCTGCATCATATCTGACATGGCGCACTGGTGGACC

GGTGACATCGCAAGGGAGCTCGGCATCCCGAGGCTCACCTTTAGTGGCTTTTGTGGCTT

>Traes_3B_3F5F50EF5

CGACGCCGTCGTCGTCGTCGACCGCGAGCAGGGCGGGAGGGAGAACCTCGCGGCGAACGG

GGTCACGCTGCACTCCCTCATGACGCTTACCGAGGTGCTCGCCGTGCTCGTCAGCCACGG

CAAGGTCACCCAGGAGAAGGTCGCCGAGGTGAAGCGCTTCCTCGACGCCAACAGGAAGGT

CTTGGTGCCGGCGGAGCCCAAGGCGGTGTCGGCAAGAGGGATGGCCTTCGCCGAGAGGGC

GAGGCTGGTCAAGAACCCGGTGGGGAGGAAGCTGCTCGAGGTGATGGAGGCCAAGCAGAG

CAACCTGCCGATGTTACAACAGCAAAGGAACTCCTTGAGCTAGCCAACAAGGTTGGTCCG

GAGATTTGCATGCTGAAAACCCATGTGGATGTCTTGTCTGATTTTACACCAGATTTTGGC

GGCAAGCTCCGTTCGATTGCAGAAGAGCACAACTTTTTGATCTTTGAAGACCGCAAGTTC

ACTGACATTGGGAACACGGTAACTATGCAATATGAAGGAGGAACATTCCGCATATCAGAC

TGGGCCGATATTGTCACTGCGCATGTAGTCCCTGGACCTGGAATTGTAGATGGCTTGAAG

CTGAAGGGTTTACCAAACGGAAAAGGGTTGCTCTTGGTTGCTGAGATGAGCTCACCTGGC

AACCTTGCTCATGGAGATTACACTGCAGCAGCTGTGAAGTTTGCCGAGCAACATTCCGAT

TTTGTGATTGAGTTTGTGTCGGTAAACCCTGCATCCTGGCCAGCTCCAGCGGCGAGTCCA

GCGTTCGTCCACATTATCGATCCGGGAGTTGATGTTGTTGCCGGAGGAGATTCTCCTGGT

CAGCAGTATGACACCCCTCATTCGATCGTAAACGAGAGAGGCGGCGATGTAATCGTGGTT

GGGCGCGGGATCATAGAGGCGAGCGATCCCGCCGAGGCCGCGAGGGAGTACCGCGTCCAA

GGGTGGCAGGCGTATCAAACCAGCTTGTAATAATGTACTACAAGTGGATTTCGGCCGCGT

TCAGGCAGGCGATCGGTGCAATAATAAAGATGGCTAGGAATATCTGAATAAGTGGATGTT

ACAGTCCCTACGGTCATGTAACAGAACAAAGCAACTCTACAGTAGTACTCCCTCGGTCTC

AAAATAAGTGTCAGTGATTTAGTACAGCTTTACAGGGAGTAAATAT

>Traes_3B_A7D7D698A

TCACGCGTGATGCATTTTCTCACTCATTATAATATGATTTTGAACCAACTGTTCACACTA

TGGAATAGTTCCCTTTTATTTGTAAAGGTGTCCACCGCTACTGACATATTTCTGTTGAAT

GCAGAGGCCTCCCATTGTTGGAATTCTCACAAGGCATGATTTTGTGGCGGAACATATCCA

CGGCCTGTTCCCTAGTCTCAATCCTCACAACTTTCATTCAGCCTCAATGGGAGGTTGATT

GTGATTTTTTTTGTTTCTTCTCATTTACAAAAATCATACAAATCT

>Traes_3B_E5B805A13

CAGGTTTGAGAGTTGAGTAGCACCATGCATATACGGCAAGTCAGCATCAGAGTTGAACAT

CATCCGGGCAGCCAAATTCAGTGCATCGAACGGCGTCCTCTTCTTTGACCATGAGGGTTA

ATCTGCGCCATCCGATGTCCCATCGGACTGCCTGGCCGCCGCCTTTCTGGAGGTTCAGAA

CTTGAGAAGATTCATTCATCCACATGGCCAACTCCACGAGTCCATGGCTGACGTTGGAGC

ATGCGTCACTAGCAAGAGGTGACCAAGTTGATACGCTGCACTCGTTCCAACGTCAGCTCC

TCAACTCAACTTAGGAAGATATAGCACCAGGACCAGGTGCTATATACACACTATGGTTGC

CATTGCACCGCTTCCAAGTCCTCCCAGCTTGACAAACTCCACTAGCACTTTTGCTGCCAC

AGCCGATCAGCCATGACCCTCTCCGGCAGCGGCGATAGCCAGAGCGGCTCCGCGAGGGCG

CACTTCGTGCTGGTACCGATGATGGCTCAGGGCCACACCATCCCCATGACCGACATGGCA

CGCCTGCTGGCAGAGCATGGCGCGCAGGTCAGCTTCATCACCACGCCGGTAAACGCCGCT

AGGTTGGAGGGCTTCGCCGCTGACGTGAAGGCGGCAGGCCTGGCTGTTCAGCTCGTGGAG

CTCCACTTCCCGGCTGCAGAGTTCGGCCTACCGGATGGATGCGAGAACCTCGACATGATC

CAATCAAAGAATTTGTTCCTGAACTTCATGGAGGCCTGTGCTGCGCTTCAGGAGCCGCTC

ATGGCGTACCTCCGTGAGCAGCAGCGCTCGCCTCCTAGCTGCATCATATCTGACATGATG

CACTGGTGGACCGGCGACATTGCAAGGGAGCTCGGCATCCCGAGGCTCACCTTTAGTGGC

TTTTGTGGCTTCTCGTCCCTTGTCAGGTACATCATTTTTCACAACAATGTATTGGAGCAT

ATCACAGATGACAATGAGCTCATCACGATCCCAGGGTTTCCTACACCGCTAGAGATGATG

AAGGCTAAATTACCTGGAACCCTTTCTGTTCCGGGTATGGAGCAAATTCGTGAGAAGATG

TTTGAAGAGGAGCTGAGATGCGATGGTGAGATCACTAATAGCTTCAAAGAGCTCGAGACA

TTTTACATTGAATCCTTTGAGCAGATAACAAGGAAGAAGGTCTGGACGGTCGGGCCAATG

TGCCTGTGCCACCGAAACAGGAACACAATGGCCGCTAGGGGTAACAAGGCGGCAATGGAC

GATGCACAGTGCTTGCAATGGCTTGATTCAAGGAAGCCAGGCTCAGTGATCTTTGTAAGC

TTTGGCAGCCTCGCTTGCACTACACCTCAACAACTTGTTGAGCTGGGACTGGGACTCGAA

GCCTCCAAGAAACCGTTTATTTGGGTGATCAAAGCAGGACCTAAGTTTCCAGAAGTTGAG

GAATGGCTCGCAGATGGGTTCGAGGAGCGTGTCAAAGATAGAGGTATGATCATAAGGGGC

TGGGCGCCACAGGTGATGATCCTGTGGCACCAAGCCATTGGAGGATTTGTGACGCACTGT

GGGTGGAACTCAATAATAGAGGGCATCTGTGCAGGTGTGCCCATGATCACTTGGCCGCAC

TTTGCAGAGCAGTTTTTGAATGAGAAGCTGGTGGTGGATGTGCTGAAAATCGGGGTGGAG

GTTGGCGTGAAAGGAGTTACACAGTGGGGAAGTGAAAAACAAGAGGTTATGGTTACACGA

GATGCCGTGGAGACGGCAGTGAACACCCTGATGGACGAGGGGGAGGCTGCAGAGGAGCTG

AGGGTGAGAGCAAAAGACTGCGCCATTAAGGCGAGGAGGGCTTTTGATAAGGAAGGCTCT

TCCTATAACAACGTAAGGCTGTTAATTCAAGAAATGGGAAACAAGACGAATGCATGTGGT

TGATACAGACGGTAATAAGCTCGCCTTTTCTGTGTAAACATGAAGTGAAAGATCCTAGAA

GACATATATCATCATATAGAATTTTGTACCAGGCTCACAGGTGATTATTTGGAGATTAGA

GATGATCCAAATGAAGGCAATATCAATTGTCTTCTTTTGTACGCTCTTTTATGGCAATCC

ACCACATAACGACTACACAGTCTGCTCTTCTAGATGGTTACGCTCTCACTTGCATTTTGC

TACCGCTGTTGCACAACTAGCCATGGTTGTCAAAGCATAGGACAATGCCGTGACGCCAAG

CAGCTCAAGGAAGAAGTGTGCCCTGGGAGCAACTGGGGAGGGGGCGTCGCCGTGCCCTCG

ACAGTGTTCTGTTGCATTGCACCAGACATTGTAAATGGCTTAATAACAGACCTTGTTTCA

TTGCTAATTGCCACGGTCTTGTATTAAATTCCTTTTAGATATAGATAATTTTGGGATAGA

TATAGATTATATTTGGTCTGTGTGCATGAAACAAAAGGAAAAGAAGGAGGCTCGGTTGTA

CCTCTTTGCGTCAGGAAGTCACTGTGTTGATTAGGATGCATTGCGTCGCTCCAAAAATGG

AGATATCACTGAAAGTTCTTTTCAGCTTCTCAAACAAAACTCTGAAAGGGAACAGACATT

GATCTTTGACTCGAGTGAGACGGTCGGAGAACGGACAAG

>Traes_3B_BBAE268C8

GCCGCGGCACCGGCGGGCGAGAGGCTAGTCCTGGTCGGGCACAGCCTCGGGGGGCTCAAC

ATCGCGCTCGCCATGGAGAGGTTCCCGCGCAAGGTCGCCGCGGCCGTGTTCCTGGCCGCG

TGCATGCCGTGCGTCGGCAGGCACATGGGCGCCACCACGGAGGAGATCATGAGACGGATC

AAGCCGGATTTCTTCATGGACATGAAGAGGATGGTTCTGAACACGAGCCAGGGCCCTCGA

CCTGCACTCGTGTTTGGTCCAAAAATATTGGCAGCAAAGCTGTACGATCGAAGCTCAGGT

GAGGATCAGACGCTGGCTACGATGCTGGTGAGACCGGGCTGCCAGTTCTTGGATGACCCT

ACCATGAAGGACGAGGCTCTGCTCACCGAAGCCAAGTACGGGTCGGTGAAGAAGGTGTAC

GTGGTGGCCATGGCCGACGCATCAAACTCCGAGGAGATGCAGCGTTGGATGGTCGACATG

AGCCCCGGCACGGAGGCCGAGGAGATCGCCGGAGCGGATCACATGGCCATGTGCTCCAAA

CCCAGGGAGCTCTGTGATGTCCTGCTCCGGATAGCCGACAAGTATGAA

>Traes_3B_879F7800C

AACATCATCTGGAAGGACTCATGGACTGCAAGATGCTGCCAATCAATAACAACCAGGTTG

CAGGGGTTGCAATGATAATGGGCCCAGACTTCTTAGCACACAAGAACTACCAGCAAAGTT

CACCTGAGGATTTGGCCCTGGCAAAAATGTTGGTGAGGCCGGGAAACCTGTTCATGGAGG

ATCCGGTGATGAAGAATGCAAGCCTACTCACCGATGACAACTACGGGTCGGTCAAGAAGG

TATACGTGGTAGCCAAGGCTGATGGAT

>Traes_3B_ADA43F567

TTTCCATTACCTTCCAGACCCCCGCCTCGGGCGACCTACATAAGCTCCCTCCTCCTCGCA

TCGCATCACCACCGAAAAATTCCACAAAATTCAACAAACAATCCAAGGCAAGCCCAACCC

GAGGAGATCGACCGAGCTCGAGATCAGCGAGCCAGAATGGCGGGCATGGTGTTCGGCTTG

GATGCCCCGATGATGACCGCGCTGCAGCACCTGCTGGACATCCCGGACGGCGAGGCCGGC

GGGCACGGCAACGCCGGCGGCGAGAAGCAGGGCCCGACGCGCGCCTACGTCCGCGACGCG

CGCGCCATGGCGGCCACCCCGGCCGACGTGAAGGAGCTGCCGGGCGCGTACGCGTTCGTG

GTGGACATGCCGGGGCTCGGGTCCGGCGACATCCAAGTGCAGGTGGAGGATGAGCGGGTG

CTGGTGATCAGCGGCGAGCGCCGGAGGGAGGAGAAGGAGGACGCCAAGTACCTGCGGATG

GAGCGCCGCATGGGCAAGCTGATGCGCAAGTTCGTGCTCCCCGAGAACGCCGACATGGAG

AAGATCTCCGCCGTGTGCCGCGACGGCGTGCTCACCGTCTCCGTCGAGAAGCTGCCGCCG

CCGGAGCCCAAGAAGCCCAAGACCATCCAGGTCCAGGTCGCCTGAGATGCATCGTGTGCG

CGTCGAATCGAAGCAGAGTGTGAGTGAGTAGCGAGTTTCCTGCGATGAGCGATGGTCTGT

CTGTCTCTTTGGTTTCGTCTGCTAGAGGGTGTGTGCCGTGTCACTGGTGATGGTTCGATG

TTTCGTTAATGGAAATGGGGATGTTCTCCGGCTTCGCCTGAATAAAACATGCGAATCTCT

TGGCCACATTTTCTATTGAAAAAACAAGATGTTCGTTTAAGTTCCAAGAGGGCCGTGTTC

TGAGAATTCAGGAAACTGCGTTTGTAACTTAACGGAAAATTGAATAAGACTAGAACATGA

GGAATAGCAAAATTAGGTGGAAAACGAACCTAGTTCTGAAGTGCTTTTTTCACCAGGGAG

AAAATCCTGGATGTCTTCCATTTTCATCTTCGAGAAATGTCTGGCTTTTGGGAAGATAGA

AAAAGGAATTTCCAGTTAGAATTGAGAATCCAGAAATGTTATTCTGCTCTAATTTGCTTG

AATGAAATATGTGA

>Traes_3B_EAF319CBD

GTAGCAATGGCCAGGGCTTTCCCCATGGGAGTGGTGGCCGCCGCGGCCGTGCTGGTGGTG

CTGTGTGCCGTCATTTCTTCTGCCGCCGCGCAGCCTCGTCCCCCTCTGCCAAAGAACTCC

CACATGATCACCCCGGGGCGGTTCGGGAAGAGGGCCCAGGTGCTCTCCTGCGACGACACC

AAGGACGGGAACAGCCCCTGCAGCGCCACCTGCGACAAGCGCTGCCCCAACGAGTGCGTC

GTCATGTGCCCAGGCTGCAAGACATACTGCTTGTGTGACTTTTACCCCGGGGTGTCATGT

GGCGACCCGCGTTTCACAGGAGCCGACGGCAACAACTTCTACTTCCACGGCAAGAAGGAC

CAGGACTTCTGTGTCATCTCCGATGCTGACCTCCATATCAACGCTCACTTCATCGGCAAG

CGCAACCCAGCTATGAGCCGTGACTTCACCTGGATCCAAGCGCTGGGCATCCGCTTCGCT

GATCACCGCATGTACATGGGCGCCCAGAAGACCACCAAGTGGAAGAACGACGTCGACCGC

CTTGAGTTGAACCTCGATGGAGAATCATTCAACATCGCTACAGACATCGGGGCAAAGTGG

CAGTCCACTGTTGTGCCTGGCCTGACCGTCACGAGGACCACCGTGACCAATGGCGTGAGG

GTCCAGCTTAAGGGTGTGTTTGACATCATGGCTAAGGTGGTGCCCATCACGCAGGAGGAC

TCCCGCGTCCACAACTACGGTGTAACTGACGATGACAGCCTCGCGCATCTGGACATCGGG

TTCAAGTTCTATGACCTCACCGACGACGTTCATGGTGTCCTTGGCCAGACCTACCGCCCT

GACTACGTCAACAAGCTCAGCGTGAGCGCTAGCATGCCTGTGATGGGTGGTGTAGCCAGC

TACATCTCCTCTGACATTTTCTCCACCGACTGCAAGGTCGCTAGGTTCGGACTTAGCACC

GGCATCTCCATGGTCACCACCACCAAATCA

>Traes_3B_B0185A209

CAAAGTCTCACCCTTCCATCACCCATAAGAATCCCTCTACTCTTCCACCCATGTACACAC

ATCCCACCCACACACATCATCCATCCACCCGTAGAAACATCGAGCATACACTTGGCGACA

TCCAGCTAGCTAGCTCTCGTCGGCGTCGACGATGGCGGGGAGGCGGCAGGCAGCCTGGTG

CCTGGTGGTCATCGCTCTGGCAGTGACGCTGGCGGTGGCGTCGGCGCAGCCCGTGCAGCC

GAGGGTGGGCGGGCCGGGGAAGAAAGGCGGCAAGATGCCGCCGGGCAAGTTCGAGACGGT

GACCTTCGCCAAGAACAACAAGCGCAAGTACGAGGTGGCCTGCACCGACAACCGCGGCCC

GCCCTGCGTCGTCTCCTGCCCCAAGACCTGCCCCAACAAGTGCCTCGCCTTCTGCGAGTA

CTGCATGACCTTCTGCATGTGCGACATGTTCCCGGGCACGTCGTGCGGGGACCCGCGCTT

CACGGGCGGCGACGGCAACACCTTCTACTTCCACGGAAAGAAGGACCAGGACTTTTGCAT

CGTCTCTGACAAGGACCTCCACATCAACGCGCACTTCATTGGCAACCACAACCCTGATAT

GAAGCGTGACTTCACGTGGGTGCAGGCCCTCGGTGTCACCTTCGTCCATGGCGGCGCCGA

CCACCGCCTCTACGTGGGTGCCAAGAAGGTCGTCGAGTGGGACGAGGAGGAGGACCATGT

TCAAATCACCCTCGATGGGGTGCCCGTGGAGGTGGAGGCCGGCAAGAATGCCCAATGGGT

CTCTAGGGCCATGCCGGGGCTCTCCGTCACTCGCACCGACACGGTGAACACCGTCGTCGT

GGAGCTCGACGGCGTGTTCAGCATCTCGGCCAACGCCGTGCCCATCACTGACGAGGACTC

CCGGATCCACAACTACGGGAAGACCAAGGACAGCCTCATGCACCTCGACCTAGGGTTCAA

GTTCCACACCCTTACTAACGGCGTCGACGGCGTGCTCGGCCAGACCTACCGCTTAGACTA

CATCAGCAAGGTCAACGTCACGGCCAAGATGCCCATCATGGGCGGCGCGCCCAAGTACCT

CTCGGCCAGCCTCTTCTCCACAGACTGCGCCGTCTCCAGCTTCCACCGTAACGGTGACGC

CGCCATCGAGACGTTCGCCTCATAAACCATTGGTTCATGGATCCTACACGAGAAATAAAC

AAATATCAACCGGAAGGGAGTGATATCATCTCCATCATGTTGTTGTGGAGTATATATGTG

TGCATCTCACACGATTGAGGTTGTGAAATAAGTTTCAAGTCAATAATTGAAGCAAAGTGT

GTACCAT

>Traes_3B_8FA11C089

CACAACTGCACGTCACTACCAAACAGAGAATATCTCAACCTAGCTAGAAACTTCGTCACT

TCAAGTAGGGAAACAGAGAATATCTCAACCTTGCTAGCCCTGTGCGCCTTCATCGCCGTC

GCCGATGCTCAGGCGAAGGCGCCAGTGGACCCCGCGCTGCCAAAGAACTCCCACATGATC

CACCCGGGCCGGTTCGGTAAGAGGGACCATGTGATCTCCTGCGATGACACCAAGGACGGG

AAGAACCCCTGCGTCGCCACCTGCGACAAGCGCTGCCCCAACGAGTGCATCGTTATGTGC

CCAGGTTGCAAGACCTACTGCTTGTGCGACTTCTACCCCGGCATGTCGTGCGGGGACCCA

CGCTTCACTGGTGCCGACGGCAACAACTTCTACTTCCACGGCAAGAAGGATCAGGACTTC

TGTGTCCTCTCCGATGCCGACCTCCACATCAACGCTCATTTCATCGGCAAGCGCAACCCC

ACCATGAGCCGTGACTTCACCTGGATCCAGGCTCTAGGCATCCGCTTCGCCGACCATCAG

CTGTATATGGGTGCCCAGAAGACCATTGAGTGGAACAACGACATTGACCGCCTAGAGATG

GCCTTCGACGGAGCATCTATTGAAATCCCTGCCAACCTTGGTGCCAAATGGGAGTCTGAC

ATCATTCCAGGGTTGACCGTCACTAGGACTGCTGTCACCAACGGTGTGAGGGTGCAGCTC

CAGGGAGTGTTCGATATCATGGCTAAAGTTCTGCCCATCACTGAGGAGGACTCCCGCATC

CATAATTATGGTGTGACCAAGGATGACAGCCTTGCACATTTGGACATCGGGTTCAAGTTC

AACAACCTTACTGACGATGTCCACGGCGTGCTAGGGCAGACCTATCGCTCCGATTACGTC

AACAAGCTTAGCATGACTGCTAACATGCCGGTCATGGGTGGTGCTACTAGGTATGTCTCC

TCTGGCATCTTTGCCACTGACTGCGAAGTGGCAAGGTTTGGCCACCATGTTGGCATCTCC

ATGGTTACTGCTCAGGCTAACTAAAATGTGGCTTCAATGTGGCCACCATCCGTGTGTGGG

AGTCATAAATAAATTTGTTTTCACGGACAGAAGTAGTTGATAATTGTAAGACTATTAATT

GTTGTTACTGTCCAAGATATCACTGAG

>Traes_3B_0B45CE6A2

GCCGCCGCCATGGACGCCGCCGCGCTGGAGGCCCTCATCCTCGACCTGCACGCCATCGAG

GCGGTCAAGCTCGGCTCCTTCGTGCTGAAATCCGGCATCACCTCCCCGATCTACCTCGAC

CTGCGCGCGCTCGTCTCGCACCCGCGCCTGCTCGCCGCCATCGCCGCCCTCCTCTCCTCC

CTCCCGGCCACCCGCCCCTACGCCATCCTCTGCGGGGTGCCCTACACGGCGCTCCCCATC

GCCTCCGTGCTCTCCGTCGACCGCGGCCTCCCCATGCTCATGCGCCGCAAGGAGGTCAAG

ACCCACGGCACCGCCAAGGCCATCGAGGGCGCCTTCCGCGCGGGGGACACGGTCCTCATC

ATCGAGGACCTCGTCACCAGCGGCGCCTCCGTGCTCGAGACCGCCGCGCCGCTCCGCGCC

GAGGGGCTCGTCGTCGCCGACGCGTTCGTCGTCGTTGACCGCGAGCAGGGCGGCAGGGAG

AACCTCGCGGCGAACGGGATCACGCTGCACTCGCTCATGACCCTCACCGAGGTGCTCGCC

GTGCTGCTGAAGCACGGGAAGGTCACCGAGGAGAAGGCCGCCGAGGTGAAGCAGTTTTTA

GATGCCAACAGGAAGGTCACCGTGCCAGGCGCCGCAAAGCCCAAGGTGGTCAGGAAGGGG

TTCCCTGAGAGGGCGGCCTTGGCCAAGAACCCCATGGGGAAGAAGCTGTTCGAGGTGATG

GAGGCCAAGCAGAGCAACCTCTGCGTCTCTGCGGATGTTGGCACAGCCAAGGAGCTCCTC

GAGCTTGCCGAAAAGGTTGGCTCTGAGATTTGCATGCTGAAAACCCATGTCGATATCTTG

TCAGATTTTACACCTGACTTTGGCGCCAAGCTTCGCTCGATTGCTGAGAAGCACAACTTT

TTGATCTTCGAAGACCGCAAGTTTGCTGACATTGGCAACACAGTAACTATGCAATATGAA

GGAGGAATATTCCGCATATTGGAATGGGCTGATATTGTTAACGCCCATGTAATTCCTGGG

CCTGGAATTGTTGATGGCTTGAAGCTTAAGGGTTTACCGAAAGGAAGAGGCCTACTTTTA

CTCGCTGAAATGAGCTCAGCTGGTAACCTTGCTCAAGGAGATTACACTGCAGCAGCTGTA

AAGATTGCCGAGCAACATTCTGATTTTGTGATTGGATTTATATCAGTAAATCCCGCATCG

TGGTCTGTGGCACCATCAAGCCCAGCGTTCATCCATGCCACCCCTGGAGTGCAGATGGTT

TCTGGAGGAGATGCTCTTGGGCAACAGTACACCACTCCTTATTCTGTGATAAACGACAGA

GGCAGCGATATAATCATTGTGGGGCGCGGGATCATCAAGGCGAGCGACCCCGAGCAGACG

GCCAGGGAGTACCGCGTCCAAGGGTGGCAGGCGTACCTGTCCAGCTTGTGAGGAAAGCTG

CTGCTCAGACGCCTCTCTCGGTGTAAAACCCAAATGAAATGCGCTGGTGTTCGTGCCATC

GACCGACCGATCAATGTAATAAGAGCGCCTCCTGTCGGCGCCGAGTGATTTCCCCCCTTG

TGCCTGGTGTCGGCGGCACGCGTCGTCTTGGGGGAAATAACTTCGAAATCGCGGTAATAG

AAACCAGAACGTTCCCATGGACACTGCCTGCTTGCTTGCTTGCTCCTTGTTG

>Traes_3B_1FABD0A3F

GTACTACACCCATCTCTAACTCCGGTGTATCAAGGCGCGCCGGCGATCCATGGCGTGGCA

GCTTATAGCTCTTCTCCCGGCTGCTTTGGTGGCCGCTGCCGCCGTCGCCGCCGCAGGGGC

GGGGCTGCCGTCCAACTTCGCCATAATAACCCCACGGGGACCCTTAATGGGGAAGCGGGA

TGCGGGGGAGTACTGCGGCAAGAGGAAGTGCATGGCCAAGTGCGAGAGCCGCTGCCCCGA

CCAGTGCTTCGTCCTCTGCCCCAGCTGCAAGACGCTATGCATGTGCGACTACTACCCGGG

CATCTCCTGCGGCGACCCGCGATTCACCGGGGGCGACGGCAACAACTTCTACTTCCATGG

CAAGAAGGACCAAGACTTCTGCGTCCTCTCCGATGCTGATCTCCATGTCAATGCCCATTT

CATTGGCACCCACAACCCCGCCACGGGTCGTAACTTCACCTGGATCCAGGCCATCGGCAT

CCGCTTCGCAGACCACCGCCTCCTCGTCGGTGCCAAGAGGACCGTGAAATGGAACAACCG

CGTCGACCGTCTTGAGATGGGCTTGGACGACGAGACCATCGATCTCCCTGCCAGGCTCGA

CACGCGTTGGGAGTCGGCAGCCGTGGCAGGCCTGACCATCACGAGAACCGCCGTGACCAA

TGGCATCAGAGTGCAGCTCAAGGGGGTGTTTGACATCATGGCCAGCGTGGTGCCCGTCAC

GGAGAAGGACTCCCGCATCCACAACTACGGTGTCACGGAGGGCGACTGTCTTGCTCACTT

GGACATCGGTTTCAAGTTTCACGACCTCACCGACGACGTGCACGGTGTCCTTGGTCAGAC

ATACCGCTCCGACTATATTAACAAGCTTAGGGTGAGCGCCAGCATGCCGGTGATGGGCGG

CATAACCAGCTATGTATCATCGGATATCTTTGCCACAGATTGCGCAGTTGCTAGATTTGG

TCGCCGTACTAGCATCTCGATGGTTGCATCAAGTGATAGTTGAAGTACTTTTCCGGCTAG

ACGTTGCCTTAGCTAGCCAGCACGCAGTCAGACATGAATAATTGAGTTCATGCACAAAAG

TGTAATATAAATAAGGCACATGGATCTTTTTGGAGAAACCCCATATATATATGCATATAT

TTTATTGGCACATAGACCCTTAGTCTTAGGGCCTACTCTCTTCCTACCATG

>Traes_3B_284C1E200

CTTGGCCCCAAATTGTTGGCAGCGAAATTGTACGATCGAAGCTCAGTCGAGGACCTGACA

CTGGCTATGTTGTTGGTGAGACCGGGCTGCCAGTTCGTGGACGACCCGATGATGAGGGAC

GAGGCTCTGCTCATCGACGCCAACTACGGGTCGGTGAAGAAGGTGTACGTTGTGCTCACG

GACGACGTTTCCACCTCCGAGGAGATGCAGTGCTGGATGGTCGACCTGAGCCCGGGCACG

GAGGCCGAGGTGCTCGCCGGAGCCGACCACATGGCCATGTGCTCCAAGCCCAGGGAACTC

TGTGATGCTCTGCTCAGGATCGCCAACAGACTGGCAGACAAGCACGATGCTAAAATTGAG

GAGCTGGCTAAGAAAAGAGTTGCTGCTAAAGATAATTATGGT

>Traes_3B_4887B0D241

CAGGAAACCGTTTATTTGGGTGATCAAAGCAGGAGCTAAGCTTCCAGAAGTTGAGGAATG

GCTCGCAGACGGGTTCGAGGAGCGTGTAAAAAATAGAGGCATGGTCATAAGGGGTTGGGC

ACCACAGCTCATGATCCTGCGGCACCAAGCCGTTGGAGGATTCGTGACGCACTGTGGGTG

GAACTCAACAATAGAGGGCATCTGTGCAGGTGTGCCCATGATCACATGGCCACACTTTGG

GGAGCAGTTTTGGAATGAGAAGCTGGTGGTGGATGTGCTGAAAATCGGGGTGGAGGTTGG

CGTGAAAGGAGTTACACAGTGGGGAAGTGAAAAACAAGAGGTTATGGTTACACGAGATGC

CGTGGAGACGGCAGTGAACACCCTGATGGACGAGGGGGCGGCTGCAGAAGAGACGAGGGT

GAGAGCAAAAGACTGCGCCATTAAGGCAAGGAAGGCTTTTCATGAGGGAGGTTCTTCATA

TGACAACATAAGGCTATTAATTGAAGAAATGGGAAACAGGATGAATGCAGGTGGTTGACA

CAGATGGTAGTAAGCTCTCTTTTTCGGTGTAAACATAAAATAAAAGAGCCTATAGTACAA

ATAACATGTCATTAGAAAGAGCCTATATGCCATTGGAAATAATTTTATTTTATAAATAAC

CCGCTGCTTGTATCAGGCTCACTGGTGTGACCGTGATTGTTTAGAGATTAAACAGGGTCC

AAATGACAGCAATATCAATTGTCTACGCCATTGTGCAGATTTAATGACAGTCAAGATAAT

GATTACA

>Traes_3B_39E043D56

CGCTAGTGGTACATACACTGCACACTGCCAGAGCTTTGCTCGCTCCTCTGTCTCCTCCCT

CCCTCCTCCAGCTCCACCACACCGCGCTCCCTGCCTCGCCGGTCGTCGCCGGCGGCCATG

GACGCCGCCGCCATGGAGTCCCTCATCCTGGAGCTGCACGCGGTGGAGGCCTACAAGTTC

GGCGCCTTCGTGCTCAAGTCGGGGATCACCTCCCCGATCTACCTCGACCTGCGCGTGCTC

GTCTCCCACCCGCGCCTCCTCTCCCGGGTGGCGGCCCTCCTCGGCTCCCTCCCCTTCACG

CGCCCCTACGGCCTCCTCTGCGGCGTGCCCTACACGGCCCTCCCCTTCGCCTCCGTGCTC

TCCGTCGCCCGCAGCATCCCCATGATCCTCCGCCGCTACCACGAGACCGGCGCCGGCGCC

GCCCCCGCGATGCGCACCCAGGGCTCCTTCCGCGACGGGGACCTCGTCACCAGCGGCGCC

TCCGTGCTCGAGACCGTCGCGCCGCTCCGGGACGAGGG

>Traes_3B_8FB469D8A

AGCGACCGCATCGAACCTCTAGAAGTTTCTCCATCAGCGGTCGAAGGACTCCCCACTGCT

CCGCGCTCGTTCCACCACCTTTCAGAAGTTTCCATCACCTTCCAGACTCCCCGCCTCCGG

CGACCTACATAAACTCCCTCCTCCTCGCCATCACATCACCACCAAAGCATTCCACAAGAT

TCAACAAGCAATCCAACCCGACGAGATCGATCGAGCTCGAGATCAGCGAGCGAGAATGGC

GGGCATGGTGTTCGGCTTGGAGAACCCGATGATGACGGCGCTCCAGCACCTGCTGGACAT

CCCAGATGGCGAGGCCGGCGGAGCCGGTGCCGGCCGCGGCGAGAAGCAGGGCCCGACGCG

CGCCTACGTCCGGGATGCGCGCGCCATGGCGGCCACCCCGGCGGACGTGAAGGAGCTGCC

GGGCGCGTACGCGTTCGTGGTGGACATGCCGGGGCTGGGTTCCGGCGACATCAAGGTGCA

GGTGGAGGACGAGCGGGTGCTGGTGATCAGCGGCGAGCGGAGCAGGGAGGAGGAGGAGGA

CGCCAAGTACCTGCGGATGGAGCGCCGCATGGGCAAGCTGATGCGCAAGTTCGTGCTCCC

CGAGAACGCCGACATGGAGAAGATCTCCGCCGCGTGCCGCGACGGCGTGCTGACCGTCAC

CGTCGAGAAGCTGCCGCCGCCCGAGCCCAAGAAGCCCAAGACCATCCAGGTCCAGGTTGC

CTGAGCTGGTATGCATTGTGTGCGCGTCGAATCGAAGCAGAGTTTGAGTGGCTGAGTGGG

TACTGAGTTTCCTGTGATGATGAGTGATGGTTTCTCTGTCTTTTGATCTCGTCTGCTAGA

GAGAGTGTGTGTCGTATCGATTCGTGCGCACTGGTGATGGTTTGATGTTTCGTTAATGGA

AATGGGGATGATCTCCGGCTTCGCCTGAATAAAACATGCGATTCTCTTGGCCACA

>Traes_3B_9C0F52B94

CCGCACCCAACCCAGACCTAGCTACCACACTTGATCCTCCACACTGCGAGCTGAGTCAAG

CTAGCAATGGCGAGGGCATCGTCGTCGTCGGTGTTTTGCATGGGTGTGGTCGCCGCGGTG

GCGGTGCTGATGGTCCTGTGCGCTGCAGTGCCGGCCGCCGCTCAGGGGAAGACGGGGCCT

CCCGCTCCTAAGCTATCGTCCAACTTCAAGACGATCCATCCGGGGCGGTTAGGGAAGAGG

GCGCAGGTGCTCACCTGCGACGACGAGAAGGACAAGAAGAACCCCTGCGTCGCCACCTGC

GACAAGGTCCGCTGCCCCAACGAGTGCATCGTCATGTGCCCAGGCTGCAAGACCTACTGC

ATGTGCGACTTCTACCCCGGCGTGTCCTGCGGGGACCCGCGCTTCACCGGCGCCGACGGC

AACAACTTCTACTTCCACGGCAAGAAGGACCAGAGCTTCTGCGTCGTCTCCGATGCCGAT

CTCCACATCAACGCCCACTTCATCGGCAAGCGTAACCCCACCATGAGCAGAGACTTCACC

TGGATCCAGGCCCTCGGCATCCGTTTCGCCGACCACCGCCTTTACATGGCCGCCCAGAAG

ACCATCGAGTGGGACAGTGACGTCGACCGCCTTGAGCTGGCCTTTGACGGCATGCCCATC

GACATCCCCACCGAGATGGATGCAGAGTGGCAGTCCACCATCGTGCCCACCTTGACTGTC

ACGAGGACCTCCGCGGCCAACGGCGTCAGGGTCCAGCTCAAGGGGGTGTTTGACATCTTG

GCCAATGTGGTGCCCATCACCGAGAAGGACTCCCGCATCCACAACTACGGCGTCACGGAG

GAAGACAGCCTCGCACACTTCGACATTGGGTTCAAGTTCAACGCCCTCACTGACGACATC

CATGGCGTGCTCGGCCAGACATACCGCACAGACTACGTCAACAAGCTCAGCGTGAGCGCC

AACATGCCGATCATGGGTGGCGCTGCCAGCTACGTCTCCTCCGACATCTTCTCCACCGAC

TGCAAGGTTGCCAGGTTCGGCCGTGGCCACAACAAGATCTCCATGGTAACCACCAAGGCC

AATTAAACACTGCACATGGACGGCTAGGTCTCACGACATCTGAGACTTGGCCCAGGAATA

AGAAAAGCGGATTTATGAGATATTGATGCCACGAAAGACACACATGATCATGTCATTCGG

ATACGCACACAAACATGATTGTGCAATTGTAACTGCAAATGATGAAATGAAATAAATTTA

TTACCGAA

>Traes_3B_A7850AFD7

GCATCATGCATGCGCTCATTCAAGGAAATTTCTCTTGTTATGCATAAAACCAAACCTAGC

CTCAGAGTGAGAATTTGTTTTTGCGGGAGGCTTTGGCAAGTGTGTGACTTTTACCCTGGC

ATGGTGTGCGGTGATCCACGTTTCACCGGGGGTGACGGCAACAACTTCTACTTCCACGGA

AAGAAAGAGCAGAACTTCTGCACCATATCTGACAGTAACCTGCACATCAATGCTCATTTC

ATTGGCAAGCGCAACCCGACTATGAGCCGTGACTTCACCTGGATCCAAGCTCTCGGCATC

AGCTTCAGCAACCACCGCCTCTATATAGGTGCCCGGAAGACTGTTAAATGGGACAGCGAG

GTTGATCGCCTTGAGCTCGCCTTCGACAACATGCCCATTGACATCCCTGCCGAGATCGGC

AGGCAATGGCAGTCTGATGTTGTATCAGATCTAACCGTCATGAGGTCTGCCATGACCAAT

GGCGTGAGAGTGCAGCTCAAGGGTGTATTTAACATCATGGCCAACGTGGTGCCTATCACA

AAGGAAGACTCCCGCATCCACAACTATGGAGTGACTGACGATGACAGCCTTGTACATCTT

GACATCAGTTTCAAGTTTCATGACCTCACGGACGACGTCCATGGCGTGCTTGGCCAGACC

TATCGCACCGACTATGTCAACAGGTTCAATGTAAGCGCCAGCATGCCTGTCATGGGTGGC

ACCGCCAACTACTTATCTTCTGACATCTTCTCCGCAGACTGCAAAGTTGCTCGGTTCGGC

CGCCACGCAGGAATCTCCATGGTTACTGCCACGGTCAAT

>Traes_3B_FD5D37A6F

CGTCACTTTGTCCTCCCCTACGCCACTTCGCAAGTAAGCCACTTCGCCTCCAATTAAGCT

CCCTGGCTAAAGTTACCACGCCACTTCCATCTCCCATAAGAATCCCCCGGTCGGCGCCAC

ACAAACGCACACCACAACCATCGAAAGCCGGCTACAAACACACACTTGATCGAACATCGA

AGAAGGTAGCTCTTCACCAGCTCCATCGCCGTCGCAATGGCGCGGCGCGGCGGCATGGTG

GTGGTAGGCTGCTGCCTCCTGGTGGCACTCGCGCTGGCGTGCGGCGTGGCGTCGGCGCAA

CCCTCGGAGAATGGTCCGCCCACGAAGCTCCCGCCCAAGGCCACGTTCCAGACCATCACC

ATCCCCAAAAACAGCACCAAGAGGCTGTACGCGGTCACCTGCCGCGAGAGGCGCGGAAAG

CCTTGCGTCGTGTCCTGCCCAAGCCGCTGCCCCAACAAGTGCCTCGCCTACTGCAAATAC

TGCATGGCATTCTGCGTGTGTGATCTCGTCCCGGGGAGCTCGTGCGGAGACCCCCGCTTC

ACCGGCGGGGACGGCAACACCTTCTACTTCCACGGCAAGAAGGACCAGGATTTTTGCATC

CTCTCCGACGAGGCCCTCCACATCAACGCCCACTTCATCGGCAACCACAACCCCGCCATA

AGGCGCAACTTCACGTGGATCCAGGCCATCGGCGTCAGCTTCGGCCAGCACCGCCTCTAC

GTCGGCGCTCGCAAGGCCGCCATCTGGGACGAGGAGGAGGACCACATCCACATCATGTTG

GACGGCAAGGCCGTCGATGTGGAGACCGTCAAGAACACCCGGTGGGTCTCCAAGGCCTTG

CCCTCCTTGTCCGTCACGCGCATCGACACGGTGAATGCCGTCATGGTGGAGCTCGACGGC

GTGTTCGGCATCTCCGTCAACGCGGTGCCGATCACCGAGGAGGACTCCAGGATCCACAAC

TACGGCAAGACCGGGAGCGACAGCCTCGTGCACCTTGACCTCGGCTTCAAGTTCCATTCC

CTCACCAAGAGCGTCCACGGCGTGCTCGGCCAGACCTACCAGCCGGAGTATGTCAGCAAG

GTAGACATCAGCGCCAAGATGCCTATCATGGGCGGCGCACCGAAATACCTATCCTCCAGT

CTTTTCTCCACGGATTGCGCCGTCTCCAAGTTCCGCAGCAACAACGTCGCCGGGAGTGTC

ATCACCTTTGCCTCGTAAACCGTTCGCCCTTATGGACATGTAAGCACCTATTAATGCTAC

AAATGGAATAAGTCAGTAATTGGGGTATATATGTACGTACACAAAAAGAAATAAGTTCAG

AAAACATCGACCGGTGGTGTTTGAAATCTTTCAAACATTGTAGAAAAGGCTGTATTTCAA

TTGAATTGTG

>Traes_3B_94D4C853F

GACGTGAAGGAGCTGCCGGGCGCGTACGCGTTCGTGGTGGACATGCCGGGGCTGGGGTCC

GGCGACATCCAGGTGCAGGTGGAGGACGAGCGGGTGCTGGTCATCAGCGGTGAGCGCAGG

AGGGAGGAGAAGGAAGACGCCAAGTACCTGCGGATGGAGCGCCGCATGGGCAAGCTGATG

CGCAAGTTCGTGCTCCCCGACAACGCCGACATGGAGAAGGTCTCCGCCGTGTGCCGCGAC

GGCGTGCTCACCGTCACCGTCGAGAAGCTGCCGCCGCCCGAGCCCAAGAAGCCCAAGACA

ATCCAGGTCCAGGTCGCCTGAGATGCATCGTGTGCGCGTCGAATCGAATCAGAGTGTCAG

TGGGTAGTGTCTGTCCTCTGATTTCATCTGCTAGTACGGTTATCTGAGAGAGAGGGTGTG

TACTGTCTGTTCGTGCGTACTGATGGTTGTTTGATGCTTCGTTAATGGAAATGCAGATGT

TCTCATGCTTTGCCTGAATAAAACATTCGAATCTTGAAATTTTGAAGCGTGTTTTTAAGT

TAGAGGCCCGTGTTTTGGAAAAAAAATTAAACTGCATTTGTAACTAAATGAGAAACTGCA

GCCAGAATTGGCAAATTTAGGTGAAATTTTTCAGCAGCTACACAAATCATTGGTGCTTCG

ATGTCTGGATTTTGAGAATATTAAGATTTCCTCCTGCAATCTGACCATTATGCTAGGAAA

ACTCACCATCGACATTCGATTACACATACTCCCTCCGTTACACATACTCCCTCCGTCCTC

CGTCCGAAAATAAATGTCCCAACTTCAACAACTTGGTTTTCTTTTTGCTTGGCCAGGGAA

TGTGGTACCCCGAGCTTTGTTACAAAGTATCGCAGGACACAGCCATACCACAAGGCAGAA

AAATCAGTTCAAACTTCAAGCTGAGCAAAAAATTCACTACTGGGTACACAAAAAAAGATA

GTCCGCCATCAAAATTGTTGTGTCTCCGAGATGTCAGGCTGAACTAACGCCATACATCTC

CAGCGAAAATGAAGCGACCAACATGATGCAAGTTTTACATAGTATCTGCATCCGAATCAG

AATCGCCGTCATCCTGTGTGGACAGTGGGCTGTTGAGATTACTCGCGTAGAGATCCTTCC

CCGTTATCTTGTCCCGCACATTCATGGACATGTAGGGCGACTCGCAT

>Traes_3B_8EAF73720

CACAGGGCTGGCATAGCTAGTAGTTTAGCTATAGCAGCCACACACACACGCACACGTACA

CTTACTCGGCTACCTCCGTTTGCGGCTTTGTAGCGCAATGGCGAGGGCATTGACCATGGG

CGTAGTCGCCGCGGCGGCCGTGCTGGTGTTCCTGTGCGCCACCGCCGTGCCGGCCGCTGC

CCAGGGAAAGAAGGGGCTGCCCAAGAACTCCCACGTGATCCACCCGAAGCAGTTCGGGAG

GAGGGAGCAGGTGCTCTCCTGCGACGACACCGCCGACGGCAACACCCCCTGCGTCGCCAC

CTGCGACAAGCGCTGCCCTAACGAGTGCGTCGTCCTCTGCCCAGGCTGCAAGACCTACTG

CATCTGTGACTTCTACCCCGGCGTGTCATGCGGGGACCCACGCTTCACGGGCGCGGACGG

CAACAACTTCTACTTCCACGGCAAGAAGGATCAGGATTTCTGCGTCGTCTCTGACACCGA

CCTCCACATCAACGCCCACTTCATCGGCAAGCGCAACCCCACCATGAGCCGAGACTTCAC

CTGGATCCAAGCCCTCGGCATCCGCTTCGCCGACCATCGCCTCTACATGGGCGCCCAGAA

GACCGTCAAGTGGGACAGCAACGTCGACCACCTCGAGCTGGCCTTCGACGACATGCCCAT

CGAAATCCCTACCGCAATCGACGCCCAGTGGCAATCCACCACCGTGCCCGCATTGACCAT

CACAAGGAGCGCCACAACTAACGGCATCAGGGTCCAACTCAAGGGAGTGTTCGACATCTT

GGCCAATGTGGTGCCCATCACAGAGAAGGATTCCCGCATCCACAACTACGGCGTAACCGA

GGATGACAGCCTCGCGCACTTCGACATCGGCTTCAAGTTCCACAGCCTCACCAACAACGT

CCACGGTGTGCTTGGCCAAACCTACCGCACCGACTATGTCAACAAGCTTAGCGTGAGTGC

CAACATGCCAATTATGGGCGGCACCTCTAGCTACGTCACCTCCGACATCTTCTCCACCGA

CTGCAAGGTTACCCGTTTTGGCCGCTCTTCGGGAATCTCCATGGTCACTGGCATGGCCAC

TTAAGCATCATGCGTCGACGGCTAAGCTCTAAGCTAGTGTCTTAACTTGACATCCTAGAG

TTTGCCGAGGAATAATTAACACCTATCTGTGACAGGCTGGTACCACCATGTTACACACGA

TCATGTCATCTTTAATACAAGCACAAGCATATGGTACGATCGATTGATTTTCAAATGAAA

TAAAGGAGTTTACCCAAGACTATGGT

>Traes_3B_AFC7D62FB

TGCAGGTGGAGGACGAGCGGGTGCTGGTGATCAGCGGCGAGCGGAGGAGGGAGGAGAAGG

AGGACGCCAAGTACCTGCGGATGGAGCGCCGCATGGGCAAGCTGATGCGCAAGTTCGTGC

TGCCCGAGAACGCCGACATGGAGAAGATCTCCGCCGTGTGCCGCGATGGCG

>Traes_3B_BCECE0C5F

GCAGGAGCTGAGGTGAGATCCTATGGAGGGGAGCGGCGGCGGCAAGCACTTCATCCTCGT

CCACGGCCTCTGCCACGGCGCGTGGTGCTGGTACAAGCTGGTGCCGATGCTCCGCGCCGC

CGGGCACCGTGTTACGGCGCTCGACATGGCCGCGTCCGGCGCGCACCCGGCGCGCATGGA

CGAGGTGGCGTCCTTCGAGGACTACTCGCGGCCGCTGCTCGACGCCGTGGCCGCGGCACC

GGCTGGCGAGAGGCTGGTCCTGGTCGGGCACAGCCTCGGCGGGCTCAACCTCGCGCTCGC

CATGGAGGGATTCCCGCGCAAGGTCGCCGCGGCCGTGTTCCTCGCCGCGTGCATGGCGTG

CGCCGGCAGGCACATGGGCGTCACCATCGAGGAGGTAGGCAGACCCTCAAGAACCCCAGC

CGATTTTTTCATGGACAGCAAGAGCATGGTTCTGAACACAGACCAAGGCCCTCGGCCTGC

AGTCGCGCTTGGCCCCAAATTGTTGGCAGCGAAATTGTACGATCGAAGCTCAGTCGAGGA

CCTGACGCTGGCTATGTTGCTGGTGAGACCGGGCTGCCAGTTCGTGGACGACCCGACGAT

GAGGGACGAGGCTCTACTCACAGACGCCAACTACGGGTCGGTGAAGAAGGTGTACGTGGT

GCTCAAGGACGATGCTTCCACCTCCGAGGAGATGCAGCGCTGGATGGTCGACCTGAGCCC

CGGCACAGAAGCCGAGGAGCTCGCCGGAGCCGACCACATGGCCATGTGCTCAAAGCCCAG

GGAACTCGGCGATGTTCTTCTCAGGATTGCCGACAAGTATGA

>Traes_3B_F269B5E46

GGCAAGGTCGCCGCCGCCGTGTTCGCAGCCGCCGGGATGCCGTGTGTTGGCAAGCACATG

GGCATCACCACCGAGGAGTTCATGCGAAGAAAATCATCCGAAGGACTCATCGACTGCAAG

ATGCTGCCAATCAATAACAACCAGGGTGCGGGGGTTGCAATGATAATGGGCCCAGACTTC

TTAGCACACAAGAACTACCAGCAAAGTTCACCTGAGGATTTGGCCCTGGCAAAAATGTTG

GTGAGGCCGGGAAACCTGTTCATGGAGGATCCGGTGATGAAGAATGCAAGCCTACTCACC

GATGACAACTACGGGTCGGTCAAGAAGGTATACGTGGTAGCCAAGGCTGATGGATCCAGC

ACCGAGGAGATGCAGCGTTGGATGGTGATGTTGAGCCCTGGCACGGAGGTCGAGGAGATC

GCCGGAGCTGACCACGCCATCATGAGCTCGAGGCCTAGGGAGCTCTGTGATGCTCTGGTC

AAGATCGCCAACGGCTTAAATACTTGG

>Traes_3B_BC070E061

CAAAGCTACCGCACTACCAATTCTGAAGGTCGATCCTCTAGAACTTTCTCCATCGGTGCC

CGAAAGACTCCCCGCTGCTCCGCGCTCGTTCCACCACCTTTCAGAAGTTTCCATTACCTT

CCAGACCCCCCGCCTCTGGCGACCTACATAAACTCCCTCTCCTCGCCATCGCATCCCCAC

CAAAGCATTCCACAAAATTCAACAAGCAATCCAACGCAGTCCAACCTGAGGAGATCGATC

GAGCTCAAGATCCGCGAGCGAGAATGGCGGGCATGGTGTTCGGCTTGGAGAACCCAATGA

TGACGGCGCTGCAGCACCTGCTGGACATCCCAGATGGCGAAACCGGTGCCCCCGGCGGCG

AGAAGCAGGGCCCGACGCGCGCCTACGTCCGCGACGCGCGCNCCCCCCGCCGACGTGAAG

GAGCTGCCGGGCGCGTACGCTTTCGTGGTGGACATGCCGGGGCTGGGTTCCGGCGACATC

CAGGTGCAGGTGGAGGACGAGCGGGTGCTGGTCATCAGCGGCGAGCGCAGGAGGGAGGAG

AAGGAGGACACCAAGTACCTGCGGATGGAGCGCCGCATGGGCAAGCTGATGCGCAAGTTC

GTGCTCCCCGAGAACGCCGACATGGAGAAGATCTCCGCCGCGTGCCGCGACGGCGTGCTC

ACCGTCACCGTCGAGAAGCTGCCGCCGCCCGAGCCCAAGAAGCCCAAGACCATCCAGGTC

CAGGTCGCCTGAGATGCATCGTGTGCGCGTCGAATCGAAGCAGAGTGTGAGTGAGTGTGA

GTGAGTAGCGAGTTTCCTGTGATGAGTAATGGAGTGTATGTCTTCTGATATCATCTGCTA

GAGCCTAGAGGGTGTGTGTCGTGATGGTTTGATGTTTCGTTAATGGAAATGGGGATGATC

TCCGGCTTCGCCTGAATAAAACTTGCGATTCTCTTGGCCACATTTTCTATTGAAAAATCA

AAGATGTTCGTTTCTTCTCAGTGTTTTGAAAATTCAGAAAGCTGTGTTTGTAACTTAACG

>Traes_3B_F4D0841D2

AGCTGGTGGCGAAGTGCTCTTTTATGGAGAGCATTCTTTACAACTGCTGTTGTTGCTGTC

GTGTTGAGGACTCTGATTGAGTTCTGTCGCAGTGGAAAGTGTGGTCTCTTTGGGCAAGGT

GGATTGATTATGTTTGATCTAAGTTCAACTGTTGCAACCTATAGTAGTCCGGATCTACTT

GCAATAATTCTCCTTGGAATAATTGGTGGTATATTTGGAGGCCTCTTCAACTTTCTCTTG

GATAAAATTCTTCGTATTTACAGTATTATCAATGAGAGAGGTGCTCCATCCAAGATCCTC

CTCACTATAATAGTATCAGTTATCACATCAATGTGCTCCTATGGCCTCCCTTGGCTTGCT

TCATGGACTCAATGCCCTGAAGATGCCGTGGAGCAATGTCCTACCGTTGACCGCTCTGGC

AATTATAAGAACTTCCAGTGCCCACCAGGGTATTACAATGGTATGGCATCACTTTTCTTC

AATACAAACGATGATGCCATACCCATACGCAATCTTTTCAGCACTGGGACAGCGACTGAG

GGGCACATGTCTAGTCTTTTCATCTTCTTGTTCGCAATCTATTGCCTTGGACTTGTGACT

TACGGAATTGCGGTCCCATCTGATCTCTTCATCCCTGTTATCCTTGCTGGAGCCACATAC

GGTCGAATAATGGGAGCACTTTCGCTGCCACAGCCGACCAGCCATGACCCTCTC

>Traes_3B_3DE5D8BC0

CCAAATCGATGGAGTGCTTTTCTCTGCAGAAGCTTCTCACTTTCAAAGGCCTGAGAGGAT

ACAGACCATACATAGTAATCTTTGACCAGGACTAATATGGTCAAAAAAACAGACGAGATC

ACAGTACACTGAATCTCCACATGGAAAATAATGCACTCCTGTCATATTATTGGCAGAACC

AGCTGGCTCTGCCTTCCACATGCTTGAGAATTGAGTAGCACCACGCACATACGGCAAGTC

AGCATCATCCGGGCAGCCAAATTCAGTGAATCGAATGGCGTGCTCTTCTTTGACCTGCGC

CATCCGACGTCAGCATCATCCGGACCGCCTGGACGCCGCCTTTCTGGAGGGGTTCAGAAC

TTAAGAAGATTCATTCATCCACATGGCCGACGTTAGAGCATGCGTCACTAGCAAGAGGTG

ACCAAGTTGATACGCTGCACTCGTTCCGACGTCAGCTCGTCAACTCAACTTAGGAAGATA

TATATAGCACCAGGACCAGGTGCTATATACACACTACGGTTGCCATTGCACCGCTTCCAA

GCCCTCCCAGCTTGACAGACTCCACTAGCATTTCAGCCGATCAGCCATGACCTTCGCCGG

CAGCGGTGATGGCCCGAGCGGCTCTGCGAGGGCGCACTTCGTGCTGGTACCCATGATGGC

TCAGGGTCATACCATCCCCATGACCGACATGGCACGCCTGCTGGCAGAACATGGCGCGCA

GGTCAGCTTCATCAGGGCTTCGCCGCTGACGTGAAGGCAGCAGGCCTGGCGGTTCAGCTC

GTGGAGCTCCACTTCCCGGCAGCGGAGTTCGGCCTACCCCGGGTGCGAGAACCTCGACAT

GATCCAATCAAAGAATTTGTTCCTGAACTTCATGGAGACCTGTGCTGCGCTGCAGGAGCC

GCTCATGGCGTACCTCCGTGAGCAGCAGCGCTTGCCTCCTAGCTGCATCATATCTGACCT

GGTTCACTGGTGGACTGGTGACATCGCAAGGGAGCTTGATATCCCAAGGCTGACCTTTAA

TGGCTTTTGTGGCTTCTCGTCCCTCATCAGGTACATCACTTATCACAACAATGTATTTCA

AAATGTCAAAGACGAAAATGAGCTCATCACAATCACAGGGTTCCCT

>Traes_3B_25B7D0DF4

CCCAAACAAATTTTACACAGGAAAAATGGAGAGCACTCAGAGCAACAGCGTGAGGAACCA

TTTCATCCTGGTGCACGGCCTCTGCCACGGCGCGTGGTGCTGGTAAAAGGTGGTCGCGGC

GCTTGAGGCAGCGGGGCACCGCGTCACGGCGGTCGACCTCGCCGCTTCTGGCGCCCACCC

GGCGCGCGTCGACGAGGTGCACTCGTTCGAGGACTACTCGCGGCCGCTGCTCGACGTGGT

GGCCGCGGCGCCGGAGGGTGACGGCGAGAGGCTGATTCTGGTCGGGCACAGCCATGGCGG

GCTCAGCTTGGCGCTAGCCATGGAGAGGTTCCCCGGCAAGGTCGCCGCCGCCGTGTTCGC

AGCCGCCGCGATGCCGTGCGTTGGCAAGCACATGGGCGTCCCCACCGAGGAGTTCATGCG

AAGAACATCATCGGAAGGACTACTCATGGACTGCAAGATGCTGCCAATCAGCAACAACCA

GGGTGCAGGGGTTGCAATCATAGTGGGCCCAGACTTCTTAGCACGCAAGTATTACCAGCA

AAATCCACCTGAGGATTTGGCCCTGGCAAAAATGTTGGTCAGGCCGGGAAACCAGTTCAT

TGACGATCCAATGATGAAGGATGCAAGCCTGCTCACCGACGACAACTACGGGTCGGTGAG

GAAGGTATACGTGGTAGCCAAGGCTGATAGCTCCAGCACCGAGGAGATGCAGCGTTGGAT

GGTGGTGTTAAGCCCCGGCACGGAGGTCGAGGAGATCGCCGGAGCTGACCATACCATCAT

GAGCTCTAGGCCAAGGGAGCTCTGTGATGCCCTGGTCAAGATCGCCAACAGCTTATATAC

TTTCTGAAGTGTATGATATATATACCTGAAAGCAAATGTATGTGTGTACATGATTTTCAT

CTAAAAAATAAATATGTCTAGTGAT

>Traes_3B_4CA2211CE

CGACGCCGCCGTCTCCGGCCCGGACGCGGCGCCCGACCTCCGCCTCGCCGCGCACCAGTA

CAACCAGCTCCTCCACCTGCTCGCCTCCGCGGACCGCGCCGCGTTCCCGGCCCACCCGGC

CGCCGCCGCGCGCCGCGTGTTCGCCCACATGCTCGGGGTCGGGGCGCCCCCCTCCGAGGC

CACCATCACCTCGCTCGCCCGCGTCGTCGCCGCCGCGGACGACGCCGAGGGCGCCGACGA

GGCCTTCGGGCTCGTCTCCACCATGCGGGAGAAGTACGGCCTCGCGCCGCGCCTCCGGTC

CTACAGCCCCGTGCTCGCCGCGTTCTGGCGCGCCGGGGAGGCTGGCAAGGCGTACGAGGT

CGAGGCCCACATGGCGGCCTCCGCTGTATCGCCGGAGGAGCCTGAGCTCACCGCGCTCCT

TGAGGTCAGCGCCAAGGCGGGGAACGCAGACAAGGTGTATGAGTATATGCACAAGCTGCG

GCGAGCCGTGGGCTGTGTTAAAGAGGAGACCACAGAGGTGTTGGAGGCATGGTTTCGGAG

CGAGAAGGCGGCGACGGCTGGTAAGGCTGAGTGGGATGTGTGTCAGGTGAAGGACGCCAT

TGTGGCGAACGGCGGTGGGTGCCACCAGCTGGGGTGGCTTCGGACCGGACCGTGGACGGT

GCAGCGGGTGAGAGTTGGGGCAGAAGGCGAGTGCGGCGGATGCAGATGCCGCCTAGCTTC

TGTTGATATCAACATGGAGGATACACAGAAGTTTGCTGACTCTATTTCTGGGTTGGCCCT

TGAGAGGGAGACCAAAGCAAATTTCAGCCAATTTAAGGAGTGGCTGGAAGCACATAAAGA

ATACGAAGCTATAGTGGATGGTGCAAATATTGCACTTTATCAACAAAATTTTGCTGAGGG

CGGTTTCAGCTTGGTTCAGCTGGATGCTGTTGTAACAGAGCTACGAGATAGATATAGTGG

GAAATGGCCACTTGTTATACTACATAATAAGCGCATCGCTAAGCTTATGGAAACTGCATC

CAATAGGCATCTTATTGAAACTTGGAGAGCAAATGGAGCATTATACACATCGCCAAGTGG

GTCAAATGATGACTGGTATTGGCTATATGCAGCAATTGGATTGAACTGTTTGCTCGTGAC

TAACGATGAAATGAGAGATCACATATTTGAGCTCCTAGGATCATCGTCCTTCTTCTACAA

GTGGAAGCAAAGACATCGGGTCAAGTACACTTTTAATAAAGGGAAGGCGGTGCTTGTTCT

GCCACCCCCCTATTCTTCGGAGATTCAAGAATCCGAGACAGGATCTTGGCACGTGCCAAT

AGAGGAGAAATCTGGTGACGAGAGAGCTAGGACCTGGCTTTGTATTGGCAGGACAGATCT

CGCCAAACCACCCCGTGAACCTCCGGTGGCCAACGGAGTTGCCCAGGATTTATCTCCATC

TGAGGCATCTAACGGAGCTGGGCAGAGGCAACCTGAAGAACTGGCTGCACCGGTAACTGG

CAAAAGAAAGGATAGAGATTGATGCCGCTGCCATTTGCAGGCAACTGAAGCATGCCGTAT

TTCTGCAACGCGTTTGGTTCTGCGTCCGCTTTAGTAGTCCAGGAACAGGGATGTGGTGTT

AGCGACATCCCTGACAGCCTGTTTGTGTTTTACAAGCATTTGTAAAGCACACCTGACTTG

TGTGCAGCTGTGCACCAATTTGGTTCAGTTTTCATCGAGTTTGTTTTTTTGGGCAAAAGA

ACTGCATTGTTTCAAAGGATAGTGTTATCATCCATGATCACATTGTACAACAGTGGGAGG

TTAAACTGGTTATACCATTTGTTTTCCGTGCTATGTTTTTCCTTGGCAATGATAGTGTGA

ATAACTGAACAAGAGCGG

>Traes_3B_342E73573

GAGCTACAGTGCGGCTTGCGCTTCATCCATCGCGTCGTCCTCCTCCATACCACGCCGCCG

ATGGCGCTGCTCCGCCTCCACCTCCTCCGGCCGCCGCCTGCTCCGCTCGCCGCCTTCTCC

TCCCCCTGGCGCCGCGCCCTCCCAACCACCAGGCCCGCTGCACCCGCCTCCCGCCTCCTC

TGCTCCAACCACCCCGCCTCCCCCTCCTCCCCCTCCTCGCCCTCCATCGTCGGCGGCCTG

CTCGACTACCTCAACGAGTCGTGGACGCAGTTCCACGCCACCGCCGAGGCCAAGAGGCAG

CTGCTCGCGGCAGGGTTCGAGCTGCTCAGCGAGAACGGCGACTGGGACCTGCAGCCCGGT

GGCCGCTACTTCTTCACTCGCAACATGTCCTGCCTGGTCGCCTTTGCAGTCGGGCAAAAG

TACAGAGTTGGTAATGGTTTCAATATAATTGCAGCCCACACTGATAGTCCATGCCTCAAG

CTGAAGCCAAGGTCTGCTTCCTTCAAATCTGGCCATCAAATGGTAGGTGTGCAGACATAT

GGAGGTGGGTTGTGGCACACATGGTTTGATAGAGATCTAACTTTGGCCGGGCGAGTCATC

CTCAAGGCTCCAGATGGTTCATTTAAGCATAAGCTTGTCAAAGTGAACAGACCACTCATT

CGCGTACCGACACTGGCTATACATCTTGACCGCACAGTGAATTCTGAAGGATTCAAGCCT

AATCTAGAGAGTCATCTGGCTCCACTTCTTGCAACAAAATGTGAAGAAACCACTGTCAGT

TCTGATGACAAAAAAGGTTCAAGTTCCACAAGGACTGTTCATCATCCACTACTATTGCAA

GTTCTTTCAGAAGAAATTGGTTGTGGATCGGATGAAATTATTGGTATGGAGTTGAACGTG

TGTGATACCCAACCTAGCTGCCTTGGTGGAGGCAAGAACGAGTTCATTTATTCTGGTAGA

TTGGATAATCTTGCTTCATGTTATTGTGCGCTGAAATCCCTCATGGACTCTTCCAAGATA

CCAGAAGAGTTATCCAACGAGAAGGGTATAAGAATGATTGCGTTGTTTGATAATGAAGAG

GTTGGTTCAAATTCAATGCAAGGGGCAGGTGCACCAACCATATTCCAGGCCATGAGACGA

ATTGTTGACTCCTTGATGCATCAGTCCATGGGGGAGGGGGCTTTAGAGCGTGCACTAACT

TCTTCTTTCCTTGTTTCCGCGGATATGGCTCATGCCCTGCACCCAAACTATCCAGACAAG

CATGAAGAGTACCACAGACCTGAACTACAAAACGGACTTGTTATCAAGCATAATGCTAAC

CAACGTTATGCCACAAGTGCTGTAACAGCTTTTCTCTTCAAAGAAATAGCTCGAATTCAT

AACCTTCCCGTCCAGGAATTTGTTGTAAGGAATGATATGGGTTGTGGCTCAACTATCGGT

CCCATACTTGCTTCCGGTGTTGGCATACGGACTGTTGATTGTGGTATTCCTCAGCTTTCC

ATGCACAGCGTTCGGGAAATGTGTGGCAAAGAAGACATAGACACCACATACAAGCACTTC

AAAGCTTTCTTCGAGATGTTCTCGGACATTGACCAGAAACTGAATGTAGACTTTTAGCTC

TTGGCATCTTAATGTGCAGTCTCTTAATAATGTATATGGATATTCATCTCGGCCTCTGAA

TAATATCTTGATCATGGTATCAGTTTTTGTTGTATATCCAATCTGGTTTAAGACAAGAGC

ACTGGCTTTGTACCTTAAATTTCTGAAGCTAATTGTTTCTCCTTGTTACTGATCACAGGT

AACGTTAAATTTCCA

>Traes_3B_651650859

CGACAAACACCTCACCACCACGACCCATCTCAGTAGTGTGCTCGATCTAGCAAGAAGGAC

GATCCATGGCGCGGCAACTGGTTGCTGCGCTGGCCGCTCTGGTGGCCGTGTGCGCCCTGG

CGGCCGCCGCCGGCGTCCAGGCCCAGCCGCCGAGGAAGCTGCCCCCCAACTACCACATGA

TCAGCCCGGGGAAGTTCGGGCAGAGGAGCCAGCAGCTGTCGTGCAAGGACACCAACGGCA

GGAAGGCGGGCTGCATGGGCAAGTGCGACAAGCGCTGCCCCAACCAGTGCATCGTCCTCT

GCCCCAGCTGCAAGACCTACTGCATGTGCGACTTCTACGCCGGCGTGTCTTGCGGTGACC

CGCGCTTCACCGGCGGCGACGGCAACAACTTCTACTTCCACGGCAAGAAGGACCAGGACT

TCTGCATCGTCTCCGACGCCGACCTCCACATCAACGCCCACTTCATCGGCAAGCGCAACC

CCTCCATGAGCCGCGACTTCACCTGGATCCAGGCCCTCGGCATCCGCTTCGCGCACCACC

GTCTCTTCATGGGAGCCCAGAAGACCACCAGCTGGAACGGCGACGTTGATCACCTCGAGA

TGTCCTTCGACGATGCGGCCATCGAGATCCCGATCGAGGCCGGCGCACGGTGGCAGTCGG

CCGCGGTTCCAGGGCTGACCGTCACGAGGACGGCGGCGACCAACGGAGTGAGGGTGCACC

TGAGGGGCGTGTTCGACATCATGGCCAACGTGGTGCCCATCACCGAGGAGGACTCGCGCA

TCCACAACTACGGCGTCGCTGGGTCAGGAGACAGCCTCGCGCACCTCGACATTGGGTTCA

AGTTCCACGACCTCACCGACGACGTGCACGGCGTGCTCGGCCAGACCTACCGCTCCGACT

ACGTGAATGAGATGAGCGTGCGCGCCAGCATGCCCATCATGGGTGGTGCGCCAAACTATG

TCTCCTCCGACATCTTTGCCGCCGACTGCGCCGTTTCCAGGTTCGGCCGCAACACCGGCA

TCTCCATGATGACCGGCAAGACCGACTGAACTATACAGATACATTGCAGACGTGAGCTTT

CCAGCCACCTCTACTACACTACTACACCCCTGACCACACAATGGCAAAAATTTCTGCTTC

GCGCAATCAACCAAATTAGTGGTGTAATGAGAAGGGAAATAAAAATGTAACGCAAGATCA

ACCTCAACCGTTGGCTG

>Traes_3B_5B906C21B

CTCGTGGTCGCCGACGCCGTCGTCGTCGTCGACCGCGAGCAGGGCGGGAGGGAGAACCTC

GCGGCGAACGGGGTCACGCTGCACTCCCTCATGACGCTCACCGAGGTGCTCGCCGTGCTC

GTCAGCCACGGCAAGGTCACCCGGGAGAAGGCCGCCGAGGTGAAGCGCTTCCTCGACGCC

AACAGGAAGGTCTTGGTGCCGGCGGAGACCAGGGTGGTGTCGGCAAGAGGGATGGCCTTC

GCCGAGAGGGCGAGGCTGGCCAAGAACCCGGTGGGGAGGAAGCTGCTCGAGGTCATGGAG

GCCAAGCAGAGCAACCTCTGCGTCGCTGCCGATGTTACAACAGCAAACGAACTTCTTGAG

CTAGCCAACAAGGTTGGTCCGGAGATTTGCATGCTGAAAACCCATGTGGATATTTTGTCT

GATTTTACACCAGATTTTGGTGCCAAGCTCCGTTCGGTATGGCCATTTATGATGCAACCT

TTATTTTCCTTCTACCATCCTTATTATTGATGAATTTTTCTCACACTTATGTCTGCTAGT

AGACTAGTATGCCATACTAGTCCAGTACTGAGTTGGAGTTTGTTTGTCAGTAATCAGATG

TTACATCATTTTCTAAAAAGAAAAGAAATATTGTCGAAGTATCTTCGTTCAATCATGCCT

ATCAGGTGTAGAAAATAAACCAGTTGTAAGTTCAGTACAGAGTATCTTTGTTAACAGTAC

TGTTCTATCAGGAAGCATGTTTCACTTCTGCACTTGTTAAAATGTGGAAGTGTGCTTTTT

GCTCATCAGTTCTGTACGCCCACAAATAATGCGTTCGCATTTGGGCACAATAGACCTCTA

CCTGATTGAATTTTGCGATCTGTTTTCTACTGCAGAAATGTTCT

>Traes_3B_B021B5C15

GTTCAGCTCGTGGAGCTCCACTTCCCGAGTGTAGAGTTCGGCCTACCAGATGGGTGCGAG

AACCTCGACATGATCCAATCAAAGAATTTGTTCTTTAACTTCATGAAGGCATGTGCCGCG

CTGCATGAGCCGCTCATGGCGTACCTCCGTGAGCAGCAGCGCTCCCCTCCTAGCTGCATC

ATATCTGACATGGCGCACTGGTGGACCGGTGACATCGCAAGGGAGCTCGGCATCCCGAGG

CTCACCTTTAGTGGCTTTTGTGGCTTCTCGTCCCTTGTCAGGTACATCATTTTTCACAAC

AATGTATTGGAGCATGTCACAGATGACAATGAGCTCATCACGATCCCGGGGTTCCCTACA

CCGCTAGAGTTGACGAAGGCTAAGTTGCCTGGAACCCTTTGTGTTCCGGGTATGGAGCAA

ATCCGTGAGAAGATGTTTGAGGAGGAGCTGAGATGCGATGGTGAGATCACCAATAGCTTC

AAAGAGCTCGAGACATTGTACATTGAATCCTATGAGCAGATAACAAGGAAGAAGGTCTGG

ACGATCGGGCCAATGTGCCTCTGCCACCGAAACAGCAACAGAACGGCCGCAAGAGGAAAC

AAGGCGTCAATGGATGAGGCACAGTGCTTGCAATGGCTTGATTCAAGGAAGCCAGGCTCA

GTGATCTTTGTGAGTTTTGGAAGCCTCGCTTGCACTACACCTCAACAACTTGTTGAACTG

GGACTGGGACTTGAAGCCTCCAAGAAACCATTTGTTTGGGTGATTAAAGCAGGAGCTAAG

CTTCCAGAAGTCGAGGAATGGCTCGCAGATGGGTTCGAGGAGCGCGTCAAAGACAGAGGT

CTGATCATAAGGGGTTGGGCACCACAGCTCATGATCCTGCAGCACCAAGCCGTTGGAGGA

TTCGTGACGCACTGCGGGTGGAACTCAACA

>Traes_3B_C1A0BCA34

GTTCAGCTCGTGGAGCTCCACTTCCCGAGTGTAGAGTTCGGCCTACCAGATGGGTGCGAG

AACCTCGACATGATCCAATCAAAGAATTTGTTCTTTAACTTCATGAAGGCATGTGCCGCG

CTGCATGAGCCGCTCATGGCGTACCTCCGTGAGCAGCAGCGCTCCCCTCCTAGCTGCATC

ATATCTGACATGGCGCACTGGTGGACCGGTGACATCGCAAGGGAGCTCGGCATCCCGAGG

CTCACCTTTAGTGGCTTTTGTGGCTTTTCGTCCCTTGTCAGGTACATCATTTTTCACAAC

AATGTATTGGAGCATGTCACAGATGACAATGAGCTCATCACGATCCCGGGGTTCCCTACA

CCGCTAGAGTTGACGAAGGCTAAGTTGCCTGGAACCCTTTGTGTTCCGGGTATGGAGCAA

ATCCGTGAGAAGATGTTTGAGGAGGAGCTGAGATGCGATGGTGAGATCACCAATAGCTTC

AAAGAGCTCGAGACATTGTACATTGAATCCTATGAGCAGATAACAAGGAAGAAGGTCTGG

ACGATCGGGCCAATGTGCCTCTGCCACCGAAACAGCAACAGAACGGCCGCAAGAGGAAAC

AAGGCGTCAATGGATGAGGCACAGTGCTTGCAATGGCTTGATTCAAGGAAGCCAGGCTCA

GTGATCTTTGTGAGTTTTGGAAGCCTCGCTTGCACTACACCTCAACAACTTGTTGAACTG

GGACTGGGACTTGAAGCCTCCAAGAAACCATTTGTTTGGGTGATTAAAGCAGGAGCTAAG

CTTCCAGAAGTCGAGGAATGGCTCGCAGATGGGTTCGAGGAGCGCGTCAAAGACAGAGGT

CTGATCATAAGGGGTTGGGCACCACAGCTCATGATCCTGCAGCACCAAGCCGTTGGAGGA

TTCGTGACGCACTGCGGGTGGAACTCAACA

>Traes_3B_111A725EF

CACAAAACAAAACAAATTTTACACAAGAAATGGAGAGGATCCAGAGCAGCAACAGCAACA

GCGTGAGGAACCATTTCGTCCTGGTTCACGGCCTCTGCCACGGGGCGTGGTGCTGGTACA

AGGTGATCGTGGCGCTCGAGGCCGCGGGGCACCGCGTCACGGC

>Traes_3B_63A565480

CTTGAAAGACTCCACTAGCACTTTTGCCGGCCATGGCCTTCGCCGGCAGCAGCGACGGCC

AGAGCGGCTCCGCGAGGGCGCACTTCGTGCTGGTGCCGATGATGGCTCAGGGCCATACAA

TCCCCATGACCGACATGGCACGCCTGCTGGCAGAGCATGGCGCGCAGGTCAGCTTCATCA

CCACGCCGGTCAACGCCGCTAGGTTGGAAGGCTTCGCCGCTGAGGTGGAGGCGGCGGGCC

TGGCGGTTCAGCTCGTGGAGCTCCACTTCCCGAGTGTAGAGTTCGGCCTACCAGATGGGT

GCGAGAACCTCGACGTATATGTCATCATCAAGGGCGGAAGAACTGATAATAGAGCGTTAG

AGCAGAGTTGGACGACCGGTTTTGCCAAATTATGGGCCCTAGAACTCCTCTTGGTGCCGC

CATTTGCCACGGCTGTGCATAGCTAGAGCAAAGGGTGGAAGGAGAAACGACTTGGCACAC

ACGCACTAGTCGGATGCTTTGCCGCCACTGGCGTCCATCCCACTACAAGAAAAAGTCATT

CGTAGCCGAGTGCTTATACTAACGTCGAATGTTATTTGTCAGATACTCTTTTATAGACAC

CTAAACCGAGTACCCTCGAAAAAGGGGTCCAGCGCGAGGTGGAGGACAAG

>Traes_3B_C22C4F578

TTGCCATCCCTAGCCCGTGCCAGTGAGATACTAGTACACGAAGCTGCAGCCACGAGCCGC

ACAGCGCCGGCAACGTAGCGGTCTCTCTCGTCTCATCGGAGGTGAGATCCGATGGAGGGG

AGCGGCGGCGACCACTTCATCCTCGTCCACGGCCTCGGCCACGGCGCGTGGTGCTGGTAC

AAGCTGGTGCCGATGCTGCGCGCCGCGGGGCACCGCGTCACCGCGCTGGACATGGCGGCG

TCGGGCGTGCACCCGGCGCGCATGGACGAGGTGCCGTCCTTCGAGGACTACTCGCGGCCG

CTGCTCGACGCCGT

>Traes_3B_3872F3D65

GCGAGAGGCTGGTCCTGGTCGGGCACAGCCTCGGCGGGCTCAACATCGCGCTCGCCATGG

AGAGGCTCCCGCGCAAGGTCGCCGCGGCCGTGTTCCTCGACGCGTGCATGCCGTGCGTCG

GCCGGCACATGGGCGTCACCATGGAGGAGTTCTCCAGAAGAACCACGCCGGATTTTTTCA

TGGACAGCGAGAGGATGGTTCTGGAGACGAGCGAGGGCCCTCGAGCTGCACTCGTGTTTG

GCCCCAAATTGTTGGCAGCAAAACTGTACGGTCGAAGCCCAGTTGAGGACCTGACGCTGG

CCACGATGCTGGTGAGGCCCGGCCGCCAGNNNNNNNNNAAGGACGAGACGCTGCTCACCG

ACGCCAACTACGGGTCGGTGAAGAAGGTGTACGTGGTGGCCATGGAGGACGTTGCTTTCT

CCGAGGACATGCAGCGCTGGATGGTCGACCTGAGCCCCGGCACGGAGGCCGTGGAGATCG

CCGGAGCCGACCACATGGCCATGTTCTCCAAGCCCAGGGAGCTCTGCGATGTTCTGCTCG

GGATCGCCAGCAAGCATGACTGAGAATCAACTAACGGGCACATGATCGGTCAATAATCCT

TGTCGCGGTGACAGACATTTCGAGTGTTGTGGTTGTGTATGACTGTACGTCCGGCCTTGA

GGTTTGTGTGTCACGCCGAATTTGAAGGATATAGGAACGAGCTTGTCACAACCTTAATTC

GATGGTGTCACACCAAGCGAATTTGGTGAAGGGATTCAGTATCATATGAGAGTACAAACA

CAGAAGTTTTGGGAGTAAAGAAGAACAGTAGAGGGGAAAAGTTCAGGCTACAGCCAACTG

ATTCGATCCATTCATCCTTGCCTACTCCACGTT

>Traes_3B_E9CFE37D3

GTGAAAACCAGGAGGTTATGGTTACCAGAGATGCTGTGGAGACAGCAGTGAACACCCTGA

TGGGTGAAGGGGAGGCTACAGAGGAGTTGAGAATGAGAGCAGAAGACTGCGCCATTAAGG

CAAGGAGGGCTTTCGACGAGGAAGGTTCTTCTTACAACAACGTAAGGCTGTTAATTCAAG

AAATGGGAAACAAGACGAATGCATGTGGTTGATAGAGATTACCTAAGCTCGCTTTTTCAG

TGTAAACATGAAGTGAAACATCCTAGAGTACATATATCATCATATAGAATTTTGTACCAG

GCTCACAGGTGATTATTTAGAGATTATAGATGATCCAAATGAAGGGAATATCAATTATCC

TCTCTATTATGTACGCTCTTTCATGGCAGTCCACATAACAACTACACAGTCTGCTATTCT

AGATGGTTACGCTCTCCCTTGCATTTTGCTACCGCTGCAC

>Traes_3B_34C1F24FA

ACCATTATCAGATTGTGCCCAACACATCGTGCTATCTTGCCGGTGTCACTGTCTTCCCAG

TTTGACTGACTCCACCACCAGTCCTTTTCCTACAATAGCCGATCAGCCATGACCTTCACC

GGCAGCGGTGATGGCCACAGCGGCTCCGCGAGGGCGCACTTTGTGTTGGTACCGATGTTG

GCGCAGGGGCACACAATCCCCATGACCGACATGGCGCGCCTGCTGGCAGAGCACGGCGCG

CAGGTTAGCTTCATCACCACACCAGTGAATGCCTCCAGGTTGGCAGGCTTTGCAGCCGAT

GTGGAGGCGGCGGGCCTGGCAGTACGGCTCGTGGAGCTCCATTTCCCGAGCGCCGAGTTC

GGCCTACCGGATGGGTGCGAGAACCTGGACATGATCCAATCCACGGATCTGCTCTCAAAC

TTCATGGATGCCATCGCTGCGCTTCAGGAGCCGCTCAAGGCATACCTCCGTGAGCAGCAG

CGCTTGCCTCCGAGCTGCATCATATCTGACCTGATTCACTGGTGGACTGGTGACATCGCG

AGGGAGCTTGGTATCCCGAGGCTGACCTTTAGTGGCTTTTGTGGCTTCTCGTCCCTCATC

AGGTACATCACTTATCACAACAATGTATTTCAAAATGTCAAAGACGAAAATGAGCTCATC

ACAATCACAGGGTTCCCT

>Traes_3B_35DBC7F70

CACAGGAAATGAAGAGCACTCAGAGCAACAGCAGCAACAGCGTGAGGAACCATTTCATCC

TGGTGCACGGCCTCTGCCACGGCGCGTGGTGCTGGTACAAGGTGGTCGCGGCGCTTGAGG

CAGCGGGGCACCGCGTCACGGCAGTCGACCTCGCCGCTTCCGGCGCCCACCCGGCGCGCG

TCGACGAGGTGCACTCGTTCGAGGAGTACTCGCGGCCGCTGCTCGACGTGCTGGCCGCGG

CGCCGGAGGGTGACGGCGAGAGGCTGATTCTGG

>Traes_3B_C8EB1373F

TCGCGCTAACGATGGAGAGGTTCCCCGGCAAGGTCGCCACGGCCGTGTTCGTGGCAGCCG

CGATGCCGTGCGTTGGCAAGCACATGGGCGTCACAACCGAGGAGTTCATGAGAAGAGCAT

CATCGGAAGGACTGCTCATGGACTGCGAGATGCTGGCAATCAACAACAACCAGGGTGTAG

GTGTTGCAATCATAGTGGGCCCAGACTTCTTAGCTCACAAGGGTTACCAGCAAAGTCCAC

CTGAGGATTTGGCCCTGGCAAAAATGTTGGTGAGGCCTGGAAACCAATTCGTGGATGATC

CAGTGATGAAGGACGCAGGGCTGCTCACCGAACGCAACTATGGGTCGGTGAAGAAGCTAT

ACGTGGTTGCCAAGGCAGATGTTTCCAGCACTGAGGAGATGCAACGCTGGATGGTGGTTC

TGAGCCCTGGAACGGAAGTCGAGGAGATTGCAGGAGCTGACCACGCCATCATGAGCTCCA

AGCCCAAGGAGCTATGTGATGTCCTGGTCAAGATAGCCAACAACTTCAATATTTAGCAAA

TTGGAACAGAAATGTTGTTTGAAATAAATTCATGTATCACGTATGTAATTAAATCTCTAG

AGATGTGTTTATGATATACATTTGATGTGGGTATG

>Traes_3B_678103DC7

CCCGCCGACGTGAAGGAGCTGCCGGGCGCGTACGCGTTTGTGGTGGACATGCCAGGTCTC

GGGTCCGGCGACATCAAGGTGCAGGTGGAGGACGAGCGGGTGCTGGTGATCAGCGGCGAG

CGCAGGAGGGAGGAGAAGGAAGACGCCAAGTACCTGCGGATGGAGCGCCGCATGGGCAAG

CTGATGCGCAAGTTCGTGCTCCCCGAGAACGCCGACATGGAGAAGATCTCCGCCGTGTGC

CGCGACGGCGTGCTCACCGTGACCGTCGACAAGCTGCCCCCGCCCGAGCCCAAGAAGCCC

AAGACCATCCAGGTCCAGGTCGCCTGAGATGCGTCGTGTGCGCGTCGAATCGAAGCAGAG

TGTGAGTTGGTACTTGGTAGCGAGTTCCGTGTGACGAGTGATGGTTTGATGTTTCGTTAA

TGGAAATGGGGATGTTCTCCGTCATCGCCTCAATAAAACATGCGAATCTTTTGGCCACAT

TTTCCATTGAAAAATCCAAGATGTTCGTTCGTGCTCAGTGTTCTGAAAATTCAGAAAGTT

GTGTTTGTAACTTAACGGAAAACTGGATAAGATCAGAACATGTGGAACTAGCAAAATTAG

GTGGAAAATGAACTAGTTCTGAAGTGCTTTTTTCAGCAGGGAGAAAATTCCTGGATGTCT

TGGCATTTTCATATATGAGGATATATGAGATGACCGATTCAGTGTATTATGAACTGATTT

TCAAATGCATTTATTTT

>Traes_3B_43C55F921

ACTCAGAGCAACAGCAGCAACAGCGTGAGGAACCATTTCATTCTGGTGCACGGCCTCTGC

CACGGGGCCTGGTGTTGGTACAAGGTGGTCGCGGCGCTTGAGGCAGCGGGGCACCGCGTC

ACGGCGGTCCACCTCGCCGTTTCCGGCGCCCACCCGGCGTGCATCGACGAGGTGCACTCG

TTCGAGGAGTACTCCCGGCCGCTGCTCGACGTGGTGGCCGCGGCGCCGGAGGGTGATGGC

CAGAGGCCGATTCTGTTCGGGCACAGCCATGGCGGGCTCAGCTTGGCACTAGCCATGGAG

AGG

>Traes_3B_58309CFB2

GCCGCCCTCCTCGGCTCCCTCCCCTCCACGCGCCCCTACGGCCTCCTCTGCGGCGTCCCC

TACACGGCCCTCCCCTTCGCCTCCGTGCTCTCCGTCGCCCGCGGCATCCCCATGATCCTC

CGCCGCTACCACGAGACCGGCGCCGGCGCCGCGCCCGCCATGCGCACCCAGGGCTCGTTC

CGCGCCGGGGAGACCGTGCTCATTGTCGAGGACCTCGTCACCAGCGGCGCCTCCGTGCTC

GAGACCGTCGCGCCGCTCCGCGCCGAGGGG

>Traes_3B_8DD5BF39D

GATATAAACTCCCTCCGCCCCCCGTCGCATCATCACCGAGGCATTCCACAAAATCAGCAA

GCGATCCAACGCAGTCCAATCGGAGGAGACCGATCGATCGAGCTCGGAGATCAGCAGCGA

GCGAGAATGGAGGCCAGGATGTTCGGGCTGGAGACCCCGCTGATGGCGGCGCTGCAGCAC

CTGCTGGACATCCCGGACGGCGAGGNCCGACGTGAAGGAGCTGCCGGGCGCGTACGCGTT

CGTGGTGGACGTGCCGGGGCTCGCGTCCGGCGACATCAAGGTGCAGGTGGAGGACGAGCG

GGTGCTGGTCATCAGCGGCGAGCGCCGGAGGGAGGAGAAGGAGGACGCCAAGTACCTGCG

GATGGAGCGCCGCATGGGCAAGCTGATGCGCAAGTTCGTGCT

>Traes_3AL_12D60DA8A

CAACCACCTATGGGAAGCACCGTCGTGCTCTACACATGGATGGTCAGGGGTCACCTCCAC

CCCATGACGCAGCTCGCGAATCACCTCGCCGGACATGGCGCTCCCGTCACCGTCGCCGTC

GCCGATGTTCCGTCCACAGGTGACTCCTCACAAACTATTGCCCGCCTCTCTGCCTCCTAC

CCTGCTGTATGCTTCCACCTGCTCCCGCCGGCAACAACCCGCTCCGAGGACGCGGCCGAT

CCCAACGCCGACCCTTTCATCACCCTCATCGCCGACATCCGCGCCACCAACGCCGCCCTC

CTCGCCTTTCTGAGGTCTCTCCCGTCTGTGAAGGCTCTCATCACCGACTTTTTCTGCGCG

TACGGGCTTGACGCAGCCGCAGAGCTCGGTGTCCCAGCCTACGTGTTCTTCACCCTTTGC

GTGTCGGCTCTTGCCACCTTCCTGCATATCCCCGTCATGCGCTCCGCCGTCTCCTTCGGG

GAGATGGGGCGCTCCTTGCTGCACTTCCCTGGAGTTCACCCAATTCCGGCGTCCGACTTG

CCGGAGGTCCTGCTCGATCGTGACAACAGGCAGTACAGTACCACTCTTGGTCTCTTCAAG

CAGCTTCCCAGAGCGAAGGGCA

>Traes_7BS_1B0DC486F

CATGACCCTACTTTCAAGGATATATATCCAGAACCTGCACAAATAAGCAAAGGATATGGA

GAAGAAATTGTCAAGCGTCAGTTACAAGCTGTCGCTGAAGGTGTGGCAGCATCTGTTCTG

CAGTCACCNCACCATTTCCTGAAAAACCAACTGAATTTTCTGGGGATCACAAAGATTTGC

CTGGAGATGTATTTGATCCAAAAAATGAGGATGCGCCGAGCAAACAGTCAGACAAAACAA

GCCAAGGAGTTCCAGTTCTAGATGACATCGATAACCTTCAGATAATAAAGAACAGTGATC

TTGAAGAATTGCGTGAACTAGGTTCTGGAACCTTTGGTACTGTTTACCATGGAAAATGGA

GAGGTTCTGATGTCGCTATAAAAAGGATAAGCGATCGGTGTTTTGTTGGAAAACCTTCTG

AGGAACAGCGCATGGTCCGTGTTAAACTTATGGCC

>Traes_4DS_2F3830A08

GTAAGAAGAAAATTTCTGCTAGAAGAATCAATCGAGCGAAATGAAGCGCTTGGTCTCGAT

TGTTTTACATACGGTTACTACCTGGCATACTTCTGGATGAACAACAGGATGACTAGAGAT

GCTCTCGGGATCAAGGGGGGAACAGTTGGCGAGTGGGTGAGATGCAAAAAAGAACTCCCC

TACACACAGGACATGCCAAGCAGCATACCGTACCATCTTAATCTCACCACGAGAGGTTAC

CGTGCACTCGTATACAGCGGAGACCATGATCTCCAGGTGCCTCAGCTCAACACGCAGGCG

TGGATAAGATCTTTGAACTTCTCCATCGACGATGACTGGAGGGCATGGCATCTCGATGGC

CAGGCTGCAGGGTTGAGTCACCTGACTCCCTTTTTTG

>Traes_2DL_787806003

CGCCGTCCCGTTCGACTTCGCGGGCGTGTGGCGCGCGATGGAGGAGTGCCACCGCCTCGG

GCTCGCCAAGGCCATCGGCGTCAGCAACTTCACCACCAGCCACCTCCACAAGCTCCTCGC

CGCTGCCACCGTCCCTCCTGCAGTCAACCAGGTGGAGATGAACCCGGTCTGGCAGCAGAG

GAAGCTGAGGGAGTACTGCGCCGAGAAGGGCATCCACGTCGCGGCCTACTCGCCGTTGGG

AGGGCAGAACTGGTCCGGCG

>Traes_2DL_B250957E6

AAGACATTGGTTGCATTGGAGCATCTGAATTTGTCTCACACACACATTGTGAGGTTACCT

GAGCGCCTATGGTTACTAAAAGAGTTGAGGCATTTGGATCTGAGTGTGACTACTGCACTT

GAAGATACCTTGAACAACTGCTCAAAGTTGCACAAGTTGAAAGTGCTCAATCTCTTTCGC

AGCCGCTATGGTATCCGTGATGTTGACGACCTGAATCTGGATTCCCTGAAGGAACAACTA

CTCTTCCTCGGAATCACTATTTATGCAGAGGATGTGCTAAAGAAATTGAACATGCCTGGT

CCTTTGGCAAAGTCAACACATCGCTTAAACTTGAAGTATTGTGCAGATATGCAATCAATC

AAAATC

>Traes_4DS_C31EAB9FE

CGGCGTCGGCGTCGACGGTGGTCACCCACCTGCCAGGATTCGATGGCCCTCTCCCCTTCT

ACCTCGAAACCGGATACGTGGGCGTGGAAGAGGAGACCGGGGCGGAGCTCTTCTACTACT

TCGCCGAGTCGGAGCGGAGCCCCGGCACGGACCCCGTCATCCTTTGGCTCACCGGCGGGC

CTCGCTGCTCAGGCTTCAGCGGCTTCGCCTTCGAAGTTGGTCCGGTAAAGTATGTGCTGG

CACCGTACACTGGCGGTTTGCCGCAGCTGGTACATAACCCGCTGTCATGGACCAAGATGG

CGAGCATCATCTTCCTGGATTCGCCGGTCTGCTCGGGCTTCTCGCATGCTCGTGACCCCA

AAGGCTGCGATGTCGGAGACTACTCGTCCTCTCTGCAAGTCCAAAGATTCCTGAATAAGT

GGTTCACTGATCACCCA

>Traes_3AL_31537DF71

CGCCACTCCCGCACGGGGTGCCGGAGGGCGACCATGTGGCCAAGGTTTTCGAGCTTACAC

GCGCTTCCAACCCTGAGCTCCGTGATTTCCTCCGCGTCACCTCCCCGGCGGCCCTCCTCC

TCGACTTCTTCTGCTACAGCGCCGCCGATGTCGCGGCAGAGATCGGCATCCCGGCGTATT

TCTTCTTCCAGAGTTGCACGGCCAGCCTGGCTGTATTGCTCCACCTGCCCGTCATCCACG

GGCAA

>Traes_2DS_BD02F5838

GCCAGCAATGTGCTCCTGGACTCGGACTGCAACCCCAAGATCTCGGACTTCGGCCTGGCA

AAGCTGTTCGGCTGGGATCAGTCGCAGGCCGTCACCAGCCACATCGCCGGAACATAACGT

TTATTTACACGACGGGATTTTTGCTCGTGTTGCACTTGCACGCAGCGGATACAT

>Traes_2DS_16A6C32B8

GATGGTGGAATACAACTTCTCTGCAGTCTGCACTACACGTGTCTGAAATCCATGTCATTT

GTTTGCAGAACGAACAAGAAATAGGACAGGGTTGATCTTGGCCATCACACTACCTATAGT

TGCTGCACTACTTCTCATTTCAACGTGCGTTTGCTTTTGGAGGAGGAGAAAATCGGCAGA

AAGAAAGCCGTCAGTACCACTACCATATTCAGCTACTAATCCAGATGACATCCAAAGCAT

CGATTCCCTCCTCCTCGACCTATCGACGCTGCGGGCTGCAACAGATAACTTCGCTGAGAG

CAACAAGCTTGGCGAAGGAGGGTTCGGTGCGGTTTACAAGGGTGTCCTTTCTGAAGGTGA

AGAGATAGCCGTGAAGAGGCTGTCACAGAGCTCCACGCAAGGGATAGAAGAGCTGAAAAC

AGAGCTGGTTCTGGTCGCTAAGCTTCAGCACAAGAACCTTGTCAGGCTCCTTGGCGTTTG

CCTGGAAGGACAGGAGAAGCTGCTCGTGTACGAGTACATGCCGAACCGGAGCCTCGACAC

CGTTCTATTCGATGCAGAGAAAAGCAGGGGCCTGGACTGGGGGAAGAGGCTCAAGATCGT

GAACGGGGTTGCCCGGGGCCTGCAGTACCTCCACGAGGACTCCCAGCTGAGGATCGTCCA

CCGGGACCTCAAGGCCA

>Traes_4DS_5439B7759

GGAAATTATGTAAACCCCGCGAATCAACTGTGTGCTGAGGTGCTAGAAACTGTAGACAGT

CTCATTTCTGAAATCACAGATGCACACGTCCTTTACAAAAAATGTGTCGTCGCCACGCCA

AAGCCCATAGATGATGCTGTAAGAAGAAAATTTCTGCTAGAAGAATCAATCGAGCGAAAT

GAAGCGCTTGGTCTCGATTGTTTTACATACGGTTACTACCTGGCATACTTCTGGATGAAC

AACAGGATGACTAGAGATGCTCTCGGGATCAAGGGGGGAACAGTTGGCGAGTGGGTGAGA

TGCAAAAAAGAACTCCCCTACACACAGGACATGCCAAGCAGCATACCGTACCATCTTAAT

CTCACCACGAGAGGTTACCGTGCACTCGTATACAGCGGAGACCATGATCTCCA

>Traes_7DL_5E66FFB18

CGGCGTGGTGCTGCTGGAGCTGGTGACCGGAAGGAAGGCGATCCACCGGGACCAGAGCCA

GGAAGGGAGCGGGTCGCCGAGGAACGTGATCGAGTTCGCGGTGCCGGCCGTGGAGGGCGG

AAACATCGACAAGATCCTCGACAGCCGGGTGCCCGC

>Traes_7DL_540E96109

CCCCGACGGGCGGGAGGTCGCCATCAAGCGCGCCGAGCGCGCGTCCACCGGTGCGCGGCG

GCGGAGGCGGTTCGACGCGGAGCGGGCGTTCCGGTCGGAGCTGAGGCTGCTGTCCCGCGT

CAACCACCGCAACCTGGTGTCCCTCCTGGGCTTCTGCGAGGAGCGCGGCGAGCGCATCCT

CGTGTTCGAGTTCATGCCGCACGGCGCGCTCCACGACCACCTCCACGGCGAGGACGCCGG

GTCCGGCCACTCCCCGCTCTTCTCGTC

>Traes_4DL_20D3C0753

CTGGAGGTGGTGGAGCGGAGCCACATGAGCGACGCGTCCGCCGCGCCGCTGCGGTTCCGA

TCGCCGCTGTCGCTCCTGTTCGCGAGCATCAGCAAGGGGAACGTGTGCGTGGCCGGCGAC

GCGCTGCACCCTATGACGCCGGACCTGGGCCAGGGCGGCTGCTCGGCGCTCGAGGACGGC

GGCATCCTGGCCA

>Traes_6BL_D412AEAC9

GCCTACGCCTGCGTCAGCAGCAACAGCGAGTGCGTCGACTCCAAGTACGGACGGGGCAAG

GGATACCTCTGCAACTGCTCCGCCGGCTACGACGGCAATCCCTACCTCCTCGACGGCTGT

CAAGATATCAACGAGTGCCAAGACGAACGGTACCCCTGCTCTGTTCCGGATACGTGCGTC

AATACCATCGGAGGATACAGCTGCGTTTGCCCTGAAAAGACATCCGGCAACGCATACAAC

GGAACATGCGAGCAAGACAAGTCTCAGATTGGGTGGGAGATCGCCATTGGAGTCAGCATT

GGTGTGATCGTACTGATAGCCGCCGCTTCGTGTGCTTACATGATCTACGCCAAACGGAGG

CTCGCCAAGATCAAGAGAGAGTACTTCGAGCAGCACGGGGGCCTGACGCTGTTCGACGAG

ATGAGGTCGAGGCAGGGGCTGTCCTTCAAGCTCTTCACCCAGGAGGAGCTGGAGGAGGCG

ACGGGCAGGTTCGACGAGCGCAACGTGATCGGCAAGGGGGCCAACGGCACCGTGTACAAG

GGAACCACCAAGGACGGCGACCTGGTGGCCATCAAGAAGTGCAGGCTCGCCAGCGAGAGG

CAGCAGAAGGAGTTCGGCAAGGAGATGCTCATCGTGTCCCAGATCAACCACCGCTACATC

GTCAAGCTCTACGGTTGCTGCCTCGAGGTGGAGGTCCCCATGCTCGTCTACAAGTACATC

CCCAACGGCACACTCTACCGGCTCATCCACGGCCGGCGCGAGCGCGAGGGCCCGCG

>Traes_5DL_BB8B4C97E

CGCCCGCCCCTCCGCGCCGCCACCGGCTCATGGAGGGCCGCCATGTTCATCATCCTGATC

GAGTTCAGCGAGCGGCTGAGCTACTTCGGCATCGCCACGAGCCTCATGATCTACCTCACC

AAGGTGCTGCACCAGGACATGAAGGTCGCCGCCGTGAACTCCCAGTACTGGATGAGCGTC

ACCACCCTCATGCCGCTCCTCGGCGGCTTCCTCGCCGACGCCTACCTCGGCCGCTTCCGC

ACCGTGCTCCTCTCCACCGTCGTATACCTCCTCGGCCTCGTCCTGCTCGCGGTGGCGCAG

CTGGCGCCGGGACTGAGGCCTGGCGGTGTCTCTGTGCCGCGGGTTCACGAGACGCTCTTC

TTCGTCGGGATCTACCTCGTNCGCTCGAGAGCTTCGGCGCTGACCAGTTCGACGACGGCC

ACGCCGGCGAGCGGCTTCAGAAGATGTCCTACTTCAACTGGTGGAACTGCGCGCTCTGCT

CGGGGGTGCTGCTTGGGGTCACCGTCGTCGTCTACATCCAGGAGCGGGTCGGATGGGGCG

CCGCCACCGTGCTCCTCGCCGCCGTCATGGGCTGCTCCCTCGTCGTCTACCTCGCGGGGT

GGCGGACCTACCGGTACAGGGTGCNNNNNNNNNNNNNNNCTGCGGGTTGCTGTGGCTGCG

GTCATGAAGCGGCGCCTTCAGCTGCCAGCCGACGCCGGCGAGCTGTACGAGGAGAACGAC

GGCAAGAAGAGGCTGCTCTGCCACACCGACCAGCTCCGGTGTCTCGACAAGGCGGCAATC

TTTGAGCATGGTGGCGAGGTCTGGAGCGGGGCGTGGCGTCTGGCGACGGTGACGCAGGTG

GAGGAGACGAAGCTGGTGGTGTCGATGGTGCCCATCTGGGTGGCCACGCTCCCGTTCGGC

AT

>Traes_2DS_DCA6F067D

GGAGGGGTTCGAGACGGTCGACAAGGGTACCCCAAAGATTTACGGTCTGGCGCAGTGCAC

GCCGGACATGGCACCGGCCGATTGCCAGACCTGCCTCCAGGGTATCATCAAGAGGATGCC

GGAATTCTTCAGTGGGAAGCAGGGTGGCCGGGTTCTAGGATTGCGGTGCAATTACAGGTA

TGAGCAGTATCGCTTCTTCAATGGGCCTTCGCTGCTGCAGCTCCCGGCGCCATCCGTGGG

GTTAGCTCCAGCTCCAGCGCCGGCGAACGTGAAGCCACCGCCAGTCGGAGGAGGTGAGCC

TGCATAAATATTCG

>Traes_2DL_2613357A6

AGGACCGACTGCTCCGCCGCCGCACCCTTCCCGAGACACGGACTCCCGAGGCAGGCATCA

CCGTGGACGCGCGCGCGCTGGTCGAATCGGGGAGGGCTCGGGAAACCTTGGAGAAGGGCG

GCGGGGACGGTAGGTACTCGCCATGGCCGGGGCACAGGTGGTGGACTCCGGCGAGAAGCG

CCTCAACGAGCTCGGCTACAAGCAGGAGCTCCGGAGGGAGATGACGCTGTTCAAGACGCT

GGCCATCTCTTTCTCCACGATGACGCTCTTCACGGGGATCACGCCGCTGTACGGCAGCAG

CCTCCAGTACGCCGGGCCGGCCAGCCTCGTGTGGGGCTGGGTCGTCGTCTCATTCTTCAC

CTGGTTCGTCGGCATCGCCATGGCCGAGATCTGCTCCTCCTTCCCCGTAAGCACACCTAC

ACCTTCCCGGAACTTCCCTGCATGCCGCCGTTCCCAGATTCAAGGACTTTGGTGCTCTGT

TTATGCTCTGCTTGTAGAATTCAGTTGGGTTTGGTCTGTCAAAATTATACTATCGGCACA

AAATTCTTGCAATTTAGTTCTGACTCTCGAGTGGCATTGTGCCGCAATGGCCGAATCTTG

AATGTATTTTCCTGTAGCTGTGCCGTATGACTTGTGTTATATTCGGGAAAAAATCCTATG

CGACCGGGTCGCATACAGCTGCTCGTGAGACCTTCTCCCGCGTGCGACACCTGGCATTAC

GAATCCTAAAGCAGCAGCATCTCGTTCCCCACTTCCCCGTTTCCAAATCTCTCCAACGAG

TCAACGACCCAAGCTGCAGCCTGCTCGTCACATCGCCGCCGGTGTGCTCCTCGACGCCAG

GTACACCACCCCAGCACGGCGCGTTCCATCCCCGCCGGCAGTACTCACGGCACCGTCGTA

ACTGCTCTCCACATTTGGCCACGGGGATGCCCCTCGATGCCGGCCGGGCGTGACTCCTTG

CTGGTGGTGCTCCACTCCCGTCAGAAGTAGCCAACTGCTAGTCCCCGACGGCCACGACCA

TGCTCGTGGGCGCCTGCACTTGCGGCACCGCCGACTCAATAAGAATTTTCCCTGTATATT

GTTGGTGCTTGATTTGAGCTGTACAATACTGAACAAAGGCAGTTTTTTTCCCTGTATATT

GTTGGTGCTTGATTAGAG

>Traes_2DL_13533D388

GGGCTACCGCCACTTCGACACGGCCTCCATGTACGCGACGGAGCGGCCCCTGGGCGAGGC

CCTCGCGGAGGCGGTGCGCCGCGGGCTCCTGGCGTCCCGGGAGGAGGTGTTCGTCACGTC

CAAGCTCTGGTGCACGCAGTGCCACCCGCACCTCGTGCTCCCGTCCCTCCGGGAGAGCCT

CCAGAACCTGCAGATGGAGTACGTGGACCTGTACCTGATCCACTGGCCGATAAGCCTGAA

GCCCGGGCCGGCGG

>Traes_6AL_A18CC316B

TCCGCCTGCTTCCCGTATGGATCACCAGCGTCGTCGTGTCATCGGCATTCTCGCAGATGA

ACACCACGTTCGTGCAGCAGGGCAGTGCCATGGAAATGACC

>Traes_5AL_F725705B9

CGGGGACCTTGACATCGAGTCGATTTCTGCCGCCCATCTGCCCAAATCACACCCTAGCTT

CGACCCACAGCGATACCTCGAAATGTCTGAACAGTGGAAGACCTCACCTCTGCCGACTGA

ACCTGTCGAGCTGTTCATTGGCATCCTTTCTGCAGCCAACCATTTCGCCGAGCGGATGGC

CGTTCGCAAGTCATGGATGATTGCCACAAGGAGATCGTCCAACAGTG

>Traes_7DL_B4D442F3A

GAGGGCCTGTCGTACCGGTCGGCGCGGCCGGAGGTGTTCGGGGTGGTCGACCTCACCAAC

CTCCGCGCCGTGAGTGTGAATCAGTCGGAGACCACAGCTTGGGTCGACTCCGGTGCGACC

ATCGGCGAGTTGTACTACACCATCGCGAAGGACAACTCTCAGCTCGCGTTCCCTGCCGGC

CTGTGCCCGACCATCGGTGTCGGCGGCCA

>Traes_5DL_81A2647C1

CAGAACCCAACCACATCCACAGCCAATTCCGAGCTCTTCCGAGTCCCAGAAGCCACCTCC

CATCTCCTCCATCGTTTCGCAGTTCGCACAGACCTACGTAGTACCATCGAGCTCGTACCG

GACCAGGACCAGCTAGCCGCGCGCCGATGGTTTCGTCGATGAAGCACTGCAGAGATCAGG

CACCGGAGGTGCCTCTGTCCCTCTCGCTCTCCCTCGGCGCCATGGCCGACCGCTCCAAGA

AGCAGCGCCGCGGCGCAGACGGCGAGTTCGTCTGCAAGACATGCAGCCGCGCCTTCCCGT

CGTTCCAGGCGCTGGGCGGCCACCGGACCAGCCACCTGCGCGCCCGCCATGGG

>Traes_5DL_AAFCF3BFC

GGAAGCGGCGCCTTCGGCTGCCAGCCGACGTTGGCGACCTACACGAGGAGGACGGCGGCA

AGAAGAGGCTGCTCTGCCACACCGAGCAGCTCCGATGTCTCGACAAGGCGGCAATCGTGG

AGCATGATGGCGAAGGGCGGCGCGGGGCGTGGCGGCTGGCCACGTTGACGCAGGTGGAGG

AGACGAAGCTGGTGGTGTCGATGGTGCCCATCTGGGTGGCCACGCTCCCGTTCGG

>Traes_6BL_931C25F08

GCCGCCTCCGGTGGATCAACTACCTGCGCCCCGACATCAAGCGCGGCAACTTCACCAGCG

AGGAGGAAGACGCCATCATCCAACTCCACGCCATGCTGGGCAACAGATGGTCCACCATTG

CCGCCAGGCTGCCTGGCAGGACGGACAATGAGATCAAGAACGTCTGGCACACACACCTCA

AGAAGCGACTCGCGTCCTCGTCCAAGACGTCCGGCCAGGCAGCGCCTAAGCACAAAGCCA

AGAAGCCTCCT

>Traes_2DL_B978B4E22

AACCCTGTGACCAACCATGTCCAGAGTGCGGAGCAGCACAACCAAGATGTCAACTCTCTT

GGTCTCATCTCCTCAAGGAAGACCGCCGAGGCCATTGACATACTCAAGCTCATGTCCTCA

ACATTCTTGGTCGCGCTGTGCCAGGCAATCGACCTCCGCCACCTTGAGGAGAATGTCAAG

AATGCTGTCAAGAGTTGTGTGAAGACTGTGGCTAGGAAGACACTGAGCACCGATACCAAT

GGCAATCTCCACAACGCACGCTTCTGCGAGAAGGACCTTCTGCTCACAATCGACCGTGAG

GCAGTGTTTGCGTACGCAGACGATCCTTGTAGCGCCAATTATCCACTCATGCAGAAGATG

CGTGCAGTTCTTGTGGAGCATGCCTTGGCAAATGGTGAGGCTGAGCGCGACGTGGAGACA

TCTGTGTTTGCTAAGCTTGCCACGTTCGAGCAGGAGCTTCGGGCAGTGCTGCCAAAGGAG

GTTGAGGCTGCCAGGAGCGCCGTGGAGAATGGCACTGCCGCACAGCAAAACCGTATTGCC

GAATGCCGGTCGTACCCGCTCTACCGATTCGTGCGCAAGGAGCTTGGAACGGAGTACTTG

ACTGGAGAGAAGACAAGGTCTCCTGGCGAAGAGGTGGACAAGGTGTTCATTGCCATGAAT

CAGGGCAAGCACATCGATGCACTGCTTGAGTGCCTCAAGGAGTGGAACGGCGAGCCCCTC

CCTATCTGCTAAATAGAGGATCGAGAAAGTGAAGAGTAGTGTGCTTCAGATTTCTGAAGG

CTCTTATGATAATACTGTTTTTTCATTATATATTCTAAAAGTTGATGTTTACAATGTTCT

TCTAGAGCTGCCAATGTATTGCCAAAGATTGCAATTGCATGACTTGGTAGTGTTGGGTAG

CCAGTAGAACTTTTATGATGTACGTAAGTTAAAAGGGCAGCGTGTGTGTTAACTTTTCAT

GATAAATTTACTGCCTCCATTTTTTGGGATCAACTGGTCCTCGTGACGATAGTCCAAGAA

CCAACGATTGGAATTTCTTATGGTGTGTGTACGGCGATTCAGAAAGTAAAACTCATGCCT

AGGTGGTCACCGGTCAGTAATAAGTTCTTTGAATTCTCTGAACGGGATGAGGGCTTCTCT

GAATTCCCAATCTCCCATCTAGGTTTTGTTGTTTCAGTATTCTACTTACTAATTTTCAGT

CTGGATTTCTATGGAGGGACACAACGATGTTTAACTTGGCGTTCTTCAACTATTGTAC

>Traes_2DL_48CCFF042

CTCATGCCCCTGTTCGAGGCCTACACCGCCAGCGCGCGCGCCCCGCTCGCGGCTCTTCTC

GAGGACCTCTCCGGTTCCTGCCGCCGCGTGGTCGTAGTGCACGACCGCATCAACGCCTTC

GCCGCCGAGGAGGCCGCGCGGCTGCCCAACGGCGAGGCGTTCGGGCTGAACTGCGTGGCC

GTGTCGATGCTCGTCGGAAGAATCGACGCCAACCACCGGCTACTGCGTGAGAACGGCGTC

GTCCTCAGCTCCGTCGAGCGCTATGCGACCAAGGAGTTCATAGAGTACGCCAGCCGGGCC

AGACCGGCGAAACAGATCTCGACCGGCGCGGGCATCCTGGCAAACACATGCCGCGCGCTG

GAGGGTGATTTCATCGACGTCGTCGCCGGGCACCTGGCCGCCGACGGCAAGAAGCTCTTC

GCCATCGGACCGCTAAANCCACTGCTCGACGACAGCGCGTCGAAGCAGAGCAAGCAGCGG

CACGAGTGCCTGAACTGGCTCGACAAGCAGCCTCCGGCGTCGGTGCTCTACGTGTCCTTC

GGCACGACGTCGTCTCTGCGAGCGGAGCAAATCGAGGAGCTTGCAGCAGCACTGCGCGGC

AGCAGACAGAGGTTCATATGGGTGCTGCGCGACGCCGACCGCGGCGACATATTCGTGGAG

GCCGGCGAGAGCCGCCACGAGAAGTTTCTATCAGAGTTCACCAAGCACACCGAAGGGACG

GGGCTGGTGATCACCGGG

>Traes_4BL_CA03BD203

CAGATTGTAATCAATAGTAATTCAAGAGAGTCGTGCAGAAAAAGTCTGGATGATGCGATC

TCCATGGTTAACAAACTTCGGTCACTGTCTACAGACTCCCAATCTAGAGTTCCCCTCGCT

TTGATCATTGACGGAAACAGTCTCGTCTACATTTTTGACACAGACCGTGAGGAGAAGCTT

TTTGAAGTCGCGATAGCATGCGATGTTGTTCTATGTTGTCGAGTGGCTCCTCTACAGAAG

GCTGGGATTGTTGATTTAATAAAGAAGCGAACAAGTGACATGACTCTTGCTATTGGAGAT

GGTGCAAATGATGTATCCATGATTCAAATGGCCGATGTTGGCATTGGCATCAGTGGTCAA

GAAGGAAGGCAAGCTGTGATGGCCTCAGATTTTGCCATGGGGCAATTTAGATTTTTGGTC

CCTCTATTGTTAGTTCATGGCCACTGGAACTACCAGAGGATGAGCTACATGATCCTATAC

AACTTTTACAGAAATGCTACTTTTGTCTTTGTGCTTTTCTGGTATGTACTTTACACTGGT

TATACCCTGTCAACAGCAATAAATGAGTGGAGCAGTGTGTTATACTCTGTGGTCTATACC

TCTGCGCCGACTGTCATTGTCGCCATTCTCGACAAGGATCTGAGCCGAAGGACATTGCTG

AAATACCCCCAACTCTACGGTGCGGGGCAGCGCGAGGAGAGTTACAACCTAAGACTATTC

ATTTTCATCATGGTGGACTCTGTTTGGCAGAGCGTCGCAGTTTTCTTCATCCCTTACCTC

GCATACAAAAACAGCGCAATCGACAGCGCCAGCCTCGGAGACCTGTGGACACTGTGTGTT

GTCATTCTTGTCAACATTCACCTTGCCATGGATGTCATCAGATGGACTTGGATCACTCAT

GCAGCAATATGGGGCAGTATTGTGGCGACATGGATTTGCGTCATCATCATAGACTCCATA

CCCACCTTGCCCGGTTTCTGGGCAATCTATGAGGTGATGGGAACTGCATTGTTCTGGGCA

TTGCTTCTTGCGGTGATTGTGGTTGGAATGATCCCTCATTTTGCTGCAAAGGCCATCAGG

GAACATTTCATGCCCAATGACATCCAGATTGCGAGAGAGATGGAGAAGTCGCGAGATTCT

CGTGATGCTAATCATCCAGAAGTCCAGATGAGTACATCCACTCGAGCTTAGGAGTAGGGG

ATCTTTGTTCATTCGCGTAGGCTAGAGTTACACCTTTCTCTTTCTGAAGAAATTACACCT

TTTCCTTCTTTTTGTTGTGTGTATATAAAGCAATTCTTTCGGCAGTGAGCATGCTTCTTG

TTGCTTGCTACCCTGTCAACTATCCAAAGGATAGATTTCTTGTAGCCATGCTGTGCATCC

GCCTGCATTTTTTTTTTGTTAATTTATATGAAAAGAATCGTACAGGAGTTAACTGTACAT

ATTCAGGGACTGATTTATTTTATGAAGATTCTGAAACTGATGAT

>Traes_2DL_D1D2DDDA1

TGCAAAGTGTCGTTGTTGCGCCAATGCCCCATAATTTTCAGTATATCTGCAAATTGATCA

TTTCACAGTGCCCCAAGTTGTTGAACATCACATGGGTCCGAAGACTTCGTCTTCTTGAGA

GGCTTTTCATATCTCATTGTGATGGGATGCTCGAATTTTTTGAAGATCGGGAAGACGAGG

AGCAGTGTGGAGAACAACTGAAAGTGCAGGGTCATGCTTCAGATAAACAAGAAGATCATG

CTATTGTAGAAACTTCACGGAATGACACAGGGCAGAATGACTTCCCAAAGTTGAGATTGA

TTGCATTGACGCAACTCAAGAAGCTGAGTAGTATTTGTAAACCAAGAGAATTTCCATGCC

TTGAGACCCTTCGGGTGGAGGAGTGCCCAAATCTGAGAAGCATCCCGCTGAGCTGCACGC

GTAACTATGGGAAACTGAAGCAGATATGTGGCTCATTTGAATGGTGGGGGAAACTGCAGT

GGGAAAATTGGGAGGAGGCGGCATATGTGGAGAGAACCTACTTCATTCCAATCTGACAGA

GCCCCAATTTCAATTGCTTTTGTATGGCATGCAGGCGGCTTAGTTCTGTCTTCAGTCTTC

ACACAAATAAGAGGTCTCCTTTTAAATTGTTTAAAATTTGTATATGTGACGGCGTAGCTT

CTATACATTCTTAGAACTACATTCGTGTACATGGTATACTCTGCAATTTGCTGATATTGC

ATTCATGTAATACGATTTGTTTATTTAAATCACAGTCAATAAGGGTCTGTGACCTTGTG

>Traes_5DL_7AC5FB2A8

ATGGAGGTGAAGGTGCTGAGCTCCAAGATCGTCAAGCCGCAGTACGCCGAGGGCACGGCG

CGGCCGGACACCACGGAGCACGTGCCGTCCTCGGTGTTCGACAAGGTCACCTACCACATC

CAGATGGCCATCATCTACGCCTTCCAAGCGCCAGCGCCCTCCACCGAGGACATAGAGCGC

GGCCTCGCCCAAGTGCTGGCCGTGTACCGCCTCTTCGCCGGCCAGGTCCGAGCTGGCCCG

GACGGCGCGCCTGGGGTGCTGCTCAACGACCACGGCGCGCGGCTCGTCGAGGCGCGCGTG

GACGGGGCCACACTGGTCGAGTTCGCGCCTCCGAAGCCGTCGCCTGTCGTGTTGCAGCTG

CACACGGACCTGGAGGGCGACGTGGAGGAGGTGGTGCAGGTGCAGCTCACGCGGTTCGCG

TGCGGCTCGCTGGCCGTCGGTTTCACGGCCAACCATGCCGTTGCTGACGGCCACGCCACC

AGCGATTTCCTCGTCGCGTGGGGCCGCGCCGCGCGAGGGCTGGACATCTCCGCCCCGTCG

CCGCCGCCCCACAACCACCTTGACCTCTTCCGGCCGCGCG

>Traes_7BL_43EC8F84D

CCACGACTACGAGGGCCTGTCGTACCGGTCCGAGAAACCCGAGACGTTCGCCGTCGTCGA

CCTCAACAAGATGCGGGCAGTGTTGATCGACGGCTACGCCCGCACGGCGTGGGTCGAATC

CGGCGCGCAGCTCGGCGAGCTCTACTACGCCATCGCGAAAAACAGCCCCGTGCTCGCGTT

CCCGGCCGGCGTCTGCCCGACCATCGGCGTCGGCG

>Traes_3DL_E0CD3F643

GGGGGTCAGTCTCCATGGCCTGGCAGCAGGGGTGGCACGCGGTGGTGCGCCCCGAGCGCG

CCTTGCAGTGTACAAGGTCGCATTTGAGGGCCCGAAGAAGGTGCAGCTTGCTAGTGCCGC

GCTGCTAGCAGCTCTGGATGATGCAATTCATGATGGAGTAGACATCCTATCCCTATCAGT

CCAGTATAATGACAATTCATTTGGTTCACTCCATGCAGTCCAGAAGGGTATTACCGTTGT

TTATGGCGCCGGAAATAGTGGGCCTAGACCACAAGTCATTTCTAACACAGCCCCTTGGGT

CATTACAGTCGCAACGAGCAAGATCGATCGGTCTTTTCCAACTGCCATTACCCTTGGAAA

CAACCAAACCATAGTGGGTCAATCACTCTATTACATGATAACAAATGAATCCAAGAGTGG

GTTCCAGCCACTTGTACAAGGTGGAAGCTGCTCAATCGAGGCGCTAAATGGCACGGAAAT

CAACGGAAAAATTGTTCTATGCATTAAAGAAACTTTTGGCCCAACAGCAGACATTCTCCC

AGACGCCATAACAAATGTTAAAAGTGGTGGGGCATTTGGTCTTATTTTCGCAATATATAC

CATAGATAAGCTTTTGAGCACCGAAGATTGTGTGGGCATGGCATGTGTCATTGTCGACAT

TGATATTGGGTTTCAAGTTGCAACATACATTGGAAGCCAAGGCTCGCCCATTGCAAAGAT

TGCGCCAGCAAGTACTATAACAGGAAACCGAGTTCCTGCTCCAAGAGTCGCATTTTTTTC

TTCAAGAGGCCCGTCCGCCAAGTACCCTACAGTTCTTAAGCCTGACATAGCCGCACCAGG

AGTGAACATCTTAGCAGCTACAGGAGACGGATATGTGTTCGACTCAGGGGCATCAATGTC

AACCCCACATGTAGCAGGCGTCGTAGCATTGCTAAAGGCTGTACATCCTGATTGGTCTCA

TGCCGCCCTAAAATCAGCAATTGTTACCACTGCATCAACCAAGGATGAGCATGGCATGCC

GATGCTAGCAGAAGCACTACCCCGGAAGGTCGCCGACCCATTCGACTATGGAGGG

>Traes_5AL_857A0C6DE

CTGGTGAGTGCCAACAGAGCCTCATTGTCGACCACGAATGTGCAAGGTCGATCAAATGCT

TGGTGATTAGCCCACGATGCCAACTGACGACGGAATATTTTGCATGTTGTTCCAGGCTGA

AGTTTTCTTTCTCGGGCAACTGAGGCACAAGAACCTGGTGAAACTCATCGGCTACTGCTA

CGAGAACGAACACCGGATGCTGGTGTACGAGTTCATGAGCGCCGGGAGCTTGGAGAAGCA

CCTCTTCAAAGGTGAGTTGCTGAGGCCAACAAGTTCAACAAATTTGTTAAATTAATTAAA

AGTACATGAGCGATGATCTGCTTCTGTTCCCTGCCTTTATTCTCTGTTTCCTTTTTGTCA

AGTCAAGGGGATAAGAGAGCAGTTATGCGCCTTGCAAACGAACATATCTCTTTTAAAATT

AGATTAGTACTGGATTGTTGGTGCAAACTTATTGGTCTTGGTCTGTTGATCAACGTGCAG

CATGTAGCTGACCTGAAATATCCTACTGTCTTCAGGTAGCATCAATNNNNNNNNNNNNNN

NNNNNNNNNNNNNNNNNNNNCCCGCCGGTGATCTACCGCGATTTCAAAGCCTCCAACATC

TTGCTCGACTCGGACTACAACACTAAATTGTCAGACTTTGGGCTGGCCAAGGATGGACCT

CAAGGCGACGCAACACACGTGACAACACGTGTAATGGGGACACACGGGTACGCAGCGCCT

GAGTACATCATGACGGGCCACTTAACCGCCAAAAGCGATGTCTATAGCTTCGGTGTGGTG

CTTCTGGAGCTCCTCTCGGGACGACAATCCGTGGACCGTGCACGACGACCAAGAGAGCAA

AACCTAGTGGATTGGGCTAGACCGTACCTCAAACGATCAGACAAATTGTACCAGGTGATG

GACTCGACCCTCGACTGCCAATACTCGTGCAAGGGTGCTGAGGTGGCTGCATTGGTGGCG

TACAAGTGTCTGAGCCAAAACCCCAAGTCAAGACCATCCATGAGGGAGGTCGTCAAGGCA

TTGGAGCCCGTAGTAGGCATGGAAGACTTCTTTCCAGTGGGACCGTTTGTGTTCACCATT

GTCGTTGAAGAGGACAAGGTGATGGACATGAAGGTGGAGGTTGAGGAAAAGCACCAACAC

CATCACCAGAACCATCAGGACAGACATCGACAAAAATACCCCGAGTCGGCAATCCATGGT

GGCATTGTGCTCCATGGTGACCACGGGCATGTTGCCGGGTTCACCGGTACGTTGCGGCGG

CAACAGAGGACGTTGAGTTACCACCGGGAAAGAGGGGCTTAGTGGTTCAAGAGTATAATA

GGGGAGGCAGTTAGGATTGTAGAGATAGAAGGTATTTATATGTACATACTATTGAGATGT

GATACGGTGTCACATCGTTTGTTTAGAACTGTGGACGTGTGTCTTTCTTT

>Traes_6BL_A5F84AD98

GTGGTTGTGCGCGCGCCCGCAGAGCGTGTGGCTGGACAGGGAGACCGGCGCCAAGTGCTA

CATGTTGTCCGCCAGGAACCTCTTCATCGTGTGGGGCGACACGCCGCAGTACTGGACCTG

GATCCCACTCCAAGACTCCAGGTTCTCCGAAGGTGCTGAACTCGTGAATGTTTGCTGGTT

CGAGATCCATGGGAAGATACATGGCAAGATGCTCTCCCAAGGCACAACCTATGCAGCCTA

CATGGTCTTCAAGATGGACGAGAATTCCTATGGGCTGAATTTTCCTGCCCAGGAGGCATC

>Traes_3AL_8804EA96B

ATGAGCGGCTACGACGAGGGGATAGTTAAAGCGGATGAGGTAGAGGGGAAGGTGAGACTG

ATCATGGAGTCCGAGCAAGGGGAGGAGATCAGGGAACGGATGACGATGGCACAAGTGATT

GCTGCCAATGCGCTGGAAAGCGGTGGATCTTCGGCGGCAGCATTTGTTGACTTCGTGGAT

AATCTGAAGATTTCGATATTCGATTGAATCGTTGACTCTGTAGTGGAATTTTCAAACTGA

GCAACACCACTTAATCAGTATGGACAAAATGAAGTGGCAATCAGCTAATGGTGTCCCTTT

TTTTTAAACACACAAACTATGTGTCTTTTGTACTGCCTGCATCCAAAAAAGATTGTCTTA

AATTTATCTAAATACGGATGCATCTAACACTACAAGCTATTTTGGGTGGAGGTAATGTGA

AGATACAGGAGAAAACAAGTTGCAAGGGGGTGCGACGGTTTCTTGGTCTGGTCCAG

>Traes_3DL_8BAF41028

GACCTGAGCCCTGGCACAGAAGCCGAGGAGCTCGCCGGAGCCGACCACATGGCCATGTGC

TCAAAGCCCAGGGAACTCTGCGATGTTCTTCTCAGGATTGCCGACAAGTATGACTGAGAA

TGGTTGTGCACATCCAGCCGTACAACAAGCATGCCAGACCAAATTCCAAAGGTCCGTTAG

AAAAAAGAATTCCAAAATCTAGTACACACTTCGATCCGAATTAATGGAGACGATCGGAGG

GAGTAAAGAAATTCTTTTCGACTCATGTACAAATGCCAGAGTGAAGCATTAGAATAAGTA

CCTTCAGGGAGTTCACGCTTAAACTGTTTTTTTACGGTTAAAGCAAAGCTTTATTATGAA

GACATGTTGGGCATTTCGCCCATTACGATGACAGCAGCCTCGGCTGGTACATCATCCTCC

CATAGGAGGGCTTCATCAACATTACATGCATAGCCAAG

>Traes_6DL_67FD04DA0

GGCACAGCAACTGGCGGGCGCTGCCGAAGCAGGCCGGCCTGCTGCGCTGCGGCAAGAGCT

GCCGCCTCCGATGGATCAACTACCTGCGCCCCGACATCAAGCGCGGCAACTTCACCAGCG

AGGAGGAAGAAGCCATCATCCAACTCCACGCCATGCTCGGCAACAGATGGTCCACCATTG

CCGCCAGGCTGCCTGGGAGGACGGACAACGAGA

>Traes_3AL_A0C2D775D

GACCAGGAGCACGAGTGCCTCCGGTGGCTGGACGCGCAGCCGGACCAGAGCGTAGTGTTC

CTCTCCTTCGGCAGCATGGGCACGTTCTCCGTTCAGCAGCTACAGGAGATTGCAAATGGA

TTGGATAAGTCAGGGGAGAGATTCCTGTGGGTCGTGCGGAGCCCGCGCAATCCCGACTAC

AAGTACGGCGATTCGCTGCCGGAGCCCGATCTCGACGCGCTCATGCCGGAAGGGTTCTTG

GAGAGGACCAAGGACAGAGGACTCGTGATCAAGTCTTGGGCGCCGCAGGTGGAGGTCCTG

CGCCACAGGGCGACCGGCGCGTTCATGACGCACTGCGGCTG

>Traes_2DL_3816B2E1D

CCATTGGACCGTTAAACCCACTGCTTGACGCCAGCGCACCGAAGGAGAGCAAGCAGCGGC

ACGAGTGCCTGAACTGGCTGGACGAGCAGCCTCCGGCGTCGGTGCTGTATGTGTCCTTCG

GCACGACGTCGTCCCTGCGAGCCGAGCAAATCGAAGAGCTCGCAGCAGCACTGCGCGGCA

GCAACCAACGCTTCATCTGGGTGCTGCGCGACGCGGACCGCGGCG

>Traes_4BL_7AF7D563F

GACTACCAAGGCGAGTCGCCGGACGAGCAGGCGTTGGTTTCTGCCGCCGCGGCGTATGGC

TTTGTCCTGGTTGAGCGAAGCTCCGGACACATTGTCATTGACGTCCTTGGTCAGAAGCAG

AGGTTTGACGTCCTTGGTCTTCATGAGTTTGACAGCGACCGCAAGAGGATGTCAGTTATA

ATTGGCTGCCCGGACAAGACTATCAAGCTGTTTGTAAAAGGTGCAGATAGTTCCATGTTT

GGAATCATCGACAAAACACTGAATCCAGATGTTGTCCAGGCAACTGAGAAACATCTCCAT

TCATATTCGTCAGTCGGCCTGCGAACACTCGTCATCGGTGTCCGGGAACT

>Traes_7DL_D33A6010D

GTTTTGATATCTTTTGTTTCACTTTTCAGTGGTTACAAAGCTCCGGAGTATGCATCTCGA

TTCTCAAGGAGTTTACTCAATGAAGACAGATGTGTTCAGCTTTGGCGTTTTGGTTCTGGT

GATTATTAGTGGCCGAAAGAATACCATACTCGACAAGCGAGGGGATACTGTTGGTGATCT

TGTACGAGATGCCTGGCATATGTGGAAGGACCAAAGGTTGCATGATCAGACTTCAACTTG

ACAAGCAGCAACTACAGAGGTCAGAGTTAAGATCTGTAACTGTACACACTAGAAACACAT

CAGGATCTCCAAATATGCACCTGAAGATCTCCATGCTCAGGTAACGTACAATATAAGAAC

TCTGAACTGAAGGTGGTCTTCTTTGTGTGTGTGTGTGTGTGCCATAGAACTCTGAACTCT

GAACTGAAGCAGAGAGACGTAAGCAACGACACATAGTAATCAG

>Traes_5BL_99BAA4CB8

GCCAATTTGAATATCTTGGTCAAACATTTCGTGGAGTAAACTGACATTTGCCCTTTGGCA

CCCGTTGAAATTCCATCGTTTAGTTATCTGATAGGGATCTTCATTTTGATTTGTGCCAGG

TCCAATGGCCTTTGCAGAGAAGATGGTTCTTGCCGTCTTTGGGGGCCTGATCGTCCTGTG

GATGACGAGGAGCCTGACGGACGACATCCCTGGGTGGTCGGTTCTCTTCCACGGCAATGT

CGGGGATGGAACAGTCACCATCATGATGGCGACGCTGCTCTTCATAATCCCGAGCGGCAA

GAGCGACGGCGAGAAGCTCATGGACTGGGGCAAGTGCCGGAGACTGCAGTGGCACATCGT

CCTCCTCCTCGGCGCCGGCTTCGCCATCGCCGACGGGTTCAAGGCGAGCGGCCTGACGGA

CATCCTCGCCGGGTGGCTGGGCTTCCTGCGGGGCGCGCCGGCGCTGGCCGTCGCGCCCGT

GGCGTGCGCCTTCAGCGGCCTCCTCACGGAGTTCACCTCCGACGATGCCACCACCACGCT

GGTGCTGCCGCTGCTGGCGGAGCTGGGCAGGAGCATCGGCGTGCACCCGCTGCTGCTCAT

GGTGCCCGGCGCCGTCGGCGCGCAGCTCTCCTACCTGCTGCCCACCGGGTCGCCCGGCAA

CTCCGTCGGGTTCAGCACCGGCTACGTCACCATCAAGGACATGGTGGTCACCGGCATGCC

CATCAAAATCGTCGGCGTCACGGCTCTCACCGTCCTGCTGCCAACGCTAGGTGTTGCTGT

TTTTGGTATGGATCAGAAGGTATAGGAACCTTAATATTTGTTGAACATTTCATCAATCTG

TAGAGTGTAGAGTGTAGGTTTATAGTATGTCTAGTAGTAGGATGTTGAGGCTGTACAGTG

TATAGTCAGTGTCATTGCCTCACGAACGATATGGGGTCATATTCTTTTCTTTATGTACGA

GACGGCATGTAAAATTTGTATCGAACGGTTCCCATATGAATATACCGAAATTTGCA

>Traes_2DS_7BDBA04EF

CTCAGATACAAGTACCAGATAACTCCAAACTCAACCAGCGGCTGCGGTGGCGCTGACAAA

TGGGCTGATTTTGGGAGCACCGAAGAAATCTTCTGTCCAGCTGGTTACCACTGCCCAAGC

ACAACATCCAAATTGTCCTGTAGCAGTGGGCATTATTGCAAATTGGGTTCCACCAGAGAA

GAGAAATGCGTCATAAAAGGCTCATGTAAAGAGAACGAAGAGAATGAAAACATCATCATT

TTGGGCGCTTGTTTAGTGGGTATAATAGGCGTGTCGCTCTTGGTCGTGTACAACTGCTCA

GGTCAGTTCCTCACTATCCGTGAACGGAGGAAAGCGAGGTCGAGGGAGAACGCGATTCAG

CTTGCTCGGCAGCAGCTCAGGGCTCACCAGGGATGGAAGGCGGCCAAGCAGATCGCGAGG

AAACATGTAACCGGCGTGCAGGACCATCTGT

>Traes_1DL_1189E4CF3

CTTCACCGGAGGAGAAGACAACGAGGACAAGGTGACGTTATCCGGGCTGCTCAACTTCGT

TGACGGCCTGTGGTCGACGAGCGGGGAGGAGAGGATCATCGTCTTCACCACAAACTACAA

GGAGCGGCTCNTGGACATGCACGTCCACATGGGATATTGCACCTCGGAGTCCTTCAGGAT

CCTGGCCAGGAACTACCACTTCGTCGAAGATGACCACACCATGTATCTGGAGATCGAGAA

GCTGATGGAGGAGGTGCCGATCACACCGGCAGAGGTTGCCGAGGTTCTGATGAGGAACGA

CGGAGCCGACGCTGCACTTAGTGATCTCGTCGGGTTCCTCGAGGCAAAGAGGGGAGAAGT

CGGTGCCAACAAGGGCGTAAAACATCATGGAAACAACAAGGTGGATAAATATGAGCAGAC

AATGGTGTTGTACTGCACTCCGGAGTACTTTAGGGGCGTGTCCCGTGACTACCAATCACT

TAAAGACCGTGCCATGATTCCCGAGGTCGAGCAGTTGTTGAGTGAGGTGCCGACCACGAT

CGAAGAGGTCACCGATGTTGTAGGGAGGAATAACGGCGGGGCGGACGCCGCAATCCGAGA

TCTCATCGGGTTCCTCAAGGCAAAGAGGGGTGACGCCGGTGAGAACAATGGTGCGAACCA

AGATGGAAACCACAATGGTGATAATAAATAGGAGAAG

>Traes_3AS_693536752

CCCGAGGCGCACGTCTTCAAGGCCGACCTCCCCGGCGTGAAGAAGGAGGAGGTCAAGGTG

GAGGTGGAGGACGGCAACGTGCTCGTCGTCAGCGGCGAGCGCACAAAGGAGAAGGAGGAC

AAGAACGACAAGTGGCACCGCGTGGAGCGCAGCAGCGGCAAGTTCGTCAGGCGCTTCCGC

CTCCCCGAGGACGCCAAGGTGGAGGAGGTGAAGGCCGGGCTGGAGAACGGCGTGCTCACC

GTCACCGTGCCCAAGGCCGAGGTCAAGAAGCCTGAGGTGAAGGCCATCCAGATCTCCGGC

TGAGTATTCGGGTCTGGTTCATGTGTGTGCGAGTTCTGCAACCTAAAAGTATGCTACAGT

CGTGTCTTTGTTCTTGCCGAGTCTGACGAGCCATCTCTGTATTGCGTTTCTTTTCCCTGT

CGTGTCTGTACTCTGTACTTGTGTAATCGCCAACAGATTTGGCGATGAATAAATGAGAAG

TAAATTGTTCTGATTTTGAGTGCAGTGCAGAATTTGCATACACAAAAATTGAATATTGTT

TTTGCTTGTGTTTCGGATGGACACGGAAAACTTGGCCACTGACAGGCTTTGTTCTACACA

AACATCTGCTTAAAGAATGATCGATACACTGAATGGTCTTCTGTCTCACATTCAGCTTTC

ATTAAGCGTGGGTCTACACTGATCAATACCCTGAATGCCCTGAATGTTGATTGTGTGACT

TCTATCTCACCTTCAGTTTTCTTGGTTCTTTCTTGAGAAATACCTTCAGTTTTCTTTGAT

CCTTTTCCTTTGAAAAGTACATTTTTTTTTGTACACACACCCATATCATCACACATATAT

TAACTCAAATGAAG

>Traes_3AL_994D1987E

TCCCCATCCGGTGGCTGTCCTGGTGCGGCAAGCTCGGCGCGTTCTGGAACACGGCGGGCG

CGTTCACGCTGGTGATCCTCATCCCGGCGGTGGCCAAGGAGAGGGCCAGCGCCAAGTTCA

TCTTCACGCACTTCAACGACGACAACGGCATGGGCATCCATGGCAAGGCCTACATTCTCG

CCCTCGGCCTCCT

>Traes_2DL_E72DCBDC9

GACTGGGTCATGAACAGCATGATGAACGGCACCGACAGCTACGGTGTCACCACCGGCTTC

GGCGCCACTTCTCACCGGAGGACCAAGGAGGGCGGCGCTCTCCAGAGGGAGCTCATTAGA

TTCCTTAACGCTGGAGCCTTCGGCAACGGCAACGACGGCCACGTTCTGCCTGCTGCGGCG

ACGAGGGCAGCCATGCTCGTGCGTGTCAACACCCTGCTTCAGGGATATTCTGGCATCCGC

TTCGAGATCCTCGAGACGATCGCCACGCTTCTCAACGC

>Traes_5DL_46BA5560E

GAGACGAAGCTGGTGGTGTCGATGGTGCCCATCTGGGTGGCCACGCTCCCGTTCGGCATC

ACGACGGCGCAGGTGTCCACCTTCTTCGTCAAGCAGGGCAGCGTGATGGACCGCCGCATG

GGCCCCCACTTCGTGCTCCCACCGGCGTCCATCTTCGCGCTGGCCGCCGTCGCCATGATC

GCCACCGTGGCGCTCTACGACAAGGTGCTCGAGCCGTGCCTGCGCCGCGTGACTGGGACG

GAGCGGGGGCTCAGCGTCCTTCGGCGCATCGGCGTGGGCATGGCGCTCGCCGTGGTGGCG

ATAGCCGTGGCCGCGGTCGTCGAGCGTCGCCGCCTGCACTCCACCGCCACCATGTCCGTG

TTCTGGTTGGTGCCGCAGTTCGCGCTGATGGGCGTGGCCGACGGGTTCGCGCTGGTGGGC

CTGCAGGAGTACTTCTACGACCAGGTGCCGGACTCCATGCGCAGCCTGGGCATCGGGCTG

TACCTGAGCGTGATCGGCGCCGGGAGCTTCCTGAGCGGCCTGGTGATCGCGGCGGCGGAC

CACGTGAGCTCGCACGGCGGGCGGCGGGCCGGGTGGTTCGGCAAGGACCTGAGCCGGAGC

AGGCTGGACCTCTTCTACTGGCTGCTCGCCGCCATCTCCGCCGTCAACCTGGGCTTCTAC

GTGCTCGTCGCCGCCCGCTACTCGTACAAGCAGACCGTCAAGGCCAAGAGGGTGAGCGCC

AGCGACGTCGAGTGCGCCACCGCCGTCGCCGCATAACCAAGTAGTAGTACTAGGCAAGGA

AGAATGATCGATGCCAATGGCCTTGCATCATCGTTCGTTGGCTTAAATCTAGTACTAGCA

TTAAGGGATTATTAGGATTTGTGTTCTGCTTTGTGATCGATCGTTGTGTTGCGTATTCAT

TCAAGTCGATGTCTGGTCTGGTGTAGCATGTTGTGGACTCTTGCAACAATGCAGGGGCTT

CTATTGTAGTCAGTGTTGATCATCATCTCATGCTTTAAACGTGAGCACAAGAGCATAAAC

CCCTGTGAATGTG

>Traes_7AS_5845260EC

GTACAACCGCCGCATCGAGGTGCCCGCTGCTGACGCGTACAAGATGGACAAGATCAATGC

CGAGATGAAGAATGGCGTGCTCTGGGTCACCCTGCTCAAGGTCAAGGAGGAGGACCGCAC

GGACGTCTTCCACGTCAAGGTCGAGTAGTGCTCATGGAGGATGGCCGGCGCGAGAGTGTG

AGAGACGCCTTGTTGATGTGGTCATGTTTGGTG

>Traes_6BL_F515D039E

TCGGAGTCGGAGGAGTTCCAGATTGACGACAGCTTCTGGTCGGAGACACTGGCGATGTCG

GTGGACAGCTCCGGTTCCGGGATGGAAACCGGCGACACCTTCGGCGCAGATAGTGTATCG

CCGTCGTCGAGAAACAATGAGATGGACTTCTGGGTCACACTGTTCATGCAGGCTGGTGAT

ATGCAGAGTTTGTCACAGATTTAATGAGAAGATTGGTTATGTTCATATTTCTCGCTAGCA

TTCGCGGTAGAACAGGCGAGCGGCCTGGCCGAACGTGGACAAACGAAGGGGCCATTTGGC

CTCGAAGTTCGTCGGCTGAGTTTTTTTTTCTTTGAGGGAAAGTTCGTCGGCTGAGTTGAC

CCTTGTTGCTGGCTCAGTTTCACGTGCGCATTGGCTGGCAACAACGAGAGAATTTTCTCC

AGGTACCCGAGGGACAAATGATGGGGCCGAAAGGTTGACTTGATCAAGGACTGAAAGCCT

GATAACACCTTTTAGCCTGATGAGTCTGTTTTACTCAGTAACCTGCTAAATCCTTTGTTC

GTTGTTCCTTGAGTTGCAATAGCACATCATTTTTTGTGTGGATAAAACACCAACAATTCT

AATGGTAAATTAGAATAATGAC

>Traes_2BS_A6CF5D121

CGCTCGCTCACTGGGCTCCATACACGTCGATGGTCAGCGCCATCGCAAATCCAAACCCAG

ACAGGATTACCCCGTCTATAAATCCACGCGCCCCGAAGGCCGTAGATCACATCACACAGC

ACACAGCACGAAGAAGAATTCCAGTCAGCTGAACTGAACAAGTAGCTCCTCCTAGACCTC

CCTCATCACCGACCGATCGCCATGGCGCAGGCGGAGCGCGAGTGCATGCGCGTGGTCATG

TTCCCGTGGCTGGCGCACGGCCACATCAACCCGTACCTCGAGCTGGCCAAGCGCCTCGTC

GCC

>Traes_3AL_33B515FA4

CTCGACCGTTCGTTCGTTCGTCGTATGTGGCGCTGAGCTGAGCTCACGCTCTCACGCGGC

CATGCATTGCGCTGGCCCTACCAATTTGGATTGAATTCGACGAGCCCCCTATATAGTCCC

GGTGGATCGTTCGTAGAGCTGAGCAGAACAGAGCCAAACCAACCCAAAAAGCATTTGGTA

CGAGGACTCTAGGAGAGTGAAGCCATGAAGAAGAGCGTTGTCCTGTACCCTGGCGTCGGC

GTCGGCCACCTGGTGCCGATGGTGGAGGTCGCCAAGCTCTTCCTCAAGCACGGCCTGGCC

GTCACCGTGGTGCTCAT

>Traes_7BL_F65D0F2E9

CCGCTTCGCCTCTAACTAAGTCCTCCAGCGCCGCGGCCGCGGTGTACAGCGATTTCATCT

CTGAATCTTGTTGGACTCTGCTGGATGTGGATTACCCTCTTCGGAGCGTGCATTTCGATA

AGTACATAGAGTCAAAGGTCCTGTCAGAGAAGGAGCTCCTAAGCTACAACGACGGTG

>Traes_2DS_262C61DCE

GGAGCTAACCGCGGATGTCATCTCCCACACGGCATTCGGTAGCAGCTACGAACAAGGGAA

AAAGGTCTTCCTAGCGCAGAGGGAGCTCCAGTTTCTTGCCTTCTCCACCGTATTCAACGT

GCAAATCCCAGCATTCAGGTACCTTCCAACTGAAAAGAACGTCAAGATATGGAAGCTTGA

CAAGGAGGTGAGGACCATGCTTATGAACATCATCAAAGGCCGCCTTGCCACCAAAGACAT

CATGGGCTACGGCAACGACCTCCTCGGGCTTATGTTGGAGGCGTGTGCGCCTGAGGACAG

GCAAAATCCGCTTTTGAGTATGGATGAGATTATAGATGAGTGCAAGACCTTCTTCTTTGC

CGGGCATGACACCAGCTCACACCTGCTCACATGGACCATGT

>Traes_4DL_6F5D84957

CGCTGATGAAGCCGTCAAGATTATTCTTGACGCCATTGAACAAGTAGGTGGCATCTTCGT

GGTCACCGCAGACCACGGCAATGCCGAGGACATGGTGAAAAGAGACAAATCCGGGAAACC

AATTCGCGACAAAGACGGGAATGTGCAGCCCCTTACCTCACACACACTGAATCCAGTCCC

CATCGCCATCGGAGGTCCAGGGCTTGCGCCAGGGGCTACATTCCGGGAGGACCTTCCGGA

TGCCGGGCTTGCCAACGTTGCGGCGACAGTCATCAACCTCCATGGTTTCGAGGCCCCTCA

TCACTATGAGCCAACTCTCATCCAAGTCTCGCCGGTTCCCTTCTGAGTTCTTCATCTGCC

GTTCCCCTTCCCTTTCTGTGATGGGCGCAGCCATGCTTATGTCTACGTAGTAAATAAATG

TCCACCTATGTATCTTTTTCTTTCTTTGATTTGAATTGATGTCCACTTCTGTATCTGAAT

CCGTCCGTGTGTGGTGATACCGTATCCGTATGTAGTATCTATTGTGTGCCTCTTTTTCCT

GGGTTTAGGTTTGTTGGGTGAGCGTT

>Traes_4DL_6373AED9E

GCAAGCAGATAGCAACGAACCAGAAATAATAGAGTGGGGAAATGCTGAAAACCCACCCGC

AATAGTTTTTCATAAAGATTCTCCAAAAGGATTCAAGAGACTTCTTAAATTTGCTCGGAA

AAACAAAGGAGACAACAACACTAATGGTTTGGCAAGCCCATCGGTGATTTCTGATGGAGA

GGATGAACAAGAAGAATCCGGTGCTAGTGATGGTGTAAATTCCAGCAGGAGAACTTTTGA

TGGTTCCAAGACTAATAGCATCTTATCAGCTCAATCAACGACCAGCAGCTTCAATGCCAC

AAGCTCGGATAGGCTACGGGACAGGCCTGGAGCTGCACCGTCAACTAAAGCAGCATCAAG

GTCATTCTTCTCCCTCTCAAACTTCCGGAGCAGCAAAACCAACGAGTCAAAGCTTCGATA

GATAGCATGCGTTGTTATACGTTAGATGTAAAAAATGGTTTGAGGTGACTGATTGTTGAG

AACGGAGCCAGGTTGTTTGTGTCCGGTTCTGCTTTCCTGCTGTTCGATATTAGCTTCTCC

TTAGCGGTCCGCATATCGTTACTTTGCTCATGTCTTGCTGTACATCTTTTAAGTATTTTT

TTGAGTTGTTTTAGATGACCGGTATGTTATCTTACTGGTACTATTTTTCTTTTTTGCGGG

TGATCTTGGCTGATAACTGCCGCAAAAGAAAAAGACAGATTTAACTGCATATCCAGCTAG

TTCGTTATATCAGGCAGCTTGACTAGTACTGATTGATGATCTAATGTTGGTGTCGCGAAC

ATGTGAGATTTGAAGCTAAAACTCTGAAG

>Traes_4DL_6C2F36471

GATGGAGACGCTGTTGTGACATTTAACTTCAGAGCTGATCGAATGGTGATGCTTGCAAAG

GCGCTAGAGTACGAGGATTTCGACAAATTCGACCGTGTCAGATTCCCGAGGATTCGCTAC

GCCGGTATGCTTCAGTATGACGGCGAACTAAAGCTACCAAGTCATTACCTTGTTGCTCCC

CCGGAGATAGAGAGGACGTCCGGTGAGTACCTGGCGCGCAACGGCGTACGCACCTACGCT

TGCAGCGAGACTGTGAAATTTGGTCATGTCACCTTTTTCTGGAACGGGAATCGGTCGGGC

TACTTCAACCCAAACCTGGAAAGATACGAAGAAATTCCAAGTGACACCGGCATACCCTTC

AATGTACAGCCAAGAATGAAGGCCGTGGAAGTTGCAGAGAAGGCACGGGATGCCATCCTC

AGTGGCAAATTTGATCAGGTACGGGTAAACATCCCGAATGCAGACATGGTTGGGCACACA

GGTGACCTTGAAGCCACCATCGTTGGGTGCAAGGCCGCTGATGAAGCCGTCAAGATTATT

CTTGACGCCATTGAACAAGTAGGTGGCATCTTCGTGGTCACC

>Traes_2DL_F91112558

GCGGCAGCATCCACCGCAACTACGACCTCCTGCTCCGCCACCGCCTGCACATCGTCGGCG

AGGTGCAGCTCCCCGTGCACCACTGCCTCCTCGCGCTCCCGGGCGTGCGCAGGGAGGACA

TCACCCGCGTCATCAGCCACCCGCAGGCGCTGGCGCAGTGCGAGCACACGCTCACCCGCA

TGCCCGGCCTCAACGCTGCCCGCGAGGCCTTCGACGACACGGCCGGCGCCGCCGAGTACG

TGGCCGCCAACGGCCTCCGCGACACGGCTGCCATCGCGTCCTCCCGCGCCGCCGAGCTGT

ACGGCATG

>Traes_2DL_E0847E88D

TGGTGAGCCAGTACTCCCTGTACGGGTACGACGCGGCGGCGCACCTGACGGAGGAGACCA

AGGGCGCCGACAAGAACGGCCCCATCGCCATCCTCTCCAGCATCGGCATCATCACCGTCT

TCGGCTGGGTCTACATCCTCGCCCTCACCTTCAGCATCCAGGACTTCGGCTACCTCTACG

ACCCGGCCAACGAGACCGCCGGCACCTTCGTCCCGGCGCAGATACTCTACGACGCGTTCC

ACGGCCGGTACGGC

>Traes_4DS_63083B4941

CTTAGCAGCAGCAACAACCAGTGCCATAGACACTCTCCATCAACAAACTCTAGCTGATCA

ATCCTAGCTAAGCTTATTACATAGCAAGCATGGGGTACTCCAAAACCCTAGTAGCTGGCC

TGTTCGCAATGCTGTTACTAGCTCCGGCCGTCTTGGCCACCGACCCAGACCCTCTCCAGG

ACTTCTGTGTCGCCGACCTCGACGGCAAGGCGGTCTCGGTGAACGGGCACACGTGCAAGC

CCATGTCGGAGGCCGGCGACGACTTCCTCTTCTCGTCCAAGTTGGCCAAGGCCGGCAACA

CGTCCACCCCGAACGGCTCCGCCGTGACGGAGCTCGACGTGGCCGAGTGGCCCGGTACCA

ACACGCTGGGTGTGTCCATGAACCGCGTGGACTTTGCTCCCGGAGGCACCAACCCACCAC

ACATCCACCCGCGTGCCACCGAGATCGGCATCGTGATGAAAGGTGAGCTTCTCGTGGGAA

TCCTTGGCAGCCTCGACTCCGGGAACAAGCTCTACTCGAGGGTGGTGCGCGCCGGAGAGA

CGTTCCTCATCCCACGGGGCCTCATGCACTTCCAGTTCAACGTCGGTAAGACCGAGGCCT

CCATGG

>Traes_1DL_1B7CF6A10

CGCCGACATCGGCCCCTCCCTCCTCCCCTCGGACCCCCACCAACGTGCCGTCGCTCGCTT

CTGGGCCGCCTACATCGATGACAAGCTCGTGATCCCATGGGTGCGGTCGTTCAGGGGCAA

GACGGAGGAGGAGAAGTCTGAGTGGATGGAGCAGAC

>Traes_4DL_95D495343

CTTTAGTTGGTTGATGAGGTGCCTACTGTTGATCCGGGATTGCCAGCCCTCAGAGCTCCC

CCCACACCAAGCTTCTACTGCTGAGAGAAAGGATGATGCACTTGAAACTCGTGATATCAG

TTCGCAAGCCAATATCCAAAGGCATATTACCAGTAATGTTTCCCCAGCAAAGCTTGCACA

AGTTGAACGTAAAATGTCAATGGAAAGCGACGATTCCTCTGAGTCCACCGATGAAGATGA

AGCTGTTGTTGAAAGAAGCCGCCCCCTTATGAGATCTGCTTCTCCTAGGAGGTCTGCTTC

TCCAATGCGAAGGGTTCAAATTGGGAGATCAGGATCTCGTAGGTCAACGCCAATTGCCAT

CAAGAGCCTAAGTTACTTCCCTCCTAGCCAGAGAGTTGCTTTGGATAAAGATGATGAAAG

CAGCTGCAATGGTGAAACAGACCAGCCTCCAAGGAGATCCGACAATAATGTAAGGAGAAT

GAGCGTGCAAGATGCGATTAGCCTTTTCGAGAAGAAGCAGAAGGGTGAGAATCTGGATTC

TGAAAGTAAGAAAGCCGGCTTGGTCGCTACCAAATCTGTACTGCGTCGGTGGAGTTCAGG

AATGGGTGACTCTTTGAACAGCAATACATCGGAAGAAAAAACCTCAGATTCTACATCTCA

AAGCAAATCTAACAATATGGCTTCTGATGCAGAGAAGAATGAAGCTGAATTACAGGCTGA

GACAGATGCAGCGCCAAACAGTGTAGTTGCACCTGAGGCAGGAAGTTACGATGCTGATGG

CCATGGCATCACAGTGTCGGAAATGGAAAATGTGGTCTCATCCCACACTAACATTTCTGC

TGAACAAACACATTCTGGGCAGGAGTCAAACAGTGATAGGGCAGTGGCCTCTGCTGAGTG

GAATCGTCAGAAGGAAGCCGAACTTAATCAGATGTTAATGAAAATGATGGAGGTCATGCC

TGGGAAGTTTGCAGGCGCCAATGTAACTGCTACCGGGCTCATTTCTGCAAGTGAGAAGAA

AGGTGGACTTCAAAGAGAGAAGCGAGACACAAAGGTTCGAACAGAGAAAAGTGTAAAGCG

ACCAGCAAAGGAGACGAGCACCAAGCTCTTGAAGGAATCCGTTGGGCAGAACAGAGCAGC

AGTTACCCCCAAGACCAGCATCACAACAGAGAAGCGTAATTCACCTATTCCACAAAGGGC

AAGGAGAAATTCATCACCTCCAGTCTTGCCAAAGGAAGTGATCTCAAAGACACCAGCAAA

AAAAAGTTCCCCCAAACCATCACCTGCACCAGCTACCCGTAGTTCATGGTCAGGATCTTT

GACTAAAGCAACTAGTAGTACTGCACAGAAAACTAAAAACTCTCCTGGAGCAGTTTCAAC

ATCGACACCAACTAGCCGGAGAAGGACCGCGACAGCATCCTTGGCACCTCAGCCGATTTC

AAAGGTGGAAAAACCCCTTCAAGCAGTGAAGAACAAGAAGGAACCTGTGACTGCGACAAA

GACAGCCATTAAGGGGCAGGAAGATAAGAAGACAAGGACAGCAACAAAGCCAAGCAGAGT

GGCTAAAAGTTCACCTGCATCAGAAGAAAAATCAAGTGCAATGACAAAGTCAGGCATGTA

TAACAAGGTCCCAAAGAAAAGCAGTGTTGTACCAGTAGAATCCAAACCCATGAAGAAAGC

CACTGGGATTAGTCAAAGTGTTGGTTCTGGTGCTGTTAAGAGTAAAGTGCCGCAGCTTGG

TGATTCTTCAAAGGGCAGTGGAATTGTTACCCGTGCGGAAGATAAGGAGCAATCTCCTGT

GACAACCGAGCCAACTACCAAGAAAACCCGGGCTCGAGTTTAAGCGCAGCCGAAATGGGC

CCCAGTGACCAGGTTGAGCCCTCTGTCGTTGAGGTCAAACCTCTTGACGAGGACATGGAC

ATCTCATCAGCTGCCTGGGTTGAAGTGGATCATCAAGAAGTTACTGATGTGGGTGAAAGC

GTGACAGGCGAGGA

>Traes_4DL_A780BF320

CCCCGACGGCCATGGCTTCGAGCCCAAGATGCTGCAGTTCGGCGGCCAGGGCTTCCGCGC

GGGCTTGGTCCCCTGCAACCGCACCGACGTCTACTGGTTCTTCACATGGTCTCCTTCTCC

TACTCCGGACGGTAAGGATGACGCCGAGCAGAACCCGGCGGAGATGAAGCAGTTCGTGCT

GGCCAAGCTCAGAAGCATCAAGGCCCCCG

>Traes_2DL_6EE2CD3A7

CGAGGTGGCTTTCCAGGCCGTGGAGAACTGGGTCGCGGACCGCGCCGTGCTCCCCGTGGA

GAACTCGCTCGGCGGCAGCATCCACCGCAACTACGACCTCCTGCTCCGCCACCGCCTCCA

CATCGTGGGCGAGGTGCAGCTCCCCGTGCACCACTGCCTCCTGGCTCTCCCGGGCGTGCG

CAAGGAGAACATCACCCGCGT

>Traes_7AS_E0F56CCBF1

GCGGACAAGAACATCCTGGTGATCAAGGGCGAGGGCGAGAAGCAGCCCTGGGACGGCGAC

GACGACGACTCCGCGGTGCCGAAGTACAACCGCCGCATCGAGGTGCCCTCTGCTGACGCG

TACAAGATGGACAAGATCAAGGCCGAGATGAAGAACGGCGTGCTCTGGGTCACCCTGCTC

AAGGTCAAGGAGGAGGAGCGCAAGGACGTCTTCCACGTCAAGGTCG

>Traes_1BL_C08B037F5

GCGGCGAGCAGCTGGACGCTGTGCACGCGGACGGACGTGGAGGAGGTGAAGCAGGTGGTG

CGCATGCTGCCCATCTGGGCCACCACTATCATGTTCTGGACCATCCACGCGCAGATGACC

ACCTTCGCCGTCGAGCAGGCGTCCGTCATGAACCGCGCCATCGGCGGCTCGGGGTTCCTC

ATCCCGGCCGGCTCCCTCACCGTCTTCCTCATCAGCTCCATCCTCCTCACCGTGCCCCTC

TACGACCGCCTCGTGGCCCCCGTGGCCCGCCGCGTCACCGGCAACCCGCACGGCC

>Traes_6DS_8F684013D

GAGGGCGGGTCGGTGTCGCCGTCGAGGAAGCGCAAGAGCGAGGAGAGCCTCGGCACGCCG

CCGCCCTCGCACCAGCAGCACTACGCCGCCGGCCTCGCGTACGCGGCGGCGCCGGACCAG

GCGGAGTGCACGTCCGGCGAGCCGTGCAAGCGCATCCGGGAGGAGTGCAAGCCCGTCGTC

TCCAAGCGCTACGTCCACGCGGACCCCGCCGACCTCAGCCTGGTGGTCAAGGACGGGTAC

CAATGGCGCAAGTACGGGCAGAAGGTGACCAAGGACAACCCCTGCCCCAGAGCCTACTTC

CGGTGCTCCTTCGCCCCCGGCTGCCCCGTCAAGAAGAAGGTGCAGAGGAGCGCCGAGGAC

AAGACCATACTCGTGGCGACGTACGAGGGCGAGCACAACCACTCCCAGCCCCCGCCG

>Traes_1AS_2B48731F7

GCACGAGTCCGACAACCACCAGAAGGACGACTTCGCCGGCCAGACCTGCCTGCCGGTGTG

GGAGCTCCGGTCTGGGATACGCTCCGTCAGGCTCTACGCCCGCGACGGCGAGGCGCTGCG

CTCGGTAAAGCTGCTTATGCGCTTCGAGTTTTCATAGAGGATCGAGCTCGCGGGCGTTTC

CCTGTTGATCCTGGCGATGCTGGTGGTGTGTGTAAAAAGATTGGAAATTTTTGTTCGT

>Traes_2DS_57B198E83

CAACGATCCTTCAGGTGATCATGCATTAGCCATTCTTCCGCTCATAGGTGGTGTCGCAGT

TGGGAAGAAAACTTTGGTTGCTCATGTGTGTGGCGATGACAGGGTCCGTTCACGCTTCTC

CTCTATTTTGCACTTGAATGGAGACAACCTTTTGGGGATACTTGGTGATGGGAGGGCCAT

GATTGAGATAATGTTGGTAGTTATTGATTTCACTTCTGATGTAGGTGATGATGACTGGAA

AGTTTTTCACTCATTTCTCATAAGAATGGGCAGAGGAAGCAAGGTCATCATTGTAAGTAA

GCTTAAAAGAATAGCCCGGTTTGGAACGGTGAAACCAATTTTACTAAGTG

>Traes_4BL_6EFA511F0

CCTACTCGGACAACCAGCCCGGCGTGCTGATCCAGGTGTACGAGGGCGAGAGGACGAGGA

CCAAGGACAACAACCTGCTGGGCAAGTTCGAGCTGTCCGGCATCCCGCCGGCGCCCAGGG

GCGTCCCCCAGATCACGGTGACCTTCGACATCGACGCGAACGGCATCCTGAACGTGTCCG

CGGAGGACAAGACGACCGGGCAGAAG

>Traes_1DL_7BCE5B151

ATCGATGAGGCGTTCGCCGGCATCGGCCCCGCTCTCCTTCCCTCTGACCCCTACGAACGC

GCCATTGCCCGTTTCTGGGCCGCCTACGTTGACGATAAGCTCGTCGCCCCATGGGTACAG

TCGTTGAGGGCCAAGACAGAGGAGGAGAAGTCCGAGGGGCTTAAGCAGACATTTGCCGCG

GTGGAGACACTGGAAGGAGCCCTGCGGGAGTGCTCCAAGGGAGAGGGCTACTTTGGTGGT

GAGACCGTCGGGCTTGTGGACATTTCACTTGGGAGCCTGCTCTCCTGGTTGAACGCGACA

GAAGTGATGTCCGGAACCAAGATATTTGATCCTGTTAAGACTCCGCTCCTGGCAGCGTGG

ATGGAGCGCTTTAGCAAGCTCGATGCTGCCAAGGCGGCGTTGCCAGAAGTTGATAGGGTG

GTCGAATTTGCCAAGAAGAGACAAGCACAGGCTGCTGCCGCCGCCGCTGCTTCAGAGACC

AAGTAAACATCATGGGGACGTGACTTCCATTGAATAAAAAAAAGTGCATGTTTTCTATAT

AGCATATATGATTGTTGTTTGTATGTCGATTTTTGTTGTAATGGTGCCAAGGATAATTAA

TGCATGTGATATTTTGTGAAGGTTTTAAGATCATTTGTAATATTTTGTGAAGTTTTAACA

TCATTTACTACTTGTATTCTTTTTGGAATTCGTATTTCTGGTCTTGCCATTTGAATAGTT

TCACTTCTTAGAAAATAAAGTTGTACTAAGTCTGCGACATTTATTTTG

>Traes_2DS_3196783D3

CAGGGCGCACGTTCTTGTACTGGTTCGGAGCCAGGCCGAGCCTGTGCGTGGCCGACGTGA

ACGTGGTGAAGCAAGTGCTCTCCGACCGAAGCGGGCTGTACCCCAAGAGCATCGGGAACC

CGCACATCGCTCGGCTGCTCGGCAAGGGGCTCGTGCTCACCGACGGCGACGACTGGAAGC

GCCAC

>Traes_5BL_8E504A80B

GCAAGGGCACCATCAGGACCGGAGATGTGCAGTGGATGACGGCGGGGAGTGGCATCGTGC

ACTCAGAGATGCCGGCAGCAGACGGAGTGCAGAAAGGCCTGCAGCTCTGGATCAACCTCG

CCTCCAAAGACAAGATGATCGAGCCGCGGTACCAGGAGCTCGAGAGCAAGGACATTAGCC

AGGCCGAGAAGGACGGCGTGGCGGTGCGGATCATCGCTGGGGAAGCATTTGGGGTGCGGT

CGCCGGTCTACACGCGGACGCCAACCATGTACATGGACTTCACAATGCAACCAGGTTCGC

AGCTCCACCAGCCAATCCCCGAGGGCTGGAACGCCTTCGTGTACGTCATCGAGGGGGAGG

GCGTGTTCGGCAAGGAGAATGCAGCGCCGGCGAGCACGCACCACTGCCTTGTGCTTGGCG

CGGGGGACGGGCTCAGCGTGTGGAATAGGTCAGGCGCACCGCTGCGGTTCACCCTTGCGG

CGGGGNNNNNNNNNNNNNNNNNNNNNNNNNNNNNNNNNNNNNNNNNNNNNNNNNNNNNNN

NNNNNNNNNNNAGGACTACTACTACGGCCGCAACGGCTTCGAGAAGGCCAGCCAGTGGAG

CTCCGCCTGATTCTCATCGGTGTCTCGCCGTGATGATGATCAACATCTGGTGCATTGTTG

TGCAAAATATTGGGAGGGAAAGGGAAATTGCATTGTACCATTATATTTATGCCTTTGTGT

AGAACGTAGAACTAGTCTTGTTTTTCTTTGATTTTTTTACTTGTTTATAAAAGAATCTGA

TGCAATAAAAGTGTGTAATGAGGAGGAACTTATTAATGATTGTTTCATCGTAAAAAAGCG

AAT

>Traes_6DS_7DD7EDF01

GGACAAGCGTGTGAGTTGTGATGTGACGCGAGCCGCTTCGTCCCCTCCCCCTCCCGCTCC

CTCCCTCCTGCGACCCGACCTTCCTAGCCAGCGACTTCGCTAGACCACTAGTCCCGTCTC

CCGTGCTTGGCTGGCTATATAACACGACTGCTCCACGCCATTCTCCTCTCACTCGGAACT

CGGAAATAGTTCTCCATCTCAACCTTCTCTTCTCCCTTCTCTTCTCTCCCGCGCGTTACC

TCGAACCGGAAGCGAACTCTACATCCATCCTCGACCGATGGATCCATGGGTCAGCAGCCA

GCCTTCCCTTAGCCTCGACCTGCACGTCGGCCTCCCGCCGATGGGGCACCCGCACCACCA

CCAGGNNNNNNNNNNNNNNGGTCGCGCTGGCCAAGCCCAAGGTCCTCGTCGAGGAGAACT

TCATGCAGCTCAAGAAGGACCCTGAGGTTGCGGTTCTTGAGTCTGAGCTACAGCGGGTGA

GCGAGGAGAACCGGCGGCTGGGCGAGATGCTCAGGGAGGTGGCCTCCAAGTACGAGGCCC

TGCAGGGCCAGTTCACCGACATGGTCACGGCCGGCGCCCACGCCGGCGGCAACAACAACA

>Traes_1AL_B427A0153

GGAGAGCAAAAGCAAATGGGTGTCGCCCAAGAGCACGCTGGACGCGGCATTCTGGGGGTC

GGAGTCAGACACCGAGGAGGCGGAGCACGACAGCACGGCCGAGAGGAGGATCGGAGCATT

GGCCTGCCCTGCCTCGGCGCTCCCGGACTGGGACTCCGACGAGGGCTGGATCGACGTGCT

CTCCGCACCAACCGAAGCAGCGGACGCAGACGNNNNNNNNNNNNNNNNNNNNNNNNNNNN

NNNNNNNNNNNNCAGTACTAGATGCAGAGTCGGGCTTTCCCGACATTGCTGTTGACGTAG

AGCACAGCAGGAGCTTCCTCGACGCAGGAGAAGCATATGAGGCTGATCCAGTCGTTAGGC

ATTACCAGTTTTTGAAGAGTTTAGTTTCTGGCCAATTAGTGTCATGCACTTGTAAATTAT

TAGTGCAAAGAGTACTGTTTTGCAACATAATCACTGATGCAATCGAATTCGTTGATACAC

AAACAGTATCCCGCTTGGCGCCGCTCCTCTGTTT

>Traes_1BL_D31C26D69

AGTTGATGCACTCGGGGCTATAAAGGGAAGCTTACTTGATCCTATGAATAACCTTGAGAA

CTGGAACAGGGGAGATCCATGCACTTCTAATTGGACAGGAGTTTTCTGCCACATCATCAA

TGATGCGCTTCATGTGACAGAATTACAGTTATTTAAGAGGAACCTGTCTGGAACTTTGGC

ACCAGAGGTCAGTCTTTTATCTCAGCTGAAAACACTGGATTTTATGTGGAACAATTTAAC

AGGCAGCATCCCAAAAGAGATAGGAAACATCACTACACTCAAACTTATACTATTGAATGG

CAATCAGCTCTCTGGTTTCCTACCAGATGAGATTGGCAACCTTCAGAACTTGAACAGGTT

ACAAATTGACCAAAACCAAATACTGGGACCGATACCGAAGTCATTTGCCAACTTAAGACA

TGTGAAACATCTGTAAGAAATGTTCATTTCCATTTTCCCTATGCTTTGCTAATTGATGTT

CAATGTGAACTTAGCTCTAACCTCTGTTTTCC

>Traes_3AS_396386369

CCGAGGCGCACGTCTTCAAGGCCGACCTCCCCGGCGTGAAGAAGGAGGAGGTCAAGGTGG

AGGTGGAGGACGGCAACGTGCTCGTCGTCAGCGGCGAGCGCACAAAGGAGAAGGAGGACA

AGAACGACAAGTGGCACCGCGTGGAGCGCAGCAGCGGCAAGTTCGTCAGGCGCTTCCGCC

TCCCCGAGGACGCCAAGGTGGAGGAGGTGAAGGCCGGGCTGGAGAACGGTGTGCTCACCG

TCACCGTGCCCAAGGCCCAGGTCAAGAAGCCCGAGGTGAAGGCCATCCAGATCTCCGGCT

GAGTGGACGCGTCTCGGCGTATGATCATCAGGGATGGAGCCAGTTTGGTTGATGTGTGTG

CGAGGTCTGCAACCTGAAAGTATGCTACAGTTGTGTCTTGTTCTTGGCGAGTCTGTTCGG

ATGGAAAGGATCAAGAGAAAAATGC

>Traes_1BL_9C0934A77

CTTTGGTCATGGTCGACGTCTAATTAATGAATCTATGCATGCTCTGTTTCTCCTACAGCC

GCGCACACGATCGGGACGTCGCACTGCTTCTCCTTCTCGGACCGGCTCTACAACTTCACC

GGCATGGAGAACGCCAGCGACATCGACCCCTCGCTGGAGCCGCAGTACATGATGAAGCTC

AAGAGCAAGTGCGCCAGCCTCAACGATAACACCACACTCGTGGAGATGGACCCCGGCAGC

TTCAAGACCTTCGACACCGACTACTTCAAGCTGGTGAGCAAGCGCAGGGGCCTCTTTCAC

TCTGACGGCGCCCTCCTCACCGNNNNNNNNNNNNNNNNNNNNNNNNNNNNNNNNNNNNNN

NNNNNNNNNNNNNNNNNNNNNNNNNNNTCCATGGTCAAGATGGGCAACAACCAGGTGCTC

ACCGGCAGCCAGGGCGAGATCAGGAAGAAGTGCAGTGTGGCTAATCATTAAGTCACCGAC

CGACGAAGTACAATGCTTTTGTTTAGTTTGTGACCATCTTTTCCCATGTAAATTGCTGTA

AAAGATTGGTATACCTCCTTTCTCTCCCCATAAAAAT

>Traes_4DL_382DEDEB6

CTCGACTCAGGGCGCAGGGCCACGCGGGGGCACGCCAAGTACCCCGACGGCCACGGCTTC

CAGCCCAAGTTCATGCAGTTCAGCGGCAACGGCTTCCGTGCCGGCCTGGTGCCCTGCGGC

GACACGGACGTGTACTGGTTCCTGACGTGGTCGCCTTCCTGTCCGGACGGCACGGAGGAC

GTCGACCCGAGCCCGGCGGAGATGAAGCAATTCGTGCTGGCCAAGCTGAGGAGCATCAAG

GCCCCCGCCGAGGTGCTGGAAGCGGTCGAGAGGAGCGAGATGAACGACGTGCTCGTGGCG

CCGCTGCGGTACCGCCCGCCGCTGTCGCTCCTCTTCGGGAGCATCAGCAAGGGGAACGTG

TGCGTCGCC

>Traes_2DS_0904D19B8

CACGAGGACTCGAGCGTAAGGATCATCCACCGAGACCTGAAAGCTAACAACATTCTGATT

GATGATGCCATGGATCCGAAGATTGCGGACTTCGGGTTAGCTAGGCTGCAAGTCGGAGGC

CATACTCAGACCATGACAGCTAGAGTTGTTGGAACATACGGGTATATGGCGCCGGAGTAT

GCAATACACGGAAACGTGTCGCCAAAGATCGACATTTTCAGCTTTGGTGTCTTGGTGCTT

GAAATTGTAACCAAGAGGAGGAACTGCGGCTCAGATGATTGCGAGACGGATACGGTGAAT

CTCCTGAGCGATGTAA

>Traes_1BL_9D96A6922

GCAGCTCAAGCTTCCCCTGATTAACTAGTCACCTAGCCATCGTCGTTTCTTGCATAGCCA

AGCAAGAAACAAGCTTCTTGACCGAGAAGATGGCGTTGAGGGCTAGGACGATGCTGGCGC

TGCTGCTCGCCGCGGTGGCGGTGACATGCGCGCGGGCGCAGCTGCACGACAATTTCTACA

GCGAGTCGTGCCCCAGCGTGGAAGACGTCGTGAGGAAGGAGATGGTGAGGGCGCTGTCAC

TGGCGCCCAGCCTCGCCGGGCCGCTCCTCCGTATGCACTTCCACGACTGCTTCGTCAGGG

GGTGCGACAGCTCGGTTCTGCTGGACTCGGCCAACAAGACGGCGGAGAAGGATGCGCAGC

CGAACCAGACGCTGCGAGGCTTCGGATTTGTCGAGAGGGTGAAGGCCGCGGTGGAGAAGG

CCTGCCCCGACACGGTCTCCTGTGCCGACATCCTCGCCCTCATTGCCAGGGACGCAGTAT

GGCTGAGCAAGGGTCCATTCTGGACAGTTCCTCTCGGCCGNNNNNNNNNNNNCGTGTCCA

TTTCCAACGAGACCGACGCTCTGCCACCCCCGACCTCCAACTTCACCGTGCTCACCCAGC

TCTTCGCCGCCGTGAACCTCGACGCAAAGGACCTTGTCGTCTTGTCCGCCGGGCACACCA

TCGGGACGTCGCACTGCTTCTCCTTCTCCGACCGGCTCTACAACTTCACCGGCATGGAGA

ACCCCAGCGACATCGACCCCACGCTGGAG

>Traes_2DL_8C21AAAEF

CTGCCTGCCGCGGCGACGAGGGCGGCGATGCTCGTCCGAGTCAATACCTTGCTCCAGGGA

TATTCAGGCATCCGCTTCGAGATCCTCGAGACGGTCGCCACACTTCTCAACGCCAACGTG

ACACCATGCCTACCGCTTCGGGGCACGATCACCGCATCCGGTGACCTCGTCCCGCTTTCG

TACATCGCGGGCCTGGTCACCGGCCGCCCAAACTCCATGGCGACTGCTCCAGATGGCACG

AAGGTTAATGCCGCTGAGGCATTTAAGATCGCCGGCATCCAGCATGGCTTCTTCGAGCTG

CAGCCAAAGGAAGGCCTAGCCATGGTGAATGGCACGGCAGTGGGCTCAGGGCTTGCATCC

ATGGTGCTTTTCGAGGCTAACATCCTTAGCCTCCTTGCCGAGGTCCTGTCAGCCGTCTTC

TGCGAGGTGATGAACGGCAAGCCAGAGTACACCGACCACTTGACCCACAAATTGAAGCAT

CACCCCGGGCAAATCGAGGCTGCTGCCATCATGGAGCATATCCTTGAAGGCAGCTCCTAC

ATGATGCTGGCGAAGAAGCTCGGTGAGCTTGACCCATTGATGAAGCCAAAGCAAGATAGG

TATGCACTCCGCACATCGCCGCAGTGGCTTGGCCCTCAGATTGAGGTTATCCGTGCTTCC

ACCAAGTCGATCGAGCGGGAAATAAACTCCGTCAACGACAACCCACTCATCGACGTCTCC

CGTGGCAAAGCTATCCACGGTGGCAACTTCCAGGGCACACCCATCGGTGTGTCCATGGAC

AACACCAGGCTTGCCATTGCTGCGATTGGCAAGCTTATGTTTGCCCAATTCTCAGAGCTG

GTGAACGACTTCTACAACAACGGTCTTCCGTCAAACCTCTCCGGTGGGCGCAACCCAAGC

TTGGACTATGGCTTCAAGGGTGCCGAGATTGCCATGGCCTCCTACTGCTCCGAGCTTCAG

TTCTTGGGAAACCCTGTGACCAACCATGTCCAGAGTGCGGAGCAGCACAACCAAGATGTC

AACTCTCTTGGTCTCATCTCCTCGAGGAAGACCGCCGAGGCCATTGACATACTCAAGCTC

ATGTCCTCGACGTTCTTGGTCGCGTTGTGCCAGGCTATCGACCTCCGTCACCTTGAGGAG

AATGTCAAGAATGCTGTCAAGAGTTGTGTGAAGACTGTGGCTAGGAAGACACTGAGTACC

GATACCAATGGCCATCTCCATAACGCGCGCTTCTGCGAGAAGGACCTTCTGCTCACAATC

GACCGTGAGGCGGTGTTTGCGTACGCAGACGATCCTTGTAGCGCCAACTACCCACTCATG

CAGAAGATGCGTGCAGTTCTTGTGGAGCATGCCTTGGCAAATGGTGAGGCCGAGCGCGAC

GTGGAGACGTCGGTGTTTGCCAAGCTTGCCACGTTCGAGCAGGAGCTTCGGGCAGTGCTG

CCAAAGGAGGTTGAGGCTGCCAGGAGCGCCGTGGAGAATGGCACTGCCGCACAGCAAAAC

CGTATTGCCGAATGCCGGTCGTACCCGCTCTACCGATTCGTGCGCAAGGAGCTTGGAACG

GAGTACTTGACTGGAGAGAAGACAAGGTCTCCTGGCGAAGAG

>Traes_4DL_2FA43439B

CCACACAAGTTTCCAAGTGCCCTTGAAGCCGTGAAGAAGCTGAGGGATGATCCCAAGGCC

AATGACCAGTATTTACCCCCGTTCGTCGTCCTCGATGAAAGCGGAAAACCGGTTGGGCCC

ATAGTGGATGGAGACGCTGTTGTGACATTTAACTTCAGAGCTGATCGAATGGTGATGCTT

GCAAAGGCGCTAGAGTACGAGGATTTCGACAAATTCGACCGTGTCAGATTCCCGAGGATT

CGCTACGCCGGTATGCTTCAGTATGACGGCGAACTAAAGCTACCAAGTCATTACCTTGTT

GCTCCCCCGGAGATAGAGAGGACGTCCGGTGAGTACCTGGCGCGCAACGGCGTACGCACC

TACGCTTGCAGCGAGACTGTGAAATTTGGTCATGTCACCTTTTTCTGGAACGGGAATCGG

TCGGGCTACTTCAACCCAAACCTGGAAAGATACGAAGAAATTCCAAGTGACACCGGCATA

CCCTTCAATGTACAGCCAAGAATGAAGGCCGTGGAAGTTGCAGAGAAGGCACGGGATGCC

ATCCTCAGTGGCAAATTTGATCAGGTACGGGTAAACATCCCGAATGCAGACATGGTTGGG

CACACAGGTGACCTTGAAGC

>Traes_2BS_11EB60656

TGACATTTCATACATTCATGCTAGTAGCAATTCTACATCTAATGTCCCATAGTATCAGGT

TAATAGTCTAATATTTTGGCCACCATTTGTTTTTTTACACGAATTATCAGCAATTTGCAT

CATTCCAAGTATATATTTATTTATTACTGCTACACAATTTTGAAGATGTAGTGTTTCTCT

TTGTAATGTTGACTCTGTAATTGATTAATATTGTATGTCTGTAATATTTCAGGCTATTTT

CACTGCTGGTTACAAGGCAGTGTTACACATCCACTCTGTTGTCGAGGAGTGTGAGATTGT

TGATCTCATAGAGGAAATTGACATGAAGAAAGCAAAAGTAACTGACCCAAAGAAAAAGAA

GAGCAAGAGGAAGCCTCTTTTTGTGAAGAATGGTGCAGTTGTAGTTTGCCGCGTCCAGGT

GACTAATTTGATATGCATAGAGAAGTTCTCCGACTTCCCTCAGCTTGGAAGGTTTACTCT

ACGAACTGAAGGGAAGACAATAGCTGTAGGCAAGGTTGTTGATGTGCCTCCAGTTGGCAG

GTCAACGTTTTCAGCCTAAGCCAGTTTTGACAGGATGATGGGAGAATGAAGTGGCGCTGT

CTTGAAATAGTTTTTGGAAGCAGCAGTGAGATCGATTTTTCCAGCCAGGCTCCATGAGTC

ACTTAGCATTAGTTTATTGCTGAGGTCCCTTTATTGAGTTGGGGGTGTATAAGTGTTGTA

GCTCTGGTCACATGTTTGAGAGGTTGTAAAATCAGTGTGGTTATATGTTCCCGCTACCCA

ATCTGATATGATGACAATATAACAGGTGTACTGTACTCCCATCTTTTTTCCTCCCCATTA

CGGCATGCTGCTCCCAGTTTCAAACAATCC

>Traes_1AL_5DDBD6138

GGGTGCGACGGCTCGGTTCTGCTAGACTCGGCCAACAAGACGGCGGAGAAGGACGCGCAG

CCGAACCAGACGCTGCGAGGCTTCGGCTTTGTCGACAGGGTGAAGGCCGCGGTGGAGAAG

GCCTGCCCCGACACCGTCTCCTGCGCCGACATCCTCGCCCTCATTGCCAGGGACGCAGTA

TGGCT

>Traes_3DL_BCE8DACDB

AAGGGGACTACACGTTCGGCAGCCTGACTTGGCGCAATGAGCACCACTCGGTGAGGATCC

CATTAGCAGTCCGGATCACAATCCAGGATTTCTACGCCGATGTTGCATAGTAACTAGGAA

GGTCATATGAAAAATAAAGATTGGTGATGTACTTAGGATTTGTAATTTGTACTTGAATAA

ATTTGTTTGCAAGAGAACACTACTGTTTTTTCAAATTTGTCTGAATGCTCG

>Traes_4BL_8C0E579F8

GACAAGACGACCGGGCAGAAGAACAAGATCACCATCACCAACGACAAGGGGCGGCTGAGC

AAGGAGGAGATCGAGCGCATGGTGCAGGAGGCGGAGAAGTACAAGTCTGAGGACGAGCAG

GTGCGGCACAAGGTGGAGGCCCGCAACGCGCTGGAGAACTACGCGTACAACATGCGCAAC

ACGGTGCGGGACGAGAAGATCGCGTCCAAGCTCCCCGCCGAAGACAAGAAGAAGATCGAG

GACTCCATCGAGGACGCCATCAAGTGGCTCGACGGCAACCAGCTGGCCGAGGCCGACGAG

TTCGAGGACAAGATGAAGGAGCTGGAGAACATCTGCAACCCCATCATCTCCAAGATGTAC

CAGGGCGCCGGCCCGGGCGGCGCGGCCGGCATGGACGAGGACATGCCCGGCGGCGGCGCG

GGCACCGGCGGTGGGAGCGGTGCCGGGCCCAAGATCGAAGAAGTGGACTGAGTGAGTCGT

GACAGGTCATGGAGTGGACGCGGCTATGCCGCACTTAAAATGTTCTGATGCCTGTGTTCG

TGTGCTTCTGTCGAATAACGAGGGTGCTTGTATGAGTTTGGTTAATGACCTAGTAGTACT

AGGTTAGTAGAGGAAATGATAAATAATCTGTAAAAGCAAGAACGACAATACACACGTCTG

GTTCCTGTACTATGTTGTTTTTGCCTGAGCTTCCAGGAGCTGAGAAATATATTATCAGTT

CGTTGTCCCGTTTCCTTGCGCCCATCAGAAACAGAGGTCAGAGATGCAGACATCATATTT

CATTCTCACACTGGAATCCAAATCTGATGAAGCTGAGAAGGCGTAATAAAGCTACTGTCA

TCAGCCAAATGATGCAGCAAAATCAACCTTTCTGGAGCAGTCGACCACACGCAAGCAACA

TCAACAACCGGCTATCCTCGTCTTCAACGTCAGCGACAACAAACCATAAATCTGAGTAAA

ATGCATGTGGT

>Traes_2DS_8B18A21F3

CGGGGACCCGACCCGGCGGTTCGGCACGGGGGAGGAGGGGTACGACGCGACGTACCCCAA

GATTTACTCGCTGGCGCAGTGCACGCCGGACATGGAGGCGGCCGACTGCCGGAGCTGCCT

CAGGGACATAATCGAGAAGTTTACCCCACAGTACTTCGTCGGGAAGCCGGGTGGGAGAGT

GTTCGGCGTGCGATGCAACTT

>Traes_2DL_55FF553FD

CCCAAGATTTCAACCCGAGAACATGGAGAAGAACGCGACGATATTCGAGCGTGTGAGCGA

GATGGCTGCAAGGAAGGGTTGCACGTCGTCGCAGCTCGCGCTGGCTTGGGTTCACCACCA

GGGAAGCGATGTCTGCCCCATACCTGGCACAACCAAAGTTGAGAATTTCAGCCAGAACGT

GAGAGCATTGTCTGTGCAGCTCACGGCTGAGGAGATGGCTGAGCTGGAATCCTATGCCAC

CATGGATGCTGTCCATGGTGATCGGTACCACAGCGCGTATCTGATGAATTTAAACACCTG

GAAGGACTCCGAGACCCCTCCAGTGTCATCCTGGAAAGCCACTTAGTTGGGTATCATGGG

AATCATTATGCTGATGCTTCGTTTCACTGTTTTGAAATAGTTGCAAATCAAATAATTTCA

TGACCACCCCGTGCATGGATGAAATCATGGAACCACCAATTGTTTA

>Traes_1BL_BD721F2E3

GTGCTCACCCAGCTCTTCGCCGCCGTGAACCTCGACGCAAAGGACCTTGTCGTCTTGTCC

GCCGGGCACACCATCGGGACGTCACACTGCTTCTCCTTCTCCGACCGGCTCTACAACTTC

ACCGGCATGGAGAACCCCAGCGACATCGACCCCACGCTGGAGCCNNNNNNNNNNNNNNNN

NNNNNNNNNNNNNNNNNNNNNNNNNNNNNNNNNNNNNNNNNNGTGGAGATGGACCCCGGC

AGCTTCAAGACCTTCGACACCGACTACTTCAAGCTGGTGAGCAAGCGGAGGGGCCTCTTC

CACTCCGACGGCGCCCTCCTCACCGACCCCTTCACCCGCGCCTACGTCCAGCGCCATGCC

ACCGGCGCCTTCAAGGACGAGTTCTTTGCTGACTTCGCCGCCTCCATGATCAAGATGGGC

AACGCCAACCCGCTCATCGGAAGCCAGGGCGAGATCAGGAAGAAGTGCAACGTGGTTAAC

CATTAA

>Traes_3AL_00566B27E

CAAGATACACCTGGTGGAGGAGATGAAGATTGGGGTGGCGGTGGAGGGCTACGAGGAGTC

GTCCGTCAAGGCCGAGGAGGTGCAAGCCAAGGTGAGGCTGGTAATGGAGTCGGAGGAAGG

GCAAAAGCTTCGAGAGAGGGCGGCCATGGCGAAAGGGATGGCTGCAGACGCCGTCAAGGA

AGGTGGATCTTCCGACGTGGCAATTTACGCCTTCTTGAAGCATGTGGAAGATGAAAGAAG

CTCGAGGAGCGCCAAATCCGGCCATTAATAAGTGATCAACCTCCGATGATGTGACCTCTT

TTCGTCAGAAAAGATGGGGGCGCAAGATGCGCGAGTCGTGCTGGGCTCACTCTTTCTCAG

AAAAAAGGAAGAAGGAAATCAAAAGAGATGCGGTGGCTTCTCTGGGATTCCATGGTGTGA

TGCTTGTCCTTTGCCTCTTTCGCAAGATTTGTACGGTGCAGACGTGCGTGTCGAATTCGT

AATTTAGCTCTTCTGCAACATTTGGCCTTTAGTGAGACGTTGCATATTTCTTACTGGCAG

TATGCGTACTTCCGTGTAGCCTCATATGCTCGTCGCACTCCTTCAGTATTGATTTGGAAA

TAGGATCCTCTGGCTGACGTTCACGGGGACAGGTGGCATTACGAACGTTTTGATGACAGT

TTTAGTTGGTCGAGGATGACAGTTTATTCACAAACTGTAATCTCCGATACAAACTGTCAT

CTCGTCTAGCTCTTCTGCAACATCTAGATGTGCTTTAATAAAACTGTATTTATATTTACC

TATTTTAAAGGGGAGTTATGGTCGTGATGGTAGGTTGATATGTTTTTGGTTTGGTTTGAT

TTCTCACGCAACTATTAATATCGGCAATGGTTTTAGAGGACTTGCCTTCGCCTCCAAGGT

TTTATTTCTTTGATTTTTAATCGCCTCGCCATCGCATCCCACTGGTTTATTTCCCC

>Traes_4DS_C927A96CB

ATAAGAGACTTGCATGTTGCAAAAAGAACAGAAGATATTGGGTTCTATGCTGGGTTTGTA

GGTGCTTCTTTTATGTTTGGTAGATGTTTGACTTCAACTCTTTGGGGAATAGCAGCAGAT

CGTATTGGGAGGAAGCCTGTTGTTGTGTTTGGCATTCTCTCTGTGGTGATATTTAATACT

TTGTTTGGGCTTAGTGTCACCTATTGGATGGCAATAGCTACAAGGTTTCTCCTTGGCGCT

TTAAATGGCTTACTTGGACCAATGAAGGCTTATGCTATTGAAGTTTGCCGACCTGAACAT

GAACCTCTAGCACTATCACTTGTCAGCACGGCATGGGGAATAGGTCTCATCATTGGTCCT

GCTCTTGGAGGCTACCTTGCACTGCCTGCAGAAAAATACCCAAATATATTTTCACCTGAC

TCATTATTTGGAAGGTTCCCCTACTTCTTACCATGCTTGTGCACATCAGTCTTTGCTGCC

ATTGTTCTGATAAGCTGCATATGGATGCCGGAGACGTTACACAAGCATAAAGTCAGTGAT

GATGGAAATCAAAGTGTTGAAGCTTTGGAGGCCCATCTGATTGATCCAAAAGAGGAGGTT

GGACAAAGTAATAGTTCGAATACCAAGAAGAGCTTATTCAAGAATTGGCCATTGATGTCA

TCTATAATTGTTTATTGCGTCTTCTCCTTCCACGATATGGCTTATACAGAGGTGTTCTCT

CTATGGGCTGAAAGTGACAGGAAGTATGGTGGACTGAGTTTATCATCTGAAGATGTAGGT

CAAACACTTGCAATTACAGGTACCTTTCTGTATGTGCTTTTTGTCAGTGCTATTTTATTT

TTTAATGGGAACCGGGGGG

>Traes_7BL_FAA0162F6

CCGCGGCGGCGGCGGCGGGAACTTCGGCGTCGTGCTGTCGTGGAAGATCAGGCTTGTACC

GGTCCCACCGACAGTGACGTTCTCCAACATCCAGAAGACCGTTGACCAGGGCGCGGCCAG

CGCCGTGACCAAATGGCAGACGCTCGCGCCGGCCCTCCCCGAGGACCTCAGCATACGGGT

GATCGTGCAGAACCAGCATGCCCTCTTCCAGACCCTGTATCTCGGCGGCTGCAGCGCG

>Traes_7DL_38B541C8F

CGAGGTGGCCTACAACGGGTTCCCGCTGGAGGAGTTTGTGCCGCAGAAGACGGCGGCGTA

CATCAGCCAGACGGACGTGCACGTCGGCGAGATGACCGTCAAGGAGACGCTGGACTTCTC

GGCCAGGTGCCAGGGCGTCGGCACCAAGTACGATCTCCTGACCGAGCTGGCGAGGAGGGA

GAAGGAGGCCGGCATACGGCCGGAGCCGGAGGTTGACCTTTTTATGAAGGCCACGTCGAT

GGAAGGAGTCGAGAGCAGCCTCCAGACGGACTACACCCTCAGGGTACGTACGTACGCACG

CCTTTCTGAATGTTTCCTCACTGACGTAGTAGTATGAAGTTGCAGTTTTCTTGGTCTTGC

TTTTTTGCCTGGCTGAAATTAGCACCGTGGCGAGGTTGGCGAGATGCTACAGCGACCACA

CGTTGACAGATTTGCACGGATCCAGAAAATGTTCCCCCTTTCTTGATCATGGCCATGGCC

AGTTGTGTAGTGTACTCAAGCAAGGAACAATCCCAATCAATCATGGCCAGCACGGCACAA

CGCAGAGGTGGTGGTAGTGTAGTAGGCACGGTGGCGGCACACGGTCCGCGCGAACCAAGA

GCACCAACCAAGCCGCTGCCACCTCCACTGGCTGACACGCACGCCGTCGTCGCAGCGAGG

AGTAGTAGTTGAGGCTGAGCCAGCCAGAAGGATAAGCACTGCGCTCGAGTGGTGGCAGCG

GGAGTCGGCAGCAGCAAGTAGGCAG

>Traes_7DL_2AFCB81A4

CGGCCGCTTCGACGGGAGGCACCTCGTCTACAACAACACCATGCCGGGAGCCTGCGCCCC

TAAGAGTAACTGCTCCTGCAACATCGTACCGGACTCAGGGAGGCTTTGCGGCACCGGCGG

CGGATTGGGCGGCGAAGAACTCGCCGTGTGCAGCCCGTGCAAGCAGCCACT

>Traes_7DL_07D295BFC

GTCGTCGTCCAGGCGGCTCTGCCGCGACGGCGACGTGGAGATCATGGTCATGCCGGCGCG

GGAGCGCTCGGCTTCGCAGCCTGCGCGACCGCTCGGGTGCGAGGAGTTCACGCTCAAGGA

TCTCTCGCGGCTGACCAACGCGTTCGCGGAGGAGGCCAAGATCGGGAGCGGCAGCTTC

>Traes_7DL_920EF333F

CGTGGCGCTGGACGCCGCCCGCGGCGTCGAGTACCTGCATTGCTACGCCGTGCCGCCCAT

CATCCACCGCGACATCAAGCCGTCCAACATCCTGCTCGACGACGACTGGACGGCCAAAGT

CTCCGACTTCGGCCTCTCCCTCGTGAGCGGCGCGTCGGCGTCGGCG

>Traes_4BL_43B3AD61B

GCGGCGAAGCCCGCGACGAAGGCGTACGTGACGTTCCTGGCGGGGGCGGGGGACTACTGG

ATGGGCGTGGTGGGGCTTGCCAAGGGCCTGCGCAAGGTTGGCTCGGCCTACCCGCTGGTG

GTGGCCGTGCTGCCCGACGTGCCCGAGCTCCACCGCAAGATCCTCCTCTCCCAGGGCTGC

ATCGTCCGCGAGATCGCCCCC

>Traes_5DL_331431F50

CGCCTGAGCTCGCACGGCGGGCGGCGGGACGGGTGGTTCGGCAAGGACCTGAGCCGGAGC

AGGCTGGACCTCTTCTACTGGCTGCTGGCCGGCATCTCCGCCGCCAACCTGGGCTTCTAC

GTGCTCGTCGCCACCCAGTACTCGTACAAGCAGCAGACCGCGAAGGCCGGCAGGGTCGGC

GCCGACAAGGACGCCGCCGCAGGCGACGTCGAGTGCGTCATCGCCGCCGCTGCTTAATTT

GAAGTATGATCGATGCCAATGGCATCATACGTATAGATCGTTCGTGATAATTAAACTGGT

GCGTGCGGATTTAGTTCAGTTGATAAACATGTACTTATGTTTGTTAGTAATCGACGATCG

TGTGGTTT

>Traes_2AS_CA08B5437

CGGCAGCTCCGCGCGCAGGGCGTCGGCGGTCCGGGCTACAGGTTCTTCGCCGGGAACCTC

GCCGAGATCAAGCAGCTCCGCGCCGACAGCGCCGGCGCCGCGCTGGACATCGGCGACCAC

GACTTCGTCCCCAGGGTCCAGCCGCACTTCCGCAAATGGATCCCCATCCACGGTAAAAAG

CCGAGCCTGTAGATTCCTCTCCTCTAGCAGATCTAGTTCGTGATCTCACGCCATTTCCCG

CTTGCAGGGCG

>Traes_4DS_D9BD007C1

CAGTTGGCGAGTGGGTGAGATGCAAAAAAGAACTCCCCTACACACAGGACATGCCAAGCA

GCATACCGTACCATCTTAATCTCACCACGAGAGGTTACCGTGCACTCGTATACAGCGGAG

ACCATGATCTCCAGGTGCCTCAGCTCARCACGCAGGCGTGGATAAGATCTTTGAACTTCT

CCATCGRCGATGACTGGAGGGCATGGCATCTCGATGGCCAGGCTGCAGGATTTACCATCA

CATATGCAAATAACTTGACCTTTGCAACAGTAAAGGGTGGAGGTCATACTGCTCCAGAGT

ACCAGCCTGAAGAATGCTTTGCCATGGCCCAAAGGTGGCTTGACAACAAGCCACTCTGAT

ACTTTGACAAC

>Traes_6DS_2480D42A8

GCCATGACGCTGACGAGGGACCCCAGCTTCAAGGCGGCGCTCGTCTCCGCGCTCTCCGGC

CGGATCCTCGAGCTATCGCCGACCAGGGACATCAATTAATCCCCACATCAGAAGACACTG

CGCGGCTCAAATTTTCTTGCGGTGGTCGATTACTTCGTTCGGCATTGCTGCCTTCCTCTG

CCGCCTCCGTCAAAACCTCAAAGAAAAACCAGCGAGGACCGGACTGCCTGACCGACGACG

GAGACGCGATCGGTTCCGGCGAGCCAGGAACGGAGCCACCGCCGCTGCTGCTCGTGTAAA

TACATCGCCAACACGAGGACGGCGTGGGAGGTCGTAACATGTAATGTACTCTAGTAGCTA

GGATAATGCAGTCAGTCATTCAGGAACACGCGAAAGTGTGCAATCCTTTTTTCTTTTCCT

TATTACTAGGCGAAATGTTCGTTCCATAATTTCCATGGAACTCGATTCGATTCAATGAGA

AAAATCAAAGTCACCGCACATTCTCAGTTCAATCGCTGAAATTTTGTCAATACTCACAGT

ACCCAT

>Traes_2DL_9A0F096AD

GTCCGAGTCAATACCTTGCTCCAGGGATATTCAGGCATCCGCTTCGAGATCCTGGAGACG

GTCGCCACGCTTCTCAACGCCAACGTGACACCATGCCTGCCGCTCCGGGGCACGATCACC

GCATCCGGTGACCTCGTCCCGCTTTCATACATCGCGGGCCTGGTCACCGGCCGCCCAAAC

TCCATGGCGACCGCTCCAGATGGCACCAAGGTTAATGCCGCCGAGGCATTTAAGATTGCC

GGCATCCAACATGGGTTCTTCGAGCTGCAACCTAAGGAAGGCCTAGCCATGGTGAATGGC

ACGGCAGTGGGCTCAGGGCTTGCATCCATGGTGCTTTTCGAGG

>Traes_4BL_A89017344

CCACCCCAAGGTGAAAATCATGGCTGCGGACATGTTGATAATACTACAGTTCTGAAGCTC

CACTGTCGTGTGATATAAAAAATTTGTGGTGTTCTTAAATTATTTTATTTCCCTATGCAG

ATTTGCCAGTTTGCGGAACAGAACCCAGATGTGCTGTTCTTGCATGTGAACTACGAGGAG

CACAAGTCGATGTGCTACAGCCTCCATGTCCATGTTCTGCCCTTCTTCAGGTTCTACAGG

GGAGTCCAGGGCCGCCTCTGCAGCTTCAGCTGTACTAACGCAACCATAAAGAAGTTCAGG

GATGCGCTTGCCAAGCACAATCCGGATAGGTGTAGCATTGGCCCTACCAGGGGCCTCGAG

GAGTCTGAGCTGCTGGCCTTGGCTGCGAACAAGGACCTGGAATTCACCTACACAAAGCAG

CCAGAACCAGTTCCGAGTGGAGACACGGAGGTCATTGCTCCTGGGAGCCCAAGGCTTCCT

CCACCTGCAAAACCGTTGGTTCGGCAGGGGTCCGGAGAGAGGACCTTGGTCTCATCAGGA

AGATGAGATTGTGATGTGACGATGGTCTAAATGGGATCGCGTCCATCGGTTCTCTGTACA

GTTTCTCGATTGTTGTTGGTGTGTATAGATTGTACTCTCTCATGGTTGTGTAGAGTGCAG

GCTTGATAGATTTTTACTACTTGTCAAGCCTGCCCCGCCTGTGTC

>Traes_7BL_D52D4DAC1

GTGCAACTCCTTCTATGCCAGTACCACACAAGAATCAAGTACTCCAGATTGCAGCCAACA

CCCACAATTAGGAGAGACTGAAACAAAGGTCGAGCAAAAATGGCGGAGTTCGCGCTTGGT

TTGACCAAGGCGGCGGTGGAAGGGACGCTCATCAGGGTGAAGTCGGCGATCGAGGAGGAG

ACGAAGCTGAGGGTGCGTGTGCAGGATGACCTTGTGTTCATCACTGGAGAG

>Traes_5DL_F292F9EA4

ATTTATGCTGGTGAACTTGGTCAGCACTCTCACAAATTAGTTGAATATTTTGAGGCAATT

CCAGGTGTTGAAAAGATCACAGAAGGATATAATCCTGCAACATGGATGCTGGAAGTTAGC

TCCCCTTTAGCTGAGGCTCGCCTGAACGTAAATTTTGCTGAAATTTATGCTAATTCTGAT

CTTTATAGGAAAAACCAAGAACTTATTAAGGAATTAAGCGTTCCCCCACCTGGCTATGAA

GATCTCTCATTTCCTACGAAGTATTCTCAGAATTTCTACAATCAATGTGTTGCAAACTTC

TGGAAGCAATACAAATCTTACTGGAAGAATCCACCGCACAACGCCATGCGCTTTCTTATG

ACGTTGATCTATGGCCTTGTATTTGGCACAGTGTTTTGGCAGAAGGGGACAAAAATAGAC

TCACCACAAGATTTGTCCAATCTACTTGGAGCCACTTATGCTGCTGTCTTCTTCCTTGGT

TCTGCCAACTGCATCACAGTTCAGCCTGTTGTGTCAATCGAACGAACTGTTTTCTACCGT

GAAAAGGCGGCAGGGATGTACTCTCCATTATCCTATGCATTAGCTCAGACATGCGTGGAG

ATGATCTACAACGTCGTGCAGGGGATTCAGTACACGGTCATCATCTATGCGATGATTGGA

TATGAGTGGAAAGCTGCAAAGTTCTTCTATTTCCTTTTCTTTACAATTTCATGCTTCAAC

TACTTCACATTGTTTGGCATGATGCTGGTGGCGTTGACCTCATCTTCCATGCTCGCAAAC

ATACCCATAGCCTTTGTACTCCCTCTTTGGAATCTTTTTGCTGGGTTCCTCGTTGCGAGA

CCGTTACTACCAATTTGGTGGAGGTGGTACTACTGGGCGAACCCTGTGTCTTGGACCATC

TATGGCGTCATCGGGTCGCAGTTTGGCGATAACACCAGTCCTCTGTCTGTCCCCGACGGG

AGCCGCCTGGTGGTGAAGCAATTCTTGGAGGACAATCTGGGCATCAAGCACGATTTCCTT

GGGTATGTCGTGCTCGCGCATTTCGCGTACGTCATCGCCTTCTTCTTGGTGTTCGGCTAC

TCCATCAAGGTGTTGAACTTCCAGAAACGTTAGGCAGACGGCCGTTTATTTTATGCTGCT

GCAAGAGAGAGGCGGCAATAATCTATAGTTTGTGTGTATTTGTGTATAGTAGAAGTAGTA

GGATACATACATAGACATCGTTGTACAAGCTCTGCTATTTAGAGGAATGGATTTTTTTTC

GACTTCATCAAAAATAAAAATGACATCCTTTTCCGTTTAGTTAATTTGTTTGTTGTAAAT

TCGGTTAATCGATCGCATGTAAATGTAATAGTTGTTGGTGCGGACAAGGTAC

>Traes_4DS_DE305687D

ATGCAAGAGAATTTAGCAGAGCTGAAGATGAGTACCCAGGAGCTGCAACTGGTTCTCAAA

AGAGGAGATGATGCGGCTGTTCAACTCAAGGTTGAGTCCTTCATTCGCCTGGCGAAGCAG

GCTCAGAAGCCTTTCAAGAATATGACAAGCAGCAAAGCTAGTGGTGAGGATTGCAGGCTG

GTCAGGCTACTGGCGGAAGCCAGAGAGATGTCTGTCT

>Traes_2DS_A6745CA91

GCAAGACCTTCTTCTTTGCCGGGCATGACACCAGCTCACACCTGCTCACATGGACCATGT

TCTTGCTGAGCACGCACCCTAAGTGGCAGGAGAAGCTCAGGGAGGAGGTGCTAAGAGAGT

GTGGCAACGGTGTTCCCACAGGTGACATGCTCAACAAACTACAGCTGGTCAACATGTTCC

TCCTAGAAACTCTCAGGTTATACGCCCCTGTATCGGCCATTCAGAGGAAGGCGGGTTCGG

ATCTCGAGGTTG

>Traes_2DS_CC522136E

GCGGGGACACCAGCGCCGCCAACTGCAGCCAATGCGTCGCCACGGCTTTCCAAGACGCTC

AGCAGCGCTGCCCGTACAACCAGGATGCTACCATCTTCTACGACCCCTGCGCACTCCGCT

TCTCCAACCAGAACTTCCTGTCCTCCACCAATGGCGACGGCACCCTCCTCCTCCTCATGA

ACACTCAGAACGTGTCCGCACCGGTGAAGGTGTTCAACGCCGCCGTGGGC

>Traes_2DS_0BED12E21

CAGTTGGCTTGGTTAATGTGTGCTTTGTGGTAAAGATGACAGACAATGGTAAAGCAAAGT

CAGGCTCAGGAGCAGCCTATACAATCAATCTAGAGACCTTCAGTAAGCGGCTTAAGGTGT

TTTATGACCATTGGAACGGAAACAAGTCTGATCTTTGGGCTTCTTCTGATGCCATTGCAA

TTGCTACTCCGCCTCCTTCTGAGGATCTCCGTTATTTGAAGTCCACAGCTCTGGATGTTT

GGTTACTTGGGTATGAATTTCCAGAAACCATAATTGTCTTCATGCAGAAGCAGATACATT

TCTTATGCAGCCAGAAGAAAGCAAATCTCATTGGAACCCTCAAGGATGCTGCAAGTGAGG

CTGTTGGTTCTGATATTATCTTGCATGTGAAAAGCAAGAATGGGGATGGCATTGACCTGA

TGGATGACATACTGCGTGCTGTTTCTGCTCAGTCGAAATCTGACACTCCAGTTGTTGGTC

ACATTGCAAAAGAGGCACCTGAGGGTAAGCTCCTTGA

>Traes_2BS_DD0D9084C

CAGCCGTGGGCGCCGCTCGAGGCCGCGGCGCGCGGCGTGCCTGCCGTCCACTTCAGCACC

TTCAGCGCCGCCGCTAAGGCATTCGTCGTCCACTGCCTCAAGAATGAACGGACCCCCAGC

GCCTTCCCGTTCGAGGCCATCAGCCTCGGCGGCGCCGAGGAGGACGCCAAATACGCGGCG

CAGCTCGTCTCCCGCGATGACGGCACAGCCCAGATCCCCGAGCGCGACCGCCTGCCGCTC

AGCCTGGAGCGCTCCTCCGGGTTCGT

>Traes_1AL_46245C5D5

ACCTTCGACACCGACTACTTCAAGCTGGTGAGCAAGCGGAGGGGCCTCTTCCACTCTGAC

GGCGCCCTACTCACCGACCCCTTCACCCGCGCCTACGTCCAGCGTCATGCCACCGGCGCC

TTCAAGGACGAGTTCTTCGCTGACTTCGCTGCCTCCATGATCAAGATGGGCAATGCCAAT

CCTCTCACCGGCAGCCAGGGCGAGATCAGGAAGAAGTGCA

>Traes_2DS_607E885CF

GCCACTGCCGCTGTCGCGAGCCGCCACCGGCGATTTCAGCCACTGCGGCGGCTGCGACGA

CGCCGACGAGGGCAGCCTGTGGAGCACCGACAACATCCTGCAGTGCCAGAAAGTCAGCGA

CTTCCTCATCGCCACCGCCTACTTCTCCATCCCGCTCGAGCTGCTCTACTTCACCACCTG

CTCCGACCTCTTCCCCCTCAAGTGGATCGTCCTGCAGTTCGGCGCCTTCATCGTCCTCTG

CG

>Traes_7BL_9868A774F

GTTCGGGAGCGGCAACAATGGCGGTTTCCACACGCATGGCACTCTTGATCGCAGTTTGCT

TCCTCGGTTGCTACGTGTTCGTCCCGTCCTTGGCTGCCTCCGGCGACTTCCTCCAGTGCC

TCTCGTCCAGCATCCCCAGCGAGCGCGTGTTCACGCCGGATTCGCCGTCGTTCACTTCCG

TCCTGGTGTCGTCCATCCGGAACCCCAAGTTCTTCACCCCGACGACGGTGAGGCCGCTCT

GCATCGTGACGCCCACCAACGCGTCCCACGTCCAGGCCGCGGTGCTCTGCGGCCGCCGCC

>Traes_2DS_A175D9393

CACACACACATTTCCCGGTCTCATCACTCACAGGCAGAGGCATACAATACAGGTCGCTGG

TATTTTTCTCCTTGTTCCTGAATCCTGAGTGAACACCGGTCTACCATGGGTCTCGTCTGG

ATGGTGGCGGCGGCCGTGGCGGTGGTGCTGGCCTCGTGGGCGTTCAACGCGCTGGTGTAC

CTCTTGTGGAGGCCGCACGCCATCACCAGGCAGCTCCGCGCGCAGGGCGTCGGCGGGCCG

GGCTACAGGTTCTTCGCCGGGAACCTCGCCGAGATCAAGCGGCTCCGCGCCGACACCGCC

GGCGCCGCGCTGGACGTCGGCAACCACGACTTCGTCCCCATGGTCCAGCCGTACTTCCGC

AAATGGATCCCAATCCACGGGCGCACGTTCCTCTACTGGTTCGGGGCCAGGCCGACCCTG

TGCGTGGCCGACGTGAACACGGTGAAGCAGGTGCTCTCCGACCGCAGCGGGCTGTACCCT

AAGAACATCAGCAACCCGCACATCGCCC

>Traes_3DL_BE597D771

CAAAACCAAACAGGTCTTACGCAACAAATGGAGAGCACCCAGAGCAACACAAGCGTGATG

AACCATTTCATCCTGGTGCACGGCCTCTGCCACGGGGCCTGGTGTTGGTACAAGGTGGTT

GCGGCGCTTCAGGCAGCGGGGCACCGTGTCACGGCGGTCGACCTGGCCGCGTCCGGCGCT

CACCCGGCGCGCATCGAC

>Traes_1DL_DD6CFF0F3

CGGTTTTGAAGAATCAACCAAACAACTAGTCCTTGGCACCGTTCTAGCAGAAGAGTCTCT

GCTATGCTCGTGTATAACTCAATATATACCTCGTGTGTTCTCCTCCGGAGTCTTAAAACC

GGGGCACCAAAACACAAGCTACGTACGTGCAAAGTTCAGTCAGACTTTGAGTTGACAGAC

ACCACACCAGCAATGGCCGGAGCAGTAGACGATCTGAAGCTGCTGGGCACGTGGTACAGC

CCGTACGTCCTGCGAGTGCGCCTTGCTCTCAGCATCAAGGGCATCAGTTACGAGTATGGG

GAGGAGGACCTCCGGCACAAGAGCGAGCTGCTCCTCCGGTC

>Traes_2DS_A28343728

GTCAAGTCAAACGCCCAACCCCAACTCCAACCCAAACCTCAACCAAATCCAAACTCCCAA

CAACCATGGCGACGACGGCGATCCCGTGCGTGACCCTGAACACGGGCCACGAGATGCCGG

TGCTGGGCTTCGGCACCGGCTCCTCGCGCGTGCCCGCCGACCTGCCGGACACCATCGTGC

ACGCCGTCCGCCTCGGCTAC

>Traes_2DL_BF4C00019

CTAACATCCTTAGCCTTCTTGCCGAGGTCCTGTCGGCCGTCTTCTGTGAGGTCATGAACG

GCAAGCCAGAGTACACTGACCACTTGACCCATAAGTTGAAGCACCACCCTGGGCAGATCG

AGGCAGCCGCCATCATGGAGCACATACTTGAAGGCAGCTCCTACATGATGCTTGCTAAGA

AGCTCGGCGAGCTCGACCCATTGATGAAGCCAAAGCAAGATAGGTATGCACTCCGCACGT

CACCGCAGTGGCTTGGTCCTCAGATTGAGGTCATTCGTGCTGCCACGAAGTCTATTGAGC

GC

>Traes_1AS_D91A7B6EC

ACTGAAGAAATGTATGTCTAACGTCTGAAAAATAAGTGCAGGTGGGTATAGCAGGAGTGG

TTGCAGACACAATGATGAAGGAGACCAAGGTGATCATGGACAACTGGATACCGACGTGGG

ACCACGAGTTCGAGTTCCCGCTGTCCGTGCCAGAGCTCGCGTTGCTGCGCGTCGAGGTGC

ACGAGTCCGACAACCACCAGAAGGACGACTTCGCCGGCCAGACCTGCCTGCCGGTGTGGG

AGCTCCGG

>Traes_2DS_7C31D694B

CTCTTGGATCGCGACATGAACCCCAAGATCTCGGACTTCGGTTTGGCGAGGATATTCTGC

TCCAATGTCACGGAGGCCAACACGACCAGGGTCGTCGGCACGCACGGTTACATCGCTCCA

GAGTATGCTTCAGAGGGTCTCTTCTCCACCAAATCCGACGTTTTCAGCTTTGGCGTCTTG

CTCCTGGAGATCATAAGTGGGAAGAGGACTGCAGGATTCTACCAGTATGGAAAATTCTTC

AATCTCACAGGATACGCTTACCAGTTGTGGCAAGAGGCGAAATGGCACGAGATGGTCGAT

CAGGTACTCGGGGTCGACTACCCGGTGACGGAGGTGATGAAGTGCGTCCAGGTGGCGTTG

TTGTGCGTCCAGGACAGCGCCGACGATCGACCCGGCATGTCCGATGTCGTCGCCATGCTC

AGCGGCGAGGGGCTCACGCTGCCGGAGCCCCGACAGCCGGCCTACTTCAACGTTAGGTTA

TCAAGCTTACCAGAGTCAAATAGTTCCTTTGGTGAATCGTCGTACATCAGCAACGTCGCC

TTGACTGATGAAGACGGCAGATAACAGGAGCAAGTGTAGGCGGCCAATCAATTTGTACTA

GCGAGGTAATTAACTATACATGGCTACTATTCTCATCAGATTTACCAAAAGGAAGGACCA

TAGTAGACTAACGTAGTTGATGCACATAAATCCCATGCCCATCAACATGTTGAAAGTTAC

TTTTTTTTTTGCATTCTCTCGTAAATTTATTTCTTTTATGTAACCTTAGGTGGTTTTTTG

CTACGTTGTAATATCTGTATTAGGACTG

>Traes_2DS_604BB66D2

CGCGACGATACATCGTGACAAGGAGGTATGGGGAGAAGATGCCAACGAATTCAAGCCAAT

GAGGTTCGAGAATGGAGTGACGAGGGCCGGAAAGCACCCCAATGCCTTGTTGTCTTTCTC

TAGTGGGCCGAGGTCATGCATAGGGCAGAATTTTGCAATGATCGAGGCCAAGGCCGTGAT

CGCCGTGATTCTTCAGAGGTTCTCGTTCTCCCTATCACCTAAGTATGTTCATGCCCCGAT

GGACGTGATCACGCTGCGGCCTAAGTTTGGGCTTCCTATGGTCCTCAAGAGCCTCGAGAT

GTAGAAAAATGTGTATGGTGTTAAGTATAGCACAGGGTTTTTGTATTACTTGACTGGAAC

TTAGCTTATGTTGCGCACTAGATAAGCTTGTAAAATCATCGATATATAATGTACCCTATT

TGTAAATATAAATAATAAAGTGTTTTTATTAATAAAT

>Traes_1BL_B312AA1F1

TAATGCTGCTCGATCATGGTTTCTCCAGAGCAAGGGTCCATTCTGGACAGTTCCTCTCGG

CCGCCGAGACGGCAGCGTGTCCATTTCCAACGAGACCGACGCTCTGCCACCCCCGACCTC

CAACTTCACCGTGCTCACCCAGCTCTTCGCCGCCGTGAACCTCGACGCAAAGGACCTTGT

CGTCTTGTCCGCCGGGCACACCATCGGGACGTC

>Traes_6BL_E54F43BD8

CACCGCGAGTGCTCCATGTCCAACAAGGAGCTCTTCACGCGGCTCTGCAGCCGCCCGGTC

CTCGACGGCGCCACCATGAGCTTCGGGCTGGACAGGCGGAGCGGCGCCAAATGCTGGATG

CTGTCGGCCAGGGCACTGAGCATCGTGTGGGGCGACGACCCCTCCTGCTGGACATGGACT

GCCGACCTCCCCGGATCACGGTTTCCTGAGGTGGCCGAGCTTGTGGACGTGTGCTGGCTA

GAGATCACCGGGAAGCTGCAGCTCTCCCTGCTCACCCCACGGACCACCTACG

>Traes_2DS_52FEAC922

GCGCTCTTGAACGCCACCGTGAGCTACGCCGTCACCAACTCCACGCGGCTGTATGCGTCC

GGGCAGGCCGACTTCGACCGGGAGCTCCCCAAGGTGTACGCCTGGGCGCAGTGCACACCT

GACCTGACGCCGGCGCGGTGCCGGGACTGCCTCGCCGTGAACATCAAGACATGGGCGCCT

GTGTTCACGGACTCCATCGGGGCCAGGATTCTTGGGATGAGGTGCAGCTACCGGTACGAA

ACAACGCCCTTCTTTGATGGCCCGGTGATGGTGCGGCTGGCAGGAACGTCGGCCAACTCT

GCTGCACCGGCGTCGGCGCCTGCTGTGGTGCCCAACGTTTTGACGCCAGCGGCGGCGGCC

GGAGAAGGGAGAAAGTACAGTGTTCCTGGCATGGTTCTCATAGTTCTTCTTCCTACCGCA

GCAGCCATAAACCTTGTCGTTTGCTTCCTTCTCTGGAGGA

>Traes_2DS_B38ADE490

AAAGCTTTTGAGCTTCTTGTTGCATCACAACCCTCCTGGTGATGCACCAGCAGTTCTTCC

AATCATAGGTGGTGTCAGGGTTGGGAAGAAAACTTTGGTTGCTCATGTGTGTGGTGATGA

AAGGGTTCGCTCGTGCTTCTCTTCTGTTTTGCACCTGAATGGGGATAGCCTCTTGAGAGT

ATTTGACCATGGAAGTACCACGTTTGGGGTGAAGATGTTGGTAGTTATT

>Traes_6AL_BD173107E

CGTGCCGGCGTTGAGGAGCTTCTCCTCGAGCGGTGACGGCGAGCCGTCGCAGCTGCAGCG

GATGGGTGCCGGGCGGCTCCTCATGGCGCTCACCATGGCGGTGGCAGCGCTCGTGGAAAT

GAAGCGGCTCGACAGCGCGGCGCGTGGGGAAGAAATCAGCATTTCGTGGCAGCTGCCCCA

GTACTTCTTCCTTGCCGGCGGGGAGGTCTTCTGCTACATCGCGCAGCTGGAGTTCTTCTT

TGACGAGGC

>Traes_7DL_D26D7E5CB

GTTTGCACAATCCACCAGCCAAGCATTGACATCTTTGAAGCTTTTGATGAGTTGCTATTA

CTGAAAAGAGGAGGCCAGGTGATATACTCTGGGAAACTGGGTCGCAACTCACAGAAAATG

ATTGAATACTTCCAGGCGATTCCTGGAGTGCCTAAAATCAAAGATAAGTACAACCCTGCA

ACGTGGATGCTTGAGGTCAGTTCAGTTGCTGCAGAAGTACGCCTGAGTATGGATTTTGCT

GACTACTACAAGACTTCAGATCTGTACAAGCAAAACAAGGTATTGGTGAGCCAGCTAAGT

CAACCAGAACCAGGAACATCAGATCTATATTTTCCTACAGAATACTCTCAATCCATTATA

GGGCAGTTCAAAGCCTGCCTCTGGAAGCATTGGCTGACCTATTGGCGCAGCCCAGATTAC

AACCTTGTCAGATTTTCCTTCACTTTGTTCACAGCCTTGCTGCTGGGCTCCATCTTTTGG

AAGATTGGCACCAATATGGGAGATGCCAATACTCTTAGAATGGTCATTGGAGCAATGTAC

ACAGCAGTGATGTTTGTTGGCATCAACAACTGTGCAACCGTTCAACCAATTGTTTCAATC

GAGAGAACAGTTTTCTACCGAGAGAGGGCTGCTGGGATGTACTCTGCACTGCCATATGCC

ATTGCTCAGGTTGTCATGGAGATACCCTATGTCTTCGTCCAAACCTCGTATTACACCCTC

ATCGTATACGCCATGATGAGCTTCCAGTGGACAGCTGCCAAGTTCTTCTGGTTCTTCTTC

GTCTCCTACTTTTCCTTCCTCTACTTCACCTACTACGGCATGATGACTGTATCAATCTCA

CCAAACCATGAGGTTGCAGGCATCTTCGCCGCAGCTTTCTATTCCCTATTCAACCTTTTC

TCTGGCTTCTTCATCCCAAGACCGAAAATCCCCAAGTGGTGGATCTGGTACTACTGGATT

TGCCCGTTGGCATGGACAGTGTATGGGCTCATAGTGACACAATACGGAGACATGGAAGAC

ATCATCACCGTCCCAGGCCAACCTAACCAGACAATCAGCTACTACATAACTCATCATTTT

GGATACCACAGGAGCTTCATGGCGGTCGTTGCACCGGTGCTTGTGCTCT

>Traes_5DL_C7E27D4F6

CCGGCCGCGATTCTGTCGTCTGGAAACGCATTGATTCGGCGATGACCAAGCACCACCAGC

ACCTGGGTTTAAGCTTCCGCCATGGCGCCGAGGAGGAGCGGTGGGTCGACGACTCCTCCG

TCNNNNNNNNNNNNNNNNNNCCCGCCCCTCCGCGCCGCCACCGGCTCCTGGAAGGCCGCC

ATGTTCATCATCCTGATCGAGTTCAGCGAGCGGCTGAGCTACTTCGGCATCGCCACCAGC

CTCATGATCTACCTCACCAAGGTGCTGCACCAGGACATGAAGGTCGCCGCCGAGAACTCC

AACTACTGGATGAGCGTGACCACGCT

>Traes_4BL_5528F3966

CAAGGAGCTGGGCGACGACGACGCGCGGGTGGTGCATGTGGGCGACGCGGAGCGCACCAA

CGAGCGCCTCCAGTTCGCCGGGAACGCCGTGCGCACCGCCAAGTACTCGCCGCTCACCTT

CCTGCCGCGGAACCTGTTCGAGCAGTTCCACCGCCTCGCCTACGTCTATTTCCTCGTCAT

CGCCGTGCTCAACCAGCTCCCCCAGCTCGCGGTCTTCGGCCGCGGCGCCTCCGTCATGCC

GCTCGCCTTCGTCCTCGCCGTCACCGCCGTCAAGGACGCGTACGAG

>Traes_6BL_D93192C47

TGTCCGAATTGTATCTAGCCTGAATGTTGCTGAATGCTGCTGCTTATCTAACTGCAACTG

CATTTGATCTGATTGTGCTGTTGCTTTATGGTCTCATGCGTTTGATTGCAATTAATCCGA

CGATGTTAATTGGTGCAACCTGCAACTGTAAAAAAGTGGTAGGATGGTAGATCAAAATAC

CTTTGCTGTGAACCAAAGTGGTAGGATGCTAGATCAAACTACCTTTGCTGTGAACCAAAA

GCTCAGAATAGGGATTTCTGCATGGTTCTGACTAGCAGCAAATTTTGTGATAAAACATGC

TTGAATCAGTTGCTGAATATCTCGAAACTGTTTGGAAGAGGTTAAGTTGAAGTCAGTTTC

ATTTGGAGAGTGGAGTACCGCTGTACATGCCAAGTCCTTGTTGCTTCACACATAAGCAGA

ACTGAGTGCTACTTTTATCTTCCTTGCTGTCAAGAGCAAAGGGCATGCCCTTTACAACTT

TGTTCTTTCTCAACTATGCACAGCACATAGATGACACTTTGCATAAACAATGTGATTTCT

ATGATCTACCTTGTTCACAGTAGGCAATACTTTGGTTAATACCAAGTTACAGAATCTTGC

TGCAATGCTAGATCGATTTCAGCAACTTATGCTGTTGATTGGTTTTTGCCTTCATTACGG

AACTTTGCTGCAATGCTAGGTTGTTTTCTGCAACTTACGCTGTTTGATTGGTTTTTGTCT

TAGTTACAAATCCTGACCCTATGTTTGCGACAGGTTCTCCGAAGGTGCTCAACTCATGCA

TGTTTGCTGGTTCGAGATCCGCGGGAAGATACATAGCAATATGCTCTCCCAGGACACAAC

GTATGCCGCCTACATG

>Traes_2DL_E372BFAAB

CGCCGGCGTCGGTGTTGTACGTATCGTTCGGCACCACGTCCTCGCTGCTCGCCGAGCAAG

TCGCGGAGATCGCCGCGGCGCTGCGTGACAGCAAGCAGCGGTTCATCTGGGTGCTGCGAG

ACGCCGACCGCGGCGTCAACGACGTACACGAGGAAAGCACTGACGGCCAACACGCCAAGT

TGCTGTCGAAATTTACCAGGCAAACCGAGGGGACGGGGCTCGTGATCACCGGATGGA

>Traes_7BL_0E12F86E9

CTTCCATACATACCTTGCAAACAGCAATCAACCACTAGAACACAACAAACACACCAACTG

CCGAAAGAGATCATCCATTAGCAATGGCGGAGTTCGCGCTTGGTTTAACCAAGACGGCGG

TGGAGGGGACGGTGAGCAGGGTGAAGTCGGCGATCGATGAGGAGGCGAACCTCAAGGTCA

GGGTGCAGAATGATCTGGTATTCATCACCGGGGAATTCCAGATGATGCAATCATTCCTTA

ACATCACCAACAAGGAGCGCACCAACAATGAAGTGGTGAGGACATGGGTGAGGCAGATCC

GTGAC

>Traes_5DL_000FCF98B

GAGCGGCGCCGCCTCGACTCCAAGGTCACCATGTCGGTGTTCTGGCTGGTGCCGCAGTTC

GCGCTGATGGGCGTGGGCGACGGGTTCGCGCTGGTGGGGCTCCAGGAGTACTTCTACGAC

CAGATGCCGGAGACGATGCGCAGCCTGGGCATCGGGCTGTACCTGAGCGTGATCGGCGCC

GGGAGCTTCCTGAGCAGCCAGTTGATCGCGGCGGCGAGCCGCCTGAGCTCGCACGGCGGG

NGAAGGCCGGCAGGGCCGGCGCCGACAAGGACGGTTCCGGCATCGATAGACCGTACAATC

ACAAGTGCGTACTACAGGGGAGCGCTTTTGGTGTATGACATAACGAAGAAGCATCAACAT

CGTCAACATTTTAACCACCAGAGGAAGATGGCCAGGCATTAGCAGAGACGGAAGCCTTGA

ACGTTGATGAGAAGGCGTTTCAGACCATTCTTTCCTACATCCATCAGATCATAAGCAAGA

AGGCGCTCCCTGCCCATGGCCTCGGACTCAAGGAACCACTATCAATGTTGCCGATTCATC

CTGCAACACCAAGAAAGGGTGCTGTTCAACCTAGGTGTGAACTGAGAAGTGGTGATAGAG

CATCTCTAACTCATCCCCTGTAAAATTAGAGCCCTAAAATTTAG

>Traes_7BS_85034CAEC

CATTTGGCATGGAATATTTGCATGGGAAGAACATTGTGCACTTCGACTTGAAGAGTGATA

ATCTGCTCGTCAACCTAAGAGATCCCCAACGCCCTATATGCAAGGTCGGTGATTTGGGCT

TATCAAAGGTTAAATGCCAGACACTAATCTCTGGTGGGGTGCGAGGGACACTTCCCTGGA

TGGCTCCTGAGCTGTTAAATGGCAGCAGTAACCTTGTTTCTGAAAAGGTCGATGTCTTCT

CATTCGGAATAGTGATGTGGGAACTTCTTACTGGTGAAGAGCCTTATGCTGACTTGCATT

ACGGCGCCATCATAGGTGGGATCGTGAACAACACCCTGCGGCCTCTGGTGCCCG

>Traes_7DL_C4602D44B

CAAGAACCCGAGGGGTGCGTACGTGAACTACCGGGACCTGGACATCGGGGAGAACGCGGT

GGTCGGCGGTGTCACGAGCTACGAAAGCGGCAAGGTGTGGGGTGAGAGTTACTTCGGGGG

CAACTTCGAGAGGCTCGCCATCACCAAAGGGAAGGTGGACGCCGGCGACTACTTCAGAAA

CGAGCAGAGCGTTCCCCCACTT

>Traes_2DL_798325B41

CTTCCGTTATATTCAGGGTAAAAGAAAACTCGTTGCTTCTGATCACGGCTGACGTGCTCT

GCTTTGAACTTGTTTTTTTTTCTTGTTTGACAACAGACAACTGGCTCCCTCTATTTCTGG

GCTGCTCATCTCGCTGGCCCGGTCTGGGGTCCATTGGCATCCTGGTGCTGCGCTTGGCTG

GAGGCCATAGGCCTCATTGCCGGAATTGGTACACAGGCCTATGCAGGATCCCAGGTGCTG

CAGAGCATAATCCTGCTCTGCACCGGCACCAACAAAGGCGGCGGCTACCTGGCCCCTCGC

TGGCTGTTCCTCGTCATGTACCTCGGGTTAACCTTCATCTGGGCCGTGTTCAACACTTTC

GCCTTGGAAGTCATCGCATTCCTCGACGTCATCTCCATGTGGTGGCAGGTGGTTGGCGGC

ACGGTCATAGTGATCATGCTGCCGCTGGTGGCGAAGACCACGCAGCCGGCGTCGTACGTG

TTCACGCATTTCGAGACGACGCCGGAGGGGACGGGGATCAG

>Traes_2DS_52DFCEB72

TACCGCAATGGGCGGACCAACCGACCGCTGCAAAGTATGTGGAGAGCGCGTGGGGAATCG

GCCTGCGTGCGCGTCGAGACCAGAAAGGGTTGGTGAGAAGGGAAGAGATGGAGAGGTGCA

TCAAGGAGGTGATGAGTGGAGAAGAGTATAAGAGAAATTCTTCTAAGTGGATGCAGAAGG

CCAAAGAGGCTATGCAGGAAGGAGGG

>Traes_2DL_661DC4EF0

GCAAGTCCGGCAGTGACTGGTTCTTGGCGCCGGCCGGTGGATCCGATGGAGTGAGATCCG

GATCGTGGCGCTGCCGTCCTCCACCGCTTTGTCGGTGAAGGAGGAAGTCGTGCTCTCGTT

TGTGTTTTTGGAGTGGTTTTTATATTTGTTGTGGTTATAGTTATGAATACGTACATACAA

GTGGAGAGTGTAGATTGGAAGTGAGAGAAGTCAAATTTCTTAGAACAGAGAGATGGTTGG

TGGAGGCTGGTTCACCGGACGGTTGGTGTCTTATGGCTGTCCTTTATCTGTACACACAAC

TGTCGGACTGGTCCTTTTGTTCTCACTTTATTATGTTGTTATATGATGTAAGCCATGCCT

TTGTTTTTTTATGTTGGCAACTAAATTTACTTGCCACCTTATTAGGTTGGAGAACTCAAG

AAGCTTGTTGAAGAAGGAAAAATAAAATACATCGGATTATCTGAAGCATCTGCATCAACA

ATCAGGAGGGCTCATGCTATTCATCCTATCACTGCAGTTCAGATGGAGTGGTCACTGTGG

TCAAGAGATGTCGAAGAAGACATAATTCCAACTTGCAGAGAACTTGGAATTGGAATTGTT

GCTTACAGCCCACTTGGTAGAGGATTCTTCTCAAAAGGAGCAAAATTGGTTGACTCACTA

TCAGACCAGGACTTCCGCAAGGTGACTTCCCA

>Traes_5BL_DAC1B7D86

CGACGTGCCAAGCGGCACGATGCTGATCGTGAACGCGTACGCCATCCACAGGGACCCGGC

CGTGTGGGAGGACCCGGCGGCGTTCCGGCCGGAGCGGTTCGAGGACGGCAAGGCCGACGG

GCTGCTGCTGATGCCGTTCGGGATGGGGCGGCGGAGGTGCCCCGGCGAGACGCTGGCGCT

GCAGACGGTCGGGNNNNNNNNNNNNNNNNNNNNNNNNNNNNNNNNCGACTGGGATCGGGT

GGACGGCGCGGAGGTGGACATGACGGAGGGGGTGGGGATCACCATGCCCAAGGCCGTGGC

TTTGGAGGCCGTGTGTAGGCCTCGCGCGGCCATGGGCGATGTCCTTCAGAAGCTCTGAGG

ATCGAACTTGTTGGCATCCTATATGTACAGTAGTTTCCAGTAGCATCGTCCCGTATGCAT

GGCCCGATACCGTTTTGATGTAGTACTGTAGTACCGAATAAACTTTTTTTTAACTACTTG

GCGCACTTTATTAAACTCATATCAAAGTTACATCATTCACAATGTCCGAATAAACTTGCT

GCGACTTCAGACCATAATAACCTTGCCATCATCTGTGATTTCTTGCACAAGCTAGGGTCT

TTGTAGTCTATCATAATGATTATCCCTAGGGCAACTCCAATAGCACGACACATTTGATCC

GCACATGTCTCGACAGAAACGCC

>Traes_2DS_B44C1979E

ATGCATGATTATTTTACGGAGAATCCTACATATCCTGCACACCTCTTCCGCATAAGGTAC

CGAATGCGCCGATCCCTCTTTGTGAAACTTGTTGAAGCTTGCGAGGCAAATTGCCGGTAT

TTTACTCAAAGAAGGAATGCCGCAGGCTTAAAGGGATTTAGTGCATATCAAAAAATCTCC

GCAGCTATGCGGGTGATTGCATATGGCGTT

>Traes_3AS_C6A90DC36

CGGCGACCCTATAAATACCTGGCCTCCATCGCCCTCCACCCCAACTCAAGCCAGTGTCAA

CCAAAAGTAGACAGCATCCTACCTGCGACCCAATCCAGAACAGATTTTTTCCCCGAGCTA

GAAACCAAAGTAACACCGACGATGTCGATCGTGCGGCGGAGCAGCGTGTTCGACCCCTTC

GCCGACCTCTGGGCTGACCCTTTCGACACCTTCCGCTCCATCATCCCGGCGATCTCAGGC

GGCAACAGCGAGACGGCCGCGT

>Traes_2DS_3091F29FD

GTCGGACGCCTTCAGCTTCGGCGTTCTGCTACTGGAGATCGTCACGGGGAGGAAGAACAG

CAGCTTCGCTAGCCCGGACTCGGAGCCATCCCTTGACCTCTTAAGCCTCGTATGGGAGCA

CTGGACTACCGGGACAGTCGAGGAGCTGGTGGATCCGTCCCTGGGAGGCCGTTCGCCGGG

AGGCCAGATGCTGAAGCTGGTCAACATCGGGTTGCTGTGCGTCCAGGACAGCCCCGCGGA

CCGGCCCACGATGTCGGCGGTGAACGTCATGCTCAGCAGCAGCACGGTGTCTCTGCAGGC

GCCGTCGAGGCCGACGTTCTGCGTCGACGACATGGAGGGTTTCTCGGGCATGTACTCGGG

TGCGTATCCAAGGGGATCCCAGTCCACCGGGGACAGCAAGCCCCAGGCAGCGATGTCGCC

GTCGCCGAACGAGGTGTCACTCACGGAGATCGAACCAAGATGATGAGCGCCTAGGCCCAA

GATAAACTTGCGGTGTCCATCGCATATATAGTCCTGAACCTGCTTGTAATAACTAAATCT

CCTGGATCATTGTCTATGGTGGTTGTAGCTTGCAATAATGTGTCACGGCTTACTATTACT

AGTATTTTTATCAGAGAATGTTGGTAGTAATATATTTTCTTTGTTCTAATCAGTCACTTA

GGGACTGTACTTGCTTCAGGGACAGCAAGACCACTTCATAGCAAATTGAGGTTCAGTTTT

GACATTTGTTGTACTACTAAACCTGGCAATCAAAC

>Traes_1DS_99E3E8761

ACGGCCGCTACCGCAGCGCGTGGCACCGCGTCCTGCCCATGCGCAACGGCAACCGCCGCT

CCATCGCGTCTTTCTACAACCCGGCGTTCGAGGCGGCCATCTCGCCGGCGGTGGGCGAAG

GCGCCGCCGCCGCGTACCCGGACTACGTGTTCGGGGATTACATGGACGTGTACAACAAGC

AGAAGTTCGATGCCAAGGAGCCAAGGTTCGAGGCCGTCAAGGCGCCAAAGTCAGCTTAAG

GCTTAAGCTCAATCAAGGAGATTAAAAAGGAAATTATTTACCTATTAGCTGGCTTTTGTT

TTACAATGAAGAAAAAAAATGTATTTTTGAGTTTGCTCAGACGCGTAGCAGTATGATGTG

GGGATACTTGTGATTGGTTCGGTGTCAAATAATTAGGAAATGAAATATGGATTTTTCGTT

GGAAGTATCTGCTTTCGTTACATTTGTTTGATTGTGCTTTATCATTTGATCAGTACATCA

TTCCACGAATGTTTCCTTGTAAAAGAAAATTTTGTAGTTATGACAAGAAACAAAAGATCT

CCACAAACAGTAAAGTACATCTCACAATTAATCTAACA

>Traes_2DL_A48D63814

TCGGGCTGCACTGCTTGGCCGTGTCCATGCTCGTTGGAAGAATGGATGCCAATCACCGGC

TACTGCGAGAGAACGGCCTCGTCTACAGCGCCATCGAGGAATGTGCGACCAAGGAGTTCG

TGGAGTACGCCAACCGGGCCAGACCGACGAAAGACATCTCGCCCGGCGCGGGCATCCT

>Traes_2DL_849039EC1

CAAGGAAAGGATGCACACCATCACAACTTGCCTTGGCTTGGGTTCACCATCAGGGAAGCG

ATGTTTGCCCCATACCTGGGACAACAAAAATTGAAAATTTCAACCAAAATATGGGAGCAT

TAGCTGTGAGGCTCACACCAGAGGAAATGGCTGAACTCGAGTCTTACGCGGCTGCAGGTG

ATGTCCAGGGTGACAGGTACC

>Traes_5DL_50E35B4F3

ATGGGAGCTGGGATGAAGCGCGCGAGGGAGGAGGAACCAACCGTGCCGCTGGCGCTGTCC

CTCAGCACGGACTCGGCGTCTTCCGCCGTGACGTCGGCGGACTCGGGCGCCGCTGGGCCG

GCCAAGCGCAGGAGGGGCGTCGTGGTGGCGACGTCGGGGGAGGGGGAGTTCGTGTGCAAG

ACTTGCGGGCGGGCCTTCGCGTCGTTCCAGGCGCTGGGCGGGCACCGGACCAGCCACCTC

CGGGGCCGCCACGGGCTGGAGCTCGGCGTCGGCGTCGCCAGAGCCATCAAGGAACGGAAA

AAGCGTGACGAGAAGCAGCACGAGTGCCACATCTGCGGGCTGGGTTTCAAGATGGGACAG

GCGCTCGGGGGCCACATGAGGCGGCACCGTGAGGAGATTGCACTGCGCAGCGCCGACGAT

CGCTGGGTCGCGCTGCTGCCGGATCAAAAGGTGGAGGGGCAGCAGGCTGCCGCCGACCGG

CCGCCGCCCGTCTTGCTCGAGCTGTTCGTCTAG

>Traes_2DL_27AAEFE5E

CTGGGAAGGGTTATCAGATAATTCGCAGACTTGTTTCAGCTTGCTTGTTGCAGGTCAGTG

GCTCAATGTCTTCAGAGGTAAAAATGCACCATACAATTAGGCAACTGGGGTTTTGGTTGG

TCAACAAGTCAGATGCAAAATTTCTTGTTCAGCCAGGGATGGCCTTGGATAATGCTCCAT

CAGCTGGAGAATGGAAAGAAGCTACCAGGATCTCCATCATGTCTAATAACATTACAGAGC

TTTCTTTCTCGCCGAAGTGCAAAAACGTCACTACATTGTTGATCCAGAACAACCCAAATT

TGAACAAGATGAGCAATGGATTTTTTAGAACTATGGCGACCTTGAAAGTGCTCGATCTTT

CTCATACTGCAATAACATCACTTCCAGATTGTAAGACATTGGTTGCATTGGAGCATCTGA

ATTTGTCTCACACACACATTGTGAGGTTACCTGAGCGCCTA

>Traes_4BL_B288394D4

TGGTCCCCGGCGCCGGCGACAAGCCGATGATCGTGGTCACCTACAAGGGGGAGGAGAAGA

CCTTCTCTGCCGAGGAGATATCCTCCATGGTGCTCACCAAGATGAGGGAGATCGCCGAGG

CCTTCCTCAGCACAACCATCAACAACGCCGTCGTCACTGTCCCGGCCTACTTCAACGACT

CCCAGCGCCAGGCCACCAAGGACGCCGGCGTCATCGCCGGCCTCAACGTCATGCGCATCA

TCAACGAGCCGACCGCGGCGGCCATCGCTTACGGCCTCGACAAAAAGGCCACCAGCACCG

GGGAGAAGAACGTGCTCATCTTCGACCTCGGCGGCGGCACCTTCGATGTGTCCATCCTCA

CCATCGAGGAAGGCATATTCGAGGTCAAGTCCACCGCCGGGGACACCCACCTGGGGGGCG

AGGACTTCGACAACCGGATGGTGAACCACTTCGTGCAAGAGTTCAAGAGGAAGAACAAGA

AGGACATCAGCGGCAACCCGAGGGCGCTCCGGCGGCTGAGGACGGCGTGCGAGAGGGCCA

AGAGGACGCTCTCTTCCACCGCCCAGACCACCATTGAGATTGACTCGCTCTACGAGGGGA

TTGACTTCTACGCGACCATCACCCGTGNNNNNGCATGGAGCCCGTCGAGAAGTGCCTCCG

GGACGCCAAGATGGACAAGACCCAAATCCACGACATCGTGCTCGTCGGAGGCTCCACCCG

GATCCCCAAGGTGCAGCAGCTCCTCCAGGACTTCTTCAACGGGAAGGAGCTCTGCAAGAG

CATCAACCCCGACGAGGCCGTCGCGTACGGCGCCGCCGTGCAGGCTGCCATCCTCAGCGG

CGAGGGCAATCAGAAGGTGCAGGACCTGCTCCTGCTCGATGTGACGCCGCTCTCGCTCGG

GTTGGAGACGGCCGGAGGCGTGATGACTACTCTGATCCC

>Traes_4DL_2AB0556C6

GGCGCCGGGGTGCTGCCGTACGAACTGCTGGCCCCCAGCTCGCCGCCTGGGGTCACCTGC

CGCGGCGTGCCCAACAGCATCTCCATATGATACGCACGCCATTGTATACCGGCGATTCGT

TCTGGATTTTGTTTAGGGGATTCCCCTTATTTAGGGCCAGCTTGTCGATCTACTAGTTTT

GATCTGGATGTTAGTCTCGTGGTCTCCCAAATCCAAATTCAGACCAATCTAAGGCCGCAT

TTGACCTAGAATGGCCGGCATGGGCGCCCGCGTCGGAGCTACGCGACTGGAGTCAGCACC

CCGACCAGACCCACCTCCTGTTCGTCTCCAAGCAC

>Traes_3AL_298EDEEF0

GTCACGGAGAAGGACTCCCGCATCCACAACTACGGTGTCACGGAGGAAGACTGCCTGGCT

CACTTGGACATTGGGTTCAAGTTTCACGACCTCACCGACGATGTGCACGGTGTCCTTGGT

CAAACGTACCGCTCCGACTATATTAACAAGCTCAGGGTGAGCGCCAGCATGCCGGTGATG

GGCGGCATAACCAGCTATGTATCATCGGATATCTTTGCCACTGATTGCGCAGTTGCTAGA

TTTGGTCGCCGTACTAGCATCTCAATGGTTGCATCAAGTGATAGTTGAAGTACTTTTTCG

ACCTGAATAATTGAGTTCATGTACAAAAGTGTCATATAAATAAGGCACATGGG

>Traes_4BL_1B8F5525D

TTCGGCCGGTGGTATGAATTCCGTTGGGTTTCTGATTATGGCTTGCATTCTTACGGATGG

ATGGTCGCAGGGATCGAAGCCATGGAGGTTCACGGGCAAAGAGGAGAACATGGACAGGGA

GGACATCAGGATCCTCGTCAGGAACTGGTGGGATATCTACAACGACGAGAGCCTCGATTT

CAAGGGCCTGGCCGCCGACGCCGACGAGCTCGAGGCGGCAGC

>Traes_3AL_C9606FE83

CCTACATGTTCTACGCGTCCGCCGCCGCCGACCTCGCAATCTACCTCCAGGTTCCTGATG

TCTTCCGCGCGGCGCCTTTCTCGTTCAAGGATATGGGAGACGCGGCGCTGCCCTTCTCCG

GCGTGCCGCCGGTTCGCGCTCTGGACATGCCGGACACCATGTCAGACCGGGAGAGCGACC

TGTGCAAGAGAAGAGTGCAGCAGCTCGCGCGGATGCCGGAAGCGAGA

>Traes_2DL_0D8943F9D

TCAGAGTTCACCAAGCACACGGAAGGGAGGGGGCTGGTGATCACCGGGTGGGCGCCGCAG

CTGGAGATCCTAGCACACAGCGCCACGGCCGCGTTCATGAGCCACTGCGGCTGGAACTCG

ACCATGGAGAGCCTGAGCCACGGCAAGCCGATTCTGGCATGGCCCATGCACTGCGACCAG

CCGTGGGACGCGGAGCTTATCTGCAACTACCTCAAGGCAGGCATCCTCGTGCGCCCATGG

GAGAAGCACAGTGAGGTGGTCACGGCGAAGGCCATCCAGGAAGTCATCG

>Traes_4BL_45E6F3783

CCCTCGTTCCCGCGCATCAGCTCCGAGACCGCGGCCTTCGCCGGCGCGCGGATCGACTGG

AAGGAGACGCCCGAGGCGCACGTGTTCAAGGCGGACGTGCCGGGGCTGAAGAAGGAGGAA

GTGGAGGACGGCAACGTGCTCCAGATCAGCGGCGAGCGCAACAAAGAGAAGGAGGAGAAG

ACCGACACGTGGCACAGGGTGGAGCGCAGCAGCGGCAAGTTCCTGCGCAGGTTCAGGCTC

CCGGAGGACGCCAAGGTGGAGCAGATCAAGGCGGCCATGGAGAACGGCGTGCTCACCGTC

ACCGTGGCCAAGGAGGAGGCCAAGAAGCCCG

>Traes_2DL_2050A1ADC

GTGGCAAGGCTATTCATGGTGGAAACTTCCAGGGCACACCAATCGGTGTGTCTATGGACA

ACACCAGGCTTGCCATTGCCGCGATTGGCAAGCTCATGTTTGCCCAATTCTCAGAGTTGG

TGAACGACTTCTACAACAACGGTCTTCCTTCCAACCTCTCCGGTGGGCGCAACCCAAGCT

TGGACTATGGCTTCAAGGGTGCCGAGATTGCCATGGCCTCGTACTGCT

>Traes_2DL_EBA70490E

CGGGTTGTCGGAGGCCTCGGCAGCGACAATAAGAAGGGCACACGCAGTTCATCCCATCAC

CGCCGTTCAGCTGGAGTGGTCTCTATGGTCAAGAGATGTTGAAGAAGATATAATCCCAAC

TTGCAGGTTTTTGCAACGACCACAGACCTACCTTTTTCTTTTCACAGTTTGTTTTCAAAA

TCAGGTTCATAGTACTAACGAAACGTCCTCAAAAAGAACTTAAAAAATATTTTCGGTTTC

AAAGAACTTGGCATTGGAATTGTGGCGTACAGTCCACTAGGCAGAGGTTTTCTATCTACT

GGACCTAAACTGGTGGACACATTACCAGAGGACGATTTCCGCAAGAATCTCCCAAGATTT

CAACCCGAGAA

>Traes_1BL_5E29B7C4A

CCTTAAGTAGTATTTCAAGCACGTAAAAATGTGTAATAAAGTAGGGGGCACTGTAGGTTT

GATAGGGGCATTTTGATTTTTTTTTGGGTATTTTGGGAGAGATCTTGTGAATAAGACTTT

AGAAGTACCAAGTATTGTGAAACAGACAGTTTATAAGTCGAACCTATTATTACCGTTCCA

TTCAACACTTATGTTTTGGCCGGTGATGAATCAAACAGTTCCCATTTCAACATTGACATT

CATCTAACTGTTATTTTCTTTCAGGGGAAGCTTATATAAGATTATTCATCGCCCTAATTG

CCAAATCGACGAGAAGCGTAGGATTAAAATGGCCCTTGATGTGGCCAGAGGCATGAATTG

TCTTCATACCAGTGTGCCAACAATTGTTCACCGGGATCTAAAATCACCAAACTTGCTGGT

TGACGATAATTGGACTGTGAAGGTCTGTGATTTCGGACTTTCACGTCTGAAGCACAGTAC

ATTTTTGTCATCAAAATCCACTGCCGGGACTCCTGAGTGGATGGCACCAGAGGTTTTGCG

GAATGAGCAATCCAATGAGAAGTAAGTGATCTTTGAGGTCTTTCTCAAATACTTACATTT

GCGAGCTTTTAATATTTTCACAAATTCCGTATTCATTGCTTGAGTTTAGCCTAGATAAAA

TCTCAGTTCGACCTAAATAAGCTTAATGCTAAATTCTGTATGTGCTATGTATAGCCTTTG

TTGGTCAACTGCTGGTCATGTTCATACCTGTGATTTGTCATGCAAGTACATTGCTATCTT

TCTGAAACATGCTATTTGTAGCTCCTCAAGATATTCCAAATTCCACAACTCCGCATGTCT

AAGTCTCTC

>Traes_4BL_9CF08143F

CCGCAGGATTTGGTGTCCGGCGGCGCGGCGCCGTGGCGGCCCGCATGTCGCCCTGCGCGC

CGGCGGCCGTGCGGATCGGCAGGAAGAGCCCGTTTCTTGGCGCCAGGCTCACGGTCGGTC

CCAGGAGATCGAAGCTCGTTCCTCGGAATCTAGTCGCCTCACCGGTGCAGGTAAATTTAA

TTTTCCGCCTCACCGATTCTCTTTTGATCGTCGGTGTCCTCTTTTTTTTTTCTTATATAT

ATATATAATGCTATATATTTTCCCTGACTTAACCTGTTCTCCGCGATAAATTCGCCGATA

TAGTCATTCTATCCGAATCTCATCCGCATGCAGAGTTGAACACTTATATTTGACTTTCTG

CAATTTTGC

>Traes_5BL_873CE02D7

GAAGGGATCACTTTTGTATCCTAGCTAGTGCTGTTACTGTAAAGGTGACAACATCTCGAC

TAATACTGAAGCACCTTTTCATTTTTACTACAGACAAACACTGAAGCATCTATCAAAGCA

TCCACAGTAGGCGAGGCTATTCTTAAATCCAAAGGCTTGTTTACTGGAGATTGGGGATTT

TGGATTTCCATGGGAGCCATCCTAGGGTTCACTATTTTGTTCAACATATTGTACATTCTG

GCACTTACGTACTTGAGCCCTCCCAGCGGCTCAAACACAGTTTCAGACAAGGAGAATGAG

AATGTGACAAACACTTCAACACCGATGGGTACCAATATTAATGAAGCCACAAACCGACCA

ACTCAGACTCAAATCACCTTGCCTTTCCAGCCTCTTGCACTATCTTTCAACCATGTAAAC

TATTACGTGGACATGCCTGCAGAAATGAGGGAGCAAGGATTCGCCGAAAGTCGTCTCCAG

TTGCTCTCTGATATCAGTGGTGCTTTTAGGCCAGGTGTTCTGACAGCATTAGTTGGTGTG

AGTGGAGCTGGGAAAACCACTCTAATGGATGTCTTGGCAGGAAGGAAAACTAGTGGATCT

ATTGAGGGAAGCATCACCCTCTCTGGTTACCCTAAAAAACAAGAAACTTTTGCCCGCATC

AGTGGCTATTGTGAACAGACTGATATCCATTCACCAAATGTTACTGTCTATGAATCCATT

CTCTACTCTGCCTGGTTGCGTCTTTCCTCGGATGTTGACGAAAAAACAAGAAAGATGTTT

GTGGAGGAAGTCATGACTCTTGTAGAGCTTGATGTCTTGCGTAATGCTATGGTTGGTCTC

CCTGGAGTGGACGGGTTATCCACTGAACAAAGAAAGAGACTGACAATTGCCGTCGAGCTG

GTAGCAAATCCTTCAATCATATTCATGGATGAGCCAACTTCTGGTCTTGATGCTAGAGCC

GCGGCCATTGTAATGCGCGCGGTGAGAAATACAGTCAACACTGGGCGAACTGTGGTTTGC

ACAATCCATCAACCCAGCATCGATATATTCGAGTCTTTTGATGAGGTACTCTCTCTCTCT

CTCTC

>Traes_3AL_223B275D2

CTTCTGCGCCTACGGGTTTGATGCCGCCGCAGAGCTCGGCGTCCCGGCCTACCTTTTCTT

CACCTCCGCTGCGTCGGTCCTTGCTGCCTACCTGCACATCCCCGTCATGCGCTCCACTGT

CTCCTTCCGGGACATGGGGCGCTCCCTGCTGCACTTCCCCGGAGTCCACCCAATTCCGGC

GTCTGACTTGCCGGAAGTGCTGCTCGACCGCGGCGACAGCCAGTACAAGACAATCCTTAG

CCTTATGGAGCAGCTGCCAAGATCTAAGGGCATTCTGTCGAACACGTTCGAGTGGCTG

>Traes_4DL_2FAC41BEF

TTTCGAGCTGTTTGTTGAGCGTTTGTGCGGGGTGCAGGGTGGTGGTGGACAACGGGGTGT

GCGACACGGCGACGCTGGTGCAGGTGCACAGCGCGCGGAAGCACGGCGTGCTGCTGGAGG

CCGTGGCCGCGCTGTCGGACCACGGCGTCTGCGTCCGGAAGGGGTACATCTCCTCCGACG

ACGGCCGGTGGTTCATGGACGTCTTCCACGTCACCGACGCCGCGGGCTGCAAGGTCGCCG

ACGCCGACAAGCTGCTCGTCCGGCT

>Traes_3AL_AAF026960

GCGGCGTGGGAGGGCTGGTCTGCGTCGGGGTCGTCGCCGTCGGCATCTTCTTCGCCGGCG

CCATGTGCATCGCCAGCAACTCGAGGATGGGGTACGCCTTCTCGAGGGACAGGGCGATGC

CGCTCTCGCGGGTGTGGCTCCGTGTGAGCAAGAACGAGGTGCCCCTCAACGTCGTCTGGC

TCTCCGTCGTCGTCGCCTTCGTCATGGCCCTCACGTCGCTGGGGAGTCAGGTGGCGTTCC

AGGCGATGGTGTCGATCGCGACGCTGGGGCAGTACATCGCCTACGCGCTGCCCATCTTCT

TCCGG

>Traes_2DL_36E87BA3F

ACAAATTGAAGCACCACCCTGGACAGATCGAGGCTGCCGCTATCATGGAGCACATCCTTG

AAGGCAGCTCCTACATGATGCTCGCGAAGAAGCTCGGTGAGCTTGACCCATTGATGAAGC

CAAAGCAAGATAGGTATGCACTCCGCACATCACCGCAGTGGCTTGGCCCTCAGATTGAGG

TTATCCGTGCTGCCACCAAGTCGATCGAGCGGGAAATCAACTCCGTCAACGACAACCCAC

TCATCGACGTCTCCCGTGGCAAAGCTATCCACGGTGGTAACTTTCAGGGCACACCCATCG

GTGTGTCCATGGACAACACCAGGCTTGCCATTGCTGCGATTGGCAAGCTCATGTTTGCCC

AATTCTCAGAGCTGGTGAACGACTTCTACAACAACGGTCTTCCCTCCAACCTCTCCGGTG

GGCGCAACCCAAGCTTGGACAATGGCTTCAAGGGTGCCGAGATTGCCATGGCCTCCTACT

GCTCCGAGCTTCAGTTCCTGGGCAACCCTGTGACCAACCATGTCCAGAGTGCGGAGCAGC

ACAACCAAGATGTCAACTCTCTTGGTCTCATCT

>Traes_3AL_829F70144

CTCGCGCTCTTCCTCAACCTGCCGACTATGCTCGCCGGCAGTAAGGTAAAGGAGCTCGGC

GACTCTATCATCACTTCCCCGGGTGTTCCTCCGTTGAAAGCTTCGGACTTACCCGAGGTT

ACACACAACGATGAAGTACTCAAGACCATCTTAGGCATGTTCGACCGAATGCCAGATTCC

AATGGAATTCTCATTAATTCCTTCGAGTCGCTGGAGACGCGCGCGGTGCGCGCTCTTAAG

GACGGGCTCTGCGTCCCTGGTCGTGCCACGCCGCCGGTCTACTGCATCGGGCCTT

>Traes_2DL_64D191C24

TTCATGTTCCCATTTTTCCTTAATTTGCTTGTGCCAGGTTGTTATCTTTATTGAAGTGTC

CAATTCAGAGACGCTGAACACAGTGGAGATACAACGGACTATCTCCGAAAGACTCAACTT

GCCATGGAATGATGAAGAGCCAATTGCCAAACGAGCCAGATTCTTGGTAAAGGCACTTGC

TAGGAAAAGATATGTAATCCTGCTTGATGATGTGAGGAAGAAATTCCGACTGGAGGATGT

TGGTATCCCAACTCCAGATACCAATAGCCAGAGCAAGCTGATCCTCACATCACGTTACCG

AGAAATATGCTTCCAGATGAATGCACAAAGAAGCTTGATTGAGATGCAGATTTTGGGTAA

CGATGCTTCATGGAAACTGTTCTTGAGCAAGCTGAGCAAGGAGGCTAGTGTAGCAGTTGA

ATCGCTTGGTTCCCAGA

>Traes_7DL_2EA8D5524

ACATGATGGCCATGGCGCTCGGCCGCAACACCACGTGCATACTCGCCGGGAACGGCACGG

TGAGGTGCTGGGGCGTGAAGGTGCCGGAGGAGTACACGCACACCACCTTCGTCTCCATCG

AGGCCGACGGCGACACGGTGTGTGCCGTCATGACGAGCAACTACTCCGTCGTCTGCTGGG

GGAACGACGGCCGCTTCGACG

>Traes_4BL_704315653

CTCGTTATGCTAAGCAAGAGACGCTGACGACGCCGCTCGAGCATCTCGCCGGCGCCGTCG

TCAGGTGCGAGCGCCCGAACCGCAACATCTACGGCTTCCAGGCCAACCTGGAGCTCCAGG

GGGAGGGCCACCGGATACCGCTGGGCCCGTCCAACATCGTGCTGCGCGGCTGCGACCTCA

AGAACACGTCCTGGGCCGTGGGCGTAGTGGTCTACGCGGGGCGGGAGACCAAGGCGATGC

TGAACAACGCGGGCACGCCGACCAAGCGCAGCCGCCTGGAGACGCACATGAACCGCGAGA

CGCTCTTCCTCTCCGGCATCCTCATCGTGCTCTGCTCGGTCGTGGCCACGCTCTCGGGCG

TGTGGCTGCGCACCCACCAGACCGACCTGGAGCTCGCGCAGTTCTTCCATAAGAAGGACT

ACCTCAAGGTCGG

>Traes_7BL_675662218

CGCGCGCCGACTGCCGGGAGATGAGCTGGCTGCAGTCCACGGTCTACATCAACTCCGGCG

ACACCAAGACGCCGGTGGAGACGCTCCTGAACCGAACGACCAGCCTGAGCACGTTCACCA

AGAACAAGTCCGACTACGTCAAGCAAGCGATCACAAAGGAGACGTGGGAAAAGATCTTCC

CCTGGTTCAACAGCTCCGCCGCGGGACTCATGATCCTGGAGCCTCACGNNNNNNNNNNNN

NGGCAGCATCGCCGATGAGGACACGCCATACCCTCACCGGAGCAGCGTCCTCTACAACAT

CCAGTACGTCGCGTTCTGGACGGGCAACGGCACGGACGGGCCGAACTGGATCGGTGGTCT

GTACAACTTCATGGAGCCGTTCGTTAGCAAGAACCCGAGGGGTGCGTACGTGAACTACCG

GGACCTGGACATCGGGGAGAACACGGTGGTCGGCGGTGTCACGACCTACGAAAGCGGCAA

GGTGTGGGGTGAGAGTTACTTCGGGGGCAACTTCGAGAGGCTCGCCATCACGAAGGGGAA

GGTGGACGCCGGCGACTACTTCAGAAACGAGCAGAGCGTACCCCCACTTCTCTCGAGGAA

GTGATAGGCTGAAAGTGTAACCTGCAATCTAATTTGAGGACATCCAACTATAAATTCTAC

ACTTACGGAATCATGGGGATTCTTTGCTTATGGATAGGGCTGCAAGAAAAGCTGAGCTGT

TTGAGATCGATTCATATTTTGGCTTGATTTGAGATTGATTCGAAAAAAAATAAACTGAAT

ATGAACACTTTATGTAACTCGATCAAGAAATGAGCTGATCTTAACCCAACACTAGCTCGC

TCGATTTAGC

>Traes_4DL_97FA38967

CCCAACGCTGGCAACTTGCTTTCCACGGAGCAGCTTGTTTCTGGATCGTCATCTATTGAA

GCCATGGAGGCATCTGAACCATCATGGCAGTTAATGGGTACATCTCCAACTCAGCTAGGA

AGGACACCTTACCCGTTGCAGCAAAGGCAAGCAAGTGTCCTTGGAGACAGGTCCAAGATC

TCCGTCGGCACCTCTCAGAATAAGAGCATTTTACCTAGTCAGCCTTCAGTTCCTTCGGCA

TCTGCTGATTCAGCCTTTAATTTAAAGACTACTGTTCCACCTTCATTAGCTCACTCTACG

AGCATGTCAAATAGTGCTCATTCCACTAGTTTTCTGCGCCCCCGGAATACCTCGACAGTT

TTGCCCCGGCAACATTCCTACACTACGGGATTTGGAAATGCTTTAAATATTGAGACACTT

GTAGCAGCAGCGGAGCAAAGAGATAATACAATTGAGG

>Traes_2DL_342D2421E

TCCATGGTGGCAACTTCCAGGGCACGCCCATCGGTGTGTCCATGGACAACACCAGGCTTG

CCATTGCTGCGATCGGCAAGCTCATGTTTGCCCAGTTCTCGGAGCTAGTGAACGACTTCT

ACAACAATGGTCTGCCTTCCAACCTTTCCGGTGGGCGCAACCCAAGCTTGGACTATGGCT

TCAAGGGTGCCGAGATTGCCATGGCCTCGTACTGCT

>Traes_4BL_1BB7D194D

CGCAAAGCGACTTATTGGCCGGCGATTCAGTGACACGTCCGTACAAAGTGATATCAAGCT

ATGGCCTTTCAAAGTCATTCAAGGTCCTGGTGACCGGTCGATGATCGTGGTGCAGTACAG

GGGGTTGGAGAAGCAGTTCGTTGCTGCAGAGATCTCCTGCATGTTGCTGATGAAGATGCG

TGAGGTGGCCGAGGCCTACCTAGGCACGGTGGTGAAGAACGCCGTCATCACTGTCCCGGT

CTACTTCAATGACTCCCAGCGCCAGGCTACCCTTGACGCCGGCACCATTGCTGGCCTCAA

CGTCATGCGCATCATCAACGAGCCCTCAGCTGCAGTCATCGCTTATGGTCTTGACAGGAC

GTCCAGCAGCAGTGAAGTGAAGACGGTGCTCATATTTGATCTTGGCGGCGGTACCTTGGA

TATCTCGGTTATCAATATTGACAAGGGAATCTTTACGGTCAAGGCCACTGCTGGTGACAC

CCACCTTGGTGGGGAGGATCTTAACAGCCAGATGGTGCAGCACTTCGTGCAGGATATTCT

TAGGAGACACAAGAGTGACATCAGAAGCAACCCGAGGGCGCTCATGCGGCTGAGGATGGC

TTGCGAGAGGGCAAAGAGGATGCTAACTTCCACAGTGCAGGCCAAAATTGAGATTGACTC

GCTCCACGATGGCATTGACTTTTATGGGACCATCACTCGTGCCCGGTTTGAGGAGCTCAA

CATGGGCCTCTTTTGCAAGTGCATTGAGCACGTCGAGAAGTGCCTCATCG

>Traes_2DL_14E458B97

CGCGAGTGGGCCGGGCGGCTCGGCGTGGACGCCAACGGCGACCGCGCTATCCTGCTCAAC

GACGCCGGCGCGCGGTTTGTGGAGGCGACGGCCGACGTGGCTCTCGTCAGCGTCATGCCG

CTCAAGCCCACGTCGGAGGTTCTCAGCCTGCACCCCAGCGGCGACGACGGGCCAGAGGAG

CTGATGCTGATCCAGGTCACGCGCTTCCCGTGCGGGTCGATCGTCGTGGGGTTCACCACG

CAGCACATCGTGTCCGACGGCCGCTCCACCGGCAACTTCTTCGTCGCGTGGAGCCAGGCC

ACCCGCGGCTCCGCCATCGACCCCGTCCCGGTGCACGACCGCGCTTCCTTCTTCCAACCC

CGCGAACCGCTGCACGTCGAGTACGAGCACCGTGGCGTCGAGTTCAAACCCTACAAGAAG

GTGTACGACGATATTGTCTGTGGAGACGGCGACGACGAAGACGACGAGGTGGTGGTGAAC

AAGGTGCACTTCAGCCGGGAGTTCATCTCCAGGCTCAAGGCGCAGGCGTCGGCTGGCGCG

CCCAGGCCCCGCAGCACCCTGC

>Traes_2DL_21A9D8581

CTTCAGCCGGGAGTTCATCTCCAGGCTCAAGGCGCAGGCGTCGGCTGGCGCGCCCAGGCC

CTGCAGCACCCTGCAGTGCGTGGTGGCGCACCTGTGGCGGACCATGACGATGGCGCGCGG

GCTCGACGGCGGGGAGAGCACCAGCGTCGCCATCGCGGTGGACGGGAGAGCGCGGATGAG

CCCGCAGGTGCCGGACGGATACACCGGCAACGTCATCCTC

>Traes_7DL_20408494C

CCTCATGATCCGCATCATCGCTTTCGGCGACACCGCCAAGTTCGAGGGCATGTACCTGGG

CACCTGCAAAACCCTGACGCCACTGATGACCAGCAAATTCCCCGAGCTGGGCATGAACGC

CTCGCACTGCAACGAGATGCCCTGGATCAAGTCCGTCCCCTTCATCCACCTCGGCAAGCA

GGCCACCCTCTTCGACCTCCTCAACCGCAACAACACCTTCAAGCCCTTCGCCGAGTACAA

GTCGGACTACGTCTACCAGCCCGTGCCCAAGACCGTGTGGGCGCAGATCTTCGTCTGGCT

CGTAAAACCCGGAGCGGGGATCATGATCATGGACCCCTACGGCGCCGCCATCAGCGCCAC

CCCCGAGGCGGCCACGCCGTTCCCTCACCGCATGGGCGTCCTCT

>Traes_3AL_3B3CC54F8

AAGTATATGCACCTTCAACCAATTTGCCGCAACGTTTACCTTCTTCTATCCAATCATATA

TACGTGTTGGTAACGTTGGCTACTTGTGTCAAGTGTGCGACTACTACCCGGGCATCTCCT

GCGGTGACCCGCGCTTCACCGGTGGCGACGGCAACAACTTCTACTTCCACGGCAAGAAGG

ACCAGGACTTCTGTGTCGTCTCCGATGCTGATCTCCATGTCAATGCTCATTTCATTGGCA

CGCACAACCCCGCCACTGATCGTAACTTCACCTGGATCCAGGCCATCGGCATGCGCTTCG

TGGACCACCGCCTCTTCGTCGGTGCCAAGAAGACCGTGAAATGGAACAACCGTGTCGACC

ATCTGGAGATGGCCTTGGACGACGAGACCATCGATCTCCCTGCCAAGCTCGACGCGCGTT

GGGAGTCGGCA

>Traes_5BL_6D3081454

CATACATCCAATCAGTCAGCCACCTCAAGACAGATCCCCACAGACCACGGAGCAGTCAGC

CATGGATAAGGCATACATTGCCGTCCTCTCCTTCGCCTTCCTCTTCCTGCTCCACTACAT

TCTGGGCAAGAAGAGCAATGGCAGCAAGGGCGCCGTGCAGCTGCCGCCGAGCCCTCCGGC

CATCCCGTTCTTCG

>Traes_4DS_ABCFAB135

ATGGGGTACTCCAAAACCCTAGCGGCTGGCCTGTTGGCCATGCTGCTCCTAGCTCCAGCC

GTCCTGGCTACCGACCCTGACCCTCTTCAGGACTTCTGCGTCGCCGACCTCGACGGCAAG

GCAGTCTCGGTGAACGGGCATCCGTGCAAGCCCATGTCGGAGGCCGGCGAGGACTTCCTC

TTCTCGTCCAAGCTAGCCAAGGCTGGCAACACGTCGACCCCGAACGGCTCGGCCGTGACG

GAGCTCGACGTGGCCGAGTGGCCCGGTACGAACACGCTAGGCGTGTCGATGA

>Traes_4DL_06FB963EA

GACAGGTTGAGTCCATGCAATCAATGATTCGCATTGTTGGCCTATCTGCTACACTGCCTA

CTTACAAAGAGGTTGCAGAATTTTTGCGGGTTAATGCAGATACTGGCCTTTTCTACTTTG

ACTCGAGTTACCGTCCAGTTCCTCTTGCTCAACAGTACATTGGAATCACTGAAAGGGATT

ACGCCAAGAGAAATGATCTATTCAATAGTATTTGCTATGACAAGGTTGTGGAGTCCATTA

AACAAGGTCATCAAGCATTGGTTTTTGTACATACCCGCAAGGATACAGGGAAAACCGCTA

GAACCCTAATTGACCTAGCTGCAAAAGCAGGGGAAGTGGAGTTATTTTCAAATGCAGACC

ATCCCCAATACGGATTGATCAAGAAAGATGTCAGTAAAGCTAAAAGTCGTGAAGTTATAG

AATTTTTTGATTCTGGATTTGGCATACATAATGCTGGAATGATGCGTTCTGACCGAAATT

TGATGGAGCGCTTGTTTGCTGATGGTCTCTTGAAAGTCCTTGTTTGTACAGCAACATTGG

CTTGGGGAGTGAACTTACCAGCTCATACAGTTGTTATAAAGGGCACACAATTATATGATG

CTAAAGCTGGCGGATGGCGAGATCTGGGGATGCTCGATGTGATGCAGATCTTTGGACGTG

CTGGGAGGCCACAGTTTGATAAAAGTGGTGAAGG

>Traes_2DS_87173CA36

TCGTCGCCGCGCCGGCGTGGTACCCTGCGTTCACCGAGTCGGCGCTGTCGCAGTTCGACG

GGCTGGACCAGGCCGACCACGTGCTCGTCAACTCCTTCCGCGACCTCGAGCCATTGGAGG

CAGATTACATGGAATCGAAATGGGGCGCAAAGACCGTCGGCCCGACATTGCCATCGTTCT

ACCTAGACGATGATCGTCTGCCATTGAACAAGACCTACGGCTTCAACCTCGTCTCAAACA

TGGCTCCATGCATGACAATGGCATGGCTCGACAAGCAGGCTCCTTGCTCTGTGCTTCTTG

CATCCTACGGTACAGTCGCCAACCTCGAGACGACCCAACTAGAGGAGCTAGGCCATGGAT

TGTGCAATTCTGGACAGCCTTTCCTTTGGGTGCTGAGGTCCAACGAGGCAGATAAGTTGC

CCCAGGAACTCCATGAAAAATGCAACAACAAAGGCCTAATTGTTCCATTTTGCCCTCAGC

TGGAGGTATTGGCTCATAGAGCAACAGGTA

>Traes_2AL_D6D12A561

ATCAAATTCAACAATTTTTGTCAACTAGCATTTGAACTTGTAAAGCTACTGGTAACAAAG

TATATTATAATAAAAATGCTGCGAGGACCATATCATTGTCTAAAATCACAGTTTTTTGTG

AACTGGCATGAGTGTACATACAAGACTTGTCCATTTCTCCTATAAAACTGAGGTGGAACT

TGTACTACCAGTTTGATCTTTTACTGATAACTTTATACTTCTACAGAATTTACCTAGATT

TCAACCAGAGAGTCTCGAGAAGAATGCCCAGATATTTGAGCGTGTTGATGCGATGGCCAC

AAGGAAAGGATGCACACCATCACAACTTGCCTTGGCTTGGGTTCACCATCAGGGAAGCGA

TGTTTGCCCCATCCCTGGGACAACAAAAATTGAAAACTCCAACCAAAATGTGGGAGCATT

AGCTGTGAAGCTCACACCAGAGGAAATGGCTGAACTCGAGTCTTACGCGGCTGCAGGTGA

TGTTCAGGGTGACAGGTACCCTCCAATGGCTAGTACTTGGAAGGAGTCTGAGACCCCTCC

ATTGTCATCTTGGAAAGCTGAGTAGACAGTTTCATCTCTTCAACAACTACACTGTTTACT

TTTGATGAGAAGGTAAAAGCAGGATAGAGTTGAACCCAGGATTTCGTTTTCCATTTCTGC

AGATGCATGTATATGAACTGAGCTGCGATGTGTGTATGTTATGCTTGGTTACCGATTTGC

TCCTGCTTAATAAATGTCATTGCTA

>Traes_7DS_28E2128F3

ACCCCGCAGGGGTACCCGGAGACCCCCGGCGTCTGGACGCAGCAGCAGGTCGATGCCTAG

AAGCCCATCGTCGATGCTGTTCACCGCAAGGGCGCTCTCTTCTTCTGCCAGATTTGGCAC

GTCGGGAGGGTCTCCACCAACGGTAAGTTAGGAATCCATGCTAACATTTTTCTGAGAGGG

AAGAATCGATGCTATTTTGTGTTGATAACCTTTACCATAACAAATTTGCTGACGCTGCGA

TTTATCTGTTGTCATTATCAATTCATCATCTGCTCTGTTTGTAATTGCAAGGCAGAGAAA

ACAGATTCATTTGATCCTTTCCTAAGCAATTAGGCATTTAGGCATCCATGTTATTTTGTG

TTGGTAACCTGTTTGATCCAACGACTGATTAATTGTTACTTATCTTGCTCTCTGTTTATA

TCTGAACAAAAACAGATTTTCAACCGGACGGACAGGCGCCGATATCAAGCACTGACAAGC

AGATAACGCCTGATGCTGAGTCTGGCATGGTCTACTCCAAGCCTCGACAGCTTCTAACAA

ACGAGATACCGCTGATCGTCGATGACTTCAGACGCGCTGCCCGTAACGCCATCGAGGCGG

GGTTTGACGGCGTTGAGATCCACGGGGCACACGGGTACCTATTGGAGCAGTTCATGAAAG

ATAGCTCTAACGACCGCACCGACGAGTATGGTGGCAGCCTCGAGAACCGATGCCGCTTTG

CGGTGGAAGTAATTGATGCTATCATCAATGAAATTGGTGCAGATCGTGTAGGAATCAGAT

TGTCTCCATTCGTGGACTATATGGACTGCTTCGACTCCAACCCACATGCACTCGGGATGT

ACATGGTACAACAACTCAACAAACATCAAGGGTTTGTCTATTGCCATATGGTAGAGCCGC

GGATGGCCATTGTGGATGGACGCAGGCAGATACCGCACGGGCTCCTGCCCTTCAGGAAAG

CATTCAAGGGCACTTTCATTGCCGCTGGAGGGTATGATCGGGAGGAAGGCAACAAAGTGG

TGGCCGACGGCTATGCTGATCTCGTTGCTTACGGGAGGATCTTTCTGGCCAATCCAGATT

TGCCTAAGAGATTCGAGCTCGACTCACCCTTGAACAAGTACGACCGTAAGACTTTCTACA

CGCAAGATCCTATCGTTGGCTACACAGATTATCCTTTCCTCGAAGGTGGCTCGAATGCCG

AGTAGTTCACCCACTCGACTTGAATTAGTGATGTGCTTGATTTTATAAAGTATGTAATGA

TTAATTCAGTGTGTGTATGCACGGGGCATGTTCATTGTATCATTTCCACCAATAATTTAC

TTTTGTTCTCC

>Traes_2DS_3109276AA

ATCAAGTGGCGGTCGCGCTCTCGCATGCTGCTGTTCTTGAGGAGTCGCAGCTGATGCGAG

AGAAGCTTGCTCAGCAGCACAGGGACTTGCTGCAGGCAAAGCATGAGGCTGTGATGGCAA

CTGAAGCCAGGAATTCCTTTCAGAGTGCCATGTATGACGGGATGCGCAGACCGATGCACT

CGGTCCTTGGTCTCGTCTCGATGATGCAACAGGAAAGCATGAATCCAGAGCAAAGGCTCG

TGATGGACGCCATCGTCAAGACAACCAGTGTTGCCTCGACGTTGATGAATGATGTCATGC

AAACATCGACGATGGACCGTGAGCACTTATCTCTGGTGAGGAGGCCCTTCAGCCTCCACT

CCTTGATTAAGGAAGCGGTCAGCGTTGTAAGGTGTCTGTGTGGCTCCAAGGGGGTTGATT

TCGAGTTTCAAGTGGAGAATTCTTTGCCCGAAAGGGTCGTTGGCGACGAGAAGAGGGTTT

TCCATATTGTCTTGCACATGGTAGGCACTCTGATAAATCAATGCCGTGCGGGCTGCCTCT

CTTTGTATGTGAATAGTTACAACGAGATGGATGAGAGGCA

>Traes_5AL_BF7B7437A

AATGCTCGCGCCAGCAGATCAATTTCGAGGTCTGGTTCATGAATGGATGACTTATATGGG

TTGCTCTTGTAGGCTGAGGTTTTCTTCCTTGGGCAACTGAGGCACAACAACCTGGTGAAA

TTGATCGGGTACTGCTACGAGGACGAGCACCGGATGCTGGTCTACGAGTTCATGAACGCC

GGGAGCCTGGAGACGCACCTCTTCAAAAGTACCAATGGCTCTCTCCCGTGGATGACAAGG

ATGAAGATCGCTGTCGGCGCGGCCAAGGGCCTTGCCTTTCTCCATGATGCCGACCCGCCG

GTGATCTACCGCGACTTCAAGGCCTCCAACATCTTGCTCGACTTGGATTACAACACCAAG

TTGTCCGACTTCGGGCTGGCCAAGGATGGGCCTCAGGGCGACGCGACACACGTGACAACA

CGTGTCATGGGGACGCATGGTTATGCAGCGCCAGAGTACATCATGACAGGCCACTTGACC

GCCAAGAGCGATGTATATAGCTTTGGTGTGGTGCTCCTGGAGCTCCTCTCTGGGCTACGA

TCAGTGGACCGTGCACGGCGACTCAGGGAACAGAACCTGGTCGACTGGGCTAGACCATAC

CTCAAGCACTCTGACAGGTTGTACAAAGTCATGGACCCAGCTCTCGAGTGCCAATACTCA

TGCAAAGGCGCCGAGGTGGCAGCACTGGTGGCATACAAGTGTCTCA

>Traes_2DL_8BA1CE63D

AATGGCACTGCCGCACAGCAAAACCGTATTGCCGAATGCCGGTCGTACCCACTCTACCGA

TTCGTGCGCAAGGAGCTTGGAACGGAGTACTTGACCGGAGAGAAGACGCGGTCTCCTGGC

GAAGAGGTGGACAAGGTGTTCATTGCCATGAACCAGGGCAAGCACATCGATGCGCTGCTT

GAGTGCCTCAAGGAGTGGAACGGCGAGCCCCTACCTCTCTGCTAAATAGAGGATCGAGAA

AGTGAAGAGTAGTGTGCTTCAGATTTCTGAAGGCTCTGATGGTAATACTGGTTTTTCATT

GTATATTCTAAAAGTTGATGTTCACAATGTTCTTCTAGAGCTGCCAATGTATTGCCAAAG

ATTGCAATTGCATGACTTGGTAGTGTTGGGTAGCCAGTAGAACTTTTATGATGTACGTAA

GTTAAAAAGGCAGTGTGTGTTAAATTTTCATGATAAATGTACTGGCTCCATTTTTTGAGA

TCAACCGGTCCTCGTGACAATAGTCCAAGAACCAACTATTGGAATTTCTTATGGTGTGTG

TACGGCGATTTGGAAAGTAAAACTCGTGCCTAGGTGGTCATCGGTCAGTAATAAGTTCTC

TGAATTCTTTGAACGGGGACGAGGGCTTCTCTGAATTCACAATCTCCCATCTAGTTTTTG

CAGTTTCGGTATTCTGCTTACTAGTTTCAGTCTGGATTTCTATGGAGGCACACAATTATG

TTTAACTTGGTGCTATTCAACTACCGTACCACTGCA

>Traes_2DL_3CD42C7D5

ACCTCGTCCCGCTTTCCTATATTGCGGGACTGGTAACCGGCCGCCCAAACTCCATGGCCA

CGGCTCCAGATGGCACGAAGGTTAACGCGGCAGAGGCATTTAAGATTGCCGGCATCCAAC

ATGGCTTCTTCGAGCTGCAGCCCAAGGAAGGCCTTGCCATGGTGAACGGCACGGCAGTGG

GCTCAGGGCTTGCATCCATG

>Traes_6AL_30A8B8D00

TGGTTCGGCACCAGCTACCTCACCCCCGTCTTCGGCGCCATCATCGCCGACTCCTTCTGG

GGCAACTACAACACCATCCTCGTCTCCCTCGTCGTCTACCTTCTCGGCATGATGCTCGTC

ACCTTCTCCGCCTTCGTGCCCACCACCACGGCGGCGCTGTGCGCGGCGGGCGCGTCGTGC

GCCGGCACCGCCGGCACGTGGGGGCTGAGCTCGCAGGCCGTGGCTTTCGTGGGGCTGTAC

CTCGTGGCGATCGGGTGCGGCGGGGTGCGCTCGTCGCTGCTGCCATTCGGCGCCGAGCAG

TTCGATGACGACAGCCCGGCCGACCGGGAGGGCAAGGCGTCCTTCTTCAGCTGGTTCTAC

CTCTGCGTGAGCTTCGGCCCCATCATCTCCGGCGTGTTCCTCGTCTGGATCCAGCAGAAC

GTCAGCTGGGGCCTCGGATTCGGCATCGCCACCGCCTGCATAGCGCTCGCCTTCGCCGCC

TTCATGCTCGCCACGCCCATGTACAAGCGCCGCATGCCCGCCGGTACGCCGCTCAAGAGC

CTGTGCCAGGTCGTCGCTGCCGCGTGCAAGAAGATTAGCGTCAAGGTGCCCGCCGAAGCC

GGACACCTCTACGAGGTCAGTGACAAGATCGACTCGCCCCAGCCCAGGATCGCGCACACC

AGCGACTTCAAGTTCCTCGACAAGGCGGCCATCGTCACGGAGTCGGACATGGAGGAGAGG

CCGGAGGCGGCGACCTCGTGGAAGCTCTGCAC

>Traes_1BL_34A2B91B8

GAGTCAGTGATCATCGTGCAGTACATCGATGAGGCGTTCGCCGGCATCGGCCCCTCTCTC

CTTCCCTCTGACCCCTACGAACGCGCCATTGCCCGCTTCTGGGCCGCCTACATTGACGAC

AAGGTTGCACATGTATCTTTTTATGCATTGAAATTTATGGCACGGGCATTCGGGGTTTGA

TTTTACTAATGTAATGTGATTTTGTGCAGCTCGTCACCCCATGGGTATAGTCATTAAGGG

CCAAGACAGAGGAG

>Traes_6AL_0444FD89D

GGCGGTGGCAGCGCTCGTGGAAATGAAGCGGCTCGACAGCGCGGCGCGTGGGGAAGAGAT

CAGCATCTCGTGGCAGCTGCCACAGTACTTCTTCCTTGCCGGCGGGGAGGTCTTCTGCTA

CATCGCGCAGCTAGAGTTCTTCTTTGACGAGGCGCCGGACACCATGAAAAGCATGTGCAC

GTCGCTGGCTCTGCTCACCATCGCGCCGGGGAGCTACATGAGCTCCTTCATCTACGCCAT

CGTGGAGGCCTTCACGGCGACGGGAGACAGCCCCGGGTGGATCTCCGACGACCTCAACAA

GGGCCACCTCGACTACTTCTTCTGGGCCATGGCTGCAATGTGCACGCTCAACTTCGTCGT

GTACAGTGGCATCGTCAAGAACTACAGGCTCAAGACGGTCATCTCGTGATGGCTTGCTCT

AAATCTTCAAGATTCCGTTCCATTCCATTACATAGATTACATTGCACAATCGCCAATTGG

TGATGGGTAAACAGTAACTAGGTAGTAGCCAGGTACATATATACACAAGAAACACTAGGA

GGAAATTGTAAGATATAGCGTAGTACAATCAATGGATTGGCGGAAACAATTTTTGGAAAA

AATATTATGC

>Traes_4DL_3E1985F0D

AGCAATCAATTTTTAATTGTATGATCTCAAATCTGTTTGAGGAATACAAGTTCTTTACGA

AGTATCCGGATAAGCAGCTTAAACTAGCTGCTGTGCTATTTGGATCCCTTATCAAACATG

AACTTGTGGCCCACCTCGGACTTGGGATTGCTCTACGCGCTGTTCTTGATGCCTTACGCA

AGTCTGTTGATTCAAAGATGTTTATGTTTGGTACGACAGCATTGGAACAGTTCATGGATC

GTGTAATAGAGTGGCCACAGTACTGCAATCACATATTGCAGATTTCACATCTTCGAGCTA

CTCATGCTGAAATGGTCTCTGCAATTGAGCGAGCACTTGCCAGGATTTCATCAAGTCAAA

ATGAGCCCAACGCTGGCAACTTGCTTTCCACGGAGCAGCTTGTTTCTGGATCGTCATCTA

TTGAAGCCATG

>Traes_7DL_6B3257C57

GTGAGCAAGAACCCCAGGCAGGCGTACGCCAACTACAGGGACATCGACCTCGGCAGGAAC

GAGGTGGTCAACGACGTCTCCACCTACGCCAGCGGCAAGGTGTGGGGCGAGAAGTACTTC

AAGGGCAACTTCCAAAGGCTCGCCATCACCAAGGGCAAGGTGGATCCTCAGGACTACTTC

AGGAACGAGCAG

>Traes_4DL_265644AFB

CCGCAGGAAGGGCGTTCGGTGCGTGGTGCTGGAGTCCTCGCCGGTGCTCCGGACTTCCGG

GTTCGCGATCGCGGCATGGACGAACGCCTTCCGCGCGCTTGATGCCCTTGGAGTAGGCGA

CACCATTAGGAGTAAACACCTGCAGATCCAGGGGGCGTGTGTCATGTCTTCGACAACAGG

GCAAGTAGCCCGAGAGGTAGATCTCCGGGTGCAAGGAAAATGGTGAGACACGATTCCATA

ATCTCTAATCTGAATT

>Traes_5BL_53F49C2AF

CCACGCTGGTGCAGTGCTTCGACTGGGAGCGCGTGGACGGCGCGGAGGTGGACATGACGG

AGGGCGGCGGACTCACCATCCCCAAGGTCGTGCCGCTCGAGGCCGTGTGCAGGCCGCGCC

CGGCCATGCGCGATGTGCTTCAGAGCCTCTGATGGTCGAATTTCTTGGCAAGGCATCATA

ATGCATGGTTTTGATAGGCTGCTGATGTATCTACTATCTACTACCACTATAAAAGAAAGA

GAGGGGCAGATCCAAACAATCCCACCCATCAATCATCAAGATCTAATGGCCCTTAATCAT

TCTGTGTTTAACGCTACCAATCA

>Traes_4DS_3D4429A85

GTAAAGTATGTGCTGGCACCGTACACTGGCGGTTTGCCGCAGCTGGTACATAACCCGCTG

TCATGGACCAAGATGGCGAGCATCATCTTCCTGGATTCGCCGGTCTGCTCGGGCTTCTCG

CATGCTCGTGACCCCAAAGGCTGCGATGTCGGAGACTACTCGTCCTCTCTGCAAGTCCAA

AGATTCCTGAATAAGTGGTTCACTGATCACCCACAGTACCTTTCAAATCCTTCTACCTTG

GAGGAGATTCATACGCGGGAAAGGTGATTCCGCTTATTGCACACTACATGTTACAAGGAA

CTGAAAAAAGGGAGCAGCCTCTTATTAATCTCAAGGGCTACCTGATCGGCAATCCTGTAA

CAGACCCAAAGTTCGATAAAAATTTCCAAGTTCAAGGGGCTCATGGCTTTGGGATAATAT

CTGACCA

>Traes_4DL_BAC2D8210

CCTCCCGAATCGATAGGGTGCGCGTCATGTCTCCGACTACCGGGGAAGTGGTGCGAGAGC

TGGATCTCCGAGTGCAAGGCAAACTGGGACCCCACGAAGCCCGGTGCGTGCAGCGTAACG

TGCTCCTCCAGGCGCTGGAGGAAGAGCTTCCGGCAGGCACCATCCGCTACTCCTCCAGGA

TCGTCTCCATCGACGACGACAAAGAAAAAGAAGGCGGCGGCGATGCCAAGATCCTGCATC

TCGCCGACGGCTCGACGCTCCGAGCAAAGGTGCTG

>Traes_2BS_CEB8A1C5A

TCATGAAGTGGCTCGACGGCAAGGAGCCGGGCTCCGTGGTGCTCGTCTCCTTCGGCAGCG

AGTACTTCATGTCAGACCGGCAGATGGCGCAGATGGCGCGCGGGCTGGAGCTCAGCAGGG

TGCCCTACCTCTGGGTGGTGCGGTTCCCGAACGCGGAGGACGACGCCCGAGGTGCGGCGA

GGTCCATGCCGCGGGGGTTC

>Traes_5DL_E1438A97F

CGATTTTGAGCACCGGGGCGTCGAATACTACCGCCCGACGACCAGCAACCCCAAGCAGGT

CGAGAGCGGGCACCACGGCGCCGACAACGTGATCATCCACAAGGCGCACTTCACCAAGGA

CTTCATCGCCGGGCTTCGCGCCAAGGCGTCTGAGGGGCGCGGCAGGCCGTTTAGCCGGTT

CGAGACCACGCTCGCGCACCTGTGGC

>Traes_6AL_A3D19D5F2

CCCATCATCTCCGGCGTGTTCCTCGTCTGGATCCAGCAGAACATCAGCTGGGGCCTCGGC

TTCGGCATCGCCACTGCCTGCATCGCGCTCGCTTTCGCCGCCTTCGTGCTCGCCACGCCC

ATGTACAAGCGCCGCATGCCCGCTGGCACGCCGCTCAAGAGCCTCTGCCAGGTCGTCGCT

GCCGCGTGCAAAAAGATCAGCATTAAGGTGCCTGCCGAAGCCAAACACCTCTACGAGGTC

AGCGACAAGATCGACTTGCCCCAGCCCAAGATCGCGCACACCAGCGACTTCAAGTTCCTC

GACAAGGCGGCCATGGTCACGGAGTCGGACATGGAGGAGAGACCGGAGGCAGCGACCTCG

TGGAAGCTGTGCACCGTGACTCAGGTGGAGGAGCTCAAGATCCTCCTCCGCCTGCTGCCC

GTGTGGATCACCAGCGTCATCGTGTCATCGGCCTTCGCGCAGATGAACACCACGTTCGTG

CAGCAGGGCAGCGCCATGGAAATGACCATCCTGTCGGTGCCGGTGCCCGCGGCGTCGCTG

GCCTCCTTAGAGGTGATCTGCGTCATGACATGGGTGCTCCTCTACAACAAGGTGATCGTG

CCGGCGTTGAGGAGCTTCT

>Traes_6AL_0C064D4DD

GGCGGGGAGGTGTTCTGCTACATCGCGCAGCTGGAGTTCTTCTTCGGCGAGGCGCCGGAC

ACCATGAACAGCATGTGCACGTCGCTCGCTCTGCTCGCCATCGCGCTGGGGAGCTACATG

AGCTCCTTCATCTACGCCATCGTGGAGGCCTTCACGGCGACGGGAG

>Traes_2DS_F5DCF1511

GTCTGCCTAGAGCAGCAGGAGAGGCTGCTCGTCTACGAGTTCCTCCCAAACCGGAGCCTC

GATAAGATCCTATTCGACACGGAGAAACGCGAGCAGCTCGACTGGGGAAAGAGGTACAAG

ATCATACACGGGATTGCTCGAGGCCTGCAGTACCTCCATGAAGACTCCCAGCTCAAAGTA

GTCCACCGTGACCTCAAAG

>Traes_2DL_44DA40D1B

CAGCGACTGGGTCATGAACAGCATGATGAACGGCACCGACAGCTACGGTGTCACCACCGG

CTTCGGCGCAACCTCTCACCGGAGGACCAAGGAGGGCGGCGCCCTGCAGAGAGAGCTCAT

CCGATTCCTTAATGCGGGAGCCTTCGGCACCGGCACCGACGGTCACGTCCTGCCTGCCGC

GGCGACGAGGGCGGCGATGCTCGTCCGAGTCAATACCTTGCTCCAGGGATATTCAGGCA

>Traes_7DS_1ED94344B

CACAGGCATACAGAGCAGAGTTCAGAAGCAAATAATAGAGCATACCATCGTACGTATTTG

CGCTGCACAATCTGCATCCAGTTTCCACAAGATGGTAGCGAGCAAGATGGTGGCGAAGGA

GGCGATCCCGTTGCTGACGACGCACAGGATGGGCCGGTTCGAGCTCTCCCACCGGGTGGT

GCTCGCGCCGCTCACGCGCTGCCGCTCCTAGGCCAACGTGCCGCAGCCGCAC

>Traes_4DL_78F59773D

CCGCGACCGGCCGAAGCTGCTGTTCGACGTCGTCTGCACCTTGACGGACCTGGACTACGT

GGTGTACCACGGCACCTTCGACACCGACGGCGATCACGCGCAGCAGGAGTTCTACATCCG

GCGGCTGGACGGGCGGGCGATCAGCTCGGAGGGCGAGAGGCAGCGGGTGATCCAACGCCT

GCAAGCGGCGATCGAGAGGCGCGCGTCCGAGGGCGTGAGGCTGGAGCTGCGCATCTCCGA

CCGGCGCGGGCTGCTGGCCTACGTGACGCGCGTGTTCCGGGAGAACAGCCTCTCGGTCAC

GCACGCCGAGATCACCACCCGGGGCGACATGGCGGTGAACGTCTTCCACGTCACCGACGT

GGCCGGCCGCCCCGCCGACCCCAAGACCATCGACGAGGTCATCCACGGGG

>Traes_2AS_A707C5A57

ATGACCGCTCTCGACTTCGTGCCCGCCGAGAAGGCCCCCATAGCCGACACCGTCGCTCTC

GGCACCGTCACCAACAATGCCGTCGCCGCTTCCGCCGTGCTGGTGCAGCAGCAGCAGCAC

AGGAGGCTGGAGGGGAAGGTGGCCATCGTCACCGGCGGGGCCCGCGGGATCGGCGAAGCG

ATTGTGCGCGCGTTCGTTCGTCAAGGCGCGCGGGTGGTGATCGCCGACATCGACGGCGCG

GCCGGGGAGGCGCTGGTGGCCGCGCTGGGCGGCGCCTGCTGCAGCTATGTGCACTGCGAC

GTGTCGGTGGAGGCTGACGTGGAGCGCGCCGTCGGGTGCTGCGTGGCGCGGCACGGGAGG

CTGGACGTCCTCTGCAACAACGCCGGCGTGCTGGGCCGGCAGGCGCCCCCGGCG

>Traes_1DL_27447A67F

CAGGGTTTGGTAGACGACATTGTTGGTCTCTCGAGATCTGCAAGGAAGATTGTAGCTTTC

GGCGAGAATAAATGCACGTTTTACTCGATTCGATTCATCCTCCCTGCTGTCGCGATTGCT

TTCATCCATGAGAAAACAATAGTACTAGTACCACGCGTGCGGCTGAGAGGAAGAAACAGA

GGGCGGCTAATCTTCCTCCATGAAACGCGTTGAAAAGTCAAACGGCCGATGGTTCTCCGT

ACTGACCGTGTGAATGAACGACCTTCTCTTCTCTGGCAGGACGGCGATCATGGCGAGGGA

CGCGCTGGCCTTGATGGACCATCTGGGGTGGAAGAAAGCCCACGTCTTCGGCCACTCCAT

GGGCGCGATGATCTCCTGCAAGCTCGCAGCGATGGCGCCTCACCGGCTGTCCTCGCTGGC

GTTGCTCAACGTCACCGGCGGCGGGATGGAGTGTTTCCCCAAGGTAGATGCACAGATGCT

ATCTCTCGCGTTCCGGTTCTTAAGGGCGAGGACTCCGGAGCAAAGAGCTCTTGTGGACTT

GGAAACCCACTATACGAAGGAATACCTCGATGAGGAAGTTGAATCCTGCACAAGGAGAGC

GATCCTATATAAGGAATATGTGAAAGGCATATCATCTTCAGGGATGCAATCTAATTGCGG

GTTTGAAGGTCAAATTAACGCGTGCTGGACTCACAAAGTGACAACTAAAGAGCTAGATAC

AATTCGTGCTGCCGGTTTTCTGGTTTCAGTTATTCATGGAAGACATGATATTATTGCGCA

AGTATGTCATGCGAGGCGGCTTGCACAAAGGCTTCTTCCTGTTGCTAGAATGGTAGATCT

TCATGGTGCACATCTAGTCAGCCATGAAAGACCACAAGAGGTCAACAACGCGCTGATGGA

TCTGATAAAGGCCACCAAGTCGGCGACGGCGCCGCACGAGTGGTCGTCCCAGCCAGAAAA

CACATCAGAAACCGGTGCCCTTATTTCTGCAAGGCCTGTAACCCTCGCGATACGAACAGG

CGAAGCCGGCAACGCTGCCGTAGCAGCGTATAACCTACTTGCAAAGTTGCAACTAAGCTT

TCTTTATGTCATAGGCCTGGTAGTGATGGCGTTTGAGCACATGAGGAACATTGTAAGAGT

AACGAAGCCCGTGAGGGTCGCGGCGATCGAGTCATCATAAAGAAACAAAAGACATGTAAA

TTATACCCCCGAAGCCGGCGAGGAGATCATATTTCATGTCTCCTCCGTATACCATCCGCG

GAGATGAGACGGATTTAGAGTAGCATCTCCGCAAGAATCGAACCTATTTTCCCTCCTCCC

TTGAGAAAGGCCCTCTGCTCTCGGCAGAAACAACGTTTCGAGACTGAAGAACAGAGGAGC

TGTACCTGCGTTGCGCACCTGGGCAGAAGCGTCACGGGGCGTGGTGATTGAGATGTGCTG

CCATACATTGTATTAGCTCGGAGAGAGCAAATGGATCGGATGTACCTTTGTGTGATGAGT

GTAATTAAGCTGTGACCAATATGTACACGGCCGCATGTTGCGTTGCGTGGGTGCTGTCGC

CGTTCTTGTGTACTCCATTGTTGTGCTGTGATCATTCCTTTGAATAAATCGGCTCGATCT

TCACTTTGCTGCACCCTTCTGGGGTAGATGAATATTTTTCTTACCTGTACAATGTTAGTT

GCTGATATAGTACTCCCGTATAACA

>Traes_7DS_0467D80FB

TCCTACGGCAACGTGCCGCAGCCGCACGCGGCGTTGCACTACTCGCAGCAGGCGACAAAG

GGCGGCCTGCTTATCGCCGAGGCCACCGGCGTCTCCGCCACCGCCCAGGGGTTTCCTGAT

ACTCCTGGCATCTGGACGCAGCAGCAGGTCGACGCTTGGAAACCCATCGTTGATGCCGTT

CACGGCAAGGGCGCTCTGTTTTTCTGCCAGATTTGGCACGCCGGAAGGGTCTCCTCAAAC

GATTTCCAGCCGGATGGGCATGCGCCGATCTCCAGCACCGACAAGCAGATACCGCCTGAT

GCTGAGTCCGGCATGGTCTACTCCTAGCCTAGACGGCTTCAGGCAGATGAGATACCGTTG

ATCGTAGATGACTTTAGACGCGCCGCTCGGAATGTCATTGAGGCGGGGTTCGATGGTGTA

GAGATCCACGGAGCACATGGGTTTTTCTTGGAGCAGTTCATGAAAGATAGCTCAAACGAC

CATACTGACAAGTATGGTGGCAGCCTTGAGAACCGATGCCGCTTTGCCGTGGAGGTGATT

GATGCTATCATCCACGAGATCGGTGTGGATCGTGTGGGAATCAGGTTGTCTCCGTTCGTT

GACTACACGGATTGCTTCGACTCTAATCCACATGTGCTCAGTACCTACATGGTACAACAA

CTCAATAAGCACAAAGGGTTTCTTTACTGCCACATGATAGAGCCTCACATGACCATTGTG

GATGGCCGCATGCAGATACCCCATGGGCTATTGCCCTTTAGGAAAGCATTTAATGGCACT

TTTATTGCCGCTGGAGGGTATGATCGAGAGGAAGGCAACAAAGTGGTGATTGATGGTTAT

ACTGATCTCGTTGCCTATGGGAGGCTATTTCTGGCTAATCCGGATTTGCCTAAGAGATTC

GAGCTAGATGTCCCCTTGAACAAGTATGACCGGTCCACCTTCTACACGCAGGATTCTGTC

ATCGGTTACACAGATTATCCTTTCTTTAATGGCTCAAATGCAGAGTAGTTTTATTGACCC

AACTTCAATCACTGATGTGGTTCGCTTATAAATGTAATGATTTATTCAATATGTGAGTTC

ACGTGCATGTGCATTGTATAATTTTCACAAATAATTTATTTGTGCTTTCCACTGTAATGC

CAAAATATAGAGATATTCATCTTGTGACAAATGTTCGTACGTATTTGTTTTTATTTCGGT

ACAAGTGTGGATTCACATTGTTCTTCATTTGAAA

>Traes_1AL_3E87FA48B

CTCGCCGAAGACCCCACGGCTGCCGCAGGTCCCGGTCCATTTCAGGCCGACTGGTCGACG

GGGAGCGAGATGAAGCCGTACAGGACCAGACGACCCCAAAGCCCGGAGGTACTGGCGGAG

GCGGCCGGCGATGGAGGGAAGCAGCCGCCGCGCCGGCAGCGCGAGGGCGTCGCTCAAGGT

GGTGCTGCTCCACGGCAGCCTCGACATCTGGGTGCGCGACGCGGGGGGCCTCCCCGACAA

GGGCGTGCTCTACAAGAAGTTCGGCGACCTCCTCGGCCTGCACATCGTCAGCTCCGTCGC

CGGCAAGGTGCCCAGCGCCTCGATGACCAGCGACCCCTACGTCACCGTCCAGGTCTCCGC

CGCCACCGTCGCCCGCACCTACGTCGTGCCCAACAGCGAGGACCCCGTGTGGGCGCAGAA

CTTCGTGGTGCCCGTCGGCCACGAGGCNNNNNNNNNNNNNNNNNCTTCGGCGGCCAGGTC

ATCGGCGCGGCGGCCATCCCAGCCGAGCAGCTCCTCTGCGGGGACAGGATCGAGGGCGCC

TATCCTCTGCTGGATCCCAATGGCAAGCCGTGCGCTCCCGGCGCGGTGCTGCGGCTCTCC

ATACAGTACACCCCGGTGGCTCGCCTCACAGCGTACCACCGTGGTGTCGCTGCTGGGCCG

GACAGCCATGGAGTGCCAAATGCATACTTTCCTCTGCGCCGTGGCATGAGGGTGACCCTC

TACCAGGATGCACATGTGCCGGAGGGCTGTCTCCCGGACATCCGGCTTGACAATGGGCTC

CAATACCAACATGGGCAATGTTGGCGCGACATGTACACCGCCATAATCCAGGCACGGCGG

CTGATTTACATCGCCGGCTGGTCGGTGTTCCACACCATTCGGCTCGTGAGGGACGGGGCC

AAGGAGGTGCCGTCACTTGGGGACCTGCTGAAGATGAAGTCACAGGAAGGGGATGGATAT

ATGCGAACACGAGATGAGGAAACACGTAGATTCTTCAAGCACTCTCCGGTTCAAATATTG

CTTTGCCCAAGATCTGCTGGGAAACGTCACAGCTGGGTGAAACAGAAGGAAACAGGAACA

ACATATAGTCATCATCAGAAAACAGTTATCGTGGATGCTGATGCTGGTGGTAATAGGAGA

AAAATAATTGCTTTTATTGGAGGCCTTGATTTGTGTGGTGGACGCTACGATATACCTGGG

CACCCTCTGTTTCGGACTCTTCAAACTTTGCACAAGGAGGATTATCACAATCCAAACTTT

GCTGTGGTTGATGCTCGTGGCCCAAGGGAACCATGGCATGACTTGCATTCAAAAATCGAC

GGTCCAGCAGCTTATGACGTTCTAAAGAATTTTGAGGAGCGTTGGTTGAAGGCATCGAAA

CGCCATGGTATTAAGAAGTTTGGAAAATCATATGATGAAGCACTTCTCAGGATTGAAAGA

ATACCTGATATCATAAACATTAGCGACACATTATATTTTAGCGATAACGATCCTGAGGCA

TGGCATGTTCAGGTGTTTCGATCTATTGATTCCAACTCTGCCAAAGGATTTCCAAAGGAT

CCACGAGAAGCAACCAGAAAGAATCTTGTTTGTGGAAAGAATGTACTAATTGATATGAGC

ATACACACAGCTTATGTGAATGCCATCCGGGCAGCCCAACACTTTATTTATATTGAGAAT

CAGTACTTCATAGGTTCTTCATTCGATTGGGATTCAAACAAAGATATTGGGGCTAACAAT

CTAGTACCAATTGAAATTGCTCTCAAAATCGCAACCAAAATTAAGGTGAACCAGAGGTTC

TCTGCATACATAGTGCTTCCTATGTGGCCTGAGGGTAAACCAACTGGTCACATAGCACAA

AGAATTCTTTACTGGCAGAACAAAACAATGCAAATGATGTACAAGATAATATATAGAGCC

TTGAAAGAGGCAGGTTTGGATGATGTATATGAGCCTCAGGATTATTTGGTCTTCTTTTGT

CTTGGCAATCGTGAAGCTTCTGACAGTCCTAGCGCTTCAAGCACAGCAGATAGTCCTCAG

GAACAAGCTAGGAAAAATAGGAGGTTCATGCTGTATGTACATTCAAAGGGCATGATTGTG

GACGATGAATATGTGATAATTGGATCAGCTAATATCAACCAGAGGTCCATGGAAGGAACC

AGAGATACTGAGATTGCTATGGGAGCGTATCAACCACAGTACACCTGGGCAAATAAAATT

TCTGCCCCTCGTGGACAGGTTTACGGGTACAGAATGTCGCTCTGGGCTGAGCATATCGGA

GCTATCGAGGAAGACTTCAACCATCCAGAGAGCATAGAGTGCATGAGGCGGGTTCGACAT

CTCGGGGAACATAACTGGGATCAGTTCGTTGCCAATGAGGTGACTGAGATGAGAGGGCAC

CTCTTGAAGTACCCTGTAAGTGTTGACCGTGAAGGCAAGGTGAAACCCTTGCCAGGATGC

ACGACATTCCCAGACATGGGCGGGAACATTTGTGGCTCTGTCCCCTTTACACTCATCCAT

GATAACCTCACAATATGAATTTGGTTGTGCAAGTATGTATAGTTGCTAGTGGTTTCTGGG

GTAGCCTGGACAGGTAAAATGGCAATTTTATAGTATCTTGGATGTTGTTAAGGACGAGCC

GTGAGAATTGTTAGTTATGTTCTTCAAGTAGTATGATGTTAGTTGCCCA

>Traes_4DL_C07E9564F

AGGATACCTTGAATCGAACACTCTGCGAAAAGGTCCCTTATTCAGTTGACCAGCACCACC

TTGATGACCCACATGTCAAGGCTAATTTACTCTTCCAGGCACACTTCTCCAGAGCAGAAT

TACCAATTAGTGACTATATCACTGACTTGAAGTCAATTCTTGATCAGAGCATACGTATTA

TACAGGCTATGATTGATGTATGTGCCAACAGTGGATGGCTTTCAAGTGCATTGACATGTA

TGCATCTCTTACAAATGATCATACAGGGTCTCTGGTTTGAGAGAGATTCATCACTCTTGA

TGTTACCATCCATGAATGATAACCTTCTAGATCATCTTAAAGGCAGAGGAGTTTCAACAG

TTCTGAGCTTGCTAGATCGTTCTCGTGAAGAATTACACAAGTTGCTTCAACCGTTTTCTG

CTGCAGAGTTGTATCAGGATCTGCAACATTTCCCTCGCCTTGATGTTAAAGTTAAGCTAC

AGAATGAAGATAAGGAGCAGTCAAAACCTCAAATGCTGAACATTAGAATGCAAATTAAAA

ATACGCGTCGTTCGCCAAGAGTATTTTCCTCTAAATTCCCTAAGGCGAAACAAGAAGCCT

GGTGGCTGGTCCTGGGGAATATTACAAGCTCGGAGCTATATGGTCTGAAAAGAATTAGTT

TTGCAGATCGTGTGCTTAACACTCGCATGGAATTGCCACCGATGTTGAATATGCAGGAGG

CAAAGCTGATTGTTGTGTCAGATTGCTACCTTGGGTTTGATCAGGAGGTCTCCCTTGGGC

ATCTAGCGAAGGTCTAATTCTTCCGCGGACTAGCAGATCTGCTTTTGTGGAACTCAGCTC

CCAGGAATCCAGATATTCAAAATCAAGCATGCTCCCATGCTCATCAGAGCCTTTCTGGAT

TTCGCTGTTGTATAGGTTTACTTGAGTGTAGCTAACAACACGTCTCTTGTAATGCTTATA

TATACAAATCGATTGTGAGGTGGTTTTGGGCCTCCTACGATTTGTACACGTCTCTTTTGG

AAGAAATGAACACCTTTTTAAGTGTTTTTTTTGAAACGAGGCAAAAGATTTGCCATTTTC

ATTGAATAAGGAGAAGAGTACATCACAACCCCGCCAAAGCGGAAAGGGCAAAGGCCTGCT

CTCGCGGCATCAAATTACACAAATGTTTGGCGCCCGCCATAGCCCAGATAGACGCCTTCT

CCTTAATAGTTCGC

>Traes_3AS_8EF6BB210

CTTCAAATATATTACCACCTTGAATTGAAAATCTGAAATTCAGCTGTTGCTGGATTTTCC

TCATTAACCAGTCAGTGGAAAGGGGGTTTGGCTTTACATAGAAGCTAGCAAAGTTATCAT

GACAATGCCAATTCATACTACAAAAAGTTTCATTCATTTGTCAATCGACTACTATACTTC

ATAATGTTTAAATTATTTGCAGACTCTTCTTATGCAGGGGACCGTAAAATGGGAACAGGG

ATGATTCTTAAAATAGTTGGCGCAGGACTAGGTGGTGCTGTACTCATGGCATGTCTCGTC

TGTTTTGTCTGGTACAAACGCAAGAAGAGGAAACAAGCTATAGCTTCAAAGGAGTTCATG

CGAAGTGGATCATCAATGACGTCATATAGTAAAGACCTTGAGTTGGATGGTTCTCCTCAT

ATCTTCACTTTCGAGGAACTTGAAGTAGCTACTGATGGATTTAGTACCTCAAGGGAGCTT

GGTGATGGTGGTTTTGGAACTGTTTATAAAGGAAAACTCAAGGATGGGAGAGTAGTTGCA

GTGAAACGCCTTTACAAGAACAACTACCGAAGGGTTGAGCAATTCCTGAATGAGGTTGAC

ATCCTGTCCCGCCTGCTCCACCAGAACCTTGTGACCCTATATGGATGTACGTCTCGGATG

AGCCGTGACCTTCTTCTGGTGTATGAGTTCATCGCAAATGGGACGGTCGCGGACCATCTT

CACGGATCCCGTTTGGCGGAACGAGGCCTCACATGGCCTCTGAGGCTGAACATTGCCATA

GAAACGGCTGAAGCACTGGCCTACCTCCATGCCGTCGAGATCATACACCGAGATGTGAAG

ACGACTAACATATTGCTGGACAACAGCTTCCATGTCAAAGTTGCAGACTTTGGGCTGTCG

CGCCTGTTCCCGCTCGAGGTCACCCATGTCTCGACCGTTCCACAGGGCACACCGGGCTAC

GTCGACCCAGTGTACCACCAGTGCTACAAGCTGACTGACAAGAGCGACGTTTACAGCTTC

GGCGTCGTCTTGGTGGAGCTGATATCGTCGAAACCTGCTGTGGACATGAGCAGGAGCCAC

AGTGAGATCAACTTGGCGAACATGGCTCTCAACAGGATTCAGAATCATCAAGTTGTTCAG

CTGGTTGATCCGGAACTCGGCTACGACACCGACCCCGAAACGAAGAGGACGATCGATCGC

ATCGCCGAGGTGGCCTTCCAGTGCTTGCAGCTCGAGAGGGATCTGAGGCCGTCGATCAAG

GAGGTGGTGGAGATCCTAACTTGCGTCAGGGATGGAGACTGTCAATCTAAGAGCATGAAG

AAGAAGGCATCTCAGAAAGAGGACGTGTGCTTGCTCGAGGACGGCCTGCAGTTCTCGCCC

GACACGGTCATACATAGATTCCATAGCCAGTCAACTAACCACTCGGTAGCATCAAATGCT

AGCGGATTATCTAACAGTAAATGTTGAAAAGAATGTTGCATAGGCCCTTTTCGGCACTCT

TTGACACTGACAAAACAAAAAATAATATTAAAGCTTGGCAACATGGGG

>Traes_6BL_2F6BE8415

AAAACGGTTGAAGAAAATCCATGCAGATGACAGACAACAACAGAAGTTAGTGGAAGTATC

CTGTGGCACAATTGATCATGTCCATACCCATCATTCTAGACATTGTCTTGACTTTATTTC

TGGAAATGGAGATGACTCAGTACATCCAACCAAATTGAAGATGTTACATCAACTAGATGC

AAATATCATGGAGGATGAATTTGCCTCCAGCCAAAAACATAATGGCTGCAACTACCTATC

TCCATCTGTAGAGCTTGGGCCAAAGCGCTTGAAAATTCTTGGCCCATCATTTCCCAGAGG

GAATCATGAGCTGGAAATCTCTTGTAGATTCCAGGAGGACAGTGACTTGGCTAGTCACCA

TGCTCGATGAAGTATAACAAGGCAATTGGTGCTAACACCACATATGTTAACAAACATCAG

TCTTAACTTCTGGAAGCTGACAACTCCTTCGAGCCAAGGTTTCCTGTTTGTTGGTAGGTC

AGTCGGTAATGGATGGTTCATAAGCAATTCTTTCCGCAGGCCTCCAATAGAGGCACTGTC

TTGGCTGGATTCTTGATTGCATTTGCCAGGTTACCTTTGTGGTAGGGGACCTGAAAAGAG

TAGGCAGACAGTTTCTGATGTTCTTTAGTTGTACATCTTCACTTATCTCCTTTTTTCTAG

GCTTTCCACTGTAAAGAGGTTAGTCTATAACTTTTACAGTTTCTTTCATTCTTTTTCCAC

AGACACACATGTACCTAGAATGCTTAAATAGATGCAAATATGAAATTACTGTATCGCCTG

ACAAACCATTCTACTTTCTACTTGTCTTCATACTTTGTGTAATCTCTCGTCATCAAGGCC

ATCAAACTTCTTATTTTTATGTCATTATTGTTCACAATATTATTTGACTCCAGTGTGTTG

TTCCCAGTATTATTTGTCTCCAGTGTGTTTATAACTTGAAAAGCAATTAATTGATTGATG

CTGACAGGAAATTAATATCAAAGATGTACATTTGTTTCCAGATTTGTTAATACTTGATGC

TGACTAGAAATCACTAGCATAACTACTTGTTAT

>Traes_2BS_1B96DD5CC

CCGAGCGCGACCGCCTGCCGCTCAGCCTGGAGCGCTCCTCCGGGTTCGTGGCCATCAAGA

CGTGCGCTGACATCGAGCGCAAGTACGTGGACTACCTGTCCCAACTCGTGGGCAAGGAGG

TCGTGCCCACCGGCCCGTTGCTCGTGGACTCCGGTGGCTCCGACGGGAAGCGCGACGGCG

GCCACATCATGAAGTGGCTCGACGGCAAGGAGCCGGGCTCCGTGGTGCTCGTCTCCTTCG

GCAGCGAGTACTTCATATCAGACCGGCAGATGGCGCAGATGGCGCGCGGGCTGGAGCTCA

GCAGGGTGCCCTACCTGTGGGTGGTGCGGTTCCCGAACTCGGAGGACGACGCCCGCGGCG

CCGCGAGGTCCATGCCGCGGGGGTTCAAGCCGGCGCGCGGGCTGGTGGTGGAAGGGTGGG

CGCCGCAGTGGCGCATCCTGTCGCACCCTTCCTGCGGCGCGTTCCTGACCCACTGTGGGT

GGAGCTCGGTGCTGGAGTCCATGGCGGCGGGGGTGCCGATGGTGGCGCTGCCGCTGCACA

TCGACCAGCCTCTGAACGCCAACCTCGCGGTGGAGCTGGGCGCGGCGGCCACGCGCGTGA

AGCAGGAGCGGCTCGGGGAGTTCAAGGCGGAGGACGTCGCGCGGGCGGTGCGCTCGGCTG

TCAACGGGAGAGAATGGGTGGCGGCGAGGCGCCGTGCGAGGGAGCTGCGTGAGGTGGTGG

CGCGGAACGACAGCGACGACCGGCAGATCGCGACGCTGCTGCAGAGGATGGCGCGGCTCT

GCGGCAAGGGCCAGGCCGTGCCAAATTAGTGGGCTTTTTTTGGTTTGAACGTTCTTTTGC

TTTTTTTTTTAACGGTTTTAGTCGAGAATAAATAAGAGTCGGGCGCAGTGGCTGCCACAG

CCCACCTGTACTCGTTTTTTTTTAGGCAAACAGCCCATCTGTACATGCCGCTCGGGCGAC

TAGTTTCGGCCTTTCCAGGTTGAACTAATTTCGGCCTTTTCAGTTTCCTTCAATAAAAAA

ACGGCCTTTTCGGAGAAATAA

>Traes_1AL_2E9C3A36B

CCGCTTTCGCGCGTTCCCACCCACCGTCCAACCCAATCCATGGCTGCCGCGACCGCGTGC

TCGAGAACCCAAACAGCTTGCTTCCCACGCCTTTTCTTTCCCTTGGGGATTTCCAATCTT

TATCGCGCACGCCAAACACCTTCCATGTTCCAAACTTGCAATCTCCCCAACTTTTCCTCT

CCCAAAGTACCAAGCCTATATATACGCTCACCCGCCACAATTCAGCATGGACACAACCGA

GTTCCATCAGCAACAGAAGGCACCACAATCCCAATTCCCAACACCGATTACACACGAGAA

GACGACCATGGCGGTCAGCAAGCAATGGACGCGGGTGCGCACGCTCGGCCGCGGCGCCTC

GGGGGCCGAGGTCTTCCTCGCCGAGGACGATGTGTCCGGCGAGCTCTTTGCGGTCAAGTC

CGCCGTGGGGGCGGCGTGTGCGGCGGCGCTGAGAAGGGAGCATATGGTGAT

>Traes_4DS_92795CFC91

CTTAGCTGCAACCACCAGTGCCTCAAAGACTCTTGATCAACAAACTCTAGCTGATCACTG

CTAGCTAAGCTTGCTACATAGCAAGCCATGGGTTACTCCAAAAACCTAGGGGCTGGCCTG

TTTGCCATGCTGCTCCTTGCTCCGGCCGTCCTGGCCTCCGACCCTGACCCTCTCCAGGAC

TTCTGCGTTGCCGACCTCGATGGCAAGGCGGTCTCGGTGAACGGGCATTCATGCAAGCCC

ATGTCGGAGGCCGGCGACGACTTCCTCTTCTCGTCCAAGCTGGCCAAGGCCGGCAACACG

TCCACCCCGAATGGCTCGGCTGTGACGGAGCTCGACGTGGCCGAGTGGCCCGGTACGAAC

ACGCTTGGTGTGTCCATGAACCGTGTGGACTTCTCACCAGGGGGCACCAACCCACCTCAC

ATCCACCCGCGTGCAACCGAGATCGGCATCGTGATGAAAGGTGAGCTCCTCGTTGGTATC

CTTGGCAGCCTCGACTCTGGAAACAAGCTCTACTCAAGGGTGGTGCGTGCTGGAGAGACA

TTCCTCATCCCACGCGGCCTCATGCACTTCCAGTTCAACGTTGGTAAGACGGAGGCCTCC

ATGGTTGTCTCCTTCAATAGCCAGAACCCTGGCATCGTCTTCGTGCCGCTCACGCTCTTC

GGTTCCAACCCGCCAATCCCCACGCCGGTGCTCACCAAGGCTCTAAGGGTGGAGGCCGGG

GTCGTCGAACTTCTGAAGTCCAAGTTCGCTGGTGGGTCTTAATCCTTGGGAGCCTGCCCT

GAAATGGTCTTAGTATATAATTGGATATATGCATGCCAATGAAATTTAATAATTGTTCAG

CAGAAGACACGTATTCAAGCTTCTAGCTAAGCTCGCATGCAGTTGTAATAAGATTGAATA

AGTTAGCCTCGCGGTTTAGCCTTCAGAGCCAATACGAGGAATCAAAATGTACTACTTTTT

ATTGTCGTATTTG

>Traes_4DS_90830E0B2

CACCATATGCCTCAGCACTAACTCATCCACCACAGCTTAGCAGCAACCACCAGTGCCTTG

GACACACTCGATCATAAACTCTAGCTGATCAATCCTACCTAAGCTTGTTCCGTAGCAAGC

AATGGGCTGCTCCAAACTCATAGCTGGCATGTTCACCATGCTCCTCCTTGCTCCCGCTGT

CCTGGCCACCGACCCTGACCCTCTCCAGGACTTCTGTGTCGCCGACCTTGACGGCATGAC

GGTCTCGGTGAACGGACACCCATGCAAGCCCATGTCGGAAGCCGGCGACGACTTCCTCTT

CTCATCCAAGCTAGCCAAGGCCGGCAACACTTCCACCCCGAACGGCTCGGCCGTGACAGA

GCTCGATGTGGCCGAGTGGCCCGGCACCAACACTCTGGGCGTGTCCATGAACCGTGTGGA

CTTCGCGCCTGGAGGCACCAACCCGCCACACATCCACCCGCGCGCAACCGAGATCGGCAT

TGTGATGAAAGGTGAGCTCCTTGTTGGAATCCTCGGCAGCCTCGACTCCGGAAACAAGCT

CTACTCCAGGGTGGTGCGTGCTGGAGAGACGTTCCTCATCCCGCGCGGGCTCATGCACTT

CCAGTTCAACGTCGGTAAGACAGAGGCCTCCATGGTCGTCTCCTTCAACAGCCAGAACCC

CGGCATCGTCTTCGTGCCACTCACGCTCTTCGGCTCCAACCCACCGATCCCGACGCCGGT

GCTCACCAAGGCACTCCGGGTGGAGGCCGGGGTTGTGGAACTTCTCAAGTCCAAGTTCGC

TGGTGGATCTTAATCCGGGGGAGCCTACCCGAAATGATAAATATATAATTCAATATATAA

ATGCTAGTGAAATTTAATATTTCTCAGCAGAAGATATGTATTCAAGCTTCTGGTTATGAA

CCTCGCATGCGGTTGTAATAAGATTGAATAAGTTAGCCTCGCGGTTCAGCCTTCAGAACC

AATACGAGGAATTGAAATGTGCTACTTTTTATTGTCGTCTTTGTTATTTCATAGAACAGA

ATATATAATTATCATTTTTGTAAATTTGTCCGTTTACCCTTTTGTAAGTCATACATGTTC

TAAAGTTTAGTCGCGGCAGCTTGGTGCGCAATCTCCAACCCATTTAGAAACATGTTCTCA

AGTTACCGAAATATATTGTAAAATGTAAAATGTATCGGTTCAATAATGCATTGTAGCACT

CTGCAAAATGTACAAAAAACAAGAGAGTG

>Traes_4DS_B5A1413E1

CTTAGCAGCAACCACCAGTGCCTTACACACTCTCGATCAACAAACTCTACCCGATTAGTT

GCATAGCAAGCAATGGGGTGCTCCAAAACCCTAGTAGCTGGCCTGTTTGCCATGCTATTC

CTAGCCCCGGCCGTCCTGGCCACCGACCCTGATCCTCTCCAGGACTTCTGCGTCGCCGAC

CTCGACGGCAAGGTGGTCTCGGTTAACGGGCACACGTGCAAGCCCATGTCTGAGGCCGGC

GACGACTTCCTCTTCTCGTCCAAGCTGGCCAAGGCCGGCAACACGTCCACCCCGAACGGC

TCCGCCGTGACGGAGCTCGACGTGGCTGAGTGGCCCGGTACGAACACGCTGGGTGTGTCC

ATGAACCGCGTGGACTTTGCGCCTAGAGGCACCAACCCGCCACACATCCACCCGCGTGCC

ACCGAGATCGGCATCGTGATGAAAGGTGAGCTCCTTGTGGGAATTCTCGGCAGCCTCGAC

TCCGGGAACAGACTCTACTCCAGGGTGGTGCGCGCCGGAGAGACCTTCCTCATCCCCCGT

GGCCTCATGCACTTCCAGTTCAACGTCGGTAAGACCGAGGCCTCCATGGTCGTCTCCTTC

AACAGCCAGAACCCCGGCATCGTCTTCGTGCCGCTCACTCTCTTCGGCTCCAATCCTCCT

ATTCCGACGCCGGTGCTCACCAAGGCGCTCCGAGTGGAGGCCGGGGTCGTGGAACTTCTC

AAGTCCAAGTTCACCGCTGGGTTTTAATTCCCGGGAGCCTTCCCTGAAATGATCAATATC

TAATTCAATTTATGCATGGTAGCAAGATTTAATAATTCTCAGCAGAACACATGTAGTCAA

GCTTGGGATTAATTTGGCATGCAGTTGTAATAAAATTGAATAAGTTAGCCTCAGGGTTTA

GCCTTCAGAACCAATATGAGGAATCGAATGTACTACTTTTTATTGTCGTCTTTGTCCTTT

CATTGGACGGAATATATAATTAGCATTTTCGTATATTTGTCCGATTGCCTTCTATATGTC

AAAACATGTGCAAAG

>Traes_7DL_CD8514472

CCGGTGCCTCCTACCGTGACCGTGTTCAAGATCCCCAAGACAGTGAATCAAGGCGCCGTA

GACCTCATCGACAAGTGGCAACTGGTCGCGCCGGCCCTTCCCGCCGACCTCATGATCCGC

ATCCTCGCTATGGGGGACACCGCGACGTTCGAGGCCATGTACCTGGGCACCTGCAAAACC

CTGACGCCGCTGATGAGCAGCAAATTCCCCGAGCTTGGCATGAACTCCTCGCACTGCAAC

GAGATGCCCTGGATCAAGACCATCCCCTTCATCCACCTTGGCAAGCAGGCCACCCTGGTC

GACCTCCTCAACCGGAACAACAGCTTCAAACCCTTCGCCGAATACAAGTCGGACTACGTC

TACCAGCCCGTCCCCAAGCCTGTGTGGGCGCAGATCTTATACGGCTGGCTCGTGAAGCCC

GGAGCGGGGATGATGGTCATGGACCCCTATGGCGCCACCATCAGCGCCACCCCCGAAGCG

GCGACGCCGTTCCCTCACCGCAAGNTCCTCTTCAACATCCAGTACGTTAGCTACTGGTTC

GCCGAAGCAGGCGGCGCCGCGGCGCTGCAGTGGAGCAAGGACATGTACAAGTTCATGGAA

CCTTACGTGAGCAAGAACCCCAGGCAGGCATACGCCAACTACAGGGACATCGACCTCGGC

AGGAACGAGGTGGTGAACGACATCTCAACCTACAACAGCGGCAAAATCTGGGGCGAGAAG

TACTTCAAGGGCAACTTCCAAAGACTCGCCATTACCAAGGGCAAGGTTGATCCTCAGGAC

TACTTCAGGAACGAGCAGAGCATCCCGCCGCTGGTGGGGNNNNNNNNN

>Traes_5DL_A7D264077

CTAGCAACGCAGGCAGCCATGGAGCCGTCCTCGACGGCAGGGCTGCAGCTGGGGCCGCTG

TCGGGCAGCCGCCGCAGCGCCAGCGCGGCCTCCTGGGGCTCCCGCCGCTCCGGGTCCATC

TCGCACTCCTTGCGCCAGCAGGCGGGCGCGGACGACCCCTTCGGCCGCGCCGCGTCGCGG

CAGGGCCACGAGGACGACGAGGAGAACCTGCGCTGGGCCGCGCTCGAGAAGCTGCCCACC

TACGACCGCATGCGCCGCGCCGTCCTGTCCAACCACGCCGGCGTCGATGGCGCCGACGGT

GCCGCTCACGAGCTGCAGGGACTGGTGGACATCAACCAGCTGGCCAGCGGCGAGGCCGGC

AGGGCGCTGCTGGAGAGGGTGTTCCAGGACGACAGCGAGCGCTTCTTGAGGCGGCTCAGG

GACCGGGTGGACCGGGTGGGCATCGAACTGCCGGCGATCGAGGTCAGGTACCAGGGCCTC

TCCGTCGAGGTCGACGCCTTCGTCGGCAGCCGCGCCCTCCCCACGCTCTGGAACTCAGCC

ACAAACTTCCTGCAGGGTCTTGTGGGCCGACTTGCTTCCTCCAACAAGAGGACCATCAAC

ATACTCCAGAACGTCAACGGCATCATCAAACCATCAAGGATGACCCTTCTGCTTGGACCC

CCATCTTCAGGAAAGAGCACATTTATGCGAGCCCTTACTGGGAAGCTCGACAAAGCGCTC

AAGGTGTCTGGCAGCATCACTTACTGTGGCCATACATTTGAGGAGTTCTACCCTGAAAGG

ACCAGTGCGTATGTTAGTCAGTACGATCTCCACAACGCAGAGATGACCGTAAGAGAGACA

CTGGATTTCTCCAGGCGGTGCTTAGGCGTCGGTGCCAGATATGACATGCTCGCTGAGCTT

GCTGTCAGGGAGCGCGAAGCTGGCATAAAGCCAGATCCCGAGATTGATGCTTATATGAAA

GCTACTGCGGTGCAAGGGCAGGAGAGTAATATTGTTACCGATCTTACTCTCAAGGTTCTT

GGGCTTGACATTTGTGCCGATATGCCCATTGGTGACGAGATGATCAGAGGAGTTTCTGGC

GGGCAGAGGAAGCGTGTAACAACTGGGGAGATGTTAACAGGACCTGCAAGGGCTTTGTTC

ATGGATGAAATTTCCACTGGTTTGGACAGCTCTAGCACATTTCAGATTGTGAAATACATA

AGACAATTGGTCCATGTGATGAATGAGACCGTGATGATCTCCCTCCTACAACCACCGCCA

GAGACCTACAACCTGTTTGATGACATTATTTTGCTATCAGAAGGATACGTAGTGTACCAT

GGGCCACGCGAAAACATCTTGGAATTCTTTGAATCTGCTGGTTTTCGATGCCCCGAAAGG

AAAGGAATTGCTGACTTTCTTCAAGAGGTCACTTCCAAGAAAGACCAGCAACAGTACTGG

TACCTTGACCAGGAGCAGTATCGTCACGTGCCTGTCCCAGAGTTTGCTGAACGTTTCAAG

TCATTCCATGTAGGCCAGCAGATGCTCAAGGAGCTGCAAATTCCTTTCGACAAGTCCAAA

ACTCATCCTGCTGCGTTGACCACCAATAAGTATGGGCAATCCAGCTGGGAGTCATTCAAG

ACAGTGATGTCAAGAGAGAAGTTACTGATGAAGCGCAACTCCTTCATCTACATCTTCAAG

GTTGCCCAGTTGGTCATCCTTGGGCTCATTGCCATGACTGTGTTCCTCAGAACAAAGATG

CCACATGGGCAGTTTTCTGACAGCACCAAATTCTTTGGTGCTTTGACTTTCAGTTTAATG

ACCGTCTTATTCAATGGGTTTGCCGAGCTACAATTTACTATTAAAATGCTTCCTACGTTC

TACAAACAGAGGGATTTCTTGTTCTTTCCTCCATGGACCATTGGACTGGTAAACATCATC

TCAAAAGTTCCTGTTTCGCTTGTGGAGTCCATAGTATGGGTCGTCCTCACGTACTATGTA

ATGGGCTTTGCACCTGCTGCAGGAAGGTTCTTTCGTATGCTTTTAGCTTTTTTCGCTACT

CACCAAATGGCAATGGGTCTGTTCCGATTTCTTGGTGCTGTTTTGAAATCAATGGTTGTG

GCCAACACCTTGGGGATGTTTGTGATCCTTCTTATTTTCCTATTTGGAGGCATTGTCATA

CCTAGAGGTGACGTCCGACCATGGTGGATTTGGGCTTACTGGGCATCTCCTATGATGTAC

AGCCAGAATGCGATATCCGTCAATGAATTCCTCTCAAGTAGGTGGGCCAACCCAAACAAT

AATACATCTATCGATGCACCAACAGTAGGCGAGGCTATTCTTAAAGCCAAAGGCCTTTTT

ACTAGAGAGTCGGGATTTTGGGTTTCCATTGGAGCCATTGTAGGATTCATTATTTTATTC

AACATACTGTACCTTCTGGCACTTACGTACTTGAGCTTTGGCAGCAGCTCAAACACAGTT

TCAGACGAGGAGAATGAGAATGAGACAAACACTTCAATACCCATAGGTGAGTTCTTAAAT

GAAGCCACAAATCCGCCAACTCGGTCACAAATCACCTTGCCTTTCCAGCCTCTTTCACTT

TCTTTCAACCATGTAAACTACTACGTGGACATGCCTGCAGAAATGAGAGAGCAAGGATTC

ACAGAAAGTCGTCTCCAGTTGCTCTCTGATATCAGTGGTGCTTTTAGGCCTGGTGTTCTG

ACAGCATTAGTTGGTGTGAGTGGAGCTGGGAAAACCACTCTAATGGATGTCCTGGCAGGA

AGGAAAACTAGTGGATCTATTGAAGGAAGTATCACCGTCTCTGGTTACCCTAAAAAACAA

GAAACTTTTGCCCGCGTGAGTGGCTATTGTGAACAAACTGATATCCATTCACCAAATGTT

ACTGTGTATGAATCCATTCTGTACTCTGCTTGGCTGCGTCTTTCCTCAGATGTTGACGAA

AATACGAGAAAGATGTTTGTGGAGGAAGTCATGACTCTTGTGGAGCTTGATGTGTTGCGT

AATGCTATGGTTGGTCTCCCTGGAGTGGACGGGTTATCGACTGAACAAAGAAAGAGACTG

ACAATTGCCGTGGAGCTGGTAGCAAATCCTTCAATCATATTCATGGATGAGCCAACTTCT

GGTCTTGATGCTAGAGCCGCGGCAATTGTAATGCGGGCGGTGAGAAATACAGTGAACACT

GGGCGAACTGTGGTTTGCACTATCCATCAACCCAGCATCGATATATTCGAGTCTTTTGAT

GAGGTTCTCTCTCTCTCTCTCTCTCTCTCTCTCTCACAC

>Traes_1AS_231DA1EB5

CATGTAGCAGAGCAGAGCACATTCATACTCTTCCCCATGCCTCTCCTCTACATATTAGTT

CTTGGGCTTCTCCTCTCACACACTCCACGCTGCTGCTCTTCAGCACCTGCGAGAGATACC

CTCACTGAAGGCCAAGTGCTTGCCGTCGGCGACAAGCTCGTCTCGACGAACGGCAAGTTT

GCGCTCGGCTTCTTCCAGCCAGCAGCGAGCACCGTCAGTAAGTCCCGGAACTCCACCGGC

TCCAGCTGGTACCTTGGCATATGGTTCAATAAGATCCCGGTTTTTACTGTTGTGTGGGTT

GCTAATCGGGAGGAGCCCATCCCCCACCTCAACATGAACTCAACAAAACTCAAGTTCTCG

AGTGATGGCAAGCTTGTCATTGTCACAAACCGTGCTGATGCTGTCACTGAATCACTTGTT

TGGTCCACTCATATTGTGAATAGGACACAATCCAGTAGCGTAAACACCAGCACCTCTAGT

GTCGTTGTTCTCTTGAACAGTGGAAACCTTGCCCTCAAAGTCACAGATAGCCCATCATCT

GACCTACTGCTGTGGCAGAGCTTCGACTACCCAACAGATGTCGGGCTTCCTGGCGCCAAG

TTGGGCCGGAACAAGGTCACTGGTTTCAGTCGCCGGTTCATATCAAAAAAGAGCCTCATT

GATCTGGGTCTTGGCTCATACGGCATGGAAGTAGAAAACACCAGCGGGATCGTCCTCAAG

CGCCGCAACAACCCCGTGGTAAAGTATCTGATTTATGCAACCTCGGGATCATCATCTTTG

ATACCAATGCTCAAGTCACTTCTAGATTTAGATCCTCGGACCAAAGGTTTGTTTAACCTA

ACATATATCAATAATAACCAGGAGGAGTACTACATGTACACTTTATTGGATGAATCATCG

TCTTCCATCTTTGTCTCACTAGACATCTCTGGCCAGGTTAAGCTGAATATTTGGTCACAA

GCCAACCAGTCTTGGCAAACCATATATTCCGAGCCTGCCGATCCCTGCAGCCTGCCTGCC

ACGTGCGGACCTTTCACGGTCTGTAACGGCATTGCGCATCCATCATGTGTCTGTATGGAG

AGCTTCTCCCATAAGTCACGGCATGATTGGGAGTTTGAAGATCGAACAGGAGGGTGCATT

AGAAACACACCATTACATTGCAGCACTAGTGGTAACAACAAAAACATGACAAGTTCAACA

GACATGTTCCACCCCATTGCTCAAGTTGCATTGCCCTACAACCCACAAATTATAGATGTT

GCTACCACTCAGAGCAAATGTGAAGAAGCATGTCTCGGTTCTTGCTCCTGCACTGCTTAT

TCCTATAACAATAGCAGATGCTCTGTCTGGCATGGGGAATTGCTTAGCGTAAATCTGAAT

GATGGCATTGAAAATAATTCTAAAGATGTTCTTTACCTTCGCCTTGCTGCCAAAGATTTG

CTACCAGGTTCCAGAAAAAGGAAAAGAAAACCAAAGGTCGGAGTTGTTACTATTGTAAGT

ATTATTGGTTTTGGGTTACTAGTGGTCATGCTGTTGTTATTGATTTGGAGGAACAAATTC

AAGTGCTGTGGTTTGCCTATATATGACAATCTAGGTAGTGCTGGTGGAATTGTAGCCTTC

AGACACACTGACTTGGTTCGTGCTACTAAAAGCTTTTCAGAAAAGCTTGGAGGAGGTGGT

TTTGGTTCTGTATACAAAGGAGTCTTAAATGACTCGACTAGTATAGCAGTGAAAAGGCTA

GATGGTGCCCGTCAAGGAGAGAAGCAATTCAGGGCCGAAGTGAGCTCAATTGGACTGATC

CAACACATAAATCTAGTCAAATTGATTGGTTTCTGCTGCGAAGGTGATCACAGATTACTT

GTGTACGAATACATGTTAAATGGATCTCTTGATGGTCATCTATTTAAGACGAGCAATGGA

AATGTTGCCGTCCTAAATTGGAACATCAGATATCAGATAGCCCTAGGAGTTGCTAGAGGA

TTGTCCTACTTGCATCAGGGTTGCCGCAAGTGCATCATACACTGCGATATTAAGCCAGAG

AACATACTTCTGGATGCATCATTTGTTCCTAAAGTTGCAGACTTTGGGTTGGCAGCATTT

GTGGGAAGGGATTTCAGCCGAATTCTGACTTCATTCAGAGGAACTGTGGGTTATCTTGCT

CCAGAGTGGCTTACTGGAGTGGCGATCACACCGAAAGTCGACGTTTACGGCTTCGGCATG

GTGCTGTTTGAAATCATATCAGGAAGGAGGAATTCCTCACCTGAAACATCATACAACACT

AGCAGCAAAAACAGTGACCAGAATATTGATTACTTCCCTGTGCAAGCCATCAGCAAGCTT

CACGGTGGAGATTTGAAGAGTTTGGTTGATCCACGGTTACATGGTGTATTCAATTTGGAA

GAGGCTGAAAGGATTTGCAAAGTTGCATGTTGGTGCATCCAAGATAATGAGTTCGATCGG

CCAACAATGGGTGAAGTTGTCCACGTTCTCGAGGGTCAACAGGACATTGATGTTCCTCCA

ATGCCAAGATTGCTTGCAGCTATAACAGAACAATCTGGTGCTGCAACTTCAATGTAA

>Traes_4AL_CA650B221

ATGAAGCTGTCGCTCACCTTCATCCTCCTCCTCTCAGGCATCAAGCAGTGCTGGTGGTGT

TCGGGGACGCCCAGGCGACCTGCGACCTCGTCCGGTGCAAACAGGGAGGGCACATCACCT

GCAAGAACTACCCCGGCCAGAAGCTCGACGGCTGCGCCTGCGTGTGCGCGCCCAAGGACG

GCAAGCACTGCGTGCTCCGCCTCGACGACGGCTCGACCTACGACTGCCCCAAAACCAAGT

GCTGA

>Traes_2DS_06941F4A8

TTTGGAAAGAGGAGATTACAACATGTCATCCTTGTTGTTGGGACACTATTCGTCATAGTC

GTCGTGCTTGCTTGTGTTCGCAGACAGAGGAAAAGGATCAAGGCGAATAAGGAACAACAA

GATAACGCAGGAGAGGGCATGAACTATATTAGTCTGGAAGTGTTAAGAGCTGCAACATCC

AATTTTTCTATAAACAATAAACTAGGAGAGGGAGGATTCGGAGAGGTTTTTAAGGGTGAA

TTGCAGAATGGAATGAAAATAGCCGTGAAAAGGCTGTCGAATAACTCAGCTCAAGGGTTT

GATGAGCTAAAAAATGAGCTAGTGTTAGCTAACAGACTTAAGCACAAGAATCTGGTGCCG

CTTCTTGGTGTTTGCTTGCAAGAGAAACTGGTAGTGTATGAATATATGCCTAATGGAAGC

CTACACACGAGCCTTTTCAATTCGGAGAAGGCACATCAATTGGACTGGATGAAAAGAAAG

ACAATCATCTCTGGAATTGCTCGAGGTCTATTGTATCTCCATGAGGAGTCTCGTCTAAAA

GTCATACACCGTGATCTGAAGCCAAGCAACGTCTTACTGGACCTGGAAATGAACGCTAAA

ATTTCAGACTTTGGTTTGTCTAGAGCTTTCGGTGAAGATCAATCGATCGATATAACAAAA

CGCCCTGTTGGTACACTTGGATACATGTCTCCGGAGTATGCATATCGCGGTCAAGTCTCT

ACAGAGTCAGACATGTACAGCTTCGGTGTCATGGTTATAGAGATTGTGACTGGTCGGAGG

AACTATAGATCACTTGACGATGATACTGCCTCCAGATATCTGCCCAGCTACGTATGGGAA

AAGTGGAGAGCTGGCTCGATGGAAGAGGTGGTAGACCCTTCCCTAGGCGGGCGTTACCCA

AAGAGCGAGTTGCTAAACTGCGTGCAAATCGGGTTGCTATGCCTTCAAGTCAATCCCAGC

TCCAGACCGGACGCGTCAGAGGTGGTCCTCATGCTCGACGGCCACTCCACGTCCATGACT

ATGCGGACTCCCTCCAGGCCAGCGTTCTGCTTCCCACGACCTGGCGTTGTTAACCCGGCG

CTCGGTTATGCCACGACCAGCGGCCAGCTGCCTACCACCGTTTCAGACAACGGCATGACA

ATTTCCGATCTCCAGCCTAGGTAG

>Traes_5BL_8A4A33744

GCGGAGACCTGGAGTGCGATGCTTGCGTCCACGGCGGCTAGTTACTGCCAGAGAGGAGGT

CGATAAGTAGCACTCCCGGAGGATAACGGTGGGATTAAGCTTGTGGCCGCGGGCTTCTTC

TTCTTCTTCTTCTTCTTCTTCGCCTGGGGGCTTTGTTGATGATTCCGCTGTAAGTTTTCT

TCAGATTCCAGCAATCTTTTTACCTGGTGTCGGATTCGGCCGCGGTGTCTTGTGTGGCTA

GCTAAGTGGTTTTGAAGTTGGGGATGAACTGGAACCTGGAACTGGAAGCAACTTGGACTA

GAACCCTGTAGAATGAGTGAGTGATGGGTTAGTATGCGTAGGCGTGTTAGTGGCTTTGTT

GGATGCGCCCTGAAGGCTTTCTGCTGCTCCAGTTCATGGTCCTTCGTTGCTGCTGCATCT

ACTAGAAAAAGTTGGCCGTGCCTTCGTTTCAACCCCAGGCTGCGCGGCGGAACACTTCAA

ACTTCATTTGCAGCTTTGCAGGGCTATTCTAACCAATTCAAGTTGAAAATCAGAGCTAGT

AGTACATGCTAGACCAGTGACTGAAAATAGTAGAATGCCTTCTTTTCCGAGGGTATACGA

CCAAAGCAAAGCCGATTCTCTTGGAAGGAATTCTGTTCTGGAAGCACAAATATACTGAAA

ACTTAGTCAGCATAATCAAATAGTACTATTTGTTTGGCAGAATGAAAATCCAACGCCTTA

AGATGTTCAATAGTATTCTATATAGGCGGGGATGGTGTGTTAACTAATAGTACAAGCAAT

CATCTGTCTTTAACGAATACATAAGATGGACGGTTTTCCCCTCTTGGATCTGATAAGCCG

TTCCCATTGAAAAATGGGCTGTTTCTGTCAAAAGTTCGAAGTGATCGCATGATCCTAGAA

CTGAATCAATCTTCATCGATTATAGATATACTAGCACCTTCCGTTTCTGAAAGCTTCCAA

TTTTATGGCATATTACAGGACTTGTCAGGCACAACTTTCACAGGGGAAAAAAGGTGCTTG

GAAATGGCCAGTGACACAAACGATGCAGAGCAAGAGCAGGCAACCGAAAATCAGTTGGCA

AGCATCAGCCGCCTCACACCAGACGTCCTGCACGAGATACTTCTCCGGCTTCCGGTCAGC

ACTCTGCAGAGACTCTGCCGGACGTGTCACCAATGGCGCGGGGTGATCAGCGACCCCTGC

TTCATCATGGACCATGCCAATCGTGCACCCGAGCACCTCCTCCTTTTCCTGCCGAGGCTG

GACGCCTCGGCGAGCCTCAAGACCGCCATGCCTGGCCGTGTGAAGCTCTTCGACGAGAAA

TGGTCCGTGTCAACGTGGGCGGCGTCTTCCATGGATCCGGACGACCACCTCTTCGCGTCA

TGCAACGGCCTGCTCTGCTTCTACCGGAAGTACACGCTGAAGATCGTCAACCCCGCGACC

GGCCAGCGCCTGCACCTCTCGAAGCCCGACGGGAGATCGCTCCGTGACCTGTACTACCTC

TATAGTTTCGGGTTCCACCCGGCGACGGGGGAGTACAAGCTCGTCTACTTCCTCCGCGAG

CCCCGGCACGGCCGGTCGTCGGGGCAGCCGTTCCGCTTCGACGCCATCCAGGTGCACACC

CTCGGCGAGGACGGGTGGCGGGACGTCAGGGCGCCGAGGGAGAGCTGCCTGGTGAACCTC

GGGGTCGTCAACGTGGACGGCGCGATGTACTGGATATCGGAGGAGGAAGGGGCGTGCTGC

GGCGCGGCGGTCATGGCGTTCGATCTCAAGGACGAGACCTTTGTGACGTTGAGGCCTCCG

CCGTTGAGAGCGTGCGGCGTGGCGACGGACGGGCCCTGCGGCGCCCCCGCGCTGTCCTAC

TACGTGACGGAGGTGGAGAAGAAGGTTTGTCTGGTGACAGCCCCTTTCAGCAGCAGCGCT

CCCCGGTGGCGCCGCTACAACGCCGAGGTCTCCGGGAGGATGGACGTCTGGACGCTCGAG

AGCCGGGGCGAGGACAGGTGGTCGCTCAAGTACAGCGTCGACCTGTCGCCGTCGGCGCCG

CGGTGCGTCCCGCAGCCGTGCTTCGTCCGCGGGGGGAAGGTTCTGCTGCACGGCCGCGAC

GGCGACGCGTTCTGCCGGGACCTGCGGGGCGACGAGGACGGCGGGGAGGAGGTGAGGCTG

CTGAATTTCAGGCCTTACAGGTACTACGAGACGCAGGCGTACCTGTACAAGGAGACGCTC

GTCCCGTTGGACGTGTACGCCGGGGCGGCCATTGTTCGCACGCCGCACTGGCCTCTCCCT

CCGCCAGGTTCCTCCTGACATGACAGTACTGGCTTTGCAGTGTTGGAGCGCCTTGTGTTC

TGAATTTCTGATTACGGCTCTGCGTTTGCCTTCGGTGGTCATGGTCCAGTATAGGTGTAG

GCAGCAGATGAATGAATTACTTCTGAAACATGAATGAATTATGGTGTAATTCTCGATGGA

GATTTAAGCAACTGGTTATTGTAACATATTCAGAAGTAATAAGTATTTTGTGCACTAAGC

TTTTCTACTTCACTCGTGTAGTGAATAGTGCAAATTCTACCCATGATATATCAAGTGGGA

ACCAATGGAAAATGGCAAAGCCTTCG

>Traes_5BL_C7085F300

CTTGTTCCTCTTGGGCGAAACCCCGGAGCCGCCAGAAGGGGTCGAAGAGCTGACGAGGGG

GGAAGCGGCGGGAAATCGTTTCACCGTCAAGCGGAGAGCCCTCCCATCCCAATCCAATGG

CGGCGACGGCGGAGCTCGTCTGATCCGAATCGCGCTCAAGGGTTTGGTATTTCAGCTGCT

TCGGCATTGGGTGCTCACTGTGGTCACTGAAGAAACCAAATCAATGAAGCAAATAAATGA

GAAATGCATCATAAACCACCTCCCAGGGGACCTCGTTGAGCGGGTGTTTTTCGGACTTCC

AGTGAGCACTCTGTTGACGTGCGTCGGAGTCTGCAAGCACTGGCACAACATCATCCGGGA

TCCACAGTTTGTTGCATTGCACCTCCAGTGTGCGCCAAGTTATGCCCTTCTGTTCTTCCC

GTCAGGTTTGGTTTCAGGCAAGCACTACCCTAGTGATGCTGTCCTAATTGATGAAGCCTG

GTCGCCATCGACATATATAGTGCCAGTGATTGGGCCTGGTGATTTTCTTTTTGGTTCATG

CAATGGCCTTCTTGGCTTATACACAAAGACATCAACGATCAAGATAGCTAACCTTGCAAC

TGGTGAATGTCTACATCTTGGGAAACCTGTGAAGAATCTGAAAGGTGATAATTTCTGTTT

CTATAGCTTTGGATTTCATCCCGTGACAAAAGAATACAAGATTACACACTTCCTTGGTGA

TTGCATTAATGGTCGCCCCCATAATAAAGACAGGTTCAACATCATTCAAGTTTACACGCT

TGGTGATGAGAAATGGAAAGATATCCGAACTCCAGAAGCTCTTAGCTTGATCAGTGTGAG

AAACTCTGGAGTTGTCAATGTTGATGGAAAAATGTATTGGTTAACTGAAGACATGTCAGC

TAGCTGGCAGCATGCAGTTATGTCCTTTGATATCAGGGAAGAAAGTTTTGCAATGATACA

ACTGCCAGCAGCACGCGAAGATCATGATTACTATGGTTCTCGTAAGTTCTTGATCAGAGA

TATAGATGGGAAAATATGTATAGTGACTGCTCAAACTAGTCGTTATGATGCCAGAACTCT

TGTCGGTGAGCTGCAGATCTGGGCACTTGACAACATGGTAGAGCAAAGGTGGAGCCAGAA

GTACAACATTAAGTACCCACCAGATTATATTCTGGGTCCACGTTTTGTTCACAGGGATAG

GATCCTCACACAACGCGGCCGCAATAACGTATGTTCGTACGAGTTGCTTGGTGAGAACTT

CGAGATTGATTCAAGTAAGATGGTGAAGCTGTTAGATTTCAGTCCCTGCAGGCACAACTT

GCAATCTCACAACTGTGTGAAGTCACTTGTACGTTTAGATGTATACAAGAATGCTAAAAT

TGTGTGTAGGCCAAAACAGTGGGAAGGCTGGGAATTGAACAAGTGGGAGGCGTGGGAGCG

TGGGCTCTCTGAGAATGAAAAATTGTGCAGTGTCATTCACCAAGTTGAGCTTAATGGAAT

TGCATGTGCACAACAAAATGGCATATGGTTCAATGAGATACTGCAACATATATTGGATGA

TGCGATTCGACGGGAAATAGGCATGAAAATTAATCAAATATTTCCAAACTTTCCAGACCA

GCAGACAAGACCCCTCCGGCATCTTAATTGTGTGGCACAGAAGCTGGATCAGGACAATTT

AATTGCTCGTATTAATAATAGTAAGACTATTATAAAGGTATAGTGTTTTAAATTTACAAG

CCAGATCATTG

>Traes_2AL_2763280FC

CGGAGGACCAAGGAGGGCGGCGCTCTCCAGAGAGAGCTCATCCGATTCCTTAACGCGGGA

GCCTTCGGCACCGGCACCGACGGCCACGTCCTGCCTGCCGCGGCGACGAGGGCGGCGATG

CTCGTCCGAGTCAATACCTTACTCCAGGGATATTCTGGCATCCGCTTCGAGATCCTGGAG

ACGGTCGCCACACTTCTCAACGCCAACGTGACACCATGCCTGCCGCTCCGGGGCACGATC

ACCGCATCCGGTGACCTCGTCCCGCTTTCGTACATCGCGGGCCTGGTCACCGGCCGCCCA

AACTCCATGGCGACCGCTCCAGATGGCACAAAGGTTAATGCTGCTGAGGCATTTAAGATC

GCCGGCATCCAGCATGGCTTCTTCGAGCTGCAGCCAAAGGAAGGCCTAGCCATGGTGAAT

GGCACGGCAGTGGGCTCAGGGCTTGCATCCATGGTGCTTTTCGAGGCTAACATCCTTAGC

CTCCTTGCTGAGGTCTTGTCAGCCGTCTTCTGCGAGGTGATGAACGGCAAGCCGGAGTAC

ACCGACCACTTGACCCACAAGTTGAAGCACCACCCCGGGCAGATCGAGGCTGCCGCCATC

ATGGAACATATCCTTGAAGGCAGCTCCTACATGATGCTCGCGAAGAAGCTCGGTGAGCTT

GACCCATTGATGAAGCCAAAGCAAGATAGGTATGCACTCCGCACATCGCCACAGTGGCTT

GGCCCTCAAATTGAGGTCATCCGTGCTGCCACCAAGTCGATAGAGCGGGAGATCAACTCC

GTCAACGACAACCCACTCATCGATGTCTCCCGTGGCAAAGCTATCCATGGTGGAAACTTC

CAAGGCACGCCCATCGGTGTGTCCATGGACAACACCAGGCTTGCCATTGCTGCGATTGGC

AAGCTCATGTTTGCCCAGTTCTCAGAGTTGGTGAATGACTTCTACAACAACGGCCTTCCT

TCAAACCTCTCCGGTGGGCGCAACCCAAGCTTGGACTATGGCTTCAAGGGTGCCGAGATT

GCCATGGCCTCCTACTGCTCCGAGCTTCAGTTCTTGGGCAACCCTGTGACCAACCATGTC

CAGAGTGCGGAGCAGCACAACCAAGATGTCAACTCTCTTGGTCTCATCTCCTCAAGGAAG

ACCGCCGAAGCCATTGACATACTCAAGCTCATGTCCTCAACGTTCTTGGTCGCGTTGTGC

CAGGCTATCGACCTCCGCCACCTTGAGGAGAATGTCAAGAATGCTGTCAAGAGTTGTGTG

AAGACTGTGGCTAGGAAGACACTGAGCACCGATACCAATGGCCATCTCCACAATGCGCGC

TTCTGCGAGAAGGACCTTCTGCTCACAATCGACCGTGAGGCGGTGTTTGCGTACGCAGAC

GATCCTTGTAGCGCCAACTACCCACTGATGCAGAAGATGCGTGCAGTTCTTGTGGAGCAT

GCCTTGGCAAATGGTGAGGCCGAGCGCGACGTGGAAACGTCAGTGTTTGCCAAGCTTGCC

ACGTTTGAGCAGGAGCTTCGTGCGGTGCTGCCAAAGGAGGTTGAAGCTGCCAGGAGCGCC

GTGGAGAATGGCACTGCCGCACAGCAAAACCGTATTGCCGAATGCCGGTCGTACCCACTC

TACCGATTCGTGCGCAAGGAGCTTGGAACGGAGTACTTGACCGGAGAGAAGACGCGGTCT

CCTGGCGAAGAGGTGGACAAGGTGTTC

>Traes_2BL_EE22FBE18

CACCAACCCACTGTCTCACACCGTACCTACTACTCGGCTGCGCCTGCGCTACCTATTTAA

TCCCTCCCCTCCCTCCATTCCCCTCCAAGAAGAGCTTCAGCTTCATCTGCAGCTACAGCT

CCTCTTCAGCACAACCCTTTCTTCAGGACACAGATCAATCCAGATACACATACGCCGCCT

GCCGAGTGCCGACGTCTAGCTGCCAGTTTGCTTTCAGAATCCAGATACACATACACTTGC

TTTAGTACGTCTGTGCATATTTCGATGGAGTGCGAGAACGCACACGTTGCCGCCAACGGC

GATGGCTTGTGCATGGCGCAGCCGGCGCGGGCCGACCCGCTCAACTGGGGGAAGGCGGCG

GAGGAGCTCTCGTGTAGCCATTTGGATGCGGTGAAGCGGATGGTGGAAGAGTACCGACGG

CCCGTGGTGACCATGGAGGGCGCCAGCCTGACCATCGCCATGGTCGCCGCGGTGGCTGCC

GGCAGCGACACCAGGGTCGAGCTCGACGAGTCCGCCCGCGGCCGCGTCAAGGAGAGCAGT

GATTGGGTCATGAACAGCATGATGAACGGCACCGACAGTTACGGCGTCACCACCGGCTTC

GGCGCCACCTCTCACCGGAGGACCAAGGAGGGCGGCGCTCTGCAGAGAGAACTCATCCGA

TTCCTTAACGCAGGAGCCTTCGGCACCGGCACCGATGGCCACGTTCTGCCTGCCGCTGCG

ACCAGGGCGGCGATGCTCGTCCGAGTCAATACCTTGCTCCAGGGATATTCAGGCATCCGC

TTCGAGATCCTCGAGACGGTCGCCACGCTTCTCAACGCCAACGTGACACCATGCCTGCCG

CTCCGGGGCACGATCACAGCATCCGGTGACCTAGTCCCGCTTTCGTACATCGTCGGCCTG

GTCACCGGCCGCCCAAACTCCATGGCGACCGCTCCAGATGGCACGAAGGTTAATGCCGCT

GAGGCATTTAAGATCGCCGGCATTCAGCATGGCTTCTTCGAGCTGCAGCCAAAGGAAGGC

CTAGCCATGGTGAATGGCACGGCAGTGGGCTCGGGGCTTGCATCCATGGTGCTTTTTGAG

GCTAACATCCTTGGCCTCCTTGCCGAGGTCCTGTCAGCCGTCTTTTGCGAGGTGATGAAC

GGCAAGCCAGAGTACACCGACCACTTGACCCACAAATTGAAGCACCACCCCGGACAGATT

GAGGCTGCTGCTATCATGGAGCATATCCTGGAAGGCAGCTCCTACATGATGCTCGCGAAG

AAGCTCGGTGAGCTTGACCCATTGATGAAGCCAAAGCAAGATAGGTATGCACTCCGCACA

TCGCCACAGTGGCTTGGCCCTTAGATTGAGGTTATCCGTGCTGCCACCAAGTCGATCGAG

CGGGAAATCAACTCCGTCAACGACAACCCACTCATCGACGTCTCCCGTGGCAAAGCTATC

CACGGTGGCAATTTCCAGGGCACACCCATCGGTGTGTCCATGGACAACACCAGGCTTGGC

ATTGCTGCGATTGGCAAGCTCATGTTTGCCCAATTCTCAGAGCTGGTGAACGACTTCTAC

AACAACGGTCTGCCTTCAAACCTCTCCGGTGGGCGCAACCCAAGCTTGGACTATGGCTTC

AAGGGTGCCGAGATTGCCATGGCCTCGTACTGCTCCGAGCTCCAGTTCTTGGGCAACCCT

GTGACCAATCATGTTCAGAGCGCGGAGCAACACAACCAAGATGTCAACTCTCTTGGTCTC

ATCTCCTCAAGGAAGACCGCCGAGGCCATTGACATACTCAAGCTCATGTCCTCGACGTTC

TTGGTCGCGTTGTGCCAGGCCATCGACCTTCGCCACCTTGAGGAGAATGTCAAGAATGCT

GTCAAGAGCTGTGTGAAGACTGTGGCTAGGAAGACACTGAGCACCGATACCAATGGCCAT

CTCCATAACGCGCGCTTCTGCGAGAAGGACCTTCTGCTCACAATCGACCGTGAAGCCGTG

TTTGCATACGCAGACGATCCTTATAGCGCCAACTACCCACTCATGCAGAAGATGCGTGCA

GTTCTTGTGGAGAATGCCTTGGCAAATGGTGAGGCCGAGCGCGACGTGGAGACGTCGGTG

TTTGCCAAGCTTGCCACGTTCGAGCGGGAGCTTCGGGCAGTGCTGCCAAAGGAGGTTGAG

GCTGCCAGGAGCGCCGTGGAGAATGGCACTGCCACACAACAAAACCGTATTGCCGAATGC

CGGTCGTACCCCCTCTACCGATTCGTGCGCAAGGAGCTTGGAACGGAGTACTTGACCGGA

GAGAAGACGCGGTCTCCTGGCGAAGAGGTGGACAAGGTGTTCGTTGCCATGAACCAGGGC

AAGCACATCGATGCGCTCCTTGAGTGCCTCAAGGAGTGGAACGGCGAGCCCCTGNCTGCC

TCTCTGCTAAATAGAGGATCGAGAAAGTGAAGAGTAGTGTGCTTCAGATTTCTGAAGGCT

CTGATGATAATACTGTTTTTTCATTGTATATTCTAAAAGTTGATGTTTACAATGTTCTTC

TAGAGCTGCCAATATATTGCCAAAGATTGCAATTGCATGACTTGGTAGTGTTGGGTAGCC

AGTAGAACTTTTATGATGTACGTAAGTTAAAAAGGCAATGTGTGTTAAATTTTCATGATA

AATTTACTGGCTCCATTTTTTGAGATCAACCGGTCCTTGTGACAATAGTCCAAGAACTAA

CTATTGGAAATTCTTATGGTGTGTGTACGGCGATTCGGAAAGTAAAACTCGTGCCTACCT

GGTCACCGGTCGGTAATAAGTTCTCTAAATTCTTTGAACGGGGACGATGGCTTCTTTGAA

TTCACAATCTCCCATCTAGTTTTTGCAGTTTCGGTATTCTGCTTACTAGTTTCAGTCTGG

ATTTCTTTGCAGGCACACAATGATGTTTAACTTTGTGTTATTCAACTACCGTACCACCGC

ATATCTATGAATCCATTTTTTTGATTATTAATTCACTAATGATCTCAGCCCATAACACAG

GGTACATACACATGAAGTTTCTCTATGCTTCTGAACATTGCACTATTTTTTAGTTCTAGC

CATACTTAGGCAGGAAATATATATGAAGATTGTAAGGTTGCTCAGGCATGTGACGCGTAT

TTATATTCTACTCCCTTTGGTGTTCTTTAATCTACACGCATCAACAGCTCTGTGCGTACA

TTGACCATATAGGAGACGTG

>Traes_2BL_E97474D26

AAAAACCCAAACGGGCACCAACACCAAGCCTGTCGCTGCCGCACGCGCAGGCGCAGCGGA

CAGCGATCCGCGCACGGTCCTGCCCCTGCGCCCTCGTCCCTTCCGCTATATATGGGGCCC

TGCCATGCCATCCCGGCACCGGCCACTCAAAATCCCCAGACTTTCCAAAGCCAAAATCCC

AACCTCCGTCCTCCGATCCCCCTCTTCTTCCTCCTTCTCGCGGCCGAGGGGGTCGTCCAT

CGATGGCGACAATGGGGGCGGGCGAAGCAGTGAAGATGATCTGCGGGACGAAGGTGGAGC

GGGTGGTGGGCACGGGCAAGGCGCCCGGTGCTTGCCCGTCCTGCGGCGGCCCCGTGGTGG

CCACCGACGTGGAGAGCGAGCGGCGCATCCTCTGCCTCCCGCTGTGCCTCAAGAACAAGC

GCAAGTACTCCTGCACCAGGTGCTTCCGCCGCCTCGTCACCGTCTACAGCTAGCCGGCCC

CAAGCCGCTGGTGGTGGTGCCACCGATCCATCCATCCTTCCATCAACTAGCGATGTACTA

CCAAGCAAATCCATTAATCCGCTCGACTTCTTTGATTCCTGTTGGCCGTGCTTACGGCTC

TAGAGTAGCTTGCTAGCCTGGATAATGTAATGTCATGTTGATCCTGTCTTAGTTCTTCTA

CAGTGTTTCTAGGGAGTACTCCTAGTAGCTAAATCTAGCTAATTTGGTGGCCGCGCATCA

CGTGAAGCGATGCAAAACTGGCACTCTGATGTACCAGTATCTTGCAGTAAGCTCTCTGTC

AATGGTGGCGGGGCACCTCCGCTTTAATGCACCGGCCTGGCCAGTATTGGAAATTCGTAA

TGCGCTTCCTTCATTAAGATGGCAATTAACGTACTGGTGGG

>Traes_4DL_7A688107B

CTCCTTCCTTCCTTCCCCGTCGCGTCCAGCAGAAAGAAAGAATCCGGTCATCCAACTCCA

TGATCCATCCAATTCGTCACGTGAAGATGAGATGATCACCTCACCTCCATCGGCCGCCCT

TCCAGCCACCTTCCAATCTCAACCTGATTCCAATCCATGCTGTTGTTATAAATACGTCAC

ACAGTGAAGCTCCTGGTTCTTCCATCAGATCCCCTCTTCTCTCTCTCTCTCTCTCTTCTT

CTTGCTCTAGCACTCTTCGCTGCTCTGTTCCTTCCGGCTAACACCGGCTACGCCCACCCC

ACGGTGGATGGAGGGCGTCGAGGAGATCGTCATCGCCGGCGCCGGGCTCGCCGGCCTCGC

GACGGCCCTGGGATTGCACAGGAAAGGGGTGAGGAGCGTGGTGCTGGAGTCGTCGGCGAC

GCTGCGGGCGTCGGGGTACGCCTTCACCACATGGACCAACGCCTTCCGCGCGCTGGATGC

CCTGGGCGTCGGGGACAAGATCAGGGAGCACCATCTGCTTTACGAGAGGCTGGTGGCCTT

CTCTGCATCCACAGGCGAGGCTGCCGCAAAACTGAGCCTAAAGATGCAGGGCAAAAGCGG

GCCTCACGAGATTCGGTGCGTGAAGCGCAACTTCCTGCTGGAGACGCTGGAGAATGAGCT

GCCGGAGGGCACCATCAGGTACTCCTCCAAGATCGTCGCCATCGAGGAAGAGGGCAACGT

CAAGCTCCTGCACATGGCCGACGGCTCCACCATCAGAGCCAACGTTCTTGTGGGGTGCGA

CGGAGTGAACTCGGTGGTGGCGAGGTGGCTGGGCCTGCCCAAGCCGATCCTCTCGGGGCG

GTCCGCCACCAGGGGCCTCGCCGAGTACCCGGACGGCCACGGCTTCGGCCCCGAGATGCT

GCAGTTCATCGGGCAGGGCTTCCGCTCCGGCGTGCTCCCCTGCTCCGACACCTCCGTGTA

CTGGAACTACACCTGGTACCCGTCCCCGGCGGACGGGGACGCGGAGGAGAGCGTGGACAA

GATGCGGCAGCACGTGCTGGCCAGGCTTCGGGCCGCCAAGATCCCGGCGGAGGCGCTGGA

CGTGATCGAGCGGAGCGAGATGAGCGACGTGGTGTCGTCGCCGCTGCGGTTCCGGTCCCC

GCTGGCGCTGGTCCGGGGCAGCATCTGCCGGGGCGGCGTGTGCGTGGCCGGCGACGCGTT

CCACCCGATGACCCCGGAGCTCGGGCAGGGCGGCTGCGCGGCGCTCGAGGACGGCGTCGT

CCTCGCCCGGTGCCTCGGCGAGGCCTTGGGCGCCGGCGGGCACGGGAGCGCCGAGGCGGC

CTTGGCCAAGTACGCCGGGGAGCGGAGGTGGCGCGCCGTCCGGCTGGTCACGGCCGCGTA

TTTCGTCGGCTTCGTGCAGCAGAGCAGCAACCCGGCGATCAAGTTCCTCAGAGAGAAATT

CCTGTCAGGATTGCTGGCCAGAGTGATGGTCGACATGGCCGACTACGACTGCGGAAAGCT

GTAGCTCTATCATTCCTTCTTCATGGCAAAACGAGAGCGACGAGAGAAAACGAATCAGTT

AAAACGGCATCAATCAAATCGATTTGATGCCTGTATTTCGACATTTTGAGTTAAATAAAG

AAAACAGTTTGATACCTGATAGGATTATTAGTCTAGATGATACCCTCGTCTTTCCTGAAA

AGAACGCCTATAGCTCAAGTTTTTTTTCGCTTTAGCTAAATGTCATCTCTGACTTTTAAG

TTTTGGGTCGGTCGATTTTTTTGACCGTAGATTGCTGCTCAAAGATGCATCCTAGTTGTT

TTCTTCCTGTTGTGTGTTGTGTCATGTATTGGGCTAGACGATTTTTTATTGACCCCTATT

TTGGGTCAGTCGATTTCTTGTTGGGCTGAAGCTTGTTACATGTTTTCCTATTAGGCTGTT

TGCGAGCGTTTATACATCTGTT

>Traes_2BL_21E338A87

GTGTTCGTGCTGGAGGTGACCTGCGGGCGCCGCCCGCTGGGGTGCATCGCGCCCGACGAC

CAGAACGTGCTGCTGGACTGGGTGCAGGAGCACGAGCGCAGGCGCGCGGGGCTCGACACC

GTGGACCCGCGGCTGTGCGGCAAGTACGACGCCGACGAGGCGCGGCTGGCGATCAAGCTG

GGGCTGATGTGCGCGCACCCGCTGCCGGACGC

>Traes_2BL_71E5A51B0

CGGTGAAGCGGGTGTCGCACGACTCCAAGCAGGGGATGAAGGAGTTCATCGCCGAGGTGG

TCAGCCTCGGCCACCTCCGGCACCGGAACCTGGTGCAGCTGCTCGGCTACTGCCGCCGGC

AGGGGGAGCTCCTCCTGGTGTACGACTGCATGCCCAACGGCAGCCTCGACAAGTACCTCC

ACGACAAGACCAAGCCCGTCCTGGACTGGAGCCAGAGGTTCCA

>Traes_1BL_AB3B8444B

TCCAGCTTCTCAAACTGAGTGTTTCATTTTTTTATCTGATCTGTGTATAGTGTTTCATTT

GTGTTGATAGATTTGTTTCCCTCTATTAAAACATTGCTATGAAATACGATTACTGCAGCA

GTAGCAATAGCACCAGATTATGTGATGTGATGAACTTGTTTATGCCCTGTCAGTTTTCTC

TGTTCATTTTATCTCCTTGACATAGTTAGTTTTGTTCATGTAGACTCCATCTGGCGGCAT

GGGCTGGGCATGTTGATGTTGTGAAATGCCTTTGCAAGCACAAGGCTGATGTCGGGGCTG

CAGCAATGGATGACACTGCCGCAATCCATTTTGCTTCCCAGAAAGGTCATTTGGAAGTGG

TGCGTGAGCTGCTGGCGTCGGGGGCCTCTGTGAAAGCAAAGAACAGGAAAGGCTTCACGG

CGTTGCACTTTGCTGCTCAGAACTCCCACCTGGATCTTGTGAAGTATTTAGTGAGGAGGG

GCGTGGACATCACAACAAAGACAAATGCAGGGCAAACGGCTCTACATGTTGCAGAGAATG

ATGATGTGCGTGCTTTCCTGAAAGAGTGTGAGCAGTCACTGAAGAAGGGAGTGGAGCTAC

CATCCGAGAAGAAGGATGATTCTGTAGCTGAGAAGGCTGATGACGGCAAGGTCTCAGGTG

AAGATAGAAAAGATGGTGTTGATGCAGGACAGGGCGAGAAGAGGAAGAGTGAGGAGATTG

GTGCTGGGACGAGGCCGACGGAGGTGAAGAAGGCCAAAGTTTCACTTTCGCATCTTGAAA

ACGACATGGAAGAAGAGGATGAAGCAGACGAATAGAATCGAATAGCCTCCAGAATGCCCT

TTGATGGTGCCAACCTGTTTGCTGTGTTATCCATTGTTCTTTTGGCAATTTTGGAATGTT

GGCAAGCAAGTGTAAGAGTTTTAGCTGTCCATGGGCATGTTATCCGTATCATAGTTGTTG

ACCAGCAGTACTATGTTTTGATCAAGTAGCTTATGATGATGATTTACTACTGATTCCCTT

AAACCAAGATGATTTTACTACTGTGAGTAAGCTTATTAAGTGCTGTGGTTTTTAATTTTG

ATGGAATATTATCTGCTGTCTGTGTTGCAATCTCGATTCCTTTTTTGAGACTGTTGTAAT

CTCAAACAAAATGGGAAGAAAAAAAGACAACGGCCCAACCCAGTTGACGAAGGAATAGGC

CCAAACGGGAAGAAGACAGTAGTCCTAGTGAGTTATCCACTCCTTCCATCTACTCTCTCA

CACTCAGAGGGAGGAGACAGGGAGCACTGCGCCGCCGCCGGCTGCTCCGTAACCCACGAA

GCTAGATAGATAAGCATGGCGGCCACCAGCGCGAGTGCCGCTGCCTCCCTCTCCGTCACG

GCTGCCACTCACCGCCTCAGGCACCGCCAGCTATACGCTTGCGCACGTGGGCCCGCGGCG

CAACCTCATCCCCTCCTCAAGCTGAACCGCCGGAGCTACGCGGTCTCGGCGTCGTCGGCG

GCGGCCATGTCTCCTCTGTCCCTGTGGGAAGGCCAAGGCATCCGGGCGGAGTTGGATGCG

CCCGGAGGAGTGGCCAGCGGTGACGTCATGGGCCTTCTTCTCCGGGAGCGCATCATCTTC

CTCGGCAACGAGATCGAGGACTTCCTCGCCGACGCCGTCGTCAGCCAGCTCCTCCTCCTC

GATGCCATGGACTCCGAATCTGACATCCGGCTCTTCGTCAACTCCCCCGGTGGATCACTC

AGTGCGACAATGGCTATCTTCGATGTAATGCAGCTGGTGAGGGCAGATGTATCCACTATT

GGAATGGGCATAGCTGGATCCACAGCTTCTATAATCCTTGGTGGTGGCGCAAAGGGCAAA

CGATTCGCCATGCCCAACACCAGGATTATGATGCATCAGCCCGTGGGAGGCGCGAGTGGC

CAGGCCTTGGATGTGGAGGTCCAAGCCAAGGAAATTCTGGCTAGCAAGAGGAATGTCATC

CGGCTGATCTCAGGCTTTACGGGGCGTACACTGGAGCAGGTAGAGAAAGACATCGATAGG

GACCGATACATGGGTCCTCTTGAGGCTGTTGATTACGGTATCATCGATGGTGTGATCGAC

GAAGACAGCATTATCCCACTTGAGCCGGTTCCGGAGAGGGTGAAGCCTAAGTACAACTAC

GAGGAAATGTACAAGGACCCTCAGAAGTTCCTTACGCCGCATGTCCCAGACGATGAGATA

TACTAAGCTAGCCTGTCAGTCACATGTATTTCAAACAGCAGGCAGTCGCTGTAGCTTTCG

TCAAACAAAGATGATGTAATCCTTGGCAGTAGACTTTATCTGCCGTTACATTTGGTGTCA

TGGCGTAGCTGTTGTGTTTTGCATTTGACCAAGCAAATGAAAGCACATGGAACATACAAC

TGGTCTAACCAGCTAGCCTCGTCGCATGTACTAGTACTATTTTACACAGCAAGCAACTGC

TGGAGCTTTTGTCAAAACAAAGTTGTAGCACTAATTTTAGCAGTAGACTTTGTTGGCCGT

TGCATTTGGTATGATTGAGAGCTATTATGTTCTGGCTTTTGACCAAGCAAATGAAAGAAT

TGTGTTCTGAACATAGAAATAGGCTCATCAGCCAACCTTGCTGCAAAGAGGAAACCAAAG

AACATGGTGAAATTTATTGACTGCACCCAACTCATGATAGTGGGGAGAAGAATCCAATGT

AGCCAGTAATCCTTTACAAGACACAACAACAATGAATACAAGAAACAATTTCTGGAACTC

TGATACAATTCTCACAACCGCCGCAATGAAGCACGATCATCTCATCAATACATGGCACCG

ACAGTCGGAACCAGGGCTGGGGACACGGCGCCTTTGACTCTGAGGCCAAGCGGGTCCCAG

AGCTGGACAGCACCAGAGCTCCCAGTTTTCAGGTTCACCGGAGCTTCAATCCGGGTAGCA

TGTTCATGGCCATGGCCCTCTGCTGCTAATTTTGCCCTCTTCAATCTGATACGTTCAGCT

CTTGAACCAATTGTCTGACCCAATATCGAGGCAGCTATGGTAAGGGCCATGGCGCTCTTC

GGCATCAGGATGGACTTCCTCAGCATCCCTATGAAAGGCACTGCAGCATGGACCGCCGCA

AACCACTGCACTGAAAACTTTGTAGTGTGCTCCCTCCAGATGCCAAGAGGAACATTTGCT

GCCATACCGAGCAGTGCGATCACCACTACTTTTGTCGGTAGGGGCTGAGGGCGAAGCCCC

TTCGCAAACGCCGTGCGGGATATTGCTGCACGGGCAGCAACAATCGCAGGAGGGCACGTC

AGTTTCATACCAGCTGGGGGTGTCAGCAGCTTTGCGACAAGAGGCACGACTCCACCAATT

GCTCTATATGACTTTGCAAGAGGGCATTGACCATTTTCCAGCCAGTCATTGCTCATCGCT

TCATGATTGTTATTAGACTGGCCTTTATTCTGAAATGAAAGGGAAAGCAAAATCAGGTAA

ATGAATGGCATGCTCCAGTTCTAAGGAATAACCTTATATTCGTTGCTTGTTTTAATATTT

GCTCCATATATGGCAGTTCAAAAATATCATGTGAAGCTTGCATCAAAGATAGATTTGCTT

TCTTTTGGTACTAAAAAATTGGTCTCATCTAAATGCTCGTTCGACAGGATAACGAAACAT

TCTAACACAAAGGATGGTAATTTGAACTAAACATTCTAACACAAATGCACGAGGAAAAGA

GAAACCATAATTACCTGGGGGAGGTTGTTGGGATTCTTCTTGTTGGAGTTCTGCTTATTG

CGCTTGTTTGAGAAATCACCGAAGCTGAAAAAGCCTCCGAATCCCGAGAGACTGATGGTA

GCTGCTTTGGCTGCCAAGGGGTTAAATTCAGGAGTGGGCTTTGGCAGAGGCTTCTCAATA

TGTGCAAAGGCTCCTTCTGAAAGTGGAACAACCCCGTCTCTGCCATGGAAAACTCGAAAC

GCCGTGTCAAAGTTGGGCCCGTCTTCAAAAATTGGACCCTTGGCTCCGGTTGCCTTCACG

AGCAGAAAATGATACAGTTTAGCAAGCAAATAAATAACATACATACACTTATATATTCAG

ATAAAGAGGGGTAGATGGTAAGGACATACCGGAGCGGGGAAATTAATGGATGTCAAGGAA

AAACTTGTAGGCTCGTTGATGTTCCTCAGGAATGGGCATTTATCAATTCCTTGTTGGACA

ACATTACTGTCTTCCGAGCACATTGGGTCGCCGAAAGCTCTTTGGAAAAAGGAATCCATG

TGAACAACAACAGCAACCTAGCCTGTGAGTGAGGAAGCAATATGAGTCAGGAGATGGTAT

ATACCAGAACTGTAAGGGATGCAGCTGCCAATCGTCCTGCGGTGGAGGTCCATGATCGGA

ACTTATGAAATCGATTAAATTCAATCTTCTTTAGCACCGACACAATTGATAGGTCGTGGA

TAGACAGATTAACCCAATACAGTCTGATTAAATTTCATCCCTAGGTGCATATGGTCGGAT

TGGTTGACTGTAGTCATAAACAATTTGTTAATCCATGGTGATTGAGTCATCGAACAACTC

ATCTATAAAAACATGCACTACACAGAGACCCACCAACCAACCCAGCAACCACGACTGTTA

CTAATTAGTATCGGCTCTTTCATCTTCTTGGTTGGAATGAAAAAAATACGAGATGGTGCA

AAATTAATTTCATTCCATGATTGCCGGCTGGAAGCCGGGCACCCAATCAGGGGAAATGAT

AGGGGCAAGGATCCCGGTAGATTTGCTCGGAATCGCAATTCGATCCCACTCGCTGTGATG

TTCATCTCGCAGGACGATCGACGAATTGACCGGCCAGACACACACCGTCCTAAATCGAAT

TGGACGAGGGCGATGCTAATCTTGCAGAGTAACCAATCAACCAATCCGTCCGCCCGTCCT

AAATCGAATTCGGCGAGCTAGTGATGAGAGGAGGCGGGAGGAAGGAGAGAGAGTAACCTG

GAAACTGAAGCGCAGGAGAGGAGGCAGACGAGGAGGCGAGGCGAGGCGGGCGGGCAGGTC

TGGATTTTTTGGTGTGGTCGTGTGGAGAGCACAGGCGAGATGAGGATGGCCGAGGAGGGG

GGCGGTGTGGGAGGCTACTCCGAGAGGGAGGGATTGGTTGGGTAGTTGTTGGGTGCGGTG

GTTGGCACGCACACCCATCAGCACATGCGTAGACAGGGAAACGGGGGTGGGTTCGGAAGG

TTTGACCTGTGACCCGGCCAAACGACAGCGAATTGCAATTCACGCACGCGGCGCTCGCAC

TGGCTGCGTCTGCGTTGCAGCCGCCCCACTGGTTCTTCCTCCTTGCTTCGTGTTCC

>Traes_1BL_28A373A1F

CACCCCCGTTTCCCTGTCTACGCATGTGCTGATGGGTGTGCGTGCCAACCACCGCACCCA

ACAACTACCCAACCAATCCCTCCCTCTCGGAGTAGCCTCCCACACCGCCCCCCTCCTCGG

CCATCCTCATCTCGCCTGTGCTCTCCACACGACCACACCAAAAAATCCAGACCTGCCCGC

CCGCCTCGCCTCGCCTCCTCGTCTGCCTCCTCTCCTGCGCTTCAGTTTCCAGGCTAGGTT

GCTGTTGTTGTTCACATGGATTCCTTTTTCCAAAGAGCTTTCGGCGACCCAATGTGCTCG

GAAGACAGTAATGTTGTCCAACAAGGAATTGATAAATGCCCATTCCTGAGGAACATCAAC

GAGCCTACAAGTTTTTCCTTGACATCCATTAATTTCCCCGCTCCGGCAACCGGAGCCAAG

GGTCCAATTTTTGAAGACGGGCCCAACTTTGACACGGCGTTTCGAGTTTTCCATGGCAGA

GACGGGGTTGTTCCACTTTCAGAAGGAGCCTTTGCACATATTGAGAAGCCTCTGCCAAAG

CCCACTCCTGAATTTAACCCCTTGGCAGCCAAAGCAGCTACCATCAGTCTCTCGGGATTC

GGAGGCTTTTTCAGCTTCGGTGATTTCTCAAACAAGCGCAATAAGCAGAACTCCAACAAG

AAGAATCCCAACAACCTCCCCCAGAATAAAGGCCAGTCTAATAACAATCATGAAGCGATG

AGCAATGACTGGCTGGAAAATGGTCAATGCCCTCTTGCAAAGTCATATAGAGCAATTGGT

GGAGTCGTGCCTCTTGTCGCAAAGCTGCTGACACCCCCAGCTGGTATGAAACTGACGTGC

CCTCCTGCGATTGTTGCTGCCCGTGCAGCAATATCCCGCACGGCGTTTGCGAAGGGGCTT

CGCCCTCAGCCCCTACCGACAAAAGTAGTGGTGATCGCACTGCTCGGTATGGCAGCAAAT

GTTCCTCTTGGCATCTGGAGGGAGCACACTACAAAGTTTTCAGTGCAGTGGTTTGCGGCG

GTCCATGCTGCAGTGCCTTTCATAGGGATGCTGAGGAAGTCCATCCTGATGCCGAAGAGC

GCCATGGCCCTTACCATAGCTGCCTCGATATTGGGTCAGACAATTGGTTCAAGAGCTGAA

CGTATCAGATTGAAGAGGGCAAAATTAGCAGCAGAGGGCCATGGCCATGAACATGCTACC

CGGATTGAAGCTCCGGTGAACCTGAAAACTGGGAGCTCTGGTGCTGTCCAGCTCTGGGAC

CCGCTTGGCCTCAGAGTCAAAGGCGCCGTGTCCCCAGCCCTGGTTCCGACTGTCGGTGCC

ATGTATTGATGAGATGATCGTGCTTCATTGCGGCGGTTGTGAGAATTGTATCAGAGTTCC

AGAAATTGTTTCTTGTATTCATTGTTGTTGTGTCTTGTAAAGGATTACTGGCTACATTGG

ATTCTTCTCCCCACTATCATGAGTTGGGTGCAGTCAATAAATTTCACCATGTTCTTTGGT

TTCCTCTTTGCAGCAAGGTTGGCTGATGAGCCTATTTCTATGTTCAGAACACAATTCTTT

CATTTGCTTGGTCAAAAGCCAGAACATAATAGCTCTCAATCATACCAAATGCAACGGCCA

ACAAAGTCTACTGCTAAAATTAGTGCTACAACTTTGTTTTGACAAAAGCTCCAGCAGTTG

CTTGCTGTGTAAAATAGTACTAGTACATGCGACGAGGCTAGCTGGTTAGACCAGTTGTAT

GTTCCATGTGCTTTCATTTGCTTGGTCAAATGCAAAACACAACAGCTACGCCATGACACC

AAATGTAACGGCAGATAAAGTCTACTGCCAAGGATTACATCATCTTTGTTTGACGAAAGC

TACAGCGACTGCCTGCTGTTTGAAATACATGTGACTGACAGGCTAGCTTAGTATATCTCA

TCGTCTGGGACATGCGGCGTAAGGAACTTCTGAGGGTCCTTGTACATTTCCTCGTAGTTG

TACTTAGGCTTCACCCTCTCCGGAACCGGCTCAAGTGGGATAATGCTGTCTTCGTCGATC

ACACCATCGATGATACCGTAATCAACAGCCTCAAGAGGACCCATGTATCGGTCCCTATCG

ATGTCTTTCTCTACCTGCTCCAGTGTACGCCCCGTAAAGCCTGAGATCAGCCGGATGACA

TTCCTCTTGCTAGCCAGAATTTCCTTGGCTTGGACCTCCACATCCAAGGCCTGGCCACTC

GCGCCTCCCACGGGCTGATGCATCATAATCCTGGTGTTGGGCATGGCGAATCGTTTGCCC

TTTGCGCCACCACCAAGGATTATAGAAGCTGTGGATCCAGCTATGCCCATTCCAATAGTG

GATACATCTGCCCTCACCAGCTGCATTACATCGAAGATAGCCATTGTCGCACTGCAATGG

AAAACAAAAAAGGTCAAGTCACAAAATGCAACTAGTCTGTATCAACAGATATGCAAAGGC

TACTATTTCACGCACTTCACCGTAATGGCAAGGCTAGCCTAAAACTCCTCTCATTCTCCA

ATAGTGTTGTTTTAGACCTCAAATATTTCAAATAAATCTACACGTAATATTTCAAGTACC

CATTCTACTAGTAGTCAAAGTTTCACTTTGCTTATGTCCAAAACTTGTTCAGGTTCCATG

AACTAGTCCTTTGGACAGGACTATTCTTGTCCCACCACTCATACCAAACTAGAAGAGGAC

ATTCCAAGTCAACTAACAATAGGGTCTTCCAAGTCAAATAACAGGGCATTCAAAGCCAAC

TAATAGTCAACTAGTCAAATGTGATATCTTGAACGAGAGAGATGAATGTTACGCACATAT

AAACAAGAAAAGCATAATTCATAAGGTGCAACAAATATCATTACAAAGTCCAGACATCAA

GCAGAATATTTTATTTTCACAGAAACGAGGGGTCTGTACAAAATGCATATATAGAAGAAC

TGTAATCATATACCCATATTGGTGTAGTACTGAAGAGTTTCATCTAGCTAATCTTATAAC

AAACCAATAAGAAACGAGCTACTGATAGAAGCTTACATACTGTTTGATCATCACAGAGAA

CAAGGTGTTCAATGCAGGTATTACCAAAAAGCTACATTCAGCTGATCTCATAACAAGCCA

ACAAGTAGCGAGCTAACGATACAAGGTTAAATATTATTTGATCGTCACAGACAACAAGGT

GCTCGATGTTGATATTGCTAAGAGCTTAATTCGGCTGATATAACAAGCCAATAAGCAGCA

ACCTAGTGATACAAAATTACGTACTGTTTGATCATCATAGAGAACAAGTTGCCCGATGCG

GATTTGAATTAGTCACTCCATTCAATTCTAGATTTTAGTTATTGCCTAGATTCAGTGGTT

AACTTAGGTGTTACCATAGGTAGATTACACTAGGATTTTTTTGTCAACCTTTTTTCCGTT

TCAGCAATTATGAGTATAGAAACGACCCACTGAATTAGACAATAATACAATCAAACTGGA

ATTATTTTAGGGATAGCAAAAGTCTAGTTTAATGAAGATAAGAACAAAGATAATATTTGC

AATCCAGAACTGTTGATGCGGTGTAGAATAAGATTTCCGCCTCTGCTCGGACCAGAAAAT

CGTAAAACAAAATTGAATGAATAATAACCTTCTGCGGGGATGGCAAATTACTAAAATACA

TAAAACGTCTAGCAAGAGGGTGACTCAAGGATAGTCAGTAAAGTATCATTCGGACATTAG

TGCAGATTTTAGCAAATGGGTTTATAGCTGGTACAGTCTGAAGGTGTGTCAGTGTCACAT

TGATCATGGCCAGCAAACTCTTATCCAGCAAGATATTGTCTGATAAAAAGATGGCCTACT

GCTGTGAATCTACTTGATGAGTCTACTACATATGCAGGTCACGATTTTGATTAGTGCAGC

CCATATCAAATCAAACAGCGGGGAGTGAATACTATCACGAATCAAACTAGTATTTGGACA

AAGTAATTTGCCCACAAGATGACCATGTGGGATAATGTAACACCACAAAAGGCATCCCTT

GTTGAGATGGTAAATTCAACCCTACACAAACCGAGGAAAGGGAGTGATGCATTGGGAGGA

GACCTGAGTGATCCACCGGGGGAGTTGACGAAGAGCCGGATGTCAGATTCGGAGTCCATG

GCATCGAGGAGGAGGAGCTGGCTGACGACGGCGTCGGCGAGGAAGTCCTCGATCTCGTTG

CCGAGGAAGATGATGCGCTCCCGGAGAAGAAGGCCCATGACGTCACCGCTGGCCACTCCT

CCGGGCGCATCCAACTCCGCCCGGATGCCTTGGCCTTCCCACAGGGACAGAGGAGACATG

GCCGCCGCCGACGACGCCGAGACCGCGTAGCTCCGGCGGTTCAGCTTGAGGAGGGGATGA

GGTTGCGCCGCGGGCCCACGTGCGCAAGCGTATAGCTGGCGGTGCCTGAGGCGGTGAGTG

GCAGCCGTGACGGAGAGGGAGGCAGCGGCACTCGCGCTGGTGGCCGCCATGCTTATCTAT

CTAGCTTCGTGGGTTACGGAGCAGCCGGCGGCGGCGCAGTGCTCCCTGTCTCCTCCCTCT

GAG

>Traes_5BL_31F1C283F

ATATAGCTGGCACAGACAACACCGACGTGAGTGGAACTCGGAAGGTTCTTGGATGCATTA

TAAACTTGAGAAATACAGTGTGCCCTACCCCCACATCACCACCTAATTAATTGAAACATA

GCTACCTATCACTGTCGAGCTTACACAAATAGATACCCTGCAAAGTGTAGAGACATATAA

ACAGTAATATTCTTATTTCATCTTATCTTTCTATGATAATGTCAACTGCCATGCGATTTC

ACCTAACCAAATTTGAACTTCTGTTATTTCAGGACTACAACACTAAATTGTCAGACTTTG

GGCTGGCCAAGGATGGGCCTCAAGGTGACGCAACACACGTGACAACACGTGTAATGGGGA

CACACGGGTATGCAGCACCCGAGTATATCATGACGGGACACTTAACCGCCAAAAGCGACG

TCTACAGCTTCGGTGTTGTGCTCTTGGAGCTACTCTCAGGACGACAATCCGTGGACCGTG

CACGACGACCAAGGGAACAAAACCTAGTGGACTGGGCTAGACCCTATCTCAAACGGTCGG

ACAAACTGCACCAGGTGATCGACTCAGCCCTCGAGTGTCAGTACTCGTGCAAGGGTGCTG

AAGTGGCTGCGTTGGTGGCATACAAGTGTTTGAGCCAAAACCCCAAGTCCCGACCATCCA

TGCGTGAGGTCGTCAAGGCATTGGAGCCTGTACTCGACATGGATGACTTTTTTCCAGCGG

GACCGTTCGTGCTCACCATCGTTGTTGAGGATGACAAGGTGATGGACATGAAGGTGGAGA

CTGAGGAGAAGCACCAAAGCCATCACCAGAACCATCAGGATAGGCACCGACATAAGTACC

CCGAATCAGCGATCCACGGTGACATTGTGATCCATGGAGACAACAGACAGGTTGCCGGGT

TCACCGGTGCATTGCGGCGACAACAGAGGACGTTGAGTTACCACCGGGAAAGAGGGGCTT

AAAAGTTAGAAGTATGGTAGGGGAAGTGTGCATGATTGTAGATAGAAAGTATGTATAGGT

ACATACTATTGAGATGTGATACGTATCACATCGTTTGTTTAGAAATTATGGATGGCGTTT

CTTTCTTCTCTTCAAAATAGTGGCAAACATACAAAAGTTTGGTTCAGATTTTGGCCAAGT

TTGTTGTTCAAATATGTGTGCAGAACTGCAAATGAATGAGTCTACCTGATGCCACATGCT

CATTACTTTCACTTTCAAATAGTTGGTCG

>Traes_5BL_388501B7C

TTCCGAGTTGTTCCGAGTCACTGAATCTGCACAGAACGACGAACTCTTGTTGAGCTCATC

GGACTAACCGCCATGGTTTCGTCAATGAAGCAGTACAGAGATCAGGCCGCCGGGGTGCCC

ATGTCCCTCTCCCTCTCGCTCTCCCTCGGTGCCGTGGCCGACCGCAGCAAGAAGATGCGC

CGCGGCGGCGCCGACGGCGAGTTCGTGTGCAAGACGTGCGGCCGCTCGTTCCCGTCGTTC

CAGGCGCTGGGCGGACACCGGACCAGCCACCTCCGTGGCCGCCACGGGCTCGCGCGCCGG

ATACGATCAGCCGGCGACCAGGAAGACCACGGACCAGAAGCAGGCGCACCGGTGCCACAT

CTGCGGGCTCGAGTTCGAGATGGGGCAGGCGCTTGGCGGCCACATGCGTCGGCACCGTGA

CGAGGCGGTCGCCACCACGGCCCAGGCACCGCCCGTTCTGCTCCAGCTCTTCGTCTAGCT

CATTTGTTTGATTCTACTTCCACGTAGATAGACATCCAGTGCAGGTTTACTGGTTCACCT

GTGTGCGCGATTGGATTTGTACATTTGCTGCACACTAGTAAGTGTTGATCGCTGCTTCAT

TCTCTCATTTTTATGATTATGTACATATTTAGGACTTGTTCGTTCGTTGATTCAGA

>Traes_4AL_91D0D50DD

ATGGAGTACGCAAAAACCATAGCTGGCCTGTTCGCCATGCTGCTCCTTGGTCCGGCTCTG

GCCACCGACTTTGACCCTCTCCAGGACTTTTGTGTCGCTGACCTGGACGGCAAGGCGGTC

TCGGTGAACGGGCACCCATGCAAGCCCATGTCGGAGGCCGGCGACGACTTCCTCTTCTCG

TCCAAGCTGGCCAAGGCCGGCAACACGTCCACCCCGAACGGCTCGGCTGTGACGGAGCTT

GACGTGGCTGAGTGGCCTGGTACCAACATGCTGGGTGTGTCGATGAACCGTGTGGACTTC

GCGCCGGGGGGCACCAACCCGCCACACATCCACCCCCGTGCCACCGAGATCGGCATCGTG

ATGAAAGGTGAGCTCCTCGTTGGAATCCTTGGCAGCCTTGACTCGGGGAACAAGCTCTAC

TCCAGGGTGGTGCGCGCTGGGGAGACGTTCCTCATCCCGCGCGGCCTCATGCACTTCCAG

TTCAACGTCGGTAAGACCGATGCTTCCATGGTCGTCTCCTTCAACAGCCAAAACCCCGGC

ATCGTCTTTGTGCCGCTCACGCTTTTCGGCTCCAACCCGCCCATCCCAACGCCGGTGCTC

ACCAAGGCACTCCGGGTGGAGGCCAGGGTCGTGGAACTTCTCAAGTCCAAGTTCTCCGCT

GGGTTTTAA

>Traes_5AL_32A81F167

CTGACTTCTGCAAACTCTAATAGAAGACTTGTTTACAGAATGGGAAAAAAGAGGTCAACG

AAGAACTGAAAAAGGAGGCAGAGTTCTTTGGTGACATTGTTCTGGTCCCTTTCATGGATA

GCTATGACCTTGTTGTTTTAAAGACTATTGCCATTGCTGAGTATGGGGTGCGAGTTGTGC

AAGCGAAATACGTAATGAAGTGTGACGACGACACATTTGTTAGAATTGACGCGGTGTTGG

ATCAAGTGAAGAAAGTAAAGAACGGGGGGAGCATGTATGTGGGGAACATAAACTACTACC

ACAGGCCTCTGCGATCTGGAAAGTGGGCTGTAACGTATGAGGAATGGGAAGAGGAAGTAT

ACCCACCTTACGCAAACGGACCGGGCTATGTGATTTCATCAGACATCGCTGAGTACATCG

TATCCGAGTTTGACAACCAGAAACTGAGACTGTTCAAGATGGAGGACGTGAGCATGGGCA

TGTGGGTTCAGAAGTTCAGTAAGACTCGCCAGCCGGTGGAGTATTCGCACGACGTCAAGT

TCTTCCAGGCCGGCTGCTTCGACGGCTACTACACGGCGCACTACCAGTCGCCGCAGCACA

TGATCTGCCTCTGGAGGAAGCTGCAGTCCGGGAGCGCTCAATGCTGCAACGCCAGATGAC

AACGAGGCTGCCTGGTAATTGAAAAGCAGCACCCGGCTCTCTGCAGAATCAAACAGCAAG

GATGAGTTCGAGTTTGATCTTTGATTTGGAGGCTCTCTGGAGAATTAGCTTGCGTAATTC

GCTCATTCTTTTGGTCAGTGGCTGGTTAATTCAGCTACTGATTCTGACACTAGCTAGATG

CTGACACCATGTACATGTGTCAATTGTTTTTGACCTAGGAACTAACAGCTCTGCTGATCT

CACCTGTATACCGGTTGTTCTCATATATACATATTAGATGTTTCAAGTCAATCATTTTTT

GTATTCAGAGGCACATGTATGCAATGTCGATTCCAC

>Traes_2DL_0FC1C6597

GAGAATCCTTCGTCCCCTCGCAGTCGCACGCACGCACCAACTTCCCACACCACAGCATAC

CAGGCGGCGGCGGCGGCGGTGATGGGAGGCGAGGGCGCCAGTTCCAGCGGCGGGGGGTTC

CGCGCCCGGATAGACCACTACCTGTACAGCGGAGAGAAGAAGCACGTCGTCGCAGGCATC

GCCATCTTCGCCGCCATTTTCGGGGTGCCCTGGTACCTCATGACCCGAGGGGCAAAGCAT

GACTCCCATCAAGATTATGTGGAGCGAGCTAACAAGGCGAGGTCGGATAGGCTTTCTTCT

GGACAGCCATCATCACTGAAAGAATGAAGCAGCTGTCTCAACCGGCCCCAGATTGCCCAA

CATCTGCATGCAGTTTACATTATGAATCTGAAGGCTTAATCTTGTGTCAAATAAACAGAC

CATCTCTGTTCTGATTGTCAGCAGCTATGGGGAAAACGTAGTGCTATTGTAATACAGTAA

TAGAAATTTTGTTCGCGGGAAACTCAGAGTAGTGTCTTTTCTATTCTAAGAGGAGATGAT

GGACAAATCATGGTATTTTTATCTCATCCGGAGCCAACTCTTG

>Traes_4DS_58C8EF3CE

GCAATGGGGTACTCCAAAACCCTAGCGGATGGCCTGTTCGCCATGCTTTTCCTAGCTCCA

TCCGTCCTGGCTACCGACCCTGACCCTCTCCAGGACTTCTACGTCGCCGACCTCGACGGC

AAGGCGGTCTCGGTGAACGGGCACACATGCAAGCCCATGCCGGAGGCCGGCGACGACTTC

CTCTTCTCGTCCAAGCTGGCCAAGGCCGGCAACACATCGACCCCAAACGGCTCGGCTGTG

ACGGAGCTCGATGTGGCTGAGTGGCCTGGGACGAACACGCTGGGCGTGTCCATGAACCGC

GTGGACTTCGCGCCGGAGGGCACCAACCCGCCGCACATCCACCCGCGCGCCACCGAGATC

GGCATAGTGATGAAAGGTGAGCTCCTCGTGGGAATCCTCGGCAGCCTCGACTCCGGGAAC

AAGCTCTACTCCAGGGTGGTGCGCGCTGGCGAGACGTTCCTCATCCCGCGCAGCCTCATG

CACTTTCAGTTCAACGTCGGTAAGACCGAGGCTTCTATGGTCGTCTCCTTCAACAGCCAA

AACCCCGGCATCGTATTTGTGCCGCTCACGCTCTTCAGCTCCAACCCTCCTATTCCGACG

CCGGTGCTCACCAAGGCGCTCCGGGTGGAGGCCGGGGTCGTGGAACTTCTCAAGTCCAAG

TTCGCCGCTGGGTTTTAA

>Traes_1BL_6E8A958A81

CCGTGCTCACCCAGCTCTTCGCCGCCGTGAACCTCGACGCAAAGGACCTTGTCGTCTTGT

CCGCCGGGCACACCATCGGGACGTCGCACTGCTTCTCCTTCTCCGACCGGCTCTACAACT

TCACCGGCATGGAGAACCCCAGCGACATCGACCCCACGCTGGAGCCACAGTACATGATGC

GGCTAAAGAGCAAGTGTGCCAGCCTCAACGACAACACCACCCTTGTGGAGATGGACCCCG

GCAGCTTCAAGACCTTCGACACCGACTACTTCAAGCTGGTGAGCAAACGGAGGGGCCTCT

TCCACTCCGACGGCGCCCTCCTCACCGACCCCTTCACCCGTGCATACGTCCAGCGCCATG

CCACCGGCGCCTTCAAGGATGAGTTCTTCGCCGACTTCGCCGCCTCCATGATCAAGATGG

GCAACGCCAACCCGCTCACCGGAAGCAATGGTGAGATCAGGAAGAAGTGCAGCGTGGTCA

ACCATTAAACCACCGACGAAGTACAAGGCTGTTTGGATTTGTGACAATCTTTTTTCCTTG

TAAATTACTGTAAAATTGTTATAGATCCTTTCTCTCCCAAATCGTTTTCTTCCATTAATT

TATTGTTTCGCTACAACCATGTTAGTTCCTTGTACACACGACGAACACTTTGGTATTGCC

ACCTCCGACGTGTCGTCCATTGTCATCTGGAGATTATTCATGTTGCGGTTACTAGTTTTC

TCTAAGTTTGTCTTTGTTTTATTTTAGATTGTTACATAATGTTTTGTTGTGTTTTTTGTT

TTACCTCAG

>Traes_5BL_FE8F2FD481

ATGGAGGTTAAGGTGTTGGGCTCCAAGCTCGTCAAGCCCGCCTACAATGGCGGCGCCGCG

CCGGCACCATCCACCGAGTACATCCCTCTGTCCATCTTCGACAAGGTGACGTTCAACATG

CAGATGGCCATCATCTACGCCTTCGCCGCGCCGGCGCCATCCACGGCCGCCATCGAGAAT

GGCCTCGCCACGGTCCTCGCCCAGTACCGAGCCTTCGCGGGCCAGCTCGGCGAGGCGCCC

GACGGCACGCCGTCCTTCATCCTCAACGACCGTGGCGCGCGCCTGGTTGAGGCGACCGTA

GACGCCGACCTCATCGACATGGTGCCAGCGAAACCCACGCCGGAGCTGCTCAAGCTGCAC

CCCGACCTAGAGACGGAGCACGAGGAGGTTGTGCTGATGCAGCTCACGCGGTTCCGGTGC

GGCTCCCTCGCCGTGGGGTTCACGTCCAACCACGTCGTCGCCGACGGCCATGCCACCAGC

AACTTCCTCATCGCCTGGGGGCGCGCCACGAGAGGCCTCCCCATCGGCCTCCCTCCCGTG

CACCACCACAAGGACCTCTTCAAGCCACGGTCGTCGCCTCGCGTGGAGCACGACCACCGC

AACAGGGAGTACTACCTGCCGTCGCCCTCCGACGTCGTCGGTCACCACGGCGATGCCGCC

GACAACATCGTCATCCATAAGGCGCACTTCTCCAAGGACTTCATCGCCGGTCTCCGAGCC

AAGGCGTCAGAAGGGCGCGGCCGGCCGTTCAGCCGGTTCGAGACCATCCTCGCCCACCTC

TGGCGCACCATGACACGCGCGCGCGACCTGAGCCCCGAGGAGACCACCAAGATCCGGCTG

TCCGTGGACGGGCGACACCGGCTCGGCCAGCCGGCGGAGTACTTCGGCAACATGGTGCTT

TGGGCTTTCCCGCGCTCCACGGTGGGTGACCTCTTGAACCGGCCGCTGAAGCACGCGGCT

CAGGTGATCCATGACGAGGTGGCGAAGGTGGACGGCGCATACTTCCAGTCGTTCGTAGAC

TTCGCAAGCTCCGGCGCCGCCGAGAAGGAGGGGCTGGCTCGGAGCGCCGTGTGCAAGGAC

GCGCAGTGCCCGGACGTGGAGGTGGATAGCTGGTTGACGTTCCCGTTCTACGAGCTGGAC

TTCGGCACGGGGAGCCCAAGCTACTTCATGCCGGCCTACTTCCCCACGGAGGGGATGCTT

TTCCTCGTGCCGTCCAACTTCGGCGACGGCAGCGTCGATGCCTTCGTACCCCTATTCCAA

GAGAACCTCCAGGCGTTCAAAGAATGCTGCTATTCCATGGAGTAG

>Traes_4BL_A35A070A6

CAATTCCGGCCCAACCAACGAACACAGTCCAGCGACCTCGAGTATTCACGCGAAGACGAG

AGAATTCTCCAGAACTGCTTTCTCCTTCTCCGCTATATCATCCCAGCTCTCTATAAATTC

TCCCTCGTCCCGATCGACAGAAACCGAAGAAAGTCTGAGGAAAGCACCGATCAAAGAGCT

CCCAGAAATCAATTGCTCACCCTTCCGCTTCCAACACCAGCCAAATCGTCGATCCATTCC

ACCAGCAGCTCCAATCCCCACCGATACGACCGACAATGTCGCTGATCCGCCGCAGCAACG

TGTTTGACCCCTTCTCCCTCGACTTCTTCGACCCATTCGAAGGCTTCCCCTTCGGCTCCG

GCAACAGCGGCAGCGGCAGCCTCGTCCCGCGCACCTCCTCGGACACGGCGGCCTTCGCGG

GCGCGCGCATCGACTGGAAGGAGACGCCCGAGGCGCACGTGTTCAAGGCGGACGTGCCGG

GGCTGAAGAAGGAGGAGGTGAAGGTGGAGGTGGAGGACGGCAACATCCTCCAGATCAGCG

GCGAGCGGAACAAGGAGCAGGAGGAGAAGACCGACACGTGGCACCGCGTGGAGCGCAGCA

GCGGCAAGTTCCTGCGCAGGTTCAGGCTCCCGGAGAACGCCAAGGCGGAGCAGGTGAAGG

CGTCCATGGAGAACGGCGTGCTCACCGTCACCGTGCCCAAGGTGGAGGCCAAGAAGCCCG

AGGTCAAGTCCATCCATATCTCCGGCTAGAGCGATGAAGTGGAGTGCAGAGCAGTGGTGT

GGAGTCTCGCCCCGGTCGTCGTGATGAAATAAAACCAAGAAGTCCGGCCGTCTGTGCGTG

CGAATGGTCCAATGTTGCAGTCAGTGTTTTGAGTCTCTCGTGTCAGTGTTTTGAGTCCGG

TTTGTTGTTTGTTCATCAGTCTGTACTGGCTCTGTACTCCATCTGCAACCGAG

>Traes_2AL_549D005B7

GTGATTCCTCGGAACGAGAAAACAAAGGAGTTATATAATTGTTGTTACCACAATATTTTC

CCCTGTGAATGCTTATCATGCGATCAAAATGAAAGACTGCCAAGTGATTAACTTGTATTT

GATAACCTGCACAGAAAACCAAACCCCCGGGTGCTGTTGGTGTGAGCATTGCACTTGAGA

AATTCCTTCCTAATAATCCTTCATCTGACGTTGGGTTGCCAAGGATTGAAAATAGCATCA

GTGTACTTGTATGTTCAAAGGGTGGCGGCGGACTCTTAAACGAGCGCATGGAACTTGTTG

CAGAACTTTGGGAAGCTAACATAAAGGCCCAGTTTGTTCCTCAGGAAGACCCAAGTCTTC

AAGAACAATATGAGTACGCCAGTGATCATGACATCAAATGCCTTGTGTTTATCACTGAAG

CAGGTCTTTCGCAAACAGATCTTGTGAAGGTTAGACATCTCGATGCAAGAAAGGAGAAAG

ATGTTGAAAAGGAAGAAATTGTGAAGTTCCTGTCTGAAGCAATATCTTTACAGTTCAAAA

ATCCTACAATCTGGAGTTGATTGGCAATCTGCTCGTTAGTGAATTTTGTTCAGGTTTGCT

ATTCCAAAGGATCAATCGATATCAGACTTTTTATCTACCTGTCCTGGATATAATCTTTCC

TATGGTCAGGTGAAAGCAGTGTTGCCTTTCCAAACATTTTGACCGACACCAAACGCATTC

ATGATGCTACAGTACAAGATTGCAAGGTGACTGGTGGCTTTGTGCAATCTTGGTTGTCCC

TGCATGGGCCAAGATTATTCGGTGTGTCCCCAGTTCGAGAAAAGTGATTTGATTTGATGG

CCTGGTGTAATCCTGATTGTCCTAGAGCAGTATCGCAGTCGATGCAAGCTGACGGGTAGC

TGTTGTACGGCAATATTGGCTGCCACCATTCTGTGGATAGCTAGATGGGGAGAGTTGCTT

CTGCGATGTGACTATGGAGCTGCAATCCTGCACAACGTTCTTGTTGATAGCGGAACGGCT

GTGCAACAGGCTCACCTAGTTACGCCCTTTGCAATTTTTTGGCTTCAAATTCTTACAGAA

GGCAGTCTTGTGCATAGTTCAAATTGTTTACAATCATGTACAGGCCAATTTAACTTCTTT

GATGCTCATAGGGAATGCCCAGTTTGCCAGTGCAGTTTTGCTACATATCAGTCAGAGTAG

AGTGTATGTAACCTGTGCACGGTGCTAGAGAGCATCACTTGATTCAACTCAAGTGTTTCC

GTCACTGTGCCAAGTACCAACCCGGCATGAGAGGCCGGTGTCGTGACGTTGATCTTTGCA

GTTGTCACCTCCCGTTTG

>Traes_1AS_C2D84E4F8

ATGGTTGTCCCGGTAATCGACTTCTCCAAGCTCGACGGCGCCGAGAGGGCGGAGACCATG

GCGCAGATCGCCGACGGCTGCGAGAACTGGGGCTTCTTCCAGCTGGTGAACCACGGCATC

CCGCTGGAGCTCCTTGACCGCGTCAAGAAGGTGTGCTCCGAGAGCTACCGCCTCCGGGAG

GCGGCGTTCCGGTCGTCGGAGCCGGTGCAGACGCTGGAGAGGCTGGTGGAGGCGGAGCGG

CGCGGCGAGGCGGTGGCGCCGGTGGACGACATGGACTGGGAGGACATCTTCTACCTCCAC

GACGACAACCAGTGGCCCTCCGACCCGCCGGCCTTCAAGGAGACCATGCGGGAGTACCGC

GCCGAGCTCAAGAAGCTCGCGGAGCGGGTCATGGAGGCCATGGACGAGAACCTCGGCCTG

GACAAGGGCCGCATGAAGGCCGCCTTCACCGGCGACGGCCTCCACGCCCCGTTCTTCGGC

ACCAAGGTCAGCCACTACCCGCCGTGCCCGCGCCCGGACCTCATCACCGGGCTCCGCGCG

CACACCGACGCCGGCGGCGTCATCCTGCTGTTCCAGGACGACAAGGTCGGCGGCCTCGAG

GTGCTCAAGGACGGCGAGTGGCTGGACGTGCAGCCGCTCGCCGACGCCATCGTCGTCAAC

ACCGGCGACCAGGTGGAGGTGCTCAGCAACGGCCGCTACCGCAGCGCGTGGCACCGCGTC

CTGCCCATGCGCAACGGCAACCGCCGCTCCATCGCGTCCTTCTACAACCCGGCGTTCGAG

GCGGCCATCTCGCCGGCGGTGGG

>Traes_5BL_99E4AE143

ATGGCTTCTGAGGAAACCAAACCAAAGAAGCAAAGAGATGAGGAATGCATTATAAACGGT

CTCCCAGGAGAACTCATTGAGCGGATATTTTTGAAGCTTCCAGTGAGCACTTTGTTGAGG

TGCACTGGTGTTTGCGAGCAGTGGCACAAAATCATCCGAGATCCTCAGTTTGTCACCTCT

CACCTCCAGGATGCGCCCCAATGTGCCTTCCTATTCTTTCCACAAGAGTCGGTCTCAGGT

GAACCCCATCCTGCTGATGCTATCCTGATTGATGAAGCCTTGTCGCCATCGACATATGCA

GTGCCAGTGATTGGGCCTGACGATTTCCTTTGCGGTTCATGCAATGGGCTTCTTGTCTTA

TACACAAAGTCATCAACACTCAAGGTAGCTAACTTTGCAACTGGTGAATGTCTGCATCTT

GAGAAACCTGTAAAGAATTTGAGGGGTGATCACTTGTTCTACAACTTTGGATTTCACCCA

TTGACAAAAGAATACAAGATTACACACTTCCTTGGTGATCTTGTTGTGGGCAGCACTCGC

TCCCATAATAATAGCAAATTCAGCGTCATTCAAGTTTACACACTTGGTGATGAGAAATGG

AGAGATATCAAAACTCCAGAAGCCCTAAGCTTAAACTGTGTAAAAAACTCTGGAGCAGTC

AATATTGACGGAACAATGTGTTGGCTAATTGAAGACATGGTAGCTAACTGGCAGCATGCA

GTTATGACCTTTGATCTCAATGAAGAAAGTTTTGCACGGATACAACTGCCAGCAACTGTA

CATGAAGATTGTGCAGGTGGCGGTCCCCGTCGGCACTGGATCAGAGAGATAGATAGGAAG

ATATGTATAGCAACTGCTCAAGCCTGTCCTTCTCTTCCCAGAAGGCTTGTTGGTACGCTG

CAGATCTGGGAACTTGAGAACAAAACGGAGCAAAGGTGGAGCCTGAAGTACAATATTCAG

TACTCGCCAGATTACATTCCGGGTCCAAATTTGGTTCATAGGAATAAGATCATACTGCAA

CGTCGCGACAGCAACCTATATTCCTATGAGTTGCTCGGGGAGAACTTCAATACTAAATTG

TGTAAGATGGCAAAGCTGTTAGATTTCTTCCCCCACAATCCTGACAACATGCAATCCTAT

ATCTGTGTGAAGTCACTTGTACGTTTAGATGTATACAAGACTGCCATTGCGCGTAGGCCA

AAACAACGGGAAGGCTGGGAATTGAAGAAGTGGGAGGCATGGGAGCACCAGCTCTCTGAT

TATGAAAAACTGTGGACTCGCCTTCATGAAGAAGAGCACGAGGGAATTGCACTTGCAGAA

CACAGTAGCATATCATTCAATGGTCTACTGCCGCGTATCTTGGATGATGCGATTCGACAG

GATATAGGCATGAAAATCAATCAAATATGTCCAAGCCTTCCAGATCAGGTAATTCTTTAT

CTAAACGAAAGTCAGCATGGCTTTTATTTTCATA

>Traes_2DS_556B96318

ATGTATCCAGTGGCATGGGCAGTTGTTGAGAGGGAAACTAATGATACATGGAAGTGGTTC

ATTGCCCTGCTTATTAAAGATCTTGAGATTAATGACAATGGAGCTGGCTGGGTGTTCATT

TCAGATCAGCAGAAGGGACTGATAAATGCAATGAAGGATTATTTGCCAAATGCAGAGCAC

AGAATGTGTGCTAGGCATATCTAT

>Traes_5BL_B94B6FD53

CCACATTTGCCCTTTGGCACCCGTTGAAATTCCAGCGTTTAGTTATCTGACGATGGGGAT

ATTCATTTTGATTTGTGCCAGGTCCAATGGCTTTTGCAGAGAAGATGGTTCTTGCCGTCT

TTGGGGGCCTGATCGTCTTGTGGATGACGAGGAGCCTGACGGACGACATCCCTGGGTGGT

CTGTCCTCTTCCACGGCAATGTCGGGGATGGAACAGTCACTATCATGATGGCGACGCTGC

TCTTCATAATCCCGAGCGGCAAGAGCGACGGCGAGAAGCTCATGGACTGGGGCAAGTGCC

GGCGGCTGCAGTGGCACATCGTCCTCCTCCTCGGCGCGGGGTTCGCCATCGCCGACGGGT

TCAAGGCGAGCGGCCTGACGGACATCCTCGCCGGGTGGCTTGGCTTCCTGCGGGGCGCGC

CGGCGCTGGCGGTCGCGCCCGTGGCGTGCGCCTTCAGCGGCCTCCTCACGGAGTTCACCT

CCGACGACGCCACCACCACGCTGGTGCTGCCACTGCTGGCGGAGCTGGGCAGGACCATCG

GCGTGCACCCGCTGCTGCTCATGGTGCCCGGCGCCGTCGGCGCGCAGCTCTCCTACCTGC

TGCCCACCGGGTCGCCCGGCAACTCCGTCGGGTTCAGCACCGGCTACGTCACCATCAAGG

ACATGGTGGTCACCGGCATGCCCATCAAAATCGTCGGCGTTGCCGCTCTCACGGTCCTGC

TGCCAACGATAGGTGTTGCGGTTTTTGGCATGGATCAGAAGGTGTAGGAATCTCGTACTG

GAAGTTCAGTTGGATTTTTTGAACATTTTCAATCTGTAGACTGTAGGTTAGTGGTAGTAT

TAGTAGGAGGATATTGAGGTTGTTGTACAGTGTATACTCCGTGTCATTGCCTCATGAACG

ATATGGGGTCCTATTCTTTTCTGTATGTAAAATTTGTACCGAACGGTTCCCATGTGAATA

TACCGGAATTTGCAACTTCTTCATCCAGTTTCATCTTTTATCTTCTGTAGGTTAGTAGTA

TCTGTTGGGGTCTAGATCACCGGCAGATTGTACAATTGCGTTATCCAGACAGCTTGATGA

TGATGAGCAGATACACAATCCAGGATTGTATGATTG

>Traes_1DS_0DF78825D

GAAAACGTCTGCTTTGTGTCCGCCGCCGACCCAAATACAACACAAATTTGAGACGGAAAT

GCGCGAACACAAAGCGGACGCAAAATGAAAATGGATCTCTGCGTTGGGCAGTCTATTTTG

TCCGCGCCGACCTAAACGGACGCAGCCGGACGAAACATCATCGTGCCGCACCTGACACCT

CCTGAGCTGGAGAGTGAGTGGGCGACCACAGTATGCAGTAGTACTACTACACGTTTTCAG

GGGAGTGTAATGCGACGACGACGACGACCACGACGACGAGGGTGAGTCGAATTTCTCGTC

GTCGATGACATGACATAGGTAGCTAGGACCTGGACGACGAGGTGGGTGTGGTGGAGCGAG

GGGTGGGTGGCACCGGCACGTAGCGAGTTGGCTGGGAATGCCTAGCGAGCAAGGGAGGAG

CGACGTCACGTAGCTAGCAGCAGCAACTCCTCACCCACTCCTGAGTCCTGACTCCTCTTT

TTTTATATAAAAAACACCAAAAAAATCACATGCTGCGCTAGAGGTAGCATCACATGGCTC

GATCGACCGAACCACCGGCGGCTACCTCTCTGCCCATCTTTTAGCCACCTTTTTGGTCGT

TCCCTGCACATGGCAATTTCGTACGTAGTAATTTTCTACTCCTACGCGCTTTGATCCCGT

GGTGCTCAGAGGGACCGACATCTCTCCCTTTGAGTGGATGCTAGGGCCCACTCCCGCACA

AGGAAGGGAAATCGAGTTGAGTTGCACCTTCCTCCGTTCCGAGTTTTCGAGAATCAATGT

CCCGGCCGGCCGGCCCCCATATGATATGATCATATCGTACCCGTCGGAGCGAGCGATCCC

CACAAGGCCGCACAACAAGAGATCCGCACCGACGCAACAATTTCCTAGCAGTACTGTGAT

ACGTTTGTCGTTGTGGTTGTTGGTTGCACGAACAAGCGCTCGAGTCTTCCAGTCAGGCCT

CCCGCATGATGTCGCGCCATCCTCGCTCACCCACCAGACGGATGAGAGGCCAAAAGGTTT

CGGATCCATCCAGAAGTGTGGGTGCATGGAGGAGGCATGGTGTCGTTTTGACCGTGTGCA

CTGCCATGACGTTGTGCTGCGGAACTCGATGATCGGAGGGCTTGCGATGAATGACCATGG

TGACCGTGCACTCGAGTTGTTCCAAAGGATGCTGGAAAACGGTTTTGTGCCAAACCAGTC

AACGTTCGTCGCTGCTTTGTGCGCGTGCACCCACACAAGGTCGTCTGGACCAAGGGAGGA

GAAAATTCCAGTCGATGCAGCAGCGCGGGGTCGAGCTCGAGCCACACAGAGAGCACTATG

GGTGCCTAGCCGATCTTCTTGGGCGCGCAGGACGCGTTGAAGAGGCTGAGACTGTGTTGC

TGGATATGCCAATGGTGCGCTGATGTCGTCACGCCGGATGCATAATGATAGTACTGTTGG

CGAACGTGTCAGGGAAGCGGCTCGTTGAGCTAGAGCCACAACATGATGGGTGTTCTGCTG

CTCTGTTGAACTTGTATGCAGTCAATGGTCGATGGGAAGATGCAAGAGCCATTCGGCAGA

TGATGGCGGAGAAGGGAGCCAAGGAAGAGGTGGGTTTGAGCTTCATGGAGTGAAATGGTT

GGTTCTATGCATATGATGGTGGTGAAGGGAGCAAGATCCAGACGAGGGATCTGCTTCAAT

GTCGACAGAAGTAGCTTCTCTGTCAACTGGTGAGGCTATCTATGTCGGTTTCATGTCCTC

CGGTGGGAAGAACACAAGCTAGCCACGGGAACCACATCACACTTTTTTCTGCACATGTTA

GTGTTTATTCTTCTGCTCCACTTGCTACCTGCAGCGACTGCTGTTCAAGTGCAACTTCTT

CTTGCCATAAAGCAAACAAAAAAAACTCTAAAAAAAACATTGCACCTGTAAAATGCCAAA

AACACGACAGAATTAAGAAGGATTAAACATAAGAGTACCACTCACGGCAAAAATGCCTTG

CAGTTTACTACTCCTGTACTATGCAAGCCAACACTATCGCGTGGACTCGATCAAACGGCT

CTCGAATTTGAGCTCAACAGCTCACCTGCATTGTTGTATATGGATGAAGAATCTTTTTCA

TAGAGCACGAGTGGGGAGTTTTGGATAAACAACACATGTCCTGCCCCATATGTGTGTTTT

TTACTGGTACTTTTTTCCATTGGGATGCGGAACATTTGCAGAAATAAATCCGAAATTTTC

AGTTTTCGATGCTTGACTTGGCTGACAGTTTAACGGTTTGCTCTGCCATTTCCTCTTCTT

CAGGTGAGACCTTGAACACGCACCACCTGAGCCCCTACATGAAGCCGCTGGGTTCCGACT

ACAGCAACGGCGTTAACTTCGCCATCGCCGGAGCGACCGCGACGCCAGGGGACACGCCGT

TCTCACTGGACGTGCAGATCGATCAGTTCATCTTCTACAGGGACAGGTGCAACGACTCCA

TCACAAGAGATGAGCCCGCCCCGCTCAACATGCTAGATTTCGAAAGAGCTCTGTACACCA

TGGACATTGGGCAGAACGACATCACTTCCATTCTCTACCTGCCCTACGACGAGGTGCTCG

CAAAGCTTCCCCACTTTGTTGCTGAAATCAGGAAAGCCATCGAGATTCTGCATAAGAACG

GGGCGAGGAAGTTCTGGATACACGGGACAGGCGCGCTGGGATGCCTGCCGGCGAAGCTTG

CGATGCCGAGGGCCAGCGACGGCGACCTCGATGAGCACGGGTGCATCGCAAAGTTCAACA

ATGCTGCCAAGAAGTTCAACACATTGCTGAGTGAGGCCTGCGACGACCTGCGGTTGCTAC

TGAAGAAGTCTTCCATCATCTTCGTGGACATGTTCGCGATCAAGTATGATCTCGTCGCCA

ACCACACCAAACATGGGATCGAGAAGCCATTGATGACATGCTGCGGCCACGGAGGGCCCC

CTTACAACTACGACCCCAAGAGGAGCTGCATGGGCGACAGCAAGGACCTGTGCAAGCTTG

GCGACAAGTTCATCAGCTGGGACGGGGTCCACTTCACCGACGCGGCAAACAGCATCGTGG

CATCCATGGCTATCAGCGGCGAATACTCCGTCCCCAGGATGAAGCTCACCAGCTTGGTCA

AGCCTGCCAAGTCCAAGGCCTCGTAATGATGCATCAATCCAGTGAACCCAATGCTTTGGT

ATAGGAATGATGAAGGTGAAGGTACCACTAACAAGAACTAACCCCCAATGCGAGTTCATC

GGAAGTCCTTAATCACAGACTCGCAGCCGCCGCAAAAAATGGGATCTTATTTTTCTTCCT

TTCTTTTTCTTTTCCTTTATGTATAATTGAAGTGTGCGTATATCCTAGGGGTTAAGTTAA

TGCATATGCAGGTTGTCTGTGGCTCTGTGCCATATATGAACTTGTCTACAAAATTCTGAA

TTTTCATCAATAAATATTAAATAGAGATGGGTATTTAACATGCT

>Traes_1DS_67E26A4F0

CGGCTTAACAAAATGATGAAAAAAAAACAATTTCAGAAGATGCACTGTGAAAAAAAAGGA

TGAGTATCCCGCCATATATATGCGTGCGCGCGTAGACACTTTCCAGTGGAAACTTTCCAC

CGCTCGCGTCGTGAGAGAGACCGGGGCAAGGGAGCGGGAAGCCGAGAAGGAGCCGCAAGG

GACCATCGATGGCCATGGCGGCGGGCGGGCCCAACGCCGGCGCGAAGGTGGTCAGCCTGC

GCCTGCAGTACTACTGCGTGTTCGCGGCGGTGGGCGTGGCGGTCATCGTGCTCTCGCTCA

CCTTCCTGTCCCCGTCCGCCATGGGCGCAGTCCGCCAGAACCTCGGCACCGTCGTGGCCG

TGGCCACGAATTCCAGCAGCGGCGACGGAGCGGCGCGGGTGGTAGGAGGCGGGGAGACCG

TGGCGGCGGTGGCCGCGGCCAAGCCGGAGCCGGAGAAGCCGAAGAAGAAGGCGGAGCCGC

CGGTGGTTCTGTTCAACTTCGGCGACTCGAACTCGGACACGGGCGGCGTGGCGGCGGCAG

GGGGCATCCACATCATGCCGCCGGAGGGGCGCACCTACTTCCGCCGCCCCACCGGCCGCC

TCTCCGACGGCCGCGTCATCATCGACTTCATCTGTGAGTTGCTCAACCACCTCCACCCAT

CACCCCGGCCGCTCCATAATTACCACATCTCGCCATTCGTCAACAAACCGAGCCCCCAAT

TGCGAATTCCAAATTCCAGATAATTACTAGCTGCTTGTCATCAATTTGAAATTATAAACT

AGAGTACTCCTGGCATCGCTGCAAAGTAACATGGCTAAATTGCACATGATCGTGTCAAGT

GAATTTCCTCCGACCCTCGCCTCGCCGGAATCCCGTGAAATTACCGCCGATCCACGGTGC

ACCGAGCAACCAAAACTCTGTAAGTAGCCGGCCCGGTCGGTAGGTGGATGGACGGATCGG

GTGGGTCTCGAGTAATCAGGGTCAACAAGCTGGTCATGTCGCCTTCAACGTGGAAGAGAT

ACTAAAAAATATCGGGTGCTGTCAAAACGGTAGTTGAAGCAGTAGTCGCGAGCATGTGCC

ACTGCTTACCTAACAATGGCTGATTCCCTCTCCTTTTTGTCCTTCTCATTGCCAATCGAG

AGAGAGAGAAAGAAAGAAACCTATCGTTTTGTAATTTTGACCTGCTAGTCGTGGTTGGGT

GTGGCTGGCCTGTAGATGCCAACTGCTGGGTTAGACTATCCATGAGACTACTCACAATGG

TGAGTAACATAGGTAGTAACATCACACATATCTAGATAAAATAGATGATGTGGCAAGTAA

TAAA

>Traes_5BL_83B00CBFC

CTTTTTTTGCTGGAACCTTATTCCATTTTTGCTGCAAATTGAGAGAAGGGTGGTGCCGTA

GCCGCCATGATTTTTTGCTACATCAGATTTTGCTGGAACTAGCATTCATTTTTGCTACCA

CCGTCTTTGAAATTTGCTGGAACCAGCCACTTTGTTTGCTNCTTTTGTTATCACCATCTT

TGGATTTTTGCTGGAACCAACCATTTTTTTTTTGCTACAATCGGCTTTCCGTTTTGCTGG

AACTAGCACATTTTTTTGCTACAAACCATGTGGGGCTTGTGCTGGAACCAACGTATTTTT

GCTACCACCATCCTTTTGTTTTGTTGGAACCAGCCTAATTTTTTGCTACCACTGGCTTTG

GCTGTGAGCGACATCGCGTCGCCATTAATGCTGCAACCGAGATCGGCAGCACTGGCGAGG

AATGATTTGCGGCCAACTCCATGGTGGGGCCGTCATGGATGCAGGGACGGGGAACGAAAA

TCTGCTACAGGGCTTTTTTTGTGATTGACGACGGCGAGNNNNNNNNNNNNGCGGCGAGCT

TCGACGGCGAGCATCGGAGGCGAGGCAAACCGACGAGGGGCGGCAACACGTGATGCTGGG

TGCCAGAGGAGACAGGGGCGGCCGCCATGTCTTTCTTAGCGAGGGATGCGAAGGCTGAGA

GAAAGGGTGGGCAACGAACAGATCGGACGGCTTACACGCACGGGATCGAAGGGCTGCGCT

GCGACCGGCCCAAATTTGGGCCGGCGCGCCGGCGCCTATCAGTGCCCAATCCGATAAGCT

AAAACTCGTGGAGGCAATCATACTCATAATTTATTTGGGGCCGGGACGGAGACCACGTCG

ACGACGACGGAGTGGGCGATGTCGCTGCTGCTGAACCACCCGGCGGCGCTGAGGAAGGCG

CAGGACGAGATCGACGCGGCCGTGGGGACCTCCCGGCTGGTGACCGCCGACGACGTGCCC

CGGCTGGCCTACCTGCAGTGCATCGTGAGCGAGACGCTGCGGCTGTACCCGGCGGCACCG

ATGCTGCTGCCGCACCAGTCCTCGGCTGACTGCAAGGTCGGCGGCTACAACGTGCCGAGC

GGCACGATGCTGATGGTGAACGCGTACGCCATCCACCGGGACCCGGCGGCGTGGGAGCGG

CCGCTGGAGTTCGTCCCGGAGCGGTTCGAGGACGGGAAGGCCGAGGGGCGGTTCATGATC

CCGTTCGGGATGGGCCGGCGGCGGTGCCCCGGGGAGACGCTGGCGCTGCGGACCATCGGC

ATGGTGCTGGCCACGCTGGTGCAGTGCTTCGACTGGGAGCGCGTGGACGGCGCGGAGGTG

GACATGACGGAGGGCGGCGGGCTCACCATCCCCAAGGCCGTGCCGCTCGAGGCCGTGTGC

AGGCCGCGCCCGGCCATGCGCGACGTGCTTCAGAGCCTCTGA

>Traes_2BL_13D5272D7

CCTCGCCGTCCAGGTCTACACCAACCCATTGCCTCACACCGTACCTACTACTCGGCTGCG

CCTGCGCTGCCTATTTATTCCCTCCCCTCCCTCCATTCCCCTCCAAGAAGAGCCTCAGCT

TCACCTGCAGCTACAGCTCCTCTTCAGCACACAGATCAATCCAGATACACATACATCTGC

CGACGTTTAGCTAGCTTACTTTCAGAATCCAGATACACACATACACTTTTAGTACCTCGC

TGCATATTCCAATGGAATGCGAGAATGCACACATTGCAGCCAACGGAGATGGCTTGTGCG

TGGCGCAGCCGGCGCGGGCCGACCCACTGAACTGGGGGAAGGCGGCGGAGGAGCTCTCGG

GGAGCCATTTGGATGCGGTGAAGCGGATGGTAAAAGAGTACCGTAAGCCGGTGGTGACCA

TGGAGGGCGCCAGCCTGACCATCGCCATGGTCGCCGCGGTGGCTGCCGGCAGCGACACCA

GGGTGGAGCTCGACGAGTCCGCCCGCAGCCGCGTCAAGGAGAGCAGCGACTGGGTCATGA

ACAGCATGATGAACGGCACCGACAGCTACGGCGTCACCACCGGCTTCGGCGCCACCTCTC

ACCGGAGGACCAAGGAGGGCGGCGCTCTGCAGAGGGAGCTCATTAGATTCCTTAACGCGG

GAGCCTTCGGCACCGGCACCGACGGCCACGTTCTACCTGCCGCGGCGACGAGGGCCGCGA

TGCTCGTCCGAGTCAATACCTTGCTCCAGGGATATTCAGGCATCCGCTTCGAGATCCTGG

AGACGATCGCCACACTTCTCAACGCCAACGTGACACCATGCCTACCGCTTCGAGGCACGA

TCACCGCGTCGGGTGACCTCGTCCCGCTTTCGTACATCGCCGGCCTGGTCACCGGCCGCC

CAAACTCCATGGCGACGGCTCCAGATGGTACGAAGGTTAATGCTGCGAAGGCATTTAAGA

TCGCTGGCATCCAGCACGGCTTCTTCGAGCTGCAGCCCAAGGAAGGCCTTGCCATGGTGA

ATGGTACGGCAGTGGGCTCAGGGCTTGCATCCATGGTGCTTTTCGAGGCCAACGTCCTTA

GCCTCCTTGCTGAGGTCTTGTCGGCCGTCTTCTGTGAGGTCATGAATGGCAAGCCGGAGT

ACACCGACCACTTGACCCACAAGTTGAAGCACCACCCCGGGCAAATTGAGGTTGCCGCCA

TCATGGAGCACATCCTTGAAGGCAGCTCCTACATGATGCTCGCAAAGAAGCTCGGGGAGC

TTGACCCACTGATGAAGCCAAAGCAAGATAGGTATGCACTCCGCACGTCGCCGCAGTGGC

TTGGCCCTCAGATTGAAGTCATCCGTGCTGCCACCAAGTCAATCGAGCGGGAGATCAATT

CCGTCAACGACAACCCACTCATCGATGTCTCGCGCGGCAAAGCTATCCATGGTGGCAACT

TCCAGGGCACGCCCATCGGTGTGTCCATGGACAACACCAGGCTTGCCATTGCTGCGATCG

GCAAGCTCATGTTTGCCCAGTTCTCGGAGCTGGTGAACGACTTCTACAACAACGGTCTGC

CTTCCAACCTCTCCGGCGGGCGCAACCCAAGCTTGGACTATGGCTTCAAGGGTGCCGAGA

TTGCCATGGCCTCGTACTGCTCTGAGCTCCAATTCTTGGGCAACCCTGTGACCAACCATG

TCCAGAGCGCGGAGCAACACAACCAAGATGTCAACTCTCTTGGTCTCATCTCCTCAAGGA

AGACCGCAGAGGCCATTGACATATTGAAGCTCATGTCCTCGACATTCTTGGTCGCGTTGT

GCCAGGCTATCGACCTTCGCCACCTCGAGGAGAATGTCAAGAATGCTGTCAAGAGCTGCG

TGAAGACGGTGGCTAGGAAGACACTGAGCACTGATAACAATGGTCATCTCCACAAGGCAC

GCTTCTGCGAGAAGGACCTTCTGCTCACAATCGACCGTGAAGCCGTGTTCGCGTACGCAG

ATGACCCCTGCAGCGCCAACTACCCCCTCATGCAGAAGATGCGTGCAGTTCTTGTGGAGC

ACGCCTTGGCCAATGGTGAGGCCGAGCGCGACGTGGAGACATCGGTGTTTGCCAAGCTTG

CCATGTTCGAGCAGGAGCTCCGTGCAGTGTTGCCAAAGGAGGTCGAGGCCGCCCGCAGTG

CCGTGGAGAATGGCACTGCAGCACAACAAAACCGTATCGCCGATTGTCGGTCGTACCCGC

TCTACCGGTTCGTGCGCAAGGAGCTTGGAACAGAGTATTTGACCGGGGAGAAGACGCGGT

CTCCTGGAGAAGAGGTAGACAAGGTGTTCGTTGCCATGAACCAGGGCAAGCACATCGACG

CGCTGCTGGAGTGTCTCAAGGAGTGGAACGGCGAGCCCCTACCTATCTGTTGAACAGAGG

ATCCAGAAATGGAAGAGCACCTGCTTCAGATTTCAGAAGGCTCCAGTGATTATACTGTTT

TTTCATTGTAATTTCTAAAAGTTGATGTGTGCAATGTTCTTTCAGAGCTTCCAATGCATT

GCCAAAGATTGCAATTGCATGATTTGGTAGTGTTGGGCAGATAGTGAAACTTCTTTGATG

TAAGTTATAAAAGGGTACAGTGTATGATAAGTTTCCATGATAAATATACTGGTTCAAGAA

AGTAAAACTCG

>Traes_6DS_4AD701BDE

GTTACGGATGTCTTGCTGGCTTCTAGGGGTCTTCAGGGGCACATCACTTCTTCCCTTGGG

AACCTCCCTGGATTGCTACGCCTCAACCTGTCAAACAACCTGCTATCTGGTGGTCTACCG

CAAGAACTGGTATCCTCCAGCAGCATCCTCAACCTTGACATCAGCTTTAACCGCCTCGAA

GGAGACCTGCACGAGCTGTCATCTATGCCTGCCCGACCACTGAAGGTGCTGAACATCTCG

AGCAATTTGTTTACAGGACAATTTCCACCCTCCACATGGGAGGCCATGACAGACCTGGTC

ACACTCAACGCCAGCAACAACAGCTTTACTGGGCAACTGCCAACTCATTTTTGCACCAGC

TTTCCATCCTTGGCTGTGCTTGAGCTCTCCACCTGAACTAGGTAGCTGCACCATGCTCAG

AATGCTCAAGGTTGGCCACAACAACCTCATCGGGACTCTCCCAGATGAACTCTTCGATGC

TACCTCGTTAGAGTTCCTCTCTTTCCCCAGGAGTGGTTTACAAGGAACACTTGAAGACAT

GAATATTGCCAAACTCACCAATTTGGCTACCCTTGATCTAGGAGAGAACAACTTCAATGG

CAAGATTCCGGAGTCTATAGGTCATCTCAAGAGATTGGAGGGGCTCCATTTGAACAATAA

TAACATGTATGGGCAGCTGCCATCAGCTTTGGCCAACTGCACAAATCTCATAATCATCGA

CCTCAGCTACAACAATTTCAACGGGCAACTCACCAAGGTCAATTTTTCCAACCTACGCAA

TCTCATGTCTTTAGATCTTATACAGAACAACTTCAATGGCACAATTCCAGAAAGTATCTA

CACTTGCATAAATCTAGTTGCACTGCGACTAGCTTACAATAATTTCCACGGCCAGCTAGC

CAAAGGGCTCGGCAATCTGAAGTCCCTGTCGTTCCTATCACTTTACTATAACAATCTTAC

AAACAGAACACATGCACTTCAGATTCTTGGGAGCTCCAAAAACCTCACCACCCTTCTTAT

TGGGCGCAACTTCCAACATGAGACCATGCCAGTCGATGACATCATTGGCAGTTTTGAAAA

TCTTAGGTTTCTTGCTATAGGTAATTGCCCATTATCTGGAAAAATACCTTTCTGGATATC

AAAGCTAGCAAATTTAGAGATACTAATATTGTCTAACAATCAACTCACCGGAACAATACC

AGCCTGGATAAAAACTCTAAGCCATGTTTTCTATCTTGACATATCAAACAACAGCCTCAT

AGGGGAAATTCCAACAGCATTGATGGATATGCCAATGCTAAAGTCAGTAAACTCAAGGGT

ATTCATACTGCCTGTTTATCCAGCTCAAACACACCAATATCGCCAAGCCTTCGCTTTCCC

AATATTGTTGGATCTAAGCAACAATAAGTTCACTGGTGAGATTCCCTTGGAGATTGGTCA

GTTAAAATCCCTCCATTCACTTAATTTGAGCTTCAATGACTTAACAGGACAGATTCCGGG

ACCAATATTCAATCTCACAGACCTGCAGGTTCTAGACTTGTCGAACAACAATCTCACGGG

TGCAATCCCAGCTACATTGAACAGCCTGCACTTCCTTGCAGTGTTTAATGTTTCTAACAA

TGATCTAGAAGGTCCTATTCCATTTGGAGGACAGTTTAACACATTTGGGTCTTCAAGCTT

TGATGGGAATGCAAAACTCTGCGGGTCTATGCTCATTCACGAATGCGGTTCAGAAGAAGC

ACCTCCAGCCACTGTTATATCAACAAAACAAACTGATTACAAGGTAGCATTTGTGATTGC

CTTCAGTGCGTTCTTTGGTGTAGGGGTGTTGTATGATCAGATAGTCCTGTCGAGGTATAT

TGGCTAGTACTACTCCTAGTTGGGTATCTGTTTATGTCAACTTCGTCCAGTAAATAAGCT

TCTCCTCTAGGTACGATTCATAAACTCATAATTAGGTCTACATAATTTTCCAGGATTAAG

GGTTATTGTATTTCTTGGATATGTACAAATATGTAATATGTGAGACAATTTAAACTGGAG

>Traes_1BL_BBC0C1E7F

CTTACTGCTTGTGCACTGTTATCTTCGTCAAGTCATGGAGTGCTTGCTTCATTCTCAGGG

TCAAATAAACTCATAGGGTCCTTGCTTCTGGAGTTGTACTCATCATACTCGAGTGACTCT

TTATTGCATCTTGGGGCTGCCTGGGTATATATTGGGATGTTGCGTTTCCAGTTATTGTTG

AGCTCATACGATCCAGATCCTGCGTTCCTATCTGCTTTTATGCATTCTCAGATTCTGGAG

AAGATTTCACTCCTAGATCTCAAGGGAAAGGTCCGTCATGAATGTGAAGAATTGGCGGGC

TCTAACTCAGCAGGAGATTGTCATGATCAAAAGTTAATGCAAGAGCTGAAAACTGAAGAG

AAGAATCTCCGGTCAAAGGTTGTATTTCGACCTCGACAGTCTAAACATAAGAGTTTGATT

GCAGCATGCTGTGAGTTTGAGGGACGGTTGTCTGACTGCAAGGATCTGTTAAGTCATTTA

AACTGCAAAGGTGCTGGACAATTGGAGGTTAACAGAATCTGCAATTGGCAGATAACATCA

GGGAACTTTATTAAGAGACTGACAGAAGAGTATGGGGAATATGTGGATTTGATTCAGCCA

GTGCAAGTTGCCGTATACGAAATGAAACTTGGCTTGGCAATTGCTCTATCTGGTTCTCTT

GAGAGAGAATATTTGAAGAAGGTGAAAGAAGATGATATCGAAAAAGTTCTGGGTGCAATT

TTTACTTTTATGCAGTTTCCCAATGGACATGTTGCTGGAATGACATTAGTGGGCGTTCCA

GATTTAACAAATTATTCAATTGGTGATCAATTGGAAACTCAATACAGTGAGTTCAAGGAT

GTAGATATATTGGAGAAGCTGTCTCGTGTGTCAAGTCAGTTAAATGTTGGCGAAGTTGCA

GATGAAGTGAGATCCCATTCGCAGATGCTAGTAACATTTCATCATGTTTCTTTAGTACGG

ACAACGTACCGTATCTGTCAGTCCCTTGTCATGGATAAAACTTCTTATCTGTCTTTGAAG

AAGATCTTTGACTATTTTAAAAGCATGTGGATCGATATGAAATCTAGTGTGAAGGCTAGG

GAAAATGACGATTCTCAGTATTACAAGTTCAGGTCCCGTATCATCGATATAGATGACATC

TTTAAAGGAGATGTGCCTTCACTTTCAGATATAGACTCTGATGTCAATGCTGGTCCAGAT

AATGAAGAAAAATTGGAGCTTGAGTTTTTCAAAATTATGGAGAGAAGTGACGAAGATGAT

GGTTCTGTTGAAGACAAATGGGATCTTGTTCCAGAGTCTGCTCTGAAATGTATTATATTA

ACACATAACCAATTGTTTGGATCTCATGATCTTATCGGAAAGACTGAAAAATTCCAAATT

AGTGATCAGCAGAAACTTCAGTCTTTTGTAGACTCGTACGATTTTGGTGCAAGAATACTG

AAAGGCCTGCCAGAATTGACTTCTTCAACACTAGATGAGAAACTCATGCCTGAGCATTTA

CTCCGTGTTTGTCTGGAATATCAGCGAACCTGTGCAGCCTCTCTAGGCAGCAATAGTTAT

AATGCTTACAAGGACCCAAATCCTCCTGTTTTATTCAAAATGGTTGAGCCACTAACTGCA

CTTCAAGAGAAAGTTAGGACCTTTTTAGACGAGTGGCCAGATCATCCAGGTCTACTGAAA

ATATTGGAGATAATCGCTTCCCTTTTGGCAATGCCCCTGAGTGCTCCACTTTCTAAGGTC

TTACTTGGATTGCAACTGTTGGCTGGGAAAGCTCAAACATTGCAGGAAAATGACTCCAAG

TTCTTTCTTAAAGATCATCTTCCACCAATATTCATGCTTCTGTCTTCATGGCAAAGGCTT

GAGTTAGAATGTTGGCCTATCCTGCTTGAGGAAGTCCAGGGAAAGTATGAAACTAATGCT

GCAAAACTATGGTTTCCACTGCGAGCATTGCTTTCTCAGTCTTGTGACATTCCAACGAAT

GATGATTTGTCTATAATCAAGAGCATAGAGGAGTTCGTCCAAACATCAAATCTTGGAGAA

TTCAAGACACGATTGCATCTCCTCCTTGCATTCCATGGTGAATTTTCTGATGGTGCTAGT

GTGGGTGTTTATTCAAGTACTCCAGTGAAGAAAATCCAGAACATTCTGTACAACGTGTTT

GGTTACTACATGCAGTTCTTGTCACTTGTTCTTCGGCAAATTGAGGTCGGTAAAGAATCA

GTTGAAAAGGAGTTGAAAGATCAGGTTAAGCTTTATCGATGGGAGCAAGATCCATACAGT

CTTGCTTCGATTGAAAATTTCAAGAGGACGAGGCAGAAATTTTTTAAGCTTCTGCAACGG

TTCAATGATATTTTGAAGAAACCTGTTATTGCCCTGCTGAATGAAGAGGCTACAGCAAGA

AAAGTTCCATGTTGGCTTGATCCTGAGATACCTGAATCACAGTTTCCTGTTGACACGGAG

AAACTTGGTGAAAGGTTTTTGTGGTACAACAAGTGGAAAAACCAGGCTTCTCTGTCACTG

CGAACATTATTGCATGCAAACGATAGTGCTGCTGCAGTTCCCAACGTGAAAGAATCTGTA

TATGCTGTTGTGCACAATATGAACCACCAACAAGATGAAACTGAACTCAACGATAGATTG

AAATTCTTTTGGTATGCACTTGAGAGGATTTGTAATGCGGCTGATTTTGGTTCTATACTT

AAGCATGGTAAAAAGAACCAGAAGAAAACTGCTTTGTCCAGTTTGTTCAAGACACTCGAG

GAATGTGGACTCTCAAAGCACAGGCCAACGAGTCATGAGTGGGGAAACGAGTTGGATGCA

CCAAGCCCGTTGTTTTTGGAGCAGTCATACAACACAACACATTTACTCCAACAAGTGACC

ACGCAAAAAGCGTGTGAAGATGTTAGCTCTATTCATTCTACCCTATTGGGCGCAAATAAC

TGGAAGCTTGCAAATCAGCAGTACTTCAGATGTTTGGCAACGGTGCAACAACTTAAGCAA

ATTTCCTTGAAATTTAACAAAGATCTAGACTTAGTAGAGGTTAATAAGGCTGCGTCCTTT

ATGAACCATCTTTTGACTACGTTGTGTGAACAAAGACATCTTGCTTATGATCTATTTGAG

CAGCTGAACCAGTTTCGGCATATGATCTTATTATTAGGTTCAGGAGGGAAGAGCGGATCT

CTATCATCCTGCCAAAATGTACTGCTAATTTCCATGTGGCAGCAAAAGCAATTTTTTGAC

AATATGCTAGCTATGGCCACGGACACCAACTTGTTGCTCAAAACCTTCAAAGGCTCTCAT

CATGCCTCTTGTGACAACTTTGAAGTAGAAGTTGCCGCGATGTCAACTCTTTTGGAAAAG

TTTATTACAAGATTTTCTGAATCCAAGGATTTATTAGACAAATTCTTAGTTGGATCCAAC

AATATCCTTGCTGGTGCCCACAAAAATATGCCTCTTGCTACAATGGAGATGGAACAACTT

GTTGCTGTGAATTGCCAGCTTGTCAACACATTTAGAGAGGATATACAGGTTTTATCCCAC

CAGGATGTTGCACTGAGATCTGTGAAGAAAGTTCTGCTCTCTCGTTTCAAAGAGTTACTG

GACAAGGGGAAGATAGCAACAGAGAGTTTCTCTAGAGAACTTAAAGACAAGCATGGACTA

TTCTCTGATGAACAAAAGCCTGAAGATGCATATGCAGAAGCTTTTAAGGAAACCTTCGCA

CTAGCTGTCGGTGTTGTAGGACATCTCACTGGTCTTGGGAGATCCATTGATGAAACTAAA

GAACCCTCGTTAGAGGGGAATATTACCTCCTGGAAAGATATTTTGCATTCGTATGTCATG

AATCTTCAAATGGACCATGTCTGTGACGCTGGTGAAAATCTTTCTGTTTTAGTGAGGAAA

CTGGTAGACTACAAACCTGAGATGTGTTCTATAATAGAGGCACAGTTAATGCATCTTCGT

GTTTTGCTGGGTGTGATCCTGTCATCAGCCGAAGGCATACTTTCTGAACTTTTGGAAGCT

CACAGAAGAACATCTGAAATGACTCATGCACTCGGGGATCTTTTTATTTATCTATGTGCG

GAGGGGTTTGGTTGCGTCGAAGATACAGCTGAAGATGCTTCAGACGGACAGAAGGATGCA

ACAGGAACTGGAACTGGCATGGGTGAGGGGGAAGGTCAAGAGAGTGCAAGCTCTAAAATA

GATGATATATCACAACTTGAGGGCACCAATGAAATGCAGGATGCTCAGTGTAAGGGAGAT

CAAACTCCTAAGGATGATGATCAAGCAATTGAAATGGAAGGAGATTTTGCTGCTGAATTA

GCTGATGTTTCTGAGAATGAAAGCAATGACTCAGGCAGCGAGGATGAAGACAACTTGGAT

AGTCAGATGGGTGACACTGGTGATGCCAGTGAAATGGTTGCGAAGAAATCCTGGGACAAA

AACGAAGATGATGATTCTAAGACGTCTGAAGAGAAGTATGAGTCGGGTTCATTAGCCAAG

GGGGCAGATGAGAATGATAGGGAATTGCGTGCTAAGGACGAATGTCCCATGGAGACAGAT

ACCGTGGAGACAGATGATAACGAGCAAGGCAAGAATAATGATATGGATGATGAACCAAGT

GCTTGTGAGGATGCTGATGAGAATACTGATGATGTAATGAACAAAGCTGATGCATATGAT

GATCGAACAGGACCTGAACTCACTGAACTGGACAAAGAGGATGAGGATGTCAACATGGAT

GACACAGAACAAGCCGATGACATGGGTGCTGACAATCCTGACAACGAAGATATGGGCCCA

GAAGAGGGGCAGCAAGAAGATGATGGTGCAGTTGGGTCAGAAGATATGGAGGAAGACGAT

CCCACACATGATGGTGACAATGTGGTTGATAATGAAGGGGATGATGATGAAGATAGAAAT

GTGGAGCCAAACAACATGGAGAAGCAACAGTTGGACAAAATCGAGTCACTTGTACATCCT

TCACAAGGTATAACACCCAGCCAATTGGAAACAGACTCTAACAAAGAATCTGAAGCAAAC

TTGGCCAACAGCATGGACATGAGCAGTGGAGTTGCTCCTTCTGTAGATTTTTCAAATAAT

CAAGTGCCTAGTCTGGAAATCTCTATGCCCAACTCTGGTGAAGGTTCAAGAAATTTGTCC

AACTCCAAGCCAGAGCTGCAGCCTGATGCTCCACCATCACACATCAAGCAGACAAATCCT

TTCAGGAGCATTGGAGACGCATTGGAAGACTGGAAAGAACGAGCAAGAGTCTCAGCTGAT

ACCCAGGATCATCAACCAGAAACTGGACACCATATTGATGATGAGAGTGCTACTGAATTT

CGTTATGTGCCTGAGGGTGAGCAGAGCACCTCACAAGCATTGGGTGACGCGACAGCTGAT

CAAATCAATGACGAGCTGCAAGTCAGGCAACCCATGTTAGATGATGAAACCCGTGCCCAA

GTGGAGCAACCTGATGAGAGGATACCCGGGGATAACAAGCCTGAAATGCCCCATCTTCAA

ACTTCACAATCACGCGTCAACAAGTCCGAAAGTGCCAACAGATTGGAGAGAAGAGATATT

CAGACTGATGCATCCATTGAGGATTTGGTTCAAGATGAAATAATTGACACTTCTGGAGAT

GTTGTTTCATTCAAGCAGCGTCTGACAGATGACAGGGTAGTTCAGCTGGATGCCCTAACA

AGTGACAGGGAGATGGCTACTCAGATGGATCTGGACATAATTAATGAAGAAACGGGAAGG

ACTATCATGGACTGGAGAAATCTCGAATTGGCAACCATGAAATTATCCCAAGAACTGGCA

GAGCAATTGCGCCTTGTCATGGAACCAACTCTTGCAAGTAAGCTTCAAGGAGATTACAGA

ACCGGAAAGAGAATAAACATGAAGAAGGTTATTCCATATATTGCAAGCCAGTTTCGCAGG

GACAAGATATGGTTGCGACGAACTAAACCCAATAAACGAAATTACCAGGTGGTTATTGCT

GTCGATGACTCACGAAGCATGTCCGAGGGCAAGTGTGGAAAAGTTGCAATTGAAGCATTA

GTTACAGTTTGCCGGGCCATGTCGCAACTTGAGGTTGGGCAGTTCGCAGTTGCAAGTTTC

GGGAAAAGAGGGAACGTTAAAGTCTTGCATGATTTTGATCAGATCTTCAATGCAGAAGCT

GGAGTCAAGATGATCTCTAGTCTGTCCTTTGAGCAAGACAACAAAATTGAGGATCAACCA

GTTGCTGATCTCTTGATGCATCTGAATACAATGCTTGATGCTGCTGTGGCAAGGTCGCGC

ACGCCATCAGGGCAAAATCCACTTCAGCAACTCATACTTGTAATATCTGATGGAAAGTTC

CATGAAAAGGAAAATTTGAGGCGCTGTATCAGGAATGTTCTAAACAGAAGGAGAATGATT

GCATATGTACTCCTAGATAGTCATGAGGAATCCATTATGAACTCACTGGAAGCGTGCTAT

GAAGGAGACAAACTAATCTTGGGAAAATATATGGACTCATTTCCGTTCCCTTACTATGTT

ATGCTGAAGAACATAGAAGCGCTTCCTCGTACACTGGCCGATTTGCTCAGACAGTGGTTT

GAGCTGATGCAGGGCGCGAATGAATGATCTTTGGGGACGTTGCCAGCTTGTCAAATTGGT

AAGGGAACCCGTAGGTGTAAAACCATGGGCGTTCTCTAGGTTCATACTGGAACAATTAGT

GGTCCAAAGTCCGAGTTTTGTACAAAATACACACCTGTTGTAGCTTGTTTTACACTTCGG

AACAAATTGTTGTGTAAAGTTTCATTTGTACGAGATATGCTACATTTCCATGTATTGACT

CGTGAGAGGTCAGATTTTGAGTAAAGCGCACATTGTATTACCAAGGGATCAATGCAAAAT

TGCTT

>Traes_5BL_630A8A142

CTTCCTCCTATCGAGCTCTCATCCTCTCACACAACTACAGCAACGCAAATCCAGCAGATC

ATCAAGAGGAGTCGCAAATCCATGGGAGCGGGGATGAAGCGCGCGAGGGAGGAGGAGCCA

GCTGTGTCGCTGGCGCTGTCTCTCAGCACGGACTCCTCGGCGTCGACTACGACGTCGGAC

AACTCGGCCTGTGCGCCGGCGGCAGCGGCCGCAGCGCCAAGGAAGAGGGCACGGGGGGGG

AGGGTGGTGGTCACTTCGGGGGAGGGAGAGTTCGTCTGCAAGACTTGCGGCCGGGCCTTC

GCGACGTTTCAGGCGCTGGGCGGGCACCGGACGAGCCACCTCCGCGGCCGCCACGGGCTG

GAGCTCGGCGTCGGCGTCGCCAGGGCCATCAGGGAGCGGCAAAGGCGCGGGGACAAGGAG

CCGCACGATTGCCACATCTGCGGGCTGGGCTTCGAGACGGGCCAGGCGCTCGGCGGGCAC

ATGCGGCGGCACCGCGAGGAGATGGCGCTCGACCGGTGGTTCGCGGTGTCAGATCAGGAG

GCGGGGCACCAGGCCGCCGCCGACCGGCTGCCTGTCTTGCTCGAGCTGTTCGTCTAGCCT

GCTAGCTGGATCTCAATGCTTGTTAGTTCAGGACCTGTTTGAGTGTTTGTACATGTCTTT

AGAATCAGATAGATTCACTATTGCTCAACGTTGTATAGAGTACGTTTTTGCTGGTTAGAG

TTCTCGTTCTTGGTGGATATTTGCTTTGGATCGGTCCCTTTCTTAACCGACTGACTTGAG

GTTTACATGCACTTGTGTACATAATGAAGACACATTCGTTTGCTCTAAATGCAACTGACA

AATTATCGCAATA

>Traes_5BL_82626DD6E

GTCGTCGAGATGGTTGGGAGCAGCCCGATGCAGGCGGTGCTGGTGGCGCCGGGGGTGAAG

GACAAGAAGGTGCTCGCGTTCCGCCGGGACGCGCTCAAGGAGAAGGACGCCGTCGCCGCG

CTCATGCGCACCATCGCGGCCGGCGGCGTGCGGAGCGCCTTCTACGTCTTCGACCTCGCC

CGGGTCGTCGACCTGTACCGCGGCTGGCGCCGCGCGCTCCCCGGCGTGCGCGCCTGCTAC

GCCGTCAAGTGCAACCCGGAGCCGGCGCTACTCGGCGCGCTGGCCGCGCTCGGGGCCGGC

TTCGAGTGCGCCAGCCGCAGGGAGATCGAGGCTGTGCTCGCTCTCGGCGTGCAGCCCGGC

AGCATCGTGTACGCCAACCCGTGCAAGCCGGAGGCGCACCTCGAGTACGCCGCTGAGGTG

GGCGTCAACCTCACCACCTATGACTCCGAGGAGGAGGTGGTCAAGGTCAAGCGCTGCCAC

CCGGGCTGCGAGCTCATCCTCCGCGTCAAGGGCCCCGACGGTGGCGAGGCCCGGGTGGAC

CTCGGCACCAAGTACGGCGCGCACGCGGACGAGGTCCTGCCGCTCCTCCGCGCTGCCCAG

CGCGAGGGGCTCAACGTGGCCGGCGTGTCATTCCACGTCGGCAGCGGCGCGTCCAACACG

GACGTGTACCGGGGCGCCATTGAGGCCGCGCGCGGGGTCTTCGACGCGGCAGCCGGGCTC

GGCATGCCGCCCATGCGCGTGCTCGATATCGGCGGCGGGTTCATGGCCGGCCCGGCGTTC

GACGAGGCGGCCGCGGTCATCAACGCGGCGCTCGAGCGCTACTTCGGGGAGCTTCCCTGC

GTGGAGGTGATAGGCGAGCCGGGGAGGTACTTCGCCGAGACCGCCTTCACGATGGCGGCT

CGGGTCATCGGGAAGCGCACTCGCGGCGAGGTACGCGAGTACTGGATCGACGACGGCCTC

TACGGCACCCTCAGCTGCATCCCCATGGACCACTACGTGCCGCACCCGAGGCCGCTCGCC

GTGCCGCGCGCCGGCGAGAAGACGTACACGTCGACGGTGTTCGGGCCGACGTGCGACTCC

CTCGACACGGTGGTGACCGGGTACCAGCTGCCAGAGATGAGCGTGGGGGACTGGCTCGTG

TTCGACGACATGGGCGCCTACACCACCGCCTCCGGCTCCAACTTCAACGGCTTCTCCACC

TCGGACATCAAGACTTACTTGGCCTACTCCAGCTGA

>Traes_4DS_E014D9A9F

CCACAGCTTACATCAGCTACCACCAGTGCCTTAGACACTCTTGATCTTGAACAACCATAG

GAAGCAATGGCGTGCTCCAAAACCATAGCTTGCCTGTTCACTATGCTACTCCTTGCTCCG

GCCATCATGGCCGCGGACCCTGACCCTCTCCAGGACTTCTGTGTGGCCGACCTCGACGAC

AACGCAGTCCGGGTGAACGGATACCCGTGCATACCCCAGTCGGAAGCCGGCGACGACTTC

CTCTTCTCCTCCAAGCTAGCCAAGGGCGGCAACACGTCCACCCCGAACGGCTCGGCCGTG

ACGAGGCTGGACGTGACGGAGTTCCCCGGCGAGAACACGCAGGGCATCTCCATGAACCGC

GTGGACTTCGCGCCCGGGGGCACCAACCCGCCGCACATCCACCCGCGCGCCACCGAGATC

GGCCTCGTGATGAAAGGCGAGCTCCTCGTGGGGATCATCGGCAGCAACGAGTCCGGGAAC

AGGCTCTACTCCAGGGTGGTGCGCGCCGGGGAGAACTTCCTCATCCCGCATGGCCTCATG

CACTTCCAGTTCAACGCCGGCGACACCGAGGCCACCATGTTCGTCTCCTTCAACAGCCAG

AACCCCGGCATCGTCTTCGTGCCGCTCACGCTCTTCGGCTCCAACCCGCCCATCCCCACG

CCGGTGCTCACTAGGGCTCTCCGGGTGGACGCCGGGGTGGTCGAACTTCTCAAGTCCAGG

TTCGCTGGTGGGTCTTTCCAGGCCTCCTAAGAGCCTGCCCAAGATGATCAATACATAATC

CGATCTGCATGCTATGCTAGCGAAATTTAATACTAATACATAATCCGACTTGTATATTCA

AGCTTCTGGTTAAGCTCGCATGCAGTTGTAATACGATTGAATAAGTTAGCCTCGCAGTTC

TGGCCATCAAAACCGACACGAGGAATTGAAATGTATTACTTTCTATTGCCATATTTCCTC

TATATATTGAACGCTATATACAAATATTAGTTCTTATATTTGCCCAATTACTTTTTTGCA

ATTCATTCTCAGCTCAGTATACGTAAATGAAATCGGAACCCACTCACACAAGACATACGG

TTCAGTGAAATATTTC

>Traes_6DS_1C67D9765

ATGCGTTTCTTCACCGTTCCTCCAGTCGTGCTGCAGCTGTTCGTGCTGCTCATCTTGGCC

TCTCCTGCCACCGCATGCACCGAGCAGGAGAAGCGCAACCTCCTCGGTTTCCTTGCCGGG

CTGTCGCAAGATGGCGGCCTCGCCACGTCGTGGTGTGACAATGGCATGGACTGCTGCGAG

TGGGAAGGCATCACCTGCAACGGAGACGGGGCCGTTACTGAGGTTTCCCTGGCATCTAGA

GGACTTGAAGGGCGCATCTTGCCATCCCTTGCCGACCTCGCCAGCCTGCAGCGTGTCAAC

CTCTCGTACAACTCCTTCTCTGGTGGCCTTCCTCTGGAGCTCATGTCCTCTGGAAGCATC

ATCATCCTCGACGTCAGCTTCAGCCAGCTCAACGGACCGCTGCCCGAGCTGCCATCTTTG

GTTACCGCCGACAGGCCTCTGCAGGTACTCAACATCTCAAGCAACCAATTCAGCTCAGAA

TTTCCATCAGCCATATGGAAGATGACGAAGAATCTGATCGCGCTCAACGCCAGCAATAAC

AGCTTCACTGGGCACATACCGTCTTCTCTTTGCCTTGGGTCGCCATCTTTGGCTTTGCTT

GATCTCTGTTACAACCGATTGAGTGGTGATATTCCCACCACACTCGGTGATTGCTCCAAG

CTCAAAGTTCTCAAGGTTGGCCATAACAACCTAAGTGGAACTCTCCCTGTTGAAACCTTC

CGTGCTACCTCGCTAGAGTACCTCTCCTTACCCGACAATGGTTTACAAGGAGAACTTGAT

GGAGCACACATGATCAAACTCAGTAATCTGCTTACTCTTGACCTTGGAGGGAACCATTTC

GGTGGCAACATCCCAGAGTCCATAGGTCAGCTCAGGAGTCTGGAGGAGCTCCATTTGGGT

AGCAATAACATGTCTGGGGAGCTGCCATCAACTATGGGGAACTGCACAAATCTCAAAACC

ATTGATCTGAAGTTCAACAACTTCAGTGGAGACCTAGGCAAGGTAAAGTTCGCCACCCTA

CAGAATCTAAAAAGTTTAGATCTCATGAGTAATAATCTCAGTGGCATGCTTCCAGAAAGT

ATTTATTCATGCAGAAATTTGACTGCACTGCGGCTGTCGTACAACAATTTCCATGGTGAG

ATATCCCTGAGAATAGGTAATCTGAAGCACTTGTCCTTCCTATCACTTGTTAGAAATTCC

TTTACGAACATCACAAAAACTTTGCATGGGCTTAAGAGCTGTAAGAACATCAGCGTCCTG

CTTATTGGAAAAACCTTCATGAATGAGGTCATGCCACAAGATGAAACCATTCACGGTTTC

CAGAATCTTCAACATCTCGCCATACACCAGTGTTCGTTGACTGGAAGGATACCTATTTGG

TTATCAAAGCTCACAAATCTGAAGATGCTAGACTTATACAGCAATCAACTCACAGGACCA

ATGCCAAGCTGGATCAACTCCCTAAACCACCTCTTTCGGCTGGATGTATCTAACAACAGT

CTTACTGGGGAAATCCCAATCACCTTGATGCAGATGCCAATGTTTAAATCAGATAAGGCA

GCCATCTATTCGGACCCAAGCCTCCTTGACCTTGATCTAATTATTTATGTTGCGGAATCA

TCAACAGTTCAATACCGGATTGACAGTGCTTGGGCCAAAGTGCTGAATCTCGGCAAGAAT

AAATTCACAGGTGTGATTCCACAAGAGATTGGTCAGCTGAAAGCACTCCTTTTCCTGGAC

TTGAGTTCCAACAACTTTTATGGAGTGATCCCACAATCAATCTGCGACCTCACGAACCTC

CAGCAACTAGATCTGTCTAATAACCATCTGACGGGTGAAATTCCTGCAGCATTGGAGATC

CTTCGCTTCCTTTCACAATTCAACATTTCCTACAACGACCTAGAAGGACCTATTCCAACA

ACAGGCCAGCTGAGCACGTTTCAGACTTCTAGTTTTGACGGGAACCCAAAGCTGTGTGGC

TCTCTGCTTATTAGAGATTGCAGTTCAGTCGAAGAAGCTCCCGTGCACATCATCTCTGCA

AAAGAATGCAGCACTAAGGTCATCTTTGCCATTGCCTTTGGTTTGTTCTTTGGGGTAGGA

GTGCTATATGACCAGTTAGTATTATTCAGATATTTTGGCTAA

>Traes_1DS_665A5B1DD

CAGAAGGTGCCGGAGAAGGAGAACGCCCCGGCGAGGGGCCGGAGGGGCGCAGCCGGCGCG

GCGGCGAGGCTCATGGAGAAGATCATGCCCGGCGCGCCGGCCATGAAGCCCAAGGCCAGC

AAGAAGGACCAGTACCTGCTCAAGCGCCGGGAAGACGCGCGCGCGCCGGCGCTGCCGCCA

GCCGCGCTCCCGGACGCGGCTCCCGCCCCGGACGACGGTGGCCCGCCGCCTGGCTTCCCC

CCGGCTGAACCGCAGACGCCGCCGCTACCCAGCAGCACCGGAGGCGCCGACGAGGAGGAG

TTTATGCTGCAGAGACGCGCGCTCCCTCCTGCCGACCAGGCGAGCGACGGCGGCGCAACC

GCAGACGCCGCTGCACCGAAGAAGGTGGCCAAGCCCAAGAAGGCCCGCAAGCGCGACAGC

GAGGAGCCGGCCGAAGACGTGGCCGCCGCCGACGGCGAGCCCAAGAAGAAGAAGAAAAAG

AAGAAGCTCGCTGAACTCAACGGCGGCGTGCCCTCTGCCGCCCCCTCCGGCGACGCCGGC

GCTACAAAGCCCGCAGCTTTCTCACCCCCCAAGGTCGACCTTGACGGCTTAGATCTGACA

CAGGTAATATCCGACCTTCAAAACCTTCCGCTGCTTCCCTTCTACGGCGCCGGCAGACGC

ATCTCTGACGCCTCTCACTCCTTCATCCTGGCGTTCCGCTCGAAACACTACAAAAAGAGC

TATGAGAACGATCCGCCGGAAGAGTCAAAGAAGGGCTTGGATAATAAGCCCAGTGTCGTT

GCCGCCGCCGCCGCCGCCGTCTCTGACGGCCAGCCCCCCAAACCGTTGAAGAAGAAGCCG

GTCATGAGGCCCAGCGACCCCACCAGTGCTGGCGTGAAGCGCCCGCCGTCTGACCGCCAG

GAGGAGATTGCCTCCAAGAAGAAAATCAAGCTGGACAAGATTAAGACTCTGGCCGCCGAC

AAGAAGGCCGGGCTGGAGCAGAAAGTCGCCACCGCCACCGCAGCTGCGGGAGGCGGGACG

ACGGCGGCGGCTCAGCTGCCACGGGCTGGCCTGAAGGAAAAGGCTCTGGCGGCAGCCAAG

AAGAAGGTGCCAGCGGCGGCGCCTGTGAAGAGGACGCCGTCGCCGACGGCGCTGATGATG

AAGTTCCCGCTGAAGAGCACGCTGCCGTCGGTGGCCTCCCTCAAGGCGAGGTTCGCGCGC

TTCGGGCCGCTCGACATCAACGGCATCCGCGTGTACTGGAAGTCCCACATGTGCCGGGTC

ATCTACAAGTTCCAGTCGGACGCGGAGGCCGCGCTCAAGTACGCCAAGGCCAACGCCATG

TTCGGGCAGGTGGCCCCCAACTACTACCTGCGCGGCGTCGAGGGAGGGTCGGCCGGCGCC

GACCCAGGGCCTGAGCCGGCCCCTCCGCAGCGCTCTGACCTGCGGCTCATGGAGACCACG

CCGTTCAGGCCTGGGAGCTCCGGCAATGGCGCTCCGCTGACGCTGTCCAAGGCGGTGCCG

GCGCGCATGTCCGTTGGGCTGCCCAAGTCAATCCTGAAGAAGAGCAACGACGAGGGCGGG

CTGGCTGCGGCCAGCGCGCTCCGAGACTCCCCTCGGGTGAAGTTCGTGCTGGACGGTGGG

GACAGCATGGTCGAGCCACCTCCATTGCCACCGAGCTTCGATGGAAACAATGGCCCGGAC

ACTGCTGCACCAGTGAGCAAGATCGCGAGGTCGCTCGGCTTCGCTCAGCCGCCTCTGCAG

CCGCCGGCACGCCCTGCGCAGCCCAACCTGCAGCCACTCATGCGCCCACAGCACCAGCAA

CTGCAGCCGCCACGCGCACCTGATTCACAGCCACTCCCACCACCGCCGCCGCTGCCATAC

CAGCCTCGCATCAACGAGCCATCGTCGTACCAGCAGCCCCGTATCAGCGAGCCATCGCCG

TACCAGTCTCGCGTCAACGAGCCACAGCTGTACCAGCCTCGTCGCACGGACGCGCCGCCA

ATGTTCAACATGCAGCCGCCGTACCAGCCTCGCCAGAGTGACGCGCCGCCACCGTTCAAC

ATGCAGCCGCAGTACCAGCCTCGCCACAGCGACGGACCGCTCGCCCTCCCTGGACAGCCG

TCGCTGCCGCCGTACCCTCCCCGTGCCGGTTTCCCCGGACAGCAGTACTCTTCCCGCTCC

GACGACATGCCGCCGGCTCACTTCGACAACAACCCCAGCAGCAACGCCATGCCGGCGTGG

AAGAGGGGGGAGAAGGAGTTCAACGAGGAGCTGATGAGGGTGATGCTGGGGATCGCCAAG

CTGGTGGAGCCATTGATGGACAAGAACGGCAACTTCCCCTACCACCTCTTCAGCCGGTCA

GCATGA

>Traes_5BL_1315614DC

TAGGCTGCCGCGACAGGAGAGCAGAAAGCCGAGGTGGTCACAAATCACATTCTGAAGGTC

TTGGGGTTGGATATCTGTGCCGATACACTGGTAGGCAATAATATGTTGAGAGGCATATCA

GGAGGGCAAAAAAAGAGAGTAACTACAGCTGAGATGCTTGTCACACCAGGACGAGCCCTT

TTCATGGATGAGATATCAACTGGACTTGACAGCTCAACAACCTTCCAGATTGTGAACTCC

ATCCGACAAACAATCCACATTATTGGTGGAACAGCAGTCATTGCCTTGCTACAACCTGCA

CCAGAGACATATGAATTGTTTGATGATATAATTCTCCTCTCAGACGGTCAGGTTGTCTAC

AATGGCCCTCGAGAACATGTGCTCGAGTTCTTTCAATCAATGGGATTCAAATGTCCTGAG

CGAAAAGGCGTAGCTGACTTCTTGCAAGAAGTTACATCAAGAAAAGATCAAGGACAATAC

TGGATAAACAGTGATGAGACATACCGATATGTTCCTGTTAAGGAGTTTGCGGAGGCATTT

CAAGCTTTCCACGTTGGTCAAGCTATAAAGAGTGAGTTAGCAGTGCCGTTTGACAAGAAT

GGAAGTCATCCTGCAGCTCTGAAAACTTCACAATACGGTGCCAGCATGAAGGAACTACTC

AAAGCTAATATCAACAGAGAGATACTGCTCATGAAAAGAAACTCCTTTGTGTATATATTC

AAGGCCACCCAGTTGACACTCATGGCGATCATCGCAATGACTGTCTTTCTGCGCATCAAT

ATGCATCATGACTCGGTGACAGATGGAGGGATATACATGGGTGCTCTCTTCTTTGGGATT

TTGATGATCATGTTCAATGGTTTAGCAGAAGTAGGTCTAACTATTGCAAAGCTCCCTGTT

TTTTTCAAGCAACGGAATCTTCTCTTCTTTCCAGCATGGACATACACGTTGCCGTCATGG

CTCATTAAGACCCCTCTCTCCTTGCTCAATGTAACAATTTGGGTCGGCATAACATACTAT

GGCATTGGATTTGATCCCAACGTACAGAGATTTTTTAGGCAGTTCCTGCTGCTCTTTTTA

ATGAACGAGGCATCGTCTGGACTCTTTCGCTTCATTGCTGGTCTTGCAAGGCATCAGGTT

GTTTCAAGCACCCTCGGTTCCTTCTGCATACTAATTTTTATGCTCACTGGTGGATTCGTC

CTGGCAAGAGAGAATGTAAAGAAATGGTGGATATGGGGGTACTGGATATCACCCCTGATG

TACGCACAAAATGCACTATCGGTGAATGAGTTCCTAGGTCACAGCTGGAACAAGACAATC

CCCGGTTTCAAGGAACCACTTGGAAACCTAGTTCTGAAATCTCGAGGGGTCTTTCCTGAT

ACGAAGTGGTACTGGATTGGTGCTGGTGCCTTGCTCGGATATGTGCTACTATTTAATATC

CTCTATACCGTCTGCCTCACATTCCTCGACCCATTTGACAGCAACCAGCCAACAGTATCT

GAAGAAACATTGAAGATAAAACAAGCTAATCTCACTGGTGAAATTTTAGAAGAATCATCG

AGAGGACGGGTTAACAACAGTACAATAGCATCTAGAGATACTGAGGATGGGAGCAATGAT

GAATCAACTTCCAATCATGCAACAGTGAATTCTAGTCCAGGCAAGAAAGGAATGGTCCTC

CCTTTTGTGCCTCTCTCCATCACATTCGATGATATAAAATACAGTGTAGACATGCCACAG

GAAATTAAAGCACAAGGTGTGGCAGAGAGTCGGTTGGAACTACTGAAGGGTATCAGTGGT

TCATTTAGGCCAGGAGTGCTTACAGCTCTTATGGGTGTCAGTGGTGCCGGTAAGACAACA

CTGATGGATGTGTTGGCTGGACGGAAGACCAGTGGATACATAGAGGGCAACATTACAATC

TCTGGTTATCCAAAGAAGCAAGAAACTTTTGCTCGTGTATCGGGATACTGTGAGCAGAAC

GACATTCATTCACCAAATGTGACCGTCTATGAGTCTCTTGCATTCTCTTCATGGCTCCGA

TTACCTGCCAATGTTGATTCCTCGACAAGAAAGATGTTCATTGATGAGGTCATGGAATTG

GTGGAACTCTCCCCCTTAAGAGATGCATTGGTTGGATTACCTGGTGTGAGTGGATTGTCG

ACTGAGCAAAGGAAAAGGCTAACAATAGCAGTGGAGCTGGTTGCTAACCCTTCCATCATT

TTCATGGACGAACCAACATCTGGACTTGATGCACGGGCAGCAGCCATTGTCATGAGGGCA

ATAAGGAATACTGTAGATACAGGACGGACAGTTGTTTGCACCATTCATCAACCAAGCATC

GACATATTTGAGTCTTTTGATGAGCTCTTCCTGATGAAACGAGGAGGTGAAGAGATTTAT

GTAGGTCCACTAGGCCGGCACTCATGTGAACTGATTAGATATTTTGAGGCTACTGAAGAT

GTCAGAAAGATTAAAGACGGTTACAATCCTTCAACATGGATGCTGGAAGTGACTAGTGCA

ACACAAGAACAGATGACTGGGATTAACTTCAGTCAAGTATACAAGAATTCTGAACTATAT

CGGAGGAACAAAAATCTAATAAAGGAGCTAAGCACACCTCCTGAAGGTTCAAGCGACCTA

TCCTTTCCAACCCAATACTCACAAACTTTCCTCACACAGTCTTTTGCTTGCCTGTGGAAG

CAAAGCCTGTCATACTGGAGAAACCCTCCATATACAGCTGTCAAGTACTTCTATACTACA

GTGATTGCACTGTTGTTTGGGACAATGTTCTGGGGCATTGGCAAAAAAAGACACAATCAA

CAGGACTTGTTCAATGCCATGGGTTCCATGTATTCCTCAGTTTTGTTCATGGGGGTGCAG

AACTCTGCCTCAGTTCAGCCAGTTGTGGCTGTTGAGCGCACGGTCTTTTACAGGGAAAGA

GCGGCTCACATGTACTCACCTTTGCCATATGCATTGGGACAGGTTGCAATTGAACTTCCA

TACATCTTTGTTCAGTCCTTAATATATGGTGTGCTAGTATATGCTATGATTGGGTTCGAG

TGGACAGCTGTCAAATTCTTTTGGTACCTGTTCTTCATGTACTTCACTCTAGCTTATTTC

ACATTTTACGGAATGATGTCGGTGGGCCTGACTCCAAACTACAACGTTGCCTCTGTTGCT

TCCACGGCATTCTATGCTCTCTGGAACCTTTTCTCAGGATTTATTACACCAAGAACTAGA

ATCCCGATATGGTGGAGATGGTACTACTGGCTCAGCCCTATTGCATGGACACTGAATGGT

CTGGTCACTTCACAGTTTGGAGACGTAACCGAAAAGTTTGATAATGGCGTGCGGGTATCC

GATTTTGTAGAAAGCTATTTTGGGTACCATCATGACTTCTTGTGGGTGGTCGCTGTGGTG

GTCGTCTCATTCGCTCTTCTCTTTGCTTTCCTATTCGGGCTCTCAATCAAGCTATTCAAC

TTCCAAAAGAGATGAGTGATCAATGAAGAACATATCCAAGTTTGACCAAAGCCCACTAAG

CAGAGATAGTACAATAGGACTACGTCCTTGTAATTGTATTGGAGACATGTGAATAGTAGC

ATAGCATATATTTTGAGAGCACAGTGCCCATATCGAATGTATAGTGACAGTGCACATTGT

GCCCAGTGCCCTAGGAGTACAGTTTTGCAAGTTGTATAAATCAAGTAATTTGCCACACCG

TTGATTTGCTCTAACACCTTTGGAGCGCTCACAAAACAAAATGGTGGCATGCTGTACTCG

AAATTTGACCAATA

>Traes_4BL_A7C76FFE4

GTCGCCTTTAACTCCCCCCTCCTCCGTTACCGTCTCCCCCCTTCCCACCTCCCCCACCCG

CTGCTCATCAACATTCAACAGGAATTCGATTTGATTTCGACTGATTGCTTGATTGCAACT

CCGCCGCGAGAGAGGCCTTCGCTGCGCTACGTTCTTGCCGGGGTTGTTCTTCTTCGATTT

TTAGGGTTGCGGAGCCCTTGGATTTCGCGTAGGAGAGCCTCCTCCCGGCCGTGTGATGGA

CGCGAGGGAGATGTGAAGCCTGGCTGTTAACTGATGCTCGTGGAGAGATTCGGAGGAGAG

GAGCGCGCGGTGAGGAGGCGGTGGAGGCGGAAGCGCCGGAGAGTTGGGCACCAGCGCAGC

CACGGACGACGCGCAGCCGCCGGGGGACGAGGCCTGGAACAGGGCTGCTCCGCCGCTGAC

GGCCGCCGCCGCCGCCTCCATGGGGGAGTCGCTGCTCACCGCGCTCTCCATGGACACCAC

CACGGCCCACCACCCGCACCAGGGCCCCTCCACCTTCCTCTCCATGGACACTGCCTCCCA

CGACGAATTCGACCTCTTCCTCCCGCCGCCAGGCCCTTTCCAGCGCTGCCTCCATGCCGC

CGCAGCCGCCCCCCCTGACATCAACCTTCCCCTCGCCGCTGACCCGTCCCCTCCCCCTCC

GGCCCTGCAGACCACCACCCTCCATGAATCCAACGTCGACATGCTAGATGTTGGCCTCGG

CTGCCCGCAGCTCTATGACTCNNNNNNCTGCTGCTGCCGCCGCCACCTCGGCCCCAGTGT

CCACCACAACAACTGTCCATGTGTCTCACACCAAGAGCTCCGGTTCCAGCGCCGCACGCA

AGTGCGTGAAGCGGAATGATAGCATCTGGGGTGCATGGTTCTTCTTCACCCACTACTTCA

AGCCGGTTATGTCGGCTGATAAGGGCAGCAAGGCCAAGGCACCCACTGCTGCTGGGAATG

GTAATAGTGCCACACTGGATGCTTTCCTGGTGCAGCACGACATGGAGAACATGTACATGT

GGGTGTTTAAGGATCGGCCGGAGAATGCCCTGGGGAAGATGCAGCTGAGGAGCTTCATGA

ATGGTCACTCGCGCCTTGGGGAGCCACAGTTTCCTTTCAGCGCAGACAAAGGGTTTGTGC

GCTCACACCGGATGCAGCGTAAGCACTACCGGGGGTTGTCAAACCCGCAGTGCCTTCATG

GGATTGAGATTGTGCGGACACCAAACTTAGTGGGTGTTCCTGAGGCTGATATGAAGAGGT

GGTTTGAGCTCACTGGGAGAGATGCCAATTTCTCGGTTCCCACTGAGGCGGATGATTTTG

AATCATGGAGGAATCTGCCGACCACGGAATTTGAGCTCGAGAGGCCTGCAACTGCTGCTC

CAGCAAAGAGCACCTCACATGGCCATCACAAGAAGGTGCTGAATGGTTCTGGCCTTAACC

TGTCAACACATGCATCAAAGCATGGTTCTGGGGATGGTATGGACATCTCAGGCGTGTGCC

ACAAGCGTAGGAAGGATTCCTCCCCCTCTGCCATGGAAGAGGATTGCAGCAATTCAAATT

CAGACAAGGTTCAGGACATGGATGTGAGCCACACTTTTGAGCNNNNNNNNNNNNNNNNNN

NCACCGGTGTGATGCGTCATGCTTCTGGGCCAGTAACTGCCGCAAAAACAATATATGAAG

ATAGCAAGGGCTACTTGATCATCATTAGCCTGCCATTTGCTGATATACAGAAGGTGAAGG

TTACCTGGAAGAATACTCTTACGAATGGCGTCGTCAAGGTATCATGCACTAGTGTCGGAC

GGATGCCATTCTTGAAGCGACATGATCGGACCTTCAAGTTGACGGATCCTTCACCTGAGC

ATTGTCCACCAGGGGAGTTCATCCGGGAAGTTCCACTGCCTACCCGGATCCCAGAAGATG

CTACTCTGGAAGCATACTGTGATGAATCAGGAACAGGCCTAGAGATTATTGTCCCTAAAC

ACCGTGCTGGACCTGAAGAACATGAAGTCCGTGTGTCCCTGAGGCCTCCCTCATCATGGT

GCCAATGAAAACATGTTGAAGTATGCGGGGAGACAAGGTGTGGATGTTACCATACAACAA

ATGCAGCTCAGTTTTGCTAATTGTGACGGAGCAGTCCTAGTCGATTGAAGCATTCTGGTA

GTTGGAAGTTGGCAGCTGTGGAAATTTAGGGGCATGCTACAAGCCACAAGGAACATATCT

GGATTACTCACTCGCTCTATGTTTTGGTATCGTTGCTGGGGAGTACCTTGTGTATTATCT

TTTGCTTTTGTGGAATTTTTCTTTGTATGAAAGGTTTGCCGCCCTGCTCAATGTGCTTCC

ATCTGTGCATTACTCTGGTGAGCATTCTGTTTAGGCATTTATGGTCAGTATTCATAGGTT

GGTGAGAAGTCTGCTTTGTGGGTGTTCCTGAAAATTTCATCCAGGAAAGGAGTTCATGCA

AGTGTTTCTAGGATTTGTCCATGTTTTGTGGGTACTATATTGTTTTTATTAGCATTCAAA

CGCTTTCCAGGAAATTGCTGGAAGGCGAGTGCAATTGTTTGAAGGTACCAGTGTAAACAA

TCCGTCATCAATATAGATTTTAGTCTTTATCAGTTATGTGCACTGCTGTAGTAATTTGAA

TTCACTTTCATATGCTTGGGT

>Traes_1DL_1DEDB0903

GGAGAAGGCGCTTAAGGAGTACCTCCCGTTCATCGTCGCCACGGCCAAGGCCATCAAGGA

CCAGGAGAGGAGCCTTAGCATCTACATGAACGAGCGCTACGACGAGTGGTCCCCGATTGG

CCTCCAGCACCCCTCCACCTTCGACACCCTTGCCATGGATCACAAGCAGAAACAGTCGAT

TGTCGACGACCTTAACAGGTTCATCAAGAGGAAGGACTACTACAGGAGGATCGGCAAGGC

ATGGAAGCGCGGGTACCTGCTGTACGGCCCTCCAGGTACCGGCAAGTCCAGCCTCATCGC

CGCCATTGCCAACCACCTCAGGTNNNNNNNNNNNNNNNNNNNNNNNNNGCTCACCGGGGT

CGACTCCAACTCCGACCTTAGGAGGCTTCTTGTCGGAATGACCAACCGGTCCATTCTCGT

GGTTGAGGACATCGACTGCACCATCGAACTGAAGCAGCGGGAGGAAGAAGACGAGGAGCA

ATCCAAGTCCAGTTCTACAGAAAAGAAGAAGGCAGAAGACAAGGTAGTCACACTGTCTGG

GCTGCTCAATTTTGTCGATGGCTTGTGGTCGACAAGTGGGGAGGAAAGGATCATCATCTT

CACGACCAACTACAAGGAGCGTCTTGACCCGGCACTTCTGCGGCCTGGCAGGATGGACAT

GCACATCCACATGGGGTACTGCACCACGGAGGCTTTCCGAATTCTTGCCAACAACTACCA

TTCCATCGACTACCATGCCACCTATACAGAGATCGAGAAGCTGATCGAGGAGGTGACGGT

GACGCCTGCAGAGGTTGCCGAGGTCCTGATGAGGAACGACGACACTGATGTTGCGCTCCA

TGATCTTGTCAAGCTCCTAAAGTTAAAGAAGAAAGATGCCTCTGAGATCAAGACTGAAAG

TAAGAAGACGGAGAAGAAAGATGCCACTGATGACATCAAGATTGAGGGTACGCAGGTGGA

TGGGAAGAAAGATGGTGGTGAGATCAAGACTGGAAGTGTGCAGGTGGAGGAGAAGAAAGA

TGACAAAGAGGTGGTGGTAAAGAATGTTCTTACAGAAAACGGAAGTAGTTAAGAGTGCGG

AAGTTAACGGTGACGAAAGAGTGCGCTTACCATGTAGATAGATCCTACAGGAGATCCATT

GCAGCATACATATGTTGTGATCATTTTGACAACTAAAAACATTGCATTTTTGTAGTTCGA

TGCATCTGTACTTTTTAACAGTGTGTTTCTAAA

>Traes_5BL_D4B7D4399

CAGTCACTGACACTCCTTCTCAGTCTCTACAGTCCTTAACAGACACTGAGACGTTGGCAA

TCTTTCTTGAAAGGAATCGGAGTACATGGCGTCGACGTGGGGGGCCAAGACGGTAGGCCC

GACGGTGCCCTCGGCGTACCTGGACAACCGCCTGCCGGACGACGCCTCCTACGGCTTCCA

CCTGCACACGCCGACGACGGCCGCGACCAAGGCGTGGTTGGACGCCCGGCCGGCGCGCTC

CGTCGCGTACGTCTCCTTCGGCAGCATCGCCGCGCTGGGGCCGGAGCAGATGGAGGAGGT

GGCGGAAGGCCTTTTCAACAGCGGCGCGTCCTTCCTGTGGGTTGTCAGGGCCTTGGAGAC

GTCCAAGATCCCCGACGGCTTCGCCGACAAGGCGGGCGGGAGCGGCTTGATCGTGCCATG

GTCGGCGCAGCTGGAGGTGCTGGCGCACGGCGCC

>Traes_2DL_452B7BC44

GTTCATCACAGCACGAACGACATGGCTGCTGCTCCGGTGGTTCTGCCGCGCATGAAGCTG

GGCTCGCAGGGGCTGGAGGTCTCGGCGCAGGGCCTCGGCTGCATGGGCATGTCCGCGGCC

TACGGCGAGCACAAGCCCGAGCAGGACATGATCGCGCTCCTCCGCCACGCCGTCGCCGCC

GGCGTCACCTTCCTCGACACCTCCGACATCTACGGCCCCCACACCAACGAGCTCTTGCTC

AGGAAGGTGCTGCAGGGAGGGGCGAGGGAGAAGGTCCAGCTGGCCACGAAATTCGGCATC

ACGCCGGCCCGGGAGGTCCGCGGCGACCCGTACGTGCGGGCGGCGTGCGAGGGCAGCCTC

GCTCGGCTCGGCATCGACTGCATCGACCTCTACTACCAGCACCGCATTGACAAGAGTGTT

CCCGTCGAGATCACGATGGGTGAGATCAAGAAACTAGTCCAAGAAGGAAAGGTGAAACAC

GTCGGGTTGTCGGAAGCCTCGGCCTCGACAATCAGAAGGGCACACGCAGTTCATCCGATC

ACCGCAGTTCAGCTGGAGTGGTCTTTGTGGTCAAGAGATGTTGAAGAAGATATAATACCA

ACTTGCAGAGAACTTAGCATTGGAATTGTGGCGTACAGTCCACTAGGCAGAGGTTTTCTA

TCCACTGGACCTAAACTGTTGGACACATTACCAGAGGACGATTTCTGCAAGAATCTCCCA

AGATTTCAACCCGAGAACATGGAGAAGAATGCGGCGATATTCGAGCGCGTGAGCGAGATG

GCTGCGAGGAAGGGTTGCACGTCGTCCCAGCTCGCGCTGGCTTGGGTTCACCACCAGGGA

AGCGATGTGTGCCCCATACCTGGCACAACGAAAGTTGAGAATCTCAACCAGAACGTGAGA

GCGCTTTCAGTGGAGCTCACGGCTGAGGAGATGGTTGAGCTCGAGTCCTACGCTGCCATG

GATGCGGTCCAAGGTGATCGTTACCACATCACGTTCCTCAACACCTGGAAGACTCCGAGA

CCCCTCCCCTGTCATCCTGGAAAGCCACTTAACTGGGTATCATCGGAATCAATCACTATG

TTGATGCTTCGTATCACTGATTTGAAATAGCTGCAAATCAAATAATTCCATGAGCACTCC

GTGCATGGATGAAATAATGGAACCACCAATTGTTTGCTAATGTAGTACAATTCCTCCCCA

TGATTACGTTCACGGTACTAAAAAAT

>Traes_1DS_933A067FA

ATGATGCCCCTCGGACGGTGTCCCAGTCCCACCCGGCCCGAGATCCCCAAATTTCCCCAA

CCCCCGAAACCCAAACAGCGCAATCCCAAACCCAAACCTAAGCCCAGACCGAGAGACCCC

GCACCCAAATCTAGGGTTTCGCTTCCCCGCCGCAGGATGGCTGCCCCCGCCGCCGCCGGC

TCCGGCGCCGTCGTGCCCGCTGGTGACGGCGAGGGCTGGACCCCCAGTTTCGGCGACATG

GTCTGGGGCAAGGTGAAGTCGCATCCGTGGTGGCCGGGCCACGTCTACAGCCTCACCCTC

TCCGACGATCCGGAGGTGCATCGCGGCCACCGCCATGGCCTCGTCCTCGTCGCCTTCTTC

GGCGACGGCAGCTACGGCTGGTTCGAGCCGCACGAGCTCGTCCGCTTCGAGGACCACTTC

GTCGAGAAGACCTCGCAGGGGGGCAGCCGCACCTTCCCCGCCGCCGTCGCCGAGTCCCTC

GACGAGATCTCGCGCCGCAGCGCGCTCGCGCTCCTCTGCCCCTGCCGTGGACCCGACACC

TTCCGCCAACACAACGAGGATCGGAGGTTCCTCCTGGTGAACGTGCCCGGGTTCGACAGC

AACGCCGAGTACCTCCCCGAACAGGTGACGGCCGCGCGGGAGCGGTTCGTCCCGCAGAAG

ATGTATGACTTCCTGCAGAACGCTGCCGTGCAGCAGCGCGACGCGGCGGAGACGGCCGCA

CGGACCTTGCCTGGGATCGAGATGGCAGCGATGCTCATGGCGTACCGCCGGTCTAGGTAC

GAGAGGTACGACCTCACCTACGCCGAATCATTTGGGGTGGATTCGAAGAAGGCCCTTGAA

GGCGAGGTCAAGGCTGAGAATGAACGGTCTCAACGAGGTATCGACACTCACATGCCCAAT

CCTAGTAGTACTACTATTCATTGTTCTGTTATTGTTTTTGCTATGTACTTGAACTTGATG

GGCAGATTACCTGATACTAATCCTTTTGATGTGAATAGTGAACTGGATATTGGTTCTTCA

AATATTTATTATTTCTCTTTTGTTGAACTGTGTGTTGCATGA

>Traes_2DL_E9405C407

ATGAAGGTGCTAGGCGCCGAGATCGGGTGGCCTGCGCTCATCGCTGATGTGGCTGTCATC

GTCGTCGGCCTGGCGCTCTCGGCGTCCGGGACATGGACGTCACTCGCGCACATGTTTGGT

GCTTCCAACGCGTAAGCATCACTGCATCAACAAGGACATGGATTCTTCGGGAATGCTCAA

GTGTACACTACTGTAATTAGCATTGAGACCCGTCCAGTTCAGCTGCCATGTCGAGTTGGG

CATGGTAGTATCTGGTCCCTTGCATTGCTTGTAAAGAAGAGGCAAATGGCATAGGCCCTC

ATGGTATCCCACGGCGAAATCTGTATTGTAATAGATGTCCCAAGGATCAAGCGACCACAA

CGAACCTGTGTGTTGTACCAGAGTATTTATGTTACTATGCAAGATGTTCTTTTTGTCTAA

AATTGTTAGGGTATGTTTGAGCAAATTTGCCTAGTTCTGAAAGGATGTGGT

>Traes_2AL_176C953B9

CTGTCGTGCACTACTGTACACACGCGGGAATTCGATGGCGGTTGACTCCATGGAATCGGT

GGCCTTGGTCGCGGTGCCCTTCCCGGCGCAGGGCCACCTGAACCAGCTCATGCACCTCTC

CCTGCTGGCCGCGTCGCGGGGGCTCTCCGTGCACTACGCGGCGCCCGCGGCGCACCTCCG

GCAGGCCCGGTCGCGCCTGCACGGCTGGGACCCGCAGGCCCTCGGCTCCGTCCGGTTCCA

CGACCTCGGCGTCTCGGCGTTCGAGTCCCCGGCGCCCGACCCGGCCG

>Traes_3AS_552E74797

CCCGAGGCGCACGTCTTCAAGGCCGACCTCCCCGGCGTGAAGAAGGAGGAGGTCAAGGTG

GAGGTGGAGGACGGCAACGTGCTCGTCGTCAGCGGCGAGCGCACAAAGGAGAAGGAGGAC

AAGAACGACAAGTGGCACCGCGTGGAGCGCAGCAGCGGCAAGTTCGTCAGGCGCTTCCGC

CTCCCCGAGGACGCCAAGGTGGAGGAGGTGAAGGCCGGGCTGGAGAACGGYGTGCTCACC

GTCACCGTGCCCAAGGCCCAGGTCAAGAAGCCCGAGGTGAAGGCCATCCAGATCTCCGGC

TGAGTATTCGGGTCAGAGCCAGTGTGGTTCATGTGCGTGCGAGTTCTGCAGCCTAAAAGT

ATGCTACAGTTGTGTCTTTGTTCTTGCCGAGTCTGGTCTCTGTA

>Traes_4BL_EB9CF2010

AAAGCACCGATCAAAGAACGATCCCTCCCAAATCAATCGCTCACCCTTCCGCTTCCTAAC

ACCAGCTAAATCGTCGATTCCTTCGACCAGCAGCTCGAATCCCCACCGACAATGTCGCTG

ATCCGTCGCACCAACGTGTTGGACCCCTTCTCCCTCGACCTCTGGGACCCCTTCGACGGC

TTCCCCTTCGGCTCCGGCGGCAGCGGCAGCCTCGTCCCGCGCACCTCCTCTGACACGGCG

GCCTTCGCCAGCGCGCGGATTGACTGGAAGGAGACGCCCGAGGCGCACGTGTTCAAGGCG

GACGTGCCGGGGCTGAAGAAGGAGGAGGTGAAGGTGGAGGTGGAGGACGGCAACATCCTC

CAGATAGCGGCGAGCGGAACAAGGAGCAGGAGGAGAAGACGGACACCTGGCACCGCGTGG

AGCGCAGCAGCGGCAAGTTCCTGCGCAGGTTCCGGCTCCCGGAGAACGCCAAGGCGGAGC

AGGTGAAGGCGTCCATGGAGAACAGCGTGCTCACCGTCACCGTGCCCAAGGAGGAGGCCA

AGAAGCCCGAGGTCAAGTCCATCCAGATCTCCGGCGCGATGAGATGGACTGCAGTCTGCA

GAGCAGTGGTGTGGAGTTTCGCCCGGTCGTCGCGATGAAATAAAATCAGTCAGGCTGTCT

AGTGCTCTAATCTTGTGTGCGAATGGTCCGATGTTGCAGTCAGTGTTTCGAGTCTGTCGC

GTCTGTGTTCGAGTCCACCTTGTTCATCAGCAACAAAGTAAATCCATTACCTTCTGCTAA

TTTGTGAATGTTCCCTCACACTTGAAAGCAATTTCAACTCGGAATAAATAGTAAACAATA

CAATCAAGCCGTTTAGTGGGATGCCAAAGGTGGGATCCGCGCTAGTAGGAGTAGGACGGC

GAGCAGCTGGTGGTCTGTTTTGTCCCGAAAATATCATTACCGAAACCAACATTCGATGGA

AGATGACCATGTTTTAATTTTTTCAAAAAATATAAAA

>Traes_4DL_A61E2C0A4

GTCAAGTGGGCCCACATGTCAGTGGCACATTGTTGATATGTCTTTGTGATGGTGCAGAGC

GATCTGCATTCATTTTGTTTCTTATGTTTATATATCTCATACTGTAAATAATATGAACTT

TACTATAAATTCCGACCTTCTGTACTTTCTGATTAAAAATCTCAATTCCATGTATATTAA

GGCAACTCCAGTAGTTTTAAAATTTCTACATAATCACATATATCTGTTTTGCTTAACTGA

TTTGTTGTTACCCTCCAGCTGGAAGTCTTGATTGCCAAGGATGGTAAAGATGTGGAAATC

CAGTCTGTTGTAGCGGAAGTTCCTGATATCTTACTCAAATGTGTTAATCGGGATGAGGCT

GCATTAGCTGTTGCACAAAAGGTTTTCAAAAGTTTGTATGACAACACATCAAATAGCGGT

TCTGTCATGTGGTTTCTAGCAACCTTAGTTGCGATAAGAGATGTATGCAAGCTTGTTGTC

AAGGAGCTAACAAATTGGGTAATCTATTCGGACGATGAGAAGAAGTTTAATAGTGAAATC

ATTTTTGCCCTTATTCGTTCTGATTTGCTGGATGTCAGGGAGTACAATGTTCAGTTAGCA

AAGCTAATTGACGGTGGAAGAAACAAGATTGCAACAGAATTTGCCATGTCACTTGTCCAG

ACATTGATTACTCAGGACTCCGCTAGTATCTCAGAACTCCAGGATGTTGTTGATGTGCTA

TCAAAGATTTCAAGGAGGTCAGGTTCACCTGAGTTGATGCAACAGGTGAATGAGATTGCA

AGGAATAATGCCAACAATGCTCCTGGTTTTGCTTTTGGGAAGGATAAAAAGGTTCTACCT

AACCGGGCAAACAAAGAGGAAAACAGTGTTAACGACGCCACTGTAGCTGATTCTGTTGCT

TTTCAAGACCAGGTTGCACACCTATTTTCTGAGTGGTGCCAAATATGTGATCATCCAAAT

GCTAGTGATGCGGCCTATAGTCGTTATGTTATGCAGTTGCAACATATTGGCCTGCTGAAG

GGAGACGAATTCACTGAACGTTTCTTCCGAATTCTCACGGAACTTGCTGTTGCACACTCT

GTAGTCTCTGAGCAAATTAATGCTCCTGGTGGATTGTCTCAGCAGTCATCACATATATCA

TATTTCCCCATTGATTCATATTCGAAGCTTGTGTCTGTGGTGATAAAGGTATCTATAAAA

TTATTTTGTACACA

>Traes_1DS_35365657E

TGGCTAGTTCGTCTAGAAATGAAAGTCACGAAAACAAAAGGTAAACAGGGGCACGCACCA

ACCCCTCCACATGTTTTGTTCATTGTGGTATTATTTCGAGCTTTCTCTTCGTTTGAAGCA

TTGTGTTGTGTGCATTTGGGAGCTCGCTAAATAACACTGTATATAAACAACACACACAGA

CACAGACTGAGAGACAGACAAGAGTCGCACATCAGAGAGCACGGAGGGAGAAGACCGACA

GCCAGCGGCAGTGGCTAGGTAGACAAGGCCAGCCATGGCGAGCGGGCCGAACGGGGGAGG

CAATGGAGGGGGCGCCGGCGGCGGGCTGATGAAGAACATGAGCCTGCTGCGGCTGCAGTA

CTACTGCGTGCTGGGCGCGGTGGCCGGGGCCGTCCTGTTCGCGACGCTCCGCTACATGCC

GGCCGCCAGCAGCGGCGCGGCCCTCTCCACCACCTCCGCGCTCGCCACCGCCGCGGCGCC

CGCCGGGGCCGGGTCCGGGGCGGGCGAGCACCGGAAGATCAAGGGGACGGCGCCGGCGCC

CGCGAAGGCGGCGGCCAAGGTGGTGACGAAGCCCAAGGAGGTGGTGGTGTTCAACTTCGG

CGACTCCAACTCGGACACGGGCGGGGTGGCGGCGATAATGGGGATCCGCATCGCGGCGCC

GGAGGGGCGCGCCTTCTTCCACCACCCCACCGGGCGGCTCTCCGACGGCCGCGTCGTCCT

CGACTTCATCTGTGAGTGCCTCTTCCCCCCGCGTTCCCCTCCCCTCTCCCCTCGTCACGA

ATGTCTTGTCGGAATGCGTAATCTATCTCCAATATGCTTAGCCGCATTGGGAATCGATTG

TGGGGAGGGAGAGGGCGCGTTTGAACTGAAGATTACTAGTAGTAACAAGGTTGGTGGAAG

AATTGAAAATCATGGTTAGTCGCTGGCTTTTGAATTGAAAATCATTTCCCTTTGATTAAT

ATTTTGGTGAGAGACTACTACAAGATTGATTTTTCTTGACCGTCGTTCGAGCTCATAACC

ATGGCGTGGCCTGCCATCAGGTGAAAAAACACGAACGCAGTCCAATGTGTACGTTGTTTT

CGCCTTCCCCACCATACCGTGTGAAAGTTTTGCCGGCCAAATCGTGACCTCCATCCATCT

ACTCCTACCCAGCTGGCATGCCATCGCATTTCTTCCACGCCACCGAATCAACGTGGTCTG

GCGGATTCCGTTGGTTTTTGACGCGTTCGGCTTGACGCCAAAAACTTTGGCGAGACGCGT

CTCTCCGCCGCTCTTGTCCCGCGTTTCCATGAGAAACCAGGCTCATGGGCCATGGCCATC

GCGGGGAGGTAGATCCACCCCCGAATTGATGGCCAACCAACGTGTGATCGCAGCTGACGC

CAAAGTCCCATAGTACGTCTGTCTGTCTGTCTGTCCGTCCGTTCGCTCTCTCATGATGCG

CACTGACCGACCTCTCTGGCCGTGGCCGGAAAGAAAGAAAGAAAAGCGAGCCGCTTGTGT

GGGAACACGGAATCTCATGCCAGGTTTTCAAGCATCCCCGTGCGGCCGTTGTCAAATTGT

CAAATGCCAACCACCCCGCACTAATTTAAATCAACTTACTCTACATAGATTTAGCTTTCG

TTGAAAGAAAGGAAAATAAAAGAAATATATATTGTAGGAGTAGATCAGTTAAGCAGAATG

GTGGCATGCATATCCATCCAGCCAGCCAGTGGTTGGTGAAGGCGCCGCGCTGGCCGCTGG

ATCCGATCGCACGACGCCGACAGCTCCTCTGCCTTCATCTCGTCACACACACACACAGGG

CGCGCACCTGAGCTTAATTATCGTAGGCCCTAAGAGCAACTCTAACGAGCCGACCTACAC

GGACATCGATTTTGTCTGCATTTTATCTGTTTGGGTCGCCCGTCCGCCTGTCCGTGTCCG

CTTTTTCGAATGGGTCGGCCTGTGCGCGGCGGCATTCTGTCGAACACTTGGTGGGCGAGC

TCCGCACCGCCGCCCCAAGCTCCCACGCCGCCTCGC

>Traes_7BL_CE7BEC05F

CCGGCGTCTGCCCGACCATCGGCGTCGGCGGCAACTTCGCAGGCGGCGGCTTTGGCATGC

TGCTGCGGAAGTACGGCATCGCCGCCGAGAACGTCATCGACGTCAAGGTGGTCGACCCCA

ACGGCAAGCTTCTCGACAAGAGCTCCATGAGCCCGGACCACTTCTGGGCCGTCAGGGGCG

GCGGCGGAGAGAGCTTTGGCATCGTCGTGTCGTGGCAAGGGAAGCTCCTGCCGGGGCCNG

TGGCAAGTGAAGCTCCTGCCGGTGCCTCCCACCGTGACCGTGTTCAAGATCCCCAAGACA

GTGCAAGAAGGCGCCGTAGACCTCGTCAACAAGTGGCAACTGGTCGGGCCGGCCCTTCCC

GGCGACCTCATGATCCGCGTCATCGCTGCGGGGAACACCGCGACATTCGAGGGCATGTAC

CTGGGCACCTGCCAAACCCTGACGCCGTTGATGAGCAGCCAATTCCCCGAGCTTGGCATG

AACCCCTATCACTGCAACGAGATGCCCTGGATCAAGTCCATCCCCTTCATCCACCTCGGC

AAAGAGGCCAGCCTGGTCGACCTCCTCAACCGGAACAACACCTTCAAGCCCTTCGCCGAA

TACAAGTCGGACTACGTGTACCAGCCCTTCCCCAAGCCCGTGTGGGAGCAGATCTTCGGC

TGGCTCACGAAGCCCGGTGGGGGGATGATGATCATGGACCCATACGGCGCCACCATCAGC

GCCACCCCCGAAGCGGCGACGCCGTTCCCTCACCGCCAGGGCGTTCTCTTCAACATCCAG

TACGTCAACTACTGGTTCGCCGAGGCAGCCGCCGCCGCGCCGCTGCAGTGGAGCAAGGAC

ATGTACAATTTCATGGAGCCGTACGTGAGCAAGAACCCCAGGCAGGCGTACGCCAACTAC

AGGGACATTGACCTCGGCAGGAACGAGGTGGTGAACGACATCTCAACCTATAGCAGCGGC

AAGGTTTGGGGCGAGAAGTACTTCAAGGGCAACTTCCAAAGGCTCGCTATTACCAAGGGC

AAGGTGGATCCTCAGGACTACTTCAGGAACGAGCAGAGCATCCCGCCGCTGCTCGAGAAG

TACTGATCGAGGACCTTGCATGGAGATTTAGTGCGTGGTTGCCGTTTCACATGCCCAACT

AGTAGAATAAGGATCGTGCATAGCCGTTTTTCTGTTACTTCTCTTTTTTTTAAGGGGGAA

TCTGCCACTTTATTTATGAATTAAAACAGGTCTGGGA

>Traes_2DL_4D00BC16D

CACCTACCTACTACTTGCGCTGCCTATTTAATCCCTTCCCCTCCCTCCATTCCCCTCCAA

GAAGAGCCACAGCTTCATCTGCAGCTACAGCTCCTCTTCGTCTTCGACACACAAGTATTT

TTTCAGGACAAAGATCAATCCAGATACACATACACCTGCCGACGTTTAACTAGCTTACTT

CCAGAATCCAGATACACATACACCTGCCGGCGTCCAGCTAGCTCACTTGCGAGCTGTAGT

ACGTCGCTGCATATTCCAATGGAGTGCGAGAATGGGCAAGTCGCCGCCAACGGCGATGGC

TTGTGCGTGGCGCAGCCGGCGCGGGCCGACCCACTGAACTGGGGGAAGGCTGCGGAGGAG

CTCTCGGGGAGCCATTTGGATGCGGTGAAGCGGATGGTGGAGGAGTACCGACGGCCCGTG

GTGACCATGGAGGGCGCCAGCCTGACCATCGCCATGGTCGCCGCGGTGGCTGCCGGCAAC

GACACCAGGGTGGAGCTGGACGAGTCCGCCCGCGGCCGCGTCAAGGAGAGCAGCGACTGG

GTCATGAACAGCATGATGAACGGCACCGACAG

>Traes_4AL_B61DB56E6

AGCTTAGCAGCAACCACCAGTGCCTTACACACTCTCGATCAACAAACTCTAGCTGATTAG

TTGCATAGCAAGCAATGGGGTGCTCTAAAACCCTAGTAGCTGGCCTGTTCGCCATGCTGT

TCCTAGCTCCGGCCGTCCTGGCCACCGACCCTGATCCTCTCCAGGATTTCTGTGTCGCTG

ACCTCGACGGCAAGGCGGTCTCGGTGAATGGGCACACGTGCAAGCCCATGTCGGAGGCCG

GCGACGACTTCCTCTTCTCGTCCAAGTTGGCCAAGGCCGGCAACACGTCCACCCCAAACG

GCTCGGTTGTGACGGAGCTCGATATGTGGCCGAGTGGCCCGGTACGAACACGCTGGGCGT

GTCCATGAACCGGGTGGACTTCGCGCCGGGGGTACAAACCCGCCGCACATCCACCCCCGC

GCCACCGAGATCGGCATCGTGATGAAAGGTGAGCTCCTCGTTGGAATCTTCGGCAGCCTT

GACTCCGGGAACAAGCTCTACTCGAGGGTGGTGCGCGCCGGAGAGACGTTCCTCATCCCG

CGGGGCCTCATGCACTTCCAGTTCAACGTCGGTAAGACCGAGGCCTCCATGGTCGTCTCC

TTCAACAGCCAGAACCCCGGCATCGTCTTCGTGCCACTCACGCTCTTCGGCTCCAACCCG

CCCATCCCGACGCCGGTGCTCACCAAGGCGCTCCGGGTAGAGGCCGGGGTCGTGGAACTT

CTCAAGTCCAAGTTTGCCGCTGGGTTTTAATTCCTGGGAGACTGCCCATAAATGATCATA

AATCATTCGGTATATGCATGCTAGCGACATTTAATAATTCTCAGCAGAAGATATCTATTC

ATGCTTCTGGTTAAGCATGCATGCAGTTGTAATAAGATTGAATAAGTTAGCCTCGTGGTT

CAGTCATCAGAGCCAATATGAGGAAT

>Traes_4DS_3D70C7840

ATTAGCTCCATGCATCCATTGCGATCCCCTATATAAAGGACTCCATATGCCTCACCATTC

ACTCATCCCGTCATCCACCACAGCTTAGCAGCAGCAACAACCAGTGTCTAAGACACTCTC

CATCAACAAACTCTAGCTGATCACTCCTAGCTAAGCTTTGCATAGCAAGCAATGGGGTAC

TCCAAAACCCTAGCGGCTGGCCTGTTTGCCACGCTGCTCCTAGCTCCAGTCGTCCTGGCT

ACCGACCCTGACCCTCTCCAGGACTTCTGTGTCGCCGACCTCGACGGCAAGGCGGTCTCG

GTGAACGGGCATCCGTGCAAGCCCATGTCGAAGGCCGGCGATGACTTCCTCTTCTCGTCC

AAGCTGGCCAAGGCTGGCAACACGTCGACCCCGAACGGCTCGGCCGTGACGGAGCTTGAC

GTGGCTGAGTGGCCTGGAACGAACACGTTGGGCGTGTCGATGAACCGCGTGGACTTCGCT

CCAGGAGGCACCAACCCGCCGCACATCCACCCCCGTGCCACCGAGATCGGCATCGTGATG

AAAGGTGAGCTCCTCGTGGGAATCCTCGGCAGCCTCGACTCCGGGAACAAGCTTTACTCG

AGGGTGGTGCGCGCTGGAGAGACGTTCCTCATCCCACGGGGCCTCATGCACTTGCAGTTC

AACGTCGGTAAGACCGAGGCATCCATGGTAGTCTCCTTCAACAGCCAGAACCCCGGTATC

GTCTTCGTGCCATTGACACTCTTCAGCTCCAACCCACCCATCCCGACGCCGGTCCTCACC

AAGGCGCTTCGGGTGGAGGCCGGGGTCGTGGAACTTCTCAAGTCCAAGTTCGCCGATGGG

TTTTAATTCCTGGGAGCCTTCCCTGAAATGAACAATATGTAATTCAATATATGCATGCTA

GAAAATTTTAATAATTCTCAGCAGAAGACTTGTATTCAAGTTTCAGGTTAATCTCGCATG

CAGTTGTAATAAGATTGGAGAAGTTAGCCTCCTGGTTTAGCCTTCACAACCAATATGAGG

AATTGAATGTACTACTTTTATTGCCGTCTTTGTTCTTCTCATTGCATGGAGTATATAATT

AGCATTTTTTTTGTATATTT

>Traes_1AL_CFF403F3E

CGTAGCATTGCATACTCCAAGCTGCTGATGTTTAAACGCGTGGTTATGGGCAGCTTAGTT

GCCTCCTGCCGTGGCGGGGGCTGCAGCCTGCGCCTGTCTCATCTTGGCGAGTTCGACCAC

TCTGTCGACGGCCGGCAGGACAGCCTTGGCGGCGTCCAGCGCGCCAAAGCGCTCCACCCA

CGCCGCCAGGAGAGGGCCGAAGAAAGGCTTCACCCAAGCCTCCTGCAGTACATCGACGAA

GCATTCGCCGACATCGGCCGCTCCCTCCTCCCCTCCGACCCCCACCAGCGCGCGGTCGCT

CGCTTCTGGGCCTCCTACATCGATGACAAGCTCGTGATCCCATGGGTGCGGTCGTTCAGG

GGCAAGACGGAGGAGGAGAAGTCTGAGTGGATGGAGCAGACGCTCATCGCCGTGGAGACC

CTGGAAGGAGCCCTGAGGGAGTGCTCCAAGGGCAAGGGCTTCTTCGGCGGCGACAATGTC

GGGCTCGTCGACGTCGTGCTGGGCAGCCTGCTCACGTGGGTGCACGCGGCTGAGGTGATG

TCCGGGGCCAAGATGTTTGACCCTGCTAAGACCCCATTGCTGGCCGCGTGGATGCAGCGC

TTCGACGAGCTTGCCGCTGCCAAGGCCGTCATGCCGGACGTTAATAGGATGGTCGAGTTC

AAGACGAGGCAGGCACAGGCCATCTCTGTCGCTGCAGCTTCACAGCGTCAGTAA

>Traes_1AL_CC4CF4E71

ATGGCCGGAGGAGATGACTTGAAGCTGCTCGGCGCTTGGGCGAGTCCATTTGTCGCCAGG

GTGAAGCTTGCGCTGAGCTTCAAGGGCCTGAGCTTCGAGGATGTCGAGGAGGACCTCAGC

AACAAGAGCGAGCTCCTCCTCAGCTCGAACCCGGTGCACAAGAAGGTGCCCGTGCTCGTC

CACAACGGGAAACCCATTTGCGAGTCAATGATCATCGTTCAGTACATCGATGAGGCGTTC

CTTGTCGGCCCCTCTCTTCTTCCCTCTGACCCCTACAAACGTGCAATTGCCCGTTTTTGG

GCCGCCTACATTGACGATAAGCTCGTCACCCCATGGGTACAGTCGTTGAGGGCCAAGACA

GAGGAGGAGAAGTCTGAGGGGGTTAAGCAGACATTTGCCGCTGTGGAAACACTGGAAGGA

GCCCTGAGGGAGTGCTCCAAGGGAGAGGGCTACTTTGGTGGTGAGACCGTCGGGCTTGTG

GACATTTCACTTGGGAGCCTGCTCTCCTGGTTGATTGCGACAGAAGTGATGTCTGGAACC

AAGATCTTTGATCCTGTTAAGACTCCGCTCCTGGCAGCGTGGATGGGGCGCTTTAGCGAG

CTCGACGCTGCCAAGGCGGCGTTGCCAGAAGTTGATAGGGTGGTCGAATTTGCCAAGAAG

AGACAGGCACAGGCTGATGCCGCCGCCGCTGCTTCGGAGACCAAGGAAAATTAG

>Traes_2AL_25944DD47

CTCCACGCTCGTCAACTCACGTGACATATGGGGTGACGCTACCCTTCCCGCCAGCCTCAC

ATGGCACTGACACTACATAAAGGTACGAGGCTACGAGCTCCTCCACGACAATCCACACCA

ACACCAAACCACGGTGGCCGCCGATCGACCCGGCCACGCCTGAGAGCAGCATCTTGCCGG

AGACATGGCATCCGCTCCTGCGGCGGTTCCCCGCGTGAAGCTGGGCTCCCAGGGAATGGA

GGTCTCGGCGCAGGGCCTCGGCTGCATGGGCATGTCCGCCTTCTACGGCGCGCCCAAGCC

CGAGCCCGACATGGTCGCGCTCATCCGTCACGCCGTCGCCGCCGGAGTCACCTTTCTCGA

CACCTCCGACATGTACGGGCCGCACACCAACGAGATGCTGCTCGGCAAGGCGTTGCAAGG

CGACATGAGGGAGAAGGTGGATCTGGCCACCAAGTTCGGCATCTCGTTCGCAGACAACAA

GTGGAACATCCGGGGGGATCCAGCGTACGTGCGCGCGGCTTGCGAGGGCAGCCTCAAGCG

GCTGGGTGTCGACTGTATTGATCTCTACTACCAGCACCGCATCGACAAAAATGTGCCCAT

CGAGGTCACGATTGGCGAACTCAAGAAGCTAGTCGAAGAAGGAAAGATAAAATACATCGG

ACTATCTGAAGCATCTGCATCAACAATCAGAAGGGCTCATGCTGTCCATCCTATTACTGC

AGTTCAGCTGGAGTGGTCATTATGGTCTAGAGATGTGGAAGAAGACATAATTCCAACTTG

CAGAGAACTTGGAATCGGAATCGTACCTTACAGCCCACTTGGTAGAGGATTTTTCTCTGG

TGGAGCAAAACTGATCGACTCGCTATCAGATCAGGACTACCGCAAGCATATGCCCAGATT

CCAGCCAGAAAATCTTGAGAAGAATACCCTGATATTTGAGCGTGTTAACGCAATGGCAAC

AAGAAAAGGGTGCACACCATCACAACTTGCATTGGCCTGGGTTCATCATCAGGGGAGCGA

TGTTTGTCCCATACCTGGCACAACAAAAATCGAGAATTTCAACCAGAATGTGGGAGCACT

ATTTGTGAGGCTCACACCAGAGGAGATGGCTGAACTCGAGTCCTATGCCACTGCCGGTGA

TGTCCAGGGTGAGCGGTACTCTGAAATGGCGAGTACTTGGAAGTATTCTGAGACCCCTCC

ATTGTCATCCTTGAAAGCTGCGTAGCCATTTTTCTTCAGTGGGACTACAGGAGTTCATGA

TTCCAAACAGGCACAGGAGGGTAATCATAGTTGTGTTTTTGGAGTATTGACCAGAGTTGG

ACTCCCATTGTTGCGAATGCCGGTATGTAAAATAAGCTGTGGTATGTATGTGTGTATGCT

TCGTTGTTGGTATACAACTAGTGTGTTGAATAAGCTTGTGTGCTGTAGTGCTACATAAAG

GTGTGGTAAAAATACATGTTGACAAAAAGAATAATCATGTACAAATTGTCCCTTGGGTTA

TATTTTTGTGAAAGCATATGGACACAGTGCTTGTAGGGGAA

>Traes_5AL_FC06F4F12

GCCCGTCGCCGTCAAGTACCTCGACCTGGACTGCGGCACGCAGGGACACAAGGAGTGGCT

GGCAGAGGTTTTCTTTCTTGGGCAACTGAGGCACAAGAACCTGGTGAAACTGATCGGCTA

CTGCTACGAGGACGAGCACCGGATGCTAGTCTACGAGTTCATGAGCGGCGAGAGCCTGGA

GAAGCACCTCTTCAAGAGCATAAATGGCTCTCTCCCGTGGATGACAAGGATGAAGACCGC

TGTCGGCGCGGCCAAGGGCCTTGCCTTTCTCCACGACGCAGACCTGCCCGTGATCTACCG

CGACTTCAAGGCCTCCAACATATTGCTCGACTCGGATTACAACACCAAGTTGTCCGACTT

TGGGCTGGCCCAAGATGGGCCCCAGGGCGATGAGACACATGTGACAACACGTGTCCTGGG

GACTCATGGTTATGCAGCGCCAGAATACATTATGACGGGCCACTTGACCGTCAAGAGTGA

TGTATATAGCTTTGGCGTAGTGCTCCTAGAGCTTCTCTCCGGGCTACGGTCAGTGGATCG

TTCACGGCGGCTAAGGGAGCAGAACCTGGTGGATTGGGCTAGACCATACCTCAAGCACTC

TGACAGATTGTACAAAGTCATGGACCCAGCTCTCGAGTGCCAATACTCATGCAAAGGTGC

CGAGGTGGCAGCACTGGTGGCATACAAGTGTCTTAGCCAGAACCCAAAGTCTAGGCCCAC

CATGAGGGAGGTGGTCAAGGCCCTCGATCCCATCCTCAGCATGCAAGACTTCTTTCCTGT

GGGCCCATTTGTGTTCACAGTCATTGTGGAGGAGGACAAGGTCGTGGACATGAAGGTGGA

GGTCGAGGAGAAGCACCAACACCATTGCCAGAACCATCAAGACAGGCACCGGCAGAAGTA

CCCTGACTCAGCAATCCATGCCGGCATTGTGCTCCGCGGCCACGATGGGTTCATTACCGG

GTACACTGGTGCGCAGCGGCGGCAACAAAGGTCATCGAGCTACCACCGGGAGGGAGGGGT

ACAAGACTAAGGTAGGGAAGTGTGTAGGACAATATGGTGTGCACATAGTATAGAGATGTG

ATACAATGTCACATCAGTTTGTTTCAGATTTGTGGATTGTGTTTCTTTTCTTCTTTTGTA

TTTTTGGAAAGGTCTCATGTGGCTTACGCCCATCTTTCTTCTTGATAGTGACAACAGGGG

TTAGATAAATATAGGGATGCAAAACGGTGTGACAAAAGTCTGTTTTTGATTTTCGCAAGG

ATTGTACAAGATGTTGTGAATGGCCTAGTTAAAAG

>Traes_4AL_B528D14EC

GAACTTCTGAAATCCACGAAGCTGGCGTCGACGCAAGCACGTACGCACCGGAGTACATCA

GTGCCGCGCGCCACGTGATGTTGCTTCTAGTCTCCAGTGAACCCACGTACGCGGCAGAGA

GTCAGAGACCCCAGCATGCAGTTCGGCTGAGCCGAAGCCGGACGTACTCCCCGATCTAGA

TATATTCATTTATACAACGAGTAATTTGAAACGGAGGGAGTACCTCCATAGCTCCCCTAT

ATAAACGCACCGGCGCCGAGCTACAAAGTGCAAACACAAATCGCACGGCGCAAACGCAAC

ACACAATCATGGCTGCCACATTGAAGCTCACAGTCGCCTTCCTCCTCCTCGTCTCCGCGC

TGGTGGCGTTCGGCGACGCCAAGAAGGCGCCGTGCGCCGTGGTGTGCGTGCAGGGAGGGC

ACATCACCTGCGACAACTACCCCGGGCAGAAGCTCGACGGGTGCGACTGCCAGTGCGCGC

CCAAGGACGGCAAGGGCTGCGTGCTCCACCTCAACAGCGGCTCCACCAACCAGTGCACCA

CCCCGCCGGAGTACTGAGATGAATTACACGCGGTTCGGCATGCTGGCTCGAGTCCGAGCT

CTATGAAGATGCAAGTGTGTGTGTCGTGGAGCAACTATAGATACACTTCCTATCAAAATG

TAAGACCATTTTTGACATTATGATAGTGTCAAAAAAGCATCTTACATTTTAGTAATACAA

AGGGAGTATCATGTAAGTTTGTGTCGTGGAATAAATAACGAAAGGAATGCAGTCTGTCG

>Traes_2AL_1B97CF2E5

GGAGCTTGGAACGGAGTACTTGACCGGAGAGAAGACGCGGTCTCCTGGCGAAGAGGTGGA

CAAGGTGTTCGTTGCCATGAACCAGGGCAAGCACATCGATGCGCTGCTTGAGTGCCTCAA

GGAGTGGAACGGCGAGCCCCTGCCTCTCTGCTAAATAGAGGATCGAGAAAGTGAAGAGTA

GTGTGCTTCAGATTTCTGAAGGCTCTTATGATAATACTGTTTTTTCATTGTATATTCGAA

AAGTTGATGTTTACAATGTTCTTCTAGAGCTGCCAATGTGTTGCCATGCAATTGCATGAC

TTGGTAGTGTTGGGTAGCCAGTAGAACTTTTATGGTGTACGTAAGTTAAAAAGTTAGTGT

GTGTTAAATTTACATGATAAATCTACTGGCTCCATTTTTTGAGATCAACCAGCCCTCGTG

GCAATAGTCCGAGAACCAACTATTGGAATTTCTTATGGTGTGTGTACGGCGATTCGGAAA

GTAAAAC

>Traes_2AL_9EC3226F7

TTGCATCTTAGATAACTTGGAATGAAGATGAACTCGGACAACGAAGTCAGCAGGGCAGAT

AGCCCTCCGTGCCACCAACCGCCGTCCCCCTCGCGCGACGCGCACCAACCAGTTCCTGCT

TTGCCCACGTCGACTGAGTCCAGCTTCGGCAAAGCCACCACCGCGCGTAGCCACCACCCC

CGCTCCGTGCGCTCCCGTGGCGACAGGAGCTCCGTCCGCACCAACCATTTCAGTGGTCAA

CCTCGCCGTCCAGGTCTACACCAACCCACTCTCTCACACCTACCTACTAGTACACGGCTG

CGCCTGCGCTGTCTATTTAATCCCTCCCCTCCCTCCATTCCCCTCCAAGAAGAGCCACAG

CTTCATCTGCAGCTACAGCTCCTCTTCAGCACAACCCTTTCTTCAGGACACAGATCAATC

CAGATACACATACACCACCTGCCGAGTGCCGACGTCTAGCTGCTAGCTTACTTTCAGAAT

CCAGATACACACACACCTGCCGGCGTCCGTTGCTGCATATTTCGATGGAGTGCGAGAACG

CACACGTTGCCGCCAACGGCGATGGCTTGTGCGTGGCGCAGCCGGCGCGGGCCGACCCAC

TCAACTGGGGGAAGGCGGCGGAGGAGCTCTCGGGTAGCCATTTGGAGGCGGTGAAGCGGA

TGGTGGAGGAGTACCGCAAGCCGGTCGTGACGATGGAGGGCGCCAGCCTGACCATCGCGA

TGGTCGCCGCGGTGGCTGCCGGCAGCGACACCAGAGTGGAGCTGGACGAGTCCGCCCGCG

GCCGCGTCAAGGAGAGCAGCGACTGGGTCATGAACAGCATGATGAACGGCACCGACAGTT

ACGGTGTCACCACCGGCTTCGGCGCCACCTCTCACCGGAGGACCAAGGAGGGCGGCGCTC

TCCAGAGAGAGCTCATCCGATTCCTTAACGCGGGAGCCTTCGGCACCGGCACCGACGGCC

ACGTTCTGCCTGCCGCAGCGACAAGGGCGGCGATGCTCGTCCGAGTCAATACCTTGCTCC

AGGGATACTCAGGGATCCGCTTCGAGATCCTCGAGACGATCGCCACGCTTCTCAACGCCA

ACGTGACACCTTGCCTGCCGCTCCGGGGCACGATCACCGCGTCGGGTGACCTCGTCCCGC

TTTCCTACATCGCCGGCCTGGTCACCGGCCGCCCAAACTCCATGGCGACGGCTCCGGATG

GTTCGAAGGTTAATGCTGCGGAGGCATTTAAGATCGCCGGCATCCAGCACGGCTTCTTCG

AGCTACAGCCCAAGGAAGGCCTTGCCATGGTGAATGGCACGGCAGTGGGCTCAGGCCTTG

CCTCCATGGTGCTTTTCGAGGCAAACGTCCTTAGCCTCCTTGCTGAGGTCTTGTCGGGCG

TCTTCTGTGAGGTCATGAACGGCAAGCCGGAGTTCACCGACCACTTGACCCATAAGTTGA

AGCACCACCCCGGGCAAATTGAGGCCGCCGCCATCATGGAGCACATCCTTGAAGGCAGCT

CCTACATGATGCTCGCAAAGAAGCTCGGTGAGCTTGACCCACTGATGAAGCCAAAGCAAG

ATAGGTATGCACTCCGCACATCGCCGCAGTGGCTTGGCCCTCAGATTGAGGTCATCCGTG

CTGCCACCAAGTCAATCGAGCGTGAGATCAATTCCGTCAACGACAACCCACTCATCGATG

TCTCCCGCGGCAAAGCTATCCATGGTGGCAACTTCCAGGGCACGCCCATCGGTGTGTCCA

TGGACAACACCAGGCTTGCCATTGCAGCGATCGGCAAGCTCATGTTTGCCCAGTTCTCGG

AGCTGGTGAACGACTTCTACAACAACGGTCTGCCTTCCAACCTCTCCGGCGGGCGCAACC

CAAGCTTGGACTATGGCTTCAAGGGTGCCGAGATTGCCATGGCCTCGTACTGCTCCGAGC

TCCAATTCTTAGGCAACCCTGTGACCAACCATGTTCAGAGCGCGGAGCAACACAACCAAG

ATGTCAACTCTCTTGGTCTCATCTCCTCAAGGAAGACTGCAGAGGCCATTGACATATTGA

AGCTCATGTCCTCAACATTCTTGGTCGCGTTGTGCCAGGCTATCGACCTCCGCCACCTTG

AGGAGAATGTCAAGAATGCTGTCAAGAGCTGCGTGAAGACAGTGGCTAGGAAGACACTGA

GCACTGATAACAATGGCCATCTCCACAACGCACGCTTCTGCGAGAAGGACCTTCTGCTCA

CAATCGACCGTGAGGCCGTGTTCGCGTACGCAGATGACCCCTGCAGCGCCAACTACCCCC

TCATGCAGAAGATGCGTGCAGTTCTCGTGGAGCACGCCTTGGCCAATGGTGAGGCCGAGC

GCGACGTCGAGACGTCGGTGTTTGCCAAGCTTGCCATGTTCGAGCAGGAGCTCCGTGCAG

TGTTGCCAAAGGAGGTCGAGGCCGCCCGAAGCGCCGTGGAGAATGGCACCGCAGCACAGC

AAAACCGTATCGCCGAATGTCGGTCGTACCCGCTCTACCGGTTCGTGCGCAAGGAGCTTG

GAACGGAGTACTTGACCGGGGAGAAGACGCGGTCTCCTGGCGAAGAGGTGGACAAGGTGT

TCGTTGCCATGAACCAAGGCAAGCACATCGACGCGCTGCTGGAGTGCCTCAAGGAGTGGA

ACGGCGAGCCCCTGCCTCTCTGCTGAACAGAGCATCAAGAAATGGAAGAGTAGCGTGCTT

CAGATTTCAGAAGCCTGCAGTGCTGATACTGTTTTTTCATTGTAATTTCTAAAAGTTGAT

GTTTGCAACGTTCTTCCAGAGCTTCCAATGCATTGCCAAAGATTGCAATTGCATGACATG

GCAGTGTTGGGTAGACAGTAAAACTTCTTTGATGTAAGTTATAAAAGGGTACAGTGTGTG

ATAAGTTTCCATGATAAATATACCGGTTCAAGAAAGTAAAACTCATGCCTTCCAGAGCTG

CCAGTGGTAACTCCGTCCTTGCTAGTTCCATCAACACTGTACGAG

>Traes_4DS_8721A5B16

GCTTAATTAGCTCCATGCATCCATTGCCGCGCCGATCCCCTATATAAACCACTCCATATG

CCTCACCACTCACTCATCCACCACAGCTTAGCATTAACCACCAGTGCCTTACACACTCTC

GATCAACAAACTCTAGCCGATCAGTTGCATAGCAAGCAATGGGGTGCTCTAAAACCCTAG

TAGCTGGTCTCTTGGCCATGCTCTTCCTAGCTCCGACCATCCTAGCCACCGACCCTGATC

CTCTCCAGGACTTCTGCGTCGCCGACCTCGACGGCAAGGCAATCTCGGTGAACGGGCACA

CATGCAAGCCCATGTCGGAGGCCGGCGACGACTTCCTCTTCTCATCCAAGCTGGCCAAGG

CTGGCAACACGTCCACTCCTAACGGCTCGGCCGTGACGGAGCTCGATGTGGCAGAGTGGC

CCGGCACAAACACGCTGGGCGTGTCCATGAACCGCGTGGACTTTGCGCCCGGAGGCACCA

ACCCGCCACACATCCACCCGCGTGCCACCGAGATCGGCATCGTGATGAAAGGTGAGCTCC

TCGTGGGAATCCTCAGCAGCCTTGACTCTGGGAACAAGCTCTACTCCAGGGTGGTGCGCG

CCGGAGAGACGTTCCTCATCCCGCGGGGCCTCATGCACTTCCAGTTCAATGTCGGGAAGA

CCGAGGCCTCCATGGTCGTCTCCTTCAACAGCCAGAACCCCGGCATCGTCTTCGTGCCAC

TCACGCTCTTCGGCTCCAACCCACCCATCCCGACACCGGTGCTCACCAAGGCGCTCCGGG

TAGAGGCCGGGGTCGTGGAACTTC

>Traes_4BL_9C8AF6380

CAGGGTTCTGCAGCTCTCCTGGTCGATCCCGCAGACCCGCACCACCAACCCGTTCATCCC

CGGCGGCGCCCCTTCGCTCCCCAGCACATTTCCGCGAGCAGCTGGTGCGCGCGCGGAGCG

GAGGCCATGGCTATGTCAAAGGCCAAATCCACCGCCCTCTCCGCCGCCGAAAAGTGCCGG

AACATCCTGGGCGCCAGTTGGGAAGCCCACCTCAACACCATCAAAGCGGATGCCAAGGGA

AGCAAGGGGGAGGTTTACACGTCGAGGGTGCACTACATGGTCCAAAAGGGCCTGCCCTAC

CTCATCGTCCCCGAGAACGACATGCACAACATTAACATCATAATCGACGAGCGGGGTTCT

CTCTCGGTGTCCAGTCCAGTCCCAGGTCGCCTTGCCAGCTTGCTCAAGTCACCCAACAAG

TTGCCTCCCCGGGTTGCTATGACCGGCGATGTCCTGCGCATGAAGGAGACAAAGGTTCCA

GTTATAGCTGAAAGCCTTAAGAAAGCTATTCTGAAGGAACATAAAGCTGCTAGCGAAGCT

ACTTATGGGGTTTCAACGGTATTGTCTTCTGCAAGTGCTACTTGTAGGTCGCGCAGTGAA

GGTCTCCTTAGCTTACTCAATGAAGAGAGCTCCTACAATATCTTGAAGTTTGAAATTGGC

TCATGTGTCTTCATAGATTCATTGGGGAGCAGCCATAATATCGAGTTGGATACTTTTGAA

CCACCAAAAGCCGACCTGCTATTGCCATTCTCTGCGAAGCTTATTGATGGTATCAACAGG

AGTGACCCAAGGCGGAGAGCGCTTATACTTTTCTGTTTCGAGTATTTTGATGTGACTGCC

AGAGATGCAGTCTTGCTCTCCGTTGATCACCATGGATTTGAGGTGCTCGCCAGGGTTCCT

GAAAGAGCTACCGCACCTGATGTCCCTCAGCAGTACCACTGGAAAGAGTTCAGGTTCACA

TTCAAGGAAGCAGTCAAGGATATTGAAGACTTCTGCCGCATGTTGGTCGAGCTAGAAGAA

GAAGCTCTGCACAGCGTAAAGAGCTATAGCGGATTAGGTTGAAGGGGATAGTAGCTCTTT

CTCTCAAACCAAAGGGCACAAGATTCAGATCTTCAGTTATGATAATAAATCATCTGAGAA

TATAACCTCCGTTTTTGTAGATGGTTTGCATTGTAAACTGACATCATTCGATTGAGAATC

AACAGTTAGGTTTGTATGTCACCATAATTTTCGGCCTCTGTTTGGTAGTACTTATTTACA

AATCAGAAAGGAAACTTCTCTGCATATTACTCCCTTCGTCCCAAATAAG

>Traes_4BL_4D370C9FA

TTCTCGGACGCGTCGAACGCAGTCGTCACCGTCCCCGCCTACTTCAACGACTCCCAGCGC

CAGGCCACCAAGGATGCAGGCGTCATCTCTGGCCTCAACGTCATGCGGATCATCAACGAG

CCTACCGCCGCCGCCATCGCCTNCAAGAAGTCCACCAGCGTCGGCGAGAAGAACGTGCTC

ATCTTTGATCTCGGCGGCGGTACCTTCGATGTCTCGCTCCTCACCATTGAGGAGGGTATC

TTCGAGGTCAAGGCCACTGCCGGAGACACCCACCTGGGAGGCGAGGACTTCGACAACCGG

ATGGTCAACCACTTCGTGCAGGAATTCAAGAGAAAGAACAAGAAGGACATCAGCGGCAAC

CCGAGGGCGCTCCGGCGGCTCAGGACAGCGTGCGAGAGGGCGAAGAGGACACTCTCCTCC

ACCGCCCAGACCACCATCGAGATCGATTCCCTCTTCGAGGGCATCGACTTCTACACTACC

ATCACCCGCGCCCGGTTCGAGGAGCTCAACATGGACCTCTTCCGCAAGTGCATGGAGCCC

GTTGAGAAGTGCCTCCGGGACGCCAAGATGGACAAGAGCACCGTGCACGATGTTGTCCTC

GTCGGAGGATCCACACGTATCCCCCGTGTGCAGCAGCTCCTCCAGGACTTCTTTAACGGG

AAGGAGCTCTGCAAGAGCATCAACCCTGACGAGGCCGTCGCGTATGGAGCCGCCGTTCAG

GCTGCCATCCTCACTGGCGAGGGCAACGAGAAGGTGCAGGACTTGCTCCTGCTTGACGTC

ACACCGCTCTCGCAGGGCCTGGAGACGGCCGGAGGTGTCATGACCGTGCTGATCCCAAGG

AACACGACAATCCCCACCAAGAAGGAGCAGGTCTTCTCCACCTACTCTGACAACCAGCCC

GGCGTGCTCATCCAGGTGTATGAGGGCGAGAGGGCGAGGACCAAGGACAACAACCTGCTC

GGCAAGTTCGAGCTCTCCGGGATCCCACCGGCGCCCAGGGGTGTCCCCCAGATCACCGTG

TGCTTTGACATTGATGCCAACGGTATCCTGAATGTCTCGGCGGAGGACAAGACCACCGGA

CAGAAGAACAAGATCACAATTACCAACGACAAGGGGCGGCTGAGCAAGGAGGACATCGAG

AAGATGGTGCAGGAGGCCGAGAAGTACAAGGCTGAGGACGAGGAGCACAAGAAGAAGGTG

GACTCCAAGAACGCCCTGGAGAACTACGCTTACAACATGCGTAACACCATCAAGGATGAC

AAGATCGCCTCCAAGCTCCCAGAGGCCGACAAGAAGAAGATCGAGGATGCTATCGATGGT

GCCATCACCTGGCTCGACAACAACCAGCTCGCCGAGGCTGAAGAGTTTGATGACAAGAGG

AAAGAGCTGGAGGGCATTTGCAACCCCATCATCGCCAAGATGTACCAGGGTGCCGGCGCT

GAAATGCCTGGTGGCATGGATGAGGATGCTCCGGCCAGTGCCGCAGGCGGCAGCAGCGGC

CCAGGGCCCAAGATTGAGGAGGTCGACTAA

>Traes_6DS_6E865520A

GTCTTACAGGACAAATCCCGTTAACCTTGACGGAGATGCCAATGTTAAAATCAATTGAAA

ACGCAACTCATTGGGACCCAAGGGTCTTTGAGTTGCCTGTTTACAATGGTCCATCACTTC

AATACCGTGTTGTTACTTCTTTTCCAGCAGTGTTGAATCTAAGCAACAACCACTTCACAG

GTGTGATTCCCCCACAGATTGGTCAGTTGAAAGTGCTTGTTGCACTTGATTTCAGTTTCA

ACATGTTATCCGGACAGATCCCACTGTCGATTTGCAACCTCACAAACTTGCAGGTGCTAG

ACTTGTCCAGCAACAGTCTAACAGGTGCTATCCCAGCTGCATTGAATAGCCTACACTTCC

TTTCAGCATTCAGCATTTCGAACAATGACCTAGAAGGGCCTATTCCATCTGGAGGCCAGT

TCAATACATTTCAGAATTCTAGTTTCGATGGGAATCCAAAGCTGTGTGGCTCTATTCTCA

CTCACAAATGTGATTCTTCAATACACCCGTCCTCCAGAAAACAACGAGATAAGAAATTTG

CTTTCGCAATTGGATTTGGTGTGTTCTTTGGAGGTATTGCTATTCTGTTGTTGCTGGTGC

GACTCCTTCTCTCAGTCAAGATGAAGGGTTTTACAGAGAAACATGGAAGCAAGAATAGCA

GAGATGTTGAAGCGACTTCACTCTACTCTAGTTCAGAGCAAACACTAGTAGTGATGCACA

TGCCACAAGGCAAGGGAGAAGGAAACAAGCTCAAATTCTCTGACATTTTGAAAGCTACGA

ACAACTTTGACAAGGAGAACATCATTGGGTGTGGAGGCTATGGGTTAGTCTACAAGGCAG

AGCTACATGATGGCTCCAAATTGGCAATTAAAAAGCTCAACGGTGAAATGTGTCTGATGG

AAAGGGAATTCAGTGCAGAGGTTGATGCTCTCTCCATGGCACAGCATGAAAATCTTGTAC

CACTCTGGGGTTACTGCGTCCAGGGAAACTCAAGGCTCCTCATGTATTCCTACATGCAGA

ATGGCAGCCTGGATGATTGGCTTCATAACAGAGATGATGACACTAGCTCATTTCTTGACT

GGCCGACTCGGCTCAAGATCGCACGAGGAGCCAGCCTAGGCCTCTCCTATATACATGATA

TCTGCAAGCCTCAAATTGTACACCGTGACATCAAATCCAGTAATATCCTATTGGACAAAG

AATTTAAAGCTTACCTTGCAGATTTTGGGCTAGCCAGGTTGATCCTTCCCAACAAAACTC

ATGTTACAACTGAGTTGGTCGGCACTATGGGTTACATTCCCCCTGAGTATGGGCAAGCGT

GGGTTGCTACGTTGAGAGGAGATATATACAGTTTTGGGGTAGTCCTGCTTGAGCTGCTCA

CAGGAAGGCGACCCGTTCCAGTTTCGTCTACAACAAAAGAACTTGTCCCATGGGTTCTAC

AGATGAGGTCTGAGGGCAAGCAGATTGAGGTCCTGGATCCAACACTTCGAGAAACAGGGT

ATGAAGAGCAAATGCTGAAGGTGCTTGAAACTGCTTGCAGGTGTGTCAATCATAATCAGT

TCAGGAGGCCAACTATCATGGAAGTGGTCTCCTGCTTGGGCAGTATAGACACTGACCTGC

GGACATAAAAGCTAGCCAATACTTATTATTAACCTTACAGTTGGGTTTTACAGCGCCCAA

CAGGTTGTTATTTTGAGCTTAATTTACTTTTCTAGAAAAGCTTCTCTTTTTGTAGTTCAT

CTTCCTTTTGGATGTTTCTTGAAGCATTATTCATGCTCAAGCTTCCCAAGAGTGTAAAAA

AAGAATAAACAAGTAAAATGATTGCCTTGTTTTCACAGTGAAACAATAAAAGTGCTTTGC

ATTCAAGTGCAGATACTATACTATGCTCTATCAGCTGTTTGCTTCGGATTAACTGATTCA

CAAAACACCAGAGTTTGAGTTGGATTGGTTGATACTGGAGTGTACAGACGTTTGGTCCAC

GCGTGAATTGAATGGATATAACAACACAACACAATACCACAACTAAATGAACAGAAG

>Traes_5BL_292516643

TATCACTTTGCACCTTGGTTGGTAGCTTGCATCTCATATCGATCAGAAGTTTTTCAGCTA

GGTGCAACCTACCAAACAATATCTGCTAAATACATCATCTCTCATACACCCTTAGATAGA

AACAACCAAACAACATCTCTGCTCAGCCTGCCCCAAAGCATATAGGCCACCAAACACACA

TCTGAGCTTATCTTTGCATCAACTCAGCCAGGATCCCAGATACGATACAGGCTGGAGTAA

TGTGAACAATCAGACACACCCTTATTTTCTGTTCCGCGGTGCCGGAATATAGCATAGAGC

TGTTTAGACATTTGGATACTACTGTAGCCACTGAAACTTACAAAAGGTGCAGAAATTGCT

GTCTATGACCGGTCCTATTTGTCGCTGATGCAGTTTTGCTGCTTGATTCTGGAGTTCGTA

CGTCTTTTCTAGTGCCAAAAAATGCGTTATCTGCTTGTTTTGTCCAGTGTCGCAATGTAA

ACTATTTCTAAGTTGTCTGGTTGTTGCGTTGATGCACATAATTGTTGAGGAGAGTCATTT

GCGGCATTTGGCTAAAATTGTATGGCGAGTTTTAGCGGTGTCATACTCGCTTTGTGTTTC

ATATATGCATATTGATATGTATTTCACCATGCTTTTAGTAATAAGCTAGCTTGATTTTTC

AACTTTGTAGGCATCATACATATATTGATTTGTTTGTGGTCATTAGTCAATAGTCATACA

GGGCTCCATATTTGTAAGGCCTGCCGCTTATTTCCTTCTTTACCAATTCTAAGAAGATAA

ACTCAAACATCTGTTAAACTTCACCTAAACTTCATGTTATTTTCCTGCAAATTACTTCAA

TCATAACCTCTACTCAGTTTGCTCTATTGTTTTTAAATGTTTCTGCAGAAGAATTTGGTA

TCTAAGTTTCCTCAAGAGACTAGATTGCTTACGATGATGGCTGAGGAAACCAAATCAAAG

AAGCAAAAAGACGAGGAACACATTATAAACGGTCTCCCAGGAGAACTCATTGAGCGGATA

TTTTTTAAGCTTCCAGTGAGCACTTTGTTGAGGTGCACTGGTGTCTGCAAGGAATGGCAC

AAAATCATCCGAGATCCCCAGTTTGTCACCTCTCACCTCCAGGATGCGCCCCAATGTGCT

TTCCTATTCTTCCCACAAGAGTCGATCTCAGGAAAGCTCTATCCTGCTGATGCTATCCTG

ATTGATGAGACCTGGTCACAGTCGACATGTGCAGTGCCAGTGGTTGGCCCTGGTGATTCC

CTTTGTGGTTCATGCAACGGGCTTCTTTGCTTATACACAAAGTCATCAACACTCAAGATA

GCTAACCTTGCAACGGGTGAATGCCTGCATCTTGATAAACCTGTAAAGAATTTAAGGGGT

GATTACTTCTTCTTGTATAACTTTGGATTTCACCCAGTGACAAAAGAATACAAGATTATA

CACTTCCTTGGTGATTCTGTCGAGGGCCGCCCTCTCCATAATAATAGCAGATTCAGAGTC

GTTCAAGTTTACACACTTGGTGATGACAAATGGAGAGATATTAAAACTCCAGAAGCCCTA

AGTTTAAACTGTCTAAAAAACTCTGGAGCAGTTAATATTGGTGGAACAATGTACTGGCTA

ACTGAAGACATGGTAGCTAGCTGGCAGCATGCAGTTATTACCTTTGATCTCGGTGAAGAA

AGTTTTGCACGGATACAACTGCCAGCAACTGTACTCGAAGACTCTGCAGGTGATGGGCCT

CGTCGGTACTGGGTTAGAGAGATTGATGGGAAGATATGTATAGCAACGGCTCAAACCTGT

TCCTTTCAGCCCAGAAGGCTTGTTGGTAAGCTGCAAATTTGGACACTTGAGAACAAAACG

GAGCAAAGGTGGAGCCAGAAGTACAATATTCAGTACTCACCTGATTACATTCCGGGTCCA

AATTTGGTTCATAGGGATAAGACCATATTGCAACGCCGTGACCGTAACCTATATTCCTAT

GAGTTGCTTGGGGACAACTCCAATACTAAATTGTGTAAGATGACAAAGCTGTTAGATTTC

AGGCCCCACAAGCCTGACAACATGCAATCCTACATCTGTGTGAAGTCACTTATACGCTTA

GATGTATACAAGAAGGCTGGCATTGTTCGTAGGCCAAAACAGCGGGGAGTGTGGGAATTG

AAGAAGTGGGAAGCGTGGGAGCACAAGCTCTCTGAGAATGAAAGAATATGGACCGATACT

CATCGAGACGAGCATGTGGGAGCTGCACATGCACAATGCTATCGCACGATGATCAATGAT

GTGCTGCCACGTATATTGGATGACACAATTCAACTGGAAATAGGCATGAAAATCAATCAA

ATATTTCCGAACATTCCTGATCAGCAGCCAAGGCCCCTCCGGCGGCTTAATTGTGTGGCG

CAGAAGCAGGATAGGGAAAATTTATTTGTTCGTATGGAGAATTGGAATAATATTACGAAG

GCTACGAGGGAGGCAAGGCATAGTATCTTTAGCATGATGGTTAATGCTGTATTAAATCAG

ATCGGCGCCTCGAGTTCAGACGCTGGCATTTCTTCTCAGAATCGCAGTGAGGGCGATGAT

GTGAAGATTTGATACTTGAGAGCTTATCATGACATCCTTCTCAGCTCGCTCAGGCTGTTG

TTCAACTCTTGTTTGTTTGTACCCCGAGGGAACGTTTAGCTGTTTCACCTGTAAAACAGT

GATCTATGCGCTGTCATGTTTCCATTTGAATTCTTGATATTTATGGCACTTAAACATATT

GGTCAATTTGAGATGTTGAAGAATCTGCGGGGCAGCAAGACCATTTGATCTGAAGATCAG

ACTAACTCCTGCCACCGAGTATATATTGAAGATTTACAGAGTACTGTACTTGGAAGTGCG

AAACTTGTAGTTGCACTATA

>Traes_5BL_7B7F3279E

TTTTATTTCTCTCATCTCGCGGGGGCCAAGGCGCCGGCGGAGGAGCTCATCTGAATTCGC

CCAGTTTATCCAGTCTCCGAATTCGTTTGACTTGCCAGTCCGTGAAACTCTTGATTGCAG

AAGGATCTGGTAGTGCTCTCACTGTGGCTTCGACCGAAACCATGGCGATGCAGCCAACAA

AGAAGAAACGCCTGGCCAGCTTGCCGCAAGATCTCATCGAGCTGATACTTGTGAGGCTTC

CAGTGAGCGATCTGCTGAGGTGCTGTGGTGTCTGCAAGCAGTGGGATGGCATCATCCGGG

ATCCGCAGTTCACCATGGCACACCTCAGGCGTGCGCGGCCTCGCCCCCTTTTATTTTTTC

AACGAGGCCGGGCTTCAGGCAAGTCCTTCCCTAGCGAAGCAGTCCTCTTTGATGAGGCCT

GGTCGCCATCGACGCGGGATGTGCCCGTGATTGATCCTGACGATTTCTTGTGCGCTTCGT

GCAATGGCCTTGTTTGCTTGTACTCGAGTAACTCAACCATCAAGATAGCCAACCTCGCTA

CCGGTGAAAGTATGCACCTAGCCAAACCCGTCAAGTACGCGACGGAATATCAATCTTCGT

TCTACAGCTTTGGTTTCAGCCCAGCTACAAATGAATACAAGATCGTGCACTTTCTTCCGG

GCCAGAAGCTCCATCCTGCTGGTGGTTCCTTCAGTGTCATTCAAGTTTACACGCTAGGCG

ATGACAAGTGGAGGGACGTCAGAACTCTAGAAGTTGTAAGCCTGTACTGTGTGAAACAAA

GTGGAGTTGTCAATGTTGACGGAGCAATGTACTGGCTAACCCATGACAAAGAAAGCAACT

GGCGACGGGCCGTTATATCCTTTGATCTCAGAGATGAATGTCTAGAAATAATACGGTTGC

CCAAAGTTGATTTTGCGGATCCTGCATTTGCTAATCCGTTCTGTTACTGGATCACCGAGA

TAGATTCCAAGGTGTCTGTGGCTGCTGTTCAAGCCAGAAGAGATTCCGTGCTCGCAAGGA

AGCTGCATATCTGGACACTTGACAACAAGGTAGAGAAAAGCTGGACCCAGAAGTATAGTA

TTGAGTTACCAGCACTTAGCGTTCTTGGACCGCATTTCATCTACGAGGATAAGATTATCA

TGCATAGTCGTAATGGTGGCATATATTGCCATGAATTGACAAGCCAGCGATTTACGATTG

ACGATACCAAGTTGGTGAAGCTGTTAGATTCCAGCCCCCGCAGCTACGAGAATACGCAAT

CCTATATGTGTGTGAAGTCACTTGTTCGGTTGGATGCATACAAGAAGGCCGGTATGGTGC

GCACGCGAAAGAGAAAAGAGGGTTGGAAATTGAAGAAGTGGGAGGCGTGGGAACATGACC

GTTCTAAGATAGAGGAGCTGTGGAGATCAGGCCATCGGATTCAGCAAAGGATGCATGAAA

TGGCACATGTGATCGGCGCAGCCATCAGTCTGAACTTGCCTGCCCCACCAGATCAGCAGG

ATAGCCCCCACCGACGGCTTAATTGGGTGCGGGTGACGGACATGCTGGAGTCTCATGTTG

ATAGTTTGGATGCTCCATGGAAGGTCTTAGTCCAGGCAGCTGACGCCTTCAGTAGTGAGA

CCAAGAAGATGATCCCAGCGGCAGACCAGGCAAAGCAGAGAAATGAAGAGGAGAGCGAGG

CTGGCCCATCAAGGCCAAAGCGGAAGAGGAAGCCCAGCTCCAGATTTGATTCGTCGACGT

GGACAACCTGATTCGGCAGCGCCGGCGGCCACCACCATATTAACAGAGAAGTATAAGAAA

TTATACTTAACTAGTAGAACGCCAGTGCGTCGTCACGGGCATTTTAAAAATCTTGCTGGT

TCATCATTATCATGTTCCACTAAAAGAATATGAGGTTTCCCCAAAAAAGAATATGAGCTT

AATGGATACTAGTAAG

>Traes_2DL_2C9A53175

CAAGTCCTTCATCGACTTCGCCAACTCCGGCGCGGTGGAGAAGGAGCGGCTGCTGGCGAC

GGCCGACGCGGCGGAGATGGTGCTGAGCCCCAACATCGAGGTGGATAGCTGGCTGCGGAT

CCCGTTCTACGACATGGACTTCGGCGGCGGCCGGCCGTTCTTCTTCATGCCCAGCTACCT

GCCGGTGGAGGGCCTGCTCATCCTGCTGCCGTCCTTCTTGGGC

>Traes_1DS_29F18D0C8

ACTAGATTTATCATGAAACATGTTCTTATAATGTATAATTGTGAAAGCTTAAACATAAGC

TATTTGAGCCCTTATCTAAAGGTATTGGGTTCTAATTACAGTAACGGAGTAAATTTTGCC

ATTGGCGGTTCAAAAACACTACCGCGGGACGTTCTTTTTGCATTGCATGTACAGGTGAAG

GAATTTTTGTTCTTTAAAGACAGATCCTTGGAACTCATCAATCAAGGTCAGGAAACCCCA

ATTGATGCAGAAGGGTTCCAAAATGCTCTATATACCATAGACATAGGACAGAATGATATT

AATGCTCTTCTGTCCAACTTGCCTTACGACCAAGTAGTCGCGAAGTTCCCTCCAATACTT

GCGGAGATCAAGTATGCTGTTCAGATTCTGTATGACAACGGGAGTCGGAACTTCTGGATA

CATGGAACAGGGGCTCTCGGTTGCCTGCCTCAGAAGCTCTCTATTCCACGCAAAAATTAC

AGTGATCTTGATCAGAATGGCTGCCTCAAGACATACAACAGAGCTGCAGTCGCGTTCAAC

GCAGCGCTAGGCAGCCTCTGTGATCAGCTGAATGTAGAGCTGAAAAACGCGACCGTTGTG

TACACCGATCTTTTTGCCATCAAGTATGACCTTGTGGCCAGCCACGCCAAATACGGTTTT

GACAGGCCATTGATGACGTGTTGTGGGTACGGAGGGCCACCATACAACTATGATTTGAGA

AGGAGCTGCCAGTCTCCGAATGCGACGGTCTGCACTGACGGCTCAAAGTTCGTTAGTTGG

GATGGCGTGCACCTCACCGAGGCCGCCAATGCTGTCGCGGCTGCAGCCATACTGAGCTCC

GCATATTCCAGACCAAAGATCAAGTTTGATCAGTTCTGCAAAGCCTGA

>Traes_1AL_579194637

CCACTGGCTACTAGTGCAAGTATGGCTCGGGTCTCTGCCAATGCAGTTGCACTTGTTGCA

CTCGTCTCCGTTCTTCTCACGTATGGCTGCTGCGCCCAGTCGCCGCTCAACTACACCGGC

TCCTTGGCCAAATCCTCCAAGGCTAGCTGGTCATGGCTCCCTGCCAAGGCCACATGGTAC

GGCGCGCCTACCGGCGCCGGTCCCGATGACAACGGTGGTGCTTGCGGCTACAAGCACACT

AACCAGTACCCGTTCATGTCCATGACTTCCTGCGGCAACGAGCCCCTGTTCAAGGACGGC

ATGGGCTGCGGCGCCTGCTACCAGATACGATGCGTCAATAACAAGGCCTGCTCCGGCAAG

CCGGAGACGGTCATGATCACCGACATGAACTACTACCCTGTGGCCAAGTACCATTTCGAC

CTCAGCGGCACGGCGTTCGGCGCCATGGCGAAGCCCGGCCAGAACGACAAGCTCCGCCAC

GCCGGCATTATCGACATCCAGTTCCAAAGGGTGCCATGCAATCATCCGGGCTTGAACGTG

AACTTCCACGTCGAGCGGGGCTCCAACCCCAACTACCTGGCCGTGCTGGTGGAGTTCGCG

AATCGGGAGGGCACCGTGGTGCAGATGGACCTCATGGAGTCAAGGAACGGCCGCCCGACG

GGGTACTGGACGGCGATGCGCCACTCGTGGGGCGCCATCTGGCGGATGGACTCCAGGCGC

CGGCTGCAGGGCCCCTTCTCTCTCCGCATCCGCAGCGAATCCGGCAAGACGCTGGTGGCC

AAACAAGTCATCCCGGCCAACTGGAAGCCCGACACGAACTACCGTTCCAACGTCCAGTTC

CGTTGATTGCTCCGAGCTTCCGATCGACCGACGAAGACGTTGATTAATTCGGTACAGTGT

GAATGCAAAGTATATTGTTGAATTTTACTTACTACGGGGTATATGTTGCGTCATCGTGCG

TGTAGCTCGAAGATATGTGTGGGAATGGAAGGAGGCTTTAGCATGGGCAGCCTGGCCCAA

AAGCCTAACATCCAGGCCGGGCTTCTGTCTAGTGGAGTAGTATCCACGTGCTACCCAGCC

CACTATCACCAGATTCACAGCTTGAATCTGATATATTGTAATTAATCAGGTTTATCCATA

TGCATTATGGGATACCAATTGTGTATTCAGTCTGTCTCAAAAAAAAATTGTGTATTCAGT

CAATCAGATGC

>Traes_2BL_46C50CABD

TTGAACGAAGACTCGCCGCTCGGGTCAATGCGTCGCACCATCGCCAAAATCAGGATCGCA

TCGTTCACACGCGGCAGCCCACTATACACTCTACACTACAAACCGCAGGCCTGAGCTGCT

CGCACAAGAGACGAGGCGCAGCGGACGCCGCAAGCAACGCACGGACACTCACCATGGCTC

CTCGCCGCTTTCTCCTCCTCGCGATCCTCCTCGTCCTCCTCGGTGCTCATGGCTTTGGCC

GTGGCGCCGCGCAGGCCGCCGACCAGTTCGCCTACAACGGCTTCGCGGACGCCAACCTCA

CGCTCGACGGCTTGGCCGCCGTGGCGCCCAACGGCCTGCTGGCGCTCAGCAACGGCACCA

GCCAGGCGGCGGGCCACGCGTTCCACCCGACGCCGCTCCGCATGCGGAACGGCACCGTGC

AGTCCTTCTCGGTGGCGTTCGTCTTCGCCATCGTCTCCAACTTTACCGTGCTGAGCGACA

ACGGCATGGCGTTCGTGGTCGCGCCCAGCACCAGGCTCTCCACCTTCAACGCCGGCCAGT

ACCTCGGCATCCTCAACGTCACGGACAACGGCAAGCCCGACAACGGCGTCTTCGCCGTCG

AGCTCGACACCATGCTCAACCCGGAGTTCCAGGACATGAACAGCAACCACGTCGGCGNNN

NNNNNNNNNNNNNNNNNNNNNNNNNNNNNNNNNNNNNNNNNNNNNNNNNNNNNNNNNNNN

NNNNNNNNNNNNNNNNCTCATCAGCCGCCAGCCCATGCAGGTCTGGGTGGACTACGACGG

CGCCACCACCCGGCTCGACGTCACCATGGCGCCCCTCGACGTGCCCAGGCCCAAGAAGCC

CCTCATCTCCGCGCCCGTCAACCTCTCGGCGGTCGTCACCGACACGGCGTACGTCGGGTT

CTCGGCGGCCA

>Traes_1DS_685E35174

CCTTGACAGTTTGCTATCATAGTCAAGTGCTGCCCCAACAGCCACCATCCATGGAATGTA

GCGGGGGCTACAGTCAGTAGCATACTAACTCATTTAACTCATTTCTATCATATGACTTTC

TAAACGGGCACACATGCATGCTGCTGCTTTGCAGAACTTTTCCCAGTCGAACATTTCTAA

GGTTGAGCATCTATATGGAAAGAAACACTAGTATAAAAACATCAAATTGGTATTACTAGA

GTTATCATGAAACATATTCTTATAATGTATAATTATTTTTATGTGAGGCTACATGTCTGT

GCATAAATTGATGTTCAAAGTTTCATCTTGAATATTATACAAGTTAATGTCTAATAAGAC

AACAAATTTTATCGGAGTTAATACTTTGCTGCTGGTGTGTTTTGCGCATCTAATCTGACT

CGCGTACATGTTGATGAGAAGCACTATCTAATTATTGAGTATGGTTGTTGGCCTTACTAA

TACTGGGTCCATGAATGACCAAAATATATCCAGGTGAAAGCTTAAACATAAGCTATCTGA

GCCCTTATCTGAAGGCAATTGGTTCTAATTACAGTAACGGAGTGAATTTCGCTATTAGCG

GTTCAACAACACTACCACGGGAAGCCCTTTTTGTATTGCATGGACAGGTGCAGGAATTCT

TCTTCTTTAAAGCCAGATCCTTGGAGCTCATCAATCAAGGTCAGGAAGCTCCAATCGATG

CAGAAGCGTTCCAAAATGCTCTATATATCATAGACATAGGACAGAATGATATTAACGCTC

TTCTGTCCAACTTGCCTTACGACCAAGTAGTCGCCAAGCTCCCTCCAATACTTGCGGAGA

TTAAGTATGCTGTTCAGCTTCTGTACGGCAACGGGAGTCGGAACTTCTGGATACATGGAA

CAGGGGCTCTTGGTTGCCTGCCTCAGAAGCTCTCGATTCCACGTAAAAACGACAGTGACC

TTGATCAGAATGGCTGCCTCAACACATACAACAGAGCTGCGGTCGCGTTCAACACAGCGC

TAGGCAGCCTCTGCGATCAGCTGAATGTAGAGCTGAAGAACGCGGCCGTTGTGTACACCG

ATCTCTTTGCCATCAAGTATGACCTTGTGGCCAACCACACCAAATACGGTTTTGACAGCC

CATTGATGACGTGTTGCGGGTACGGAGGGCCACCGTACAACTATGATTTGAGCAGGAGCT

GCCAGTCTTCGAATTCAACGGTCTGCGCCGACGGCTCAAAGTTCATCAGCTGGGACGGCG

TGCACCTCACCGAGGCCGCCAACGCCGTCGTGGCTGCGGCCATACTGAGCTCCGCATATT

CCAGACCAAAGATCAAGTTTGATCAGTTCTGCAAAGCCCGATAGATCATACTTCATACTA

TATTGGTGTAGTACTTATACATAACTTAAACCTGCAAGGATCTGGTTTTTCTCCTATTTG

GGGCTGACAGCCGATTAATGTGGACGTGACATCTGCAATTCGGG

>Traes_2DS_1689489FF

CGGTGAGCGTGGACCTCGGGCCCGAGGACCTGTCGCCGTTCCTCGTGTCGCCGGAGCTGT

ACCCCAAATACCTAGACGTGTCGATCCGGCAGTTCGAGGGCCTCGAGGACGCCGGCGACG

TGCTCGTCAACTCCTTCCGTGACCTCGAACCGCAGGAGGCCGAGTACATGGAGTCCAGAT

GGCGCGCCAAGACGGTCGGCCCGACGCTGCCGTCCTTCTTCCTCGACGACGGCCGCCTGC

CGTCCAACAAGGCCTACGGCGTCAGCTTCTTCAGCAGCGACGCGCCCTGCATGGCATGGC

TGGATCGGTAGTCCTCGCGTCCTACGGGACGGTCTACAGCCTCGACGCCGGCGAGCTTGA

CGAGCTTGGAAATGGGCTCTGCGATTCAGGCAGGCCATTTCTCTGGGTCGTGAGGTCCAA

CGAGGCACAGAAGATATCTGAAGAACTCCACGGCAGATGCAAGGAAAATGGATTGATTGT

CCCTTGGTGTCCCCAGCTTGAGGTTCTCGCGCATAAGGCCACAGGTTGCTTTTTGACTCA

TTGTGGATGGAACTCAACGACAGAAGCACTTGTTGCCGGCGTGCCAATGGTGGCGATGCC

GAGGTCGGCCGACCAGCCAACCACGGCAAAGTACGTGGAGAGCGCATGGGGCATCGGCGT

GGGGATGCACACAGATGAGAAAGGCTTGGTGAGAAGGGAGGAGGTGGAGAGGTGCATCAG

GAAGGTGATGGATGGGGAGGGAAAGGTTGAATACCGCAAAAATGCCACAAAATGGATGAA

GATGGCCAAAGAGGCAATGCAGGAAGGAG

>Traes_4DS_B9FC9F877

TTGAGGCGAAGGTGCAGCTGACATCAGTTGAGAACCAAATTGACAGGCTGTTGGAAGGTA

ATGTCTTCTGGTCCTTCCAACATACACCTCAGTTGCACGACTTGCATTCTAGTTTATTTT

TCTTCTGTTTGCTGATCTGGATTCTCTGTTAAGCTAAGCACTTGTGCCGATGCTTTGCCT

CCAATCGTCGAGACATGGCATCCACTTAGTGATGCTTGTCTCCTTAACAAGATTGTAACA

GATAGAGATGCTTATGCTGCTCCAAAAAATTGATTCATCATTGGCAATACATGGAGCGTG

GTGCACAATATTCCTTGCCAGCAAGACTGCTTTTGTTTCTTGACTATAAATAGAGTGCAC

CACAATGGTTGAGCACCAACAAAGCGCATCTGCTCTTCACTTTCTCTTCCATTACGAACA

TACACACATCATCAGTTCATCCATTAGAAACATGGTTTGCCATCAAAGATCCGCAAGCTT

GCCTTACTCCAGTGAATCCCAAGTGGAGGTGGAGCTGCAGGGCCTGAAGTCATGCATCTC

TTCACCCTCCGCAACCATCGACACAATGTGTGGTGGTTACGCAAGGCTTGGAGACATCTA

CAAGTGCATAGAGGAGATCATGGGCTTGCCCAGCAACCAAGTTGGCCTCTCCTTCCCCCA

AAACAAGAAAATGGTGGAAGAGRAACTGGAGCGATCCCTCCTGCTGATTGATCTCTGCAA

TTCAATGCAAGAGAATTTAGCAGAGCTGAAGATGAGTACCCAGGAGCTGCAACTGGTTCT

CAAAAGATGAGATGATGCGTCTGTTCAACTCAAGGTTGAGTCTTTCATTCGTCTCGCCAA

GCAGGCGCAGAAGCCTTTCAAGAAGATTGCAAGCAGCAAAGCTAGTGGTGAGGATTGCAG

GCTGATCAGGCTACTGGCGGAAGCCAGAGAGATGTCTGTCTCTCTACTCGAGTCCACGTC

GCAGCTGTTGCCGAAGCAGTTCGCCACCACCAAAGGAAGCAAATGGTCTCTTGTCCAGAA

GAGAAGGGTCGTTTGCGAGGAGGAACAGTTGCAAGCGCTAGAGCGAAGCATGGGAGATGT

TGAAAACGGCGTTGAGCTTCTGTTCAGGAGATTGATCCAAAGCAGGGTTTCGCTCCTGAA

CATTCTCAGCTCCTAGACACTCTCAGGCCTTGTTTCTCTAGCATATTGCGATTGGCATCC

ACCTTTTAAAGGATTCACCAGTCATATAGAACCTTTCTAGCTTGTGTACTGAGTACACTA

CTAAATGTACATACAAATGGTCAGTTGATGAAAGCAGTTTTAACTATGCCCATAAAATTA

CTTCTAATTTCACCGATCTTTACCGTGCGATGATTTAGATTTATTTTTCATAGCTGAATG

TCATGT

>Traes_6DL_2CD01D459

GCCGTATTCTTCCTAGCAGCTCTGGCAGCCTTTACCTTCGGATTAGCGTCAACACACTGA

GCAATCCATACATCACACACACAGCACCAGAGGTTTAACTTAATTAGTCGGCCGAGAAGA

GCAGATGAGTGAGCAAGGTAGCTATGGGGAGGTCTCCTTGCTGCGAGAAGACAGGGATCA

AGAGGGGCCCGTGGACGGCGGAGGAGGACATGACCCTGGTCGCTCACATCAAGCAGCACG

GGCACAGCACCTGGCGGGCGCTGCCGAAGCAGGCCGGCCTGCTGCGCTGCGGCAAGAGCT

GCCGCCTCCGGTGGATCAACTACCTGCGCCCCGACATCAAGCGCGGTAACTTCACCAGCG

AGGAGGAAGACGCCATCATCCAGCTCGACGCCATGCTCGGCAACAGATGGTCCACCATTG

CCGCCAGGCTGCCTGGGAGGACGGACAACGAGATCAAGAACGTCTGGCACACACACCTCA

AGAAGCGACTCGACTCGTCCTCGTCCAAGACGTCCGGCCAGGCATCACCTAAGCGCAAAG

CCGAGAAGCCTGCTGTGGCTGCGAGCACGCTCGAGGATCCGACCTCCGACCCGGTGTCGC

CGGAGCAGTCCCTGTCGACGTCATCTGCCACCGACTACTCGATGGCCTCGTCGTTGAACA

CGGGCAGCCTCGGAAGAGTTCCAGATCGACGACAGCTTCTGGTCGGAGACACTTGCGATG

TCGGTGGACAGCTCCGGTTCCGGGATGGCAACCAGCGACACCTTCGGCGCAGATCGCGCA

TCGCCGTTGTCGAGCAACGATGAGATGGACTTCTGGGTCACGCTGTACATGCAAGCTGGT

GACATGCAGAATTTGTCACAAATTTAATGAGAAAATTGGGTTATTTTACATAACATATTT

TTGAAGAAAAGGGGTTCCCCCTCCCAATTTTTATTGGAAACCAG

>Traes_6BL_5614B8A85

GCACTGTACTTATGAAGGAACAAGGTGGGCTGAAGTTTACTACCAGAGTGCAGCCTCTCC

GTCTTGCTGAATGGTCCACGGATGACAAGTTTGCTTTCTTCATGAGCGGAAGAAAGTACA

CATTTCGAGATTTTGAGAAGATAGCAAATAAAGGATTTGTTCGAAGATACTCTAGTTCTG

CCTGTCTTCCAGCAAGGTATATGGAGGAAGAATTTTGGCATGAAATAGCATTTGGCAAGA

TGGAATCCGTTGAGTATGCATGCGATATTGATGGTAGTGCATTCTCTTCTTCTCCTAATG

ACCAACTTGGGAGAAGCAAATGGAACTTGAAGAAACTTTCTCGGTTGTCCAAATCAATCC

TGCGCCTTCTCAGAACAGCAATTCCAGGAGTAACTGACCCAATGCTGTATATCGGAATGC

TCTTTAGTATGTTTGCCTGGCACGTGGAAGACCATTACTTGTACAGCATTAACTATCATC

ACTGTGGTGCTTCAAAAACATGGTATGGGATCCCAGGAAAAGCTGCTCCAGATTTTGAGA

AAGTGGTACGTGAGCATGTATATGATCATGAAATTTTATCAGGTGAAGGGGAAACTGCAG

CATTTGATATCCTTTTGGGGAAGACAACAATGTTCCCTCCAAATATTCTGCTCCATCATC

ACGTTCCAGTTTATAGAGCTATACAGAAACCGGGAGAGTTTGTGATCACGTTTCCTCGAG

CATATCATTCAGGTTTCAGCCATGGTTTTAACTGCGGCGAGGCAGTGAATTTCGCTGTTG

GTGAATGGTTTCCTCTTGGAGCAATTGCTAGTCAACGTTATGCACTTCTAAAGAGGATAC

CATTACTGCCTTATGAGGAACTTCTTTGTAAAGAGGCAGCACTTCTTGATCATGAATTTT

CTACACCTAGTTATAAAGATTTGACAACATCAACTGGAGATACACACATTCAGCATTGTA

TGAAAGTCCCTTTTGTGCAGTTGATGCGGCTCCAGCATTGTGTCCGTTGGTCACTTATGA

AAATGGGTGCTCGCACGCACTATAAAGCAGATATTGATGCCACAGTTCTCTGTAGCATAT

GTAAACGTGACTGCTATGTAGCCCATGTTATGTGTAACTGCAGAGTTGATGCAATTTGCC

TTTGTCATGAGGAAGAGATTAGGAAGTGCCCTTGCAGCCATGATCGTGCTGTATTTGTGA

GAAAAGACATTATTGAGTTGGAGGCACTGTCAAAAAAGTTTGAAGAGGAAAAGGGAATAG

TCAATGCTGTCAGAAAGCAAATGTCTCGTGGCTCTAGCACACATTCTTATTTCAACCGCA

TTAGTCACAATGCTGAATACTTCCCATACTGCAAGATTCACATAGATGCACCACCTGAAG

TTCATAGCATTTCAGAGACACATGTTTTTGGATATGATCTGAACAAGCCATATCCTGATG

CATCAACAATAACATTTTGTTTTGGACCCCATGAGTATTCTACACAAAGTGATGAGTGCA

CAAGTACTAACAGAAGGATCTTCGCTAGCTCTTGTCCTGAGAATGGATTTACTCCTCAAA

CCACAATCATTAATGCATACCCTTCGTCTGCGCCTGATCAAACATGCTCTTCTGAAAAAT

TGGCTGCAGAAGATGCTGATGATTCTGACTGTGAGGTATTTAGGGTGAAGAGAAGGTCTG

GCATAACTCCTGAGAGAAGGCATACAGAAGATTCAACCATAACAACTTTCACAGGGAATC

AGGTACAGA

>Traes_6BL_099BB023B

AAATTGCACTAGAAGCTCCTAAACTTGTGCAGTGAAATCACTTAGGTCTAAAAGCTTGTT

TTGTGTATGGTCAACTATTTCATGCAAGCACACAAAACTCGCAAATAAACAAATTAAAAT

CCCTATTCTCCCAATGCATCAAACAAGAGATCAGAACAATCTAATATNAGGGGAATGTGG

GTCCCGGCTTGAGGCTGTCGCGGGACGGGAGGCCACGGTGGAGTGGAGGCGCATTTTATT

GAGCTGGGGATATAAAGCATGGGTGCCGTCAGCCTGTAATCTCAGGATTTGTGGGTCAAG

GGGTTGATGTCTTCAGGATAATGGCAGGGAAGGAGGAAGCTGCTCTGAAACCTGTATCAT

GTGGGGCAAGGTTACGTAGGAGTCGTGATGCATCACTAAGAGAAGAAGTGTCCATGAGGG

ACCCTTTCTTGAAGCACAGAGTGAAGAAGTTTGATCTATCTAGCCTAGATTGGATTGATC

AGATTCCAGAATGCCCAGTGTTTTCTCCATCGGTGGAGGAGTTCGAGGATCCATTTGTTT

ATCTCAGTAAAATTGCCCCTGTTGCTGCAAAATATGGTATAAG

>Traes_4BL_4D370C9FA1

TTCTCGGACGCGTCGAACGCAGTCGTCACCGTCCCCGCCTACTTCAACGACTCCCAGCGC

CAGGCCACCAAGGATGCAGGCGTCATCTCTGGCCTCAACGTCATGCGGATCATCAACGAG

CCTACCGCCGCCGCCATCGCCTNCAAGAAGTCCACCAGCGTCGGCGAGAAGAACGTGCTC

ATCTTTGATCTCGGCGGCGGTACCTTCGATGTCTCGCTCCTCACCATTGAGGAGGGTATC

TTCGAGGTCAAGGCCACTGCCGGAGACACCCACCTGGGAGGCGAGGACTTCGACAACCGG

ATGGTCAACCACTTCGTGCAGGAATTCAAGAGAAAGAACAAGAAGGACATCAGCGGCAAC

CCGAGGGCGCTCCGGCGGCTCAGGACAGCGTGCGAGAGGGCGAAGAGGACACTCTCCTCC

ACCGCCCAGACCACCATCGAGATCGATTCCCTCTTCGAGGGCATCGACTTCTACACTACC

ATCACCCGCGCCCGGTTCGAGGAGCTCAACATGGACCTCTTCCGCAAGTGCATGGAGCCC

GTTGAGAAGTGCCTCCGGGACGCCAAGATGGACAAGAGCACCGTGCACGATGTTGTCCTC

GTCGGAGGATCCACACGTATCCCCCGTGTGCAGCAGCTCCTCCAGGACTTCTTTAACGGG

AAGGAGCTCTGCAAGAGCATCAACCCTGACGAGGCCGTCGCGTATGGAGCCGCCGTTCAG

GCTGCCATCCTCACTGGCGAGGGCAACGAGAAGGTGCAGGACTTGCTCCTGCTTGACGTC

ACACCGCTCTCGCAGGGCCTGGAGACGGCCGGAGGTGTCATGACCGTGCTGATCCCAAGG

AACACGACAATCCCCACCAAGAAGGAGCAGGTCTTCTCCACCTACTCTGACAACCAGCCC

GGCGTGCTCATCCAGGTGTATGAGGGCGAGAGGGCGAGGACCAAGGACAACAACCTGCTC

GGCAAGTTCGAGCTCTCCGGGATCCCACCGGCGCCCAGGGGTGTCCCCCAGATCACCGTG

TGCTTTGACATTGATGCCAACGGTATCCTGAATGTCTCGGCGGAGGACAAGACCACCGGA

CAGAAGAACAAGATCACAATTACCAACGACAAGGGGCGGCTGAGCAAGGAGGACATCGAG

AAGATGGTGCAGGAGGCCGAGAAGTACAAGGCTGAGGACGAGGAGCACAAGAAGAAGGTG

GACTCCAAGAACGCCCTGGAGAACTACGCTTACAACATGCGTAACACCATCAAGGATGAC

AAGATCGCCTCCAAGCTCCCAGAGGCCGACAAGAAGAAGATCGAGGATGCTATCGATGGT

GCCATCACCTGGCTCGACAACAACCAGCTCGCCGAGGCTGAAGAGTTTGATGACAAGAGG

AAAGAGCTGGAGGGCATTTGCAACCCCATCATCGCCAAGATGTACCAGGGTGCCGGCGCT

GAAATGCCTGGTGGCATGGATGAGGATGCTCCGGCCAGTGCCGCAGGCGGCAGCAGCGGC

CCAGGGCCCAAGATTGAGGAGGTCGACTAA

>Traes_2AL_9F4901A76

CACTCAGCACCACACAACGAACGGCGATGGGGAGAGAGAAGCACGCGATGGATTCCGTGG

CCGTCGTGGCGGTGCCGTTCCCGGCGCAGGGCCACCTCAACCAGCTGCTGCACCTGTCGC

TGCAGCTGGCGTCGCTCGGCCTGGGCGTGCACTACGCCGCGGCGGCGCAGCACGTCCGGC

AGGCGCGCACGCGCGTGCACGGCTGGGGCGACGAGGCGCTCCGCTCGATCCACTTCCACG

ACCTCGGCATCGCCAGCTACGTCTCGCCGCCTCCAGACCCCGCCGCCGACTCGCCCTTCC

CCTCCCACCTCATGCCCCTCTTCGAAGCCTACACCGCCCGCGCGCGCGCCCCGCTCGCGG

CCCTCCTCCAGGACCTCTCCGGTTCCTGCCGCCGCGTGGTCGTAGTGCACGACCGCATCA

ACGCCTTCGCCGCCGAGGAGGCCGCGCGGCTGCCCAACGGCGAGGCGTTCGGGCTCAACT

GCGTGGCCGTCTCCATGCTCATCGGAAGAATCGACGCCGACCACCGGCTACTGCGTCAGA

ACGGCGTCGTCTTCGGCGCCGTCGAGCGCTATGCGACCAAGGAGTTCATAGAGTACGCCA

ACCGGGCCAGACCGGCGAAGCACATCTCGACCGGCGCGGGCATCCTGGCAAACACGTGCC

GCGCGCTGGAGGGTGATTTCATCGACGTCGTCGCCGGGCACCTGGCCGCCGACGGCAAGA

AGCTCTTCGCCATCGGGCCGTTAAACCCACTGCTCCACGACAGCGCGCCGAGGCAGAGCA

AGCAGCGCCACGAGTGCCTGAACTGGCTGGACGAGCAGCCTCCGGCGTCGGTGTTGTATG

TGTCCTTCGGCACCACGTCGTCCCTGCGAGCCGAGCAAATTGAAGAGCTCGCAGCAGCAC

TGCGCGGCAGCAACCAACGGTTCATATGGGTGCTGCGCGACGCCGACCGCGGCGACATAT

TTGCCGAGGCCGGCGAGAGCCGCCACGACAAGTTCCTATCAGAGTTCACCAAGCAGACGG

AAGGGACGGGGCTGGTGGTCACCGGGTGGGCGCCGCAGCTGGAGATCCTAGCGCACAGCG

CCACGGCGGCGTTCATGAGCCACTGTGGCTGGAACTCGACCATGGAGAGCATGAGCCACG

GCAAGCCGATTCTGGCATGGCCCATGCACTGCGACCAGCCGTGGGACGCGGAGCTTGTCT

GCAACTACCTCAAGGCCGGCGTCCTCGTGCGGCCATGGGAGAAGCACGGCGAGGTGGTCA

CGGCGAAGGCCATACAGGAAGTCATCGAGGAGGCCATGCTCTCCGACAAAGGAATGGCCA

TGCGGCAACGGGCAAGTGCGCTCGGGGAGGCCGTCCGCGCCTCCGTGGTTGACGGCGGTT

CGTCGCGCAAGGACCTAGATGATTTCACTGCTTACATCACAAGGTGATCGACAAAATGCC

ATCTGGGACGATGGCCTTTCACGCCGACACAGTTTCTACCTGCTGATGGTACACTGTCCG

ATATGTAGTTCTTCACTATTGGTGAATCCAAAAATAAAATTATAGGTTGCAATAGCAGTT

GCTGATGTGAAGCTGCACCAATGGAACATTTTTCTGAACTTCTTTTAGGTTCTTTCTGCT

GCTAAAAGTTGGGATTGCCATAGAGATGCTTTGAGAACACAAAGTTTATTACTCCCTATC

TTTGCTTTTTCCTTTTTGTACATTC

>Traes_2DS_4BF700485

ATGAAGGAGAGGAGCTCTTTATGTGATAGTGCAGCAGATGGAAATTGGGTTTCAAAATTC

AAAAGAAAGCGAAGCAAGTTAACAGCTAGCTCGTCAAATGAGCATGAGGCTACATCACCA

ACATCGGACTCTATAATGAACAATGGTTCCATAAAAAAGAGGTCCAAGCATGACACTAGC

ATTTCTACATCAGCCAAGAAGATAAGAGGACACGATGGGTATTTCTATGAGTGTGTAGAA

TGTGACCTTGGTGGCAATTTGTTGTGTTGCGATAGCTGTCCACGGACATACCACTTGGAA

TGTCTTAACCCCCCCCTTAAGCGTGCACCGCCTGGGAACTGGCAATGCCCAAGATGTCGT

ACGAAACAAGTTAGCTTGAAGTTGTTAGGAAATGCGGATGCTGACACTTCCAAACGTGAA

AGAACAAGGAGAATGCGTACAAGCACCATAGCAGATAGCCCATCTCCCCATAACAAAGTC

TCTCTGAATACCCGGAGTCCCATACAAGAGAAAAGTGAATCAAACGAACAAGTAAAACTA

TCATGCCCTGGTGCATTGAAGGAAGGTGATCTCAGTGTGAAGGACAATGAAGTTCAGAAA

AAGAAGCCATTGATAGTACATTTGAAGAGGCGCTCGACCAAAGAATTATCTGCAATTACG

AAGCCCTTGAAGTCAGGATTCATTGGCCAGTCTTCAGAAGAGAAACGGGAGAAACATGGA

GGTGCTTTGAAAATGAAGAAACATCTGCGCTCCATGGACTTGTCTCCAAAGAAATATAAA

ACCAAGAGGCAGCATAGTCACAAAGACTCTAAGCGATCTGAAACAAAAAGGCTCAGATAT

TTGGCAACTGATGTTGGCAGTGATTCGTCAATGGGGGCATCTACATCTCCTGAGCACTGC

GAATCACCACCAAAAAGGAGATCTCTGGATGGCAGAACACCTACATCTAGTGCCAAGAAA

GGGAAAAAGAAAGTGAAATTTGTTGATAAACAACATTCAGAGGTGCTACCTGTGGCGGGG

GATATGATAATAACTCCTCAGGAGGATCTGCAGGTTGACCGCATCCTAGGCTGTCGGCTT

CAGACAAGCCAAATCATTTCGCAGCCCCATGCTTTATCAGAGCAGATTGAATTAACTAAT

TTGCAGCTGGAAGGTGGGGTTCCTTCTAGTTCTTCCAGTGTGCCAACACAGCCGTTAAGA

AATGATACTGACACCACTCCAGAGGATGTATGCGCTGATGAATCAGCACATGATCCCTGT

GAAGATCATTTGGATGGTGGTGAAATGCAAAAGGAGAATAATGGAAAATACCATGAAAAG

GAGTCAGTGAACCCAGAGGAAGCCACAAAGACACTGTCAGATTGTTGCACCGACCAAATC

ATCACAGTAAAGGATGCTGGAGTAGTAGGAGAGAACATACCTTCAGTAAATGGTGACTTT

GAGGCAGTATCTGATGTTCCAGTTGAAGGAACATCGGAAAAGGGTGATATCAAACTTTCA

GTTTCCGAAGTTGATGCAACTGTGCAGACCAAACAAGAGTTGACACCTGAAAGTAAAGTC

CATGGAAACATAAACGAAATTGCAGGAAATGGCCATGATGATACTGCATATGAATTTTTG

GTCAAATGGGCTGGAAAATCAAACATCCATAATAGCTGGATTTCTGAATCTGAACTAAGA

ACATTAGCAAAACGGAAACTAGAAAATTACAAAGCAAAGTATGGAATGGGTTTGATAAAC

ATTTGCAAGGACCAATGGTGCCAACCACAACGCGTTATTTCTCTGCGTGTTTCCTTAGAT

GAAGTAGAAGAGGCTTTAATCAAATGGTGTGGCCTTCCTTATGATGAATGCACCTGGGAA

AGATTAGATGAACCTATCATGCTGAAGTATGTCCATTTGGTTACTCAGTTCAAAAACTTT

GAATGCCAAGCTTTGGACAGGGATGTGAGAGGTAACCGTGCAAATGCAAGAAACCGCCAA

GAGCTGACTGTGTTGATTGAGCAGCCAAAAGAACTTAAGGGGGGCATGCTCTTCCCACAT

CAACTGGAAGCATTGAACTGGCTGCGTAAATGTTGGTGCAAGTCAAAAAATGTTATCCTT

GCTGATGAGATGGGTCTTGGGAAGACGGTGTCAGCTTGTGCTTTTCTGTCGTCCTTGTGC

TGTGAATTCAAGATTAACTTGCCTTGTCTTGTCTTGGTTCCTCTTTCTACTATGCCTAAC

TGGATGGCTGAATTTGCATCTTGGGCGCCTCATTTAAATGTTGTGGAGTATCATGGTTCT

GCACGGGCAAGATCTACAATTCGTCAATATGAGTGGCATGAAGGCGATGCAACCCAGATA

GGTAAAAGCAAAAAGTCTCACAAGTTCAATGTCTTGCTTACCACTTATGAGATGGTGCTT

GTTGATGCTACATATCTTCGTTCTGTGCCATGGGAAGTTCTTATAGTTGATGAAGGGCAT

CGTCTAAAGAACTCTAGCAGTAAACTCTTCAATTTGCTCAATACATTTTCATTTCAGCAC

AGGGTTTTGTTGACTGGAACCCCATTACAGAACAACATTGGTGAAATGTATAATTTGTTG

AACTTCCTACAGCCTGCTTCTTTCCCTTCTCTAGCATCATTTGAGGAGAAGTTTAATGAA

CTTGCAACAGCAGAGAAAGTGGAGGAGCTGAAGAAACTGGTAGCACCACATATGCTTCGA

AGGCTGAAAAAAGATGCAATGAAAAATATCCCCCCGAAGACAGAGCGAATGGTGCCTGTC

GAACTGACATCAATCCAGGCTGAATACTACCGTGCTATGCTTACAAAGAACTACCAAGTA

CTGCGTAATACCGGAAAAGGTGGTGCTCATCAGTCATTGCTCAATATAGTAATGCAGCTT

CGGAAAGTTTGCAACCATCCATATCTTATTCCTGGAACTGAACCCGAATCAGGTTCACCA

GAGTTTTTGCATGAAATGAGAATAAAGGCCTCAGCAAAGTTAGCTTTGTTGCATTCTATG

CTCAAAGTGTTACACAGTGATGGGCATCGTGTTCTTATTTTTTCTCAGATGACTAAACTT

CTTGACATCCTCGAAGATTATCTGACTCTGGAATTTGGTCATAAAGCATTTGAAAGAGTA

GATGGTTCGGTGTCTGTGGCTGAGCGCCAGGCAGCTATTACTCGTTTCAATCAGGACAAG

ACCCGTTTTGTATTTTTGTTATCTACACGGTCATGTGGTCTTGGGATCAATTTGGCAACT

GCAGACACTGTTATCATATATGATTCTGATTTCAATCCGCATGCTGATATACAGGCGATG

AATAGGGCACACAGGATTGGGCAGTCAAACAGACTTTTAGTGTACAGGCTTGTAGTGCGT

GCTAGTGTTGAGGAGCGTATCTTGCAACTTGCCAAGAAGAAATTGATGCTTGATCAACTT

TTTGTTAACAAATCAGAATCACAGAAGGAAGTGGAAGATATCATTCGCTGGGGAACTGAG

GAGCTCTTCAGGAATAACGATGAGGTGAATGGTAAAGATAATGATGAAGCTTCTGGTGCT

AAACATGATGTATCTGATATTGAGGTTAAGCATAGGAGGAAAACTGGTGGCCTAGGTGAT

GTTTATGAGGATAAATGTATTGGCAGTTCTACCAAGCTTATTTGGGATGAAAATGCTATT

ATGAGGCTTCTTGATAGATCTAGCCTTCCAACAACTGTAGCTGAAAGCACCGATGGGGAT

TTGGACAATGATGATATGCTTGGCACTGTAAAGTGATT

>Traes_2AS_8615CCEF0

AAGCAAAGCAGCAAACCATTTCCATGGAGCTAATCGGAGCCACCCTCAGCTTCTGCTTCG

TTTCTCTCATCACAGTGTCTGTGATTCTAGTTTCCTTGCTTAGCCGCAAACTAGCACCAA

GTTCCGACAAGCGGCGGCCTCCTGGCCCATGGAGACTTCCCTTGATCGGAAACCTGCACC

AAATCCTAACCACCAAGCTGCCCGTCGTCCTCCGGGACCTGGCAAAGAAGCATGGGCCGG

TCATGTACCTTCGGCTGGGCCAGATCGACACCGTGGTGATCTCCTCGCCGGCGGCCGCGC

AGGAAGTGCTGCGGGACAAAGATCTTACATTCACGTCGCGGCCGAGCATTCTGCTCTCCG

AGGTCACCCTCTATGGAAACCTCGACATCGGCTTCGCGCCATACGGCTCATACTGGCGGA

CGCTCCGCAAGATCTGCACGGTGGAGCTCCTAAGCGAGCGCAAGGTGAGGCAGTTTTCGC

CCGTAAGGGACAGCGAGACCATGCGCCTCGTCAGGAACGTGGGCGACGCCGGCAGAGGTG

GCAAGCCGTTCAATATCAGCAGGCTGCTCGTGTCGTGCTCAAATTCGATAACCGGGAGGA

CGGTGTTCGGGGAAATGTGCAGCCCCGAGCTCCAAAAGGAGTTTATGTCCGCGTTGGACA

AGGCGCGCAACCTTGCCGCGGGGCTCTGCATCGGGGACCTCTTCCCGTCGCTGCGGTTCA

TTGACGTGCTCTCCGGGCTGACAGGCCGGCTATGGCGAGCCCGCCACCAGCAGGACAAGG

TCCTGGACGAGATCATCTCGCAGTCCGAGATGCGGCAAGGTCATTACCTTCTCAGCGTTT

TGCTTAGGATCAGGGACGAGGGGGAGCTTGACTTCCCGATGGAACTCGATAACATCAAGG

CAATCATAATGGATATGTTCGCGGCCGGGACAGACACGACATCATCAGCTGCCGAGTGGG

TCATGTCGGAGTTGATGAGGAACCCGGAAGTGATGGCCAAGGCGCAGGCGGAGGTGCGAC

GAACATTCGGCAATAAAAGCCCACAAGACCATGAGGGACACATTCTGGAGCTACCCTACA

CAAATATGGTAATCAAGGAGACCATGAGGCTAAATCCAGTGTTGCCGCTGCTGGTTCCCC

GTGTCTGCCGGGAGACCTGCAGCGTTGGTGGGTTTGAGGTCACGGAGGGTACCAGGGTTA

TGGTAAATACCTGGGCACTGGGTAGAAACCCTGAGTATTGGCTTGAACCTGAGGAATTCA

GGCCGGAGAGGTTCGAGGATGTCAGGACAAGCCAAAAAGGCGCACAGTTCGACTACTTGC

CATTCGGTAGCGGAAGGAGAACCTGCCCAGGAGATATCTTTGCGCTGGCTGTGTTGGAGC

TCATGGTGGCACGACTTCTTTACTACTTTGATTGGAGCCTCCCTGCGGGAGTGAAGCCGA

GTGAGCTGGACATGGAAATGACAGTTGCCTTATCGGCAAGGAGGAAGAACCAACTGCACC

TAGTGGCGAAACCGTATAAAGCATATGCAGTTGTTAGTTGAACTTTACTTATGCACGTTA

TGGGGAATAATACTGGTTGAATTGTACACTTGAGCCTTCTATATGGGGGATTAATAGTCC

GATTGAGCTAGGCGAGTCATGGTGTGATCTCATGGTAAGGGGA

>Traes_4DS_1684EB8521

CGGCTCGGCCGTGACGGAGCTCGACGTGGCCGAGTGGCCCGGTACGAACACGCTAGGCGT

GTCGATGAACCGCGTGGACTTCGCACCGGGGGGCACCAACCCGCCGCACATCCACCCCCG

TGCCACCGAGATCGGCATCGTGATGAAAGGTGAGCTTCTCGTTGGAATCCTTGGCAGCCT

TGACTCCGGGAACAAGCTATACTCCAGGGTGGTGCGCGCTGGCGAGACGTTCCTCATCCC

ACGGGGCCTCATGCACTTCCAGTTCAACGTCGGTAAGACCGAGGCCTCCATGGTCGTCTC

CTTCAACAGCCAGAACCCCGGCATCGTCTTTGTGCCCCTCACGCTGTTCGGCTCCAACCC

ACCCATCCCAACGCCAGTGCTCACCAAGGCACTCCGGGTGGAGGCCGGGGTCGTGGAACT

TCTCAAGTCCAAGTTCGCCGCTGGGTTTTAATTTCCTGGGAGCCTGCCCTGAAATGATCA

ATATATAATTCAATATATGCATGCCAGCAATACTTAATAATTCTCATCAGAAGACATGTC

TTCAAGCTTCGTGTTAATCTCGCATGCAGTTAATTATAATAAGATAGAATAAGTTAGCCT

CATGGTTTAGCCTTCAGATCCAATATGGGGAATTGTATGTACTACTTTTTATTGTCGTCT

TTGTTCTTTTCATTGAACAGAATATATAATTAGCATTTTCGTA

>Traes_2AL_5C6928F55

ACCATGGCGACCGCTCCTGTCACGGTGGTGCCGCGCCTGAAGCTGGGCTCCCAGGGTATG

GAGGTCTCGGCGCAGGGCCTCGGCTGCATGGGCATGTCCGCCTTCTACGGCCCGCCCAAG

CCCGAGCACGACATGGTCGCGCTCATCCACCACGCCGTCGCCGCCGGCGTCACCCTGCTC

GACTCCTCCGACATCTACGGGCCGCACACCAACGAGATCCTCCTCGGCAAGGCGCTGCTG

GGGGGCGTGAGGGAGAGGGTAGAGCTGGCCACCAAGTTCGGCATCTCGTTCGCCGACGGC

AAGCGGGAGGTTCGCGGGGATCCGGCCTACGTGCGGGCGGCGTGCGAGGGCAGCCTCGAG

CGGCTCGGCGTCAGCTGCATTGACCTTTACTACCAGCACCGCGTCGACACCAGGGTGCCT

ATCGAGGTCACGGTTGGAGAACTCAAGAAGCTTGTCGAAGAAGGAAAAATAAAATACATC

GGATTATCTGAAGCATCTGCATCAACAATCAGGAGGGCTCATGCTGTTCATCCTATCACT

GCAGTTCAGATGGAGTGGTCACTGTGGTCAAGAGATGTCGAAGAAGACATAATTCCAACT

TGCAGAGAACTTGGAATTGGAATTGTTGCTTACAGCCCACTTGGTAGAGGATTCTTCTCT

AAAGGAGCAAAATTGGTTGACTCACTATCAGACCAGGACTTCCGCAAGATGGTGCCATTG

CAAAGA

>Traes_5BL_151997828

CGACGATGCTGAGGCAGCCCCTCCCCCTCTTCTCGTTCTCCTCTTCTTCTTCTCCCCCTA

TTTATAGGGCGCTCGCGCGGCTCACCAGGCAGAATAGCCATTGTTTTGTGTTCATCTCGC

GTCCAGGAAGACAAGCTCAAGCTGTACAACGATCATGTCTTCGTCTTCCTCCTCCGTGAC

GTTCGAGAATCCGAGGAAGGTGGTGAAGAAGGTGCTGTCATTGTCCCAGTCCGAGGGGGA

CGGCGCCACCGTCCGCCGGAGCATCGGCCGGCACGAGCTCCGGAACCTGGACCCGTTCCT

CCTGCTCGACGAGTTCTCCGTCTCCAAGCCCGCCGGCTTCCCCGACCACCCTCACCGTGG

CTTCGAGACCGTCACCTACATGCTCGATGGGGCCTTCACCCACCAGGACTTCTCTGGCCG

CAAGGGCACCATCAGGACAGGAGATGTGCAGTGGATGACTGCGG

>Traes_2DL_1AF45DD78

CCGCCGTCCAGTTCTTCACCAACCAAATCTCATACCTACCTCCTCAGCTGCCTATTTAAT

CCCTCGCCTCCCTCCTTTCCCCTCCAAAGAGCCTCAGCTTCATCTGCAGCTGAGCTCCTC

TTCAGCACATACATCTGCCGGCGTCTAGCCAGCTTACTTTCAGAATCCAGATACACATAC

CCCTGCCGGCGTCCAGCTAGCTCACTTGCTTTAGTACCTCTCTGCATCTTTCGATGGAGT

GCGAGAATGCACACGTTGCCGCCAACGGCGATGGCTTGTGCGTGGCGCAGCCGGCGCGGG

CCGACCCACTGAACTGGGGGAAGGCGGCGGAGGAGCTCTCGGGGAGCCATTTGGATGCGG

TGAAGCGGATGGTGGAGGAGTACCGAAGGCCCGTGGTGACCATGGAGGGCGCCAGCCTGA

CCATCGCCATGGTCGCCGCGGTGGCTGCCAGCAGCGACACCAGGGTGGAGCTCGACGAGT

CCGCCCGCGGCCGCGTCAAGGAGAGCAGCGACTGGGTCATGAA

>Traes_4DS_805590E76

GCCATGGGTTACTCCAAAAACCTAGGGGCTGGCCTGTTTGCCATGCTGCTCCTTGCTCCG

GCCGTCCTGGCCTCCGACCCTGACCCTCTCCAGGACTTCTGCGTTGCCGACCTCGATGGC

AAGGCGGTCTCGGTGAACGGGCATTCATGCAAGCCCATGTCGGAGGCCGGCGACGACTTC

CTCTTCTCGTCCAAGCTGGCCAAGGCCGGCAACACGTCCACCCCGAATGGCTCGGCTGTG

ACGGAGCTCGACGTGGCCGAGTGGCCCGGTACGAACACGCTGGGTGTGTCCATGAACCGC

GTGGACTTCGCGCGGGGAGGCACCAACCCGCCTCACATCCACCCGCGCGGGACCGAGATC

GGCATGGTGATGAAAGGTGAGCTCCTCGTTGGAATCCTCGGCAGCCTCGACTCCGGAAAC

AAGCTCTACTCCAGGGTGGTGCGTGCTGGAGAGACGTTCCTCATCCCGCGCGGGCTCATG

CACTTCCAGTTCAACGTTGGTAAGACGGATGCCTACATGGTTGTCTCCTTCAACAGCCAG

AACCCCGGCATCGTCTTCGTGCCGCTCACGCTCTTCGGTTCCAACCCGCCAATCCCCACG

CCAGTGCTCACCAAGGCGCTAAGGGTGGAGGCCGGGGTCGTCGAACTTCTCAAGTCCAAG

TTCGCTGGTGGGTCTTAA

>Traes_2BL_5A50FDA1A

CTGGCACCAATTAGGCAAAGCTGCCTTCAGCAACCCGCTGTAGAATGCAGCAGCCAGCAG

CAGACCCCGCCTTCCAGCTCCACACCCCACACCAACCCACCCACCATGCCCCGCTCCTCT

CCCTATTTAACCCGTCTTCTGCCTCCATTCCTCTCCAAGAAGAGCCACAGCTTCTTCTCC

AATTTGAGCTCCTCTTCAGCGCCACTCCGACCTCCAGAATCCACCACCACTTCCAGAATT

CATCCAGAATCACAGATACGCATACACCTGCCCGGCTTCAAGCTTCCTTGCTTTAGCACT

TCTTTGCATATCTCGATGGAGTGCGAGAACGGGCACGTTGCCGCCAACGGCGATGGCCTG

TGCGTGGCGCAGCCGGCGCGGGCCGACCCGCTCAACTGGGGGAAGGCGGCGGAGGAGCTG

TCCGGTAGCCATTTGGATGCCGTGAAGCGGATGGTCGAGGAGTACCGTAAGCCGGCCTGA

CCATTGCCATGGTCGCCGCGGTGGCTGCCGGCAGCGACACCAGGGTGGAGCTCGACGAGT

CCGCTCGCGGCCGCGTCAAGGAGAGCAGCGACTGGGTCATGAACAGCATGATGAACGGCA

CCGACAGCTACGGTGTCACCACAGGCTTCGGCGCCACCTCTCACCGGAGGACCAAGGAGG

GCGGCGCTCTCCAGAGGGAGCTCATCCGATTCCTTAACGCTGGAGCCTTCGGCAACGGCA

GCGACGGCCACGTTCTGCCTGCTGCGGCGACGAGGGCAGCCATGCTCGTGCGTGTCAACA

CCCTGCTTCAGGGATATTCTGGCATCCGCTTCGAGATCCTCGAGACGATCGCCACGCTCC

TCAACGCCAACGTGACACCATGCCTACCGCTTAGGGGCACTATCACCGCGTCTGGTGACC

TGGTCCCGCTTTCCTATATTGCGGGACTGGTAACCGGCCGCCCAAACTCCATCGCCACGG

CTCCGGATGGCACCAAGGTTAACGCGGTGGAGGCATTTAAGATTGCTGGCATCCAGCATG

GCTTCTTCGAGTTGCAGCCCAAGGAAGGCCTTGCCATGGTGAACGGCACAGCAGTTGGCT

CAGGGCTTGCATCCATGGTCCTTTTCGAGGCTAACATCCTTAGCCTCCTTGCAGAGGTCC

TGTCGGCTGTCTTCTGTGAGGTCATGAACGGCAAGCCGGAGTACACCGACCACTTGACCC

ATAAGTTGAAGCACCACCCTGGGCAGATCGAGGCTGCCGCCATCATGGAGCACATACTTG

AAGGCAGCTCCTACATGATGCTTGCTAAGAAGCTCGGCGAGCTCGACCCATTGATGAAGC

CAAAGCAAGATAGATATGCACTCCGCACATCACCGCAGTGGCTCGGTCCTCAGATTGAGG

TCATCCGTGCTGCCACCAAGTCCATTGAGCGAGAGATCAACTCCGTCAACGACAACCCAC

TCATCGACGTCTCTCGCGACAAGGCTATCCATGGTGGAAACTTCCAGGGCACACCAATCG

GTGTGTCTATGGACAACACCAGGCTTGCCATTGCTGCGATTGGCAAGCTCATGTTTGCCC

AATTCTCAGAGTTGGTGAACGACTTCTACAACAACGGTCTTCCTTCCAACCTCTCTGGTG

GGCGCAACCCGAGCTTGGACTATGGCTTCAAGGGTGCCGAGATTGCCATGGCCTCGTACT

GCTCCGAGCTCCAGTTCTTGGGCAACCCTGTGACCAACCATGTCCAGAGCGCAGAGCAGC

ACAACCAAGATGTCAACTCTCTTGGCCTCATCTCCTCAAGGAAGACTGCCGAGGCCATTG

ACATACTGAAGCTCATGTCCTCGACATTCTTGGTCGCGTTGTGCCAGGCTATCGACCTCC

GCCACCTTGAGGAGAATGTCAAGAATGCCGTGAAGAGTTGTGTGAAGACTGTGGCTAGGA

AGACACTGAGCACCAACAACAATGGTCATCTCCACAATGCGCGCTTCTGTGAGAAGGACT

TGTTGCTCACAATCGACCGTGAGGCGGTGTTCGCTTACGCGGATGACCCCTGCAGCGCCA

ACTACCCACTGATGCAGAAGATGCGTGCAGTTCTCGTGGAGCATGCCTTGGCCAATGGTG

AGGCTGAGCGCGATGCGGAGACATCAGTGTTTGCTAAACTTGCCATATTTGAACAGGAGC

TCCGTGCAGTGCTTCCAAAGGAGGTCGAGGCTGCCCGAAGTGCCGTGGAGAACGGTACTG

CTACACAACAAAACCGTATCACCGAATGTCGATCATACCCTCTCTACCGATTCGTGCGCA

AGGAGCTTGGAACTGAGTATTTGACCGGGGAGAAGACTAGGTCTCCCGGTGAAGAGGTGG

ACAAGGTGTTCGTTGCTATGAACCAGGGCAAGCACATTGATGCACTACTTGAGTGTCTCA

AGGGGTGGAACGGTGAGCCCCTACCTATCTGCTGAGGATCAAGAACTTGAAGAGAAGATA

GAGTGCTTCAAAGTTCAGAAGGCTTCAGATATTCTTAGCTGATAAGTGATAATACTGTTT

TTTCATTGGATTTTCTTAGAAGCCGATGTTTTGTAATGTTATTACAAAGCTACTAGGTTG

TTGCCAAAAATTGCAATTGCATGCTTTGGTAGCGATAGGTAGCCAATAGAACTACTGTCA

TGTAAGTTAAAAAGGGTACAATATGTGATAAATATTCATGATAAATTTACTTGTCCCTTT

TTTGAGATCAATGGTCTAGTGGTCTTGAGACAACTTGGAACAATGCAAAGAATCTATTGT

GC

>Traes_1AL_BEB451473

CTTTGTTCTACTCCATATCACCAATGACTAATCTTCAGTTGGCACATCAATTCACTCGAT

TGCCACGAGCCCATGATTAACCGACCTGATCACCAAACATATAAAAAAGCTCCGAATTAT

AGACAACCTAACAGCAGCGCTGAACTCCCCGAACTCCGAAATGTCCACTCGTTTATAACC

AGTCGTCTTCAAGTCCAAGGCTCCAGCATCCAAGATCCCATCACCAGCACATCCCAAGAA

CCCAGAGATCCAAGCGCTGAGCCACGGCGTGTCAATGGCAGCGGCTGCAGGAGGAGACGA

GGTGAAGCTGCTAGGCGTGTGGGACAGCCCGTTCGTGAACAGGGTGCAGATCGTGCTCAA

CCTCAAGGGGATCAGCTACCAGTACGTCGAGGAAGACCTCCACAACAAGGGCGAGCTCCT

CCTCGCCTCCAACCCCGTGCACAAGAAGGTGCCCGTCCTCATCCACAACGGCAAGCCCAT

CCCGGAGTCGCAGGTCATCGTGCAGTACATCGACGAGGTCTGGNGTCCTCCCCGCCGGCC

CGCACGAGCGCGCCACCGCCCGGTTCTGGGCCGCCTACGTCGACGACAAGGTTGGGTCGC

CGTGGTTCACCATCCTGTTCGCCCGCAAGACCGAGGAGAAGATCGAGGCGGCTGTACGGG

CCATCTCGGCGATGGAGACGCTGGAAGGCGCGTTCGGGGAATGCTCCGGTGGGAAGCCGT

TCTTCGGCGGCGACGGCATCGGGTTCGTCGACGTTGTGCTCGGCAGCTACCTGGGCTGGT

TCGTGGTGATCGAGAAGATGATCGGGATCAAGCTCCTGGACGCGGCGAGGACGCCGGCCC

TGGCCGCGTGGGCTCAGCGGTTCAGGATGGCGGATGCGGTGAAGGGTGTCTTGCCTGAAG

ATGTCGACAAGGTGCTCGAGTTTTTGCAGACGTTCCTTGATTAGGTGCGTGGAAGCGCTG

TCAGCGGTTTGGACTTTCGATTATTAATTTTCGAGGAAATTTGGACTTTCGATTTGTTTC

TTTTCAAATGTGGGGTTGTAATGGGCCATCGCTTATTTCACGGCCTACAAAAAGGATTTA

ATAAAAGATGGCCTCAAATTATTAACTTTTAAACAAT

>Traes_1DL_269FE4712

CTTCGGCAATGGTTTACTGTTCGCACTGTGATGATGATTGCCCATATGTAAAGGATCCTG

ACAACGGGTTCACATGTTGTGGAATGTGCGGCAAGGTTATTGATCAGGATATGTATACTG

AGGAGCCTACTTTTTTCAAGGACTCCTCGGGACAGAGTCGGCTTCGTGGACACATTATAG

GCATTGCGAAGGGCGGTTCACTATCCCGTGAAAGAACTGAAGAGAAAGGGAGAGATGAGA

TCTGGCAGATTGTCCATGGGCTGCATGTGAGTGGTGGTGATGATATCATTTGCACTGCTC

ATAACTTTTATAAACTAGCTCTTGATAACTTTACTAGGGGCCGCCGAACAACTCATGTCG

CAGCTTCTTGCCTTTACATTGCTTGCCGGCGAAGTGAAAAACCCTACCTTCTTATTGATT

TCTCGGACTATTTGCATATAAGTGTATATGTCCTAGGTGCTGTTTTTCTGCAGCTTTGCC

AAGTTTTGCTACTCGGAGAACACCCAATTGTTCAAAAGCTTGTAGATCCTAGCCTTTTCA

TCCATCGTTTTGCCGAACGTTTATTGGGAAAAAGGGACAATGCTGTCTCGGACACAGCTT

TACGCATTGTAGCTAGCATGAAGCGAGACTGGATGCAGACTGGGAGGAAGCCAAGTGGTT

TATGTGGTGCAGCATTATATATAGCCGCGCTTTCTCATGGATATATGTACACCAAGGCGG

ATATTGCTGCTGTTGTGCATGTCTGTGAGGCAACACTATTTAAGCGCTTGATAGAGTTTG

AAAATACAGATTCGGGTAGCTTAACGATCGAAGATTTTTTGGCAAAGGCTGATGAAGAGC

CGGTTTCAAAATGTTTAGCCAAGTCTGGAGAAGTCCTTTGCGAGCACAAGGATAAGGGTG

CTGAGCATTTTTCTCATGGGCTTTGTGAGGAATGCTACGACAATTTCACTGAACTGTCAG

GTGGACTGGAGGGTGGTGCTGACCCTCCAGCTTTCCAGCGAGCTGAAAAACAAAGATTGG

ATGCTGCTAAAAGAGCCAAGGAAGCTGCTGTGGACGAGGCAATATGTGAGTTACATAATT

CTGATTTTGAAGATAACATCATGAGCCCTGGGAAGAAGTCTGAAGGCAAATCTTCCACAA

TTGCTTCCAGCCAAATTGCAAATGATTTTGTTGATTTCAAAGACTCGGAAGTGGAAGGTT

TGTTGGTTCCTGTAATATGATTATTGAAATAGGCTTTCGCTCCACTATAAATAAAGCAAC

CACCACGTACATACAAGAAGTGCAAAGTGCATGAAAGAATATAAGAGCTTTG

>Traes_7DS_D7F6AF9811

ATGACCACGAAGGAGCCGATCCCGCTGCTGACACCGCACAGGATGGGCCGGTTCGAGCTC

TCCCACCGGGTGGTCCTCGCGCCGCTCACGCGCTGCCGCTCCTACGGCAATGTGCCGCAG

CCGCACGCGGCGTTGCACTACTCGCAGCGGGCGACAAAGAGCGGCCTGCTCATCGCCGAG

GCCACCAGCGTCTCCGCCACCGCCCAGGGGTTTCCTGATACTCCTGGCATCTGGACGCAG

CAGCAGGTCAACGCTTGGAAACCCATCGTTTATGCCGTCCACAGCAAGGGCGCTCTGTTT

TTCTGCCAGATTTGGCACGCCGGAAGGGTCTCCTCAAAGCACCGACAACGCCGGCGAAGC

TCCGTTGCAGCACCGACAACGCTCCAATGCATCACCGATGGAGCTCCATTGCAGCCTAAC

GACCCGGCGA

>Traes_5BL_40DC18914

GCCCGCTCGCTTCATCGATCCATCGTCTTCTTCCTCTGCTCGCTCGCCAGCAACACTCCC

TTACACTCCATCGTAAGTGCACGTACTCACGGTAGATCGCATCAACAGCAAGAAAATACA

TCAACCGTACTTGGGGAACATCGTACGGTGACCATGGAGGTGAAGGTGGTCAGCTCGAAG

ATCGTGAAGCCGAGGTACGCCGAGGGCGCGGCGCGGCCGGACACCACGGAGCACGTGCCG

TCCTCGGTGTTCGACAAGATCACGTACCACATCCAGATGGCCATCATCTACGCATTCCAA

GCGCCGGCGCCCTCCACCGAGGACATAGAGCGCGGTCTCGCCCAGGTGCTGGCCGTGTAC

CGCCTCTTCGCTGGCCAGGTCCGAGCTGGCCCGGACGGCGCGCCCGGGGTGCTGCTCAAC

GACCACGGCGCGCGGCTCGTCGAGGCGCGTGTGGACGGGGCCACACTGGTCGAGTTCGCG

CCGCCCAAGCCGTCGCCCGTCGTGCTGCAGCTGCACCCGGACCTGGAGGGCGACGTGCAG

GAGGTGGTGCAGGTGCAGCTCACGCGGTTCGCGTGCGGCTCGCTGGCCGTCGGGTTCTCG

GCCAACCATGCCGTCGCTGACGGCCACGCCACCAGCGACTTCCTCGTCGCGTGGGGCCGC

GCCGCGCGAGGGCTAGACATCTCCGGCCCATCGCCGACGCCACCGCCGCACAACCATCCC

GATCTCTTCCCGCCGCGCGACCCGCCAGTCGTCAACTTCGAGCACCGCGGCGTCGAGTAC

TACCGGCCGTCGCCCAGCAACCCCAAGCAGGGCGAGGGCGGACACCACGGCGCCGACAAC

GTGGTCATCCACAAGGCGCACTTCACCAAGGACTTCATCGCCGGGCTGCGCGCCAAGGCT

TCGGAGGGGCGCGGCCGGCCGTTCAGCCGGTTTGAGACGACGCTCGCGCACCTGTGGCGC

AGCATGACGCGCGCGCGCGACCTGAGCCCCGAAGAGACCTCCACCATCCGCATCTCCGTG

GACGGACGGCGGCGCCTTTCCGCCCCGCCGGGCTACTTCGGCAACCTGGTACTTTGGGCG

TTCCCCCGGTCCACGGTGGGGGACCTGCTCAGCCGGCCGCTGAAGCACGCGGCGCAGACG

ATCCACGACGCGGTGGCCCGTCTCGATGGCGCCTACTTCCAGTCGCTGGTGGACTTCGCG

AGCTCGGGCGCCGTGGAGCGGGAGGGGCTGGAGAAGACGGCGGTGCTAAAGGACGTGCTA

TGCCCAGACCTGGAGGTGGACAGCTGGCTGACGTTCCCGTTCTACGATCTGGACTTCGGC

GCCGGCAGCCCGAGCTATTTCATGCCGTCCTACTTCCCCACGGAGGGGATGCTGTTCCTC

GTTCCGTCCTACCTCGGCGACGGCAGCGTCGACGCTTTCGTCCCCGTCTTCCAGCATAAC

CTCGAGGCCTTCAAGCAGTGCTGCTACTCCATGGATTAAAGATGAACTAATTAAGCTATA

TGATCATTGGAAATACACTTTGGAACCTGCATGTATATACACCCTATATGGGATTTTTTT

TTATTATTTTAATAAAAAGTCAAAATAGGTAAGAACTATTTTGACAAAACACTTGACTTA

CTTTTGC

>Traes_4DL_270AF9312

CCAAACCTGAAGATGAATCTAAAATTTGACATTGAGGTCCTGTTTAAGAACCTCAGTGTG

GACATGAAAGATGTGAAACCGACTTCCCTTCTTAAAGATCGAGGACGTGAAGTTGAGGGA

AACCCAGATTTTTCAAACAAAGATGTTGCTTCGTCCCAAACCCCAGTGGCTGCAGAAGTC

TCTTCAGGTGTCAACCCCCCAATAAAACATGTAGACCTGCAGCCTGAGGTCAATAGCACT

TCCCGTACCCTGAGCCTTCCAACTATATATACTGCTCCTGTTCGTCTCCCTCCTAACAGT

ATGGTGGAGGATGATAAAATTGCTCTAATGATGCCTGAGCAAGTCCCCTCTCACACCTTG

ACCCAGGTCTCATCTTCACAAACACCATTAGTATCACTATCGCCATCGCCACTCTCTCTA

ACTCAGCTTTTGTCGCTGATTCCACATGATGAGATACGCTTCAAAATAAGCTCAAAACTT

TTGCCTTTTGGCTCACAGTTGCAGTTCAGCAAAATCATGGGTGTGGCTTTGGATAAGGCT

ATCAAAGAGATAATACTCCCTGTCATTGAAAGAAGTGTCTCAATAGCAAGCAAAACTACA

AAAGAAATTATTCTAAAGGATTATGCAACGGAATCTGATTACAGTGCTGCAAATCGCTCG

GCTCGTTTGATGGTCGGAACGTTAGCTGGAAGTCTAGCCCATGTTACTTGCAAGGAACCG

CTTCGTGTTGCGCTACTGTCTCATCTCCGAAGCCTTACTCAAAACCTTGCTAGCAACAGC

GAAACTCTTGAGCAAATAGTACAGATTCTGATCAACGACAATTTGGATCTTGGCTGTGCA

AGTATTGAGTCTGTAGCGGCGCGCAAGGCTGTTGATTCGATTGAGGGTGAAATAACTCAA

TCCTTTTCACAACAAAGGAAGAAAAGAGAGGCAGCTGGTCCTACATATTATGACTCTTTT

GCTTATGCTCAAGGTCCATTTGCCCCTGTACCTGAAGTATTCCATACCAAGGCAGAGTCT

CCTGCCCAACAACGAGTATATGAGGAGTTTGTTCATGTATGGCAGAGTAGTCATGGCCAA

AGTGTTGGTGCTGCAAGTTCTGGTACAGCTGCTGTATCGAGCAATTTTGGTGTACCTCGA

GCTTACAGCCCAAGTTTAGCACAAGCTTCCAGTGGTTTCTTGTCTGCTCAAACAGCTCCA

CTTACATTAACTCAGCCTACAGAGCTGGTGTATGAGGAATTGATTCCTGGCGCTTCACAA

CTTTCTAGTGATTCTCCTGCTCAAGTTGGGACCAGCGACTCTTCTGGCTGGCTTGGTGGA

ACCATTGGCGATGCATCCACATCTCCACCCTTGATGTCTAATGACCTACCTGCGGGAGGA

ATAATGGATTTAAATGTTATGACGCCACCTCCAGCTACATTTCCTGACAATCTAGGATCT

GGTTTACCTGATACTTTGAGCACTGGTGATGCTATGGAGAGATACGAACAGGTTTCACAA

AAGGAATGTATATGCCTCCACTTTGTGCTTCGCCTACAGACTGCCCACACCACACCTAGT

CGTGCTTCTTTGTCACCTGTGCCATGCTTCATTTTTGTTGGAGACTCTTTAGTAGTTGGT

TGGGTATCCACTGTGTTTTAG

>Traes_6DL_CC87CFA38

GGGGTGGCGGCCGCGGCGGGGGCGGAGGAGGCCTACGTGACGCTGCTCTACGGGGACGAG

TTCGTCCTGGGCGTGCGCGTCCTCGGGAAGTCCATCCGCGATATGGGCACCCGCCGGGAC

CTTGTCGTGCTCGTCTCCGACGGCGTCTCCGACTACTCTCGGAAGCTCCTCGAGGCTGAC

GGTTTTATAGTGAAGCATATAGTATTGCTGGCGAATCCTAATCAAGTGAGGCCAACAAGG

TTTTGGGGTGTGTATACCAAATTGAAAATATTCAACATGACTACCTATAGAAAAGTTGTT

TATCTTGACGCAGACACCGTTGTAGTGAAAAGTATCGAGGATCTTTTCAACTGTGGAAAG

TTCTGTGCAAACTTGAAACATTCTGAGAGAATGAATTCTGGAGTAATGGTTGTTGAGCCA

TCTGAAACTCTTTTCAAGGATATGATGAGCAAAGTTGACAGCTTACCTTCTTACACGGGA

GGAGATCAAGGTTTTCTCAATTCTTATTATGCTGAGTTTGCCAATTCCCGTGTTTTTGAT

CCCAATAAACCTTTAACGCCTGAACCTGAGACGCAACGCCTCTCCACGTTGTACAATGCT

GATGTCGGTCTTTACATGCTTGCGAATAAGTGGATGGTCGATGAGAAAGAACTTAGGGTT

ATTCACTACACACTTGGACCTCTTAAGCCGTGGGACTGGTGGACAGCTTGGCTTGTTAAA

CCAGTAGCTGTATGGCAGGATGTTAGGCAAAATCTTGAAGAATCTCTTCCTGGAACTGGT

GGAGGGAAAAACCCTCATGACCAGTTGGTGGTCAAAATTCTCTTCATTCTTCCCTTTTGC

ATGCTGTTATGTGGTTATTATGGGTCATGCTTTCAGACTAATAAGGAGCTGTTGAGTATG

AGAACTCTGTGTGCTTTTGCTAGACAAGCTCGCTATAAATACAAATCTGAAGAGGCACTT

CCATCTTATTCAACAGTTGGAGTGGCTTCATCTTCCTTTGGTATATCAAATCAAAAGTTA

TCTACTGGAGCACATCTGAAGTTGCCTTCTTATTTTGGTGCAATCGCTGTGGTAGTCTGT

TTCATTTGTGCATTGATATCTCTTGCATTTGCCTTCCTTGTTATTCCACGGCAAGTGATG

CCATGGACGGGTTTGCTGCTGATGTTCGAGTGGACCTTTGTGACATTCTTTTTGTTGTTC

GGGGGCTACCTTCGTTTTGTATACAAATGGGGAAGTTTTAGTGCAAATCATGCTGGGTAT

GGCAGTTTGGATTCATCGGAGAATCATACTGGCACAGGCCATCAGGGGAATACGTCTGAT

TGTGACACAGCTTCAGCTTTCTATTGGATGGGGATGGCTACCATCTCTACGATAGCACCA

TTATCACCAACCGTCCTCGGCATAACTGCCATTTTTGCAAAACTTGGGTTGATGGTAGCG

GGTGGTGTGGTGCTGGCGTCATTTATGACATACGCTTCGGAGCATCTGGCTGTGTCTGCC

TTTGTCAAGGGTCAAAGAGGTAGATCGAAAATACCCGAGAGCTCTCGCAGCGAGTAA

>Traes_4BL_6C3CCAEED

CCAATCCCGGGCCAACCAACGAACCCAATCCAGTGACCTCGAGTCTTCACGGGAAGACAA

TAGAATTCTCCAGAAGTGCTTTCTCCTTCCCCACCGTATCATCCCCAGCCCTCTATAAAT

TCTTCCCCGTCCCGATCGACAGAAACCCAAGAAAGTCTGAACAAAGCACCGATCAAAGAA

CGATCCCTCCCAAATCAATCGCTCACCCTTCCGCTTCCTAACACCAGCTAAATCGTCCAT

TCCTTCGACCAGCAGCTCGAATCCCCACCGACAATGTCGCTGATCCGTCGCAGCAACGTG

TTCGACCCCTTCTCCCTCGACCTCTGGGACCCCTTCGACGGCTTCCCCTTCGGCTCCGAC

GGCAGCGGCAGCCTCGTCCCGCGCACCTCCTCTGACACGGTGGCCTTCGCCGGCGCGCGG

ATCGACTGGAAGGAGACGCCCGTGGCGCACGTGTTCAAGGCGGACGTGCCAGGGCTGAAG

AAGGAGGAGGTGAAGGTGGAGGTGGAGGACGGCAACATCCTCCAGATCAGCGGCGAGCGG

AACAAGGAGCAGGAGGAGAAGACCGACACGTGGCACCGCGTGGAGCGCAGCAGCGGCAAG

TTCCTGCGCAGGTTCAGGCTCCCGGAGAACGCCAAGGCGGAGCAGGTGAAGGCGTCCATG

GAGAACGGCGTGCTCACCGTCACCGTGCCCAAGGTGGAGGCCAAGAAGCCCGAGG

>Traes_1DS_26E6C748F

GTGGACGACATGGACTGGGAGGACATCTTCTACCTCCACGACGACAACCAGTGGCCCTCC

GACCCGCCGGCCTTCAAGGAGACCATGCGGGAGTACCGCGCCGAGCTCAAGAAGCTCGCG

GAGCGGGTCATGGAGGCCATGGACGAGAACCTCGGCCTGGACAAGGGCCGCATGAAGGCC

GCCTTCACTGGAGACGGCCTCCACGCGCCATTCTTCGGCACCAAGGTCAGCCACTACCCG

CCGTGCCCGCGCCCGGACCTCATCACCGGGCTCCGCGCGCACACCGACGCCGGCGGCGTC

ATCCTGCTGTTCCAGGACGACAAGGTAGGCGGCCTCGAGGTACTCAAGGACGGCGAGTGG

CTCGACGTGCAGCCGCTCCCCGACGCCATCGTCGTCAACACCGGCGACCAAGTGGAGGTG

CTCAGCAACGGCCGCTACCGCAGCGCGTGGCACCGCGTCCTGCCCATGCGCAACGGCAAC

CGCCGCTCCATCGCGTCCTTCTACAACCCGGCGTTCGAGGTGGCCATCTCGCCGGCGGTG

GGCGAAGGCGCCGCCGCCGCGTACCCGGACTACGTGTTCGGGGATTATATGGACGTGTAC

AACAAGCAGAAGTTCGAGGCCAAGGAGCCAAGATTCGAGGCCGTCAAGCAAATATGA

>Traes_7DS_72929F5D3

GGACGCAGCAGCAGGTCGACGCCTGGAAGCCCATCGTCGACGCCGTCCACCGCAAGGGCG

CTCTCTTCTTCTGTCAGCTTTCGCACGTCGGGAGGGTCTCCACGAATGATTTCCAGCCAG

ATGGACAGGCGCCGATCTCCAGCACCGACAAACAGATAACACCCGATGCTGAGTCCGGCA

TGGTTTACTCCAAGCCCCGGCAGTTTCAAACAGAGGAGATACCATTGATCGTCGATGACT

TCAGACGCGTTGCCCGGAATGCCATCGAGTCGGGGTTCGACGGTGTTGAGATCCACCGGG

CACATGGGTACCTATTGGAGCAGTTCATGAAAGACAGCTCTAACGATCGCACCGACGAGT

ATGGTGGAAACCTCGAGAACCGGTGCCGTTTTGCAGTGGAGGTAATTGATGCTATTGTCC

ATGAGATTGGTGCCGATCGCGTAGGAATCAGGTTGTCTCCATTTGTGGATTACATGGATT

GCTTCGACTCTGATCCACATGCACTCGGAATGTACATGGTGCAACAACTCAACAAACATC

AAGGGTTTGTCTATTGCCATATGGTAGAGCCGCGGATGGCCATTGTGGATGGCCGCAGTC

AGATACCCCATGGGCTCTTGCCCTTCAGGAAATCATTCAACGGCACTTTCATTGCCGCCG

GAGGGTATGATCGAGAGGAAGGCAACAAAGTGGTGGCCGACGGCTATGCTGATCTCGTTG

CTTATGGGAGGATCTTTTATCTTTCTGGCTAATCCAGATTTGCCTAAGAGATTCGAGCTC

GACTCACCCTTGAACAAGTACGACCGCAACACTTTCTACACGCAAGATCCTGTCATTGGC

TACACAGATTATCCTTTCCTTGAAGGATCGAATGGCGAGTAGTTCACCCAGTCGACTTGA

ATTAGTGATGTGTTTGATTTTATACAGTAGGTAATGATTTATTCAATGTGTGTATGCACG

GGCATGCGCATTGTATCATTTCCATCAATAATTTACCTTTGTTCTCCCATGTAATGCCAA

TATGTCAAGAT

>Traes_7DS_2BF9F77CA

CTAAGCACTAAAATCTACTGTTTCTTGCTTCTGGACTCCAGAAGCAAAAACAGTAGATTT

TAGCGATTCCTTTGGCATAGCATCCTATTGGCGCTACAAAATCTGCATCTAGTTCACACA

AGATGGTAGCGAGCAAGATGATGGTGGCGAAGGAGGCGATCCCACTGCTGACGCCGCACA

AGATGGGGCGGTTCGAGCTCTCACACCGGGTGGTGCTCGCGCCGCTCACGCGCTGCAGCT

CCTATGCCAACGTGCCACAGCCGCACGCCGCGGCGTACTACTCGCAGCGGGCCACCAGGG

CGGCCTCCTCATCGCCGAGGCCACCGGCGTCTCCGCCACCGCGCAGGGGTACCCGGAGAC

ACCCGGCATCTGGACGCAGCAGCAGGTCGACGCCGTCCACCGCAAGGGCGCTCTCTTCTT

CTGTCAGCTTTGGCACGTCGGGAGTGTCTCCACCAACGAGTCTGGTATGGGCAGGCGCCG

ATCTCCAGCACTGACAAGCAGATAACGCCTGATGCCGAGTCTGGTATGGTCTACTCCAAA

CCCCGACAGCTTCAAACGGACGAGATACCACTGATCGTTGATGACTTCAGACGCGCTGCC

AGAAACACAATCGAGGCGGGGTTCGATGGCGTGGAGATCCACGGGGCACACGGGTACCTA

TTGGAGCAGTTCATGAAAGACAACTCTAACGACCGCACCGACGAGTATGGTGGCAGCCTT

GAGAACCGATGCCGCTTTGCAGTGGAGGTAATTGATGGTATCGTCTATGAGATTGGTGCG

GATCGTGTAGGCATCATGTTGTCTCCATTCGTGGACTACATGGACTGCTTCGACTCCGAC

CCACATACACTCGGGATGTACATGGTACAACAACTTAACAAGCATCAAGGATTTGTCTAT

TGCCATATGGTAGAGCCGCGGATGGCCATTGTGGATGGCCGCAGGCAGATACCCCATGGG

CTCCTGCCCTTCAGGAAAGCATTCAAGGGCACTTTCATTGCCGCTGGAGGGTATGATCGG

GAGGAAGGCAACAAAGTGGTGGCCGACGGCTATGCTGATCTCGTTGCTTACGGGAGGATC

TTTCTGG

>Traes_4DL_DDF95FBC8

GGCTGGTGCTGCAGGGCGCCACCGACGCCGACCGCGAGTCCGTCCGCCGCGAGCTCTGCC

AGTTAGTTGACTATGGTCACGATGGATGTCTTTTGCTGCTCCAAGTCTGTTTGGATGAAG

TGCTGCTGAATGACAGGGAGGCCAAAAACTTGCAGTTGAAGCATGATCTTCTATCCGCCA

CTTTCAGATATTGTCTGGATAAAACGTATTTTAGCACTTGTTTCTGTGAGGCACTGATGC

GGATAAAAACTGGTACTGATGGCCTCCTTGAGACTCTGTCGAGTGCACTGGAACTCTCAG

CAGCCGAGAAAGTCGGTATTGGTCTTGCGCTGTCAGATTCTGATAATTCAGGCATGAAGC

TGAAAGGGCAACAATTTGCAATTGCTCAAATTGAGGAGTTGTGTTTGAATCCTAATCAAT

CGGTACCAAATGATCAAATTCATGAAATCGTCGTCTTCCTTCACCAGACCGATGGCCTGG

CAAAGCATATGGATACTTTTAGTAACATTGCTTCTCTTCTAGAAGTTGGACAGAGTCCTT

TTTTTGCTCCCATCCCCAAAGAGCAGCATGATGTTCAGTCAATAAATCATTCAAGACATT

TGGAGATGTACTTGGATAGCACAAATGATGACTTTGAGTCACTTCTTTCTGAAATTGGGA

AAGAAATAAGTATGGCGGACATAGTAACTGAGTTGGGTTATGGATGCACTGTTGACAGCA

CACAGTGTAAGGAAATACTTTCCACTTTTCAGCCTCTTGATGATGTGGCAATATCTAAGT

TGCTCGGGGCAGTTATTGGCACTCAAAATAGTCTTGCAGAGGCCCATAACACTTATGCAA

CCTTTGTATCTGCTATTCGAAATACCCACTTGAGTGAGTCACCTCAATTGACTACATGGA

ACACAGATGTTCTTGTGGATTCAATCAACGAATTGGCCCCAAGTACTAATTGGGTGCACG

TTATGGAAAGCCTTGATCATGAGGGTTTCAGTATTCCTGATGAAGCAGCTTTCTGTTTAC

TGATGAATATATATGGCCGCGCTTGCAAGGATCCCTTTCCGCTTCATGCTGTTTGTGGGT

CAATGTGGACAAATACAGAAGGCCAGATATCATTTTTAAAGCATGCAATTTCTGCCCCTG

CTACTATTTTTACATTTGCACATAGTTCGAGGCTGCTGGCACTTCCGGACTTTGCAAGTC

TTGGTCCAGGTAATCATGCTTGGTTTTGCCTTGACCTTCTAGAGGTTTTATGCCAGCTTG

CTGAAGTTGGACACACTGTGTCGGTGCGGTCCATGCTTGATTATCCTTTGGGACATTGCC

CTGAACTCCTACTTGTTGGTCTTGGACACATCAACACTGCCTATAATCTCCTCCAATTTG

AAGTGCTGTCCTGTGCATTTCCTGCTATTCTGAAGGACGCCACAAAGAGAAATGTGGTGA

ACTATCTCTGGCATATTAACCCATGCCTCACCCTTCGAGGATTTGTTGATGCTCATTCTG

ATCCAAGCTGTCTCCTGAGAATTGTGGATGTGTGTCACGACCTTAAGATCCTATCTGCTG

TCCTCGAGTCCACTCCATTCGCATTTAGCATTAGATTGGCGGCTGCTGCTTGTAGGAAAG

ATCATAGCCATCTTGAGAAATGGCTCACTGAGAAGTTAACTGTATACAAGGACGGTTTTG

TCGAGGAATGCGTTAATTTCCTGAAAGAAGCTATGAACGCCGCATCTTATGTGGTAGAGG

GTACCACGGAACAACCTCAAGCTAGTGTTGTGGACACGTATTGGGAAGCTTGTCCTCCCT

TTATAAAGGTTCTCCAGTCTCACTTGGGACAGCTCTTATCCAACCAACTCTCGGATGAAC

TAAGGGAGTTGTGTGCTCTGTATGAATCGAGAAATCAGGGTCCTGTAGCAAGGGACATAC

CTACCTCGGAGGGAGGTTCAGATGATGTTGAAGTAGAAGCAAATGCGTATTTTCACCAGA

TGTTTTCTG

>Traes_1DL_281C17822

CGCCGCCTAGATGTGTCACCTGACTTGCCACCGTCCTCCACTCTCGTCTCAGATTTGACC

CCAAAGTCAAGCGGCAAGCAGCAGCAGCAGGTTCCCGTGTCGCCATGAGCTGGATGGGGA

AGCGGGGGTTGGGTGGGGAGAGCCCGCACTGTCGGAGAAGACGAACGAGGGCTGGCGGCC

CCGGAGCTACCTCCACTACCTCGCCGCCGCATCCAGTCCGACCCTGCCTCCTGCAAGCAC

GGCGACTTCTTCGACACCGTCTGGGCGGGCCACGAGCTAACCACGCCCAATCTCTTCAGC

AATGTAGATGTCGACCGTAGTTGGATGTACAAAGAGAGACGAGGGAAGTTGATCAGTGAT

GCTTGGCAACATGGAGTAGATAATTTCCTAGATCATGCATTCTCATTGCCCGATGCTGCT

GTAGATGGGAAATCCCATTGTCCTTGCAGCAAGTGCTTTTGCGGTCATAAGTGGACAAGG

GATGTCATGACCACACACTTGTGCAGCAATGGGTTCATGTTAGGATATGAGAGATGGACT

AGCCACGGGGAGTCTGATGCACCTGAAAATGTAGAGCATGATAACGTGGGGGATGGAGAT

AGAATGAATGACATGCTAGTTGATGCAATTGTTGCTGAAGGAGTTTCTAAAGGTGATGAG

CCAACAAAATCTGCCAAATAATTCTATGAAATGTTGATGGAAGCCGACAAACGTTTGCAT

GAGAAGACCACACAATCACGTCTATCCATTGTAGGAAGGCTAATGACTATTAAAACACAA

CATAATCTGTCAGAAGCTTGTTACAATGAGATGATGACTCTCATACATGACATTGTTGGG

GACGATGCTGCAAAAGATCTCCCAGCAAACTTCCATAGGTCCATGAAGCTTGTGCATAGT

CTAGCAATGCCTTACGTCAAAATTTATGCATGCCCCAATAATTGTATGATTTATTATAAA

GAGAATGAAAATAAAGAAAAATGCACTATCTGCAAAGAACCTAGATATGAGGAAACAACC

GCAGGAAATAAATCTACGAAGATACCAAGAAAAGTTTTGCGCTATCTCCCAATTACTCCT

AGGCTACAACGGTTGTACATGTCACAAAGCACTGCCAAGCACATGGACTATCACGCAAGG

CCTCATAACAGCGATGAAGTAATGGTTCACCCTTCTCATGGTGAAGCTTGGAAGGAATTT

AATAAGGAATTTAAAAAATTTGCTGAGGAAGTAAGGAATATAAGGCTATGTCTAGCGACT

GACGGCTTCACACCTTTTGAAATTAAAGCAGCCTCCTACTCATGTTGGCCAGTATTTGTT

GTACCTTATAATCTTCCACCAGAAATGTGCATGAAGCAGAGTAACCTGATCCTTGCACTA

GTTATTCCCGGTCCAGATCATCCAGGAAAGAACCTAAACGTTTTTATGCAGCCTCTAGTT

GACGAACTTGATGATTTGTGGAGAAATGGTGTAGAAACTTATGATAGTTATCGAAAGCAA

AATTTCACCTTGAAGGCAGGTTTGCTTTGGACTATTCATGACTTTCCAGCTTATGGATTA

GTAGCATGCTAGAGCACTCATGGAAAGCTTGCTTGCCCTATATGTGGATCTGACATAAAG

ACATTCTCATTGAAGAATGGGAGAAAGCCATGCTGGTTTGATTGCCATAGGAGATTCCTT

CCCACTGACCATGCATTTCGTAGGAGTGTGAAATGTTTTCGCAAGAAAAAAACAGTTTTA

GATCCTCCACCAAAGCCCTTGTCCGGAGAAGAGGTGTATGAAGAACTCCAAAGTCTTGTC

CATGATGAAACTGGCAAGAGTAAATTTGAGGGGTTTGGGAAGGAGCACAATTGGATAGCT

ATAAGTGGTTTGTGGCAGCTAGAGTATTTTCGGAAGTTGCTGCTTCGCCATAACATTGAT

GTAATGCACAATGAGAAAAATGTATCTGAGGCTTTACTAAACACATGCCTTGATATTCCT

GAGAAAACTAAGGATAACAACAAAGCACGCCTTGATGTTGCTTTGTATTGTGATTGACCA

AAGCTACACCTGAACAAGAATTCAAGGGGTGTGTGGAAGAAACCTAGAGCGAAATATTGT

GTCAGCAAGGAAGATAGAATGACCATCCTTAAGTGGTTTAAAGAAGTGAAGTTTTCTGAT

GGGTTTGCTGCTAATTTGAGTAAAACTGTCAACTTGAGTAAAAACAAATTCATTGGGCTA

AAAAGCCATGACCACCACATCATCATGGAGCGCCTCCTGCCAGTTGCCCTACGAGGCTTC

ATCCCAGAACCTGAATGGAAGGTCATAGCAGAGTTAAGTTTTTTTTATCGGCAATTGTGT

GCCAAAGAAATTGCTCCAAAACGGATGCGTGAACTTGAGGAGGAGGTTCCTGTTCTATTA

TGCAAGCTTGAGAAAATGTTCCCACCAGGCTTCTTCAATGTAATGCAACACCTGATAGTG

CATCTTCCATATGAGGCAAGGGTTGGCGGCCCTGTGGCATATCGTTGGATGTATGTTTTT

GAGAGGGCAATGCACTATCTTCGTTTAAAAGTGCGAAACAAGGCAAGAGTGGAGGGTTCA

ATCGTTGAAGCTTGTATCGTGCAGGAGATCACGAATTGTGTTTCTCTTTACTTTAGTGAT

CGCGTTCGCACTATATGGAAGAAGAATCCTCGATATAATAATGGAGGAACACGCGTGCAG

AATGATGGTTGCACCTTAGATGTGTTTCAGCATGTAGGAAACCTACATGGGAGGCCTATT

GCCCGAGAACTATCGCGTGACGAGTTGAATGCAGCAAGGCTGTACATTTTGACTAACTGC

TCTGCTGTTGATAGATTTCGTGAAACATTTGAAGATGAAAAATATGCAAGTCATCCAAAC

TTGACATCGGAGGGCCTTGATGAAATGATGGCATCCGAATTTGTGGAATGGTTCGAGATT

GCTTGCAAAGAAGATCCTAATTCTGATGAGGACTTGTGGAATTTAGCTAATGGATGCAGC

TCTAGAGCCTATTCCTATAGCTCCTATGATGTGAATGGCTTTCGCTTTAGATCAGAGATA

TCTGAAAAAAAACGTAGAAGGTTGAAAACGGTGAACACAGGGGTTTGTTTGTCATCCACC

GCCAATTGGTCCAAAAAATGATCAGAAACCGGTATTGTGTTGGGAGGACTACAAGTGGTC

CCCTGGTCCAGAAGTAAGAACAGCAGCTTCCAAAATAGTGGAAGAATTTTG

>Traes_5BL_DDAC16D2C

ATGGCTTCTGAGGAAACCAAACCAAAGAAGCAAAGAGATGAGGAATGCATTATAAACGGT

CTCCCAGGAGAACTCATTGAGCGGATATTTTTGAAGCTTCCAGTGAGCACTTTGTTGAGG

TGCACTGGTGTTTGCAAGCAGTGGCACAAAATCATCCGAGATCCTCAGTTTGTCACCTCT

CACCTCCAGGATGCGCCCCAATGTGCCTTCCTATTCTTTCCACAAGAGTCGGTCTCAGGT

GAACCCCATCCTGCTGATGCTATCCTGATTGATGAAGCCTTGTCGCCATCGACATATGCA

GTGCCAGTGATTGGGCCTGACGATTTCCTTTGCGGTTCATGCAATGGGCTTCTTGTCTTA

TACACAAAGTCATCAACACTCAAGGTAGCTAACTTTGCAACTGGTGAATGTCTGCATCTT

GAGAAACCTGTAAAGAATTTGAGGGGTGATCACTTGTTCTACAACTTTGGATTTCACCCA

TTGACAAAAGAATACAAGATTACACACTTCCTTGGTGATCTTGTTGTGGGCAGCACTCGC

TCCCATAATAATAGCAAATTCAGCGTCATTCAAGTTTACACACTTGGTGATGAGAAATGG

AGAGATATCAAAACTCCAGAAGCCCTAAGCTTAAACTGTGTAAAAAACTCTGGAGCAGTC

AATATTGACGGAACAATGTGTTGGCTAATTGAAGACATGGTAGCTAACTGGCAGCATGCA

GTTATGACCTTTGATCTCAATGAAGAAAGTTTTGCACGGATACAACTGCCAGCAACTGTA

CATGAAGATTGTGCAGGTGGCGGTCCCCGTCGGCACTGGATCAGAGAGATAGATAGGAAG

ATATGTATAGCAACTGCTCAAGCCTGTCCTTCTCTTCCCAGAAGGCTTGTTGGTACGCTG

CAGATCTGGGAACTTGAGAACAAAACGGAGCAAAGGTGGAGCCTGAAGTACAATATTCAG

TACTCGCCAGATTACATTCCGGGTCCAAATTTGGTTCATAGGAATAAGATCATACTGCAA

CGTCGCGACAGCAACCTATATTCCTATGAGTTGCTCGGGGAGAACTTCAATACTAAATTG

TGTAAGATGGCAAAGCTGTTAGATTTC

>Traes_5BL_6FBA7CC33

CCTCAACGATCACTCGATCGCCTTCTTCCTCCATCTTACGTGCACTGCCGGTATCTAGAA

TTGTAGATCGAGCACTCAGGACATCAACAACAAAACCCAAAGGAGGAGTAAACTACCTTG

CTAGCTAGCCGGCCGGTGATCATGGAGGTGAAGGTGGTGAGCTCCAAGATCGTGAAGCCG

CGGTACGCCGAGGGCGCGCCGCGGCCGGACACCACGGAGCACGTCCCGTCCTCAGTGTTC

GACAAGATCACGTACCACATCCAGATGGCCATCATCTACGCCTTCCAGGCGCCGGCGCCC

TCCACGGAAGACATCGAGCGCGGCCTCGCCCAAGTGCTGGCCGTGTACCGCCTCTTCGCC

GGCCAGGTCTGTGCCGGCCCGGATGGCGCGCCGGGGGTGCTGCTCAATGATCACGGCGCG

CGGCTCGTGGAGGCGCGGGTAGAAGGGGCCACACTGGTCGAGTTCGCACCGCCCAAGCCG

TCGCCCGTCGTGCTGCAGCTCCACCCGGACCTGGAGGGCGACGTGGAGGAGGTGGTGCAG

GTGCAGCTCACGCACTTCGCCTGCGGCTCGCTCGCCGTCGGGTTCACGGCCAACCATGCC

GTTGCTGACGGACATGCCACCAGCGACTTCCTCGTCGCGTGGGGCCGCGCCGCGCGAGGG

CTGGACATCTCCGGCCCGTCGCCGACTCCGCCGCCGCACAACCACCCCGATCTCTTCCGG

TCGCGCGACCCGCCAGTCGTCAACTTCGAGCACCGCGGCGTCGAGTACTACCGGCCGTCG

CCCAGCAACCCCAAGCAGGGCGAGGGCGGACACCACGGCGCCGACAACGTGGTCATCCAC

AAGGTGCACTTCACCAAGGACTTCATCGCCGGGCTGCGCGCCAAGGCTTCGGAGGGGCGC

GGCAGGCCGTTCAGCCGGTTTGAGACAACGCTCGCGCACCTGTGGCGGACCATGACGCGC

GCGCGTGGGCTCAGCCCCGAGGAGACCTCCACCATCCGCATCTCCGTGGACGGGCGGCGG

CGCCTTTCAGCCCCACCGGGGTACTTCGGCAACCTGGTCCTGTGGGCGTTCCCGCGCACC

ACGGTGGGGGACCTGCTGAGCCGGCCGCTGAAGCACGCGGCGCAGACGATCCACGACGCG

GTGGCCCGCCTCGACGGTGCATACTTCCAGTCGCTGGTCGACTTCGCCAGCTCGGGCGCC

GTGGAGCGGGAGGGGCTGGAGAAGACGGCGGTGCTGAAGGACGTGCTGTGCCCGGACCTG

GAGGTGGACAGCTGGCTTACGTTCCCGTTCTACGAGCTGGACTTCGGCGCCGGCAGCCCG

AGCTACTTCATGCCGTCCTACTTCCCCACGGAGGGGATGCTGTTTCTGGTGCCGTCCCAC

CTCGGCGACGGCAGCGTCGACGCCTTCGTCCCCGTCTTCCAGCACAATCTCGACGCCTTC

AAGCAGTGCTGCTACTCCATGGACTAAGATGGCTAGCGTGCGGCTACATATGATTATTGG

GTGATTAAATCAGCGATTTGAGCTTAATCATATCGCCTGCAACAGCGTGCGTACGTGCAT

GCATGCATCCAACTATTATCCTCGCTTATCTCCGTGTATCTATCGTATCACGTATATAAG

TATGTATTTCAGTTTTGCTGTACATGCATTTGTTGTTTTCCGTAATGCACGGTATATATG

TCATGTACTTTTGCTTCGTCCGGT

>Traes_5BL_D02A5BA15

GAAAGAACAACCTCGATCTCTTGTTGCCAACTTTTGATCTTTCTCGCCGACGGATCAAAC

TTGATTAGCCACAGAGAAACTACTTCTCCGGCCTAAGCTCAGTACACAGCAATGGAGGTC

AAGGTGTTGAGCTCCAGGCTCGTCAAGCCTGCCTACACTGCCGGCGAAGCGCCGATGCCG

GCCACCGAGTACATTCCGCTGTCCATGTTCGACAGGGTGACGTTCGAGATGCAGATGGCC

ATCATCTACGCCTTCGCGCCACCCGCGCCCGCCACGGCCTCCATCGAGAAGGGCCTTGCC

ACGGTCCTCGCCCAGTATCGCGCCTTCGCCGGGCAGCTCGGCGAGTCCCCCGACGGCACG

CCGTCAGTCATACTCAACGACCGTGGCGCGCGCCTAATTGAGGCGTCCGTGGACGCCGAC

CTCGTCGACATGGCGCCCTCGAAGCCCACGCCTGAGCTGCTGAAGCTACATCCTGACCTG

GAGGCGGAGCACCAGGAGGTCGTGCTGCTGCAGCTGACACGGTTCCGGTGTGGCTCGCTC

GCTGTAGGGTTCACCTCCAACCACGTCGTCGCCGACGGCCACGCCACCAGCAACTTCCTC

GTGGCCTGGGGGCGCGCCACGAGAGGGCTCCCAATGGGCCTTCCGCCCGTGCACCACCAC

AAGGACCTCTTCAAGCCACGGTCGTCGCCTCGCGTGGAGCACGACCACCGTAACAGGGAG

TACTACCTGCCGTCGCCCACCGACGTGGTCGGTCACCACGGCGACGCCGCCGACAACATC

GTCATCCACAAGGCGCACTTCACCAAGGACTTCATCGCTGGTCTCCGCGCCAAGGCGTCA

GAAGGGCGCGGGCGGCCGTTCAGCCGGTTCGAGACCATCCTCGCCCACCTGTGGCGCACC

ATGACGCGCGCGCGCGACCTGGGCCCCGAGGAGACCTCCAAGATCCGGCTGTCCGTGGAC

GGCCGGCACCGGCTCGGCCAGCCGGCGGAGTACTTCGGCAACATGGTCCTGTGGGCGTTC

CCGCGCTCCACGGTGGGCGACCTCCTGAACCGGCCGCTGAAGCACGCCGCCCAGGTGATC

CACGACGACGTGGCGAGGGTGGACGGTGGCTACTTCCAATCTTTTGTCGACTTCGGGAGC

TCCGGCGCCGCCGAGAAGGAAGGGCTCGCGCGGAGCGCCGTGTGCAAGGACGCGCACTGC

CCGGACGTGGAGGTGGACAGCTGGCTGACGTTCCCATTCTACGAGCTGGACTTCGGTACG

GGGAGCCCGAGCTACTTCATGCCGGCCTACTTCCCCACGGAGGGGATGCTTTTCCTCGCG

CCGTCCAACTTCGGCGACGGCAGCGTCGATGCCTTCGTACCCCTATTCCAAGAGAACCTC

CAGGCGTTCAAAGAATGCTGCTACTCCATGGAATAGTAGGTGGCCAAACTTGAATTAGTC

CAAGGAACAGGGAGTGAGGCATACATTAGTTCATTTATTGAGAGTGTGGCATAAAAAATA

TGTGTTCTCTTTTTTGTTTTTGAGAATGTTATTGTGCTACTGTATATACACAGATGTTGT

TGTGTACAGTTAGCACGAATTTTCGGAGCAAACAAATTACATTAT

>Traes_2AS_AFC450E36

CCACGACTTCGTCCCCAGGGTGCAGCCGCACTTCCGCAAATGGATCCCCATCCACGGGCG

CACGTTCTTGTACTGGTTCGGAGCCAGGCCGACCCTGTGCGTGGCCGACGTGAACACGGT

GAAGCAGGTGCTCTCCGACCGCAGCGGGATGTACCCCAAGAACATCGGCAACCCGCACAT

CGCCCGGCTGCTCGGCAAGGGGCTCGTGCTCACCAACGGCGACGACTGGAAGCGCCACCG

CAAGGTCGTCCACCCTGCCTTCAACATGGACAAGCTCAAGGTGCGTGATGTGTGTTTTGT

TTTTTAACTGTACTATTCATTGCGCTGACAATTATCCAGTTAATGCACCGTGCCATAAAA

AAGGAAACTAGTAATGTAGATTATTTTCTTTCCTAAACCTAAAAGACACGTTATTCACGA

GATCAAGGGTATTATATTAGTTTACCTTTTCCAAAACAAAAAATACGCTGAAATCGGTGC

CTTTTCAAAAGCAAAATACTAGTAACTTATTGCCGTACTAGTAGAATGCCCGTGCGTTGC

TACGGGCTATAATGTATATAAATGAATCAAATAAATGATTAAGGTC

>Traes_4AL_BCAF03F9B

GTCGTGTTCCTCTTGCGTCCAGAAAGACAAGCTCAAGCTCTCTAGAAGTAGCGCCAACAA

CACCAGACCTTTTCTGTGCCTGGCCCTCTTCCTCCTCCTGGTTCTTGGTCTGACGGTCGT

CTTCTTGTTCCCACCAGCGATCATGTCTTCCTCTTCCTCGTCCGTCTCGTTCCAGACTCC

GAGGAAGGTGGTGAAGAAGGTGCTCTCGTTGTCCCAGTCCGAGGCGGACGGCGCCACCGT

CCGCCGGAGCATCGGCCGGGGGCCTTCACCCACCAGGACTTCTCGGGGCGCAAGGGCACC

ATCAGGACCGGAGATGTGCAGTGGATGACGGCTGGCCGCGGCATCGTGCACTCGGAGATG

CCGGCATCTGACGGCGTGCAGAAGGGCCTGCAGCTCTGGATCAACCTCGCCTCCAAGGAC

AAGATGATCAAGCCGCGGTACCAGGAGCTCGAGAGCAAGGACATCAGCCAGGCCGAGAAG

GACGGTGTGAAGGTGCGGATCATCGCCGGGGAGGCCTTTGGGGTGCGGTCGCCGGTCTAC

ACACGGACGCCAACCATGTACATGGACTTCACAATGCAACCAGGGTCGCAACTCCACCAG

CCAATCCCCAAGGGCTGGAACGCTTTCGTGTACATCATCAAAGGGGAGGGTGTATTTGGC

AGGGAGGCCGCGGCGTCGGCGAGCGCCCACCATTGCCTTGTGCTCGGCACTGGCGATGGG

CTCAGTGTGTGGAACCGGTCCGGTGCGCGGCTGCGGTTCATGCTGGCGGCAGGTCAGCCC

TTGAACGAGCTAGTGGTGCAGCAGGGGCCCTTCGTCATGAACTCTCGTGCCCAGATCCAG

AAGGCCATGGAGGACTATTACTACGGCCGCAACGGCTTCGAGAAGGCCAGCCAGTGGAGC

TCCACTTGAGTCTGATCAGTTTGTCGATGTCATGATGATTAACGTCGCGGTGCATTGTCG

CGCAAAAAATTGGGGAGGAAAGGGAAATTTCATTGTACCATTTTATTGATGCCTTTGTGT

AGAGTGCAGTAGTAGTCTTGTTTTTCTTTCATTCGTTGTACCATAGAAGAATGTAATACA

ATAGAAGTGTGTGATGAGGAGGAAGTAAGTAATTTGTTTCATCATAAGAAAACAAATTGT

TTCATCATATGTTTGGGCAAGCAGAGCAAGG

>Traes_7DL_B86B36F67

GTCAAGGCCAGCAGGGTCTCCAAGCTCTACGTCCACGCCGATCCCTCCGACCTCAGCCTC

GTGGTGAAGGATGGGTACCAATGGCGGAAGTACGGGCAGAAGGTGACCAAGGACAACCCG

TGCCCGAGGGCCTACTTCCGGTGCTCGTTCGCGCCGTCGTGCCCGGTGAAGAAGAAGGTG

CAGCGCAGCGCCGAGGACAAGACCGTGCTTGTGGCCACGTACGACGGCGACCACAACCAC

GCGCCGCCGCCCAAGCAACAAGGCTCTGGTGGCAGGAAGAGCGGCNNNNNNNNNNNNNNN

NNNNCGCCGGCGCTCGTCCAGCAGCAGCAGAAGCAGGAAGCTTCGACGGCGGAGCAGGTG

GCCGATAGGAAGAACCTGGTGGAGCAGATGGCGGCGACGCTGACGAGGGACCCCGGGTTC

AAGGCGGCACTCGCCTCCGCGCTCTCTGGCCGGATCCCTGTTGCTTGAGCGGAAACCTGC

CAGCACAGGAGAATACTCTACTGGAAACCTGCCAGCATCGAGTTCTTGTCGATGTAAGCC

GAGTACAGCTCTCGGTAATTAATCAGCAGGACACGACAGATGCGTAGCCGAGAGTTAAGC

CGCAGGTGCTCGACAAAGATCAAAAAACATGGCTTGACAGATGTTTGCCGAGAGCTAACA

TCCAGTTACACGGCAATAGGATGTGCACCAGTGGCACGTATGAATGATCATTCAATCCAA

CATGAGAATTGTTGACAGCTAACAGTAGTTTGCTTA

>Traes_4BL_CDACE3D5D

GTGCATATCTTTGTCAGGCCACTGACTGACAGGATCATGACTATTGATGTTGACAGAGAG

GATACCATAAGCAGTGTGAAGGCAATGATTTTCGAGAAAAACGGCATTCCCCCCCGCAGA

CAGCGTCTCTGCTTTGCAGGGAAGCAGCTGGAGGATGGCCTCACCTTGGCTGACTACGAC

GTGTACAATGAATCTACCCTTTTTCTTGTGGTGCGCCTCCACAAGTGTGA

>Traes_2DL_10A4DDD75

CATGGAGATGGCTTGTGCGTGGCACAGCCGGCGCGGGCTGACCCGCTCAACTGGGGGAAG

GCGGCGGAGGAGCTCTCGGGGAGCCATTTGGAGGCGGTGAAGCGGATGGTAGAAGAGTAC

CGTAAGCCGGTGGTGACCATGGAGGGCGCCAGCCTAACCATCGCGATGGTCGCCGCGGTG

GCTGCCGGCAGCGACACCAGGGTCGAGCTCGACGAGTCCGCCCGCGGCCGCGTCAAGGAG

AGCAGCGACTGGGTCATGAACAGCATGATGAACGGCACCGACAGCTACGGTGTCACCACC

GGCTTCGGCGCCACCTCTCACCGGAGGACCAAGGAGGGCGGCGCTCTCCAGAGGGAGCTC

ATCCGATTCCTTAACGCTGGAGCCTTCGGTACCGGCACTGACGGCCACGTTCTGCCTGCC

GCGGCGACAAGGGCGGCGATGCTCGTCCGAGTCAATACCCTGCTCCAGGGATATTCAGGC

ATCCGCTTCGAGATCCTGGAGACGATCGCCACACTTCTCAACGCCAACGTGACACCATGC

CTACCGCTTCGGGGCACGATCACCGCGTCGGGTGACCTCGTCCCGCTTTCCTACATCGCC

GGCCTGGTCACCGGCCGCCCAAACTCCATGGCGACGGCTCCGGATGGTTCGAAGGTTAAT

GCTGCGGAGGCATTTAAGATCGCTGGCATCCAGCACGGCTTCTTCGAGCTACAGCCCAAG

GAAGGCCTTGCCATGGTGAATGGCACGGCAGTGGGCTCAGGCCTTGCATCCATGGTGCTT

TTTGAGGCAAACGTCCTTAGCCTCCTTGCTGAGGTCTTGTCGGCCGTCTTCTGTGAGGTC

ATGAACGGCAAACCGGAGTTCACCGACCACTTGACCCACAAGTTGAAGCACCACCCCGGG

CAAATTGAGGCTGCTGCCATCATGGAGCACATCCTTGAAGGCAGCTCCTACATGATGCTC

GCAAAGAAGCTCGGTGAGCTTGACCCACTAATGAAGCCAAAGCAAGATAGATATGCACTC

CGCACGTCGCCGCAGTGGCTTGGCCCTCAGATTGAGGTCATCCGTGCTGCCACCAAGTCA

ATCGAGCGGGAGATCAATTCCGTCAACGACAATCCACTCATCGATGTCTCGCGCGGCAAA

GCTATCCATGGTGGCAACTTCCAGGGCACGCCCATCGGTGTGTCCATGGACAACACCAGG

CTTGCCATTGCTGCAATCGGCAAGCTCATGTTTGCCCAGTTCTCGGAGCTGGTGAACGAC

TTCTACAACAACGGTCTGCCTTCCAACCTCTCCGGCGGGCGCAACCCAAGCTTGGACTAT

GGCTTCAAGGGTGCCGAGATTGCCATGGCCTCGTACTGCTCCGAGCTCCAATTCTTGGGC

AACCCTGTGACCAACCATGTCCAGAGCGCGGAGCAACACAACCAAGATGTCAACTCTCTT

GGTCTCATCTCCTCAAGGAAGACCGCAGAGGCCATTGACATATTGAAGCTCATGTCCTCG

ACATTCTTGGTCGCGTTGTGCCAGGCTATCGACCTCCGCCACCTCGAGGAGAATGTCAAG

AATGCTGTCAAGAGCTGCGTGAAGACGGTGGCTAGG

>Traes_1DL_2200EA8BC

CGCCAACCACCTCAGGTTCGACATCTACGACCTCGAGCTCACTGGGGTCGACTCCAACTC

CGACCTCAGGAAGCTTCTCGTCGGGATGACCAACCGGTCCATTCTCGTAGTTGAGGACAT

TGACTGCACCATCGAGCTGAAGCAGCGGGAGGAAGACGATGAGGAGCACTCCAAGTCCAA

CTCTGCAGCAAAGAAGAAGGCAGAAGACAAGGTAACATTGTCTGGGCTGCTCAATTTTGT

TGATGGCTTGTGGTCGACGAGTGGGGAGGAAAGGATCATCATCTTCACAACCAACTACAA

GGAGCGTCTCGACCCGGCACTCCTGCGGCCTGGCAGGATGGACATGCACATACACATGGG

GTACTGCACCACGGAGGCCTTCCGGATCCTTGCCAACAACTACCATTCCGTCGACTACCA

TGCCACCTATCCAGAGATCGAGGCGCTGATCCAGGAGGTGACGGTGACGCCTGCAGAGGT

CGCCGAGGTTCTGATGAGGAACGACGACACTGATGTTGCCCTCCATGATCTTGTCAAGCT

CCTAGAGTTAAAGAAGAAAGAGGCCACTGAGATCAAGACTGAAAGTAAGCAGGCGGAGGA

GCAGAAAGACGGCACTGAGAAGATCAATACTGAAAGTAAGCAGGCGGAAGAGAAGAAAGA

TGGCGATGAGATCAAGACTGAAAGTGTGCAGGTGGAGGAGAAAAAAGATGACAACGAGGT

GGTGCTGAAGAATGAGTTGACAGAAAACGGAAGCGGCTAGGAGTGCAGAAGCTGATTGTG

ACCAGAGACTGAGCATAGTACTACGTAGATAGGTCCTACAGGAGATACATATGTTGTGAT

CATTTTGACAACTAACAACATTGCATTTTTTGGATTTGTTCGGCGCACTTGTATTTAACT

TAAACACAGTGCTTCTAAATAATTGTGCAAGTGCTGTTACATATATGCAAATTTGACATC

TTGCATAAGATATTGGCCAACAAAGCCATGATGATGAACAAACGAGGCATGTGGGTTGTG

GCCCATACTTGTCCACCAGAACAGAACGATCTTCATCTTCACAGAACAAGATGGTGCTAC

TGATAAGGAATGATCGACACTCTACTTGATTAGCCAGATAGATAAAGTAGTATGAGTCGT

AGTAATCTCATCACTAGCCCCAAGAGGATACGAGTAGCCAGCCACTGGAGTCAGGACGCA

TATAGTTGCGACGACATCACCTCGGTCCTCGGCGTACGTCGACCGGTCTTTGGTCAGAGC

AGGCCGTGCGTGGAATTCCAGCATGCACGCGGAACTGAATGACCGTGGAATGAAGTTTGA

CAAACTAAAGCTAGGCGTAATGCATACATGATACAGCGCAGCACACGCACCGAC

>Traes_2AL_C07505E42

ATGGAGTGCGAGAATGCACACGTTGCCGCCAACGGCGATGGCTTGTGCGTGGCACAGCCG

GCGCGGGCCGACCCACTGAACTGGGGGAAGGCGGCGGAGGAGCTCTCGGGTAGCCATTTG

GATGCCGTGAAGCGGATGGTAGAGGAGTACCGACGGCCTGCGGTGACCATGGAGGGCGCC

AGCCTGACCATCGCCATGGTCGCCGCGGTGGCTGCCGGCAGCGACACCAGGGTGGAGCTC

GACGAGTCCGCCCGGGGCCGCGTCAAGGAGAGCAGCGACTGGGTCATGAACAGCATGATG

AACGGCACCGACAGCTACGGTGTCACCACCGGCTTCGGCGCCACCTCTCATCGGAGGACC

AAGGAGGGCGGCGCTCTCCAGAGAGAGCTCATCCGATTCCTTAACGCGGGAGCCTTCGGC

ACCGGCACCGACGGCCACGTCCTGCCTGCCGCGGCGACGAGGGCGGCGATGCTCGTCCGA

GTCAATACCTTACTCCAGGGATATTCTGGCATCCGCTTCGAGATCCTGGAGACGGTCGCC

ACACTTCTCAACGCCAACGTGACACCATGCCTGCCGCTCCGGGGCACGATCACCGCATCC

GGTGACCTCGTCCCGCTTTCGTACATCGCGGGCCTGGTCACCGGCCGCCCAAACTCCATG

GCGACCGCTCCAGATGGCACAAAGGTTAATGCTGCTGAGGCATTTAAGATCGCCGGCATC

CAGCATGGCTTCTTCGAGCTGCAGCCAAAGGAAGGCCTAGCCATGGTGAATGGCACGGCA

GTGGGCTCAGGGCTTGCATCCATGGTGCTTTTCGAGGCTAACATCCTTAGCCTCCTTGCT

GAGGTCTTGTCAGCCGTCTTCTGCGAGGTGATGAACGGCAAGCCGGAGTACACCGACCAC

TTGACCCACAAGTTGAAGCACCACCCCGGGCAGATCGAGGCTGCCGCCATCATGGAACAT

ATCCTTGAAGGCAGCTCCTACATGATGCTCGCGAAGAAGCTCGGTGAGCTTGACCCATTG

ATGAAGCCAAAGCAAGATAGGTATGC

>Traes_2AS_280CEC75B

ACGATCACTTCACCCGCTTGCTTGGTAAGGGGCTCATCCTCATCGACGGCGACGAGTGGA

AGCGCCACCGGAAGGTCGTCCATCCAGTCTTCAATGTCGACAAGCTAAAGTTGGAAAAGA

ATAATGGGCAGGTGGAGGTTGATATTAGTTGTCAGTTCGAGGAAGTTGCAGCGGAGGTGA

TCTCACACATGGCATTTGGGAGAAACCACAAAGAGGCCAAGGAAGTCTATCTGGCGCAGA

AAGAGCTCCAATTTCTTGCCTTCTCTAGTATATTCAATGTTTTCAACCTCGTCCCGGGAT

TCAGGTACCTTCCTACCAAAAGTAACTTAAAGATGCATGCACTTGAGAAAGAGGTGAGGA

GCATCCTCGTCAACATCATAAAGAATCGGCTCAACTGCAAGGACACCATGGGATACGGGA

ACGACATGCTTGGAATCATCATGAATACTTGCGCGCCAGAGCACGTGCACAACCCACTCA

TGAGTATGGACGAGATCATAGAAGAGTGCAAGACCTTCTACTTAGCTGGCCATGAGACCA

CCACACAGCTGCTCACCTGGACCATGTTCTTGTTAAGCACGCACTCAGAGTGGCAAGAGA

AGCTCAGGGAGGAGGTGATGAGAGAGTGTGGCAGGGAAATCCCTACTACTGACATGCTCA

ACAATATGAAGCTGGTTAACATGTTCATTCTAGAAACTCTTAGGCTATATGGCCCTGTCA

CAAACATCCAGAGGAAAGCAGGCTCCGATATTGAGCTCGGGGGCATCAGGGTACCGGAAG

GAACAATTCTTTCGATCCCAATCAAGACGATACACCGCGACAAGGAGGTCTGGGGCGAGG

ATGCTGAAGATTTCAAACCTCAAAGGTTCGAGAACGGGGTGAGCGGGGCTGCCAAGCACC

CCTATGCATTTCTACCTTTCTCTATGGGTCCGAGGACTTGCATTGGACAAAACTTTACCA

TGATTGAGGTGAAGGTTGTATTCGCAATGATACTGCAGAGGTTCTCAGTATCTCTCTCCC

CCAAGTATGTCCATGCGCCGATGGATGTATTCACGATACGCCCGAGATACGGTCTTCAAG

TGGTCCTCAAGAGCTTGCAGATGTAGAGTTGTGCTCATACCAGAAATATTTCCAACCCGA

CGTGTGGACTGTACCACGCAACGTGCAACTCAATCTACGCTTGGTGATTAATAATTCCCT

TATTTTGTCATCTTCATTGTACTACACCCTACCTTCAAACATCAATAAATTTTCCATTTA

ATGTATAGAA

>Traes_5BL_429152528

GCCAAGTTTCCAATCGAACAGCAGTGCCGTGTTCCCGCGCCGATAAGAGCAACCAAACTT

TTTGGACACCACACCTCAAGAAGCAACCAAGAGCGCGCGTGGCTGGGTACAGATCATCGA

CGGCGGAACGAAACGAAAGCAGCCCGTAACGATCTGTTGGATTCGACCGGCCATGGACCC

GCGGTGCAGCGGCCACTGGGAGAGCTCGTCGGAGGACGTGACCAGGTCGCTACTGCCCCT

GCACGACATTACGGCGGACGACGGCGCCCACCGCTCCTCCTGCTCGCCGCTCGTCGCGTC

GCTGCTCGCCAACAGGTACCTGTCGATCGCGGCCGGCCCGCTGGCCGCCGCGCTGATCTG

CGCGCTCGTCGACCTCGGCCCCGGGCACGCCGCGGCGCGCAACATGCTCGGCGTGCTGGC

GTGGGTGTTCATCTGGTGGATCACCGACGCCGTGCCGCTCGCCGTCGCGTCCATGGCGCC

GCTCTTCCTCTTCCCCGTCTTCGGCGTCTCCTCCGCCGACGCCGTCGCCAAGGCCTACAT

GGACGACGTCATCGCCCTCGTCCTCGGCAGCTTCATCCTCGCCCTCGCCATCGAGCACTA

CAACATCCACCGCCGCCTGGCCCTCAACATCACGTCGCTGTTCTGCGGGGACCAGGTGAA

GCCGGCGCTGCTGTTGCTGGGGATCTGCGGCACGACCATGTTCATCAGCATGTGGATCCA

CAACACGCCGTGCACCGTCATGATGATGCCGGTGGCGACGGGGATCCTGCAGAGGCTCCC

CGGCGACGCGGCCGCCGGCGCCGACGCGCGCGAGTTCAGGCGGTTCTCCAAGGCGGTGGT

GCTCGGCGTCGTGTACGCGTCGGCGATCGGCGGGATGGCCACGCTCACCGGCACGGGCGC

CAACATCATCCTGGTGGGGATGTGGTCCACCTACTTCCCGGAGGAGGAGCCCATCACCTT

CAGCTCCTGGATGTCCTTCGGCCTCCCCATGTCGCTCGTGCTCTTCGCGGCGCTCTGGGC

CACGCTCTGCCTCATGTACTGCTCCAACAACACCGGCAGGGCGCTCTCCGCTTACCTTGA

CCGCACCCATCTCAGGAGGGAGCTCAGCTTGCTTGGTAC

>Traes_1BS_C428DA20B

ATGGTTGTTCCGGTGATCGACTTCTCCAAGCTTGACGGCGCCGAGAGGGCCGAGACGATG

GCGCAGATCGCCGACGGCTGCGAGAACTGGGGCTTCTTCCAGCTGGTGAACCACGGCATC

CCTCTGGAGCTTCTTGACCGCGTCAAGAAGGTGTGCTCCGAGAGCTACCGCCTCCGGGAG

GCGGCGTTCCGGTCGTCCGAGCCGGTGCAGACGCTGGAGAGACTGGTGGACGCGGAGCGG

CGCGGCGAGCCGGTGGCGCCAGTCGACGACATGGACTGGGAGGACATCTTCTACCTCCAC

GACGACAACCAGTGGCCGTCCGACCCGCCGGCCTTCAAGGAGACCATGCGGGAGTACCGC

GCCGAGCTCAAGAAGCTCGCGGAGCGGGTCATGGAGGCCATGGACGAGAACCTCGGCCTC

GACAAGGGCCGCATGAAGGCCGCCTTCACCGGCGACGGCCTCCACGCGCCGTTCTTCGGC

ACCAAGGTCAGCCACTACCCGCCGTGCCCGCGGCCGGACCTCATCACCGGGCTCCGGGCG

CACACCGACGCTGGCGGCGTCATCCTGCTGTTCCAGGACGACAAGGTCGGCGGTCTTGAG

GTGCTCAAGGACGGCGAGTGGCTCGACGTGCAGCCGCTCGCCGACGCCATCGTCGTCAAC

ACCGGCGACCAGGTGGAGGTGCTCAGCAACGGCCGCTACCGCAGCGCGTGGCACCGCGTC

CTGCCCATGCGCAACGGCAACCGCCGCTCCATCGC

>Traes_1DL_B63CADDCD

GGCAAGGACAAGACCGGCAGCGTGCTCCAGCAGGCCGGCGAGACGGTGGTGAACGCCGTG

GTGGGCGCCAAGGACGCCGTGGCGAACACGCTGGGCATGGGCGGCGACAACACCAACACC

GCCAAGGACACCACCACCGAGAAGATCACCAGGGATCACTAGACGCATACATACGTCCAG

TCTTCCTAATTTGCTTCCTTTACTCGTTTGGTCGTTCGCGGGCCTTTTACATATTTGTAT

GTTTCCCTCTTGTGATTTCCGCTCATTTAGGGTAAGTTTGCCTTCGATTTGATGTTGATG

TACTCGTGTCCGGTTCTGTAATGAGTTACTTATAATACTTTGGTGTAAATGGATAACGAG

GACAGTCGAAGGTGCTTCAATCCGTTGGTTGTCAGAAGCTTTCTTCACTTCTTATCTTTC

TTTTTTTTGGAACGGGAAAGGCACTCTCAA

>Traes_4BL_C8337C9F6

CGCCCACCTCATCTCCCGCGCGAACCTTCCCGAGCTCCCACCGCTCTAGCGGGTCCGTAT

ATAAGAACCCCACCCCCTTCGTCCCCTTCCATCATCAATTCAACTCAACTCAGAGAGCAA

ACCAAAGCATATCCACATCCACCGAGCGGACCGAAAGAACAACTCGATCAGTCACAGACA

GAGTCTCGAATCTTGCGTTCGATTTGATCGGAGAAGATGGCGAGCAAGGGAGGCAACAAG

GGGGAGGGCCCTGCAATCGGCATCGACCTCGGCACCACATACTCCTGCGTCGGCGTCTGG

CAGCATGACCGGGTGGAGATCGTCGCCAACGACCAGGGCAACCGCACCACGCCGTCCTAC

GTCGCCTTCACCGACACCGAGCGGCTCATCGGCGACGCCGCCAAGAACCAGGTCGCCATG

AACCCCACCAACACCGTTTTTGATGCCAAGCGACTCATCGGACGGCGCTTCTCGGACGCA

TCCGTGCAGTCGGATATGAAGATGTGGCC

>Traes_1DS_3463BE1BB

ATGGTTGTTCCGGTGATCGACTTCTCGAAGCTCCACGGCGCCGAGAGGGCGGAGACCATG

GCCCAGATCGCCGACGGCTGCGAGAACTGGGGCTTCTTCCAGCTGGTGAACCACGGCATC

CCGCTGGAGCTCCTTGACCGCGTCAAGAAGGTGTGCTCCGAGAGCTACCGCCTCCGGGAG

GCGGCGTTCCGGTCGTCCGAACCCGTGCAGACGTTGGAGAGGCTGGCGGAGGCGGAGCGG

CGCGGCGAGGCGGTGGCGCCCGTGGACGACATGGACTGGGAGGACATCTTCTACCTCCAC

GACGACAACCAGTGGCCCTCCGACCCGCCGGCCTTCAAGGAGACCATGCGGGAGTACCGC

GCCGAGCTCAAGAAGCTCGCGGAGCGGGTCATGGAGGCCATGGACGAGAACCTCGGCCTG

GACAAGGGCCGCATGAAGGCCGCCTTCACTGGAGACGGCCTCCACGCGCCATTCTTCGGC

ACCAAGGTCAGCCACTACCCGCCGTGCCCGCGCCCGGACCTCATCACCGGGCTCCGCGCG

CACACCGACGCCGGCGGCGTCATCCTGCTGTTCCAGGACGACAAGGTAGGCGGCCTCGAG

GTACTCAAGGACGGCGAGTGGCTCGACGTGCAGCCGCTCCCCGACGCCATCGTCGTCAAC

ACCGGCGACCAAGTGGAGGTGCTCAGCAACGGCCGCTACCGCAGCGCGTGGCACCGCGTC

CTGCCCATGCGCAACGGCAACCGCCGCTCCATCGCGTC

>Traes_2DS_5BE75B34E

CAGTGGAATGACCTCTTCTGGGCAGTGCACCCGGGCAGCCGTGGGATCTTGGACCAACTT

GACAAGACACTCCAGTTGGAGCCCACGAAGCTAGCGGCGAGCAGAACTGTCGTACAGAAG

TTTGGGAACATGTTTAGCGCCACCGTGATCTTCGTGCTTGATGAGCTACGGCGTCGAATG

GAGGAGGAAGGAGAGCAAGCTGAGTGGGGGGCCATGGTGGGATTTGGACCAGGCTTCACT

ATCGAGACCATGGTGCTCCATGCAACTGGCGCTCTCAANNNNNNNNNNNNNNNNNNNNNN

NNNTATGTACGCAGCAGCGGAGAATAAGATTAAAGTCAATGTGCCCAACTATTTTAATTG

TTCTCATACAAAAGTTATTTTGATTGGATCTGTAACCTATTTGATTACCAAATCAAATAC

TCCAATAATGTACGATGTTATGGTTTGTGCTGATTTTGACAGCCATTAATGAATCTATAT

CGAAAGGCAGTCTAAAAGTATTGCTCACAAG

>Traes_2DS_56E6F7ABF

CTGGCCTACAACGTGGAGGACTGCATCGAGCTCCTCGTCCACGTCGACAATGGGACGACC

AGCTTCTGGCTCCGCCTCCGCCGGCTCTGCGTCCCCTGGGCGCGCCCGCCGGCGCTCGAC

CAGGCGCTCGACGAGATGGAGCAGCTCAAGGCCAGGGTCGCCGACGTCAGCACCACCTGC

TTGGTTCTGCAGACCTCACCGTATAGTTGTTGTTCAGGTGCCTAGAGGTATTGGGAAACT

GACGGCGTTGCACACGCTTGGTGTTGTCAACACTCAACATCTCTGCTTCCGGTGGGAAGG

CCATGGCCAAAGCTGCTCTCAAGAAGCTCACCCAGTTGCGCAAGCTCCGAGTGTCCGGCG

TTAACAGGAAGAACAGCAAGGATATGTTCTCTGCAATCAAGGGCCATCTCCATCTGGAAT

CCGTTGTCTGTCCAGCCTGACCATTTTATAGGTGCGCAACTCCAGCTCATTATCTGTCAA

GTTTGGTTCAGAGACGATGAAAAGTCTTGAGCTGCTCAAGCTACTGGTATATATATTTAA

TAGGAAAGCTAGCCAATATGTCCAGCCTTCTCTCACATTGCTCAACGGTCCTTAACACCA

TTGTGGTTTGAGTGGAAAACTGCTCAACTGTCCTTTTTCTCACATTGCTGCCAAAAGTGC

AAGGGAAAAATGGAGACAACACTAAGAGGCTTCTCTGACGAGCACAATGATCTGAGTGGA

AAAGAGTGAGATTGTTGAAAAACAGTGCATCTTATCCCAAGCCAAGCAAGGTGGGTCAGG

CATGACTGGAAAGCTTGTATGTTCTCCCTTGTTGGGAAGCCTTTTAGTACGTTCGGATAA

TGTTTTGTGTGAAAAAGAGCCAAAATTTTGGCAGAACTGATCATTGCTTAGATGGTTAGA

AGGGTGGTTTTACCTTCTGCCTACCTAGGGTCAAATACTAGACTTGGTGCTCTGTGTATC

TTACTAATGCGGATTATTATCTGTATAGCATCTCACATGGTGTCAGTGTCGATGTCCTTG

CTATAGTGTGGAAATATTTAGGATCATGTAGACATGGATTATTGTTTTTTTTACTATTGA

TTATTCTATATTTAGAGTGACGTGGACGTGGATTATTGTACTACATTTTTTTATTCACCC

AGAAAGAGTCGAACACGGTGGGCATGAGTCAAGTCAATGCATACTTTTCTACGCGGTAGG

TGGGCATCTCATTGGGTTGGTGGTATATATCCAACTGAGGCTAGCAGGAAGCGGTTCCTT

CTCAGAAGCTTCATCCCCGTTTCTCCTCTGCTCTCTTCACAACTGCTAGAGGATCCCTCA

AGAGTCCCATCTTCACAAGAAATGGCTACTAGTGCTAGCGGAGGTAGAACTCAAGAGAAC

AAGCCGAGCATTTTGAGCACATTACCGAAGCATCTGCCATTAGACTTTCTGAAGAGTATC

ACAGATCAGTTCTCAGAGAAGCGTGTAATTGGTGAAGGCGCTTTTGGAACTGTTTATAAG

GGAACAGCGCCAGATGGGGAAACCATTGCTGTGAAGAAACTTGCCGAAAACTCGCCCCTT

CCACGAGATAAAGCATTTAATAATGAGGTTCAAAATATTATGGCTCTCCATCATGAAAAT

GTTGTAAAGTTGGTTGGTTACTGTCATGAAAGTCAAAAGAAAGTGGTGCAGAACAATGGA

AGGTATATTGTTGCTGACATTGTTGAAAGTGTACTCTGCTACGAATATTTACCAGGGGGA

AGCCTTAAGAAGAATCTTTTTGGAGATACCAAAATGGACTGGGACACACGATTCAACATA

ATTAAGGGGATCTGCGAGGGTTTACTCTTTGTACACAGCATTCCTATTGTCCATATGGAT

CTTAAGCCCGAAAATATACTGCTGGATAGTAACATGGTACCGAAAATAGCGGATTTTGGT

CTCTCGCGACTCTTCGGTCAAGAACAAACACGGATGAACACACAAAATGTAGTGGGGTCA

TATGGATACATTGCTCCAGAATATCTATACAGAGGTGAAATCTCCACAAAGTCAGACATA

TACAGTTTAGGTGTACTAATCCTGGAGACCACCACAAGAGAGGAGAATTGCCGCGGGAAC

AAACCATCTGCAGAACAATTTATTAAAAAGGTACGTGAAAATTGGACAGAGCAGCACATC

GTATCAGAGTACCCATCATTAAAAGCAGATAGCCTCCGACAAATAAAAAAATGCATCGAA

ATTGGACTGCAATGTGTTGAGACTGATCGGCAGAGGAGACCTTCCATAGAAGCAATTATC

AACCAGCTCAATGGACGGCATTCTAATTGAGCTACGATGCCGTACTGATCTCCAGTCTCG

AGGGAGAATTAAGTTCGGGTCTTCTTCAGGAGACATGTGCAAGAAGCAAAGGACATGCTC

GGCTGTTTGCCAAATAATTTGCTTCGATATGTACGTCATTGTGGAACCTGTTAGCTTTCA

TTTCTTATGATGATTGCTACTCCAGATACAGTGCAGTTGCCTGTCCAATTTAAGTGCGAC

GTAAGAGTTTTATTCTCCCCATGTAGATTTTAAGACGCGCTAACATTTTCTCAAGGGCAA

TTAGAGTATTTGTAGTTCTAATTTCTAAACATAATACACTCTCACATTTAATTTACAAAA

AAAACTGCTTAAGGATTTTTTGCCGAAAAAGGCCGGTATACCCTAGGCCTCTCTGCGCCA

ATTGTATGCATGTATCCATCATTAAGAAGCTTCACGTACAAAGTAAAGTCATACAAGCCA

GATAAAAAGGCCAACACAACCGAAAGGCAATAGAGATTACATGACACTCTCAAGGACCAC

TAATACTAACTGTGTGGTGCTTGATATCAGGATTTTTTTTTAATCTAGCTTGATATCAGG

TGGGGAGCGCAGCGAATAGTCAATAATAGTGCAGCCTGATTGCTTACCTGGGCCAGCCAG

GCCATCAGTTCTCTCTTTTGAAAAATGTTCAACCTGTGTTTAAAAATGTTCATTGTGTAT

GAAAAAAAGGTTCAAAGTGCTTTTCACAAATGCTCAACATGTATTCAGATAATTTTTTCA

GAAAAACATGTATTCTAATAAATATTAATCATATATTTAAAAAATGTTCAACATGTGTCT

AAAAATGTCCAGCCTGTACGGAAGATGTTCAATTGCTATATATTTAAAATTCGACATGTA

TTTGAAAATTCTAAAAATAAAATAAAAATAACCGAAAATAACAAAAACATAGTAAAACGA

AAAATGAACAGAAACAAAAAGAAAATCAATAATAACTGATAGAGAAACATAGAAAATATT

TTATAACTTGCAAAACCTTCCC

>Traes_5DL_8E18BBBB0

CAAAAATACCAGATATAAGTGAATCATGCAGCATACATATATTTGTAATTTTTCTCACTG

GTCTATTAACTTGCAACAGTGCATCCGACTGGAAAGTGCCAACTACTTTCATATATGGTC

ATGACGATTGGATGAATTACCAAGGGGCGCAGCAAGCACGCAAGGACATGAAAGTTCCTT

GCGAAATCATCAGAGTCCCACAGGGAGGACATTTTGTGTTTATAGATAACCCTGCGGGGT

TCCACTCGGCGATCTTCTACGCGTGCCGGAAATTTTTATCTGGAGATGCAGGGGAGGGTC

TCTCGCTTCCTGATGGCTTGATATCTGCATGAGGTGTCATCTCGTGTGATATCATACCGA

GTAGCGGTATGGGCATAAAGCAAAGCTATACGACTATAGAAATGTTCAATTACTGGTGTC

ACCAATTTGGTTTTGTATGTATGAATTGTGTGAATATGTCATTCATATGTGCTTGCTTAC

CAACAACACTAGTGAGACAATTATGTTTTTGTTGTGGGTTTAGATATAATTATGTCATAT

ACGCAGGATATCCCTAGTCAGTTTTTAGGTCAAGTTACTGTGAGTGATGCTCTATATTCT

TTCAGGG

>Traes_7DL_EBF393F0D

CGGGCCGGCACTTCCCGGCGACCTCATGATCCGCGTCATCGCTGCCGGGAACACGGCGAC

GTTCGAGGCCTTGTACCTGGGCACCTGCAAAACCCTGACGCCGCTGATGAGCAGCCAATT

CCCCGAGCTTGGCATGAACCCCTATCACTGCAACGAGATGCCCTGGATCAAGTCCGTCCC

CTTCATCCACCTCGGCAAACAGGCTGGCCTGGACGACCTCCTCAACCGGAACAACACCTT

CAAGCCCTTCGCCGAATACAAGTCGGACTACGTGTACCAGCCCTTCCCCAAGCCCGTGTG

GGAGCAGATCTTCGGCTGGCTCGCGAAGCCCGGCGCGGGGATCATGATCATGGACCCCTA

CGGCGCCACCATCAGCGCCACCCCCGAAGCGGCGACGCCGTTCCCTCACCGCCAGGGCGT

CCTCTTCAACATCCAGTATGTCAACTACTGGTTCGCCGAGCCAGCCGGCGCCGCGCCGCT

GCAGTGGAGCAAGGACATTTACAATTTCATGGAGCCGTACGTGAGCAAGAACCCCAGGCA

GGCGTACGCCAACTACAGGGACATCGACCTCGGCAGGAATGAGGTGGTGAACGACATCTC

AACCTACAGCAGCGGCAAGGTGTGGGGCGAGAAGTACTTCAAGAGCAACTTCCAAAGGCT

CGCCATTACCAAGGGCAAGGTAGATCCTCAGGACTACTTCAGGAATGAGCAAAGCATCCC

GCCGCTGATCGAGAAGTACTGATCGAGGACCTTGCATGGAGATTTAGTGCGTGGTTGGCG

TTTCACATGCCCAACTAGTAGAATAAGGATCGCGCGTAGGTGTTTTTCTGTTACTACTTC

TCTATGTACCCTGCTCACTGCATTTACTTCGTAGTGAATAAATGATGAAGCTTGTGCCCT

CTACGAGTTTATTCTTGTGATCATTAAAATTTCCATTGAATGTTTTCTGAGACCCCACTA

CAGTAAATCATAAAGTTGCCTAGTGTCGG

>Traes_7DL_90CA0995F

TCAACAAGATGCGGGCCGTGTCGGTCGACGTCAAGGCTCGCACGGCGTGGGTGGACTCCG

GTGCGCAGCTCGGCGAGCTCTACTACACCATCGCCAAGAACAGCCCCGTGCTCGCGTTCC

CGGCGGGTGTTTGCCCCACCATCGGTGTAGGCGGCAACTTCGCTGGCGGCGGCTTCGGCA

TGCTGCTGCGCAAGTACGGCATCGCCGCCGAGAACGTCATCGACGTCAAGGTGGTCGACC

CCGACGGCAAGCTGCTCGACAAGAGCTCCATGAGCGCGGACCACTTCTGGGCCGTCAGGG

GCGGCGGCGGAGAGAGCTTCG

>Traes_1AS_A79E210D7

ATGCCTCCCCTCTACATACTGCTCGGGCTTCTCCTCTTGCACACTGCTCCTTGGTGCTCC

TCTGTAGCTGCAAATGAAGACACTCTCACATCAGACCAAGCACTTGCCGCCGGCGACAAG

CTCGTCTCAAGAAACGGCAAGTTCGCCCTTGGCTTCTTCCAGCCAGCAGCAAGCAGCATC

AGTAAGTCCTCCCGCAATGCCACCTCCCCTAGCTCCAGCTGGTACCTTGGCATATGGTTC

AATAGGATCCCAGTTTTCACTACCGTGTGGGTTGCTAATAGAGAGGAGCCCATCACCCAC

CGCAACCTCAACCTGACACAGCTCAAGATCTCAAGCGATGGCAATCTTGTCATCGTGAAC

TATGCCGCCAAAACTGAATCCATTGTTTGGTCCACTCACATTGTCCATGGTCGAACCAGT

AGCATAAACACCACCGCCTCTATAGCCGTTGTTCTCTTGAATAGTGGAAACCTTGCCCTA

CTCGCAAATAGCCAAGACATGTTGTGGCAGAGCTTCGACTACCCAACAGATGTTGCGCTT

CCTGGTGCCAAGTTGGGCCGGAATAAGGTCCCCGGTTTCATTCGTCGGTACATATCAAAG

AAAAGCCTAATTGATATTGGTCTAGGCTCATACAGCATTGAACTAGACAACACCGGGATC

GTCCTCAAGCACCGCAACCCCTCGGTAGTGTACTGGCATTGGGCATCCTCCAGAACATCA

TCGTTGAATCTTGTACAATTAATCAAGACCACGTTAGATTTGGATCCCCGGACAAAAGGT

TTGATTAACCCAGCTTATGTTGACAACGACCAAGAGGAGTACTACATGTACACTTCACCG

GATGAATCATCGTCTTCTGTGTTTGTCTCAATAGGCATCTCTGGTCAGATAAAGGTGAAT

GTTTGGTCACAAGCCAGCCAGTCTTGGCAAAGCATATATTCTGAGCCAGCCGATCCCTGC

ACTCCGCCTGCTACGTGCGGACCTTTCACGGTCTGCAACGGCATTGCACATCCATCTTGT

GACTGTATGGGGAGCTTCTCCCAGAAGTCACCGCAGGATTGGGAGATTGAGGATCGAACA

GGAGGATGCATCAGAAACACACCCTTACATTGCAGCACAAGTCGTAACAACAAAAGCATG

GCAAGTTCAACAGACATATTCCACCCCATTGCTCGAGTCGTATTACCCTACAACCCGCAA

ATTATAGATGTTGCTACCAGTCAAAGCAAATGTGAGCAAGCATGTCTTGGTTCCTGCTCC

TGCACTGCTTATTCCTATAGCAATAGCAGATGCTCTATCTGGCATGGGGAATTGTATAGT

GTAAATTTTAATGATGGCATTGATAATAATTCTGAAGATGTTCTTTATCTTCGCCTTGCT

GCCAAAGATTTGCTACCAGGTTCTAGAAAAGAGAAAAGAAAACCAAAGGTCGGAGTTGTT

ACTATTGTAAGTATTATTGGTTTTGGGTTAATAATGGTCATCCTGCTGTTACTGATTTGG

AGGAACAAATTCAAGAGGTGTGGTTTGCCTATATATGACAATCCAGGTAGTGCTAGTGGA

ATTGTAGCCTTCAGATACACTGACTTGGTTCGTGCTACTAAAAGCTTCTCAGAAAAGCTT

GGAGGAGGTGGTTTTGGTTCTGTATACAAGGGGGTGTTAAGTGACTCAACAACTACTATA

GCAGTGAAAAGGCTAGATGGGGCCCGTCAAGGAGAGAAGCAATTCAGGGCTGAGGTGAGC

ACAATTGGACTGATCCAACACATAAACCTAGTGAAATTGATTGGTTTCTGCTGCGAAGGT

GATCACAGGTTACTTGTGTATGAACACATGTTAAATGGGTCTCTTGATGGTCATCTATTT

AAGAAGAGCAATGCAAATGTTATCGTCCTGAATTGGAACATCAGATATCAAATATCCCTA

GGAGTTGCTAGAGGATTGTGTTACTTGCATCAGGGTTGTCATGAGTGCATTATACACTGC

GATATTAAGCCGGAGAACATACTTCTGGATGCATCATTTGTTCCTAAAGTTGCAGACTTT

GGGTTGGCAACGTTTGTGGGAAGGGATTTCAGCCGAATTCTGACTTCATTCAGAGGTACT

GTGGGTTATCTTGCCCCAGAGTGGCTTACTGGAGTGCCGATCACACCGAAAGTCGACGTT

TACGGCTTCGGCATGGTGCTATTGGAAATTTTATCAGGAAGGAGGAATTCCTCACCTGAA

ACATCGTATAACACTAGTAGCAGCAACAGTGGTCAGAATATTGAATACTTTCCTGTGCAA

GCCATCAGCAAGCTTCACGATGGAGATTTAAAGAGTTTGATGGATCCACAGTTACATGGT

TATTTCAATTTGGAAGAGGCTGAAAGGGTTTGCAAAGTTGCATGTTGGTGCATCCAAGAT

AATGAGTTGGATTGGCCAACAATGGGTGAAGTTGTCCGGGTTCTCGAGGGTCAACATGAC

ATTGATGTTCCTCCAATGCCAAGATTGCTTGCGGCTATAACTGAACAATCTGTTGTAA

>Traes_2DS_DF9A59ADB

GTCCGCTACTACCTTTAATGAGCTAATAAAATGTAGAGCGACTCCAACTATCTCCCCTGA

ATTTGCCTCCCAAAACCCCATTAGGGGGGAGGCGAGTGGCGTAGGGTTAGAGTGAGTTTT

TTGCTCAGAACCCCTAAAACAGGGGGATAGTTGGAGTTGCTCTTTAAGTATCATTTTAGG

TCCAAACATGTTTTGTGGCAACATACACAGTAAATATTCTTATATATCATACACTTACGA

GTTTATAGACTTCATCAAGTCTGTCTGACCTATGATGATATTGAAGCCCAACTGGACGTT

GACCTTGACTTGCTCTGTCCAGCGAAAGTAGTATGAAATTGTTACCTTTATAAGTATGAC

ATCTCTGATGAGATTATTATTAACTTGTTAGGTCGAATAGTAGGGCATGGGATGTTAAGC

ATTAAACCTGTAGTCGGAGAAGATAAGCATAATCATAAAAACAAAAAATAAGATAATATT

TGGTGATAATTTCAGTGGAATGTGTTGAGAAGTGGATGTTCTAGAGCCAGTAATGCATAC

AGGGATACCTAAAATGATCATGGATGAGAATATTGACAGTCTGCAGACTGCACAATTATT

TCAGCCTTTTACTGATTATCGTGGAGTTATATTCCCATGTAAGGTTATGAGCTTAGTATT

GAGTTGGTGGGAATCTAGTAAGATGTAGTTTAGTTGGTACAAGGAATTAAAAATAACTCG

TGGGGTCAACTTTTATATGCAGGAAACAGATGCAAATGGTCCTGCATATCACATTTTTAC

TCCATTCTTGTGTGTACTAAAAAGCTACTCATTCAAAAAGCTGGATGTGTAGGTTGGTGT

AATGATTATTAAATGGTCAGGAATTTGAGGTTGATTGATGTTGCCATTATGAAAGCTACC

ACTCTAGATAAAGTTCTGCATAAGTGGATGTACCTTTACTGAGTAATAGATGAATAGTGG

ACTAGCATCTGAGCTGTATTGGTGAATTGACTAATCCGAAATGTGTCCTGCAAAAAGAAC

GTGATAAAATGGACTGAAGGTTCATGATTTATTATACCACTTTCCTGTTGTACTGCCGAT

GAAGAAAAAGATAATGGCCATTTTATTGGTCTTGAAACTGACAAGCCACTTCAGAATTGA

CTGCTGATTGCTGAATCACTAGTGATATGCTTGCATGTTGTCAATTATGTTCTGATTAAC

ATGGTCTGGAGTTCTTAATGGGATGGTCCCCCTGTAAACCCTCTCTTTTCCTTTCCACCA

CTTCCATAACTAGTGCACTTAACGAAACCAGGCTGGTAACAGCCTTTATGTTCTTCCAAG

TTCTTTCATCTTTCCTGCGCATGATGCCCTCCTTGTCCCAGTATGCATCTCCATGAAGTG

CACCCATGTGCGATCCACCATTTTGGAGTGATCAAACTATGATAGTTATATTTTAGAGAT

ATATTTTAGATGGTGGTCTCTACTCTCTATGCCATATCATGCACCATTTAAAGTCCTTTA

GTATCAATCTCATGTAAAAATGTATGTGGTTTTGCTTGCTCTGGTATGCCTTTGGTGTTC

TCTGCCTCCAGTCCGAAATTCTCTAACATTTGTCTCAATTGCTACAGGTGGCTATGCATC

AAGACCCTCGAGATGCTTGTGCAGCAATCGCTGCAGAGTCATACAAACTATGGCTAGAGC

ATGAAAACAGGACAGACGATATAACAATAATCATCGTGCATATCCGGGATGCTCAAAATT

CAGGTCCTGCAGGGAGTGACAAAGAGAACTCCAGCAGCACCGGGGCGCCGATAGCATTGC

ACACGGTGCAGCCGGAGCTACCTGTATTTGTACCGTCAGAAGCAAGTCACCTGAACGGAG

TGGCTGCTGCTGAACTGCGGCGGCCATCCTCCTCTGGCTCTCCATCGGAGCGACGCCTCT

CATGCGTTACTCCTTCGCCTACACACCCTTTATTAGAAGGCGGTAAAGCATTGGAAGCTT

CCAGGTCGACCCAGATCGACATTGCCATATCCCAGCCAGTGGAAGCTTGGCATCCAAGGG

AAGGTGGTAACAAGCTAGAACGGGCAGTCTCCTGCTGACCATGCATGAGTTCTTTAGCTT

GAATCTCACAACAGAAGCGGCAGGACACCCGTCGTCATCCTCCGTAAGGGGGGGCTGACT

TCCCGAACAGTGGGTGTGATGCAGCCATGCAGGCTCGCAATGCTTGTTCTGCGCCGGTTG

CAAGTTGCTCGTGTTTTCTTAGAGCACCTGCTTACTGACAATGCAAAAAGGATTCTGACC

CAAACAGGGTCGTTTAACATAAAGATTGATGGGCTTGATCATGTGGCATTGGACGCCGCC

AAGAGTTAACTGGAAATGGAGTAAAGTGTCAGATAATTTGTACTATTTGTTATTTATCAC

ATCATGTTGAGTTGATGATTACAGACCTGTAGTCATATGGTATCAGCTGCAGTGCAGTGT

GGATACCACAACGAATAATTGTTGCTTCCTCTTAACGGGAGGGAGGGGTCTTTGTGACTT

GTGAGTTGGATATCAGTGAGAAATGCCCAGAGCTTACTGAAAGAAACACGTTAGCTTTAT

TCCAAAATGCTTATGGTTGCCGGAG

>Traes_2DS_C2B17C651

ATGGAAGCTTCAATATCTGTAGTCGCAGGTGAACTAGTGAGTCGTTTCATCTCCTTCCTG

ATGAACAAGTACCACTCCTCCTTGAGCCATGCACAGTCAGAGGAGAAGCTGGTGGAGAGA

TTGCAGAACCTCCTGATGAGAGTCAGCATGATCGTTGAGGAAGCAGATGCGCGGTACATA

ACAAATTCTGGGATGTTGTTACAGCTCAAGATACTCTCAGAGGCCATGTACAAAGGATAC

CGCGTGCTGGACACCTTGAGGTACCAAAACCTCCAAGACAGTGCAGGCATTAACGAGGTT

AGCATCAACGACTCATCTAGCAGCAGCTTGTACTTATCTATTCCTGTCAAGCGTTCTCGA

ACAAAAGCTGAGAAGGATGAGAAGGCCATGCGCCTCGAGTATGGTGCCTTGAAAAGCTTA

GAAATTGTTGTTGCTAACATGGCAGAATTTGTAGTGCTTCTGGGTGGATGCGAGCACATG

TCTCGTAGGCCATATGATGCTTATCTTTACACAAAAAACTTCATGTTCAGCCGACATGCT

GAAAAACAAAAGCTCTTGAGCTTCTTGTTGGAGCACAAGAACCCTCCTGGTGATCATGCG

CCGGCAGTTCTTCCGATCATAGGTGGTTTTGGAGTTGGGAAGAAAACATTGGTTGCTCAT

GTGTGTGGCGACGAAAGGGTTCGGTCACGCTTCTCTATTTTGCACTTGAATGGAGATAGA

CTCTTAACAATACTTGACCATGGAAGGACCATGTCTGGGACGATGTTGGTAGTTATTGAG

TTTGCTTCTGATGTAGGTGAAGATGATTGGAAAAAACTTAAAAGATTAGCCCGGTTTGGA

TCTGTGAATCCTATTTTCCTAGGTGTTTTGTCTTACGATGAGTTGAGGTACCTTTTCAAG

ACACTGGCATTCGATAGCGCAGACCCCGCAGAACATCCACGACTAGTACAATTAGCAGAT

GAGTTTGCCAAGGAGTTGGACAGTAAGCAAGGTTCACTTGTCGCAATAAATACATTCGCA

TATGCGTTGAGAATGAACCTCAGTGTTCAGTTTTGGCGTTGCTTATTTGACAAGGGGGTA

AGATACGTTAAAAGAAACCTCTCCATACATGGTGTACACCCAAGTATGCTTATAGAACAA

GGCCATCCAGTGGACATCACGGACTTCGCCTTGCATCCACTTACCATGACAAGTAATGTT

CAAATCAAGGAGGAATCACCAAGTGTGACATTGGTGGAACTTCTGGCAGATCCTAGTGTT

ACACCGGAAGGAGACTTCCATCTAGTTGCATGGGAATCAAGGATACCGCCTCATAAATCA

TTTGTTCATTTTGTTACAAGTCGTGCTCAGGATACACATGAAGGTAGTAG

>Traes_2AS_F05C69BE0

CTGTCATTCACGCCACCACCCATCCACGATCACGTAATACGTGCGCCACCAAACCATGTC

GGCCACCTCGTACGACCGCGCAGTGGAGCTCCGCGCGCTGGACGCCACCTACGCCGGCGT

CCGCGGCCTCGTCGTCTCCGGCGTCACCCACGTCCCGCGCATCTTCCGCGTCCCCGAGCA

GCACCAAGAACCACCGCAGGACACCACCGAGCCCATCGGCCATGAACCAGCAGCCATCCC

GATAATAGATCTGGCGTGCGGCGACCACGCAGCCGTTGTCGCTGCCGTGCGCCGGGCCGC

GGAAGAGTGGGGGTTCTTCGAGGTGATGGGCCACGGCGTGCCGGAGGTGGCTATGACCGC

GGCCATGGACGCGGTACGGGCATTCCACGAGACTGATGGCGGCGAGGGCAGCGACAAGGC

GCGGCTCTACTCACGTGAGCCGGCGAGGGCGGTCAAGTATCACTGCAACTTCGACTTGTA

CCAGTCGCCCGTGGCCAACTGGCGCGACACGCTCTACCTCCGCATGGCACCCACCCCGCC

TGACGCCGATGACCTGCCGGACAGCTGCCGTGACGTGTTGTTTGAATATGCCCACCAAAT

GAAGAATTTGGGGAATACTTTGTTTGAGCTGCTCTCAGAAGCTCTTGGACTCAAACCAAG

CCACCTAGCAGATATAGGGTGCAACCAAGGACAAGTATTACTATGCCACTATTACCCTCC

CTGCCCCCAGCCAGAACTCGCCATCGGGACAAGCCGACATTCAGATGGTGGCTTCCTGAC

CATACTTCTCCAAGACAAAATTGGCGGCCTCCAGATCTTTAACGAGGAGCAGTGGGTAGA

CATCACACCTACACCCGGAGCATTCATCATTAACATCGGTGATCTCTTGCAGTTGATCTC

CAACGATGGATTTAGGAGCGTGGAACATAGGGTTTTGGCAAAGAACATTGCTCCACGGGT

GTCGATTGCAAACTTCTTCGGAACGCATATCGATCCGACATCAATGAGGATTTATGGTCC

AATTAAGGAGTTGTTGTCTGACAAGAACCTACCATTATATAGGGAAACCCTCGCCAGTGA

TTACATCAAACATTACTACTCCATTGGGTTGGATGCGAAAACTGCTATTTCTCATTACCG

GCTATGAATTTTGATATGTGAAGGAGAACTTGTGCTTTGTTGGTGCAACAACAACCATGT

ATACTTATAACATCAATAATTGAGCAATGTGCAATGCTAAGAAATGGATAGTAAAATTAC

ATACATGTTG

>Traes_2AS_2B63DDD75

CTGCAAGCGTCCTCGGTCATGGAGCTAACCGGAGCCACCTTATTCATCGTTTCTCTCGCC

TCAGTGGTGATTCTAGCTTCCTTTCTGAGCCGCAAATTAGCACCAAGTTCCAAGAGTAGG

CGGCCTCCTGGCCCATGGCGTCTGCCCTTGATCGGAAACCTCCACCAGATCGTCGGGGCC

NNNNNNNTACTCCGGGACCTGGCCAAGAAGCACGGGCCGGTGATGTACCTTCGGCTGGGC

CAGGTCGACACCGTCGTGGTCTCCTCGCGGTCCGCCGCAGAGGAGCTGCTGCGGGAGAAG

GACCTCTCCTTCGCGTCGCGGCCCAACCTTCTGGTCGCGGAGATCAGCTTCTACGGCAAC

ATCGACATCGCCATGACCCCGTACGGCCCGTACTGGCGGACGCTGCGCAAGATCTGCACC

GTCGAGCTCCTCAGCGAGCGCAAGGTGAGGCACTTCTCGCCGGTGAGGGACAGCGAGACC

ATGTCCCTCGTTCGGAACGTCCGCGAGGCCGCCGCCGCCTCGGGTGGCAACCCATTCAAC

CTCGGCAGCCTGCTAATTTCATGCTCCAACTCGGTGACCGGGAAGGCGGTGTTCGGGGAG

AAGTGCAGCCCCGAGCTCCAGGAGCAATTTCTGTCGGCCATGGATGTGGTGCTTAAGCTC

AGCGGGGCGCTCTGCATCGGGGACCTCTTCCCGTCGCTGTGGTTCGTCGACGTGCTCACC

GGGCTCAGAAGCAGGGTATGGCGGGNGGGCCCGCCGGCAGCAGGACAAGGCCCTGGACAA

GATGATCTCTCAGTCCGAGTCCAAGATGCAGCGCGGTGATCACCTTCTCGGCGTTTTGCT

TAGGATCAAGGACGAGGGGCAGCTTGAATTCCCCATGGAAATGGATAACGTCAAGGCAAT

CATAATGGATATGTTCACGGCTGGGACGGAGACAACATCATCAGCAGCCGAGTGGGCCAT

GTCGGAGCTCATGAGGAACCCTGAGGTGATGGCCAAGGCACAGGCTGAGGTGCGCCGAAC

ATTCGACGATAAGAACCCACAAGATCATGAGGAACACGTTGCGGAGCTACACTACACGAA

GATGGTAATCAAGGAGGCCATGAGGCTATATCCAGTGGTACCAATGTTAATTCCCCATGT

CTGCCGAGAGACCTGCGACCTTGGCGGGTTTGAGGTCACCAAGGGTACAAGGGTTATGGT

CAATACCTGGGCTTTTGGTAGGAACCCCGAGTATTGGCATGAGCCTGAGGAGTTCAAGCC

AGAGAGGTTCGAGGACGGCACCGCAACCTACAAAGGATCGCGGTTTGACTACTTGCCATT

CGGGAGCGGGAGGAGGAACTGCCCCGGTGATAACTTTGGTTTGGCAGTGCTGCACCTCAT

GGTGGCACGGCTTCTATACTATTTTGACTGGAGCCTCCCTGCTGGTGTGAAGCCGAGTGA

GCTGGACATGGAAATGAGGGTTGGCATGACCTTAAGGAGAAAGAACCAACTGCACCTAGT

GGCGACACCATATAAGGCATGCAGTTGATATTTGAACCAAACTTATGCACCTTGTGGAGA

ATAATAATAGTGGAGTTGTACACACGATCGAGCCTGCACACTTGTGCGTACTCTGTATAA

GGTCCAGTTGTAAGGTTTGTGCGGTTTCTAGACGACGTTCATCGCTAGTCATGCTAAGTT

TTCATGCTCGTCTATTGTTCGGCTAGGAACTGTTGTTCTGATATATCAGATCGTGAAGTG

TTCGGATATGAAATAAAATGAAAATATGTTGTCCAAGATTGAGTGGCACA

>Traes_2AS_51739A52D

CAAATTAAGTCCACTGAAGCAATCCTCATGGAGCTAAGCGCAGCCACCCTAGTGCTCTCC

CTCCTCTCACTTGCGATTCTCGTGGGCTTTTTTGGCCGCAAATCAACACCAAGTCGGCGA

GCTCCCGGACCACGGTGTCTGCCTGTTATCGGGAGCCTCCACCACCTTCTCACGCCGCAG

CCACAGGTCGCCCTCCGAGACCTGGCCAAGAAGCATGGCCCGGTGATGTACCTCCGGCTG

GGCCAGATCGACGCCCTGGTGGTGTCCTCCCCGGCGGCGGCAAAGGAGGTGCTCCGAGAC

AAGGATCTCAAATTTGCGTCCCGGCCGAGCATTCTGGTCTCGGAGGTCATGGGCTACGGA

CAACGCGACGTCGTCTTCGCGCCATACGGCGCGTACTGGCGGACGCTGCGCAAGATCTGC

ACGGTGGAGCTCCTCAGCGAGCGGAAGGTGAGGCAGTTCGCGCCGGTGAGGGACAGCGAG

ACCATGTCCCTCGTTAGGAACGTCCGCGAGGCCGGCCGAGGTGGCAAGCCGTTCAACCTC

GGCAGGCTGCTCGTGTCCTGCTCCAACTCGATAACCGGGAAGACGGCGTTCGGGCAGACG

TGCAGCTCCGAGCTGCAGGATCAGTTTCTCTCGGCGATAGATGTGGCGCTCAAGCTCAGC

GCGGGGCTCTGCGTCGGGGACCTCTACCCGTCGATGTGGTTCGTCGATGTGGTCACCGGG

CTGACAGGCCGGCTATGGCGAGCCCGCCGGCAGCTGGACAAGGTCCTGGACAAGATCATC

TCTCAGTCCGAGATACGGCAAGGTGATCACCTTCTGAGCGTTTTGCTTAGGATCAGCGAC

GAGGGGGAGCTTGACTTCCCGATCGACATGGACAACGTCAAGGCAATCGTAATGGACATG

TTCACGGCCGGGACAGAGACGACATCGTCAATCTCCGAGTGGGTCATGTCGGAGCTCATG

AGGAGCCCGGAGGTGATGGCCAAGGCGCAGGCGGAGGTGCGACGAACGTTCGACAACAAG

AGCCCACAAGACCATGAGGGACTCGTAGGGGAGCTGCACTACACAAAGATGGTAATCATG

GAGAGCATGAGGCTGAATCCAGTGGTGCCACTGCTGGTTCCCCATGTCTGCCGAGAGACC

TGCGACGTCGGCGGGTTTGAGGTCACGGAGGGTACCAGGGTGATGGTAAACACGTGGGCG

TTGGGTAGGAACCCCGAGTACTGGCATGAGCCTGAGGAGTTCAGGCCCGAGAGGTTTGAG

GACGGCACTGCGACCTACAAAGGGTCGCGGTTCGAGTACTTGCCGTTCGGAAGCGGGAGG

AGGAACTGCCCCGGCGACACCTTTGGGCTGGCCGTGCTGGAGCTCATGGTGGCACGGCTT

CTCTACTACTTTGACTGGAGCCTCCCTGCCGGAGTGAAGCCGGGTGAGCTGGACATGGAA

ATGATGGTTGCCGCAACCTCAAAGAGAAAGAACCAGCTGCACCTAGTGGCAACACCGTAC

AAGGCATGCAGTTTTTAATTAAAACTTGACTTATGCACGTTGTAGTAGGGAATGATAATG

GTCGAGTGGTGTACACGCGAGCCTATGGTGTGGAGATTCTGTGCGGTCTCGCCAGGAAGT

CCGCGGATTTGTAGCTGCATTTTCATCGTCATGGGATGGCATGACTATCAGATGCTTTAC

TGTTCCAATATAAAATAAAATAAAATAAGTTGTCCAAGGTTGAGTACCTCAAAAAAATCT

CCC

>Traes_2AS_10D194CC7

AAGCAAGCAAGCAAAATAGCAAAATACTAGCTAGGAAAGCATCGATCATGGAGATGGAGC

TAAGCCTAGGCGTAGCCACGTCCCTGTTTTTGGTTTCTGTCATCTCAGTGCTGGCCTTAA

GCTTGCTTATTAGCCGCAAAAGACTGGCAGCAGCAAGCGGCACGAAGAAGAAGAAGCAGC

TTGGGCGGCCTGCTCCTGGGCCATGGCGTCTACCCTTGGTGGGCAACCTCCACCAGATCG

TGACGTCCAAGCTGCCGGTGGTCCTCCGGGACCTGGCGGAGAAGCACGGGCCGGTGATGT

GCCTCCGGCTCGGGCAGGTGGACACGATCATCATCTCGTCCCCGTCGGCGGCGCAGGAGG

TGCTCCGGGAGAAGGACCTCAACTTCGCGTCGCGGCCGAGCCTGCTGGTGTCGGAGGTGA

TGCTGTACGGGAACCTCGACATTGGGTTCGCGCCGTACGGCGCGTACTGGCGGACGCTGC

GCAAGCTGTGCAGGATGGAGCTCCTGAGCGAGCGGAAGGTGCGGCATTTCATGCCCGTGA

GGGAGAGCGAGACCCTGGCGCTGGTGAGGGCGGTGCACGAGGCGGGCCAGGGCGGCAAGA

GGCCCGTCAACCTCGCGCTGCTGCTCGTCTCCTGCTCCAACGCGATCACCGAGCAGACGG

CCTTCGGGCAGGTCGCCGGGCGCGAGCTCCAGGAGCAGTTGCTGGCGGCCATTAACGTTG

GCATGACGATCAGCAGCGGGTTCAGCTTCGGGGACCTCTTCCCGGGGCTGGGGTTCATGG

ACACCGTCACTGGGCTCACACGCCGGCTGTGGCAGGCGCGCCGTCAGATGGACGCCGTCC

TTGACAAGATCATCGCTAAAAGCGAGCAGAAAGGTGATGACCTTCTGAGCGTCATGCTTA

GGATCAGGGACGGTGGAGACCCTGAATTCCCCATCGAAACGACAACCATTAAGGCAATCA

TAGTGGATATGTTCTCGGGAGGGACTGACACCACAGCGACGGCTGCTGAGTGGGTCATGT

CAGAGCTCGTGAGGAACCCACCGGCGATGGCCAAGGTACAGGCAGAGGTGCGGCGAACAT

TCGACGGCAAGACCCCACAAGAGCACGAGGGGCACATACATGAGCTACACTACATGAGGA

TGGTCATCAAGGAGAGCATGAGGCTAAACCCGGTGCTGCCGCTGCTGGTCCCCCGAATCT

GCCGGGAGACCTGCCATGTCGGGGGTTTTGAGATCGTCGAGGGTTCCAGGCTCATGGTCA

ACTCATGGGCTATCGGTAGGAGCCCAGAGAGTTGGGACGATCCTGATGAGTTCAGGCCAG

AGAGGTTCGAGGACAGCATGGCTGATGACAAAGGACCCAGGTTCGACTACCTTCCATTCG

GAGGTGGGCGGAGAATGTGCCCCGGAAGTACCTTTGGGTTGGCCGTGCTGGAGCTAATCA

TGGCACGCCTTCTCTACTACTTTGATTGGAGCCTCCCAACCGGTGGGACTGAGCTTGACA

TGGACATGACCGTGGGCATAACGGCGAGGAGAAAGAACCAGTTGCACGTAGTGGCATTGC

CGTATAAGGAGGTTCCACTGCAAAGCTGATTCATATCTTTGTGGTGGACAATTTACCCAG

TTGTGCTCATTTGCGTCATGTGCATTTCATGTTTGTCCCGGGGAAATGGACAAATAAATA

AATTGGGCAATCGCCTAACTACTACTAGTACTATTTTAGCGCGGGGTTACTCCAGCAGAT

TTGGACGTTCGAGTAAAGGGAATGTGGTGTTGCACAAGTGTATCATGTAATGTCTATGGG

CCTTGGGTGGTGGGCTCTACATTTATTGTTTAAGAGATTAAATTGTGGGTTAAATTAAGA

CTTTGTGCATCAATTGATGCGGAGGCCTGTGGTCTTTCTCCTTTTAAGAAAAGTGTTTCC

AGT

>Traes_2AS_D3212BACF

TCTGAAGTTGCCAGGTCGTTGATCAAGTAGCTGAAAGTTCACTTAATTCGAACCTATATA

TATACTTGGTCTCTTTCCAGAAAGTTGGCGCAGAGGCACAGTTAGCTTCTCAGCCGACCC

CTCTGTTTGCTGCTATATTAAAACCCTGAGCCAGTGAGGTGATTGCCCCATTTCCCATGG

CGAATCCTGCACGAGTTGGTGCCTATTCCATCCTCCTGCCATCTGCACCTCACCAGGCCG

TCCGCCTCCCGGCGGCTGCTCCACCATCTGCGTCAAAGTGTGGGTTGCAGATGCACCATC

ATGGAAGAAGCAAGCCTCCGCGCGTCAGCTTCGCCTGCTCAGCCTCCAACCTTCCTGGCA

CCGAACCAGCATATGTCCTCAAGGCAATGTTGGCAGAAGAAACTATAGGACCGCGCAGTG

ATGTGGAACGGGATGCTAGAATACGAAAGCATCTCAAAAACCCTGAACTCTCGCCGTCTG

CGTATGACACGGCATGGGTGGCTATGGTGCCATTGCCGGACTCCGATCCGCAGGCTCCAT

GCTTCCCTCAGTGTGTTGAATGGATATTGCAAAATCAACACTCTAGTGGGTCTTGGGGAA

TCAACGAATTTGGCTTATTAGCCAACAAGGATATTATGTTATCCACATTGGCCTGTATCA

TTGCACTTCATAAGTGGAACGTTGGCTCCGACCACATAAGGAGAGGATTAGAATTTATTG

GAAGGAATTTCTCCACTGTCATGGATGATCAAATTGTTTCTCCGGTAGGCTTCAATCTCA

TTTTCCCTGGTATGCTTAACCATGCTTTCGGGATGGGTTTGGTAATTCCAGTCCCAGAAG

CTGATATCAATGGGATACTTCACCTCCG

>Traes_2AS_5F7EC1988

TTTGGCCTATGTTGCTGAAGAAGGGTTAGTAAACCTGCTCGACTACAATCAAGTGATGAA

GTTCCAGCGAAAGAATGGGTCGTTGTTCAACTCTCCTGCCGCAACTGCTGCTGCATTAGT

GCACTACTATGATAATAAAGCTCTCCAGTACCTCGACTCCATTGTCAGTATATTTGGTGG

TGCAGTACCAACAGCGTACCCACAGAATATATATTATCAGCTCTCAATGGTGGATATGCT

CGAAAAGATCGGAATATCTCGGCATTTTTCCAGTGACATAAACAGCATCCTGGACAAGGC

ATACATTTCCTGGTTACAGAGAGACGAGGAGATCATGCAAGATGTAGAAACATGTGCAAT

GGCGTTTCGCCTTTTACGAATGAATGGTTATGATGTGTCGTCAGATGACTTGTCCCATGT

TGCTGAAGCCTCCACTTTCCATTGCTCACTTGAAGGATATTTAAATCATACAAAATCTTT

ATTGGAGTTATACAAGGCTTCAAAAGTATGTTTGTCCGAAAATGAATTGATCCTGGAGAA

CATAAGCAACTGGTCAGGCCACTTATTGGCAGAGAAACTGCGCTGTGATGGGACACAAAG

AATGCCAATTTTTGGAGAGGTAGAATATACTCTTAAATTTCCCTTTTATGCAACAGTAGA

ACCTCTAGACCATAAGAGGAACATTGAACATTTTGATTCTAGGGTTACTCAGCAGCTAAA

GAGAAAAAACATGCCATGTCATGCCAATCAAGATCTTCTAGATTTTGCCGTTGAAGATTT

CAGTTTTTCTCAATCTATATACCAGGATGAACTCTGCCACCTCGAGAGTTGGGAGAAAGA

AAACAAGCTGGAACAACTCAAATTTCTACGCAAGGGGAGTCTGATAAATTGTTATCTCTC

TGCTGCTGCCACCCTATCCACGCATGAACTCTCTGATGCTCGCATTGCATGTGCGAAAAC

TATTGCGCTCGTACTTGTTACTGATGACTTCTTTGATGTTGGAGCATCGAAAGAAGAACA

AGAAAACCTCATAGCATTAGTAGAGAAGTGGGATCACCATCACGAAGTTGAGTTCTGCTC

TGAGCAAGTAGAAATAGTATTTTCTGCTTTTTATAGTACAGTTAAGCACATTGGAGAAAT

GGCTTCTGCAGTGCAAAAGCGTGATGTTACAAAACACCTGAATGAAACATGGCTACATTA

CTTGAGGTCTGCAGCGACTGAGGCAGAATGGCAACGGAATCAATATGTGCCAACAGTTGA

GGAATACATGATAGAAGCGGTTAACTCATTCGCAGAGGGGCCCATTATGCTAACATCACT

ATATTTTGTCCAACAAAAACTCGAGGAGTACATAATCAAAGACCCGGAGTACGATGAGTT

GCTTAGAATAAAGGGGAACTGTGGCCGTCTCCTGAATGATACTCGGGGCTTCAAGAGGGA

GTCAAGTGAGGGAAAACTGAACATCATCTCACTGCTTGTTCTTCAGAGTGGAGGTTCCAT

GTCCATAGAAGGTGCTCAAGAGACTGTACAGGAGTCTATAGCCTCATGTCGGAGAGACCT

GCTAAGGATGGTTGTTAGAGAAGACCGTGTAGTTCCTAGGGCATGCAAGGAGGTGTTCTG

GAGGTTTTGCAGGACAGTTCACTTGTTCTACTGTCACACCGACGGATTTTCCTCGCCCAA

AGAAATGCTCTGCACGATGAATGCAATATTCAGAGAGCCACTTAAACTTCAAACAACCAG

TCCTTTGGCTGTTCAATCAGAAAAATAATCAAGGATTCCTTATGCTATTATCCTGATCAT

ATATGGATGACACGGAAATATACCAAGCGGGGAGAGTAAATGCAATAATAATCACATGAA

CCTTACAAATGAATCACTCACCGGTATCTGATTGATATGCATGTCGCGAGCTAAAGGGTC

TCCGTCACGTGGTCTGAACCTTCTTGGTCAATGGCATAGTTAGCTCGACGTGATGTTTCT

AGCGATACTTTTGTCAAAAACTTTATTCTGCATTGTAAGCTGGTATGCCCAGCAAATTTA

TTTTAGCAATCAATCGAAGCTACAAATTTCTCACTCTTC

>Traes_2DL_D4AAE1E4C

ATTTTATGTGCACAAAAGCCCCACTTCTATTATCTGGCACCAGGGCCCCTCATTTGTGAC

CAGTGCCGCCGGTTCTTGCCTGCAGCCCCTCTGCGAATGCTTGGATGACACGGCGGCCCG

AGAGGTCGCCTCGTTCCTTCACCTCAAATCCAACTGGGCCGATCTGGACAAGGCCGAGAA

GCTACTGCTAGCTGTCGAGAAGAGGGTGAGGGCGCGAGTCACCGCAGAGGTGGACAAGCT

GAACCTCTGTGACCCTCAGGTGCAGGTCTGGCTGAGGCGTGTCGAAGAGCTACAACTGGA

TGCCATCGATGAGGACTACAGCCAGTTGAGGAAGTATTCTTGCCTCGGCCAGTGCACCAT

CCATGCTCACCAGTGTACATCGATCAGCAGGCGTGTTCTTGAGGCTCTAGATGAGGCAAA

TAAACTTATCGAAGAAGGGAGGCGGTTCAAGATATTTGGATTCAAGCCCCTGCCGAAGAT

TGTTGATCCCTTGCCTCAAATCGAGACGTTTGGTTTGGAGACCATGCTGAGTCAGCTCCA

TGATCTGTTTGAGAAGGGCGACTCGAACATAATTGGTGTGTGGGGTCAAGGAGGTGTTGG

TAAGACGACGCTTCTACATGTTTTCAACAATGATCTTGAAAAGAAGGCCCATGATTATCA

GGCATATGAAATTTAACACGGCTTTCACACGGAAAGGCAGTTAATACCATTCACCATGAC

AATTTGACCTGTTCACAGAATTATAGGTTGTTATCTTTATTGAAGTATCCAATTCAGAGA

CGCTAAACACAGTGGAGATACAACAGACTATCTCTGAAAGGCTCAACTTACCGTGGAATG

ATGCGGAGCCAATTGCCAAACGGGCCAAATTCTTGATAAAGGCACTTACTAGGAAAAGAT

TTGTAATCTTGCTTGATGATGTAAGGAAGAAATTCCAACTGGAGTATGTTGGTATCCCAC

AGCCAGAGCAAGCTGATCCTCACAACACGTTACCGAGAAGTATGCTTTCAGATGAATGCA

CAAAGAAGCTTGATTGAGATGCGAATTTTGGGTAACGATGCTTCATGGGAACTGTTCTTG

GGCAAGCTGAGCAAGGAGGCTAGTGCATCAGTTGGATGGCTTGGTTCCCAGAATGATACT

AGAGAGCACGCTATGAAAATAGCCCAAAGTTGTGGAGGCCTACCACTTGCACTCAATGTC

ATTGGGACCGCTGTGGCAGGCTTGGAAGAGGGAGAGTGGAGATCAGCTGCGGATGCAATT

GCTACCAATATGAACAATATTGATGGTGTGGATGAAATGTTTGGTCAGTTGAAATACAGC

TACAAAAGTCTCACACCCACTCAACAACAGTGTTTCCTATACTGCACTCTTTTCCCAGAA

TATGGATCTATCAGTAAAGAGCAACTTGTTGATTATTGGTTAGCCGAAGGTCTGCTATTA

AATGACTGTGAAAAGGGTTATCAGATAATCCGCAGTCTTGTTTCAGCCTGCTTGTTGCAG

GCCAGTGGCTCAATGTCAACAAAGGTAAAAATGCACCATGTAATTAGGCAACTGGGGCTT

TGGTTGGTCAACAAGTCAGATACAAAGTTTCTTGTTCAACCAGGGATGGCCTTGGATAAT

GCTCCATCAGCTGGAGAATGGAATGAAGCTACAAGGATCTCCATCATGTCTAATAACATC

ACCGAGGTTTCTTTCTCACCAAACTGCAAAAATGTCACGACTTTGTTGATGCAGAACAAC

CCAAAACTGAACAAAATGAGTTATGGATTTTTCAGGACTATGTCGTCCTTGAAAGTGCTG

GATCTTTCTCATACTGCAATAACATCACTTCCAGAATGTGCTACATTGGTTGCATTGGAG

CATCTGAATTTGTCTCACACACACATTATGAGATTACCTGAGTGCCTGTGGTTACTGAAA

GAGTTGAAGCATTTGGATCTGAGTGTGACTATTGCACTTGAAGATGCCCTGAACAACTGC

TCAAAGTTGCACAAGTTGAAAGTGCTCAATCTCTTCCGTAGCCAGTATGGTATCCGTCAT

GTTGACAACCTAAATCTGGATTCTCTGAAGGAACTAGTGTTCCTTGGAATCACTATTTAT

GCAGAGGATGTGCTACAGAAATTGAACATGCCTCGTCCTTTGGCAAACTCAACACATCGA

CTAAACTTGAAGAATTGTGCAAAGATGATATACATCAAAATCTCTGATCTAAACCACATG

AAGCACCTTGAGGAGTTGTATGTTGAATCATGCTATGACCTGAACACAGTAGTTGCTGAT

GCTGAGCTTACAACTTCACACTTGCTGTTCCTGACCCTGTCAGTTCTTCCCTCATTGGAA

AGTGTCGTTGTTGCACCAATGCCCCATAATTTTCAGTATATCCGCAAATTGTTCATTTCA

CAGTGCCCCAAGCTGTTGAACATCACATGGGTGCGAAGACTTCGTCTTCTTGAGAGGCTT

GCCATATCTCATTGTGATGGGATGCTCGAAATTGTTGAAGACGATCATGCTTCAGATGAA

CAAGATCATGGTATGGTAGAAACTTCACGCAATGACACAGGGCAGAGTGACTTCCCAAAG

TTGAGATTGATCGTATTGACAGGACTTAAGAAGCTGAGATGTATTTGTAAACCAAGAGAA

TTCCCATGCCTTGAGACCCTTCGGGTGGAGGATTGCCCAAATCTGAGAAGCATCCCGCTG

AGCTTCACACATAACTATGGGAAACTGAAGCAGATATGTGGTTCAGTTGATTGGTGGGAG

AAACTGCAGTGGGAAAATAGGGAGGAGGTGGCATGTCTGGACAGCAAGTACTTCATTCCA

ATCTGACAGAACCCCAATTTCAATGGCTTTTGTATGGCATGCAGGTGGCTTACTTCTGTC

TTTAGTCTTCACACAAATAAGAGGTCTCCTCTTAAATTGTTTAAAATTTGTATATGTGAC

AGGCATAGCTTCTGTACATTGTTAGTATTGTATTCATGTATACAATATGATATACTCTGT

AATTTGCTGGTATTGCATTTGTGTAATATGAATTGTTCATTTAAATCATAGTCCAACAAG

GCTCTGTGACTTTCTGTCCTATCAATTGTGCAGTATCTCCACAACAAAG

>Traes_2AL_A8DEE8564

GCGGTGTGGAGGAGACACTGGGTGGCGGCTAGAGCTGGGGAAGGAAACGCTCATCCGAAC

CGAAGTTGGTCGCAAGGTCTCGCCGCCTCTGCGCTGACTGGATCGAGATAACAGTCTGGA

CCCCGGTCACGACGCACGCAGCCCCTTCCACTCCATTCCACGTCAAAACCTCCTCCCGGA

CTCGAGTCAGAGAAACCCCAGCACACAGATCCCCTCCCTCACCCGGAACATCCAATCCGA

TCATCCCCGTCTCCCCACGGGACCAAATCGCCGCCGCCGCACCCCCTCTGGTCCGGCGCT

GCCTCCGACGCGGCCGCGCGCGCTCCGCTCCGATGGGGCATAGCGCGAGGAAGAAGAAGA

AGAAGGGCGGCGCCGGGCGCAAGGCGGCCAAGGACCACGCCGCGCAGCTCGAGGGCGACC

AGACCGCGCTCACGGACGAGCTCACGGCGCTGGCTGCAATTTTTCTCGAGGACTTCAAAA

TAACTTCACAATCACCTCACACCCGATTTAGCATATGTATCAGGCCTTACTCTGATGGCA

TGGGCTTTGGAGATTTAAATGTTTCGGCTATTCTTGACGTAATTTGCTTTGCTGGTTACC

CCCACAAGTGCCCAAAGTTGCGAATCATACCTGAGAAAAACTTGTGTAAAGAAGATGCTG

ATCGGCTACTTTCCCTTCTTTCTGACCAGGCAAACATTTATTCCCGAGAAGGGCGTGTTA

TGATTTTTGATTTGGTAGAGGCTGCTCAAGAATTCCTGTCAGCAATTGCTCCAGCTACTG

ATTCAACGACTACTGCTCCTCACTTAGGTTCAAATACAATACAGGAAGCAACTGACGCAG

ATGTGAAAGCTAGCCTTGATGGCGGTCCTTACCCTGGAATCTTTTACATCTATAACTCAT

TCGATTTGTATAATCAACTATATGATGATAATAGTTGGCAAAGGCAAGGTTTCGATCCTA

CAACTGATAATGCCAAGAAAAATATTGGATCTCAAGTCAATTCAAATGTTAGAAGCAAGA

GGAAAACAGTCAATGAGAAATCCCGTTTTTCAGCCGATAAGGTCAACGCTGCAAAAAATT

CATCTCAGGATAATGGTGAACAGCAGCATGCCATGAAGCATGGTGTTGTACGAGAGGTAG

TTCCAAGTTTACCTGTTGTTGCAGAGGAAACTGATAATGACAGCAAAACTTTGTCCACAA

GCAACAGAGGAGGTATGGCAGATACTCCAGAGAGGAGTTTCAGCAGTGTACATGAATCCG

AGGACTCTGACCTTGCAGATGAAGGTTGGAATGATGAAGATTCTGCCCCAGACTCTGGCT

CTTCAAATGCACCATCTCATGTTTCAGACATGTATGATGATGCTTCGCAAAATAAGAGAA

GGGATTTAATTCTGGTACACTTGCTTCGGTTAGCCTGTGCATCAAAGGATTCTCTTTCAG

CTGCTTTGCCAGTAATATCATCGGAGTTATGCAACATAGGGGTTCTTTCTGAATGGGCTA

AGCAATTAATTTCTGAATCTCCTGCTGTTTTTGGGGAAACTTTTGATCATGTTTTTGGGC

AACAAATGATATCTTCAGAGTGCTCTCTATTTTGGAGGGCTGACAATTCATCGTCTAGGC

CAAACTCCCGTTATTTGAATGATTTCGAAGAGCTCCGTTCACTTGGTCAGGGGGGCTTTG

GCCGTGTGGCATTGTGTAAAAACAAGCTCGATGGACGCCAATATGCTGTAAAGAAGATAC

GCCTCAAGGATAGAAGTCCTCAAG

>Traes_7BL_C3CF4F012

ATGAATTTCTGGTCGGCCGCCACGGACGGATCGGCGCAGACGAGGTGGCTCAGAGAGTTT

TTCGCGTTCGTGGGGCCGTACGTGAGCAACAACCCCAGAGAGGCGTATGTGAACTACAGG

GACCTTGACCTGGGCCAGAATGTGGTTGTGGGGAACGTCACCAGCTACCAGGCCGGTAAG

GTATGGGGCGAGAAGTACTTCAAGGGTAACTTTCGGAGGCTCGCGGTGGCCAAGGGCAAG

GTGGATCCCGACGACTACTTTAGGAACGAGCAGACCATCCCACCACTAGTGGCGACGAAG

TAACGATGAATATGCCATGCGATCAAGCTATATGTGTGAAGGAGTGGGTTTCTCTTAGGA

GTATTTTGGTGGCTGGTAGCTATTCTAGTTGTTCCATACTTTGGCACGGAAAGTTTCAAC

AAAAGTTTCGATGCTATTCAAATTTGGCTCAAATTGCTCCCACCAGACTAATTTGACCGA

GATGCAAATATTTGGTACCTGTTCACATGAGCAAAATTTACAACCGTTGACCAATAGGCG

AAACTCTATTGATCATAGCCTTACTATTTTTT

>Traes_2AL_748546422

CAACTTTTTCACTCCTCATACTGACATCGCTCTAATTCTCTGCTCCCACACCCGCGCCCA

CTGCGTGCGTGTATATAAGGCGGGCAGGCGGCTATCCCCTTCACCAAATCATCACAAGCT

AGATACTTGCATCTGCAGAAGACCGATCAATAGCTACCCAAACCAATAGCCTCGCTCGAA

CACGAGCATTTCCCAAATTTCTATCTCTCCTTGATAGCTAGCCATCTTACAACCCAGCAA

TGAAGCAAAAGATTGTCATCCAATTGAGCATGTCGTGCGACAAGAGCAGGTCCAAAGCCC

TCACCGTGGCCGCCAGAGCAGCCGGGGTGACATCCATGGGGATAACCGGCGACGCGAGGG

ACCAGCTGGAGGTGGTCGGCGACGGCGTCGACCCGGTGTGCCTCGTCAGCTGCCTCCGCA

AGAAGCTCGGCCACGCCCAAATCATCAAGGTGGAGGAAGTGAAGAAGCCGGAGGAGAAGA

AGAAGGAGGACCCGAAGCCGCCGGCGCCCATGCCCGTGCCCGTGCACCCGCCGCCGTACT

TCTACCCGCCCAGCTCCTACTACCCCCACCAGTACCCGCCGCACTTCTGCGACGAGCAGC

CCGGCAACTGCCGGACCATGTAATTTAATTCTGTCGGCGACTAGAAATAGCTACATGCGC

GTGTTTATTAATTCATTGAAGGAAATGAAAAGGACGAGAGATACTCTAC

>Traes_7DL_B979E9D9C

CCCTCCCGTGTCCCCTCCTCTCTATAAATCGATGTCCGCGCGCCTCCTCCAAGCCACGAC

CACTTCTCTAGCTCATGCCCGTCTAGCTTCCGGGTCTTCTTCTTCGACTTCTTCCTCGGC

TGCCGCCTGTGTCCCGATAGATCCGTTTCCATTCTTGAGCTACTGCGAATTCAAGGAAGA

CATGGACGAGCAGTGGATGATCGGGCAGACTTCCCTCAGCCTCGGCCTCAACGTCGGCGG

GCCGCGACGGGCTCCTCCGGTGACCAGGGACCTCGTGGAGGAGGACTTCATGTCCTCCAA

GAAGAATCACGAGGCTGAGGCGCTGGTGGCCGAGCTCCGGCGAGTGGGCGAGGAGAACAG

GAGGCTCAGCGACATGCTCCGCGCGCTGGTGGCCAAGTACGCCGACCTGCAGGGCAAGGT

CAGCGGCATGATGGCGGCGGCCAACAACCACCACCAGTCGTCCACGACATCGGAGGGCGG

CTCCTCGGCGTCGG

>Traes_5BL_C0367D3F5

CGACGCGTTACTGCAGCCGTGGGCGCCAAGCGTGGCGCGGCGGCACGGCGCTGCGTGCGC

GTCCCTCTTCACGCAGGCGCCCGCAGTGAACGTAGCCTACGCGCACGCCGGGGCTGGGCG

GCTGACAGTGCCGGTTGTAGCTGGCACGGTGCCGCCGGAGCTTCTCGGGCTGCCGGCCGG

GCTTGGACCGGACGATGTGCCGTCATTCTTGGGCAAACCCGACGAGTGCCCTGCGTCCCT

GGACCTGCTGGTGACTGGTGAGACAGTTCGTGGGCCTCGACGCCGCCGACCACGTGCTCG

TCAACTCCTTCCATGAGCTCGACAACCGCCTGCCGGACGACGCGTCCTACGGCTTCCACC

TGCACACGCCGACGACGGCCGCGACCAAGGCGTGGCTGGACGCCCGGCCGGCGCGCTCCG

TCGCGTACGTCTCCTTCGGCAGCATCGCCGCGCTGGGGCTGGAGCAGATGGCGGAGGTGG

CGGAGGGCCTTTTCAACACCGGCGCGCCATTCCTGTGGGTGGTCAGGGCCTCGGAGACGT

CCAAGATCCCGGACGGCTTCGCCGACAAGGTGGGAGAGAACGGGCTGATCGTGCCGTGGA

CGGCGCAGCTGGAGGTGCTGGCGCACGGCGCCGTGGGGTGCTTCGTGACGCACTGCGGGT

GGAACTCGACAACGGAGGCGCTGAGCGCCGGGGTGCCGATGGTGGCGGTGCCGCACTGGT

CGGACCAGCCGACGAACGCCAAGTACATCGAGGACGTGTGGCGCGTCGGCGTGCGGGCGC

GGCCGGACGCGGTTGGGGTGGTGAGGCGGGAGGAGGTGGAGCATTGCGTGAGGGAGGTGA

TGGGGAGCGAGGAGTACCGGACGAGGGCGACGGAGTGGAGTGTGAAGACGAAGGCGGCCA

TGAGCGAAGGCGGGAGCTCCGACCGCAACATCTTGGAGTTTCTCCGTGGACTCGGATCGA

GGAAATCCGAGCGGTGGAGAGCGGCGGAAGATCTGTAGTTTCGTGCTGTTTCTCTGACAA

GTAAGGCTGTGTTTGAC

>Traes_5DL_13700A47A

GCGCCGTCCTACCTCGGCGACGGCAGCGTCGACGCCTTCGTCCCCGTGTTCCGGCACAAC

CTGCAGGCCTTCAAGGAATGCTGCTACTCCATGGAGTAGAGTCATGCATTAATGGTGCAA

TGAACAAGAAAGAATGTTACCGTACGTGCGAGAGAGATGGAACAGAACAGCATGCCAAAG

CGGCGCGGTTGTTGGGTCGGGTTGGTGAGGGGTGGTCGAAATTTTTTTAGTTGTGAGTGA

CGACGCACTTTATGGTGGCGTCCTCCGATTGGTCGATCGATTGGTTATTTGTGCCCTGCT

CGAAGTTAATAATGTTGATGGATTTTCTTAATCTTCCTTTTGGTTGGCTCCAGTGCACCT

TTGAGGCCTCATTCGGTTTGAAGGATTTTCGTAGGAAAAATAGAAACAAAAATTTTG

>Traes_5DL_EE6797852

GCGGAGGGTCGGCAGCATGTCGCCGCAGGCGGCGGCGGCGCTCGGGTTCCTCCTGCCCAC

GTGCTGGGAGATCGAGGTCACCTGCGCCGCGGCCATGCTCATCGTCGCGCTCTACGCCGC

CTACGAGCTCCTCGGCCCGCGCCCCCCGCCCGCNNNNNNNNNNNNNNNNNNNNNNGCGCG

GGACCTCGACGGCGCCGACAAGTACTCCACGGGGCCGTCGGCGTACGTGGTCAAGCTGGA

GCTGCTAGCCGCGAAGAATTTGATCGCGGCGAACTTGAATGGGACCTCGGACCCTTATGC

ACTCATCACCTGCGGCGAAGAGAAGCGTTTCAGCTCCATGGTTCCTGGCTCAAGAAACCC

AATGTGGGGAGAGGAATTCAATTTTGTTGTTGACTCTCTTCCTGTAAAGATACAGGTGAA

AATATATGATTGGGACATTGTATGGAAGAGCACAACACTTGGTTCTGTTACTGTTCCAGT

CGAGTCTGAGGGGCAGAGTGGACCGGTTTGGTATACACTTGACAGCTCATCAGGTCAGGT

TTGTCTCCATATTAAGGCGATCAAGGTTCATGAGAGTTCCTCCAGGGCTCTAAACAACAC

TGCCGAGGCTGATGCTCGTAGAAGGATTTCCTTAGACAAACAAGGCCCTACTGTAGTTCA

CCAAAAGCCAGGTCATTTGCAGACAATTTTTGGGCTCCCTCCAGATGAGGTTGTTGAACA

CAGCTATTCATGTGCACTTGAGAGATCATTTCTATACCACGGTCGCATGTATGTTTCTTC

ATGGCACATTTGCTTCCATTCAAACGTCTTCTCTAAGCAGATTAAGGTTATGCTCCCTTT

GAGAGATATTGATGAGATAAGAAGAAGCCAGCATGCAGTTATTAATCCAGCAATTACGAT

ATTCCTTCGAACGGGAGCTGGAGGACATGGAGTTCCTCCCTTAGGATGCCCTGATGGAAG

GGTTAGGTACAAATTTGCATCGTTTTGGAACAGAAACCACACATTTAGAGCATTGCAACG

AGCTATGAAGAACTTCCATGCAATGATAGAGGCTGAGAAGCAGGAACGTGCTCAATCTGC

ATTGCGCGCACTCAGTAGCTCAAGAAAAAATAGCAGCAAGGAGATAAATGTTCCAGAAGA

TTGTGCTGATTTAACAGGGCAACTACAACCTTTTGTCAAAGAAGAAGTTCTAGTCTCTGT

ATTTAATGGAACATTCCCGTGCACTGCAGAACAGTTTTTTAATAATCTATTAAGTGATGA

CTCAAGTTACATAACAGAATATAGGACAGCTCGTCAGGATAAAGATATCAACCTGGGCCA

GTGGCATGTTGCAGACGAGTATGATGGTCAGGTGAGAGAACTTAACTGTAAATCCATATG

CCACAGTCCGATGTGCCCTCCATATTCAGCAATGACAGAGTGGCAGCATATGGTTCTTTC

AGCTAACAAAACCGATCTGGTATTTGAGACTGTGCAACAAGTACATGATGTTCCATTTGG

TTCCTTTTTTGAGGTACACTGTAGATGGTCTGTGAAAACTATCGATTCTAGTTCGTGCAG

TGTTAACATAAGTGCTGGTGCACATTTCAAGAAATGGTGCATAATGCAGTCTAAAATAAA

GAGTGGCGCTGTGGACGAGTTAAAGAAGGAAGTTCGAGAAATGCTAGAATTTGCGGAGTC

GTATATGCAAGAGGTTAGCTCTCCTAACCAACAAGACAAAGATCTTGGCCAGGATACGGC

ACCAGACACCGATGACATACCTGGTGATCAGTAACTATATTTGACCTACCGTCCAGTCCA

GTGATAGTTTATAATGTTATTTATACCGAGAAGAAGGCCCGGTGGATCTGACCTGTAACT

GTAAGTAACTTAGTCCAAATCTGATGTGATTGGCTGCCTCAAAGAAGGATGAGTAAGAGA

CCTTGTGGTGTATACACTGTTGGCTTCTTTATGTATATCTGTGAAATGGCCGATATAGGG

CACCCATAAGCATGTCAGTTACTGTTTCTCAAGTTGTACAGCATGTCAGCATAGAGGGGG

AAAGATCGTTTTCAGTTCCGTTTTATGGGAAAAAGAA

>Traes_7DS_8990E8E56

AGATGCCCCACGGGCTCCTGCCCTTCAGGAAAGCATTCAAGGGCACTTTCATTGCCGCTG

GAGGGTATGATCAGGAGGAAGGCAACAAAGTGGTGGCCGACGGCTATGCTGATCTCGTTG

GTTACGGGAGGATCTTTCTGGCTAATCCAGATTTGCCTATGAGATTCGAGCTCGACTCAC

CATTGAACAAGTACGACCGCAACACTTTCTACACGCATGATCCTGTCATTGGCTACACAG

ATTATCCTTTCCTTGAAGGCTCGAATGCCGAGTAGTTAAC

>Traes_5BL_50A355E47

ATGGCTGCGCAATCTTGGAACCCATTCTCGTGCTGCGTCGGCGGCAGCAGAGTGGCGGAC

GACGACTACGACGACTGCAAGCGGCGGATCAGGCGGAGTGTGAAAGGCTGCCCGAGGTCG

TCCTCGAGGATGTCCTTCAAGAGCCTCAGCTCGTCGGGGACGCTGTCGCCGGAGGATCTG

TCCATCACGCTGTCCGGCTCCAACCTGCACGCCTTCACCTACGCCGAGCTCCACAAGGCC

ACGGGGAGCTTCTCGCGCGCCAACTACCTCGGCTGCGGCGGCTTCGGCCCGGTCTACAAG

GGCGCCGTCGACGACAAGCTCCGCCCCGGGCTGGCCGCGCAGGCCGTCGCCGTCAAGTAC

CTCGACCTGGACTGCGGCACGCAGGGCCACAAGGAGTGGCTGGCTGAGGTTTTCTTCCTT

GGGCAACTGAGGCACAAGAACTTGGTGAAATTGATCGGGTACTGCTACGAGGACGAGCAC

CGGATGCTGGTCTACGAGTTCATGAGCGGCGAGAGCCTGGAGAAGCACCTCTTCAAGAGC

ATAAATGGCTCTCTCCCATGGATGACAAGGATGAAGATCGCTGTCGGCACGGCCAAGGGC

CTTGCCTTTCTCCATGACGCAGACCCACCGGTGATCTACGGCGACTTCAAGGCCTCCAAC

ATCTTGCTCGACTCGGATTACAACACCAAATTGTCCGACTTTGGGTTGGCCCAAGATGGG

CCCCAAGGCGACGAAACACACGTGACAACACGTGTCATGGGGACTCATGGTTATGCGGCG

CCGGAATACATTATGACGGGCCACTTGACTGCCAAGAGTGATGTATATAGCTTTGGTGTA

GTGCTTCTGGAGCTTCTCTCCGGGCTGCGATCAGTGGATCGTTCACGACGGATAAGGGAG

CAGAACCTGGTGGATTGGGCTAGACCATACCTCAAGCACTCTGACAGATTGTACAAAGTC

ATGGACCTAGCTCTCGAGTGCCAATACTCATGCAAAGGCGCCGAGGTGGCAGCACTGGTG

GCATACAAGTGTCTCAGCCAGAACCCAAAGTCTAGGCCCACCATGACGGAGGTGGTCAAG

GCCCTCGAGCCCGTCCTCAGCATGGAAGACTTCTTTCCTGTGGGCCCATTTGTGTTCACA

GTCATTGTGGAGGAGGACAAGGTGGTGGACATGAAGGTGGGGGTCGAGGAGAAGCACCAG

CACCGTTGCCAGAACCATCAAGACAGGCATCGGCAGAAGTACCCCGACTCAGCAATCCAT

GCCGGCACTGTGCCCCGCGCCCGCGATGGGTTCATTACCGGTTTTTGGGAAGATCTCAGG

GGGCTTACGCCCGTCTTTCTTCTTGATAGAGACAACGGGGGTTAG

>Traes_4DL_BBAAD2076

CCAGTGTCAGTTCCTCAACTCGCCCTCGGTCATTTAAACCCCGCATTCCGCAGTTGTGCC

TTTTCCTGGCGTCCATTCCCCCACTCGCTGCTGCAACTGCAACTGCCCCAGCGAAAAAAG

GAGTGAGTCACGGAACAAAAATTCTCCGTCGGTTCCGAGCGCTCGATCCAGTCGGCGCGG

CGCCGGATCGAATGCGGGAGGCGGAGGCCGCCGCCGCTCGATCTGGTATTTGAAGTAGAC

AAGGAGCTGATACGATCTATAGAGGATTGCTGCGCTTGTAGTTGTGGGCAAAATGGAGGG

GAGAGTTGCTGGCGACGTGGAGCTTGATTCTGCAGTGTTCCAAGTGTCATTGACCAAGAA

CAGGTATGAGGCAATTGCTTGTAATGGAGAGAGTGCTGAATCAGTGGCATCTGGTCCTTT

CGACCAACTAGTCCTGCACTTGGAAGATGCCAAAAATTTCCAATCACGTTCATCAAGTGG

CTCTTTTAAGCTGTTACTGGCTGGGGATGCAAAAGGCTCTACCTGGTTCACAAAATCCAC

CTTAGAGAGATTCCTACATATCATAAATTCGCCTGATGCATCCAAAACGGCGAATGGGAT

TTTACAGGAAATGTCTCAGCTGGAGGAAACTAGAAAGTTTCATGATTATCTACAGTCCAA

GGAACAACAAAATCTTATGGGTGGTGCTTTGACAGGTACATTCAAAGAAAGTTTTGCTTC

TTCCATTGTTCTTTGTACACGAAAGCCATCACAACCTTTCAGCGTGTATGGCTGTATGCA

ATATGGCTTTTGTCTCCTTTTTATGAAGCCTATCTAATCAAACTTTGGATTCACTTTACC

ATTTTAGTGCTATGAGTGGTGGACTTACTAACAGTTTAGTTCTACTCTTAGAGCCTGTTA

CTCATTCCCTGGTTGAAATCACATTTTTTATCGAGCCTAACACTCAAATAGTTGATGAAT

CGCAGTACTTCGTTGGTGACTTTAGTTAGTTCCTTGACTATTTAGTTGGTGACTTTATTG

TACCATTGTGTTTTGATATCTTTAAGACTTTGTTGGTGCATTGAGGCCATTGGCATATTT

TCC

>Traes_3AS_7E128A012

CAGAATCTTCCCCGAGCCACAAACCAAAGCAACACCGACAATGTCGATCGTGAGGCGTAG

CAACGTGTTCGACCCATTCGCCGACCTTTGGGCTGACCCCTTCGACACCTTCCGCTCCAT

CATCCCGGCGATCTCAGGCAACAACAGCGAGACAGCCGCGTTTGCGAATGCCCGGATGGA

CTGGAAGGAGACCCCCGAGGCGCACGTCTTCAAGGCCGACCTCCCCGGCGTGAAGAAGGA

GGAGGTCAAGGTGGAGGTGGAGGACGGCAACGTGCTCGTCGTCAGCGGCGAGCGCACAAA

GGAGAAGGAGGACAAGAACGACAAGTGGCACCGCGTGGAGCGCAGCAGCGGCAAGTTCGT

CAGGCGCTTCCGCCTCCCCGAGGACGCCAAGGTGGAGGAGGTGAAGGCCGGGCTGGAGAA

CGGCGTGCTCACCGTCACCGTGCCCAAGGCCCAGGTCAAGAAGCCCGAGGTGAAGGCCAT

CCAGATCTCCGGCTGAGT

>Traes_2AS_6B52B5150

CGTGGGGGTAGCTCCAGCTCCGGCGCCGGCGAGCGTGGGGCCACCACCAGTCGGAGGAGG

AAGCGCAGGGAACAAAACAGCTAGAATTTTAGCTATTACACTGCCGATAGTTGCTGCAAT

ACTGGCTGCTGTTGTAATTTGTCTTTATCTATGGAGGAGGAAGAGCAAACCGGCGCGGAA

GACATCACTATCATATCCAACTAATCCAGAGGACATACAGAGTATCGATTCACTCATTCT

CGATCTATCAACTCTACGAGCCGCAACAGATAACTTTGATGAAAGGAATAAACTTGGTGA

AGGAGGGTTTGGTGTGGTTTATAAGGGAATCCTTCCTGACAATGAAGAAATAGCAGTTAA

AAGGCTCTCACACAGCTCTCGCCAAGGGATAGAGGAGCTCAAAAATGAGCTTGTTTTGGT

TGCTAAGCTTCAACACAAGAATTTAGTGAGACTTCTTGGTGTTTGCTTGGAGGAACAAGA

AAAGTTACTTGCGTATGAATACATGCCCAACAAAAGCCTTGACACCATTCTTTTTGATCC

TGATAGGAGCAGTCAGCTTGGTTGGGGGACGAGATTTAGGATAGTCAATGGGATTGCTCG

AGGCTTACAATACCTTCATGAAGATTCTCAGCTGAAGATAATTCACCGGGACCTCAAAGC

GAGCAATGTTCTTTTAGACTCTGAATTTAATCCTAAAATTTCAGATTTTGGCTTAGCAAG

GCTATTTGGCAGTGACCAATCCCATGATGTCACCAACCGTGTCGTCGGAACCTACGGATA

CATGGCCCCTGAGTATGCTATGCGTGGGAATTACTCTATCAAGTCAGACGTGTTCAGCTT

CGGCGTCTTGATTTTAGAAATCGTCACTGGAAAAAGAAACAGTGTCGCATATGACTCCGA

GCAAGCTGTAGATCTCTTAAGTTTGGTGTGGGAGCACTGGACGATGGGAACAATTGTAGA

GATCATGGATTCGTCTATGACTAGCCATTCTCCAGGAGACCAGATGCTCAAGTGCATGCA

CATCGGACTTCTGTGTGTTCAAGAAGACCCCGCTGATAGACCGATGATGTCGGTTGTGAC

TGTCATGCTAAGCAGCAGCACTGTGTCTCTCCAAGCTCCATCAAGGCCAGCATTTTGCAT

CCAAAAGAGTGGCATGAACTACTCAGGCATGCACACAGATCCATATCCAGGAGTTTCACA

TTCTACCAGTAGATCACCTATGTCACCCAACGAAGTTTCGATTACTGAACTTGAGCCGAG

ATAAGTCCGAGACTGATGATGTATTCATTATGTACGCCATTCTTGCCAAAATAACCTATT

TTGATTTAGGGAAAGACCCTACATCATATGTATAATCTACTCGACACCCCCGTGGTAGAA

ATTTTACTGAGCAATGGCATAATACGCCAGTATAATTATATTGTCGGGGTTGTCTATTTG

TCGGGTTACGGCAACATTTTAAGATACGCTATCATCTCAACTTGTGGATCACTATCCTGT

ATATGCCTTATGTTGAGTCTCCATATGAAGTTCGTGGTCCAAAAGGTGAACTTGAACTTC

AATAAAAT

>Traes_5BL_6B3A76B8A

CGAACAGTAGCGTCGTGTTCCCGCGCCTATAAGAACAACCAAACTTTTTGGACACCACAC

CTCAAGAAGCAACCAAGAGCGCGCGCGGCTGGGTGCAGATCATTGACGGCGGAACGGAAG

CAGGCCATAACGATCTCTTGGATTCGACCGGCCGGCCATGGACCCGCGGTGCGGCGGCCA

CTGGGAGAGCTCGTCGGAGGACGTGACGAGGTCGCTGCTGCCCCTGCACGACATCACGAC

GGACGGCGGCGCCCACCGCTCCTCCTGCTCGCCGCTCGTCGCGTCGCTGCTCGCCAACAG

GTACCTGTCGATCGCGGCCGGCCCGCTGGCCGCCGCGCTGATCTGCGCGCTGGTCGATCT

CGGCCCCGGGCACGCCGCGGCGCGGAACATGCTCGGCGTGCTGGCGTGGGTGTTCATCTG

GTGGATCACCGACGCCGTGCCGCTCGCCGTCGCGTCCATGGCGCCGCTCTTCCTCTTCCC

CGTCTTCGGCGTCTCCTCCGCCGACGCCGTCGCCAAGGCCTACATGGACGACGTCATCTC

CCTCGTCCTCGGCAGCTTCATCCTCGCCCTCGCCATCGAGCACTACAACATCCACCGCCG

CCTGGCGCTCAACATCACGGCGCTCTTCTGCGGGGACCCGGTGAAGCCGGCGCTGCTGCT

GCTGGGCATCTGCGGCACCACCATGTTCATCAGCATGTGGATCCACAACACGCCGTGCAC

CGTCATGATGATGCCGGTGGCCACGGGTATCCTGCAGAGGTTCCCCGGCGAGGCGGCCGC

CGGCGCCGACGCCCGCGAGTTCAGGCGGTTCTCCAAGGCGGTGGTGCTCGGCGTCGTGTA

CGCGTCGGCGATCGGTGGGATGGCCACGCTCACGGGCACGGGCGCCAACATCATCCTGGT

GGGGATGTGGTCCACCTACTTCCCGGAGCAGGAGCCCATCACCTTCAGCTCCTGGATGTC

CTTCGGCCTCCCCATGTCGCTCCTGCTCTTCGCGGCGCTCTGGGCCACGCTCTGCCTCAT

GTACTGCTCCAACAACACCGGGAGGGCACTCTCCGCTTACCTTGACCGCACCCATCTCAG

GAGGGAGCTCAGCTTGCTTGGTCCAATGGCTTTTGCAGAGAAGATGGTTCTTGCCGTTTT

CGGGGGCCTGATTGTTCTTTGGATGACGAGGAGCCTGACGGACGACATCCCTGGGTGGTC

AGTCCTCTTCCACGGCAATGTCGGGGACGGAACAGTAACTATCATGATGGCGACGCTGCT

CTTCATAATCCCCAGCGGCAAGGGCGACGGCGAGAAGCTCATGGACTGGGCCAAGTGCCG

GCGGCTGCAGTGGCACATCGTCCTGCTCCTCGGCGCCGGCTTCGCCATCGCCGACGGGTT

CAAGGCGAGCGGCCTGACGGACATCCTTGCCGGGTGGCTGGGCTTCCTGCGGGGCGCGCC

GGCGC

>Traes_2AS_5AC5CDB7D

CGCCACGCGACCTAACCGTATCAGATCACGACCTCACTCTACTCATCCTACTCCTTTCGC

CATCAGCTGCTGCGCCAAGGCAGCAACAGTTCGATCGTCAGCGTCGGCCGTGGAAAATCC

ACACTTCTATTTACTTGGAAACTACCAGTACGCCCGTACTGTACCTCTACTCCGCGATAG

CACCCAGCAGCTCAACTCCATGGCGATGGCGGCCATGCGCATGCGACGCAGTCTTGCTCT

CCGTTACCAACTCGCCGCCGCCCTCCTCCTCCTCCTCGCGTTCCTCAACGCGCCGCTNNN

NNNNNCAGCCGCTGCCGTGGCAGCTCTGCGACGCCGCCGCCGGGAACTACACGGAGGGCA

GCGCCTACCAGGCCAACATACGCGCCCTCGCCGGCGGCCTCCCCAGGAACGCCTCCGCGT

CCCCGGCGCTCTTCGCCAAGGGCTCCGCCGGCGCCGCGCCGGACGCCGTCTACGCGCTCG

CGCTCTGCCGCGGCGACACCAATGCCTCCTCCTGCGCGGCCTGCGTCGCCGCCGCCTTCC

GGAACGCGCAGCAGCTCTGCGCCTTCAACCGGCGCGCCACCATGTTCGACGACCCCTGCA

TCCTCCGCTACTCCGCCCACGACTTCCTGGCCAACGTCACCGACAACNNNNNNNNNNNNN

NNNNNNNNNNNNNNNNNNNNNNNNNNNNNNNNNNNNNNNNNNNNNNNNNNNNNNNNNNNN

NNNNNNNNNNNNNNNNNNNCTATGCCGCCGGGGACCCGACCCGGCGGTTCGGCACGGGGG

AGGAGGGGTACGACGCGACGTACCCCAAGATTTACTCGCTGGCGCAGTGCACGCCGGACA

TGGCGGCGGACGACTGCCGGACCTGCCTCAGGGACATAATCGAGAAG

>Traes_2AS_C0D44124A

CGACGCAGTGAGCCACCGCTGGCGGGATTGCCATGGCCAAACCCCACCGCTGCTTCTCCC

CGTACCTCGCCGGCGTCGCTGCGACCTTCCTCCTCTCCATCCTCCACGCGNNNNNNNNNN

NNNGCCGCCGATGACGAGCCGCCTCCGTGGCCGATCTGCGGGCCCTATCCCCCCAGCAGC

AACTACACGCAGAACACCACCTACCGGGCCAACATCGATCTTCTCTCCGCCACCCTCCCC

AGGAACGCCTCCTTGTCTCCAGCCTTCTACGCCACCGGAGACGTCGGCGACGTGCCGGAC

ATCGTCTACGGCCAGGCGCTCTGCCGCGGCGACGTCGCCAACGCCTCCGCCTGCGAGGCC

TGCGTCGCGGCCGCCTTCCGCGGCGCGCGGCGGGCGTGCCCGCTCTACAAGGACGTCATC

ATCTTCTACGACCTCTGCCAGCTCCGCTTCTCCAACCGCAACTTCCTCCTCGACGACGAC

TACCTCGTCACAACCTACACGCTCCTGCGCTCCCGGGTCGTGGCCACGCCGGCGTTCGAC

GCCGCCGTCGGGCTGCTCCTCAACGCCACCGCAGACCACGCGGTGGAGGACTCCTCCAGG

AGGTTCGGCACGGGGGAGGAGGGCTTCGGCGACAGGAGGAACACCACGATTTACGCGCTG

GCGCAGTGCGCGCAGGAAAAGACGGCGGACGTCTGCCGGAGCTGCCTCAGCATCATAATT

GGGCAGCTGCCCAACTTGTTCAGAGGCAGGACAGGAGGGGGCATGTTCGGGGTGTGGTGC

AACTTCCGGTACGAGGTGTACCCTTTCTTCCCCGGCCGTCCGCTGGTGCAGCTTCCGCAG

TTCGTAGAGAGCCCGCCTGCTTCCGCGCCACCGGTGACCGGAGGGGAAGAGAAAAAGAGA

AATAGTGCAGGTAAAGTTCTAGCTATTCTGATGCCTACAATTGCTGTGATATTGTCCATC

GCTGTGGTGTACTTTTTCTGTTGGAGGAAGAGAAGACCAGAAGAAGATGCGTATCTACCT

TCTACCTCAGATGATATTCAACACATCGATTCGCTTCTTCTCGATCTAGCAACACTGAGA

ATTGCCACCGATGACTTTGACAACAGCAAAATGCTTGGTAAAGGAGGGTTTGGTATGGTT

TATAAGGGAGTCCTACCTGACGGTGAAGAAATAGCCGTGAAAAGGCTTGGTCAGACTTCC

AGACAAGGAATAGGAGAGCTGAAGAGTGAACTGGTTCTGGTTGCCAAGCTTCACCACAAG

AATCTTGTGAGACTTGTTGGTGTTTGCTTGGAAGAGCAAGAGAAAATACTTGTTTATGAA

TATATGCCCAATAGAAGCCTTGATATGATTCTTTTTGATTCTGAAAAAAACAAAGAGCTA

GACTGGGGAAAGCGGTTCAAGATAATCAATGGAATCGCTCGAGGCTTGCAATACCTCCAC

GAAGATTCTCAACTGAAGATAGTTCACCGAGACCTCAAAGCGAGCAATGTACTACTAGAT

GTCGATTACAATCCTAAAATTTCCGACTTCGGCTTAGCGAAGATATTCGGAGGGGATCAG

TCAGAAGATGTGACTCGTCGTATCGCCGGCACATACGGATACATGGCCCCGGAGTACGCC

ATGCGCGGTCAGTATTCCGTCAAGTCGGACGTGTTCAGCTTCGGCGTCTTGGTCCTGGAG

ATCATCACAGGGAGAAGAAACAGTGGCTCATATAACACTGAGCAAGACGTGGATCTTTTG

AATCTTGTATGGGAGCACTGGACCCGGGGAAACGTCGTCGAGTTGATGGATCCATCCCTG

AGCAACCACCCTCCCGTCGACCAGGTGCTCAAGTGCATCCACGTCGGGCTCCTGTGCGTG

CAGAGGAAACGGGCGAGCAGGCCGACGATGTCGTCGGTGAATATCATGTTCAGCAGCCAC

ACCGTCCGTCTCCCTTCTCTGTCCAGGCCGGCATTCTGCATCCAGGAGGTCAGTGTCAGT

GAAACCTCGACTGCCTATTCGGAAGCATATCCGCTCACGGAAAATTCAACTGTGATGTCT

TCGAACCAAGTGTCAATCACGGAGCTTTCGCCAAGATGAGTCCTGTATAGTTTGAAGCAG

TTAACTGAACCAGCTGTGTATAGTGTTTCTTACGGTACAGTATGTCAGTATCTTAACTTG

TTGGAAATGATCTTGGGAGGAGGAAGAGGCCCTGCTGTATCGGTGTCTATTACTGAATCT

TTGCAATGTGGATGTAAGTAGAACTGCTAAGATGCTATTAATAGGGATTGCAACTGGCAA

GAGTGGGGAACAAAAGTCATGGTAAAGCTGATGTATGGCGTGATAGATTATAAAGAATGC

CAATTTTTCTGCTCAACTAAATTATTACAACGGG

>Traes_2AS_B17A4F9EF

GCAGGCCATAAACACACATACTTTTTTTCTGGAATAAAATTAAAAAACGATTAGAAGAGA

AAGTCTTTGCTTGTTCTAGCTCCCGGTATATATACCCCCACCCTGGTCTGGTTGTAGCAA

TATCGTTCAAGACGATCACAGAACCAAACATCCACCAACAATCCACTTCGTGTCCTTTGG

ATCTACGCGGAGGAGACATGAAGGCCAGCTCCGGAGCATTGCTCGCCGTCATGGCGGTGG

CGGCGTTGGCCACGACGGCGCTGGCGATCGACTACACGGTCGACGACTCCCTCGGCTGGG

ACACGTACGTCGACTACGAGAAGTGGATCGCCGACAAAGTCTTCATGGTCGGCGACACCA

TAAGTACGTGCACAAACTTCCTATGATCTACACTCTACATGTTTTCTTCTCGACGGTAGG

GCAGCGTGGTATAACTCCGGCCGCG

>Traes_4DS_F2F520B2C

GGGAGACGTTCCTCATCCCGCGCGGGCTCATGCACTTCCAGTTCAACGTAGGCAAGACCG

AGGCCTCCATGGTCGTCTCCTTCAACAGCCAGAACCCCGGCATCGTCTTCGTGCCACTCA

CGCTCTTCGGCTCCAACCCACCCATCCCGACACCGGTGCTCACCAAGGCGCTCCGGGTAG

AGGCCGGGGTCGTGGAACTTCTTAAGTCCAAGTTTACCACCGGGTTTTAATTCCTGGGAT

ACTGCCC

>Traes_2AL_4C6E19DEA

GTCCACTACCTACTGCACCCACCATAGAGGCTAGTACGCCATTCATTGCATAAATCGTAC

TCCAAGTCTCCGGTACTCCACGACGTCCAATTCTTTCCGGCTCCACTGACACTCACGGTC

AACACAGAGAATGGAAGGGAGGGCGAAGAACCCAGCGGCGTCGGTGGCCGTCGTGGCGGT

GCCGTTCCCGGCGCAGGGCCACCTCAACCAGCTCCTGCACCTGTCTCTGGAGCTAGCCTC

GCGCGGGCTGGACGTGCACTACGCCGCGTCCCCNGCCGCACGTCCGCCAGGCTCGCGCGC

GCGTGCACGGCTGGGACGACGACGCGCTCCGCTCGATCCACTTCCACGACCTCGCCATCT

CCACCTACGTCTCCCCGCCTCCAGACCCCGCCGCCGACCCGCCCTTCCCCTCCCACCTCA

TGCCCATGTTCGAGGCCTTCACCGCGGGCGCGCGCGCCCCGCTCGCGGCCGTCCTGCGCG

AGCTCTCTGCTTCTCGCCGCCGTGTCGTCGTCGTGCACGACCTCATGAACGCCTTCGCGT

CCGAGGAGGCGGCGCAGCTGCCCAACGGGGAGGCCTTCGGGTTTTACTGCACCGCCGTGT

CGTCCATCGTCGGGCGGATGGACGCCGGGCACCGGCTCCTGCGCGACAACGGCCTCACGC

ACCTCCCCACCCGCGTGTCCCAGGAGTTCCTGGACTACGCCAGCAAACGCGCGATGGTGG

CGCGGTCGATTTCGGACGGCGCCGGCATCGTCGTCAACACGTGCCGCGCGCTTGAGGGCG

AGTTCGTCGACGTTGTTGCGGAGCAGACGGCGGCCGACGGCAAGAAGCTCTTTGCCATCG

GGCCGTTGAACCCGCTGCTCGAGGCGACGGCGTCGAACCAGGGGAAGACGCAGCGGCACG

AGTGCCTGAGCTGGCTTGACCAGCAGCCCCCGTCATCTGTGCTCTACGTGTCGTTTGGCT

CGAGCTCCTCGCTCCGGGAGGAGCAAGCCGCGGAGCTTGCGGCGGCGCTGCACGGCAGCA

AGCAGCGCTTCATCTGGGTGCTGCGTGACGCCGACCGCGGCGACATATTCACGGACGCTG

GCGACAACCGGCACGCCGAGCTGCTGTCCCAGTTCACCAAGCAGACCGAAGGCATGGGGC

TGGTGATCACGGGGTGGGCGCCGCAGCTGGAGATCCTGGCACACCGCGCCACGGCGGCGT

TCATGAGCCACTGCGGCTGGAACTCGACCATGGAGAGCATGAGCCACGGGAAGCCAATTC

TGGCCTGGCCCATGCACTCCGACCAGCCGTGGGACGCGGAGCTAGTGTGCAGCTACCTCA

AGGCCGGCCTCCTTGTGAGGCCGTGGGAGAAACACGGCGAGGTGATACCGGCGACAACCA

TACAGGAGGCCATTGAAACGATGATGGTCGCCGAGGAAGGGCTCGCGGTGAGGCAGCGGG

CAGAGGTGCTCGGGGAGGCCGTCCGTTCGTCGGCGGCTCAGGGCGGATCATCGCACAAGG

AAATGGACGACTTCATATCATACATGACAAGGTGATGTATGATGATGGAAATTTTACACT

GATGAAGAATGCTGTAGTTTCTGTTTAAATAAACTTTGAGCAGTTTGAGTTGAGCTTGGA

TTGTATCACTTGAGAGGAAATTTCATTTCGTAATTCTTGTCGTGGTTTTAGTTTAGAATT

ATGGAATGGAGTGATAGTATTAGGAGTAGTACTAAACAAGCATGAATGGCCCAGTATTTT

CTTTGGTTGACACTGTCTCTTTGGGATCACGGATTAGAAGACTTGTCTGCCTTTTCTAAT

GTATTCTTCCTGTAATCTTTGAC

>Traes_2BL_8F1E5D5D9

CTCGGCGCAGGGCCTCGGCTGCATGGGCATGTCCGCTGTCTACGGCGAGCGCAAGCCCGA

GCAGCATGATCGCGCTCCTCCGCCACGCCGTCGCCGCCGGCGTCACCTTCCTCGACACCT

CCGACATCTACGGCCCCCACACCAACGAACTCTTACTCGGCAAGGCGCTGCAGGGAGGGG

TGAGGGAGCAGGTCCAGTTGGCCACGAAATTTGGCATCACGGCCACCTGGGAGGTCCACG

GCGACCCGGCGTACGTGCGGGCGGCGTGCGAGGGCAGCCTTGCTCGGCTCGGCGTCGACT

GCATCGACCTCTACTACCAGCACCGCATCTACAAGAATGTCCCCGTCGAGATCACGATGG

GTGAGATCAAGAAACTAGTCCAAGAAGGAAAGGTGAAATACGTTGGGTTGTCGGAAGCCT

CCGCGTCCACAATAAGAAGGGCACACGCTGTTCATCCCATCACCGCTGTTCAGCTGGAGT

GGTCTCTGTGGTCAAGAGATGTCGAAGAAGATATAATCCCAACTTGCAGAGAACTTGGCA

TTGGAATTGTGGCGTACAGTACACTAGGCAGAGGTTTTCTATCCACTGGACCTAAACTAG

TGAACATGTTACGGGAGGACGATTTCCGCAAGAATCTCCCAAGATTTCAACCCGAGAACA

TGGAGAAGAACGCGGCGATATTCGAGCGCGTGAGCGAGATGGCTGCGAGGAAGGGTTGCA

CGTCGTCCCAGCTTGCGTTGGCTTGGGTTCACCACCAGGGAGGCGATGTGTGCCCCATAC

CCGGCACGACGAAAGTTGAGAATTTCAATCAGAACCTGATAGCGCTGTCTGTGGAGCTCA

CGGCTGAGGAGATGGCTGAGCTCGAGTCCTACGCTGCCATGGATGCGGTCCAAGGTGATC

GGTACCACAACACGTTCCTCAACACCTGGAAGGACTCCGAGACCCCTCCCCTATCATCTT

GGAAAGCCACTTAATTGGGTATTATAGGAATCAATCACTATGCTGATGCTTCGTATCACT

GCTTTGAAATAGTTGCAAATCAAATAATTTCATGAGCACTCCGTGCATGGATGGAATCAT

GGAACCACCAGTTTTTTGCTAATGTAGCACAATTCCTGCCCATGAGTGTTGAACCTTTTA

TTATGCCTACAAGGATGTATCCCCTGAGTGTTGAACTTTTTGGCAAGGCTATAAAGGACC

TTTTGTAACACATTTCCATCCTAAAAACCATTTAGAATTCCAACCTCATGTGAAGATTGT

GGTAAGCGTCGGACCTCCGTACAGGGGCCGAGTTACATCAATTGTTG

>Traes_2DS_E45461FE6

TTCCTGGCGCGGCCGGACGACAACAACCCGGTCATCAACGCCATGGACGTGAACGGGTCG

ACGTACGCCGCGTGGGACAGCCGGAACGCCACGTCCCGGAGCTTCTTCCTGTCTCTGGTC

GGCACGCTGTTCGGGGAGATGTCCATGTACGGCGCGTACAACTCGTCGGTGCGCCGGTTT

GCCAGCGCCGTCATGTACATCAACCCGCAGCTGCCCACGGTGTACGGCCTGGCGCAGTGC

ACGCCGGACCTCTCCCCGGGGCAGTGCTGGCACTGCTTCCAGGGCCTCCAGGAGCAGACC

CGCCAGTGGTACGACGGCCGCGAGGGCGGCCGCATCGTCGGCGTCCGTTGCAATATTCGC

TATGAAGGCTACCAGTTCTACGACGGCATGGCCAACGTCAGGATCGGCACACACGGTGAC

TCATCTTCACCAACAGAAAGAAGCAAGCACAGGCAGACCCTAATAATCGTTCTATGCGTG

TCCGTTACGGTGTTCTGTTCTATGTTGGTTGGCTGCCTTCTGCTCATCAGAAGACTAAGA

AAAGGAGCTGGAAACACGAAATTAGAACAAGCACATAAGAGGAACAACTCGAAGACAGAG

GAGGCGCTGAAGCTGTGGAAGATCGAAGAGAGCAGCTCGGAGTTCACCTTGTACGACTTC

CCTGAGCTCGCCGCTGCCACGGACAATTTCTCCGAAGAGAACAAGCTCGGACAAGGTGGC

TTCGGTCCGGTTTACAAGGGAAAGTTTTCTGACGGGGCGGAGGTGGCGGTGAAGAGGCTG

GCGGCGCAGTCCGGGCAGGGGCTTGTGGAGTTCAAGAACGAGATCCAGCTCATCGCCAAG

CTGCAGCACACGAACCTCGTCAAGCTCGTGGGCTGCTGCGTGCAGGAGGAGGAGAAGATG

CTGGTCTACGAGTACCTGCCCAACCGAAGCCTGGACTTCTTTATCTTCGACCAGGAGCGA

GGGCCCTTGCTGGACTGGCAGAAACGGCGGCACATAATGGAGGGGATCGCGCAGGGGCTC

CTGTACCTGCACAAGCACTCCCGGGTGCGCATCATTCACCGGGACATGAAGGCCAGCAAC

ATACTGCTGGACAAGGATCTCAACCCCAAGATCTCCGACTTCGGCATGGCCAGGATCTTC

GGCTCCAACATGACGGAGGCCAACACCAACAGGGTGGTCGGCACCTACGGTTACATGGCA

CCTGAGTATGCTTCGGAGGGCCTCTTCTCGGTCAAGTCTGACGTCTTCAGCTTCGGCGTG

TTGCTGCTGGAGATAGTGAGCGGCAAGAGGAACAGCAGCGGCCACGGCCAGCACTACGGA

GAATTCGTCAACCTCCTCGGCTACGCATGGCAGCTGTGGAGGGATGGGAGAGCGTTCGAG

CTGGTCGACCCGACGCTGGGCCACTGCAGCGAGGTGGCGGACATCATGCGGTGCGTCAAG

GTGGCGCTGCTTTGCGTGCAGGACAACGCCATGGACCGGCCGACGATGACGGACGTGACG

GCGATGCTGGGCAACGACGGGGTGCCCCTGCCGGACCCGAGGCGGCCGCCGCATTTCCAC

CTCAGGGTCACCAGCGACGATGAGGAGGACGGCGCCGGCGGGTCTGGGACGCGGACACGG

TCCACGCACTTCACCGGATCGTGCAGCACCAACGACGTGACCATCAGCACCATCGAGGAA

GGGAGGTGA

>Traes_7DS_D7F6AF981

ATGACCACGAAGGAGCCGATCCCGCTGCTGACACCGCACAGGATGGGCCGGTTCGAGCTC

TCCCACCGGGTGGTCCTCGCGCCGCTCACGCGCTGCCGCTCCTACGGCAATGTGCCGCAG

CCGCACGCGGCGTTGCACTACTCGCAGCGGGCGACAAAGAGCGGCCTGCTCATCGCCGAG

GCCACCAGCGTCTCCGCCACCGCCCAGGGGTTTCCTGATACTCCTGGCATCTGGACGCAG

CAGCAGGTCAACGCTTGGAAACCCATCGTTTATGCCGTCCACAGCAAGGGCGCTCTGTTT

TTCTGCCAGATTTGGCACGCCGGAAGGGTCTCCTCAAAGCACCGACAACGCCGGCGAAGC

TCCGTTGCAGCACCGACAACGCTCCAATGCATCACCGATGGAGCTCCATTGCAGCCTAAC

GACCCGGCGA

>Traes_2AS_72C4C0EAC

GCGACGCCAACGCCTCTGCCTGCGGCGCTTGCGTCTCCGACGGCTTCAAGGACGCGCAGC

AGCTCTGCCCCTACAGTAAGGTCGCCGCCGTCTACTACGACCTCTGCTACCTTGGCTTCT

CCAACCAAGACATCCTCTCCGCCACCGACGGCGACAACAACGCCCTCACGCTGGTGAACA

GCGAAAACGTGACCGTGCCAGCGAAGGTGTTCGAAGCCGCCGTGAGCGTCCTCATGAACG

CCACCGCGGATTACGCGGCCGCTGACTCCTCCAGGCGGTTCGGCACAGGGGAGGAGGGGT

TCGAGACGATCGACAAGGCGAAACCCAAGATTTACGGTGTGGCGCAGTGCAGGCCGGATA

TGTCACCGGCAGATTGCCGGAGCTGTCTCGCAGATATCATTACGTACATCCCTCAGCACT

TAACAGGGAGGCGGGGTGCTAGGGTTGTTGGATTGCGGTGCAACTACAGGTATGAGCAGT

ATTCCTTCTTCACCGGGCCTTCTATGCTGCAGCTCCCGGCGCCATCCGTGGGGGCACCTC

CATCTCCAGCGCCGGCCAACGTGACGCCACCGCCAGTCGGAGGAGGTGAGCTTGGAGAAA

TAATTTCATCTTATTTGCACCATTCAGTCTTTAGACCAGAAGTAATTTCAAAATTGAGGG

ACAAAAACAGTGAGGTTTTATTTTTTCGGGAAGGTAAAACGGTGAGGTTCGATCTGCGAA

GAAACCAAAAAGTACAAACTCAAGGTGGACAGGATCAATTTCTATTATTTAATTTGATTC

TAGCATATAATTATAGAGTGTCAACACCAGCCAGGGGCCTTTTTACAGTCTGATTCAGTT

GTGCTAAAATAAGTAATCCAGTCTTCAGAGTTCTGTAAAGAACTCGGAATAAATTAGTTT

GTAGGACAGCTGAGACAAAACAAACCTTGGTAAACTGAAAAAGTAAGTGATGAGGGAAAG

ATTGCTCTCAACAAGAAACTTTGAAAATTTCTGTTGTTTGGTCTTGACTACTGCGCAGTC

GCGTAGCTATCAGTTAATTGACTGTGCAACAGCCATCACCGGTGTGG

>Traes_2AS_95E586D43

CCGCCGCTGGCCGCTTCCCAGTGGCCGAACTGCGGCAATAAGGGCAACTTCACCACGAAC

AGCCCCTACCAAGCAAACATCCGGGCCCTCTCCACCACCCTTCCCAAGAACGCCTCCTCC

TCGCGGACGCTCTTCGCGGCCGACAGCGTCGGCACCGTTCCGGACATCGTCTACGCGCT

>Traes_3DL_4C255B900

GTTATTTTAGATTTGTCTAGATACAGATGTATCTAAATATGTTGAGATACATCTGTATCT

ACACAAATCGTATCTAAATATGTTGAGATACATATGTATGTAGACAAATCTAAGACAACT

AATTTAGAACGGAATGAGTACTAATAAACTTTATAGGTAATCAAATTATAAATGTTTGTA

GGAGGTTATATGAACCAATTTTTTGTTGGAATATAAAAGGTTGGTCTCCGACTCTATAAC

TCCAATATTAATGCTAATTTTTAACTTATCTTGTGTTGATGCAATTAGTTCATGCGAAGG

ACATCATCGGAAGGACTCATGGACTGCAAGATGCTGCCAATCAATAACAACCACGGTGCA

GGGGTTGCAATGATAATGGGCCCAAACTTCTTAGCACGCAAGAACTACCAGCAAAGTTCA

CCTGAGGATTTGGCCCTGGCAAAAATGTTGGTGAGACCGGGAAACCTGTTCATGGAGGAT

CCGGTGATGAAGGATGCAAGCCTGCTCACCGATACCAACTACGGGTCGGTGAAGAAGGTA

TATGTGGTAGCAAAGGCTGATGGCTCCAGCACCGAGGAGATGCAGCGTTGGATGGTGTTG

TTGAGCCCCGGCACGGAGGCCGAGGAGATCGCGGGAGCTGACCACGCCATCATGAGCTCG

AGGCCTAGGGAGCTCTGTGATGCTCTGGTCAAGATCGCCGACAGCTTAAATACTTGCTAA

ATTGTACGATATATATCCCTGGAAATCAATGTAGGTGTGTACTTGATTTTCATCTAAAAA

TAAATATGTTCAGTGATACTATTAAATCAATGGAAATTAAG

>Traes_5BL_63AE36D3A

GCCAGTTGTGTCGCTGGCGCTGTCGCTCAGCACAGACTCCTCGGCGACGTCCACCACGAC

GTCGGAGTCAGACAGTTCGACCGGTGCGCCGGCGATGGTCCCCAGGAAGAGGGCGCGGCG

AGGGAGAGCTGTGGCCACGTCCGGGGAGGGGGAGTTCGTGTGCAAGACTTGCGGGCGTGC

CTTCGAGACGTTCCAGGCGCTCGGCGGGCACCGGACTAGCCACCTGCGCGGCCGCCATGG

GCTGGAGCTCGGCGTCGGCGTCGCCAGGGCCATCAAGCAGCGGAAGCGGCAGGAGGAAAA

GCAGCACGACTGCCACATCTGCGGGCTGGGCTTCGAGACGGGCCAGGCGCTGGGCGGGCA

CATGCGGCGGCACCGCGAGGAGATGTCACTCAGCGGCGCCATGGACCGGTGGGTCGCGCT

GTCGGATCAAGAGGCTGGGCACCAGCACGCCGCCGTGGACCGGCCGCCAGTCTTGCTCGA

GCTTTTTGTCTAGTTAGCTAGCTGGATCTTTGTGCAGCTCTGCTTGTTAGTAGTTCAGGA

CTTATTTCGCAGAAAAAAAAATCATGACTTGTACATATCTTCAGAATGAGATTGGCTCAT

CGCTCGAGCGGTTCGTTTTCTTAACAGATACTGTACATAGCTAAGACAAATTCTTTTGTT

GTAAATGTGATTGACAGAATCTTGTTCTCACACTGCTGTTCACCCCATTTTGTATTTTAT

TTTTGCGGGGCCATTTTGTATATGATCACAAGTCCAATTCGGA

>Traes_5BL_56E52A2FC

CGATCGTACATTTCCCGGCCGCAGTCTCACCGGTTGGTTTTGGTTGATCGGTAGTCGATC

CATCACAGCTCGGCGGGCCTCCATGACGAAGCACCAGAGAGCGCCGGCGGACCAGCTGGT

GTCCCTCTCCCTCTCGCTCTCCCTCGGCGCCGTCGCCGACCGCAACACCAAGAGGACGCG

CCGCGCCGCCGCCGCCGCCGGTGGGGAGTTCATGTGCAAGACGTGCCACCGCTCGTTCCC

GACGTTCCAGGCGCTCGGCGGCCACCGGACCAGCCACCTCCGCGGCCGCAACGGGCTCGC

GCTCGCGCTCGCCGGGACCGGGCCGGAGCCCAGGAAGGCCACGGAGCAGAAGCAGGGGCA

CCAGTGCCACGTCTGCGGGCAAGGGTTCGAGATGGGCCAGGCGCTCGGCGGCCACATGCG

CCGGCACCGCGAGCAGGAGGTCGCCGCCGCCGTGGCGCAGGCGCCGCCCGTTCTGCTCGA

GCTGTTCGTCTAGATTGATGATTGGCTAACTTTAGTTCTACTAGTACAGGGTTCTGATCT

GGCTCTGTAGTGTGCGCGACTGATTTGATAGTTGTTGCACACTCGTAGCTAGGTGCTGAT

CGTGGTTCATTCATTCATGTGTACTTTTGTGCACATATTGGTGACTTGTTCATTCATTCA

TTGGTTCAGAGATTATATATACACGTCAACTGATTTGCTCGTCTCTTTTGTTACTAGCCC

GGTGTCCCATTTTAACGGGGAGGGAGGACAAGTATGATGACTTTTGTTTATAAACATCGT

CCAATATAGTTCTTTTTGTTTCATTCTTCTTTTCACTTTATTACCGTTTTTAGTATCTTG

TGTGCT

>Traes_1AL_2103C5913

CCGCCCCGTCGATGCTTCAAATCCACTTCATATAATAGACATACCACACCTCAGCTCAGT

CCTCACTGCAGAAACACCACACGATCTGAAAAAACCGACAGAACATGTCGTCGGGGAAGC

AGGAGACGGCGGCGGTGCGCGTGCTGGGCAGGTGGCCGAGCCCGTTCGTGATCCGGGTGC

TGATAGCTCTTGGGCTCAAGGGCGTGGACCACGAGCTCGTGGAGGAGGCGGCGGGCAACA

AGAGCGAGCTGCTGCTCGCCTCCAACCCCGTGCACAAGAAGATCCCCGTGCTCCTGCACC

ACGGCAGGCCCGTCTCCGAGTCCCTCATCATCGTCCAGTACGTCGACGAGGCCTGGGCCT

CCCAAGCCCCGGCGCTCATCCCGTCCGACCCCTACGCCCGCGCGGCCGAGCGGTTCTGGG

CCCAGTACGTCGACGACAAGTTTCCTACGGCGATCCGGGTCCTGAGGGGAAGGCTGGACG

GAGACAAGGAAGAAGCGGCGGCTCAGGTGTGCGCCGCTCTGCAGCACCTGGAGGTGGCCT

TCGTCGAGTGCGGCCAAGGGAAGGATTACTTCGGCGGCGACGGCGTCGGTTACCTGGACA

TTGCTCTCGGGTCGCACCTCGGATGGGTCAGGGCGGTAGAGAGGATCGCTGAAATCAGGC

TGCTCGACGCGGCCAAGGTTCCTAAGCTGGCGGCGTGGGCGGATCGGTTCTGCGCCCACC

CGGCGGTGGCGAACGCCATGCCTAACGTGGACAGGTTCGTGGAGTTCAGCGTCAAGAATG

ACGGCGTTCTGAAGGCGGCTAGTGCTAATTCCAAGTGAGCGAACTGTTGTGGGCACTAGA

CAGCGCTCGATGGCATAGCCAGAATAAGTAACCTGCTGGCTGTTGGTGTAAACCCTCTTT

TATTATATGGCAATTGATAAATCTCGAATGTTTGGATTTTAAGGTGGCAATCCAAAAGTT

TTTCATGCAACTTTAGTTCACGCAAAGTTGACAAACATGACAATTTTCTGACAAAAAAAT

AAACTGAGACAAAGTTGTTATGTTTTAACAACTAAAGTTACCACCTCATGTCAACTAAAC

TTGCCATGAAAAAATGTTTGGGATGACATGCTTAAAATCCGAACATTCAAGATTTATCAG

GGTCTTTTTTATATAATGAAGAAAAGAGCTCTCGGTGCAATGCAG

>Traes_2AL_8DD050D521

ATGAAGCAAAAGATTGTCATCCAGTTGAGCATATCATGTGACAAGCGCCGGTCCAAAGCA

CTGACGCTAGCCGCCAGAGCAGCCGGGGTGACGTCCATGGGGATAACCGGCGACGCGAGG

GACCAGCTGGAGGTGGTCGGCGACGGCGTCGACCCAGTGTGCCTCGTCAGCTGCCTCCGC

AAGAAGCTCGGCCACGCCCAAATCATCAAGGTGGAGGAGGTGAAGAAGCCGGAGGAGAAG

AAGGAGGAACCGAAGCCGGCCGTGCCCGTGCCTGGGAACCCGCCGCCGTGCTACTACCCG

CCCAGCTACTACCACCACCAGTATCAGGCGCCGCACATGGTAGTCTGCGAAGAACAGCCC

GGGAACTGCCGGACCATGTAA

>Traes_2AL_247F64940

AGAGAAGTTGCCACACTTTCACGATTGCAGCATCAGCATGTTGTTCGTTACTACCAGGCA

TGGGTTGAAACTGAATATGGTCAACATAATGTTGTGAACACTGGGGGGTCACGCACTGCT

GAGAGCTCTATGTACAGTTTCGACGAGATCAGCTTGTCAGATTCAGGTGCTGGAAATAAG

CAGGAATGCACATACTTGTACATCCAAATGGAGTATTGTCCTAGAACCCTGCGACAGGAC

TTTGAGACATATAGTTCATCTTTCAATGTCGACCATGCATGGCACCTATTTCGACAAATC

GTGGAAGGCCTAGCACATGTTCATAGTCAAGGCATCATACACCGGGACTTGACACCTAGC

AACATATTTTTTGATGTGCGCAACGACATCAAAATTGGAGATTTTGGTCTTGCCAAATTT

CTAAAGCTGGAGCAGCTGGATCATGACCAATATATTCCTACTGAAGGAATGGGTGTTTCA

ATGGATGGAACAGGTCAAGTAGGCACCTATTTTTATACTGCACCAGAGGTAGAACAGAAG

TGGCCACAGATAAACGAAAAGGTTGACATGTACAGTGCGGGTGTTATATTCTTTGAGCTT

TGGCATCCATTTTCAACAGCAATGGAAAGACATCTTGTTCTCACTGATCTTAAACAGAAA

GGCGAGTCTCCAGTATCATGGTCAACACAATATCCTGGTCAATCAAATCTATTAAGACGC

TTACTATGCCCAAGCCCGTCCGAACGTCCATCTGCGATTGAGCTTTTGCAAAATGACTTG

CCTCCTCGGATGGAGGACGAGTGGTTAAATGATGTACTTAGAATGATACAGACACCTGAA

GACACTTACGTTTATGATCGAGTTATATCTACAATATTTAATGAAGATCGACTGGTCGCC

AAAATGCAATGTCAGCATGAAAGCAGTAAAAAAACCACTTATAAAAATGATAACAGTGAA

CTCTTGGATTCCATTATTGAGGTTAGCAAAGAGGTTTTTAAGCGACATTGTGCTAAAAGG

TTCCAGATATCACCCTTGCATACATTGGATGGAAAATTTACTGAAAATAGTGGAAAGACA

GTGAAGATCCTAACACAAGGAGGAGAGATGCTAGAACTTTGCTATGAGCTGCGAACACCA

TTTGTTATGAACGTTGCTGCTAACCAGTTATCATCATGTAAGCGTTATGAAATATCATGG

GTTCACAGAAGAGCAGTTGGCCATTCAACTCCTTATCGTTTTCTTCAGGGTGATTTTGAC

ATCATTGGAGGTTCTTCACCAATAACACATGCCGAAGTTATCAAGGTAGCTTTGGACCTT

GTGAGACGTTTTTACAATTCGAAGGCAATAGTTATTCGGCTGAATCATAGCAAACTCGCT

GAAGCAGTTTGTTCCTGGGCAGGAGTTCCTCAGGAACGAAGACAGAATGTTGCTGAGTTT

CTATCTTCTACTCTTGTTCAATATTGCCCAAATAAGGCGGATCGTAAGTCACAGTGGAGT

TTGATTCGAGGGCAACTATTACAGGATCTTCGTCTTTCTGAAGAAGTTGTTGAGAAGCTG

CATAAAGCAGATCAGCGGTTTTGTGGATCTGCAGATCTAGTACTTGCTAGATTAAGAGGG

ACCCTTTTCTATGATAAATCTGCTTGCAAGGCTCTTGATGATCTCTCCACTTTCCTCAAA

TGTTTGAGGATCTACTCAAAAGAAGGTAGTCCTGCGTCGAGTTCCCATGAAAAGCTGCTT

GCTGTTGGC

>Traes_6DS_324E2E32A

GTAAAATCCAACCGTGCTGATTACCACTACAAGGGACAGAACAAATGGGAGCAAGATCTG

CTTCTTTCTTTCCCATCATGCTCTTTCATGATTAGATCACCATTGATGCATCAGGTCAGG

GCACAACTTAAGCTCTGCTTCCCGTGCTAGTTCCACCAATAAACCATGCAGACACTCCAG

CTTCCCAACAAGACACACAGTAAGATATCGCACATACCTTCCCTTGGCCTTGCTCTTGTG

CTGCTGATATGCTTGGCCGCTCCTACCAGTTCCTGCACCGAGCAGGAGAAGGGCTTCCTT

CTCCAGTTCCTAGCCGGGCTTTCACAAGACGGTGGTCTTGCTGCCTCCTGGCACCATGGC

ACGGATTGCTGCCAGTGGGAAGGGATCACTTGCAGGCAAGATAGGACGATCACCGATGTC

TTGCTGGCCTCAAAGGGCCTTGAGGGGCACATCTCAGGGTCCCTTGGGAACCTAACCGGG

CTGCAGCATCTTAACCTCTCCCACAACTCACTGTCCGGTGGTCTGCCACTGGAATTGGTA

TCGTCCAGCATCATTGTCCTTGACATCAGCTTTAATCAGCTCAACGGAACACTGCTAGAG

CTGCCAACATCGACCGCTGCCTGGCCACTCAAGGTATTGAACATCTCGAGCAACTTGTTT

ACAGGACAGTTGCAATCCACCACATGGAAAGTGATGAAGAATCTGGTCACGCTCAATGCA

AGCAACAATAGCTTTACTGGGCAGATACCAACTCATTTCTGTAATACCTCACCATCCTTC

TCTGTGCTTGATCTGTGTTTCAACCGCTTCAGTGGCAGCATCCCCCAAGGCCTTGGTGGT

TGTTCAAAGCTGAGAGAGCTCAGGGCTGGGTACAACAACCTCAGTGGAACAGTCCCGGAT

GAAGTGTTCAATGCTACTTCGCTGGAATACCTGTCTTTTTCTAACAATGATTTACATGGA

GTTCTTGATGGTGCACACATACTCAACCTCAGAAATCTGTCTACCCTTGATCTAGGAGGA

AACAATTTCGGTGGCAACATTCCAGATTCCATAGGCCAGCTCAAGAAATTGGAGGAGCTC

CATTTGGACAACAACAAATTGTCAGGGGAGCTGCCATCAGCTCTAAGCAACTGCACAAAT

CTCATAACAATTGACCTCAAGAAAAACAATTTCAATGGACAGCTCACCAAGGTCAATTTC

TCCAACCTGCCCAATCTAAAAACATTAGACCTTCTAGAGAACAACTTCACTGGCACAATT

CCAGAAACTATATACTCTTGCAGCAATCTGACAGCACTGCGGCTGACTCGCAACCATTTA

GATGGGCAGCTTTCACCACGAATTGGCGATCTGAAGTACCTCAGCTTCCTATCACTTGCT

GAAAATTCTTTCAGAAACATCACAAATGCACTTCGGATCCTTAAGAGCTGCAGGAACCTT

ACCACCCTGCTTATCGGGGGAAATTTCAGGGGAGAGCTCATGCCAGAGGATGACATTCTT

GATGGTTTTCAGAATCTTCAGGTTCTTGCGATAAATGGGTGCTCATTGTTGGGAAAAATA

CCTCGTTGGTTATCAAAGATAACAAACTTGGAAATGTTATTTTTACATAGCAATCAACTT

ACTGGGCCGATACCTGACTGGATCAGCAGCCTAAATTTCCTCTTCTGCCTAGACATAAGT

AATAACAGCCTCACCGGGGAAATTCCAACAGCACTGATGGATATGCACATGCTGAAGTCA

GAAATGACTAAAGTCCATTTGGATCCAAGTATCTTTGAGCTTCCAGTTTATACAGGCCCT

TCATTTCAATACCAATACCGTGTACCCACTGCATTCCCTAAATCGCTGGATTTAAGCAAC

AATAAATTCACTGGTGAGATACCCTTGAATATCGGTCAGTTGAAAGGCCTCCTGTCAGTC

AATTTGAGCTTCAACGACTTGACAGGACAGATACCACAATCAATATGCAAGCTCACAAAA

CTGCAGGAGCTAGACTTGTCCAGCAACAATCTCACAGGTGGTATCCCAGCTGCATTGAGC

AACCTGAACTTTCTTTCAGCATTCAACATTTCACACAATGACCTAGAAGGGCCTATTCCA

TCTGGAGGCCAGTTTAATACATTTCAAAATTCTAGTTTTGATGGGAATCCAAAGCTCTGT

GGCACTGTGCTCACTGAGAAATGTCGTCCAGGATCAACACCTTTAGTTCCCAGAAAACAA

GGAGATAAGAAGGCCATTTTTGCAATTGCATTTGGTGTGTTCTTTGGAGGTATTGCTATT

CTTTTGTTGCTGGGGCGTCTCCTTGTCTCAGTCGGGATGAAAGGTTTTACAGGAAAAAAT

CGATGGGAGAATAACGAAGGTAATGAAGGAACTTCATTCTACTCCAGTTCAGTGCAAACA

CTAGTGGCGATGCGGATGCCACAAGGAAAGGGAGAAGAAAACAAGCTCCAATTCACCGAC

ATTGTGAAAGCTACGAACAACTTTGACAAGGAGAACATCATAGGATGTGGAGGTTATGGA

TTAGTCTACAAGGCAGAGCTACCTGATGGCTCCAAGCTTGCAATTAAAAAACTCTACGAT

GAAATGTGTCTGATGGAAAGGGAGTTCACTGCAGAGGTTGATGCTCTCTCCATGGCACAA

CATGAAAACCTCGTACCGCTGTGGGGTTACTGCATCCAGGGGAACTCAAGGTTCCTCATA

TATTCCTACATGGAGAATGGCAGCCTGGATGACTGGCTCCATAACAGAGATGATGGTGCT

AGCTCATTTCTTGACTGGCCGACACGGCTCAAGATTGCACAAGGAGCAAGCCTGGGCCTT

TCTTATATTCATGATGTCTGCGAGCCTCATATCGTCCACCGTGACATTAAATCCAGTAAC

ATCCTACTGGACAAAGAATTCAAAGCTTATGTTGCAGATTTTGGGCTAGCCAGATTGATC

CTTCCCAACAAAACTCATGTTACAACTGAGTTGGTCGGCACTATGGGTTACATCCCTCCC

GAGTACGGGCAAGCATGGGTTGCTACTTTGAGAGGTGATATATACAGTTTTGGAGTAGTC

TTGCTTGAGCTGCTCACAGGAAGCAGACCTGTTCCAGTCCTGTCTACATCAAAAGAACTT

GTCCCATGGGTTCGACAGATGAGATCTGAGGGTAAGCAGACTGAAGTCCTGGATCCAACA

CTTCGAGGAAGAGGATATGAAGAGCAAATGTTGAAAGTGCTTGAAACCGCTTGCAAGTGT

GTTGATAACAATCAATTCAGAAGGCCAGCTATCACAGAAGTTGTCTCCTCCCTGGCCAGT

ATAGAAGCTGACCCATAGATGCAAAGACCAGCCAAGATAAAATGAACTATGCATGATAGA

TAATCCTGTATTTCCGTGCATGATGTACATGTAACATTCTTAGTTTTACTAAAACTGCTA

CTTTTGATAAACATTAGAGTTTGTTTTTTACTTTGTACAGAGCCTAACAGGTTGCCATTT

TGAGCTTTACATACTTCTTTAAGGAAAGATTTTGTAGAATTCATCTAAGTTTCCTATGTT

TCTAAATTACTATACATATACGTAAGTTTCAC

>Traes_4AL_71CD6D929

CAATCAATGGGGTGCTCCAAAACCCTAGCAGCTGGCCTGCTCGCCATGCTTTTCCTAGCT

CCGGCCGTCCTGGCTACCGACCCTGACCTTCTCCAGGACTTCTGCGTCGCTGACCTCGAC

GGCAAGGCCGTCTCGGTGAATGGGCACACGTGTAAGCCCATGTCGGAGGCCAGCGACGAC

TTCCTCTTCTCATCCAAGCTGGCCAAGGCCGGCAACACATCCACCCCGAACGGCTCGGCC

GTGACGGAGCTCGACGTGGCCGAGTGGCCCGGTACCAACACACTCGGTGTGTCCATGAAC

CGCGTGGATTTTGCACCTGGAGGCACCAACCCGCCACACATCCACCCGCGTGCCACCGAG

ATCGGCATCGTGATGAAAGGTGAGCTCCTCGTGGGAATCCTTGGCAGCCTCGACTCCGGG

AACAAGCTCTACTCGAGGGTGGTGCGCGCCGGAGAGACGTTCCTCATCCCACGGGGCCTC

ATGCACTTCCAGTTCAACGTCGGTAAGACCGGGGCCTCCATGGTCGTCTCCTTCAACAGC

CAGAACCCCGGCATCGTCTTCGTGCCCCTCACGCTCTTCGGCTCCAACCCGCCCATCCCG

ACGCCGGTGCTCACCAAGGCGCTCCGGGTAGAGGCCGGGGTCGTGGAACTTCTCAAGTCC

AAGTTTGCCGCTGGGTTTTAA

>Traes_6DL_41CBE9959

CCGGCATTAAAGTGCGCCCCCCGGGATCCTCCTCGGCCCCAAACAAAACCATCCTCGTCC

CCCGCGCCCCCCCAAATCTTTCCCTTCCACTCCGGGATACCCCCTCTCGTTTCACATCGC

ATTCATCGCTCCGTCCGCTCTATCCCCCAGGCCTCGACAGCTACATCGGTCTAGCCTCGT

GTGCGCGCTGGCGCCTCGATCCTCCAGCAACTACTAGTACCAGTTGAGTTTGTTTTCCTT

GTAGTCCTCCTTCTGCTCAGGAACGCGAGGGAGATCATTACCCATGGGGAGGGCGCCGTG

CTGCGACAGGAAGGGTCTCAAGAAGGGGCCGTGGACGCCGGAGGAGGACAAACAGCTCGT

CGACTTCATCCAGGCCAACGGCCATGGCAGCTGGCGCCTGCTCCCCAAACTCGCAGAGCT

GAACCGGTGCGGCAAGAGCTGCCGGCTACGGTGGACGAACTACCTGCGGCCGGACATCAA

GCGCGGGCCCTTCACCGCCGAGGAGCAGAAGTCCATCGTCCAGCTCCACGGCATCGTCGG

GAACAAGTGGTCCATGATCGCGGCGCAGCTGCCCGGCCGGACGGACAACGAGATCAAGAA

CTACTGGAACACGCACCTCAAGAAGCAGCTCCGCCGGATGGGCCTCGACGAGCCCCCG

>Traes_5BL_6240DE6BA

ATGGATAAGGCATACATTGCCGTCCTCACCATCGCCTTCCTCTTCCTGCTCCACTACATT

CTAGGCAAGGTCAGCAATGGCAGACGCGGCAAGGGCGCCGTGCAGCTGCCGCCCAGCCCC

CCGGCCATCCCGCTCCTCGGCCACCTCCACCTCGTGGAGAATCCGTTCCATGCCGCGCTG

TGGCGCCTCGCCGCGCGCCTCGGTCCGGTCTTCTCGCTGCGCCTCGGCTCGCGCCGCGCC

GTGGTCGTCTCCTCGGCGGAGTGCGCCAGGGAGTGCTTCACGGAGCACGACGTGACGTTC

GCCAACCGGCCCCGGTTCCCCTCGCAGCTGCTCGTCTCCTTCGACGGCGCCGCGCTCGTC

ACGTCGAGCTACGGCCCGCACTGGCGCAACCTCCGCCGCGTCGCCGCCGTGCAGCTGCTC

TCCGCGCACCGCGTCGCCTGCATGTCGGGCGTCATCGCCGGCGAGGTGCGCGCCATGGCG

CGCCGGCTGTTCCGCGCCGCCGCCGCGGCCCCCGACGGCGCCGCGCGGGTCCAGCTTAAG

CGGAGGCTGTTCGAGCTCTCCCTCAGCGTGCTCATGGAGACCATCGCCCAGACCAAGGGG

ACCCGGTCGGAGGCCGACGCCGACACGGACATGTCAGTGGAGGCCCAGGAGTTCAAGAAG

GTGGTGGACGAGCTCATCCCGTACCTCGGCGCCGCCAACACGTGGGATTACCTGCCCGTG

TTGCGGTGGTTCGACGTGTTCGGCGTGAGGAACAAGATCCTGGCCGCCGTGAGCAGGAGG

GACGCATTCCTGCATCGTCTCATCGACAACGAGCGCCGGAGGCTCGACGATGCCGGCACC

GAAGGCGACAAGAAGAGCATGATCGCCGTGCTCCTCAATCTGCAGAAGACGGAGCCGGAG

GTGTACACCGATACCATGATCACGGCTCTCTGCGAGATGGGACCAGAATCCTGTCGTGGT

GTATGTGGGTTCAGTGGTCAGTGGTCACTAGCTACTGTAGCTAGCACGGTAGATAACGCC

GTATCACTTGGTTG

>Traes_4DL_8B7CD793D

CTCAATTGTCCTTGTACTCTACTTGCTGTGCGGCTGTTATGCACAAGGTTGGTTAGCTAT

CAGGATTGGTTAGGGCGCTCACTTTCAAGTAGCAGCCAGCAAAACAGCATGGCTCTTAAT

TTGGATATAAGACCTTGCGTCAAGATGACCAGCCCAATGCCTTTTAATAAAGCAGGCCTC

ACCTAGTAGTGATGACTTGACAAAAATAAAGAAAATTACACTAGGGTAACTATATAGATG

ATACTGCTGCTGCCCCTTGGTCTTGGTGCAGCCTCCAGCCTCTCTGGATTATGTGCACAT

GCCTGCTTCTCCTTGTTGTCGTCAGACCAAAATCCTGTAATAGTGGTTGATAGTTTCTGG

TTTTTTACTGCAGCTAAACCTCAAATGTTCACTTATAGATTTGTACTATGTTTGAAATCT

GTATTGGTAATATTTTGGTAATCTGATACATTGTTCTGTTGCAGCTTTGCTTGGCTTGAG

TTGGTGAGTCATAGAACCTTTATGGCAAGACTGCTGACGGGCAATTCACAAAAAGGGTGG

CCTTTTTTCCAACGACTACTTATTGATCTGTTCAAATTCATGGAACCGTATTTGAGAACT

GCCGACCTTTTAGAACCAGTGCACCTTATGTACAAAGGAACTATGAGAGTGCTGCTTGTA

CTACTCCATGACTTTCCTGAATTTCTGTGTGATTATCACTTCAGTTTTTGTGACGTGATC

CCTTCCAGTTGCATTCAAATGCGCAATGTCATTCTTAGTGCTTTTCCGCGCAACATGAGA

CTTCCAGACCCATCTACTCCTAACTTGAAGATTGATCTGCTAGCTGAAATTTCATTACCT

CCACGAATCATGTCAGATGTCGATGGTGCCCTCAAGTTAAAGCATATGAAGGTTGATGTC

GATGAGTATCTTAAGAGACCAGAAGGCTCATCCTTTTTGAATGATCTGAAGCAAAAATTG

CTGCTGCCTCAGAATGAAGCTAATGTTGCTGGGACACGCTACAATGTGCCTCTGATGAAT

TCGCTTGTTCTCTACATTGGCATCCAAGCGGTGCAACAATTGCAAGCGAACAAGGCAAAT

GCATCTGCGTCAGCACAGCAGATTAACCATAATACAATGGATATTTTTCAGATCGAAACA

GCGACTGAATTTTTCAGATATCTTGTAACGAACTTGGATACAGAGGGGCGCTACCTGTTT

TTGAATGCGATTGCTAACCAACTGCGCTACCCGAATAGCCACACACACTACTTCTCCTTC

ATCATTCTGTATCTATTTGCTGAAGCTGCCCAGGAACATATCCAGGAGCAGGTAACCAGG

GTTCTCTTAGAACGGCTGATAGTGAACCGTCCTCACCCCTGGGGATTACTCATCACCTTC

ATCGAGCTGATAAAGAACCCACGCTACAACTTTTGGAACCGAGCCTTCACACACTGCGCA

CCTGAGATTAAGCAGCTGTTTGAGTCGGTCGCAAAGTCATGCGGTGGAGGCGCTGGAAAG

GCAGTGGACGATGGCTCTGATGGCGGCCACTAATGTAGTTAGTTTCTTGTAAATGGTATA

TGGCTGCTTGTCATGGTCTGATGGGTTGACGTGCGGTTGACTTGCTGACAGATATTTGTT

TTGTATCATACTGGATATTGTGCTGTGAAGATTGTGGTGAACCCTCGAACCATCAGCAAA

TATTAGCCCTTAGGGGTACTATTCATATTATATCTGTATAAAAATCAGAATCAGGTGTTT

GTGGTAGCCGCGTCTGGACTTTGTTGTTTGTTGAAAAGTGTCTTTCAATCCCTGAGCAAT

AAACATATCATGCAACAATTGAAAAACAGTTTATTTTTTGACGGA

>Traes_4DL_9C2EC1F52

TTATAACTTCAAAGACCGGCAATCAAACCGTTACTACAAAGTGACCCTAATGAATTCTTC

AGATAGAGCAATTTGGCACCTCATACAGAAATGTTAGAATTAACATTGTGCATCTAACTG

TGGTGCAGACATGCTATCTGAGAGCAGGGTGGAGAAGCCGCATCGAATATACGTACCTCG

AGATGAGACATTTGAGGAGCTGAAACAGGGAGCCTTTATATCAGGGAGGCTCCGAGCAGT

ACTTCACACCCTAATCCCCTCGCTAATTGCATCAATCTCAGCTGACACCCACAACTTCCA

AGGCTTCCACCACGTTGACAACCTGTACAAGGAGGGTCTCAGGCTCAAGCTAGGCCTCCA

GGAGCACCTGTTTCAGAAGATACCATTTGTACAAAAGATTCAGGAGTCGAGCGAAGGGAT

GCTTCGCTATGACACACCTAGCATCCTTTCCAAGGACAAGTTTGCATGGCTCCGTGATGA

TGAGTTTGCGCGGCAAACTATTGCAGGAATAAACCCAGTTAGCATTGAGAGGCTCAAGGT

TTTCCCACCAGTGAGCAAGCTGGATCCTGCAATGTACGGTCCACCTGAATCATCAATTAC

AGAACGGCACATTTCTGGTCAGCTCAACGGACTGACAGTACAAGAGGCAATAGACATGGA

GAAGCTCTTCATTGTAGACCACCATGATGTGTACATGCCATTCTTGGACCGGATCAACGC

GATCGAGGGACGGAAAGCATACGCAACCCGGGCGATCTTCTTCCTGACTCAAGGTGGCAC

ATTGAAACCGATCGCGATTGAGCTTTCTCTTCCTCCAGCACAGTCAGGGGAGCCTCAGCC

GAGCAAGGTCCTTACCCCTGCCTGTGATGCTACATCCAACTGGATCTGGATGCTTGGCAA

GGCGCATGTCAGCTCCAATGACGCCGGTGTTCATCAGCTTGTTAATCACTGGTTGAGGAC

GCATGCTATAATGGAGCCATTCATCCTGGCGGCGCACCGGCGAATGAGCGCGATGCACCC

CATCTTCAAGCTCCTACATCCTCACACNGAATGAGCGCGATGCACCCCATCTTCAAGCTC

CTACATCCTCACATGAGGTACACGCTGGAGATCAATGCGCTTGCGCGGCAGAGCCTGATC

AACGCCGAAGGAGTGATCGAGTCCTGCTTCACCCCTGGCCCTGTCTCTGGCGAGATCAGC

TCCGCCTACTACCGCAACCACTGGCGCTTCGACCTCGAGGGCCTCCCGGCTGACCTCCTC

CGCAGGGGAGTGGCCGTGGAGGACGCCACACAGCCTCACGGCATCAGGCTTCTCATCGAG

GATTACCCTTATGCAAACGACGGGCTTCTGCTGTGGTCCGCCANAACTGGGTGGAGTCCT

ACGTTCAGCTCTACTACCCAGACGCCGGCACGGTCCAGTCCGACGACGAGCTCCAGGAGT

GGTACCACGAGTCCATCCATGTCGGCCACGCCGACCTCCGGGACGCGCCCTGGTGGCCGC

CGCTGTCCACGCCGCGTGACCTCGCCAACATCCTGACCACGCTCGTCTGGCTCGCGTCGG

CGCAGCACGCGGCGCTCAACTTCGGGCAGTACCCGCTGGGCGGCTACGTCCCGAACCGGC

CGCCGCTGATGCGGCGGCTGCTGCCGGACCCGGAGCGCGACGCGGCGGAGTACGCCATGT
[truncated: 736,717 more chars]
